# Parts-per-million level loading organocatalysed enantioselective silylation of alcohols

**DOI:** 10.1038/ncomms8512

**Published:** 2015-06-18

**Authors:** Sang Yeon Park, Ji-Woong Lee, Choong Eui Song

**Affiliations:** 1 Department of Chemistry, Sungkyunkwan University, 2066, Seobu-ro, Jangan-gu, Suwon-si, Gyeonggi-do 440-746, Korea

## Abstract

The field of organocatalysis has blossomed over the past few decades, becoming an alternative to transition-metal catalysis or even replacing the realm of transition-metal catalysis. However, a truly powerful organocatalyst with a high turnover number (TON) and turnover frequency (TOF) while retaining high enantioselectivity is yet to be discovered. Similar to metal catalysis, extremely low catalyst loading (p.p.m. or p.p.b. levels) is the ultimate goal of the organocatalysis community. Herein we report a remarkable contribution in this context: 1 p.p.m. loading of a simple 1,1′-bi-2-naphthol-based organocatalyst was enough to achieve highly enantioselective silylation reactions of alcohols. The unprecedented TONs and excellent enantioselectivity are ascribed to the robustness of the catalyst and systematic cooperative hydrogen-bonding organocatalysis in a densely confined chiral space.

## Abstract (web summary)

Organocatalytic methods typically do not approach the extremely low loadings that can be achieved with some metal catalysed processes. Here, the authors report a remarkably powerful organocatalyst capable of performing asymmetric silylations with sub parts-per-million level loadings.

## Graphical abstract

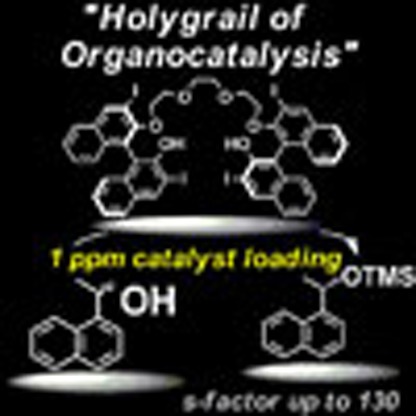

Catalysis, in principle, should offer an infinite turnover number (TON) for a desired chemical transformation by regenerating the catalyst without altering its molecular-level structure and physicochemical properties. However, limitations arise from different deactivation mechanisms and catalyst poisoning by chemical impurities and/or side products, lowering the lifetime of catalytically active species^1^.

The current strong interest in asymmetric organocatalysis^2^,^3^ has pinpointed that small organic molecules are comparable to transition-metal catalysts owing to their noble activation modes and excellent selectivity. However, further application of organocatalysts in industry is often hampered by their relatively low turnover efficiency than those of the corresponding homogeneous or heterogeneous metal-catalysed reactions^4^. Despite many efforts^4^,^5^,^6^, the current limit of catalyst loading for asymmetric organocatalysis is usually in the range of 0.1*–*1 mol% (refs 6, 7) for overriding of the non-selective background pathway. Thus, high turnover organocatalysis (for example, TON>100,000) is considered as a formidable challenge^8^.

Our group has developed a new type of easily accessible organocatalysts, 1,1′-bi-2-naphthol (BINOL)-based polyether catalysts **1** (Fig. 1), that bears phenols and polyether units, for asymmetric cation-binding catalysis^9^,^10^,^11^. Its unprecedented activation mode and remarkable enantioselectivity are explained by multiple binding and hydrogen-bonding interactions of the polyether and phenols with the metal salt to generate reactive ‘chiral' anions. Strikingly, the robustness of catalyst **1** enabled the one-pot large-scale production of unnatural α-amino acids even under harsh reaction conditions (refluxing in 6 N HCl) and the subsequent quantitative recovery of the catalyst^11^. The structural simplicity and vast application potential of the catalyst stimulated us to explore other challenging catalytic asymmetric reactions.

The preparation of enantiopure secondary alcohols is of significant interest in the pharmaceutical industry; therefore, diverse methodologies have been developed for preparing these synthons with high enantiomeric excess^12^,^13^,^14^,^15^. The kinetic resolution of racemic alcohols is an ideal approach to access both enantiomers by one enantioselective transformation, for example, acylative kinetic resolution reactions^16^. Although silylation of alcohols is one of the most common strategies in protecting alcohol functionalities, the asymmetric silylation reactions^17^,^18^,^19^,^20^,^21^,^22^,^23^,^24^,^25^,^26^,^27^,^28^ of functionally unbiased alcohols is still challenging^17^,^18^,^21^ due to the lack of additional functional groups needed to interact with a chiral catalyst. Until now, the substrate scope of the reported silylation reactions has been mostly limited to the substrates with pendant donors (for example, additional hydroxyl or pyridyl groups), which are necessary to achieve good selectivity by increasing the binding affinity towards catalysts. Snapper and Hoveyda demonstrated remarkable examples in this context by using amino-acid-derived bifunctional organocatalysts^25^,^26^,^27^,^28^. However, as a high catalyst loading (20–30 mol%) was necessary to obtain the desired products with high enantioselectivity, it may hamper the large-scale application of these organocatalysts. On the other hand, our group showed that catalysts such as **1** (X=Cl, Br or I) catalyse desilylative kinetic resolution of silyl-protected racemic alcohols in the presence of potassium fluoride. Although this approach provided a new synthetic route for chiral alcohols, a practical application was also far from satisfactory due to the high catalyst loading (20 mol%) and very low turnover frequency (TOF; 43–55% conversion after 5 days at 20 °C) (Fig. 1)^10^.

Herein we report that the same catalyst **1** can catalyse the enantioselective silylation of simple aryl alkanols, which do not contain any pendant donors, exhibiting unprecedented catalytic TONs (substrate to catalyst=up to 1,000,000) and excellent enantioselectivity. Extremely high TONs and excellent enantioselectivity were obtained by thorough catalyst structure analysis, detailed mechanistic studies and the identification of catalyst regeneration conditions.

## Results

### Reaction design

We commenced our study by selecting 1,1,1,3,3,3-hexamethyldisilazane (HMDS) as the silylating reagent (Supplementary Table 1). HMDS is a stable, commercially available and inexpensive reagent for the trimethylsilylation of alcohols, releasing ammonia as the sole by-product. However, the weak silylating ability of HMDS is the main drawback to its application^29^, particularly in asymmetric catalysis. Because HMDS is usually activated using an acidic catalyst^29^, chiral Brønsted acid catalysts were chosen first for investigation of the enantioselective silylation of alcohols with HMDS; however, these catalysts resulted in very low conversions and selectivities (Supplementary Fig. 1).

**Figure 2:**
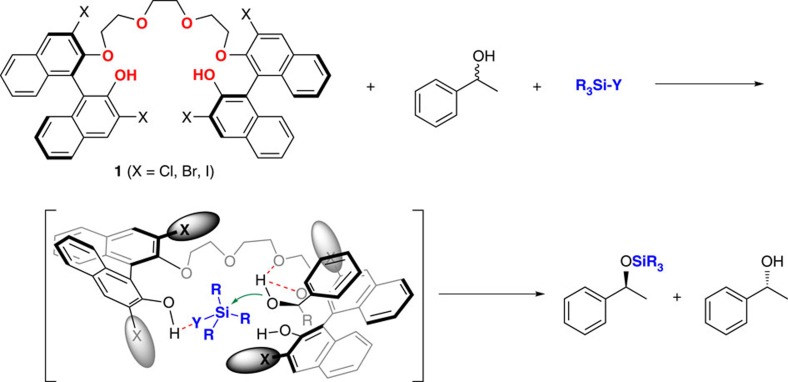
Proposed catalytic enantioselective silylation of racemic alcohols. The proposed transition state for silylation of *rac*-alcohol is shown.

Given our previous studies on catalyst **1** (refs 10, 11), in spite of the lower acidity of phenolic protons than those of the common Brønsted acid catalysts^30^, we presumed that catalyst **1** would act as a catalyst for the silylative kinetic resolution of racemic alcohols; the interaction between the acidic phenolic protons of the catalyst and the leaving group of the silylating reagent (**Y**) by hydrogen bonding would enhance the electrophilicity of Si. Moreover, the ether moieties of the catalyst would function as a Brønsted base by the association with an alcohol substrate, enhancing alcohol reactivity (Fig. 2).

### Catalyst screening

**Figure 3:**
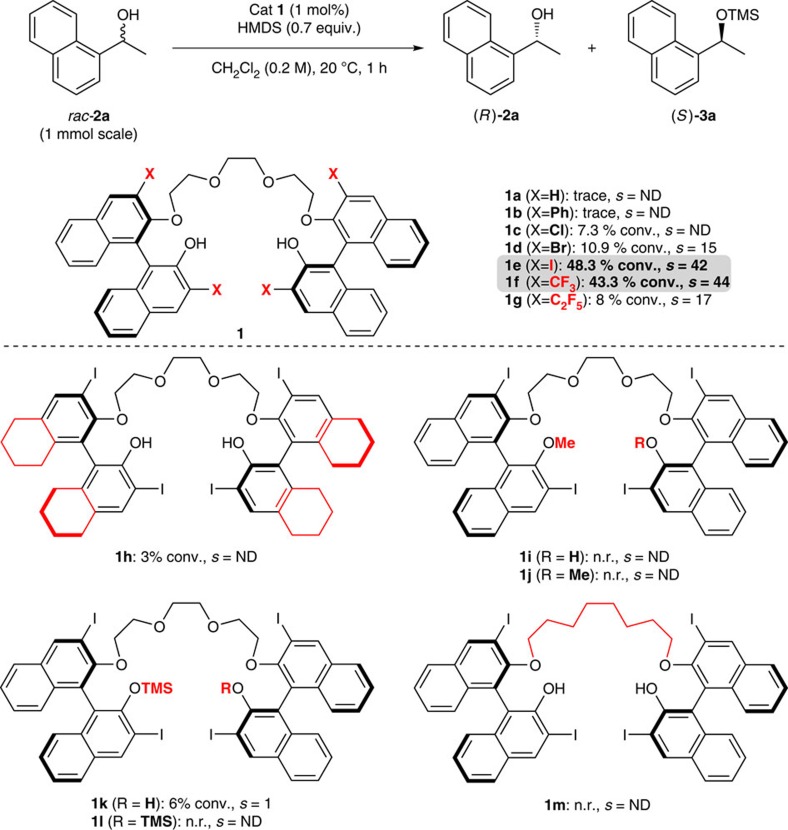
Effect of variations in catalyst structure on the silylative kinetic resolution of substrate **2a**. Calculated conversion, *c*=ee_(*R*)-**2a**_/(ee_(*R*)-**2a**_+ee_(*S*)-**3a**_). Selectivity factor (*s*)=ln((1−*c*)(1−ee_(*R*)−**2a**_))/ln((1−*c*)(1+ee_(*R*)−**2a**_))=ln(1−*c*(1+ee_(*S*)-**3a**_))/ln(1−*c*(1−ee_(*S*)−**3a**_)).

To prove this assumption, several chiral BINOL-based bis(hydroxy) polyethers **1a–1m** (Fig. 3) were screened to shed light on the relationship between the catalyst's structure and the reaction outcome. Gratifyingly, after the optimization of the reaction conditions (Supplementary Tables 1–3), catalysts **1c**, **1d**, **1e**, **1f** and **1g** bearing electron-withdrawing groups Cl, Br, I, CF_3_ and C_2_F_5_, respectively, on the 3,3′-positions showed some catalytic activity (Fig. 3). The catalytic performance of these catalysts depends significantly on the steric demand of the substituents. Thus, catalysts **1e** and **1f** show the best catalytic performance with *s*-factors of 42 and 44, respectively (Fig. 3). In contrast, other catalysts without electron-withdrawing substituents at the 3,3′-positions (catalysts **1a** and **1b**) were inactive under identical reaction conditions. In addition, the less acidic H_8_-BINOL-derived catalyst **1h** showed negligible conversion, thus, indicating the importance of the Brønsted acidity of the phenols. To further confirm the importance of an acidic phenol moiety for catalysis, we synthesized the catalysts **1i–1l**. A total loss in catalytic activity was observed when one or both phenol groups were replaced by OMe (**1i** and **1j**). Silylated catalysts (**1k** and **1l**) also showed no activity, excluding the possibility of a silyl-transfer reaction from the silylated catalyst to the starting alcohol to afford the desired product. The importance of the polyether chain was confirmed unambiguously by replacing the polyether with the alkyl chain (catalyst **1m**), indicating that the polyether backbone is crucial in achieving the observed catalytic activity.

The complexation of an alcohol substrate with the polyether moiety of the catalyst was clearly verified by observing the decreased ^13^C spin–lattice relaxation time (*T*_1_) on complexation of catalyst **1e** with racemic alcohol **2a** (Supplementary Table 4). This significant decrease in the *T*_1_ value of ether moiety of **1e** indicates that the complexation of the alcohol with catalyst **1e** significantly reduces the mobility of the ether units. Moreover, the fast-reacting (*S*)-configured substrate ((*S*)-**2a**) exhibited a more decreased *T*_1_ value of ether moiety than that of the slow-reacting substrate, (*R*)-**2a**, directly indicating a selective association of the catalyst with the fast-reacting (*S*)-**2a** (ref. 31). On the other hand, no change in *T*_1_ values was observed when alcohol **2a** was mixed with the alkyl chain-replaced catalyst **1m**, verifying the crucial role of the polyether backbone for the interaction with alcohols (Supplementary Table 5). ^29^Si nuclear magnetic resonance (NMR) experiments were also used to establish the interaction between the phenolic proton of the catalyst and HMDS (Supplementary Table 6). As expected, the ^29^Si NMR data of HMDS in the presence of diverse phenols exhibited ^29^Si chemical shifts that are shifted downfield relative to the uncoordinated HMDS, dependent on phenol acidity. This result clearly indicates that the acidic phenolic proton interacts with HMDS, increasing the electrophilic character of the silicon atom. The results from NMR spectroscopy and other experimental results (Fig. 3) strongly support our proposed transition-state model shown in Fig. 2 (Y=NHSiMe_3_).

### Optimization of the reaction conditions

More importantly, during the optimization studies, we also realized that the major deactivation pathway of the catalyst is the silylation of the phenol moieties under the reaction conditions. In a separated set of experiments, we verified that the steric demand of the 3,3′-substituents of the catalyst is crucial to this deactivation pathway (Supplementary Table 7). As mentioned earlier, the silylated catalysts (**1k** and **1l)** are inactive towards the kinetic resolution reaction (Fig. 3).

Recognizing that the rate of desilylation of trimethylsilyl (TMS)-protected phenols using KF in oligoethylene glycol^32^ is much faster than those of TMS-protected secondary alcohols, we speculated that the addition of KF and an appropriate proton source would regenerate the catalyst *in situ* (Table 1a). As predicted, the desilylation reaction of the silylated catalysts (**1k** and **1l**) and subsequent protonation of the K salt of catalysts clearly improved TON and TOF (Table 1b). As shown in entries 1 and 7 of Table 1b, with catalyst loading of 0.01 mol% (100 p.p.m.), almost no catalytic activity was observed without the additives. However, the addition of KF and a proton source such as water, *t*-BuOH and Amberlite CG 50 (a weakly acidic cation-exchange resin)^33^ was able to restore catalysis (entries 2–5). In particular, a combination of KF and Amberlite CG 50 provided the best result (entry 5). Even with 100 p.p.m. of catalyst loading, 52% conversion was obtained in 12 h with excellent selectivity (*s*=57). This can be ascribed to the adequate acidity of Amberlite CG 50 for rapid protonation of the K salt of **1e**, releasing the insoluble polymeric K salt of Amberlite CG 50 as a by-product without interfering with the catalysis. Moreover, Amberlite CG 50 alone was completely inactive towards catalysis. In addition, we also confirmed that, under the reaction condition where KF and Amberlite CG 50 were used as additives (condition in entry 5), the unwanted desilylation process that takes place with a TMS-protected secondary alcohol such as (*R*)-**3a** did not occur (Supplementary Fig. 2) and that the relative catalytic activity of **1a–1f** is very similar with that shown in Fig. 3 (Supplementary Table 8).

Further improvement of selectivity was achieved by lowering the reaction temperature to −30 °C (*s*=132) (entry 8). More surprisingly, the catalyst is active even at 1 p.p.m. catalyst loading (0.0001, mol%) with excellent *s*-factor (*s*=99 in Table 1, entry 10). The reaction proceeds with p.p.b.-level catalyst loading (100 p.p.b.); however, a significant decrease in selectivity (*s*=17) was observed (Supplementary Fig. 3). To the best of our knowledge, this is the lowest catalyst loading for metal-free organocatalysis reported to date, with excellent enantioselectivities and high TOF (TOF ∼1,000 h^−1^, entry 11) also being achieved. The high turnover efficiency can be attributed to the stability of the catalyst, its insensitivity to impurities and rapid reactivation under the reaction conditions. Notably, the reactions do not require exclusion of air and moisture, and thus solvent purification is not needed. As shown from the results of entries 6 and 9, the presence of water does not affect catalytic results, which is highly beneficial for further large-scale applications.

### Scope of the reaction

**Figure 4:**
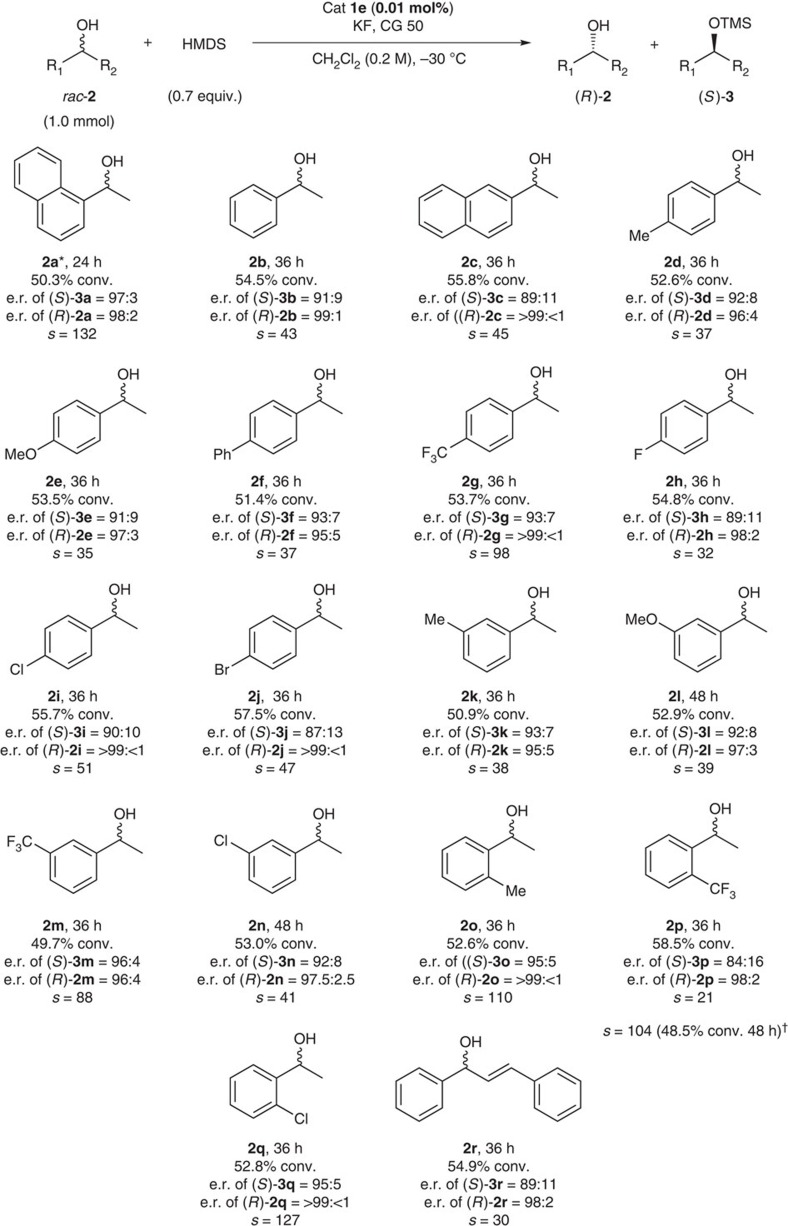
Substrate scope of the silylative kinetic resolution of racemic alcohol **2** catalysed by **1e**. The reactions were performed in the presence of 1 equiv of KF and 0.8 equiv of Amberlite CG 50 (CG 50). *Using catalyst **1c** (X=Cl, 16.8% conv., *s*=20), catalyst **1d** (X=Br, 36.7% conv., *s*=48), catalyst **1f** (X=CF_3_, 50.8% conv., *s*=50) and catalyst **1g** (X=C_2_F_5_, 34.1% conv., *s*=30). ^†^Using catalyst **1d** (X=Br). e.r., enantiomeric ratio.

With the optimized reaction conditions in hand, the substrate scope of our protocol was investigated, and the results are shown in Fig. 4. Various simple acyclic 1-arylalkanols **2a**–**2r** without any secondary binding functionality were successfully resolved with excellent selectivity factors. Both the electron-withdrawing and electron-donating substituents on the aromatic ring were tolerated under the reaction conditions. Sterically demanding *ortho*-substituted substrates were also smoothly and selectively converted to TMS-protected alcohols with excellent *s*-factors. Notably, the *s*-factors for the silylation of the sterically bulky alcohol **2p** increased from 21 to 104 by changing the catalyst **1e** (X=I) to **1d** (X=Br), a sterically less demanding catalyst, indicating that stereoselectivity can be controlled by tuning the cage size of the catalyst^34^,^35^. However, 1-cyclohexylethanol, which does not contain an aromatic moiety, exhibited a poor selectivity factor (*s*=1.4, Supplementary Fig. 4), indicating that the π–π interaction between catalyst and substrate might play an important role in obtaining high stereoselectivity, as shown in Fig. 2.

**Figure 5:**
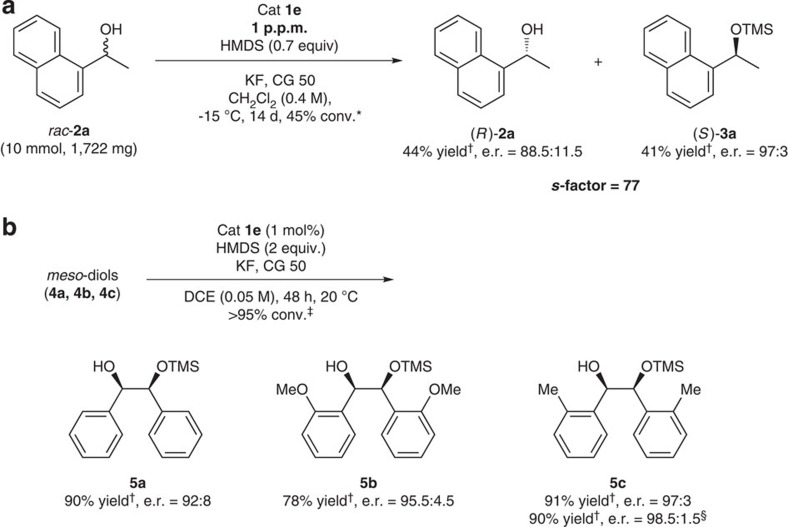
Applications of the silylation reaction protocol. (**a**) Gram-scale kinetic resolution of secondary alcohol **2a** using 1 p.p.m. of catalyst: The reactions were performed in the presence of 0.5 equiv of KF and 0.2 equiv of Amberlite CG 50 (CG 50). *Calculated conversion, *c*=ee_(*R*)−**2a**_/(ee_(*R*)−**2a**_+ee_(*S*)−**3a**_). ^†^Isolated yields. (**b**) Catalytic enantioselective silylation of *meso*-diols **4** (0.1 mmol scale): The reactions were performed in the presence of 1 equiv of KF and 0.6 equiv of Amberlite CG 50 (CG 50). ^‡^The conversion was determined by ^1^H-NMR. ^†^Isolated yields. ^§^The reaction was performed at 0 °C. DCE, 1,2-dichloroethane; e.r., enantiomeric ratio.

To demonstrate the synthetic utility and potential large-scale applications of this protocol, 1.722 g (10 mmol) *rac*-**2a** was resolved at −15 °C by using only 0.012 mg of the catalyst (1 p.p.m. to *rac*-**2a**), with a high TOF (1,309 h^−1^) and an excellent *s*-factor of 77 (Fig. 5a), affording TMS-ether (*S*)-**3a** (41% yield) and (*R*)-**2a** (44% yield) after simple filtration of insoluble additives (KF and CG 50) and column chromatography. Finally, to demonstrate the generality of our silylation protocol, the silylative desymmetrization reactions of *meso*-hydrobenzoins **4** were conducted using 1 mol% of catalyst **1e** at room temperature. As shown in Fig. 5b, monosilylated alcohols **5a–5c** were obtained in high yields and with excellent enantiomeric excesses (ee) (up to 97% ee). Furthermore, an essentially linear relationship between the optical purity of the catalyst and enantioselectivity of the major product **5a** was observed, indicating that the reaction involves only one catalyst in the enantio-determining step, supporting our proposed model shown in Fig. 2 (Supplementary Fig. 5).

## Discussion

In summary, a p.p.m.-level loading organocatalytic enantioselective silylation of simple alcohols was developed using the easily accessible BINOL-based polyether catalyst with the commercially available HMDS as the silylating reagent. This unprecedented catalyst turnover was achieved by adding KF and a weakly acidic cation-exchange resin, thus regenerating the active form of the catalyst. Using this protocol, diverse racemic secondary alcohols were resolved with excellent selectivity factors under mild reaction conditions and the reaction was conducted without protecting moisture and without the use of an inert gas atmosphere. Moreover, the utility of this silylation protocol was further extended to the enantioselective silylation of *meso*-diols. The features of very low catalyst loading, high enantioselectivity, broad substrate scope and mild reaction conditions can make this protocol easily adaptable to the practical synthesis of diverse biologically and pharmaceutically important chiral alcohols. The extremely high turnover efficiency and selectivity achieved can be ascribed to the robustness of the catalyst and systematic cooperative hydrogen-bonding catalysis in a densely confined chiral space^34^,^35^, which mimics the action of enzymes. In a confined space, active sites activate both the reactants simultaneously and keep them in close proximity, thus enhancing reactivity and efficiently transferring the stereochemical information. Our results may contribute to the development of an ‘ideal' catalyst system based on weak interactions (hydrogen bondings, charge–charge interactions and π-interactions) such those of enzymes.

## Methods

### General procedure for catalytic asymmetric silylation of secondary alcohols

To a solution of chiral catalyst **1e** (0.12 mg, 0.01 mol%) and secondary alcohols **2** (1.0 mmol) in distilled CH_2_Cl_2_ (5.0 ml), spray-dried KF (58.1 mg, 100 mol%) and Amberlite CG 50 (80 mg, 0.8 equiv.) were added in one portion. The reaction mixture was stirred at −30 °C for 10 min, followed by addition of HMDS (0.15 ml, 0.7 equiv.). After stirring for 24–48 h at −30 °C, the reaction mixture was filtered and the filtrate was concentrated *in vacuo* to afford a colourless oil. The residue thus obtained was purified by short silica gel chromatography (EA/hexanes=1/5). Silyl ethers **3a***–***3f, 3i***–***3j, 3m***–***3o** and **3r** are known compounds in the literature and their spectroscopic data were consistent with previously reported values (Supplementary Methods and Supplementary References).

The enantiomeric excesses of **2** and **3** were determined by chiral high-performance liquid chromatography (HPLC) analysis. The enatiomeric excess of TMS-ether products **3** was determined after converting them to their corresponding alcohols **2** by the deprotection of its trimethylsilyl group with TBAF. Thus, the absolute configurations of **3** were determined by comparison of the retention time of HPLC with the literature data of the corresponding alcohols **2a**–**2r** (Supplementary Methods and Supplementary References).

For NMR and HPLC analysis of the compounds in this article, see Supplementary Fig. 6–20.

## Additional information

**How to cite this article**: Park, S. Y. *et al.* Parts-per-million level loading organocatalysed enantioselective silylation of alcohols. *Nat. Commun.* 6:7512 doi: 10.1038/ncomms8512 (2015).

## Supplementary Material

Supplementary Information Supplementary Figures 1-20, Supplementary Tables 1-8, Supplementary Methods and Supplementary References

## Figures and Tables

**Figure 1:**
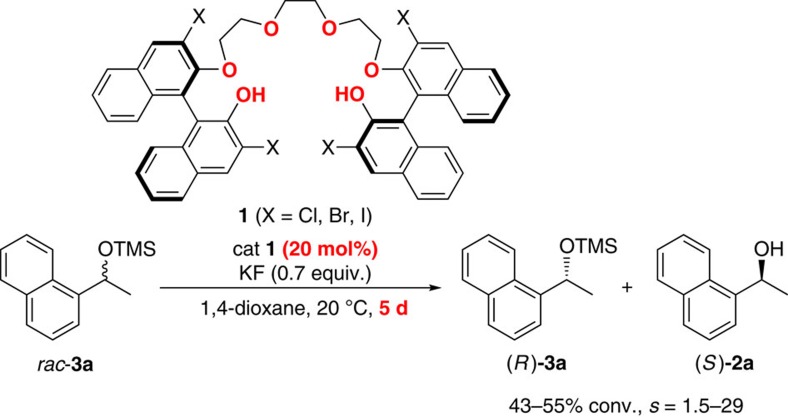
Structure of BINOL-based polyether catalyst 1 and our previous work: desilylative kinetic resolution of racemic alcohols. Selectivity factor (*s*)=(rate of fast reacting enantiomer)/(rate of slow reacting enantiomer).

**Table 1:**
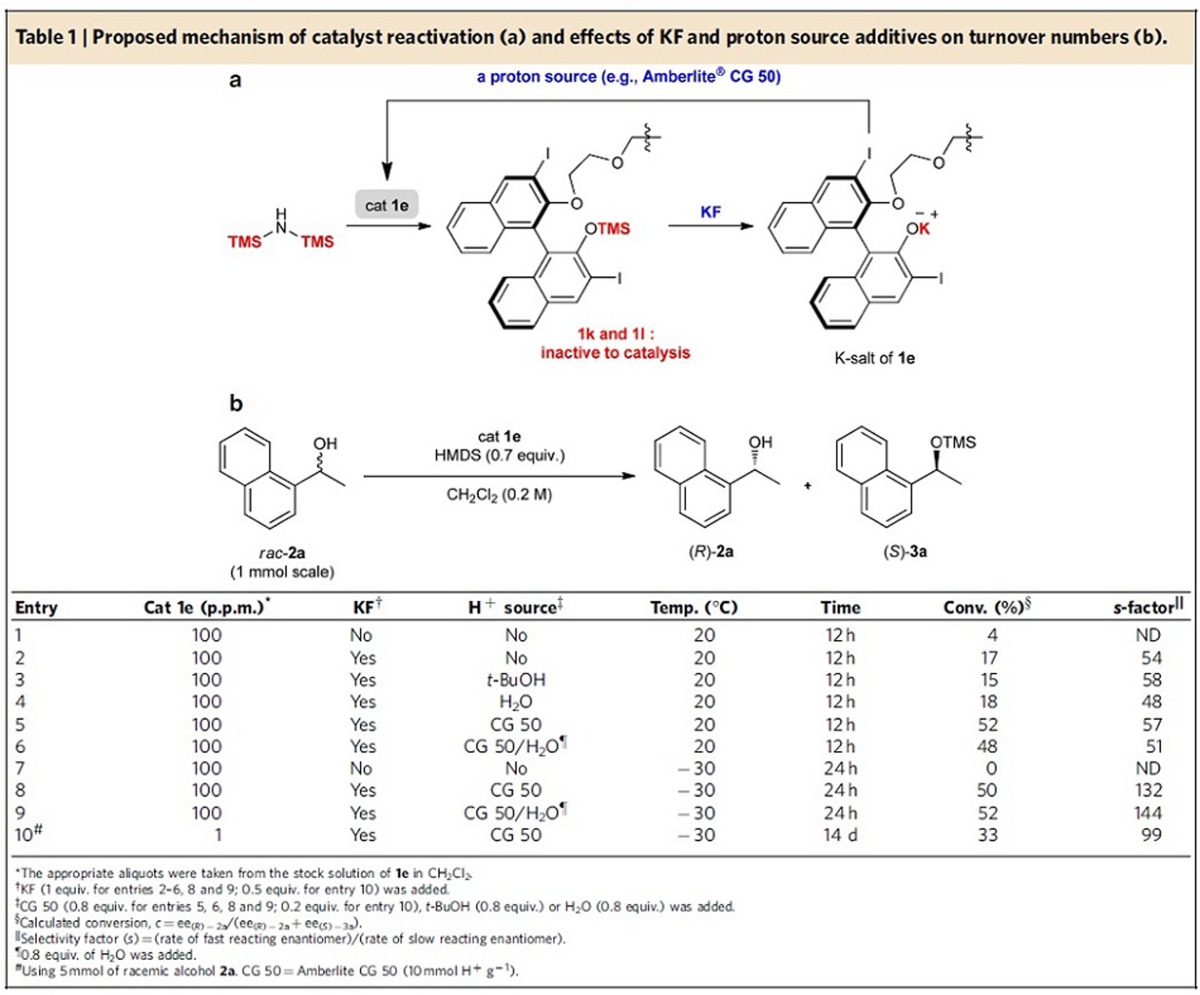
Proposed mechanism of catalyst reactivation (a) and effects of KF and proton source additives on turnover numbers (b).
