# Supplementary material for: Parts-per-million level loading organocatalysed enantioselective silylation of alcohols
Source: Nat Commun. 2015 Jun 18;6:7512. doi: 10.1038/ncomms8512 (PMC4557389; doi:10.1038/ncomms8512)
Supplement: Supplementary Information — Supplementary Figures 1-20, Supplementary Tables 1-8, Supplementary Methods and Supplementary References [file ncomms8512-s1.pdf]

Supplementary Figures

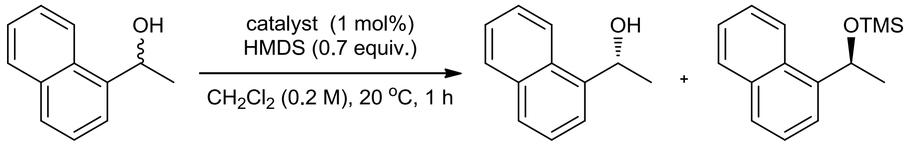

rac-**2a** (*R*)-**2a** (*S*)-**3a**

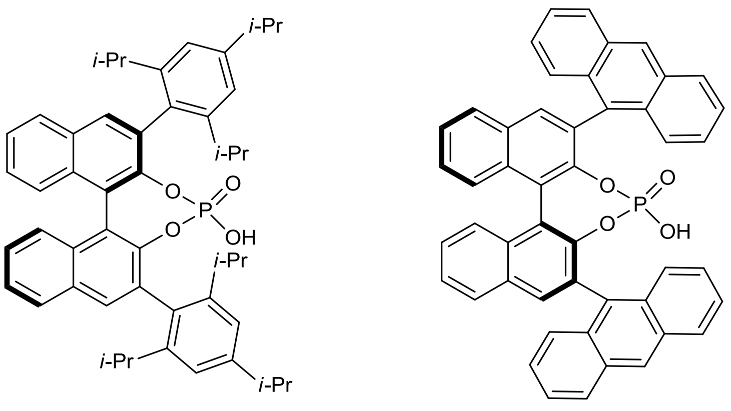

(*R*)-(2,4,5-*i*-Pr-Ph)-Phos
11.1% conv., *s* = 1.7

(*S*)-(9-Antracenyl)-Phos
6.2% conv., *s* = 4.3

Supplementary Figure 1. **Initial screening of chiral Brønsted acids as catalyst candidates for silylative kinetic resolution of substrate 2a.** The reactions were conducted with 0.2 mmol of *rac*-**2a**. Selectivity factors (*s*) were calculated according to Kagan's equation (s = ln[(1−conv.)(1+$ee_{(S)\text{-}3a}$] / ln[(1−conv.)(1−$ee_{(S)\text{-}3a}$])[1]. The conversions were monitored by ^{1}H NMR analysis and calculated by the following equation: $c = ee_{rsm}/(ee_{prod}+ee_{rsm})$. We presumed that a Brønsted acid moiety could catalyze the silylation reaction by protonating the amine of HMDS, however, we observed only low conversion of the starting material with disappointing enantioselectivities (See above). The lack of a proper binding functional group in a secondary alcohol promoted us to explore more sterically demanding hydrogen bonding donor catalyst, such as catalyst **1**.

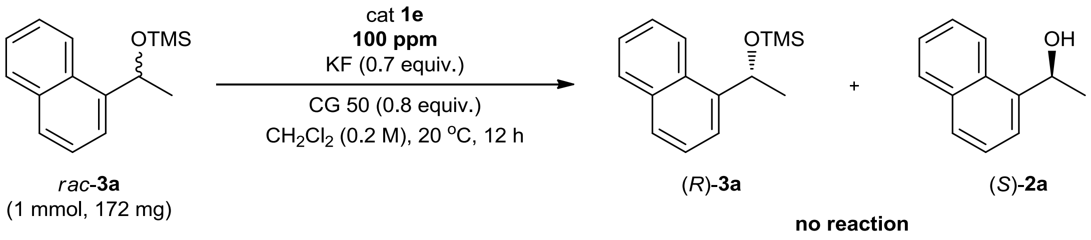

Supplementary Figure 2. Desilylation reaction of TMS-protected secondary alcohol with KF and Amberlite® CG 50 as additives. Under the reaction condition (0.01 mol% catalyst loading) using KF and Amberlite® CG 50 as additives, the desilylation process of a TMS-protected secondary alcohol such as (*R*)-**3a** did not occur. As we reported[2], the rate of the desilylation of TMS-protected secondary alcohols is much slower than those of TMS-protected phenols.

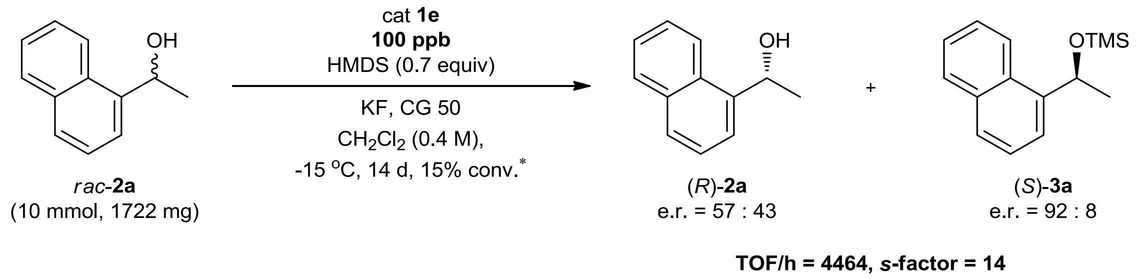

Supplementary Figure 3. Silylative kinetic resolution of substrate 2a with ppb-level catalyst loading (100 ppb). The reactions were performed in the presence of 1 equiv of KF and 0.8 equiv of Amberlite® CG 50 (CG 50). *Calculated conversion, $c = ee_{(R)\text{-}2a}/(ee_{(R)\text{-}2a} + ee_{(S)\text{-}3a})$. e.r. = enantiomeric ratio. We applied our catalytic system to gram-scale enantioselective kinetic resolution with ppb-level catalyst loading (100 ppb). Even at ppb level catalyst loading, the catalyst was still highly active (TOF/h, 4464), and thus the racemic alcohol was resolved with a reasonable *s*-factor (*s* = 14).

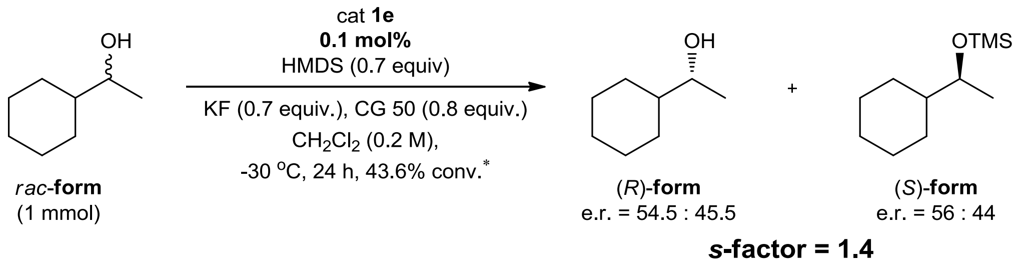

Supplementary Figure 4. Silylative kinetic resolution of 1-cyclohexylethanol. *Calculated conversion, c = $ee_{(R)\text{-2a}}/(ee_{(R)\text{-2a}} + ee_{(S)\text{-3a}})$. e.r. = enantiomeric ratio. Comments: The absolute configuration of silylether product was established to be (*S*)-form by its conversion to the corresponding (*S*)-1-cyclohexylethyl-2-naphthoate. The chiral HPLC condition: Chiralcel OD-H, Hexane/IPA = 98/2, flow rate: 0.7 mL/min, 220 nm, t_R = 7.5 min, t_S = 8.2 min.

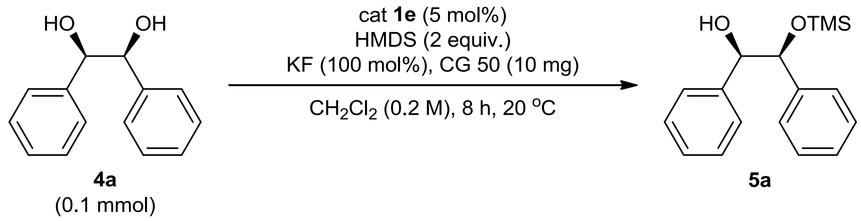

ee of cat **1e** [%]	0	27	52	75	100
ee of **5a** [%]	0	22	40	61	81

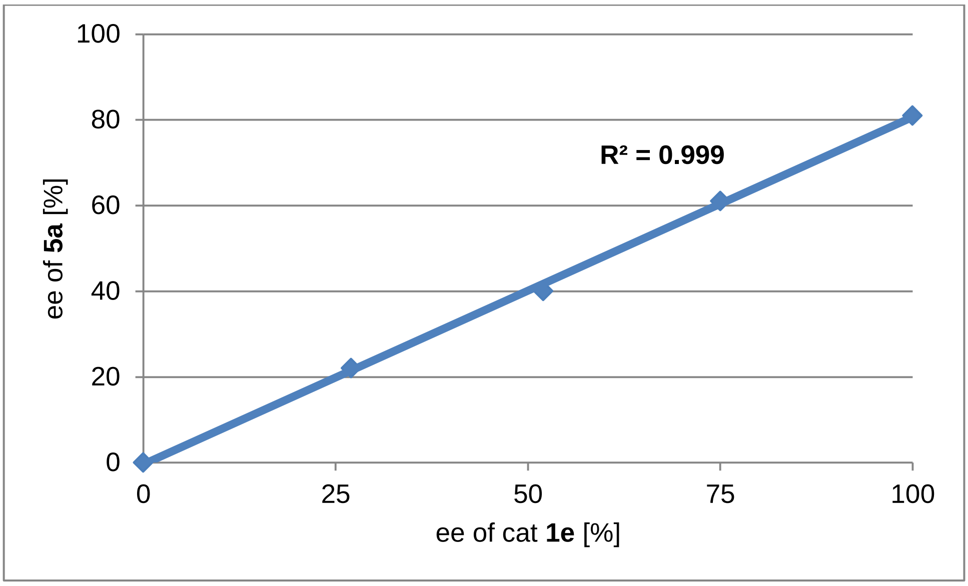

Supplementary Figure 5. Study of the effect of catalyst optical purity on product optical purity. To verify reaction mechanism, we confirmed that there is no non-linear effects during the catalysis. This indicates that there is only single chiral catalyst involved in the enantio-determining step of the silylation reaction. Although many polyether-based catalysts can form complex dimer or oligomers in the presence of metal salts, our system showed essentially linear relationship between enantiopurity of catalyst **1e** and enantioselectivity of product **5a**.

Supplementary Figure 6. ^{1}H, ^{13}C and ^{19}F NMR spectra of catalyst **1f**

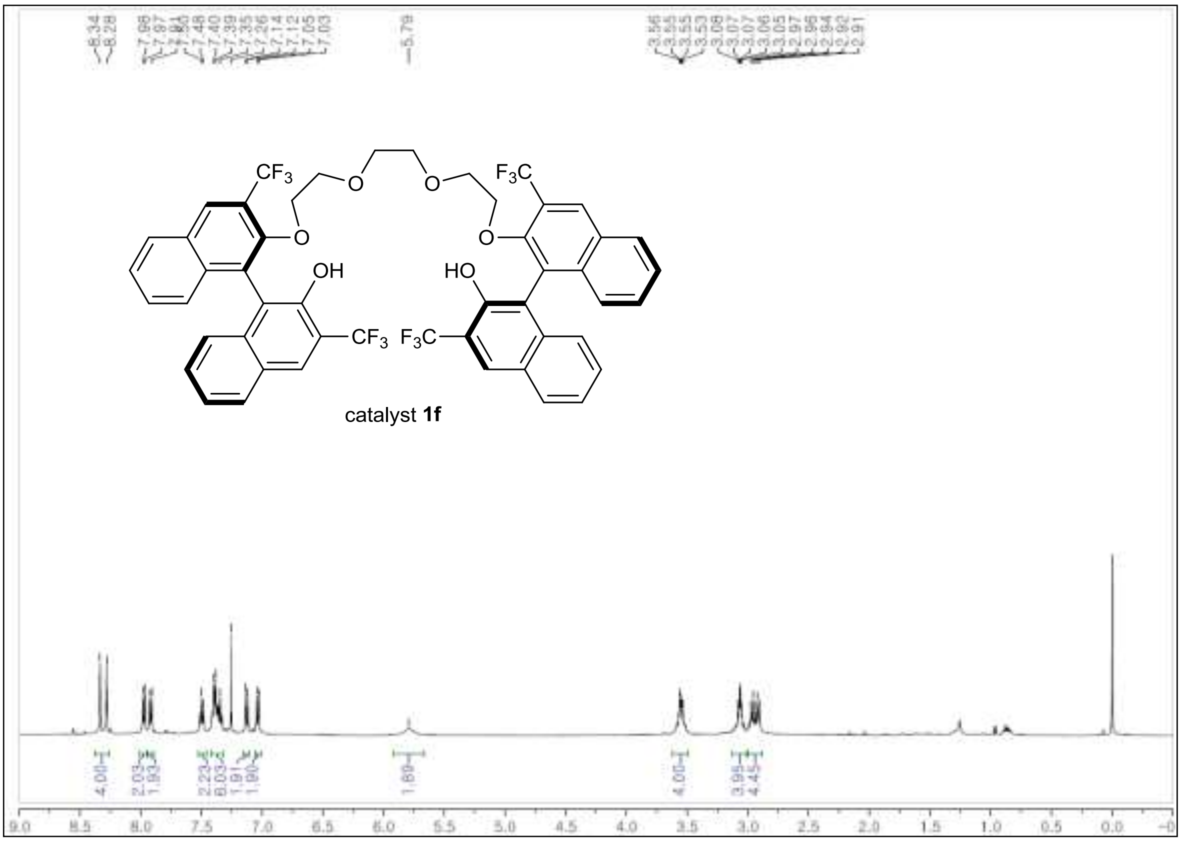

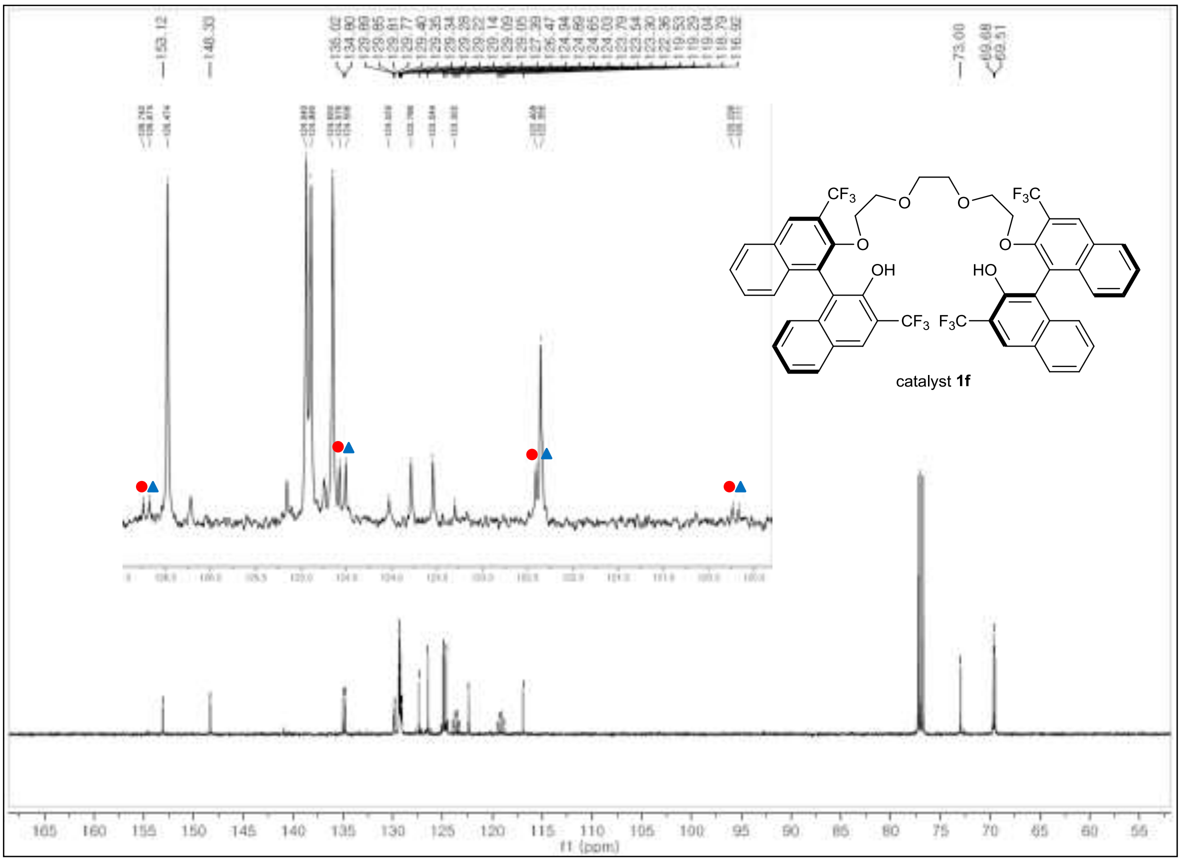

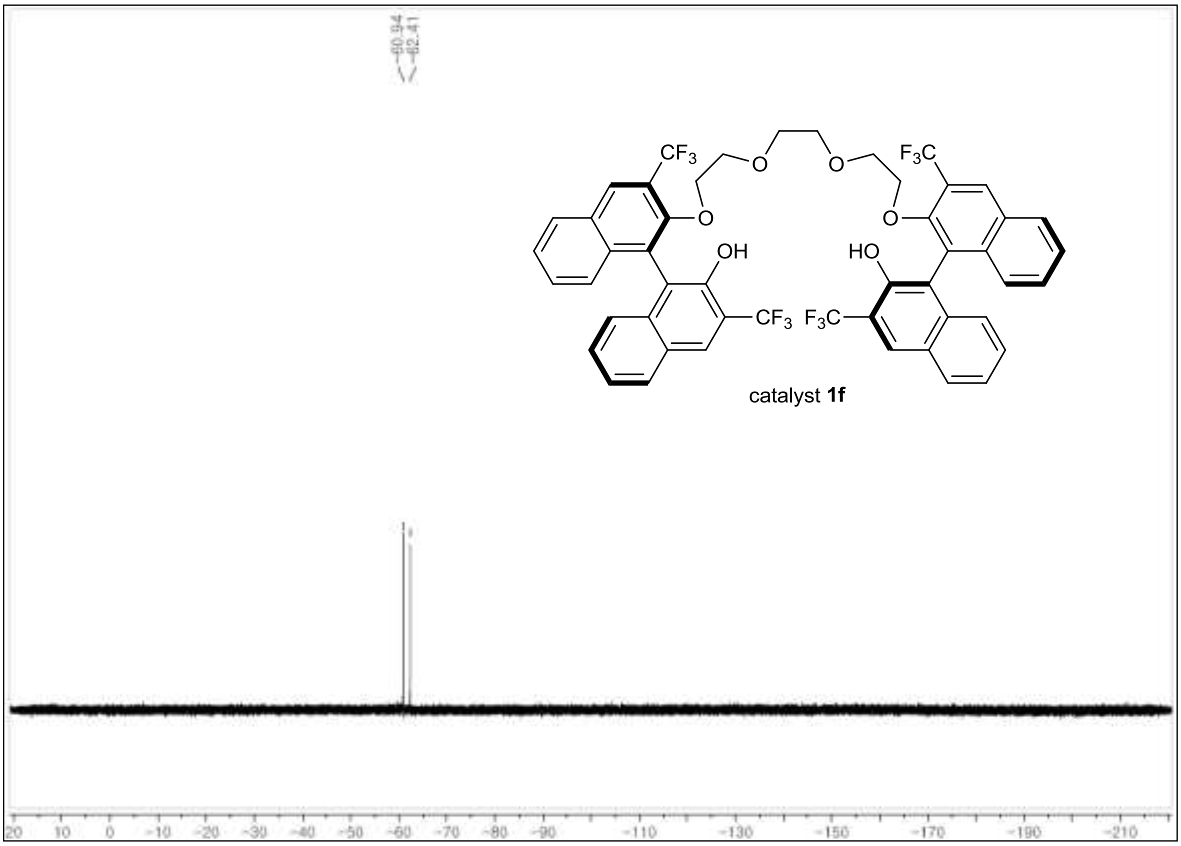
-60.94
-62.41
CF3
O
O
F3C
O
O
OH
HO
CF3
F3C
catalyst 1f
20
10
0
-10
-20
-30
-40
-50
-60
-70
-80
-90
-110
-130
-150
-170
-190
-210

Supplementary Figure 7. ^{1}H, ^{13}C and ^{19}F NMR spectra of catalyst **1g**

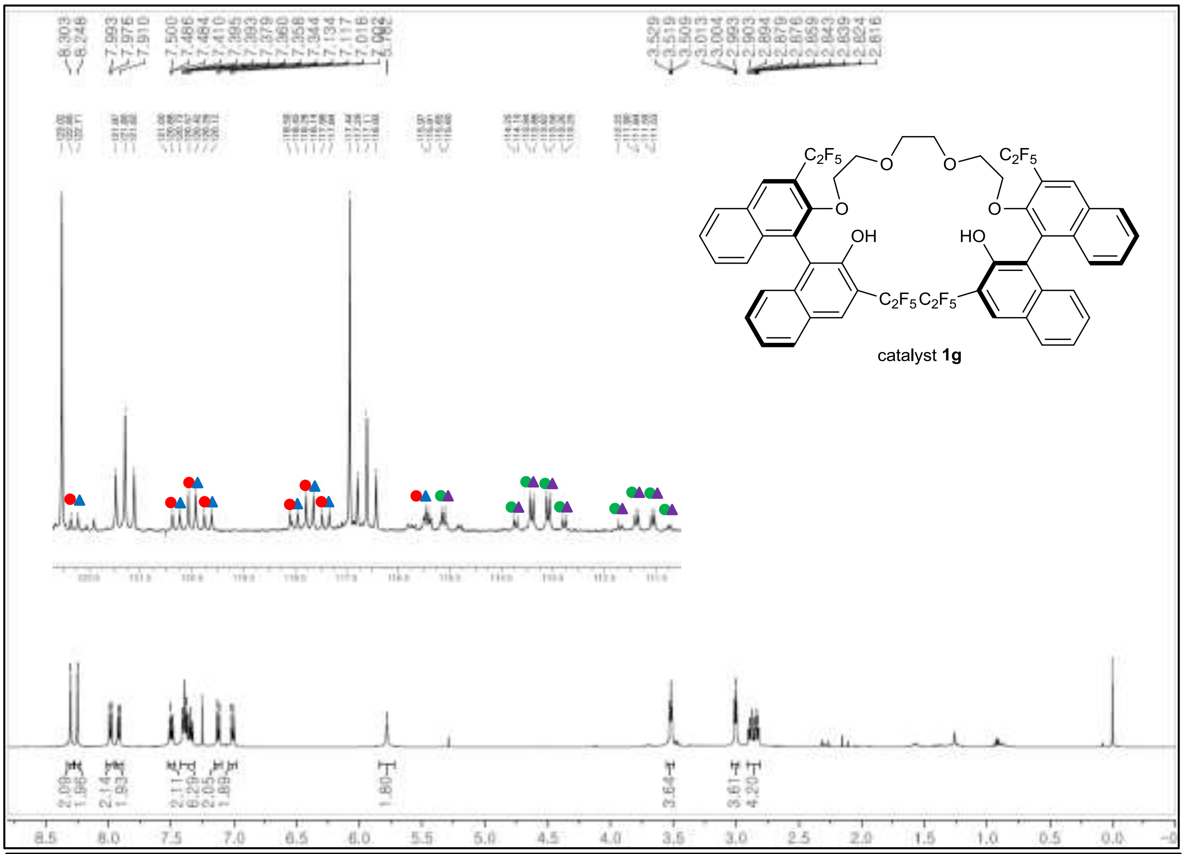

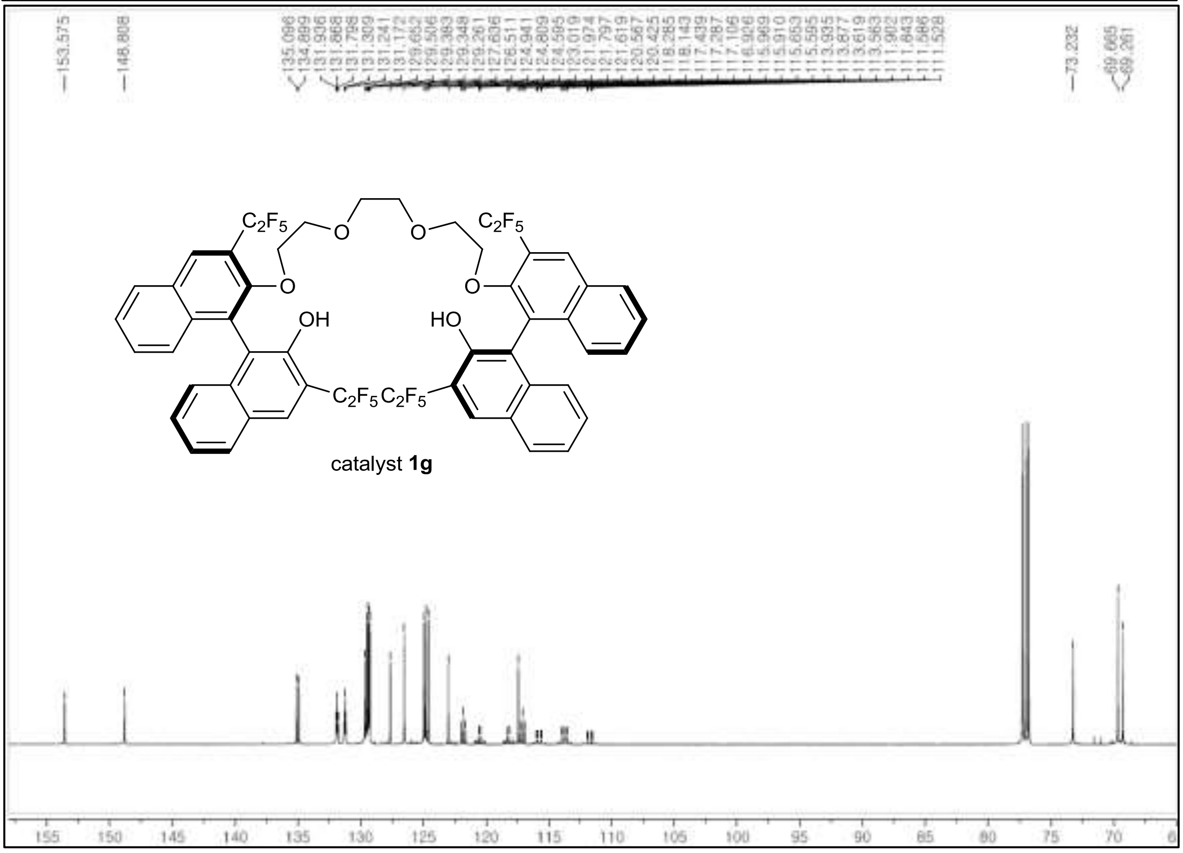

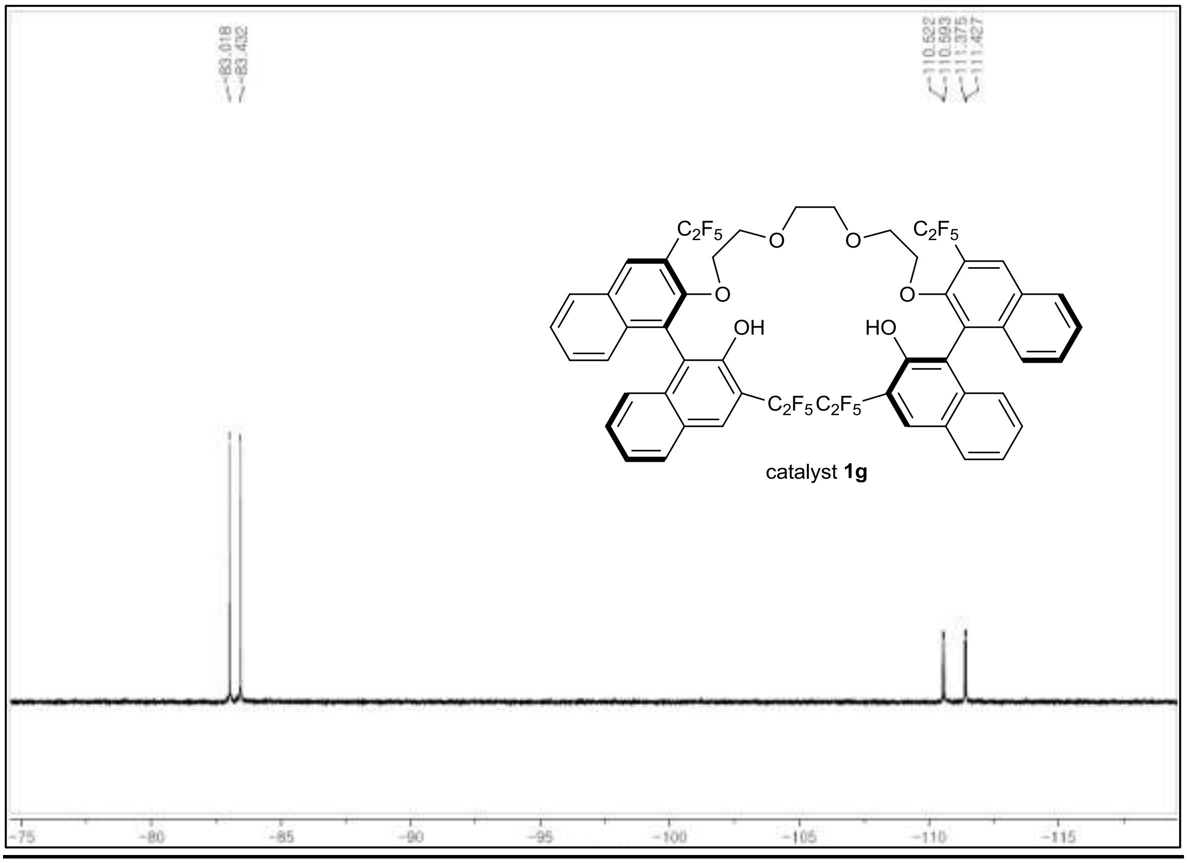

-83.018
-83.432
-110.522
-110.593
-111.375
-111.427
C2F5
O
O
C2F5
O
O
OH
HO
C2F5C2F5
catalyst 1g
-75
-80
-85
-90
-95
-100
-105
-110
-115

Supplementary Figure 8. ^{1}H and ^{13}C NMR spectra of catalyst **1h**

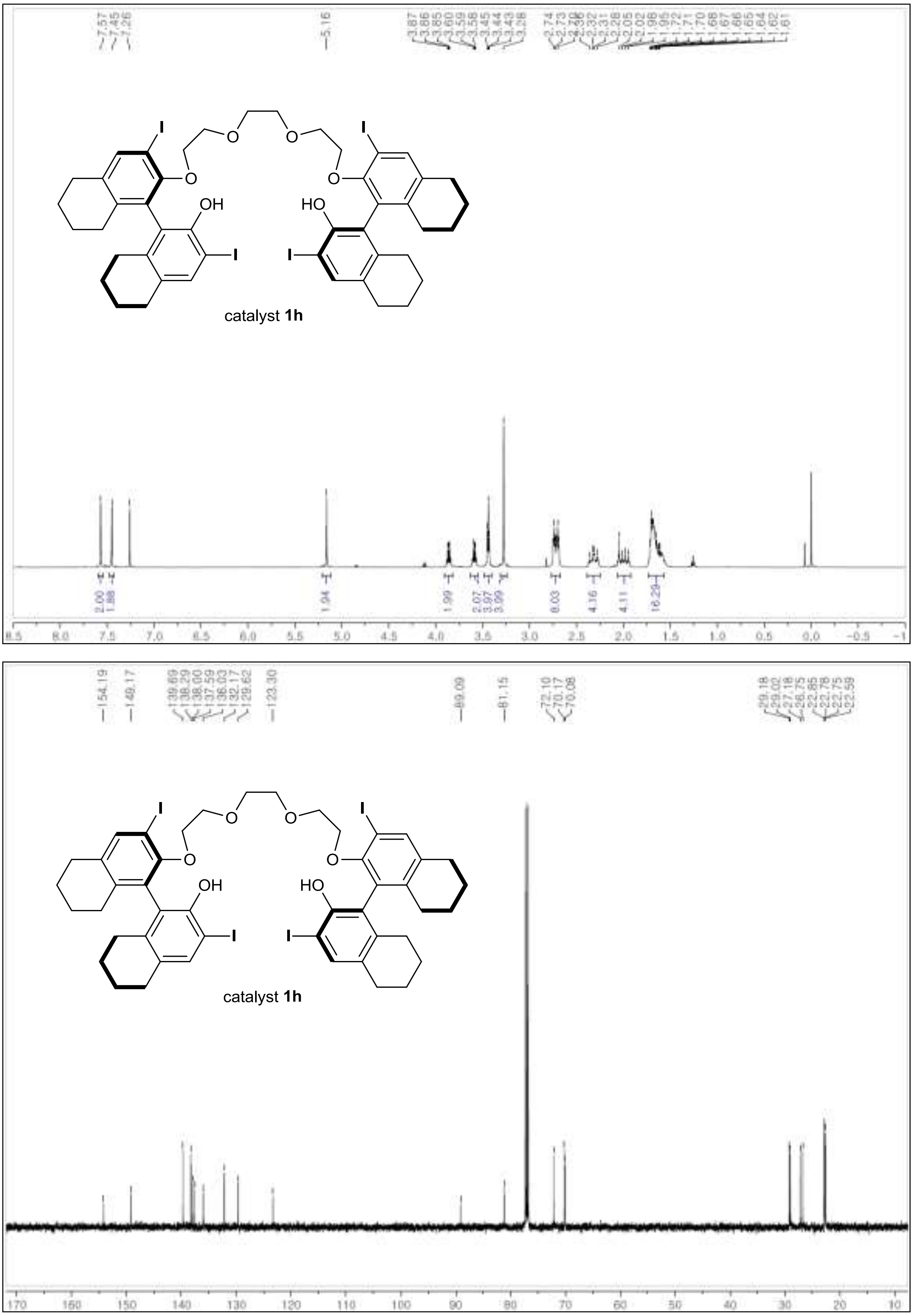

Supplementary Figure 9. ^{1}H, ^{13}C and ^{29}Si NMR spectra of catalyst **1k**

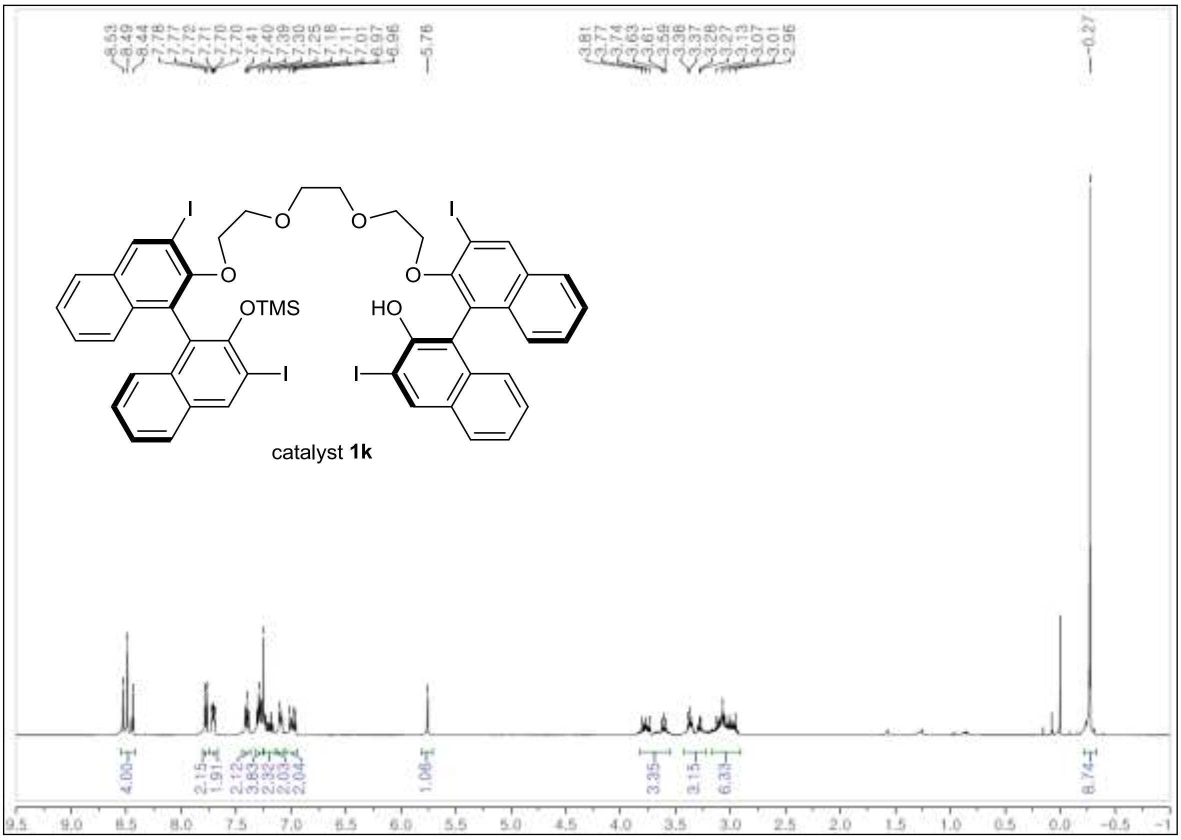

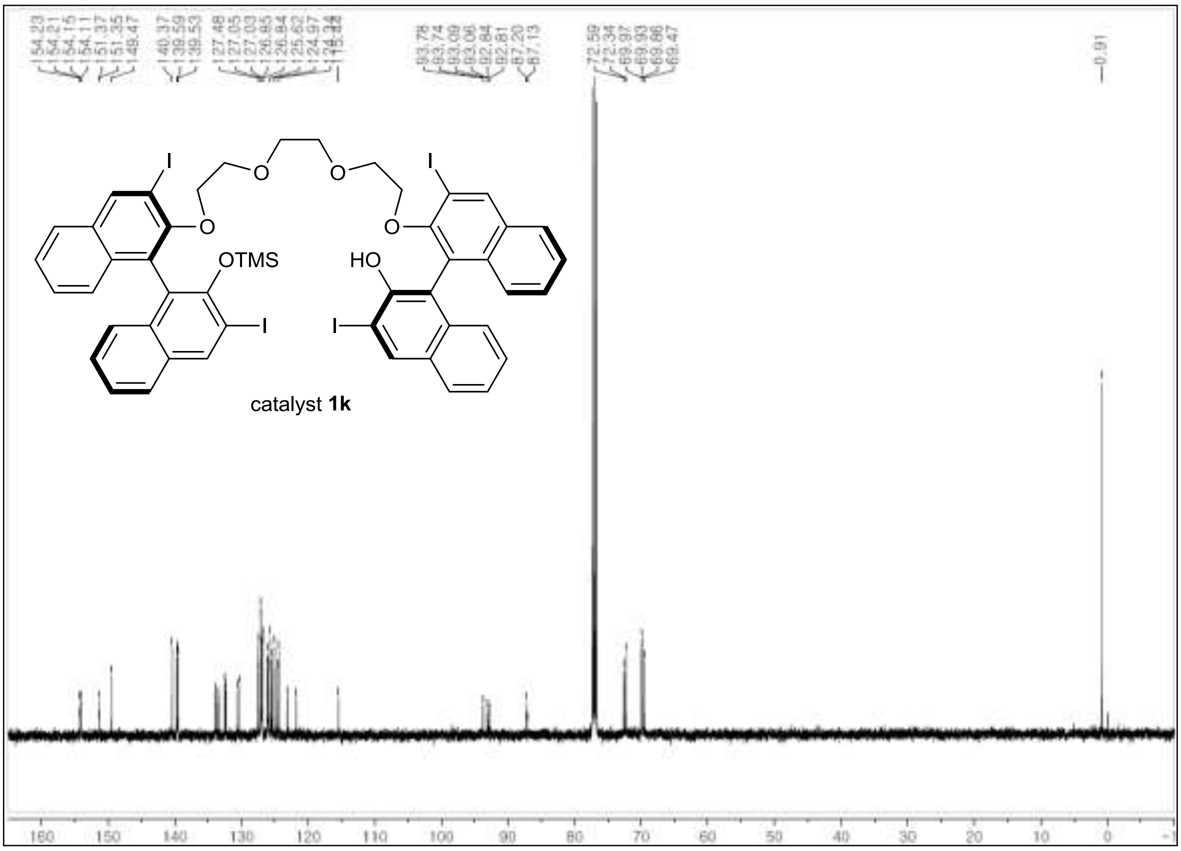

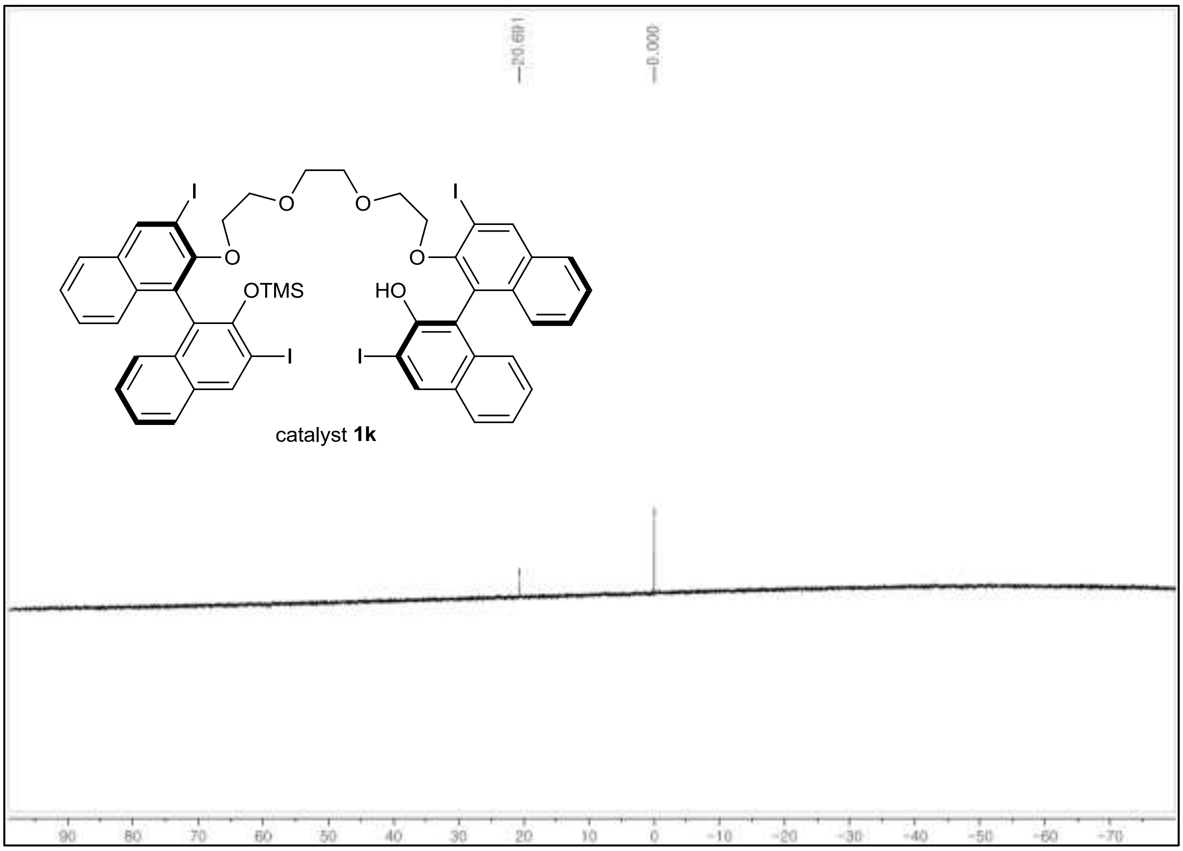

20.691
0.000
I
O
O
O
O
I
OTMS
HO
I
I
catalyst **1k**
90
80
70
60
50
40
30
20
10
0
-10
-20
-30
-40
-50
-60
-70

Supplementary Figure 10. ^{1}H, ^{13}C and ^{29}Si NMR spectra of catalyst **1l**

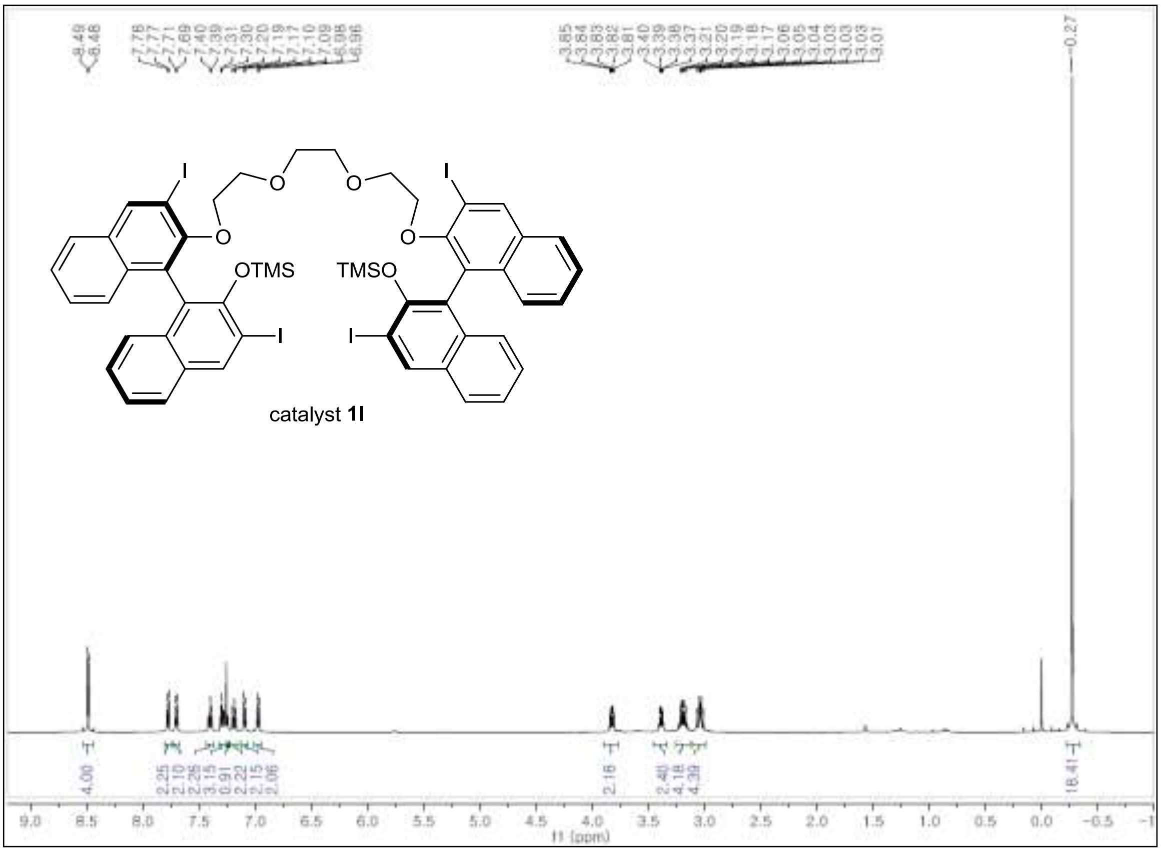

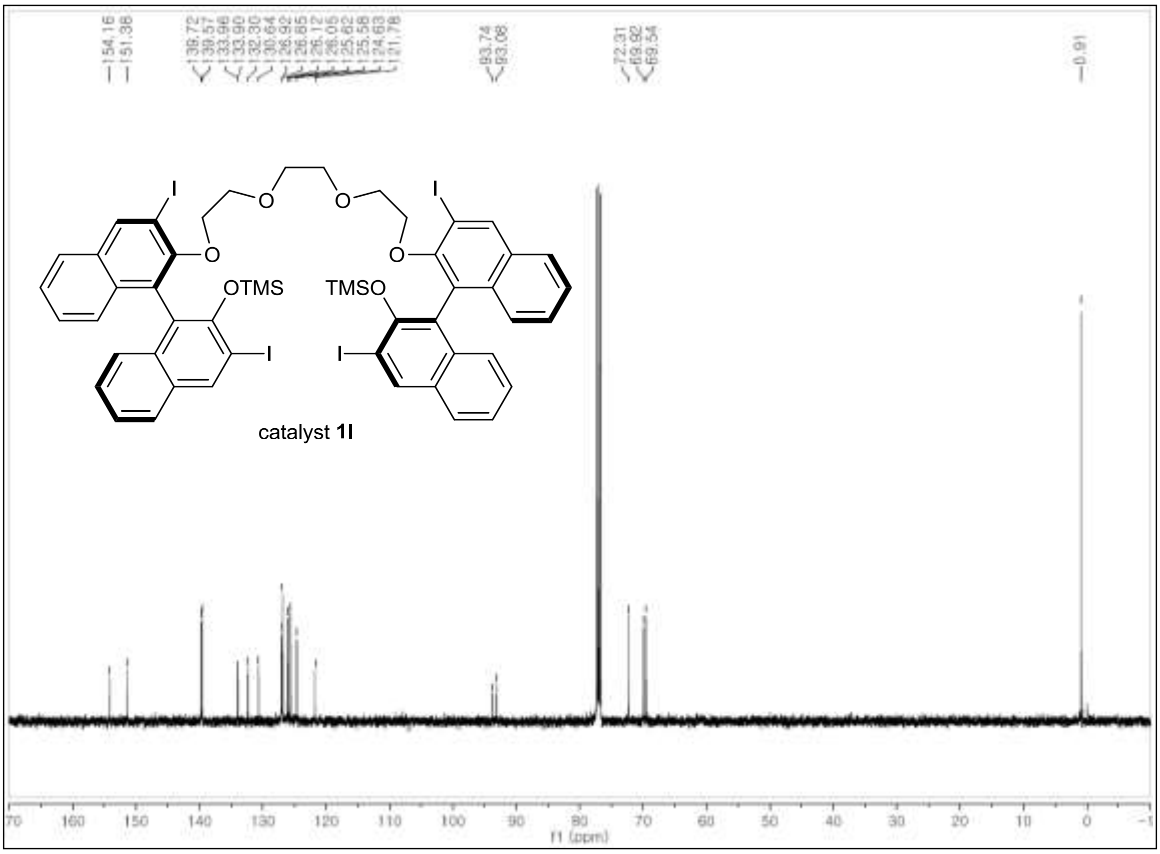

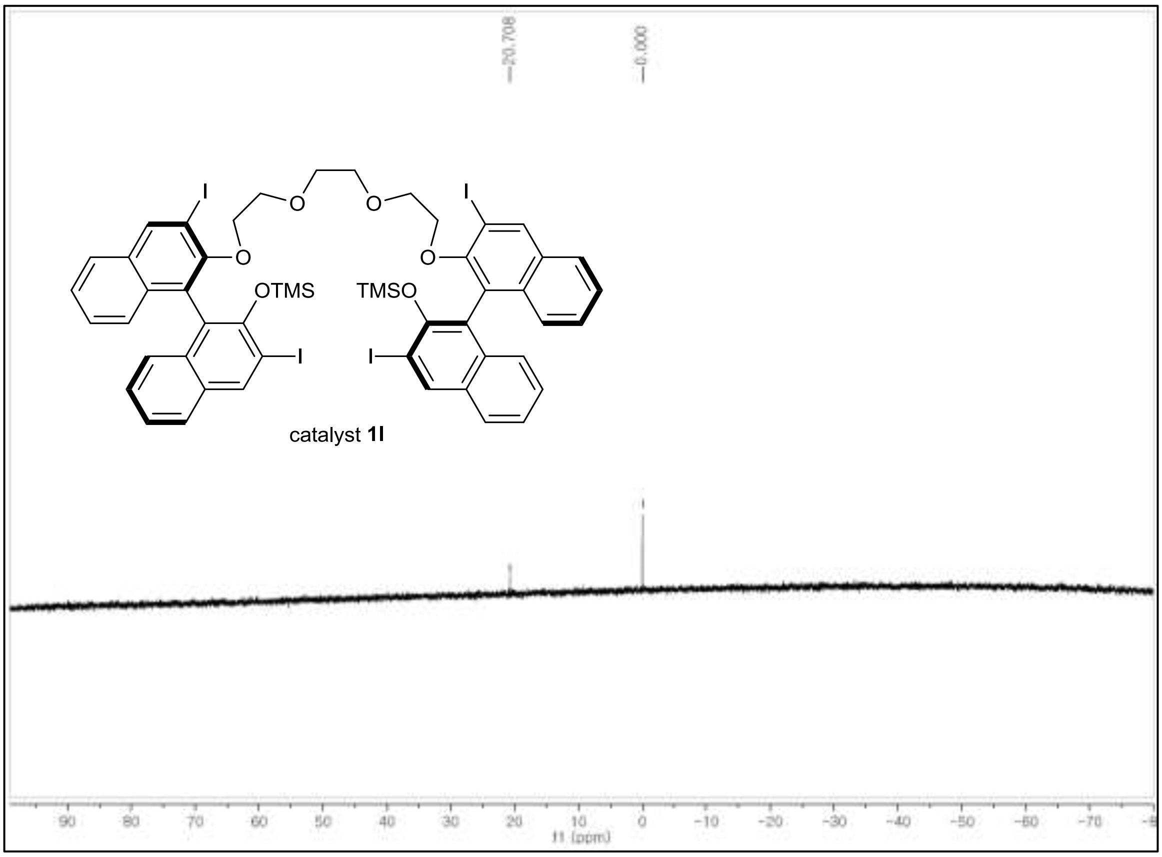

—20.708
—0.000
OTMS
TMSO
catalyst 1l
90 80 70 60 50 40 30 20 10 0 -10 -20 -30 -40 -50 -60 -70
f1 (ppm)

Supplementary Figure 11. ^{1}H and ^{13}C NMR spectra of catalyst **1m**

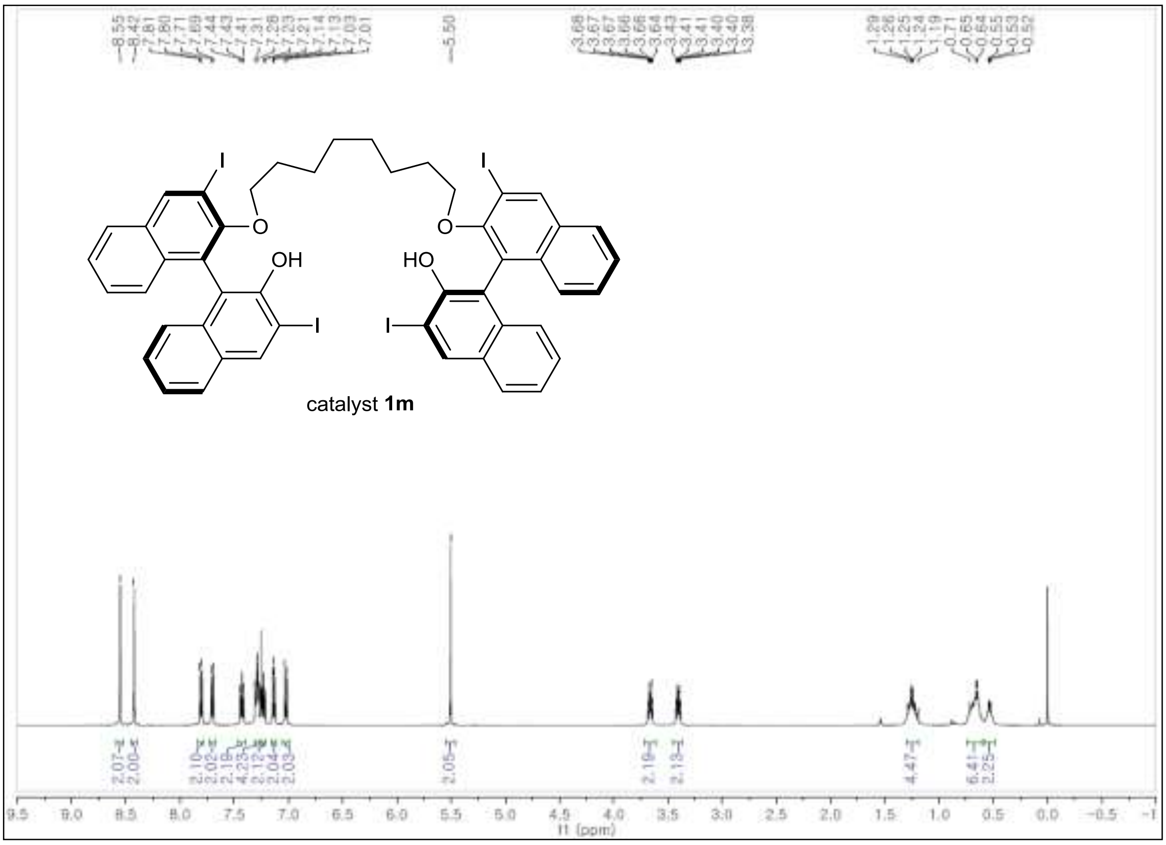

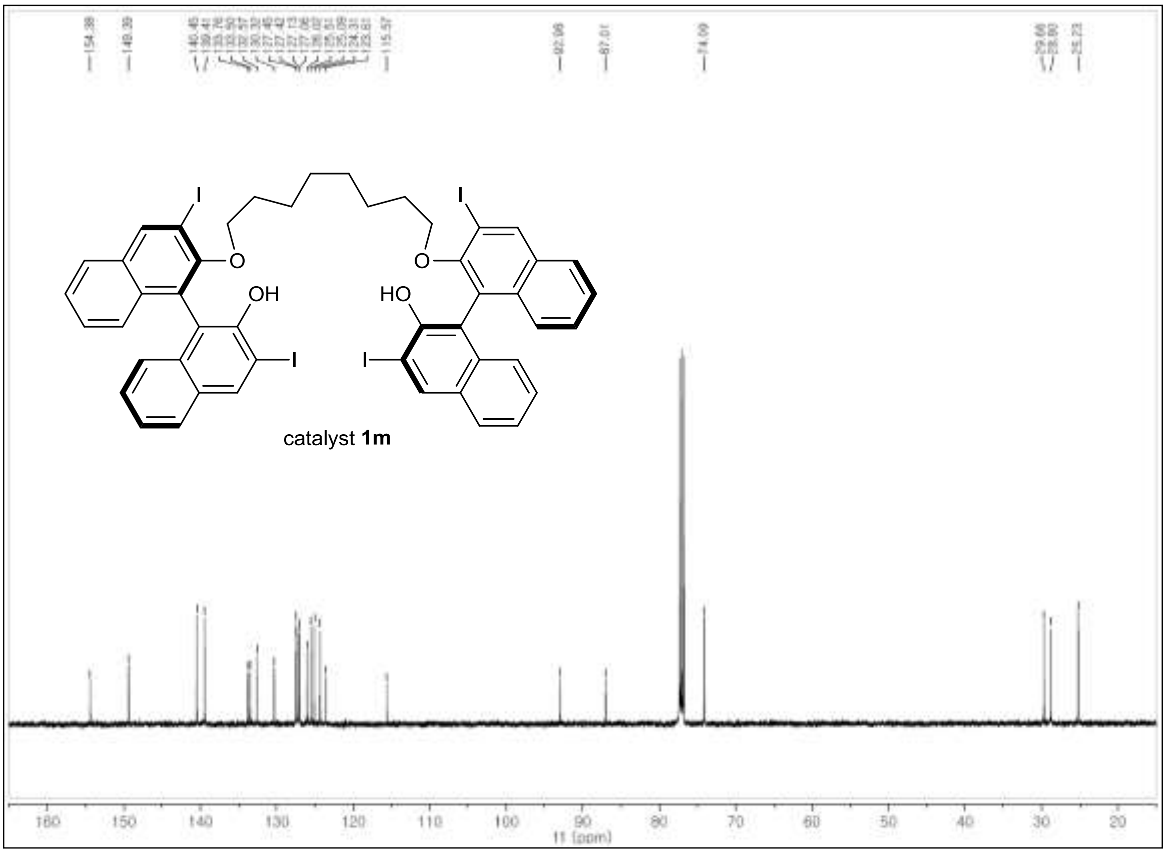

Supplementary Figure 12. ^{1}H, ^{13}C and ^{19}F NMR spectra of the remaining alcohols **2a–2r**

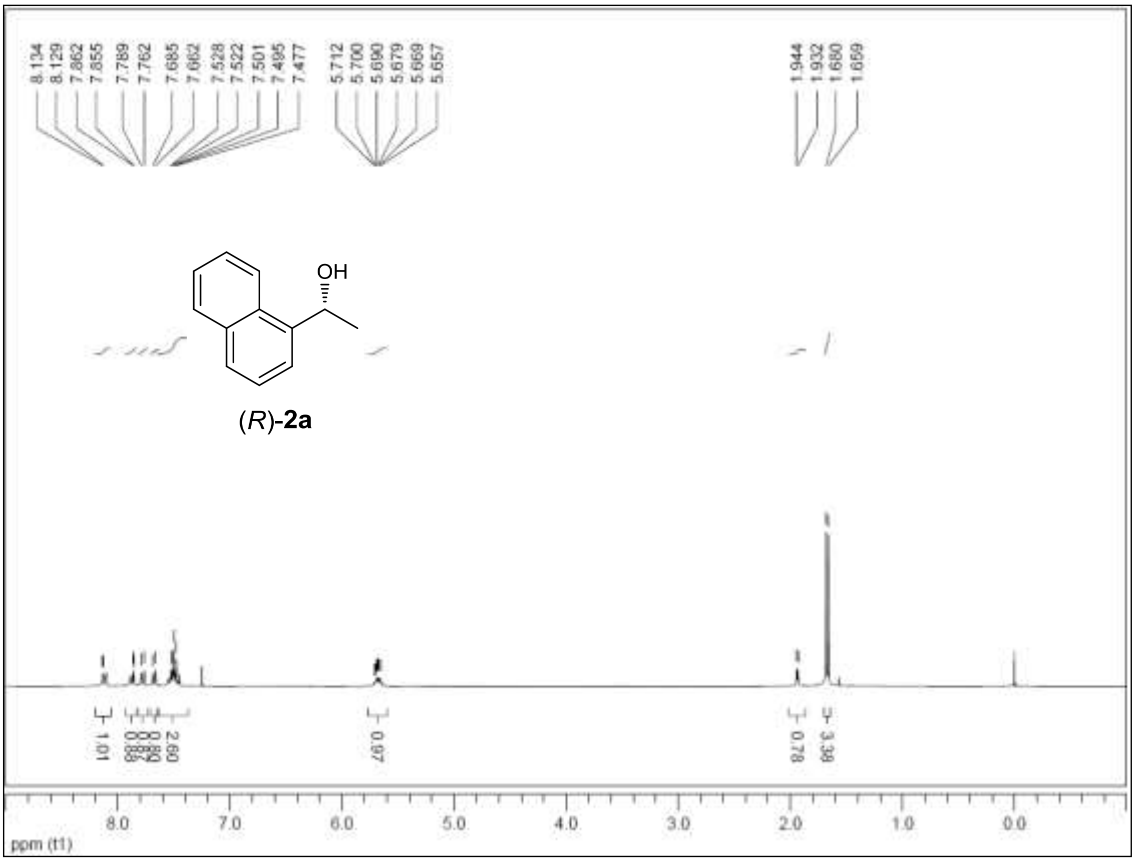

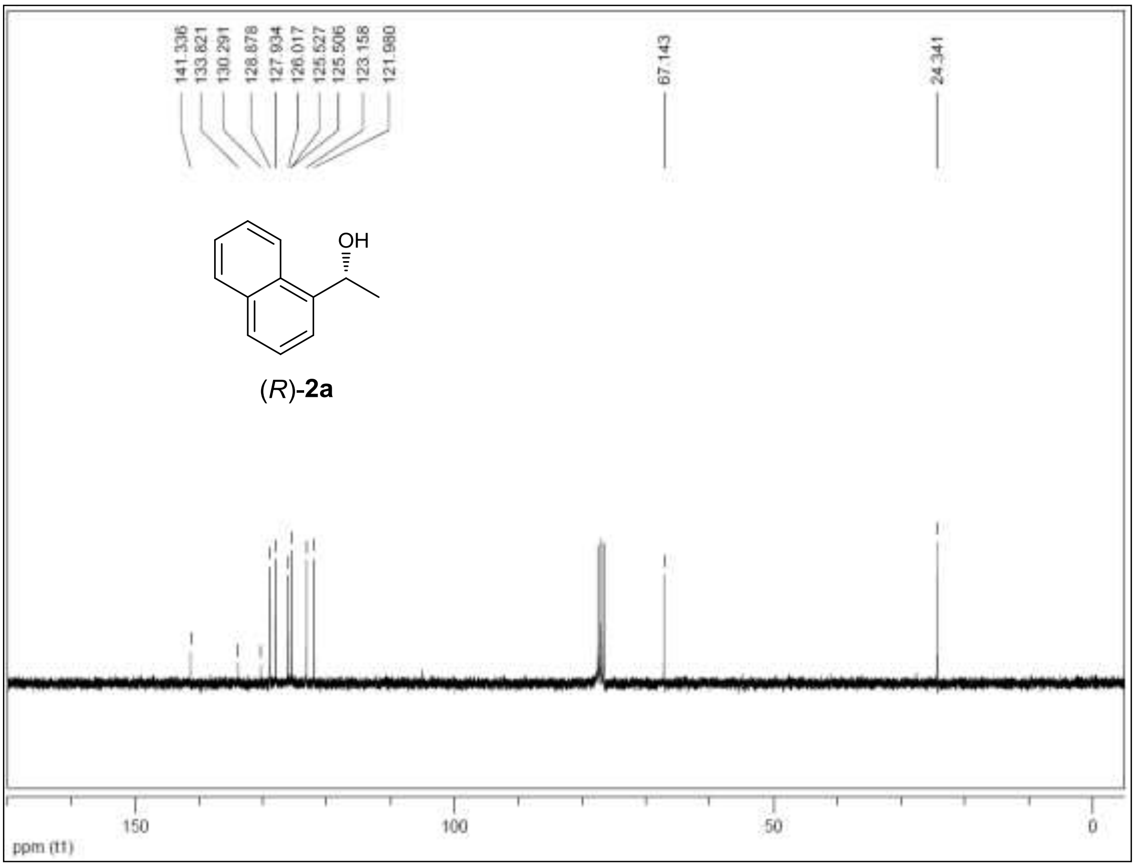

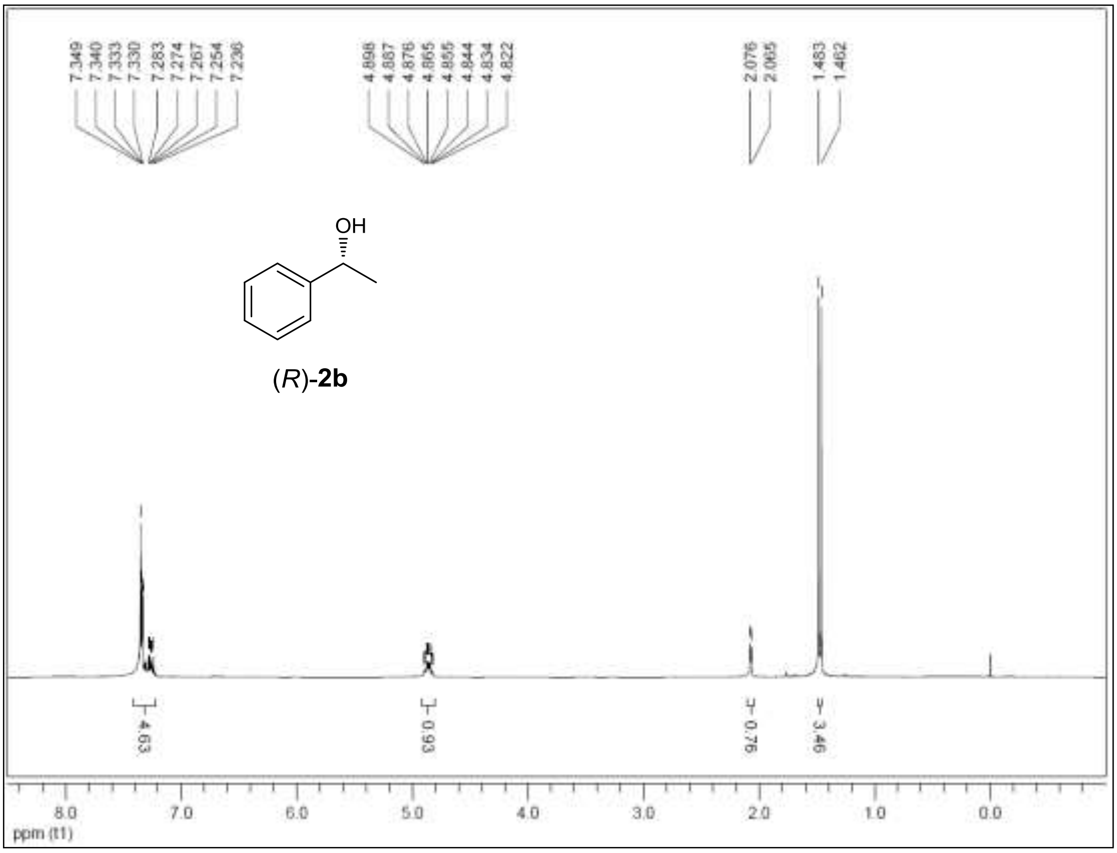
7.349
7.340
7.333
7.330
7.283
7.274
7.267
7.254
7.236
4.898
4.887
4.876
4.865
4.855
4.844
4.834
4.822
2.076
2.065
1.483
1.462
OH
(*R*)-**2b**
4.63
0.93
0.76
3.46
8.0
7.0
6.0
5.0
4.0
3.0
2.0
1.0
0.0
ppm (t1)

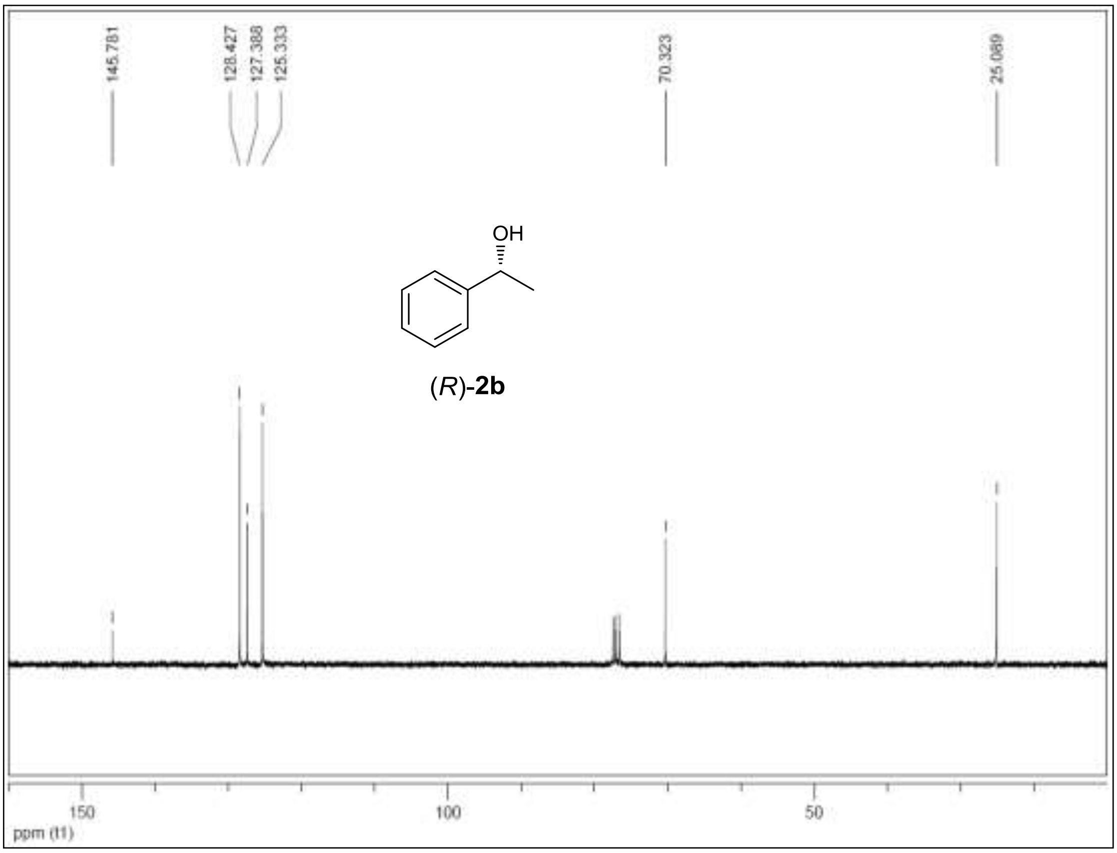
145.781
128.427
127.388
125.333
70.323
25.089
OH
(*R*)-**2b**
150
100
50
ppm (t1)

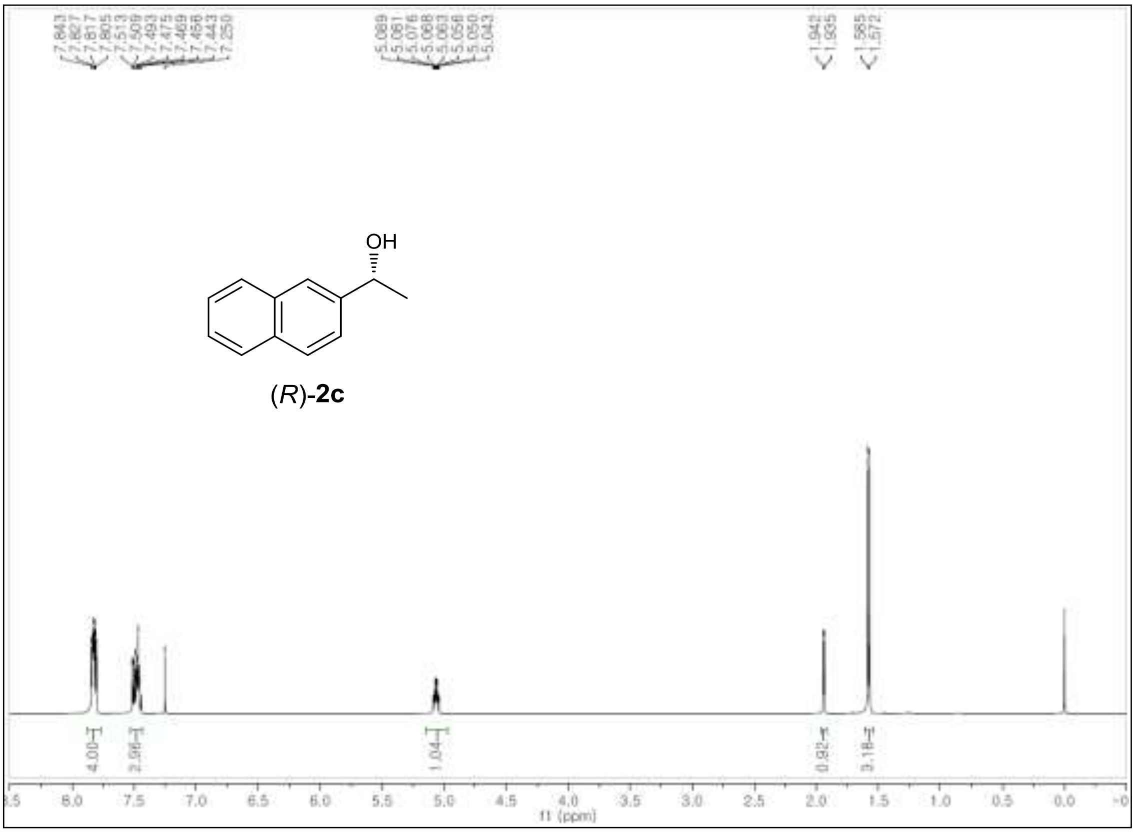
7.843
7.827
7.817
7.805
7.513
7.509
7.493
7.475
7.469
7.458
7.443
7.250
5.089
5.081
5.076
5.068
5.063
5.058
5.050
5.043
1.942
1.935
1.585
1.572
OH
(R)-2c
4.00
2.96
1.04
0.92
3.18
f1 (ppm)

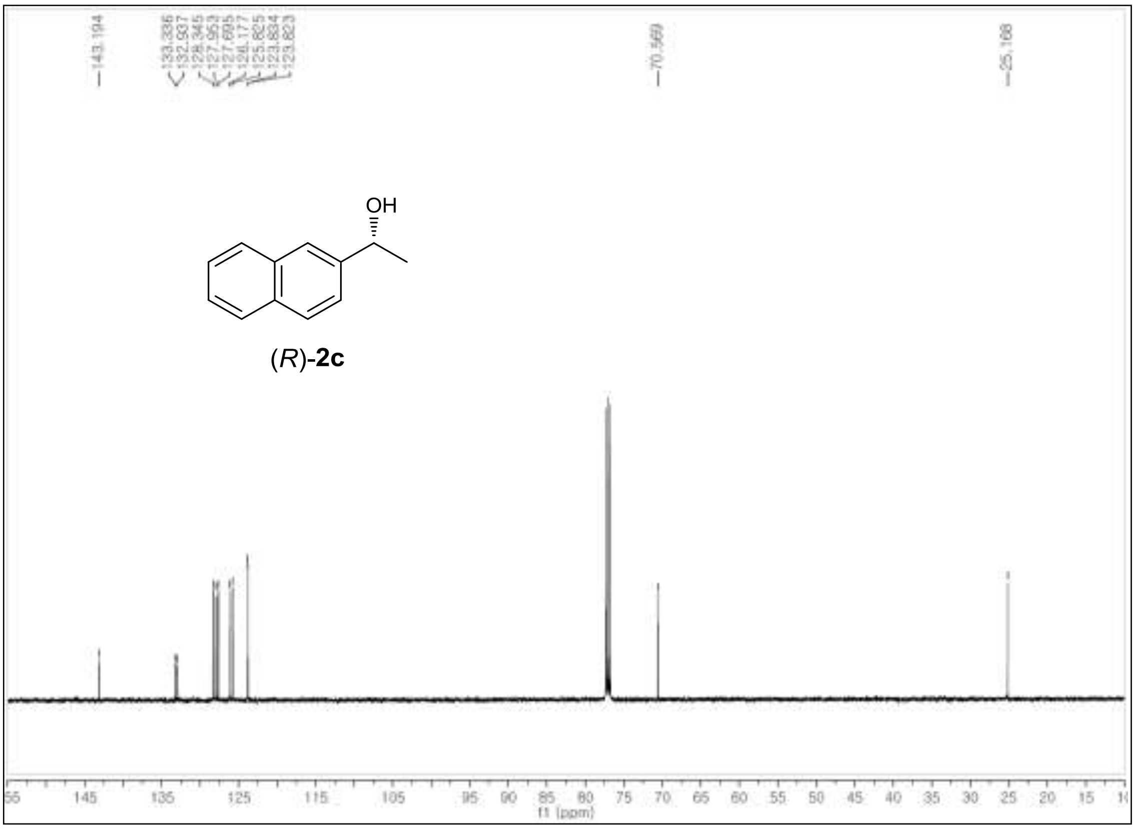
143.194
133.336
132.937
128.345
127.953
127.695
126.177
125.825
123.834
123.823
70.569
25.168
OH
(R)-2c
f1 (ppm)

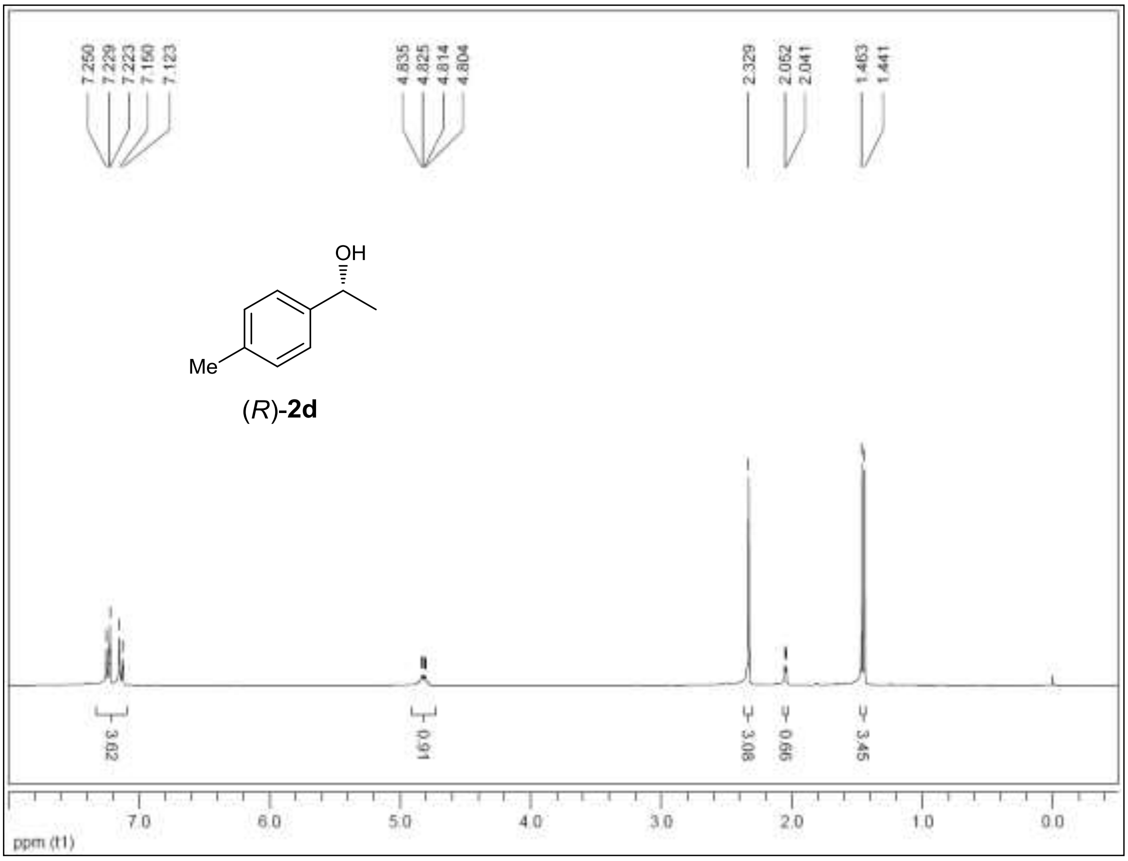
7.250
7.229
7.223
7.150
7.123
4.835
4.825
4.814
4.804
2.329
2.052
2.041
1.463
1.441
OH
Me
(*R*)-**2d**
3.02
0.91
3.08
0.66
3.45
7.0
6.0
5.0
4.0
3.0
2.0
1.0
0.0
ppm (t1)

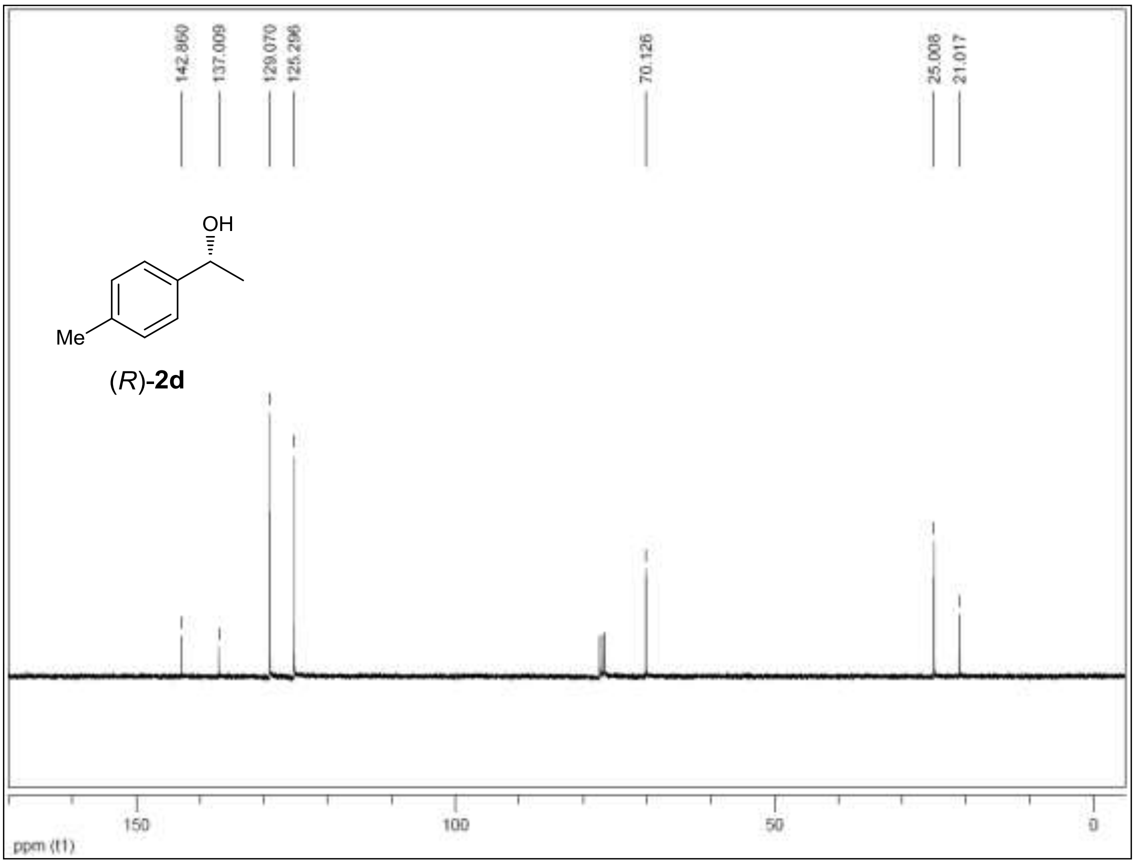
142.860
137.009
129.070
125.296
70.126
25.008
21.017
OH
Me
(*R*)-**2d**
150
100
50
0
ppm (t1)

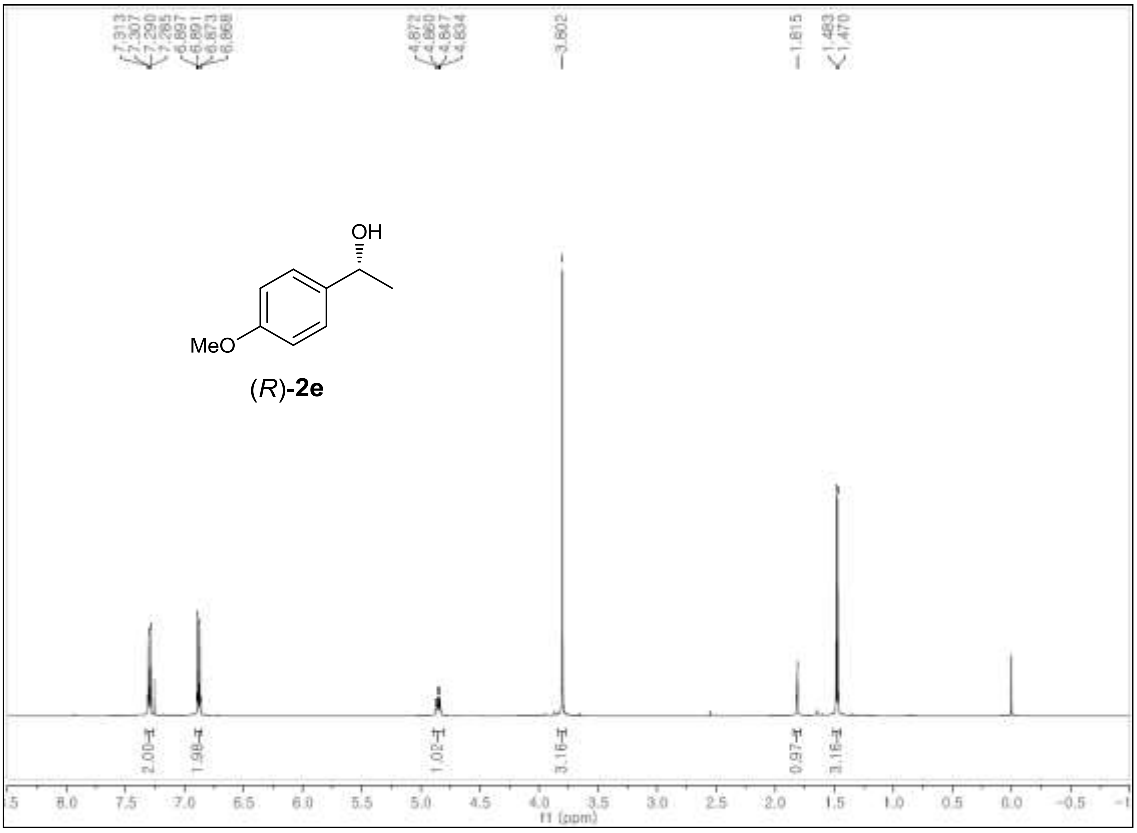
7.313
7.307
7.290
7.285
6.897
6.891
6.873
6.868
4.872
4.860
4.847
4.834
3.802
1.815
1.483
1.470
OH
MeO
(R)-2e
2.00
1.98
1.02
3.16
0.97
3.16
f1 (ppm)

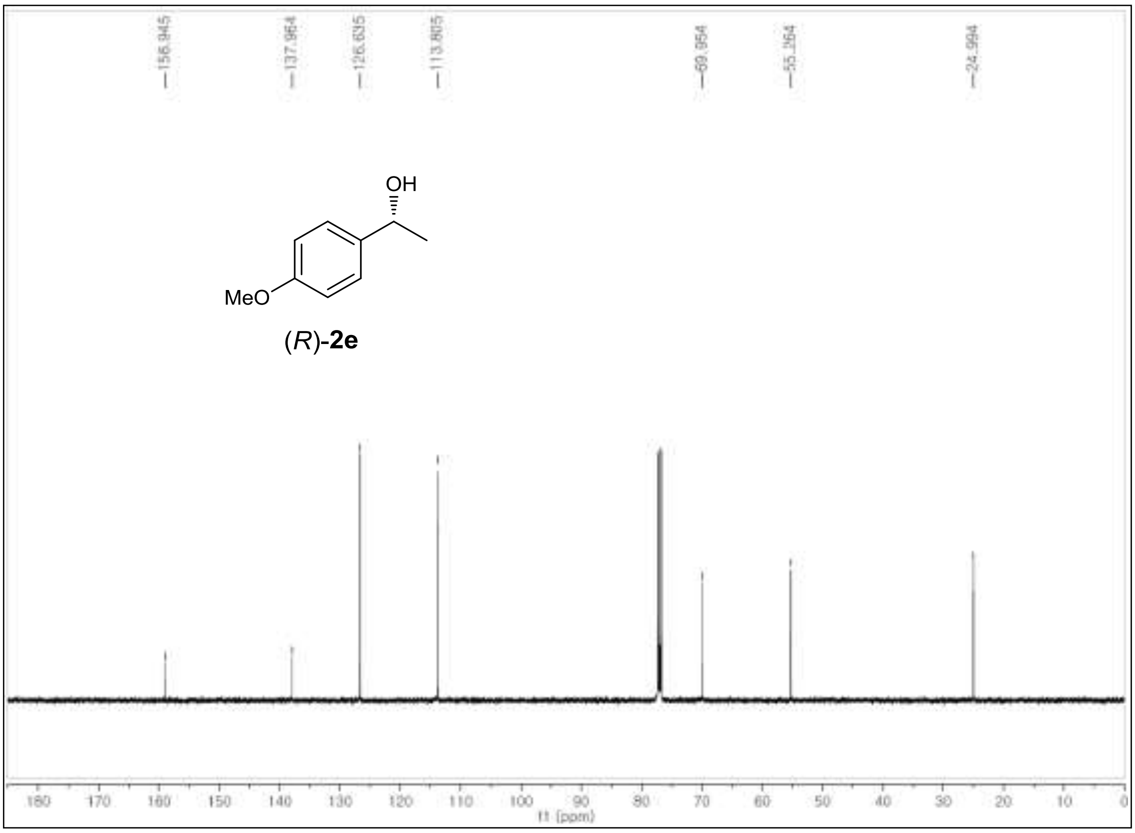
158.945
137.964
126.635
113.805
69.954
55.264
24.994
OH
MeO
(R)-2e
f1 (ppm)

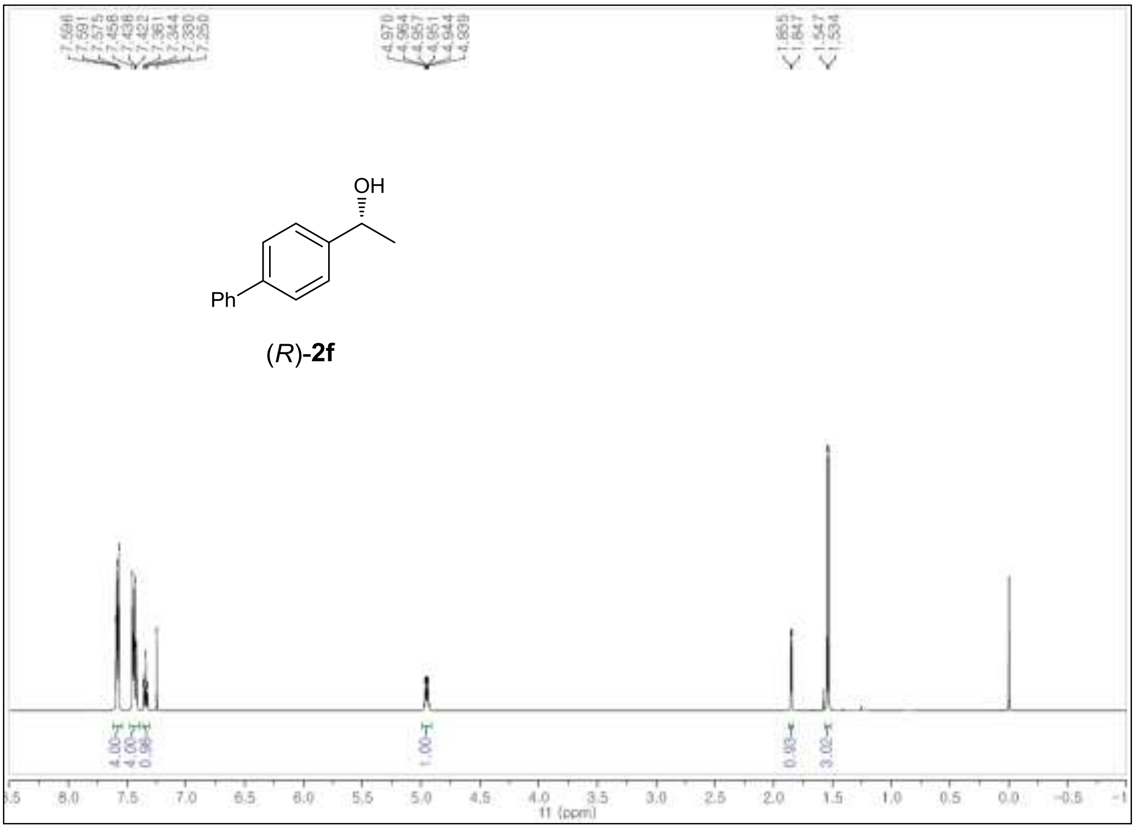

7.596
7.591
7.575
7.458
7.438
7.422
7.361
7.344
7.330
7.250
4.970
4.964
4.957
4.951
4.944
4.939
1.865
1.847
1.547
1.534
OH
Ph
(*R*)-**2f**
4.00
4.00
0.98
1.00
0.93
3.02
f1 (ppm)

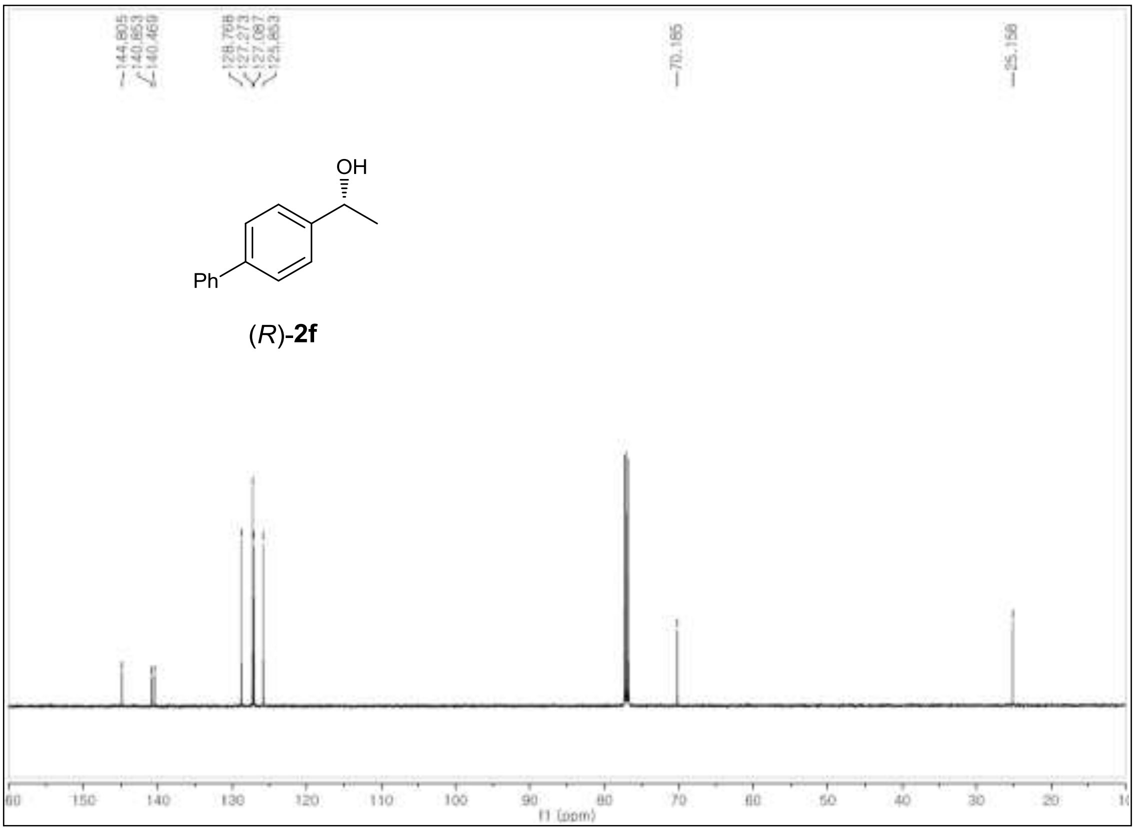

144.805
140.853
140.469
128.768
127.273
127.087
125.853
70.165
25.156
OH
Ph
(*R*)-**2f**
f1 (ppm)

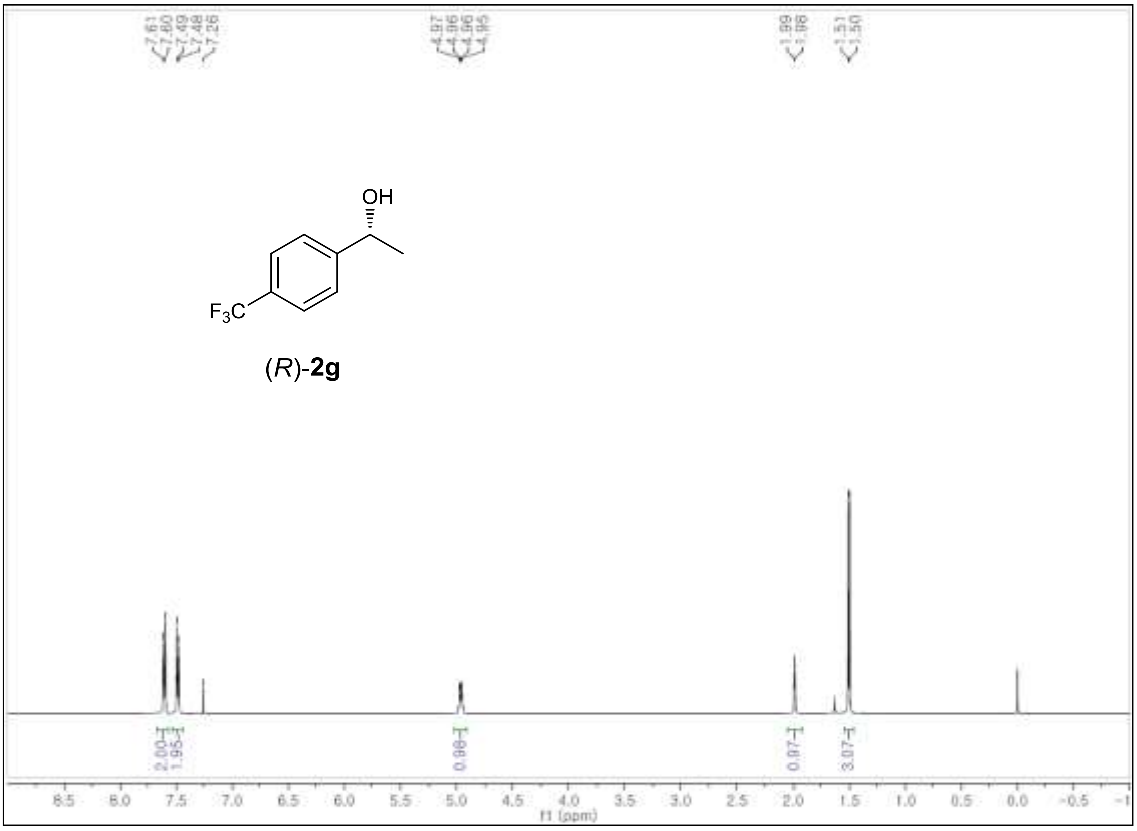
7.61
7.60
7.49
7.48
7.26
4.97
4.96
4.96
4.95
1.99
1.98
1.51
1.50
OH
F3C
(R)-2g
2.00
1.95
0.98
0.97
3.07
8.5
8.0
7.5
7.0
6.5
6.0
5.5
5.0
4.5
4.0
3.5
3.0
2.5
2.0
1.5
1.0
0.5
0.0
-0.5
f1 (ppm)

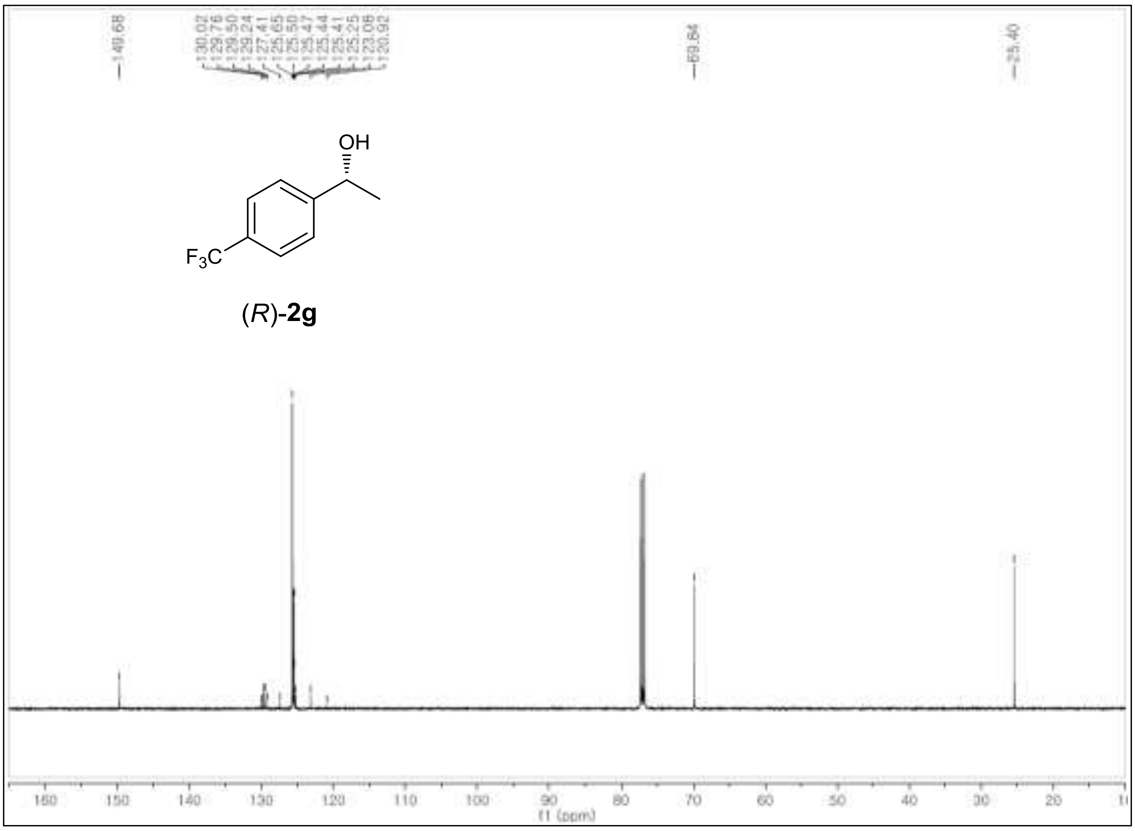
149.68
130.02
129.76
129.50
129.24
127.41
125.65
125.50
125.47
125.44
125.41
125.25
123.08
120.92
69.84
25.40
OH
F3C
(R)-2g
160
150
140
130
120
110
100
90
80
70
60
50
40
30
20
f1 (ppm)

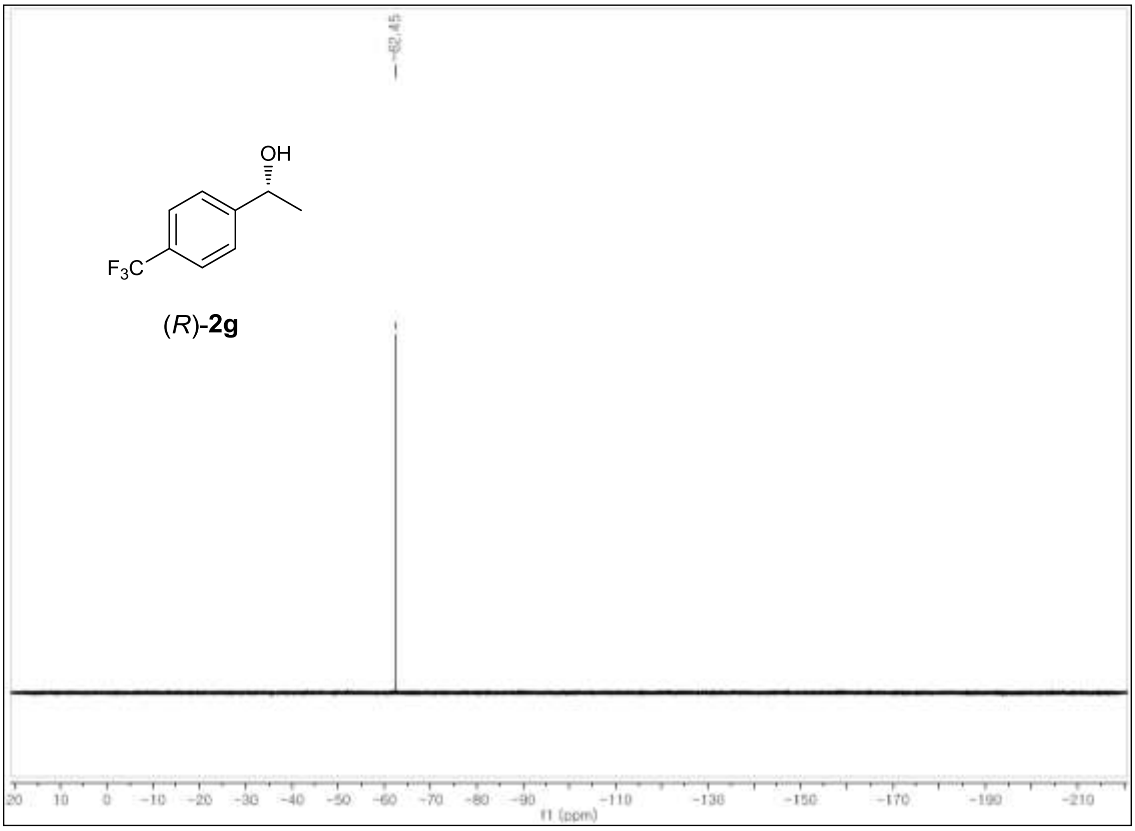
-62.45
OH
F3C
(R)-2g
20
10
0
-10
-20
-30
-40
-50
-60
-70
-80
-90
-110
-130
-150
-170
-190
-210
f1 (ppm)

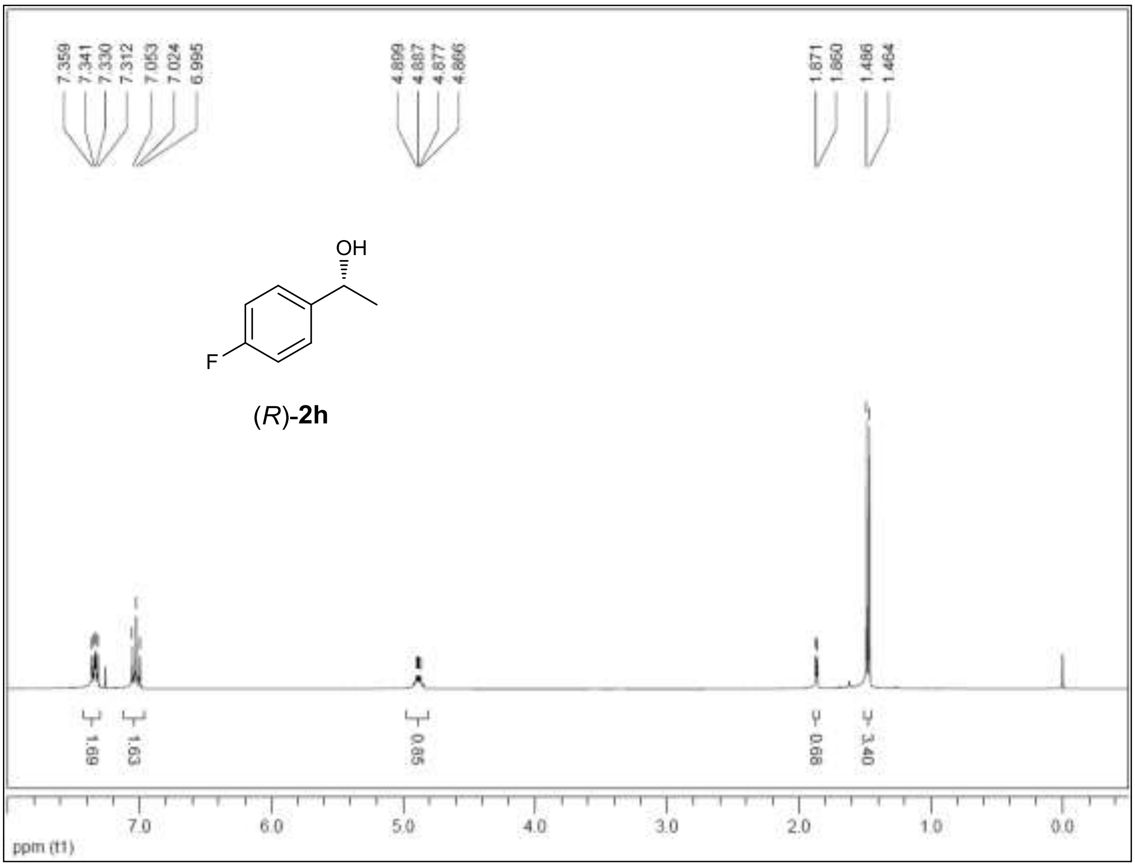
7.359
7.341
7.330
7.312
7.053
7.024
6.995
4.899
4.887
4.877
4.866
1.871
1.860
1.486
1.464
OH
F
(R)-2h
1.69
1.63
0.85
0.68
3.40
7.0
6.0
5.0
4.0
3.0
2.0
1.0
0.0
ppm (t1)

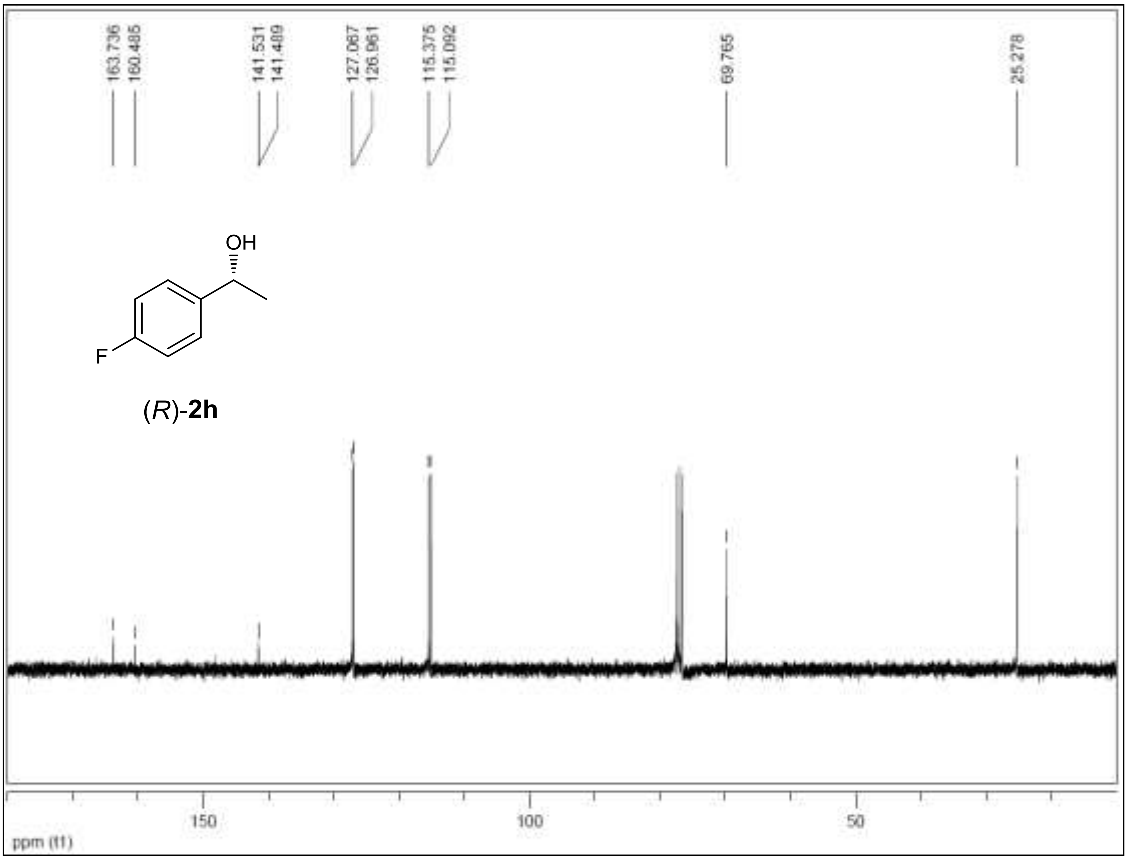
163.736
160.485
141.531
141.489
127.067
126.961
115.375
115.092
69.765
25.278
OH
F
(R)-2h
150
100
50
ppm (t1)

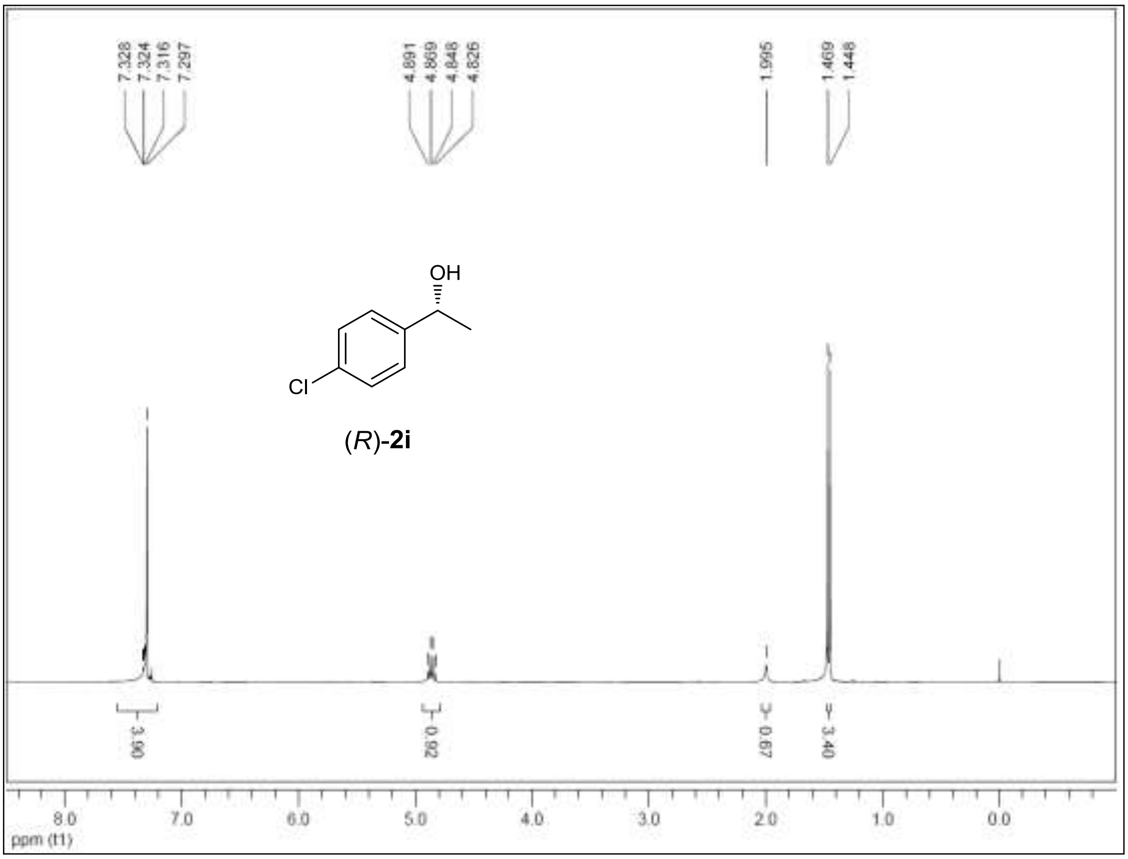
7.328
7.324
7.316
7.297
4.891
4.869
4.848
4.826
1.995
1.469
1.448
OH
Cl
(R)-2i
3.90
0.92
0.67
3.40
8.0
7.0
6.0
5.0
4.0
3.0
2.0
1.0
0.0
ppm (t1)

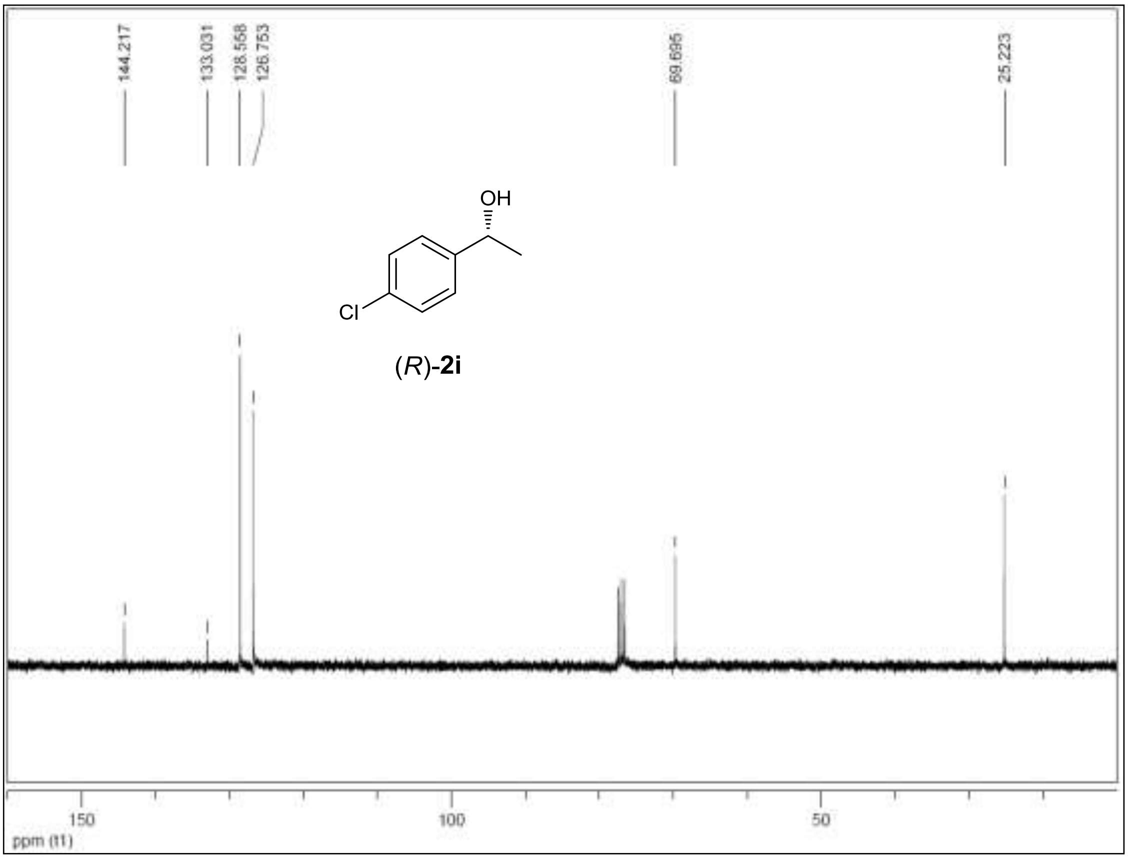
144.217
133.031
128.558
126.753
69.695
25.223
OH
Cl
(R)-2i
150
100
50
ppm (t1)

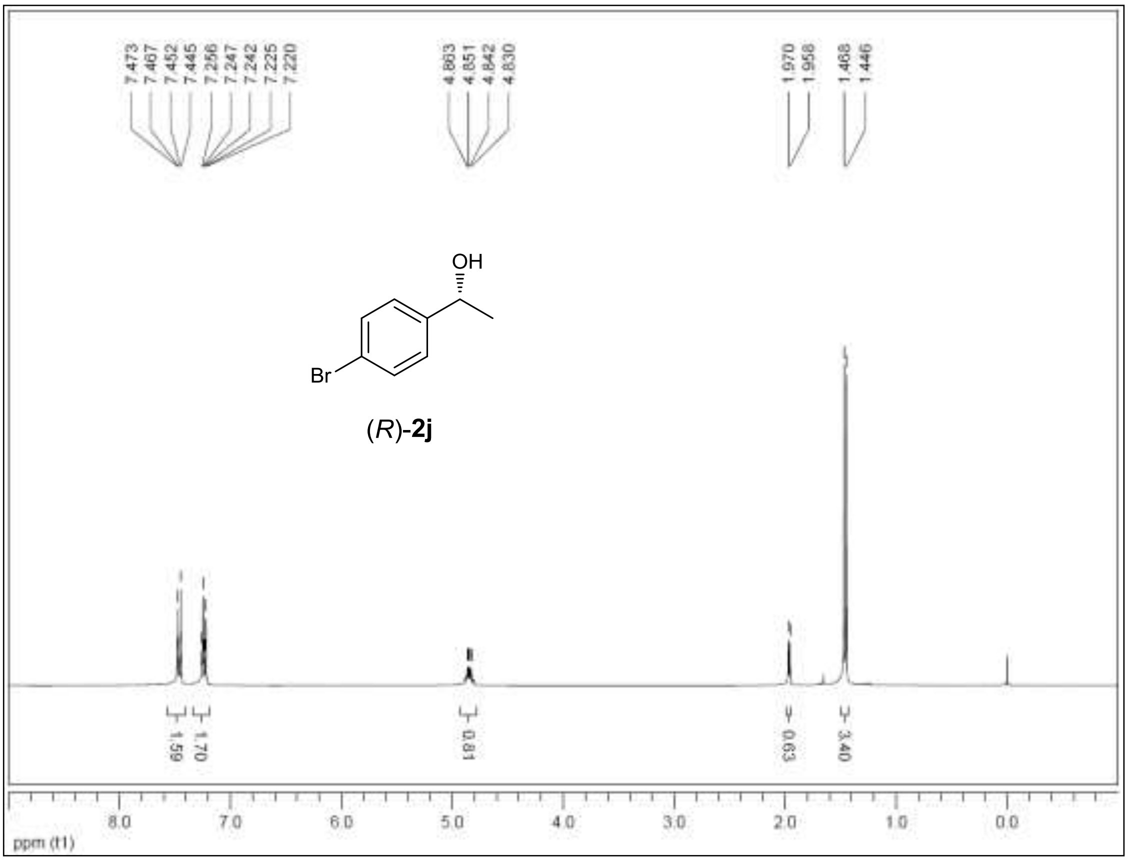
7.473
7.467
7.452
7.445
7.256
7.247
7.242
7.225
7.220
4.863
4.851
4.842
4.830
1.970
1.958
1.468
1.446
OH
Br
(R)-2j
1.59
1.70
0.81
0.63
3.40
8.0
7.0
6.0
5.0
4.0
3.0
2.0
1.0
0.0
ppm (t1)

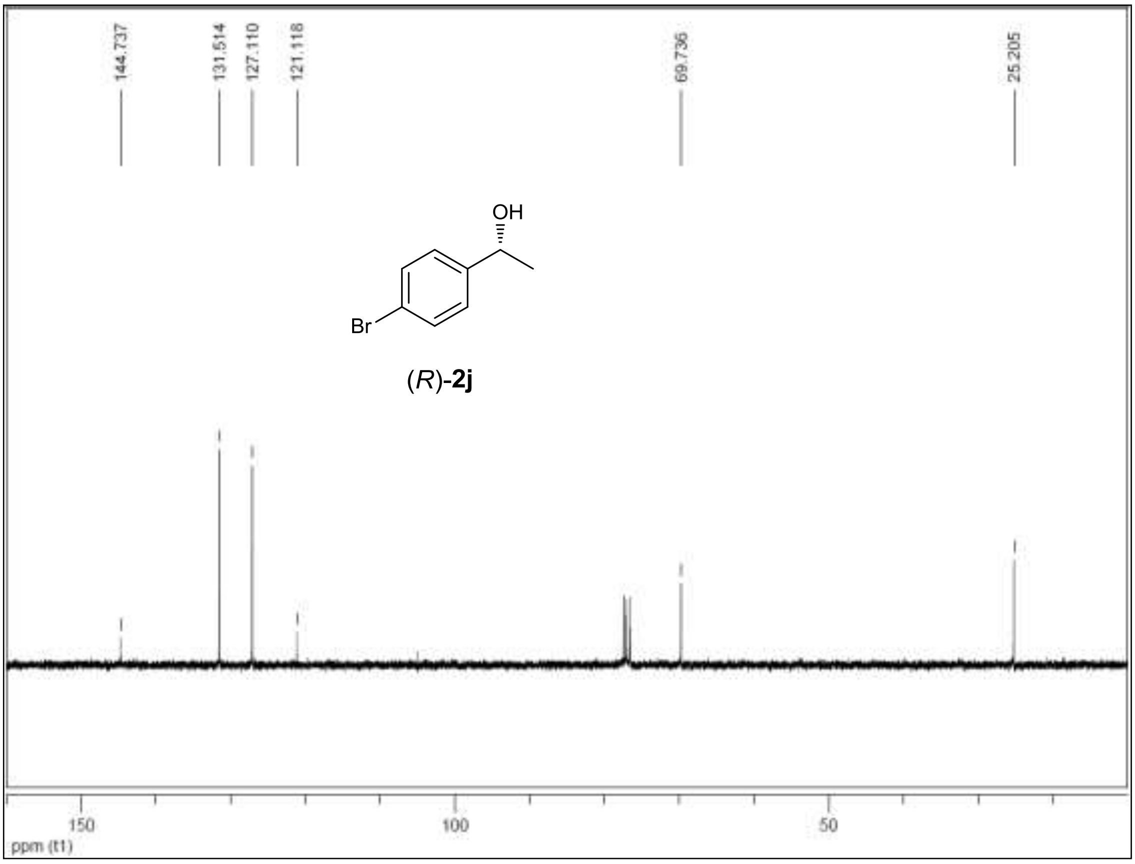

144.737
131.514
127.110
121.118
69.736
25.205
OH
Br
(R)-2j
150
100
50
ppm (t1)

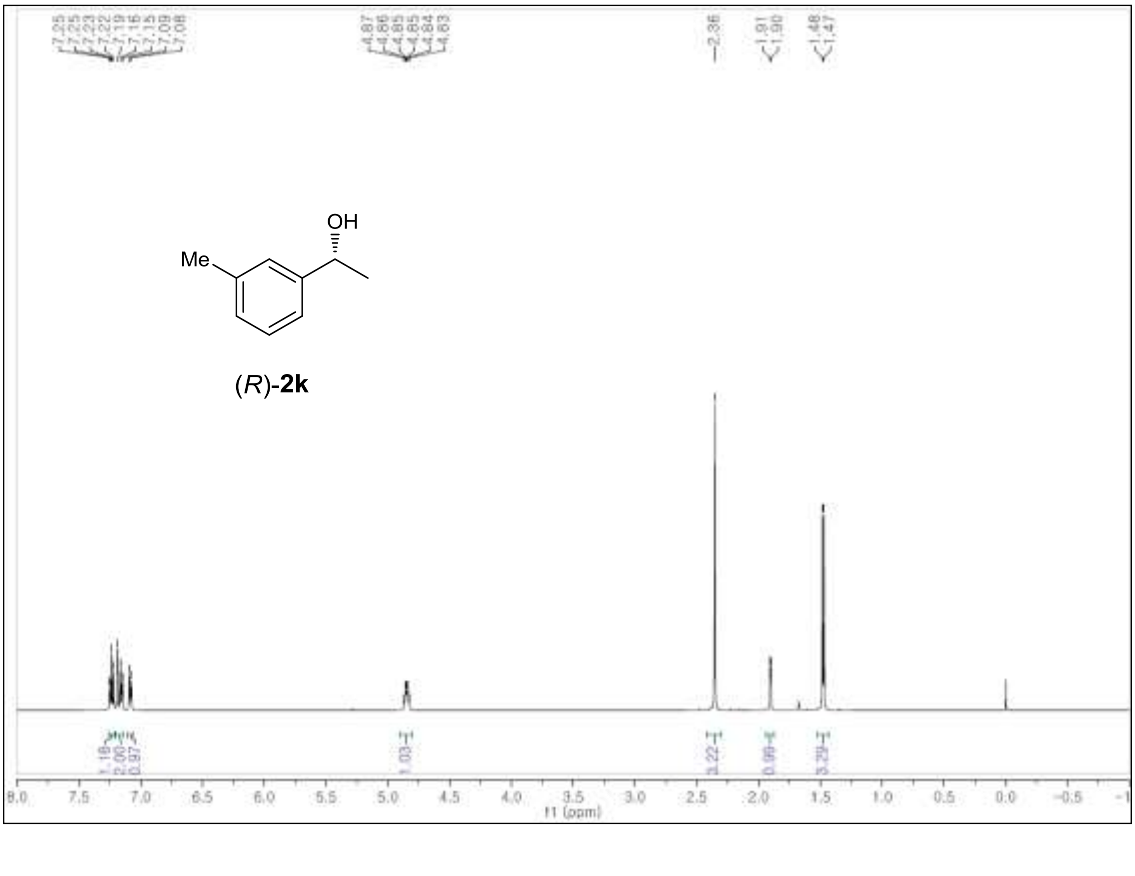

2.36
OH
Me
(R)-2k
1.03
3.22
8.0
7.5
7.0
6.5
6.0
5.5
5.0
4.5
4.0
3.5
3.0
2.5
2.0
1.5
1.0
0.5
0.0
-0.5
f1 (ppm)

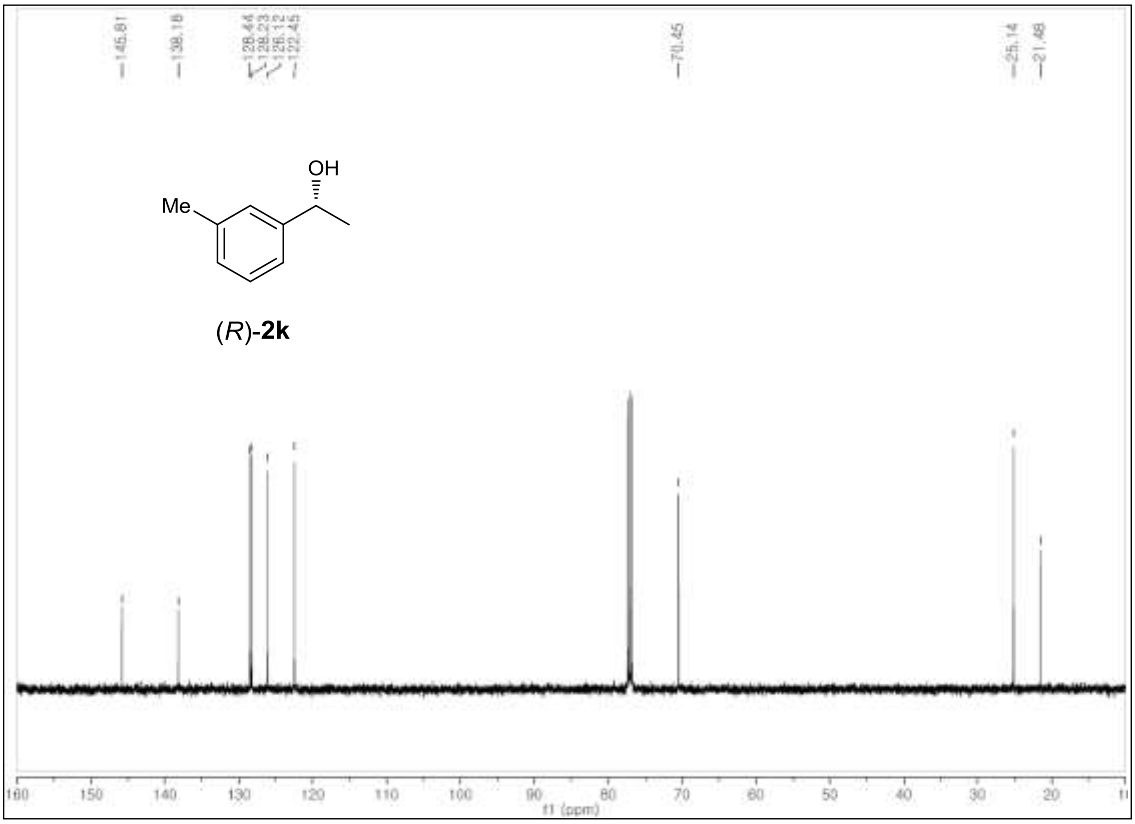

145.81
138.16
128.44
128.23
126.12
122.45
70.45
25.14
21.48
OH
Me
(*R*)-**2k**
f1 (ppm)

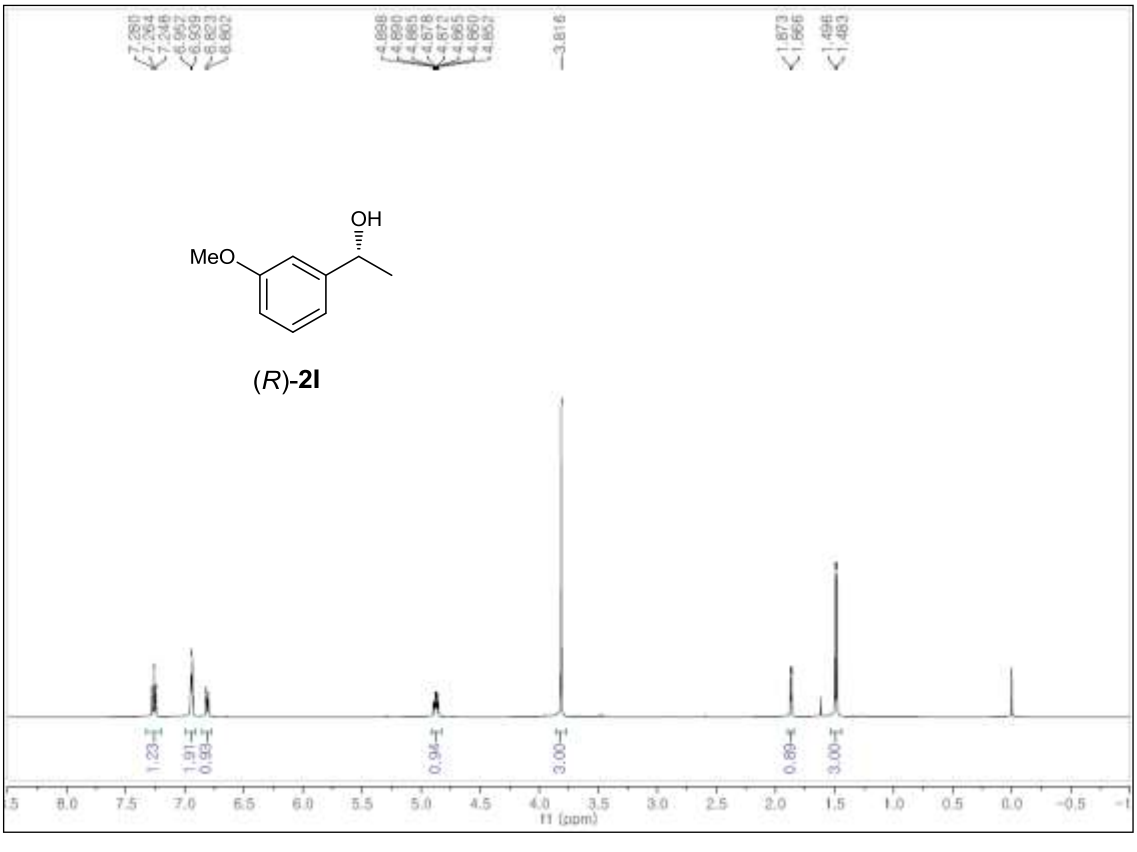

7.280
7.264
7.248
6.957
6.939
6.823
6.802
4.898
4.890
4.885
4.878
4.872
4.865
4.860
4.852
3.816
1.873
1.866
1.496
1.483
OH
MeO
(*R*)-**2l**
1.23
1.91
0.93
0.94
3.00
0.89
3.00
f1 (ppm)

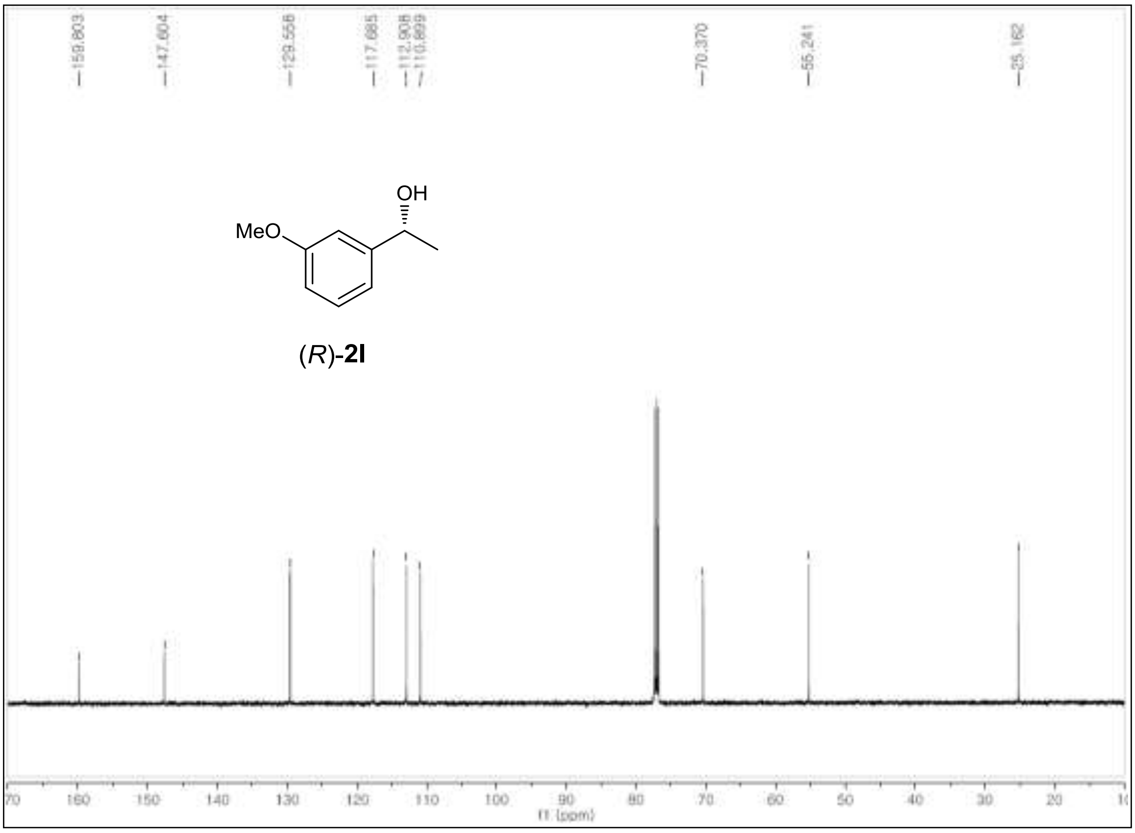
159.803
147.604
129.556
117.685
112.908
110.899
70.370
55.241
25.162
OH
MeO
(R)-2l
f1 (ppm)

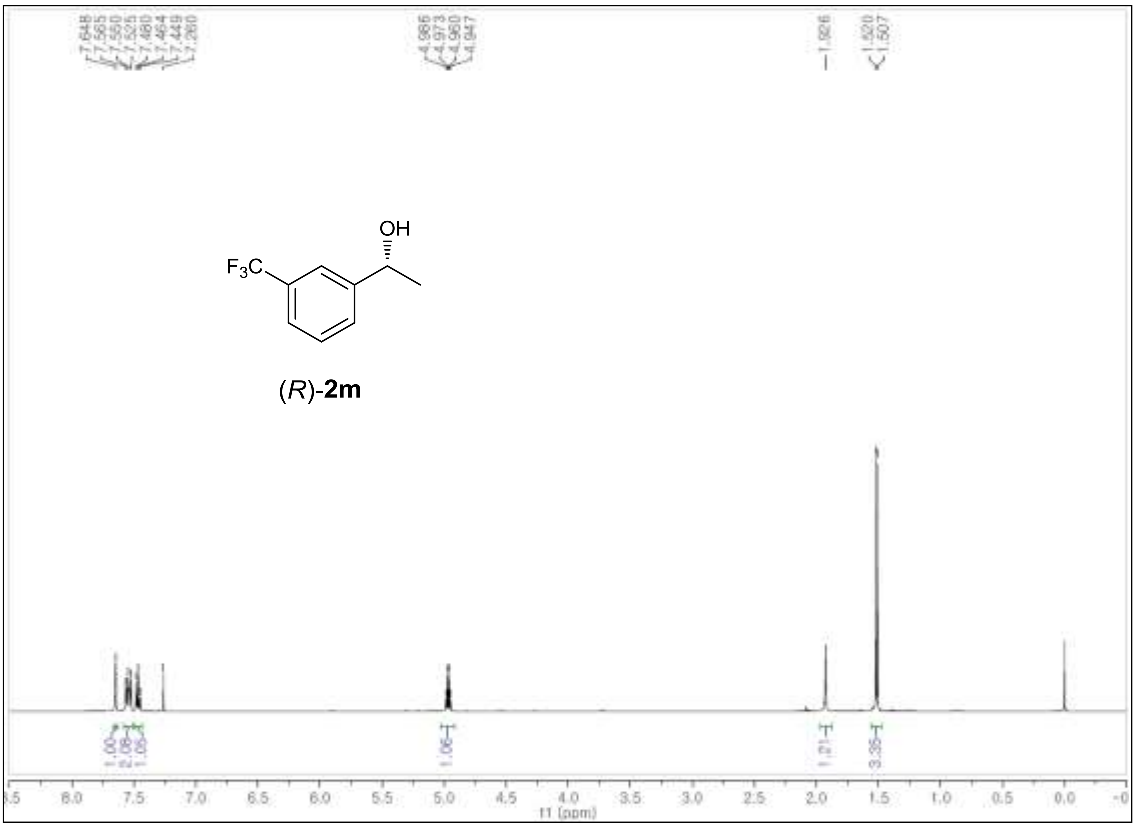
7.648
7.565
7.550
7.525
7.480
7.464
7.449
7.260
4.986
4.973
4.960
4.947
1.926
1.520
1.507
OH
F3C
(R)-2m
1.00
2.08
1.05
1.06
1.01
3.35
f1 (ppm)

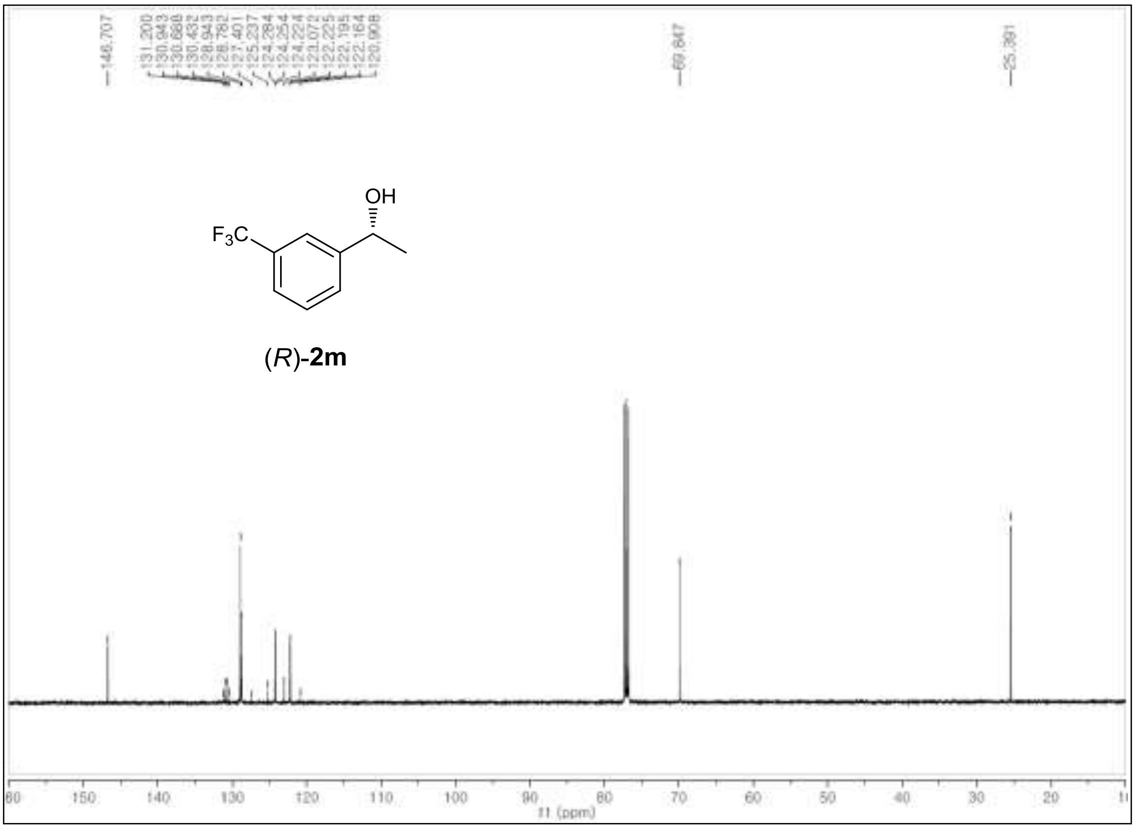
146.707
131.200
130.943
130.688
130.432
128.943
128.782
127.401
125.237
124.284
124.254
124.224
123.072
122.225
122.195
122.164
120.908
69.847
25.391
OH
F_3C
(*R*)-**2m**
f1 (ppm)

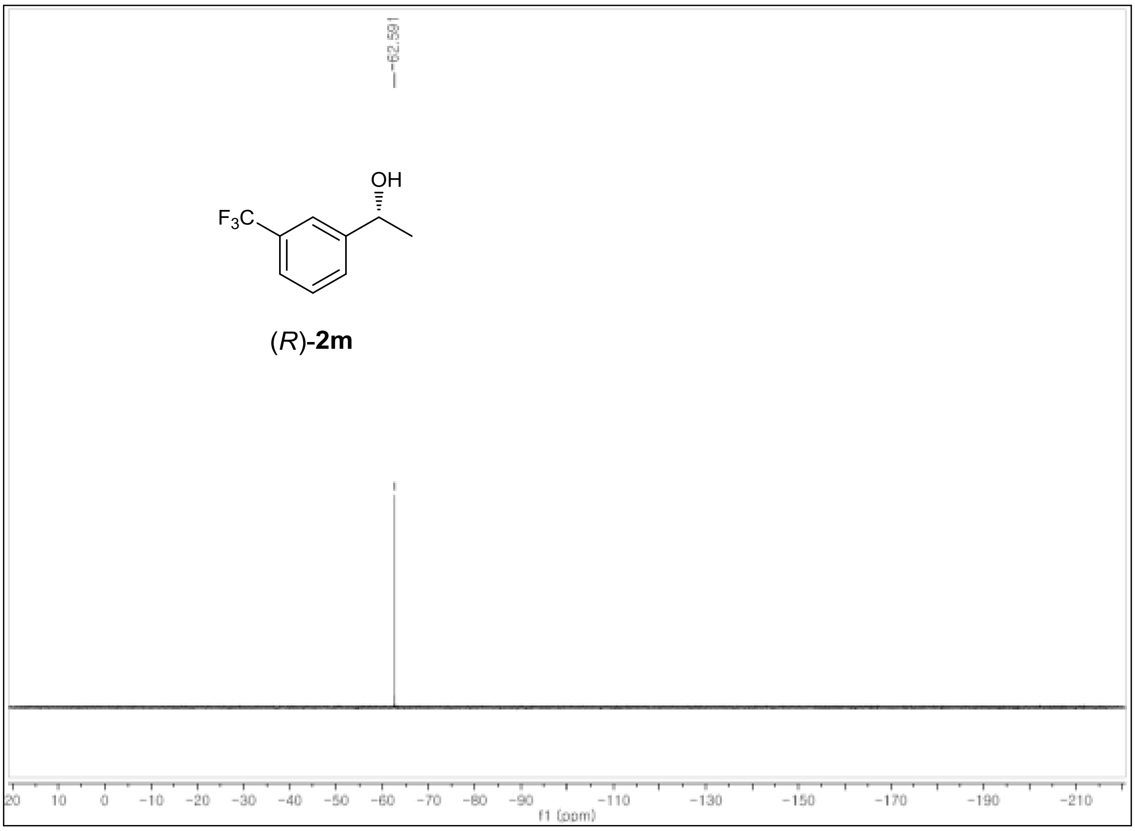
-62.591
OH
F_3C
(*R*)-**2m**
f1 (ppm)

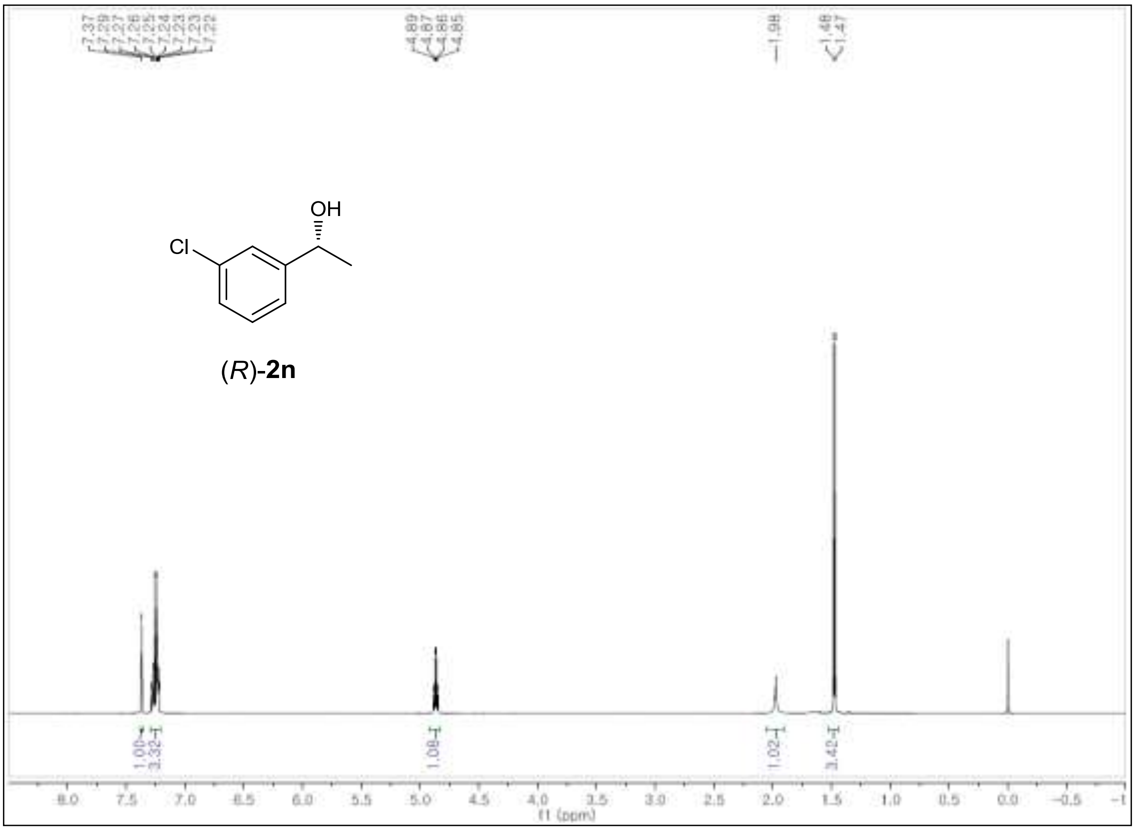
7.37
7.29
7.27
7.26
7.25
7.24
7.23
7.23
7.22
4.89
4.87
4.86
4.85
1.98
1.48
1.47
OH
Cl
(*R*)-**2n**
1.00
3.32
1.08
1.02
3.42
8.0
7.5
7.0
6.5
6.0
5.5
5.0
4.5
4.0
3.5
3.0
2.5
2.0
1.5
1.0
0.5
0.0
-0.5
f1 (ppm)

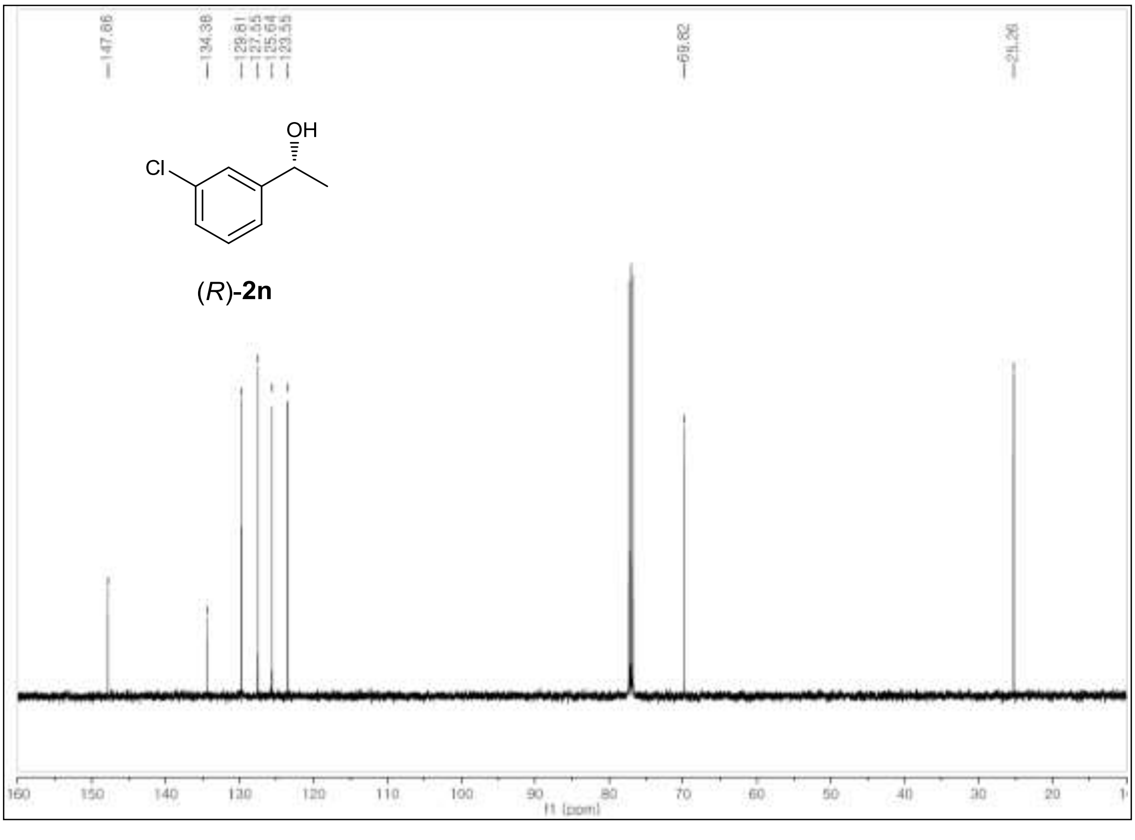
147.86
134.38
129.81
127.55
125.64
123.55
69.82
25.26
OH
Cl
(*R*)-**2n**
160
150
140
130
120
110
100
90
80
70
60
50
40
30
20
f1 (ppm)

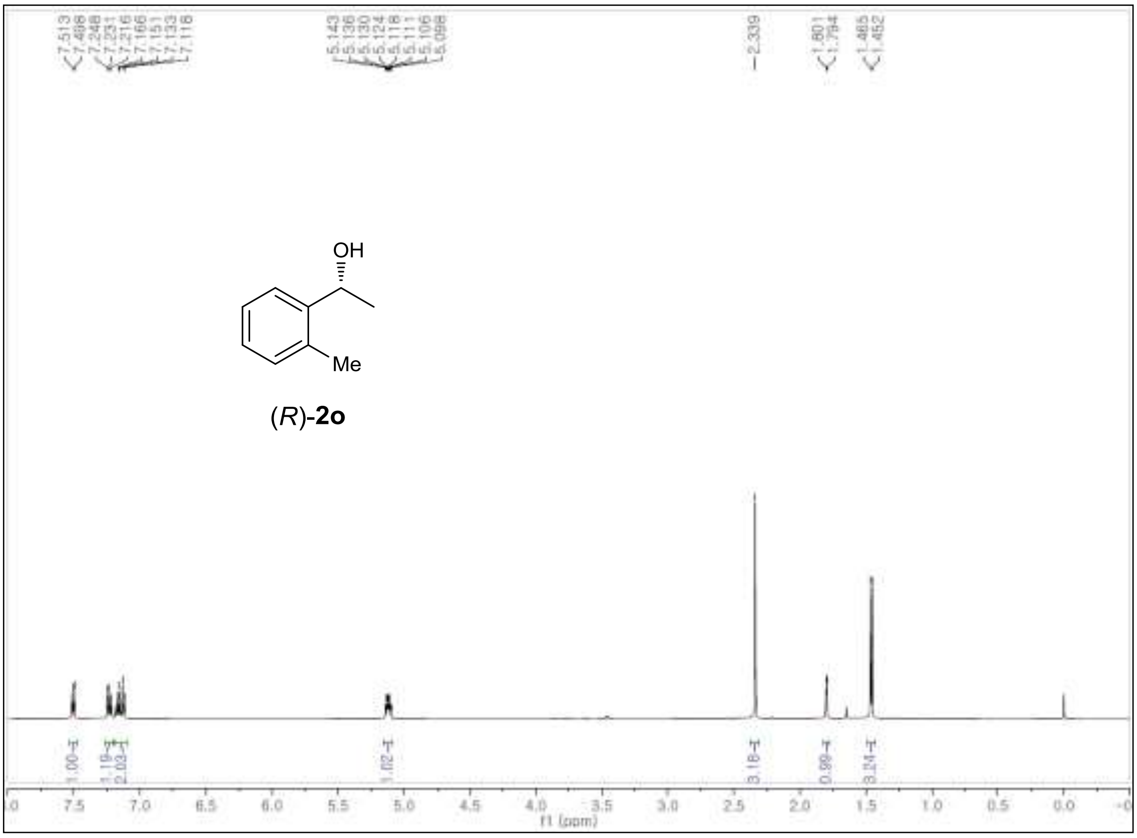
7.513
7.498
7.248
7.231
7.216
7.166
7.151
7.133
7.118
5.143
5.136
5.130
5.124
5.118
5.111
5.106
5.098
2.339
1.801
1.794
1.465
1.452
OH
Me
(R)-2o
1.00
1.19
2.03
1.02
3.16
0.99
3.24
f1 (ppm)

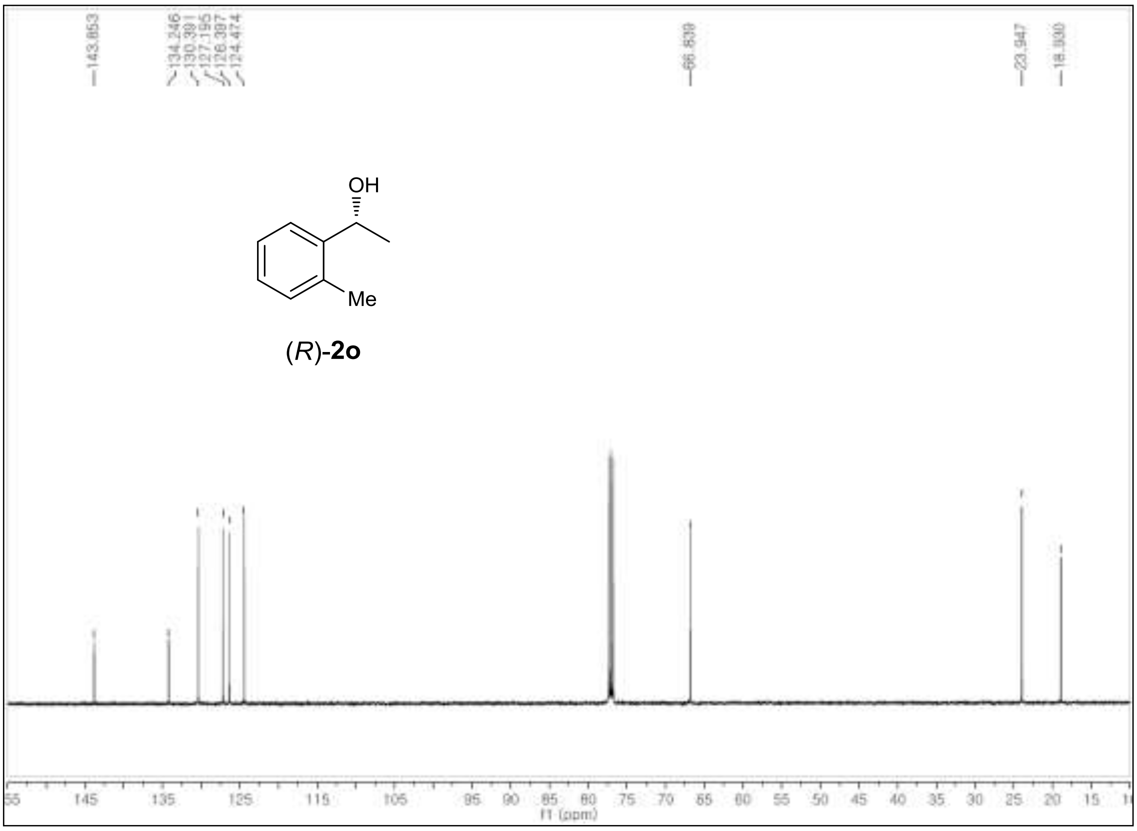
143.853
134.246
130.391
127.195
126.397
124.474
66.839
23.947
18.930
OH
Me
(R)-2o
f1 (ppm)

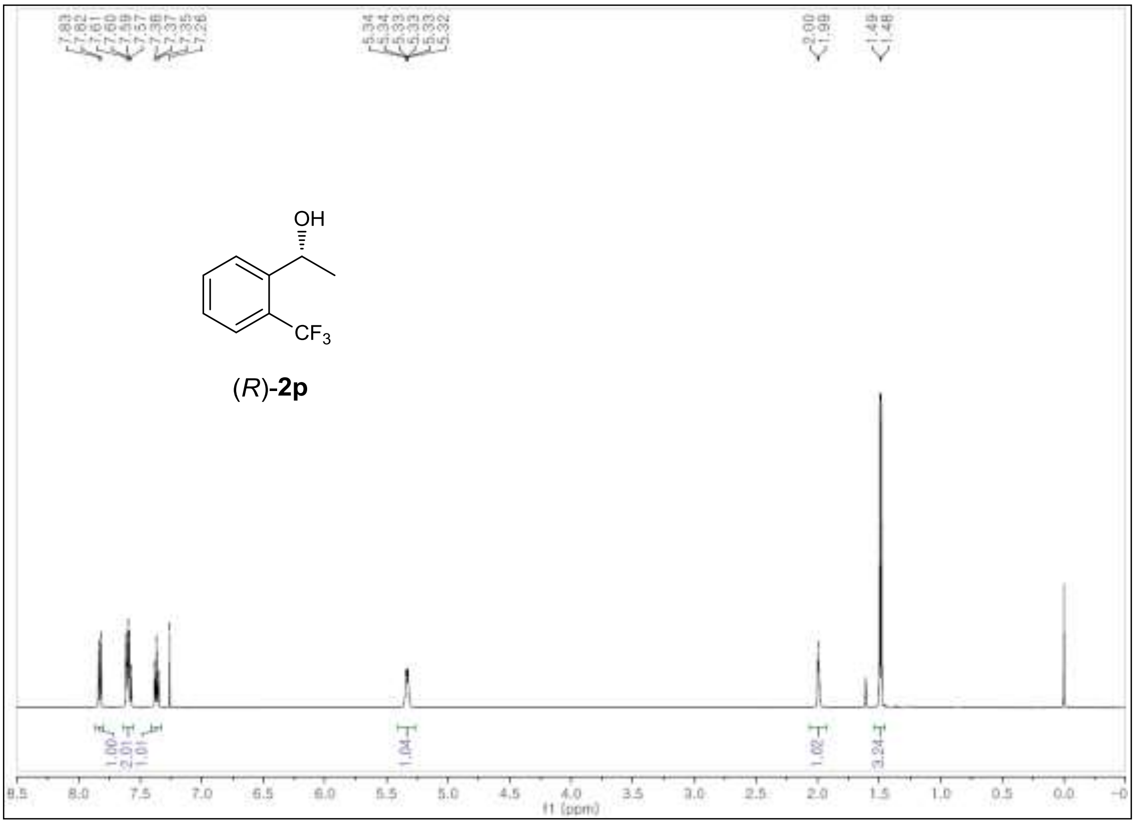
7.83
7.82
7.61
7.60
7.59
7.57
7.38
7.37
7.35
7.26
5.34
5.34
5.33
5.33
5.33
5.32
2.00
1.99
1.49
1.48
OH
CF3
(R)-2p
1.00
2.01
1.01
1.04
1.02
3.24
f1 (ppm)

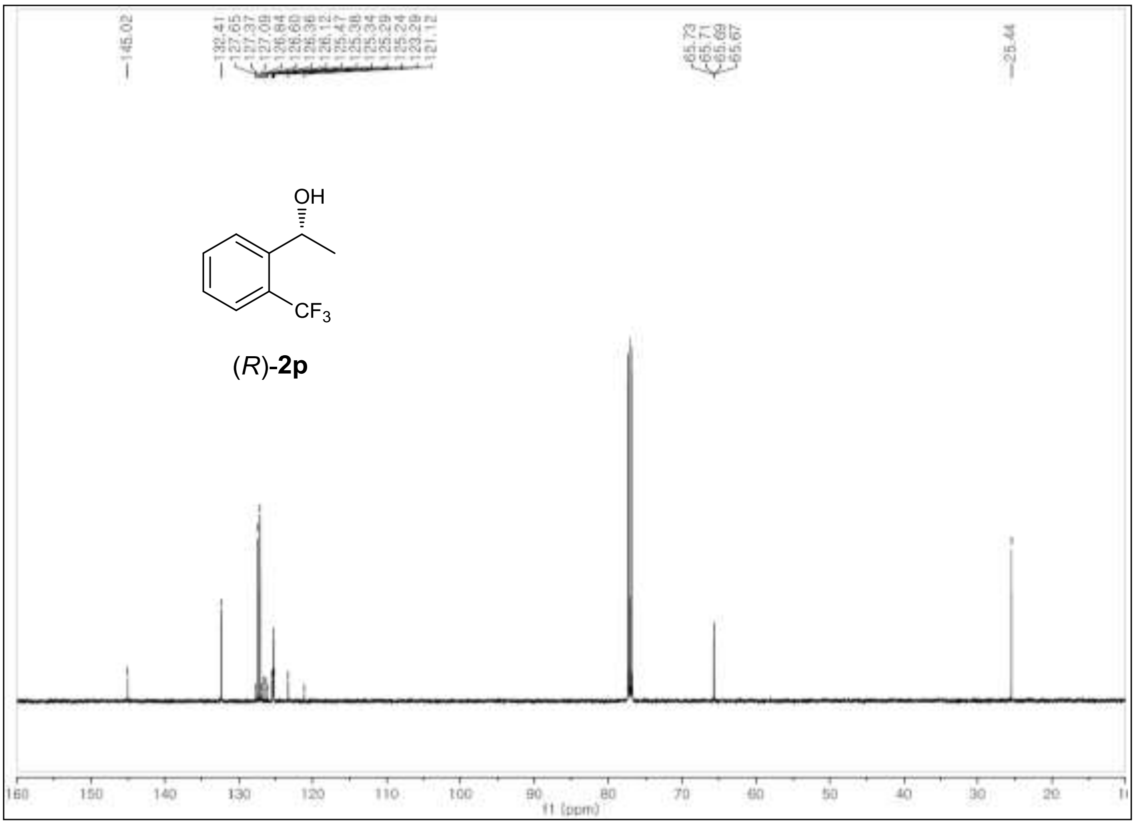
145.02
132.41
127.65
127.37
127.09
126.84
126.60
126.36
126.12
125.47
125.38
125.34
125.29
125.24
123.29
121.12
65.73
65.71
65.69
65.67
25.44
OH
CF3
(R)-2p
f1 (ppm)

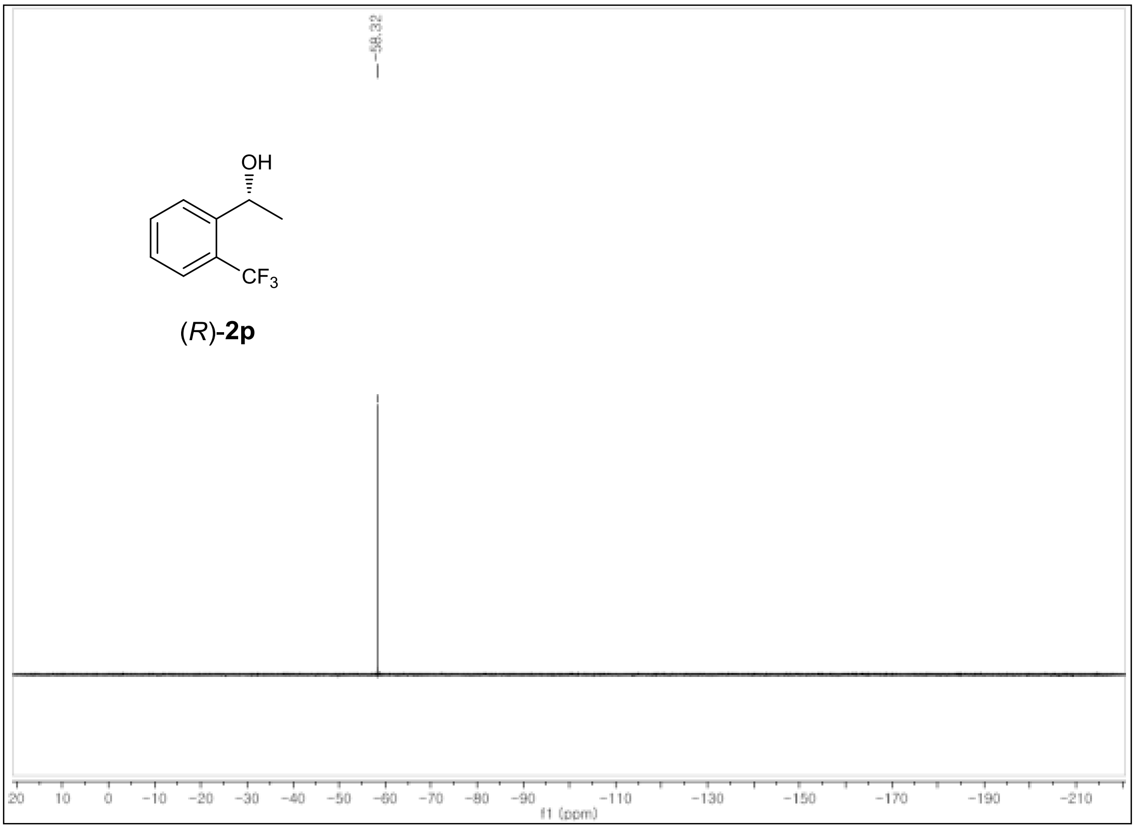
-58.32
OH
CF3
(R)-2p
f1 (ppm)

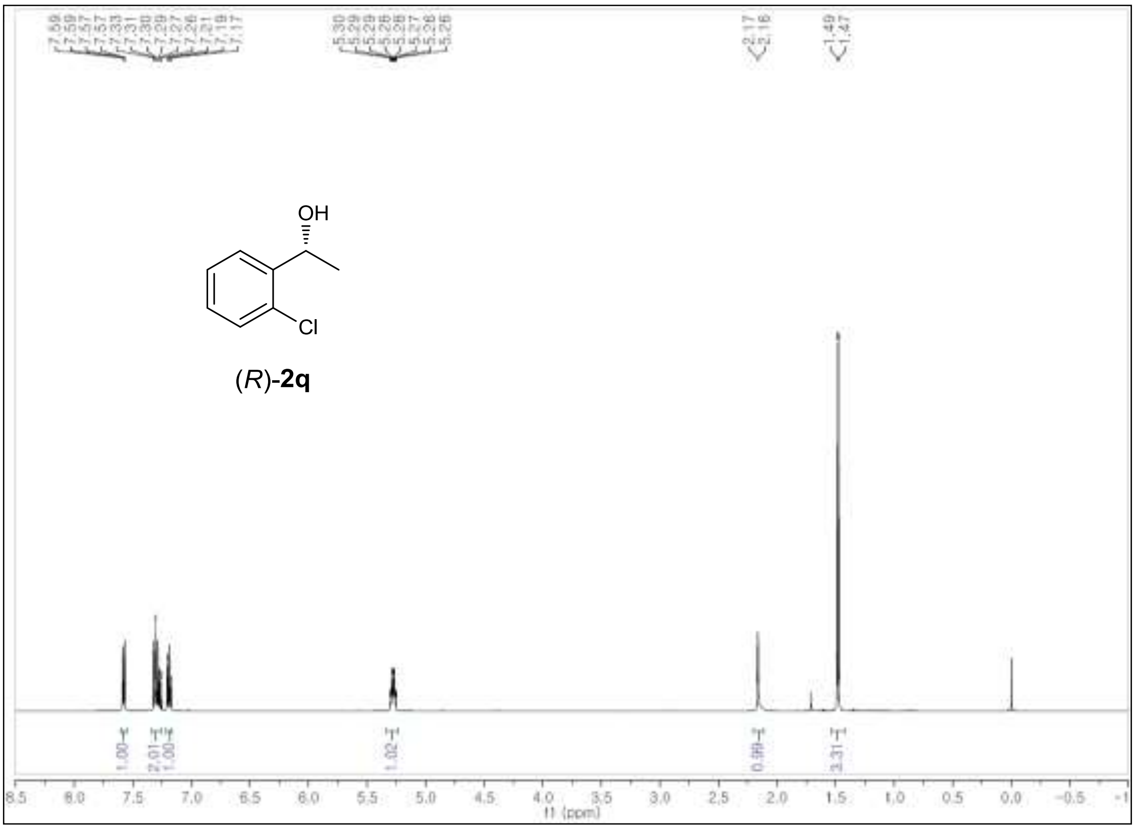
OH
Cl
(R)-2q
f1 (ppm)

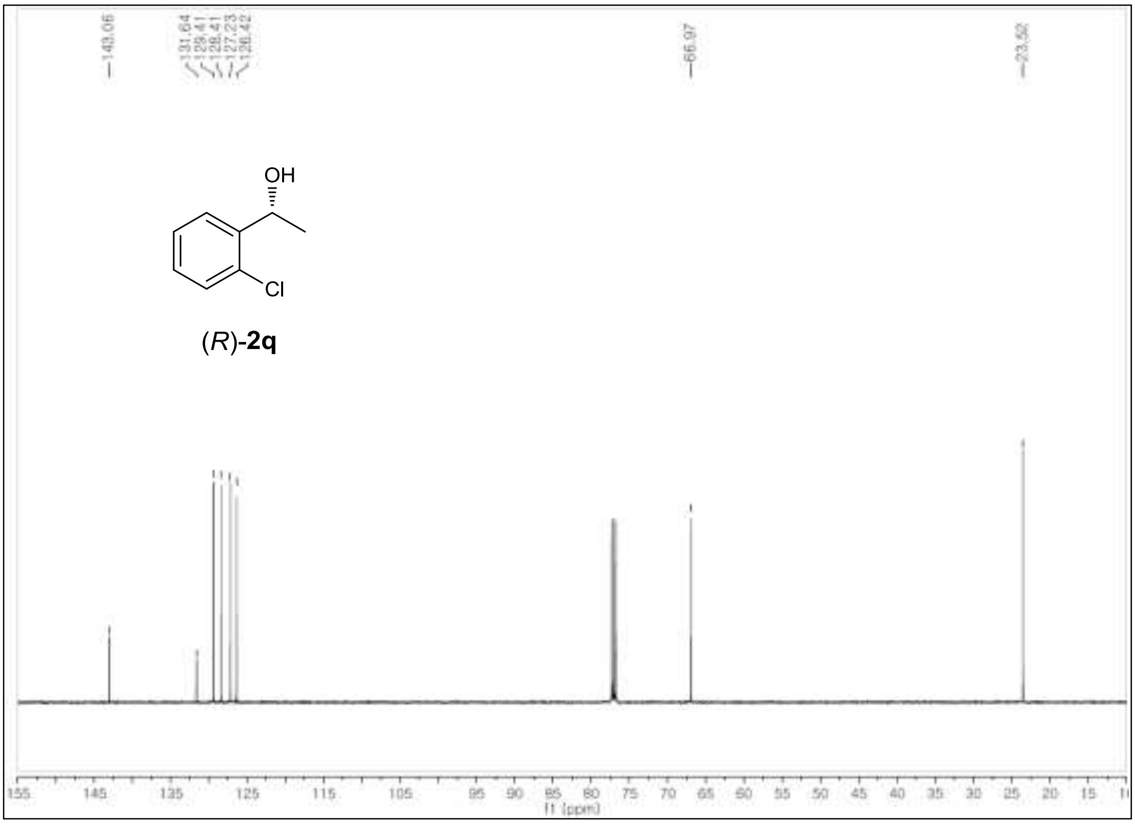
143.06
131.64
129.41
128.41
127.23
126.42
66.97
23.52
OH
Cl
(*R*)-**2q**
f1 (ppm)

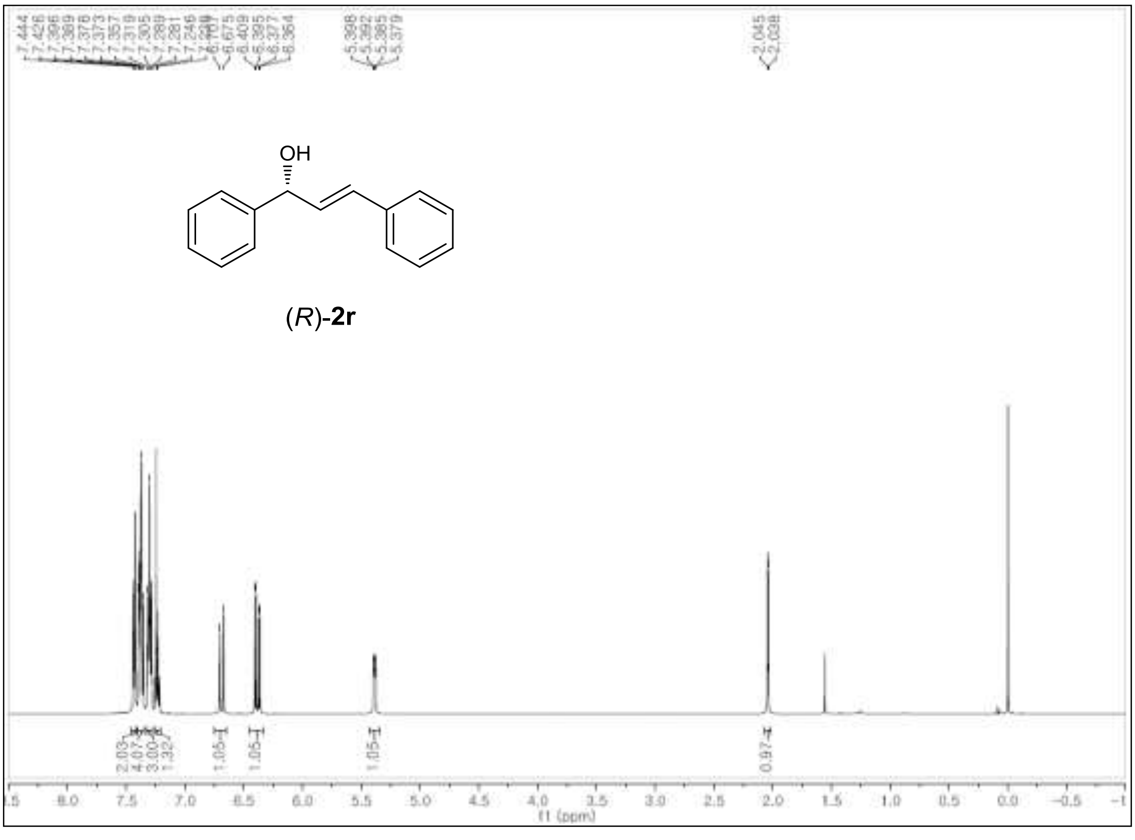
7.444
7.426
7.396
7.389
7.376
7.373
7.357
7.319
7.305
7.289
7.281
7.246
6.675
6.409
6.395
6.377
6.364
5.398
5.392
5.385
5.379
2.045
2.038
OH
(*R*)-**2r**
2.03
4.07
3.00
1.32
1.05
1.05
1.05
0.97
f1 (ppm)

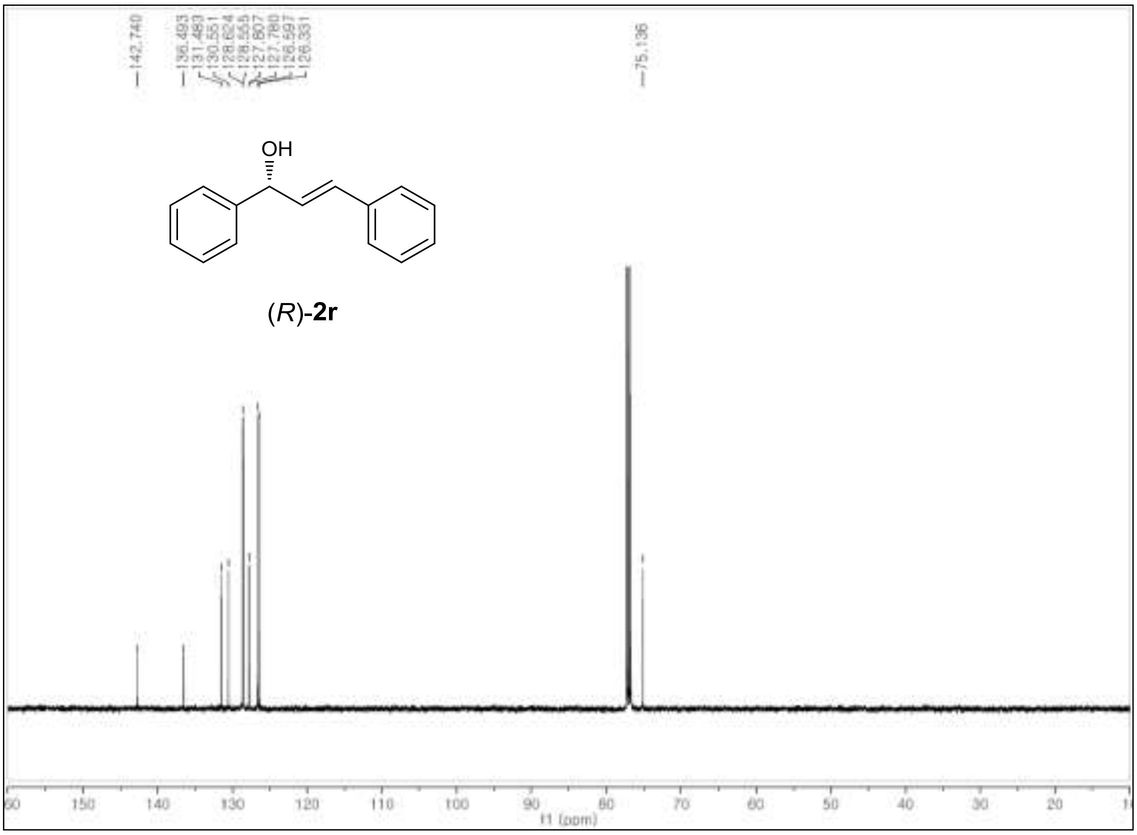

142.740
136.493
131.483
130.551
128.624
128.555
127.807
127.780
126.597
126.331
75.136
OH
(R)-2r
160
150
140
130
120
110
100
90
80
70
60
50
40
30
20
10
f1 (ppm)

Supplementary Figure 13. ^{1}H, ^{13}C and ^{19}F NMR spectra of the TMS-ether products **3a–3r**

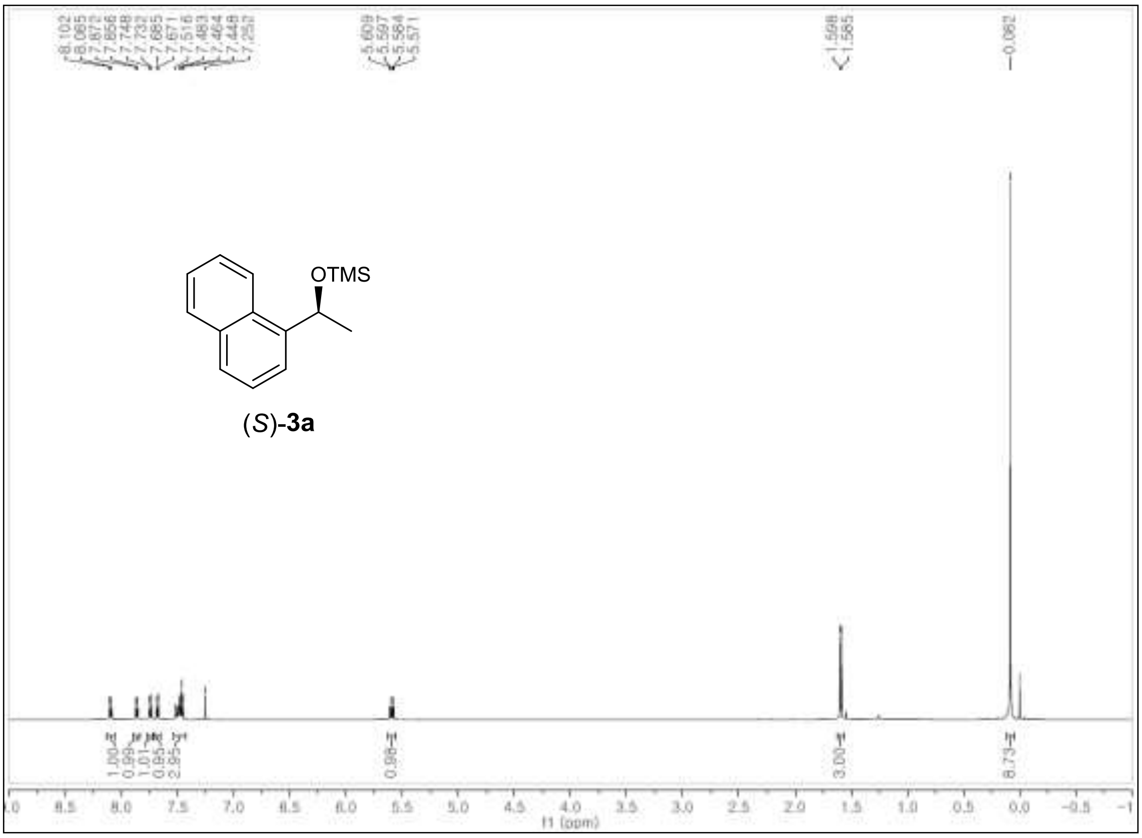

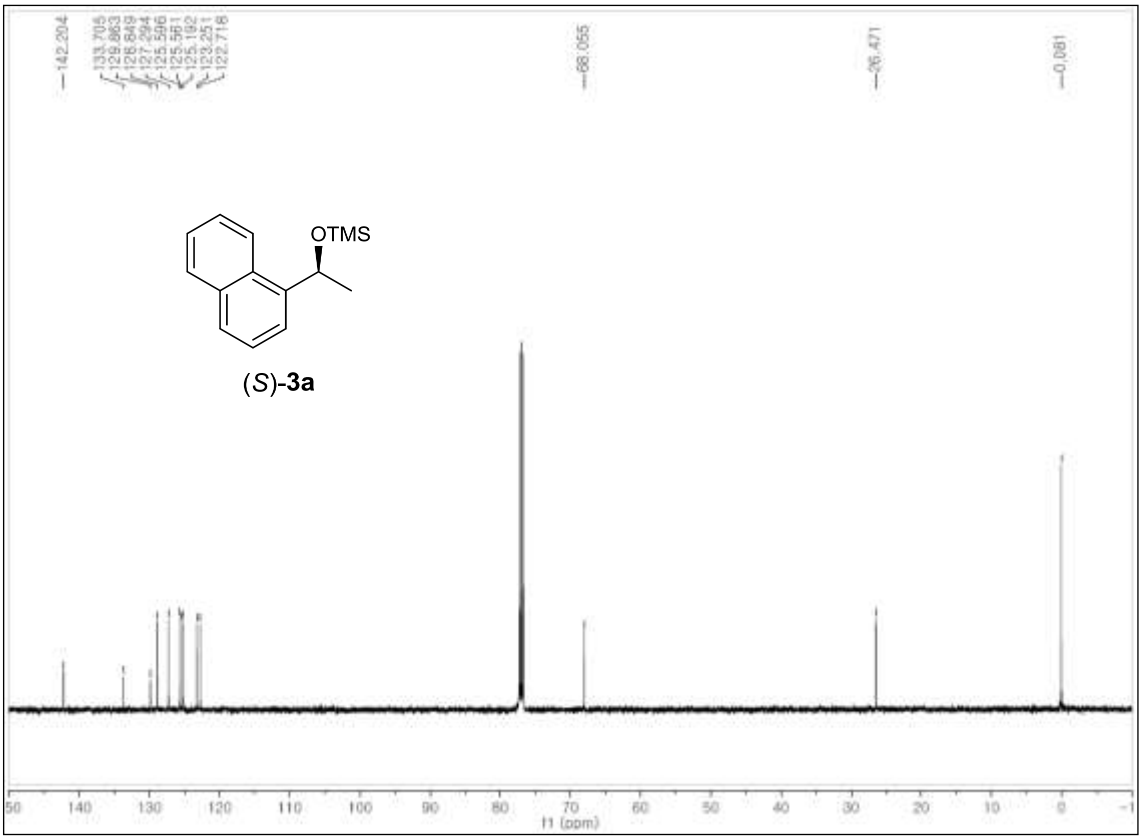

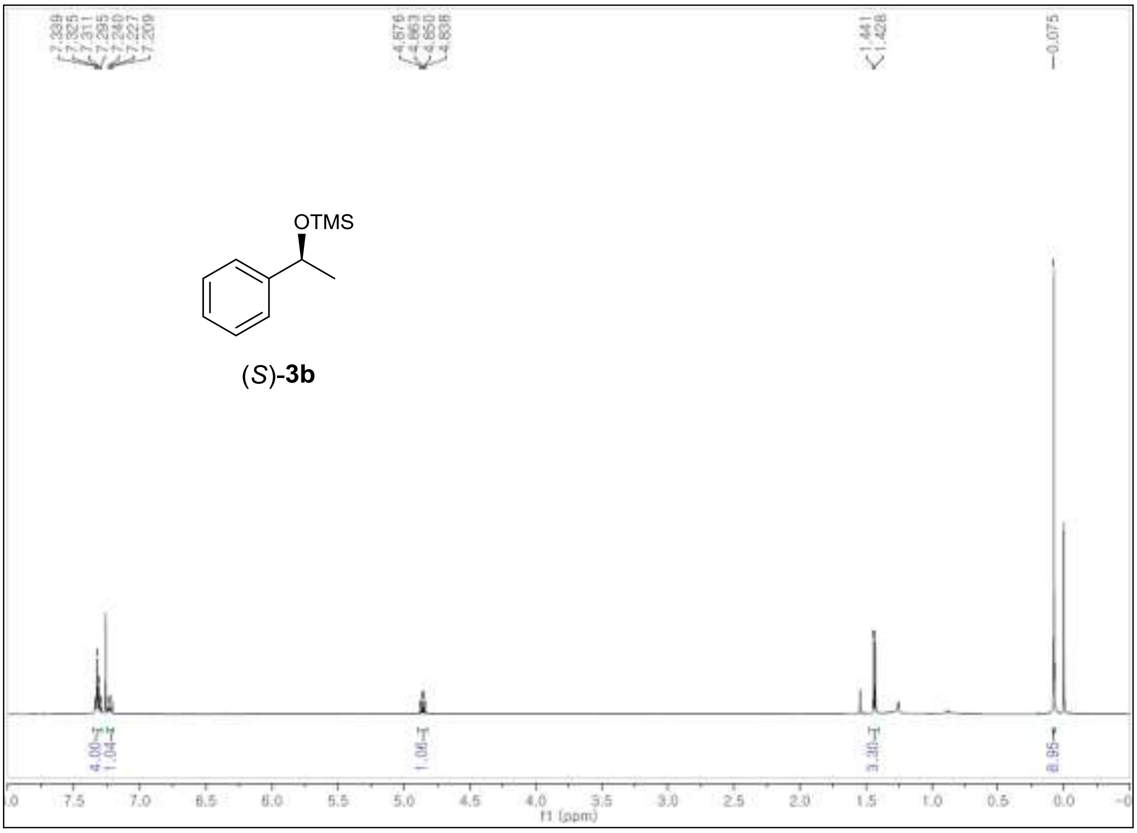

7.339
7.325
7.311
7.295
7.240
7.227
7.209
4.876
4.863
4.850
4.838
1.441
1.428
0.075
OTMS
(S)-3b
4.00
1.04
1.06
3.30
8.95
f1 (ppm)

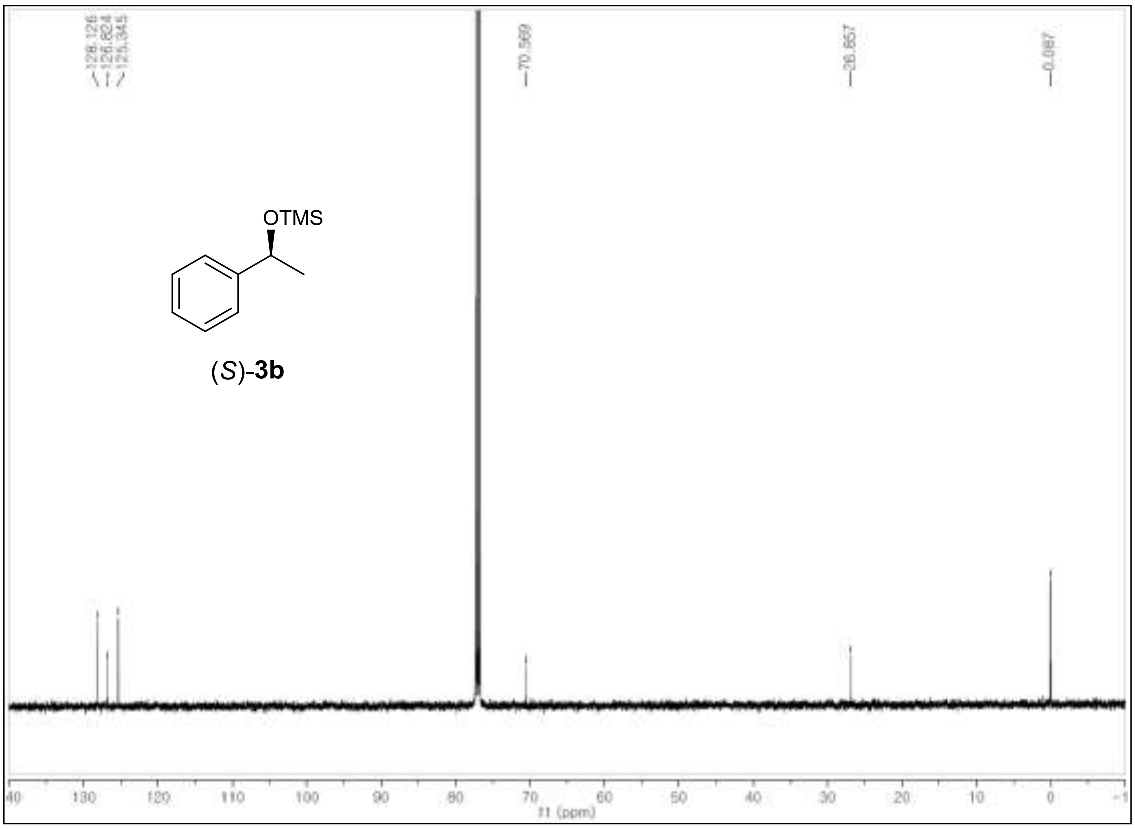

128.126
126.824
125.345
70.569
26.857
0.087
OTMS
(S)-3b
f1 (ppm)

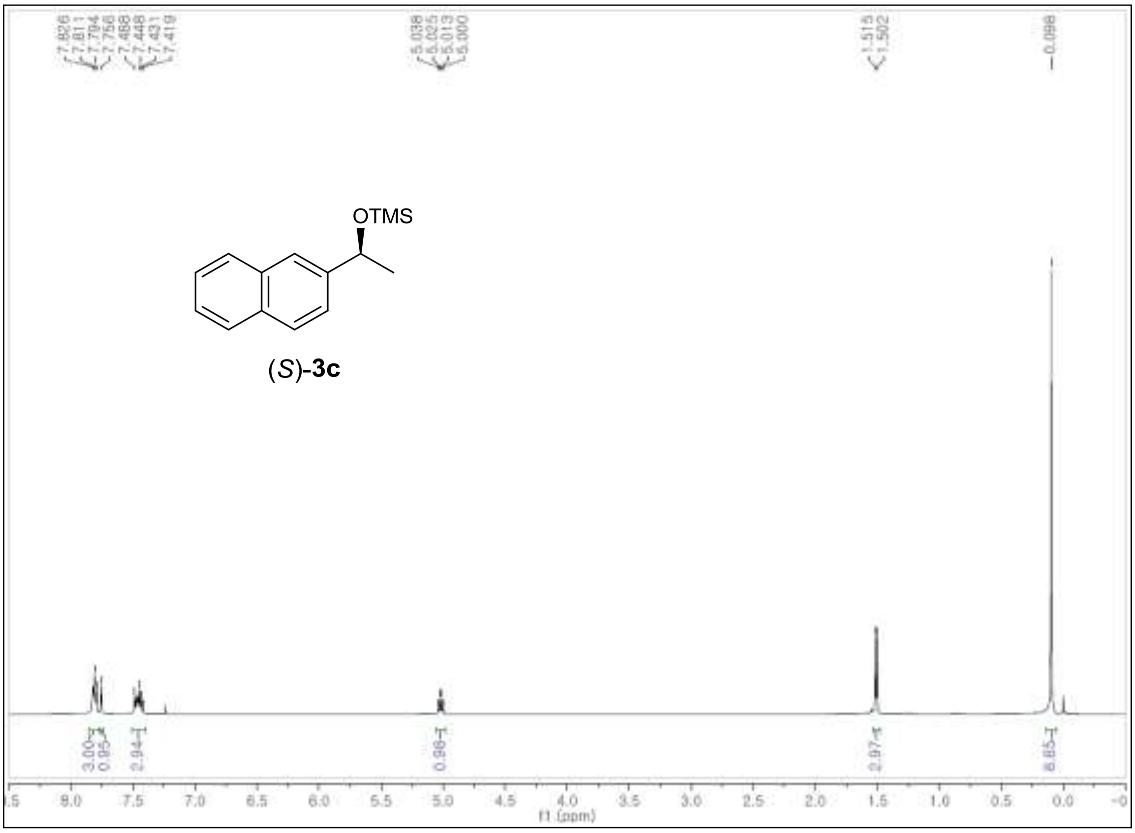

7.826
7.811
7.794
7.756
7.488
7.448
7.431
7.419
5.038
5.025
5.013
5.000
1.515
1.502
0.098
OTMS
(S)-3c
3.00
0.95
2.94
0.98
2.97
8.85
f1 (ppm)

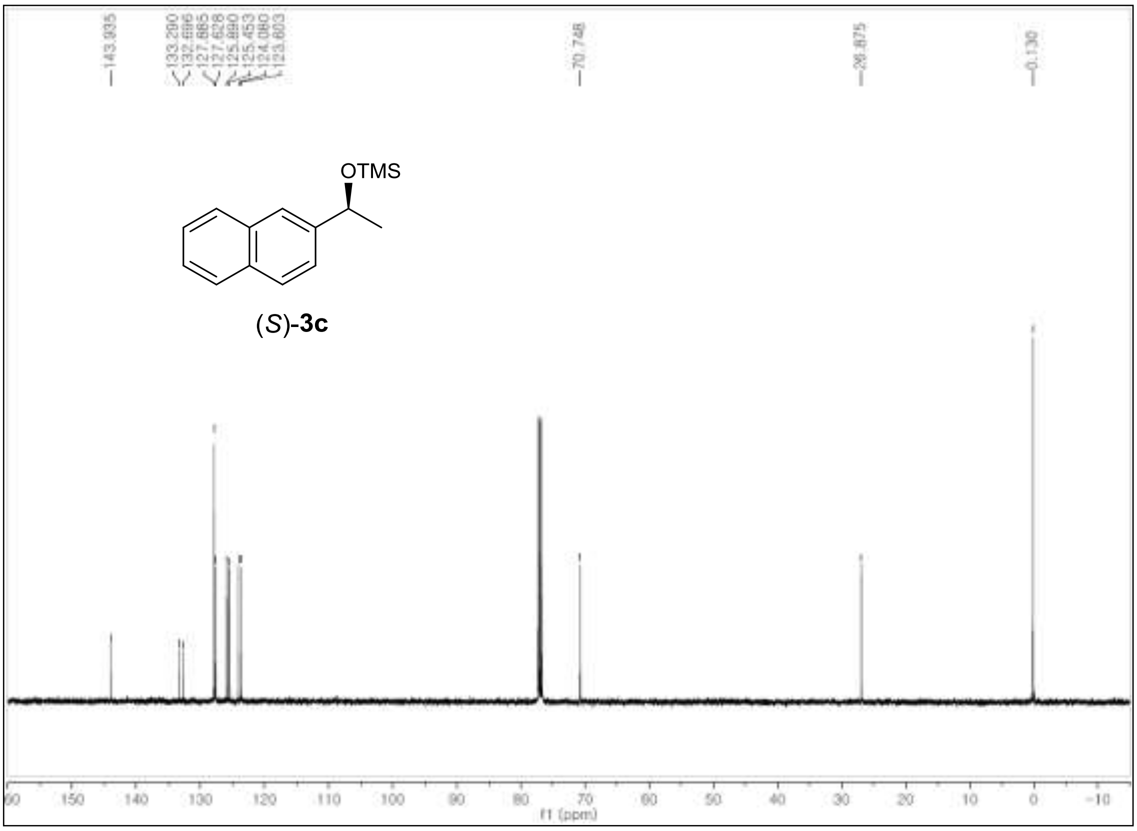

143.935
133.290
132.896
127.885
127.628
125.890
125.453
124.080
123.803
70.748
26.875
0.130
OTMS
(S)-3c
f1 (ppm)

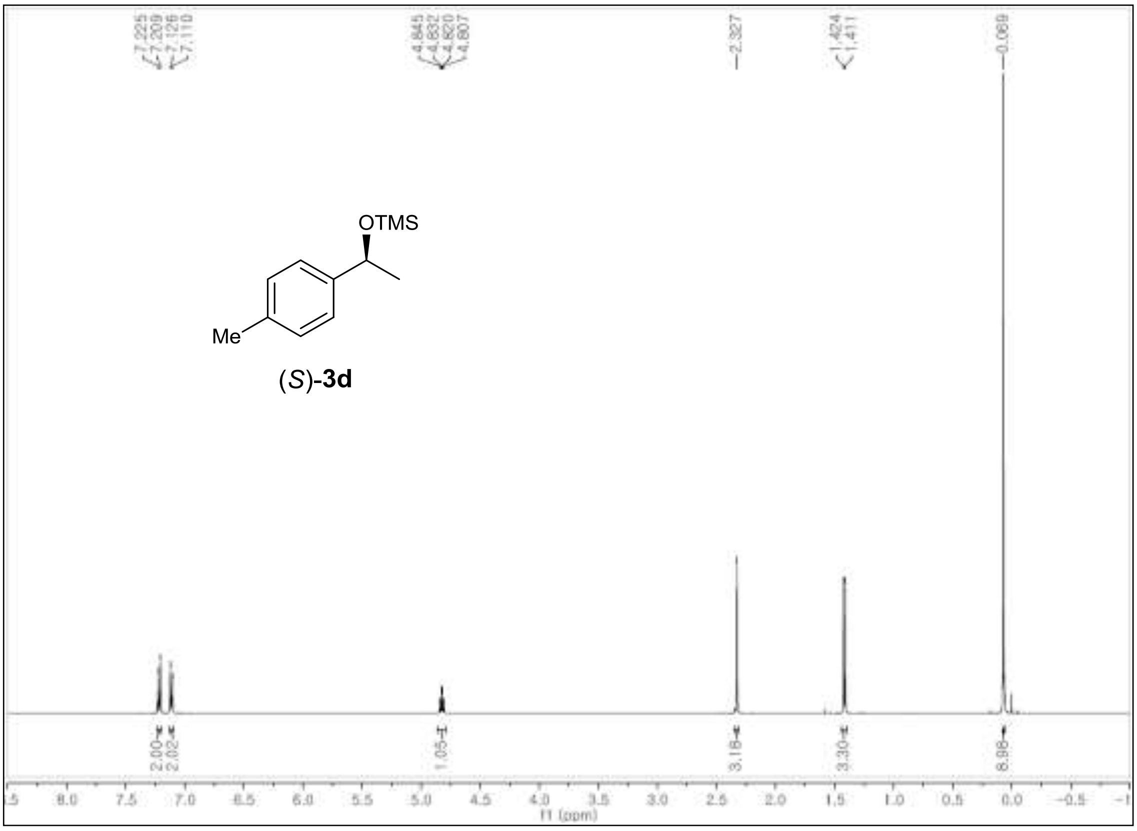

7.225
7.209
7.126
7.110
4.845
4.832
4.820
4.807
2.327
1.424
1.411
0.069
OTMS
Me
(*S*)-**3d**
2.00
2.02
1.05
3.16
3.30
8.98
f1 (ppm)

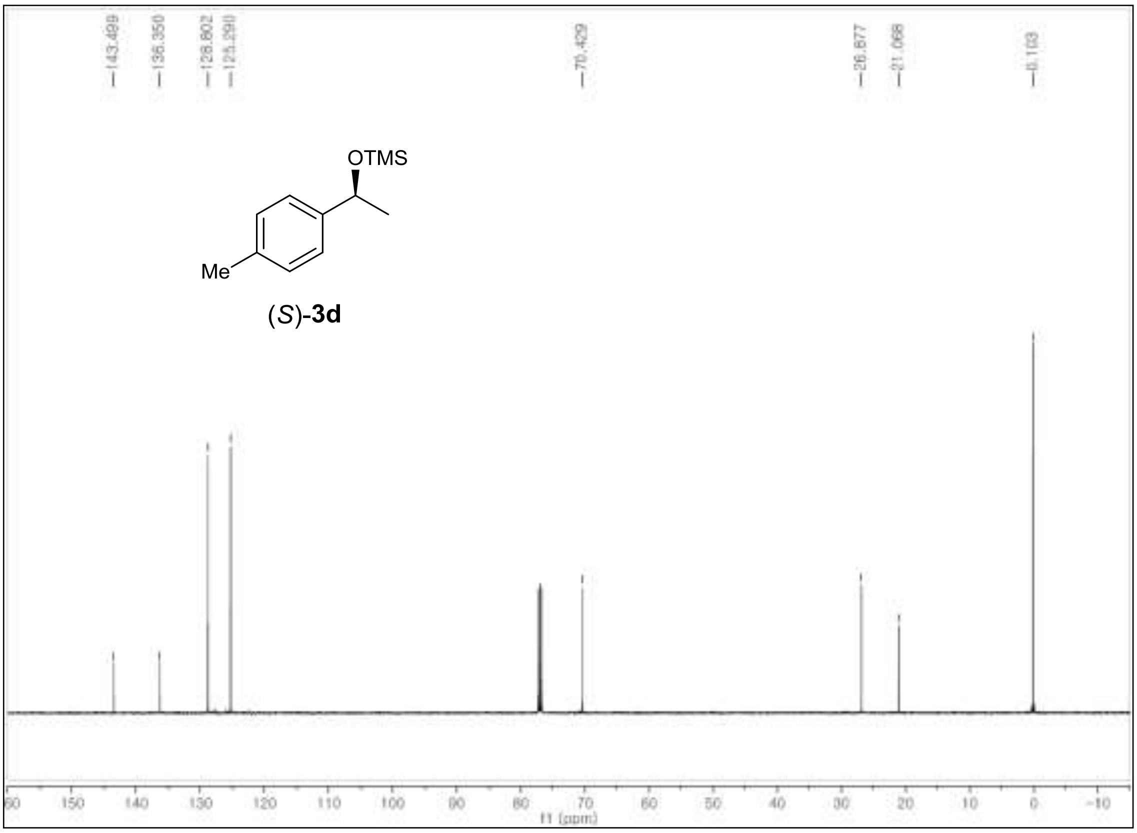

143.499
136.350
128.802
125.290
70.429
26.677
21.068
0.103
OTMS
Me
(*S*)-**3d**
f1 (ppm)

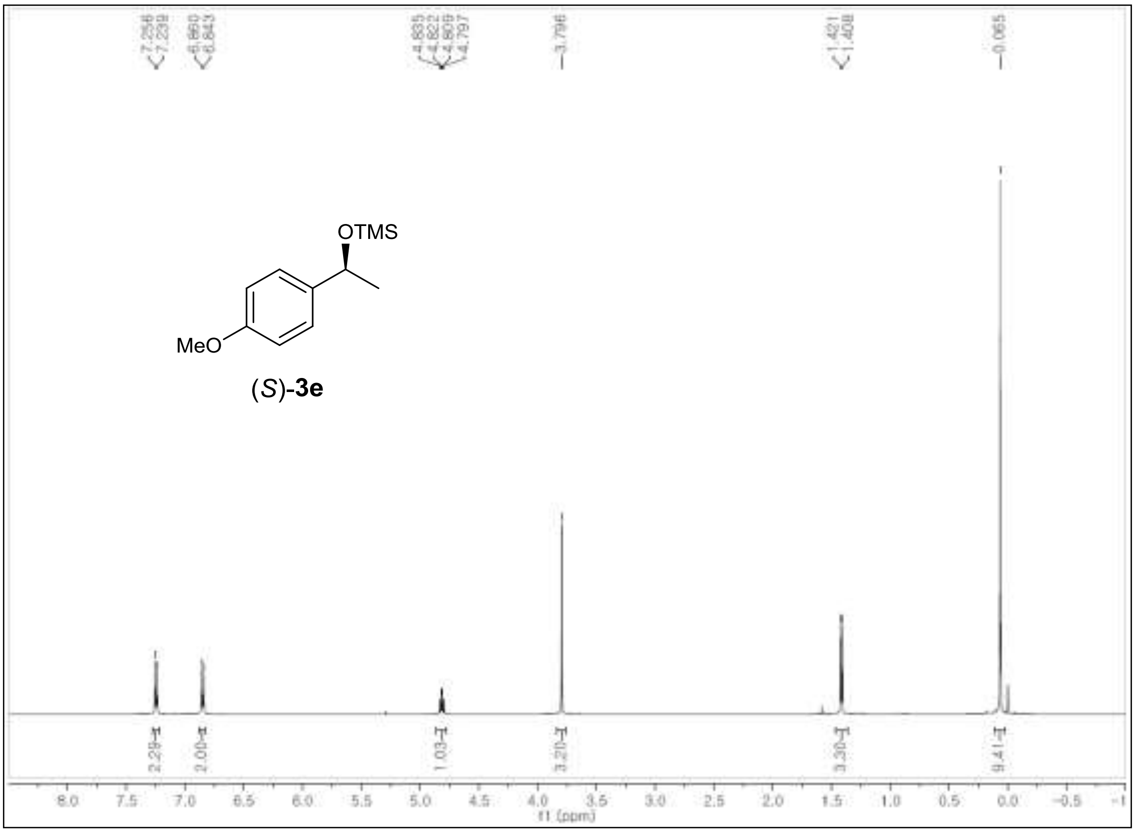
7.256
7.239
6.860
6.843
4.835
4.822
4.809
4.797
3.796
1.421
1.408
0.065
OTMS
MeO
(S)-3e
2.29
2.00
1.03
3.20
3.30
9.41
f1 (ppm)

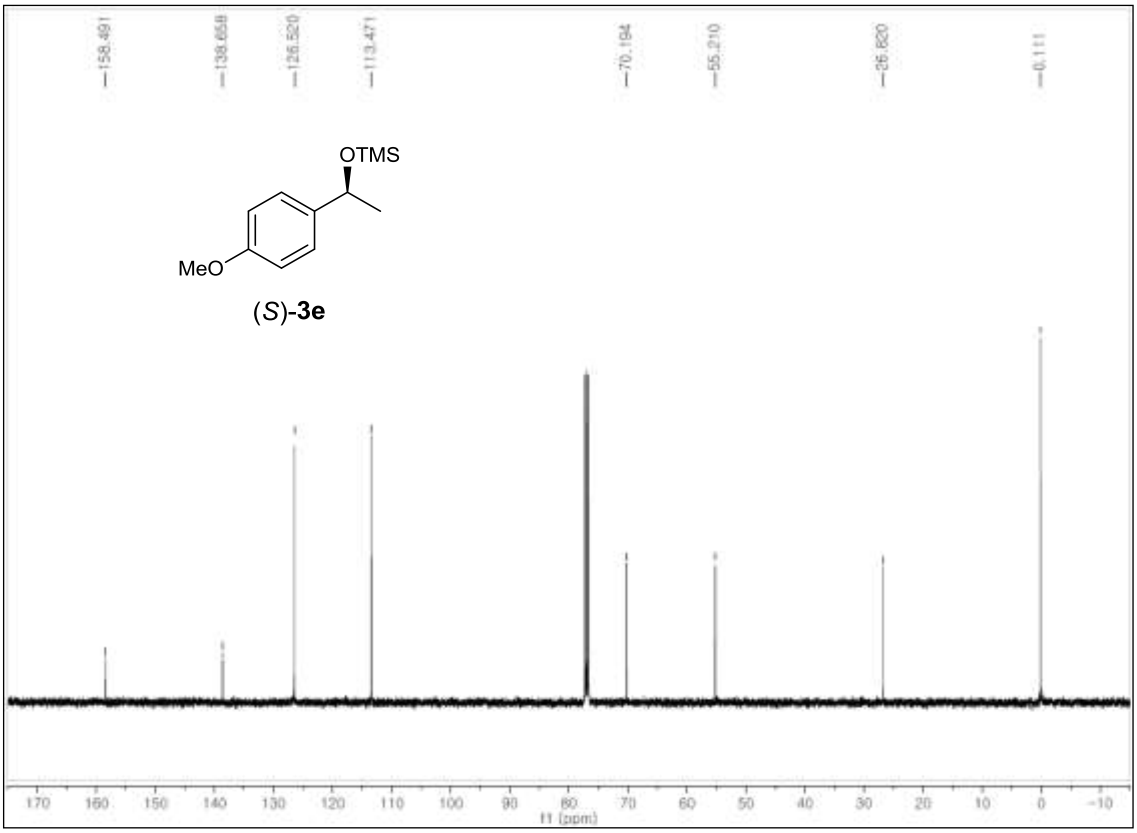
158.491
138.658
126.520
113.471
70.194
55.210
26.620
0.111
OTMS
MeO
(S)-3e
f1 (ppm)

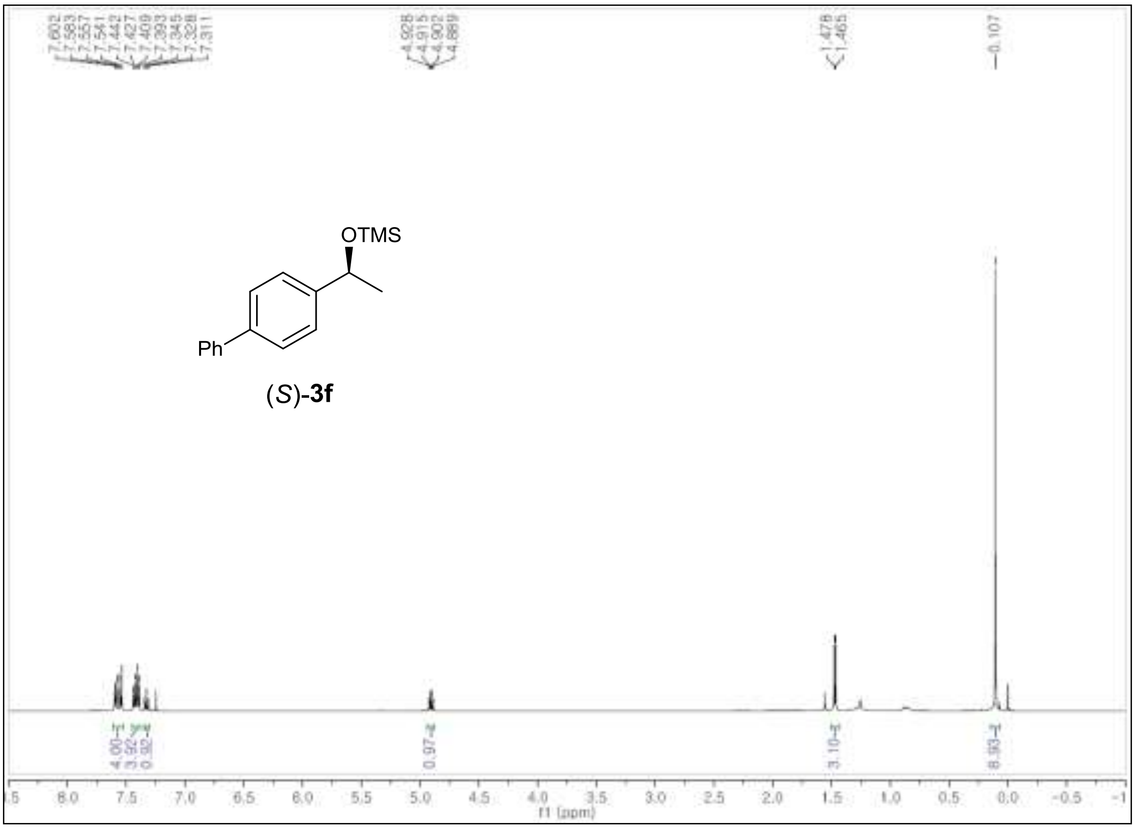
OTMS
Ph
(S)-3f
f1 (ppm)

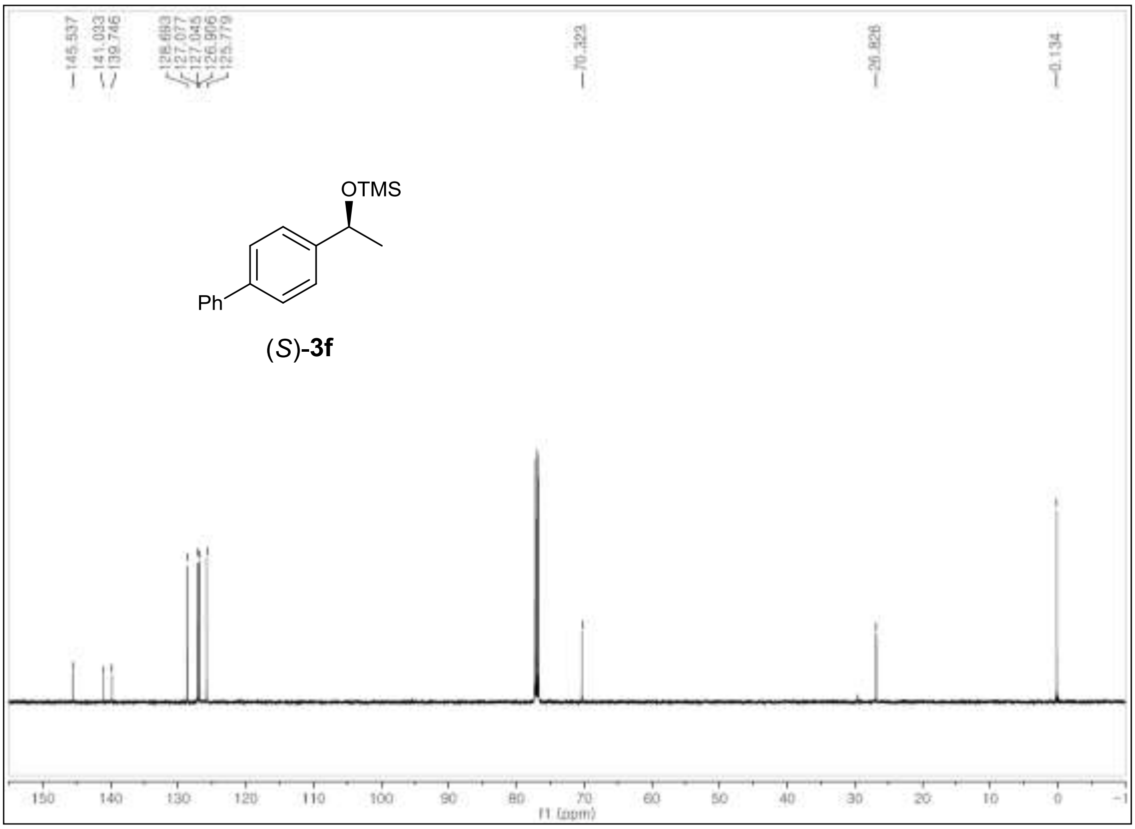
OTMS
Ph
(S)-3f
f1 (ppm)

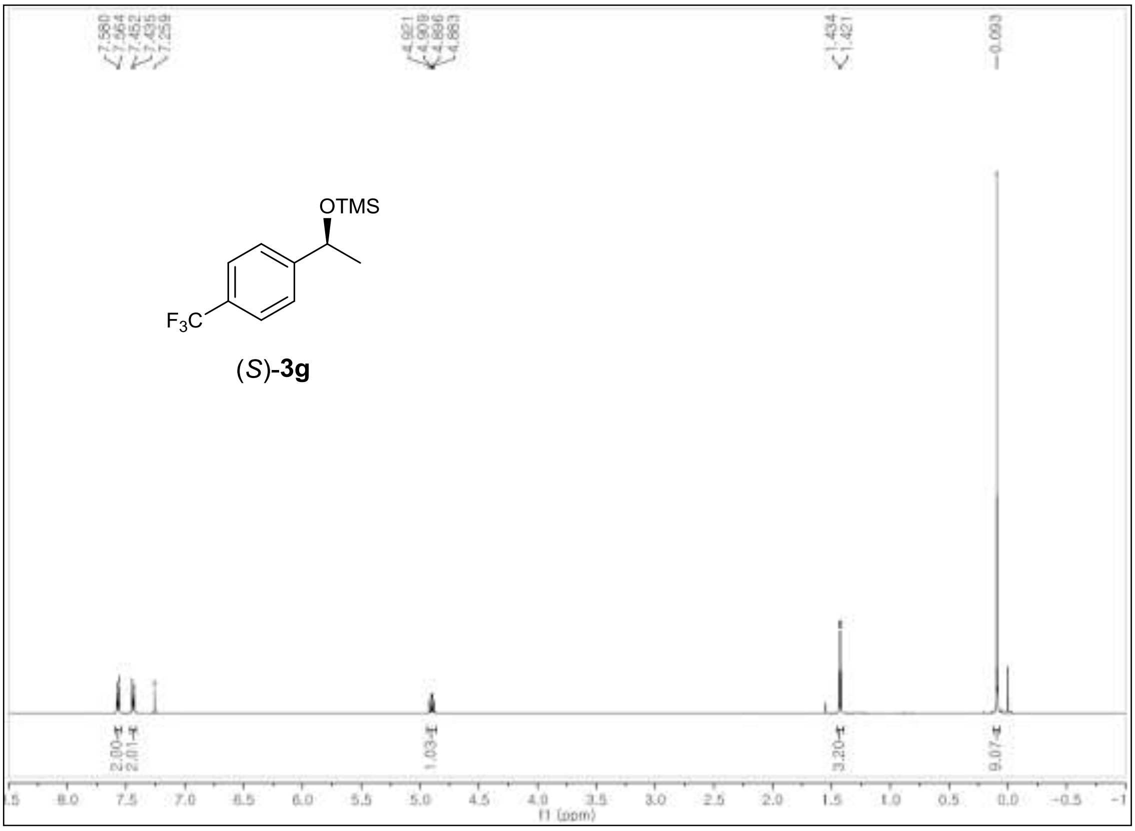
7.580
7.564
7.452
7.435
7.259
4.921
4.908
4.896
4.883
1.434
1.421
0.093
OTMS
F3C
(S)-3g
2.00
2.01
1.03
3.20
9.07
f1 (ppm)

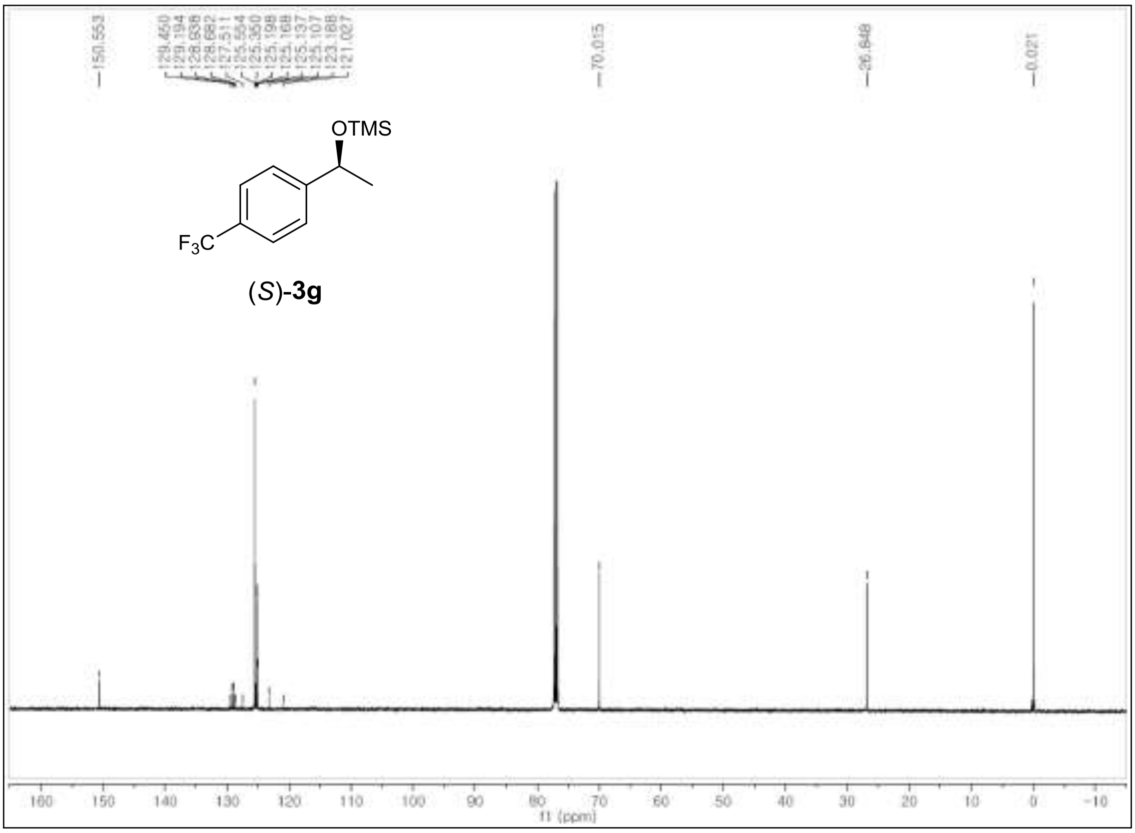
150.553
129.450
129.194
128.938
128.682
127.511
125.554
125.350
125.198
125.168
125.137
125.107
123.188
121.027
70.015
26.848
0.021
OTMS
F3C
(S)-3g
f1 (ppm)

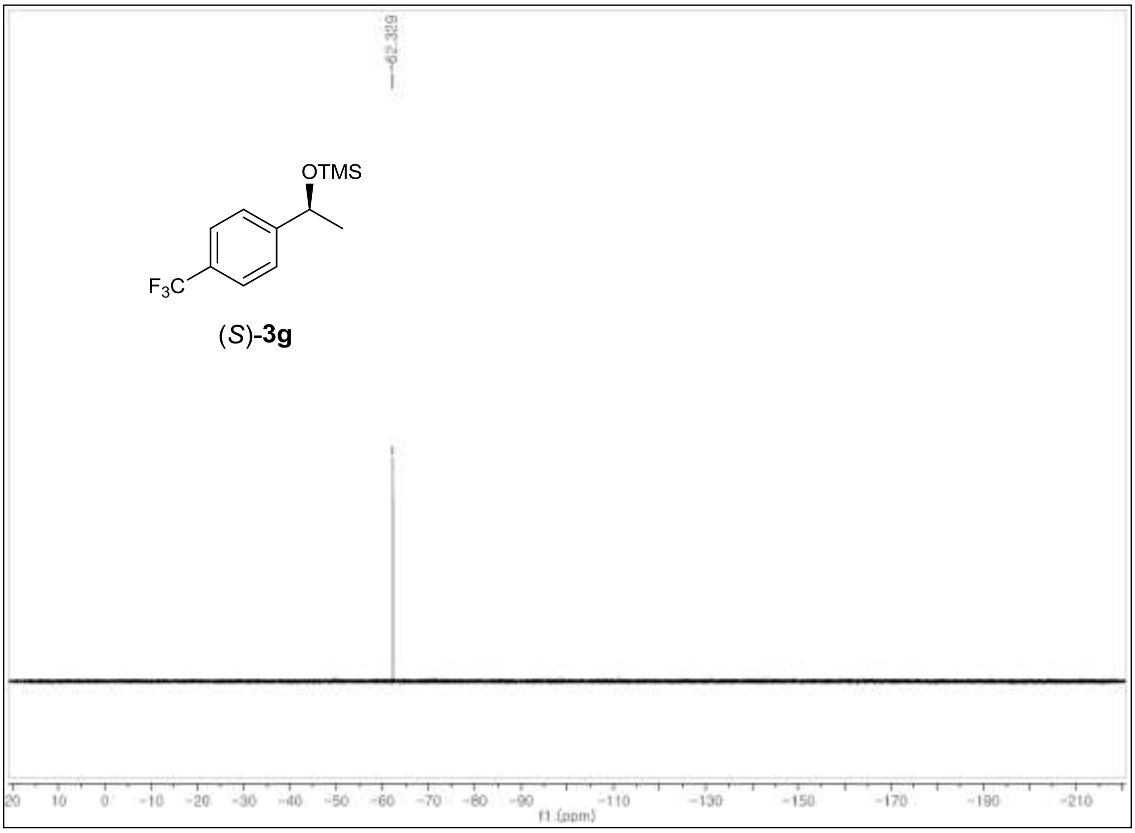
-62.329
OTMS
F3C
(S)-3g
f1 (ppm)

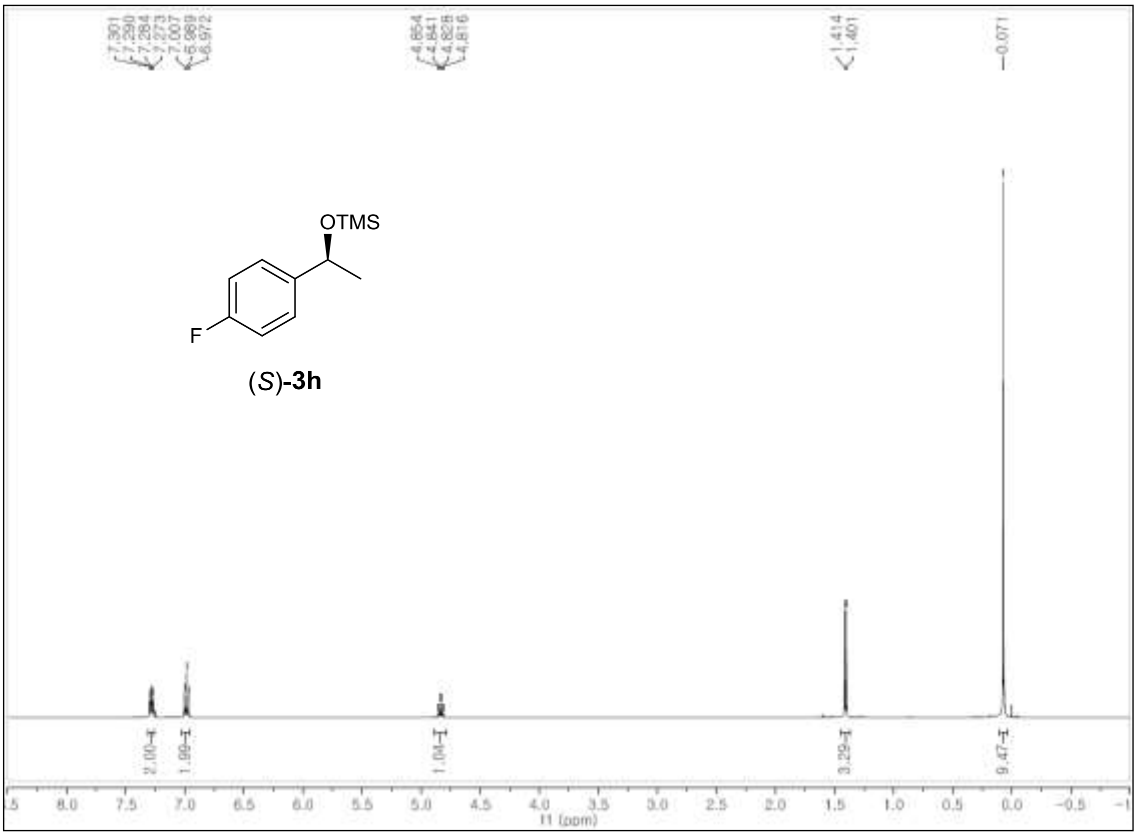
OTMS
F
(S)-3h
f1 (ppm)

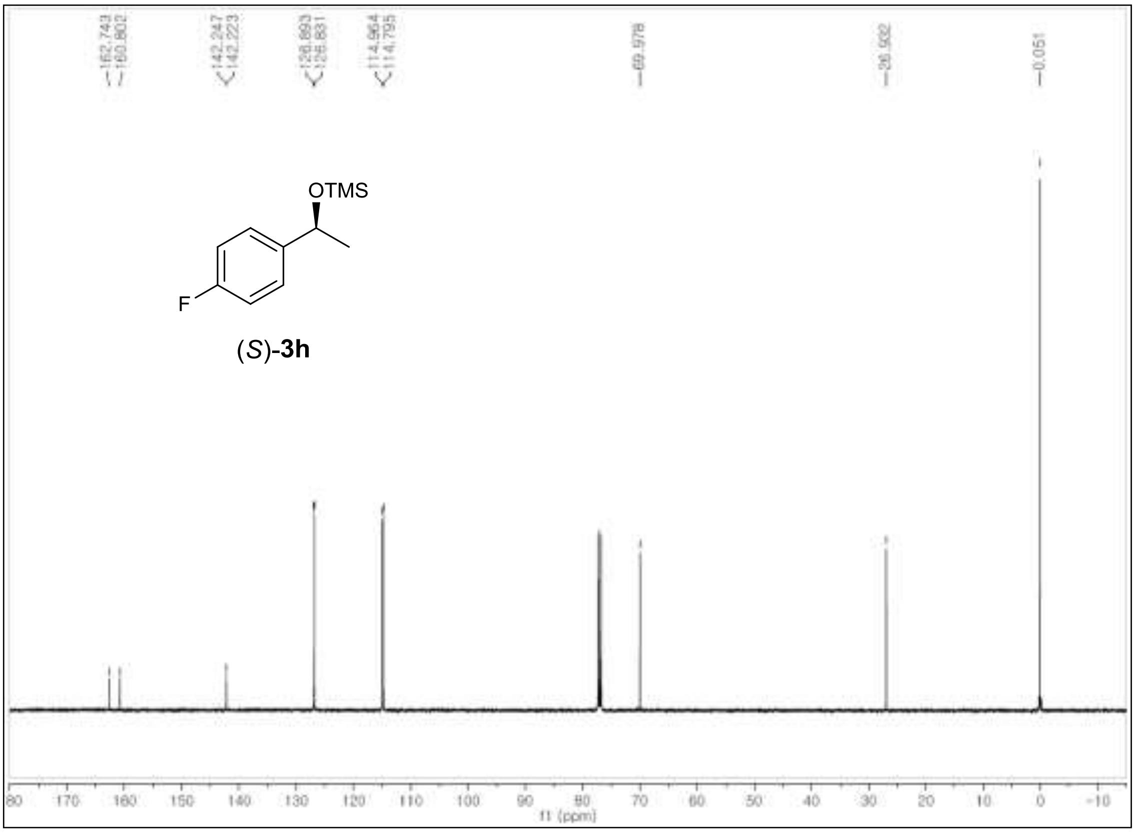
162.743
160.802
142.247
142.223
126.893
126.831
114.964
114.795
69.978
26.902
0.051
OTMS
F
(S)-3h
f1 (ppm)

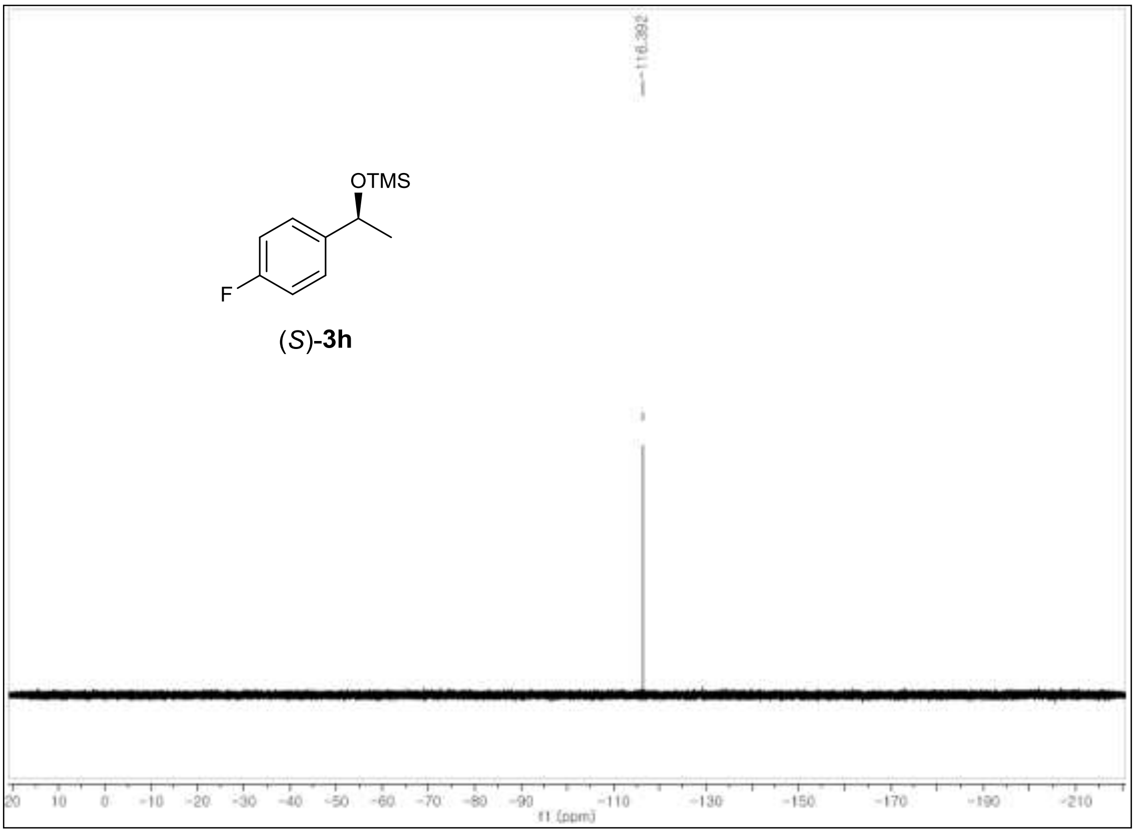
-116.392
OTMS
F
(S)-3h
f1 (ppm)

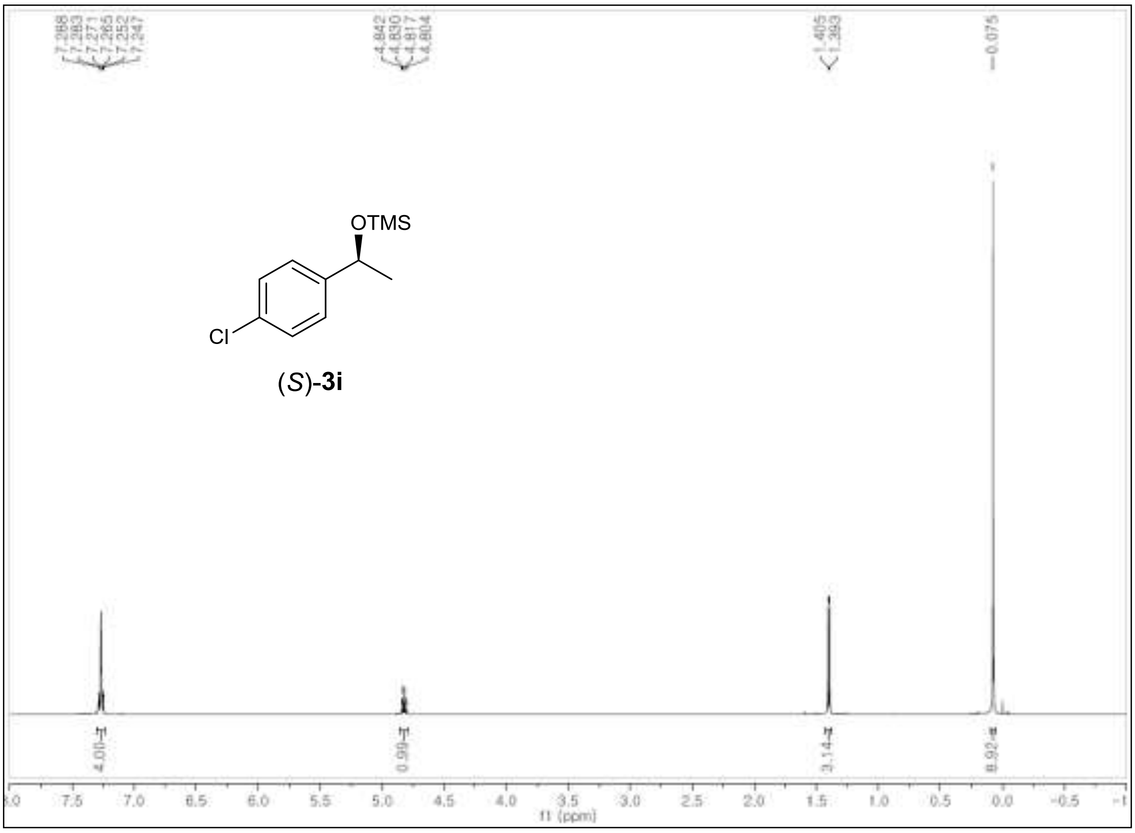

7.288
7.283
7.271
7.265
7.252
7.247
4.842
4.830
4.817
4.804
1.405
1.393
0.075
OTMS
Cl
(S)-3i
4.00
0.99
3.14
8.92
f1 (ppm)

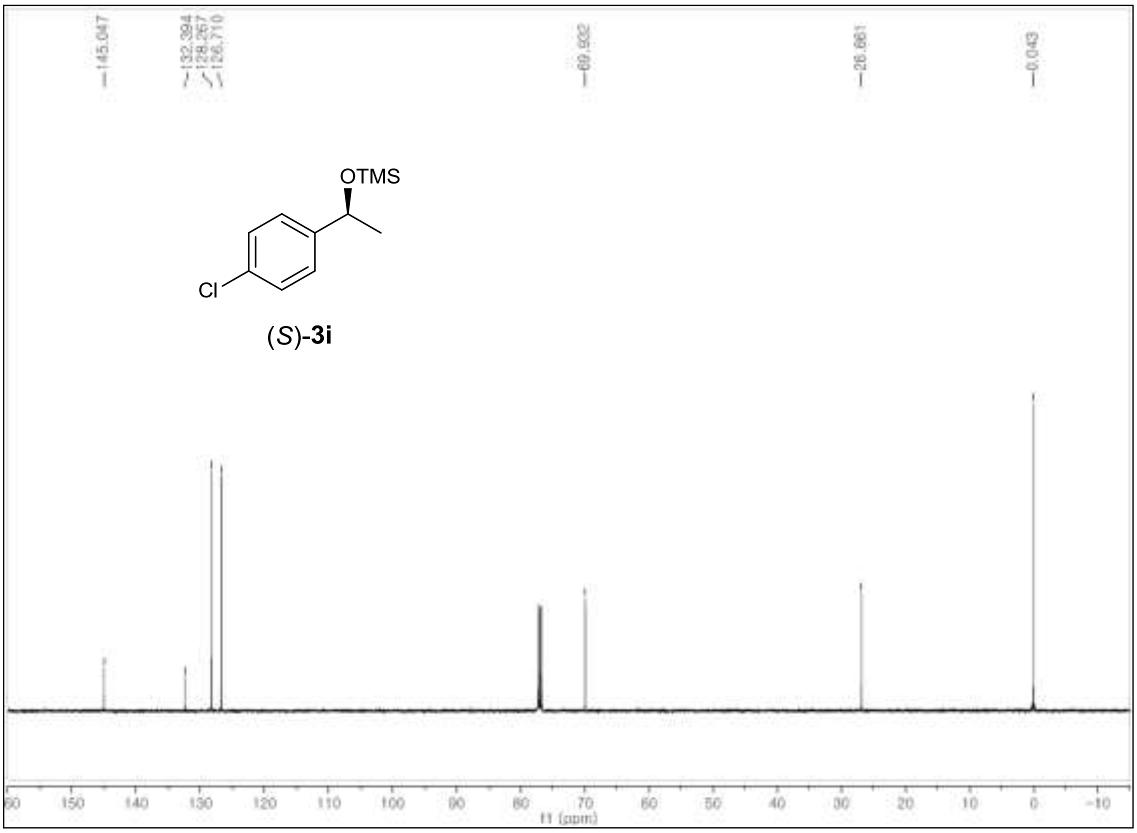

145.047
132.394
128.267
126.710
69.932
26.661
0.043
OTMS
Cl
(S)-3i
f1 (ppm)

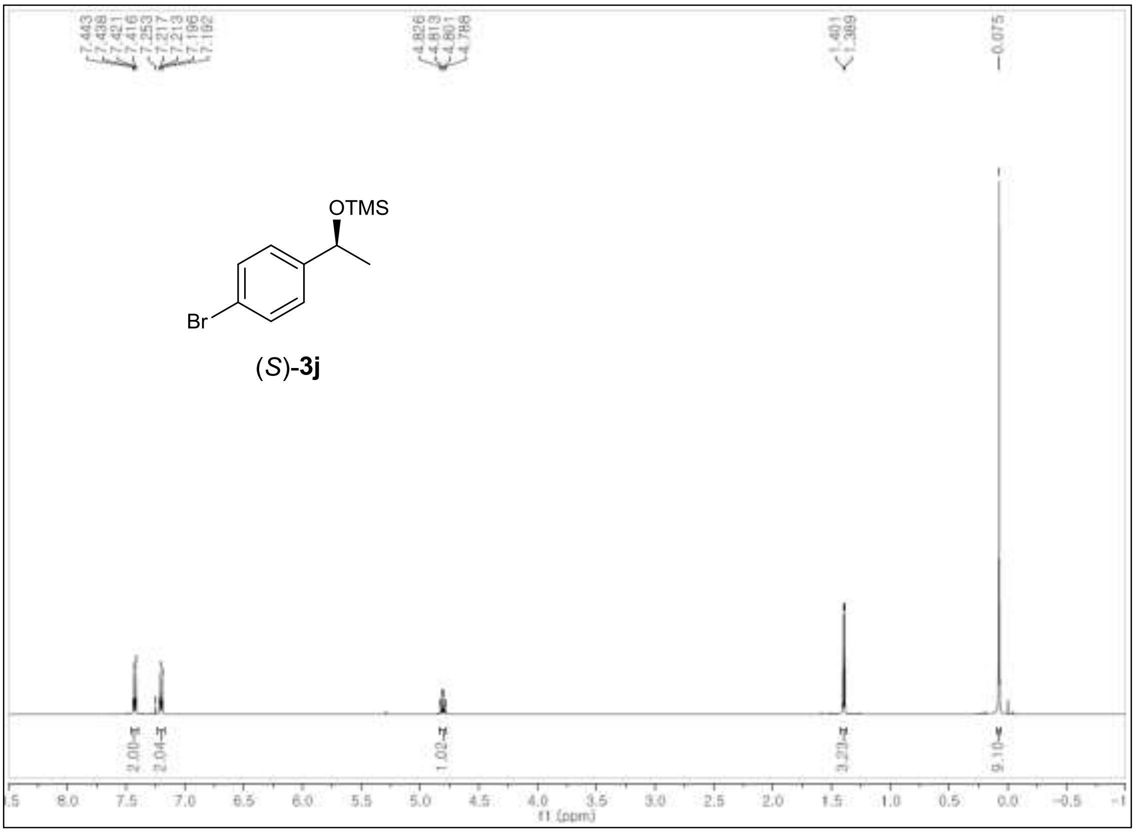
OTMS
Br
7.443
7.438
7.421
7.416
7.253
7.217
7.213
7.196
7.192
4.826
4.813
4.801
4.788
1.401
1.389
0.075
2.00
2.04
1.02
3.23
9.10
f1 (ppm)

(*S*)-**3j**

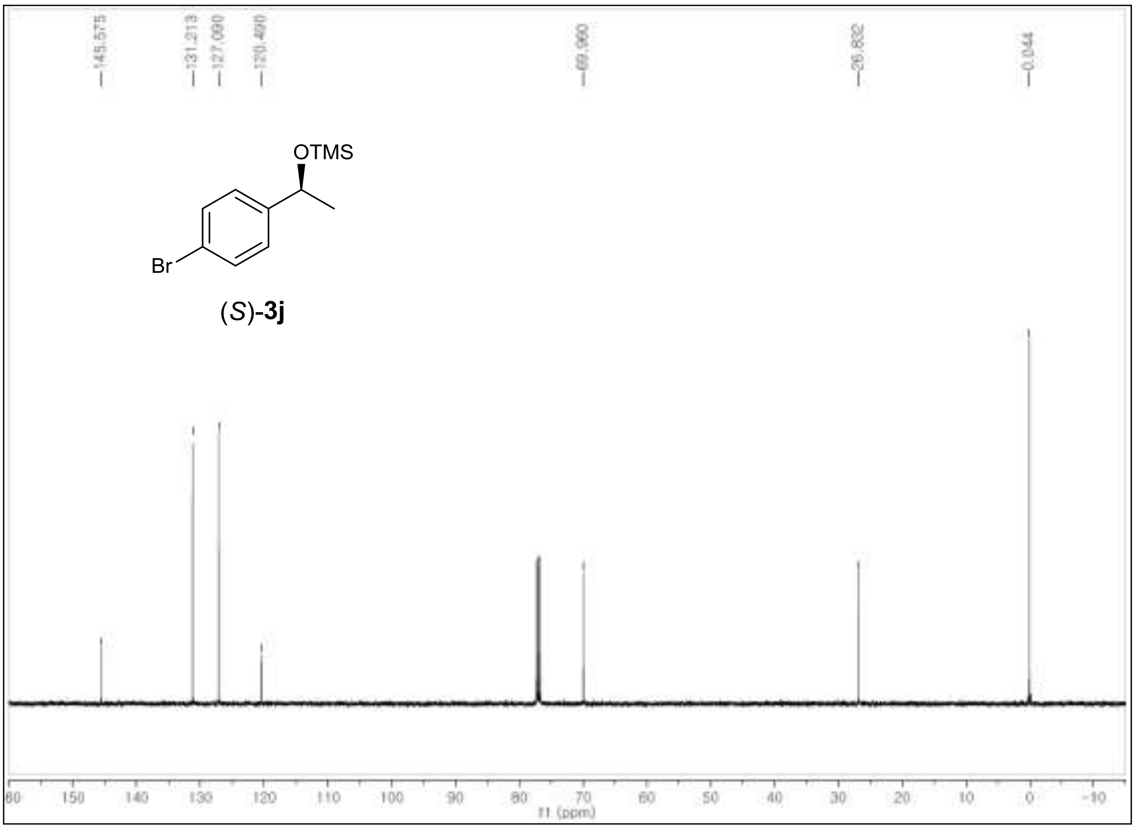
OTMS
Br
145.575
131.313
127.090
120.490
69.960
26.832
0.044
f1 (ppm)

(*S*)-**3j**

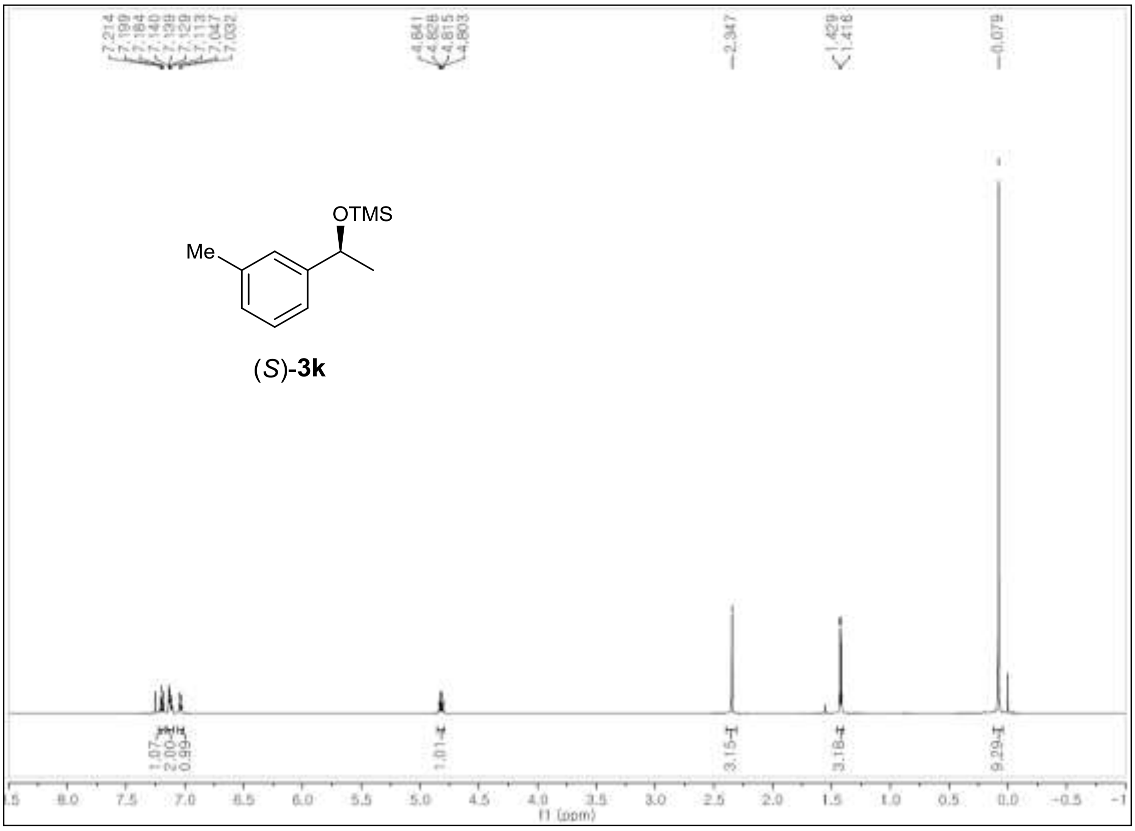
7.214
7.199
7.184
7.140
7.139
7.129
7.113
7.047
7.032
4.841
4.828
4.815
4.803
2.347
1.429
1.416
0.079
OTMS
Me
(S)-3k
1.07
2.00
0.99
1.01
3.15
3.18
9.29
f1 (ppm)

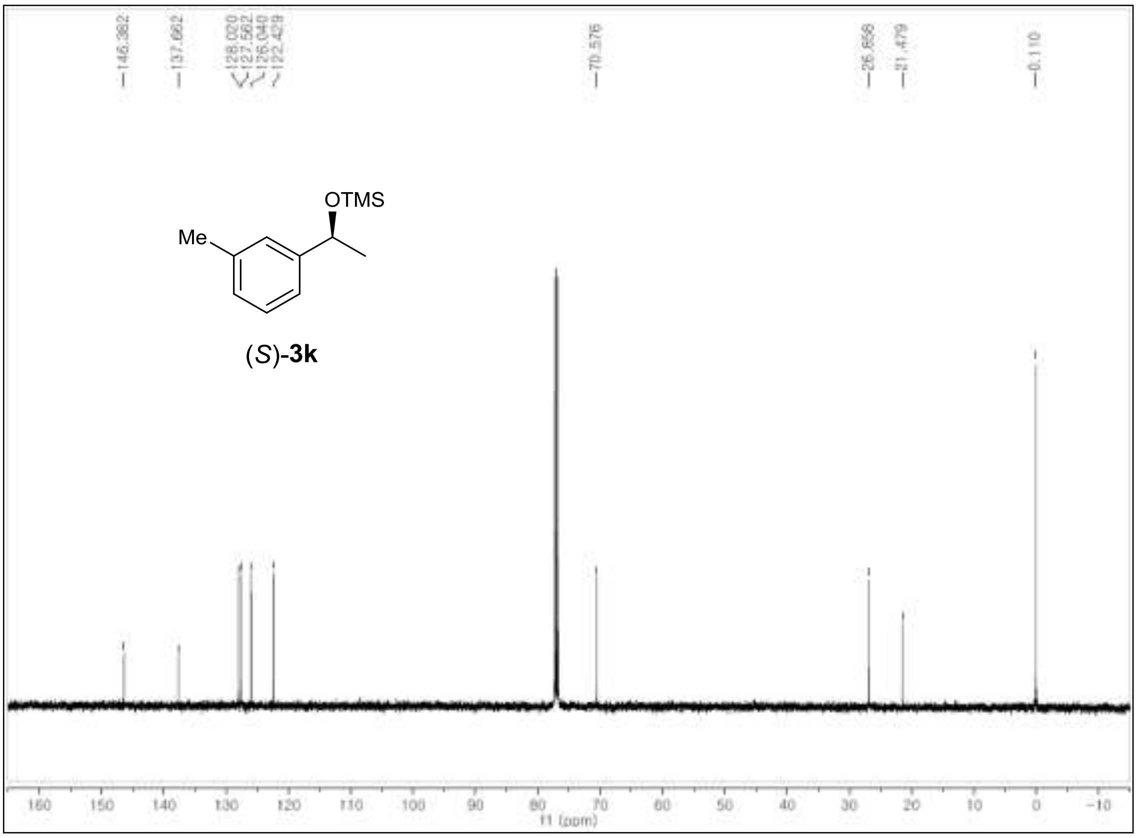
146.382
137.662
128.020
127.562
126.040
122.429
70.576
26.658
21.479
0.110
OTMS
Me
(S)-3k
f1 (ppm)

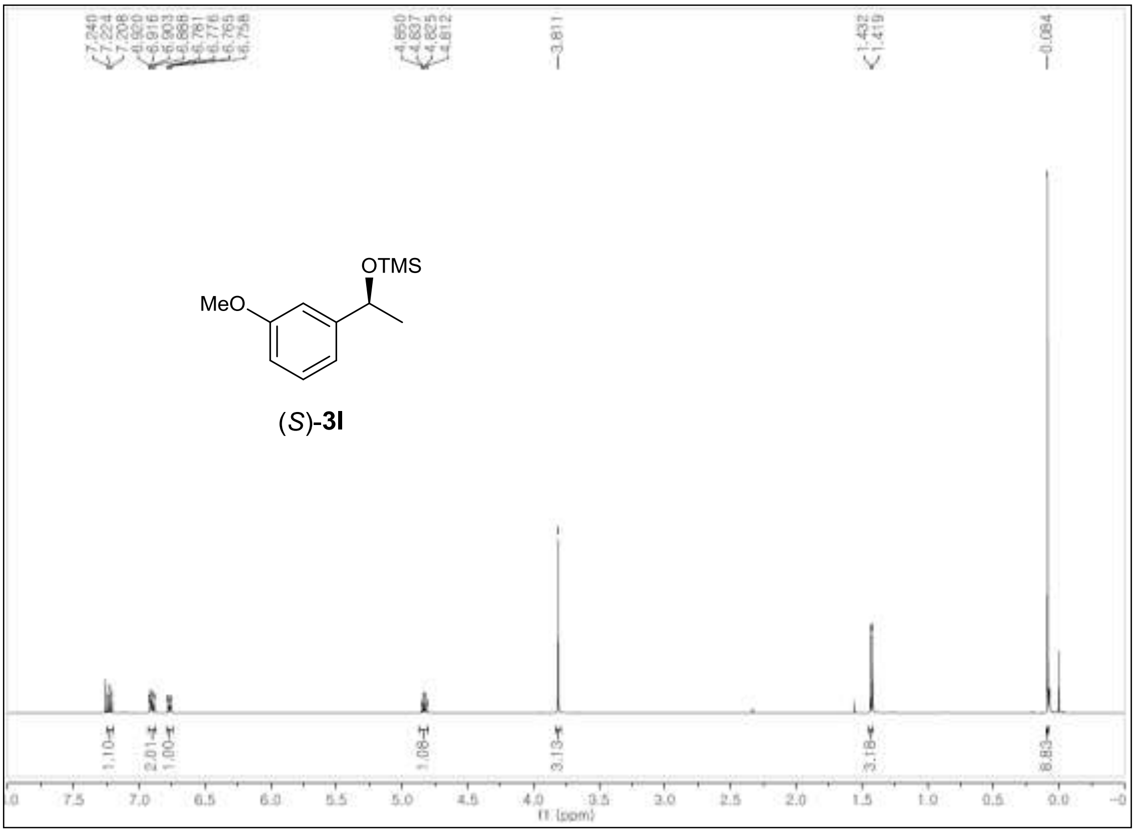

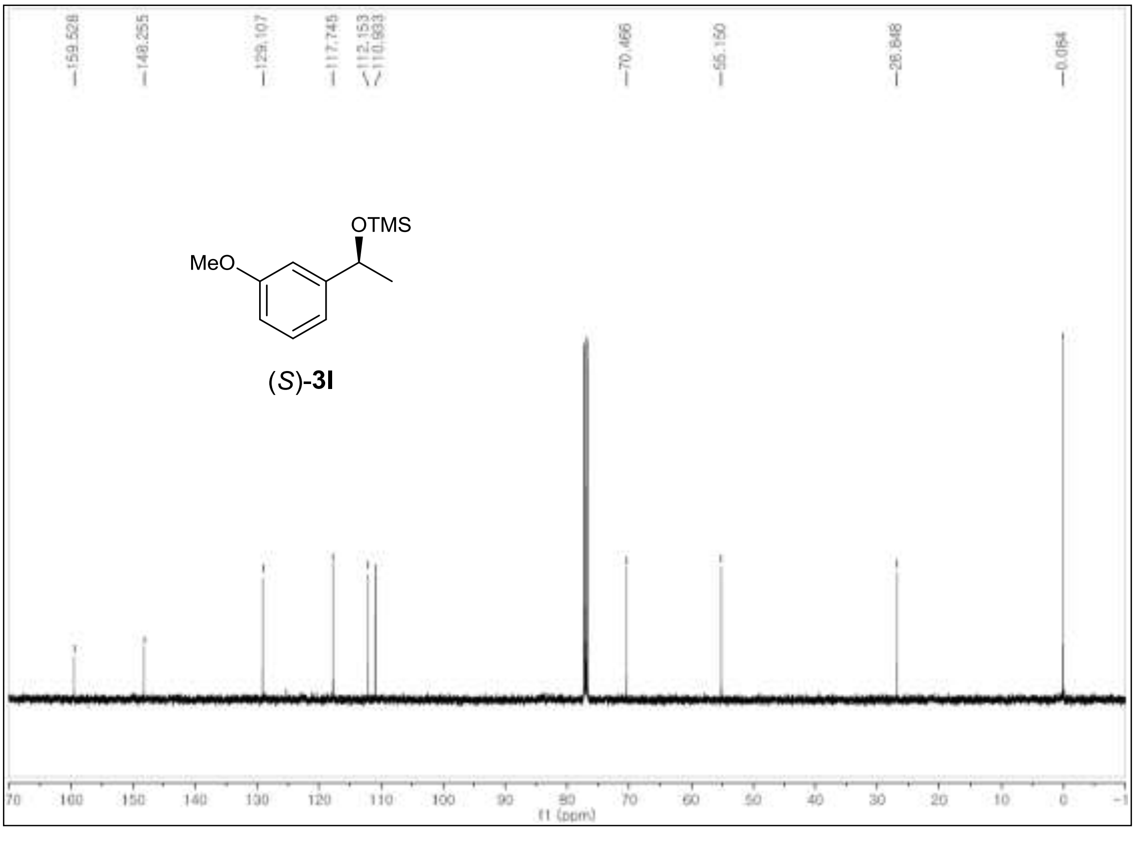

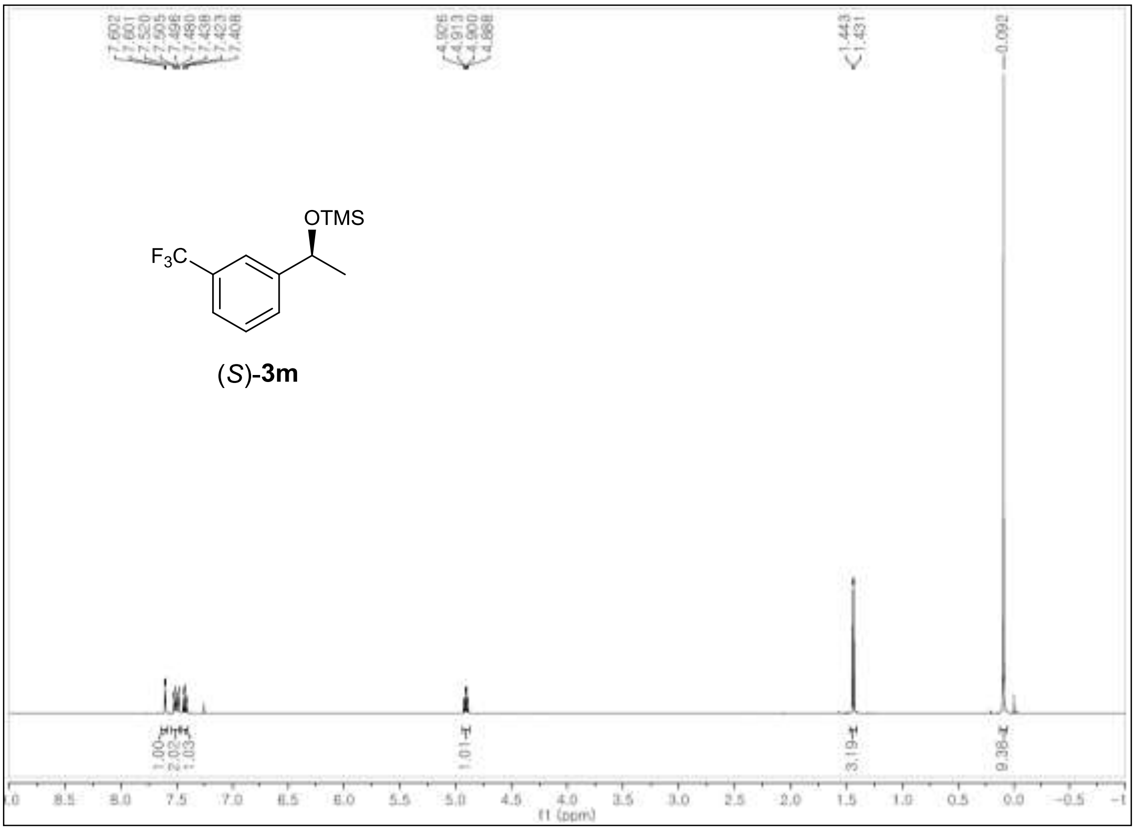
OTMS
F_3C
(*S*)-**3m**
f1 (ppm)

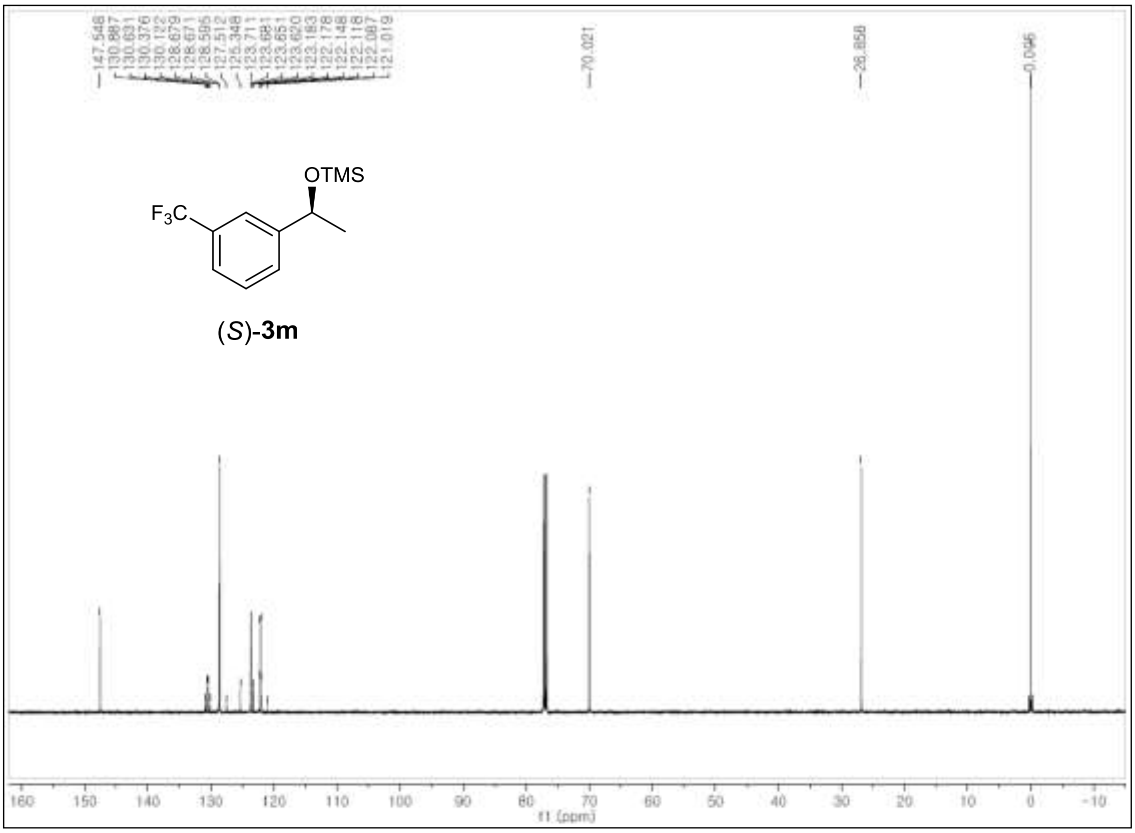
OTMS
F_3C
(*S*)-**3m**
f1 (ppm)

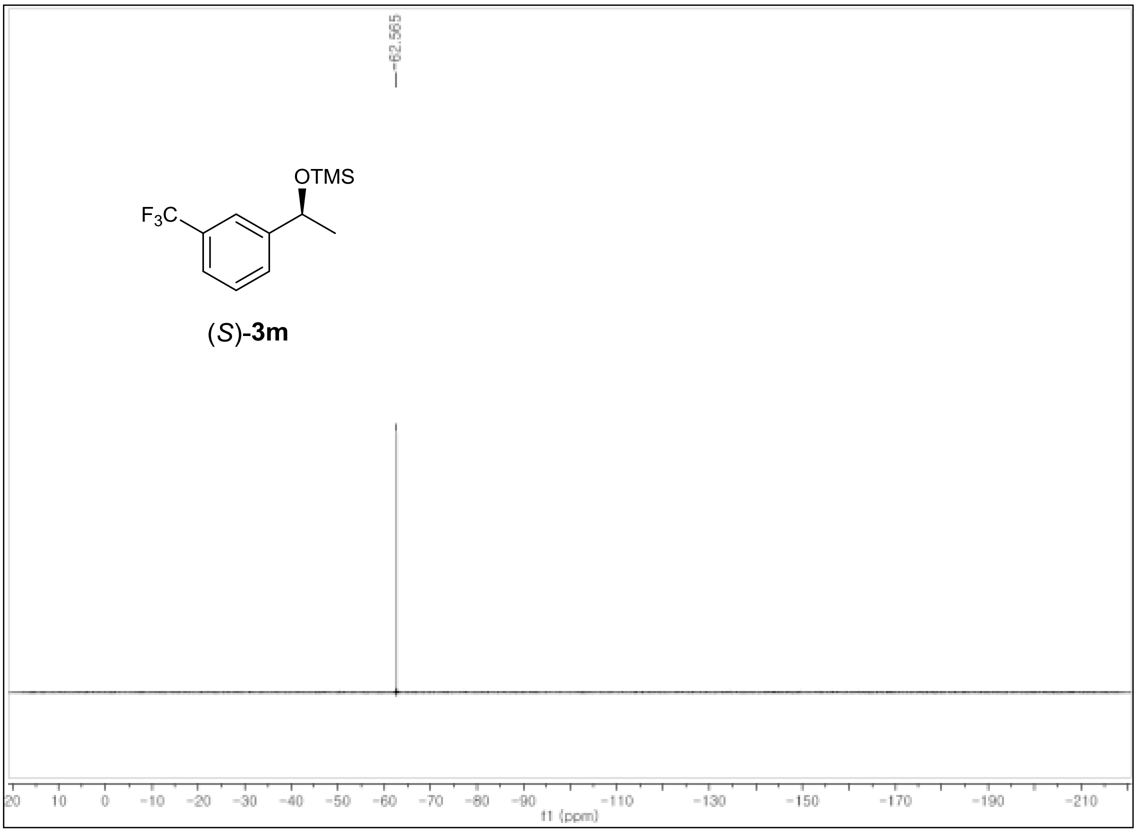

-62.565
OTMS
F3C
(S)-3m
20 10 0 -10 -20 -30 -40 -50 -60 -70 -80 -90 -110 -130 -150 -170 -190 -210
f1 (ppm)

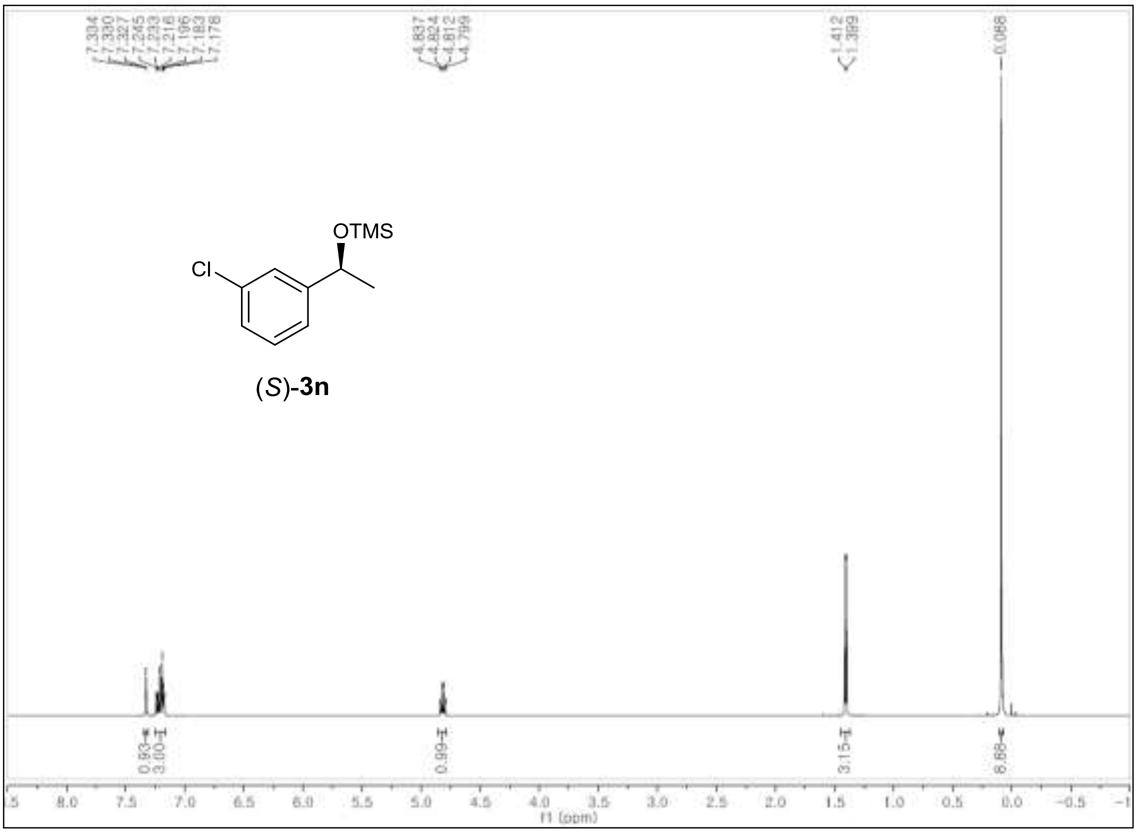

7.334
7.330
7.327
7.245
7.233
7.216
7.196
7.183
7.178
4.837
4.824
4.812
4.799
1.412
1.399
0.088
OTMS
Cl
(S)-3n
0.93
3.00
0.99
3.15
8.88
8.0 7.5 7.0 6.5 6.0 5.5 5.0 4.5 4.0 3.5 3.0 2.5 2.0 1.5 1.0 0.5 0.0 -0.5
f1 (ppm)

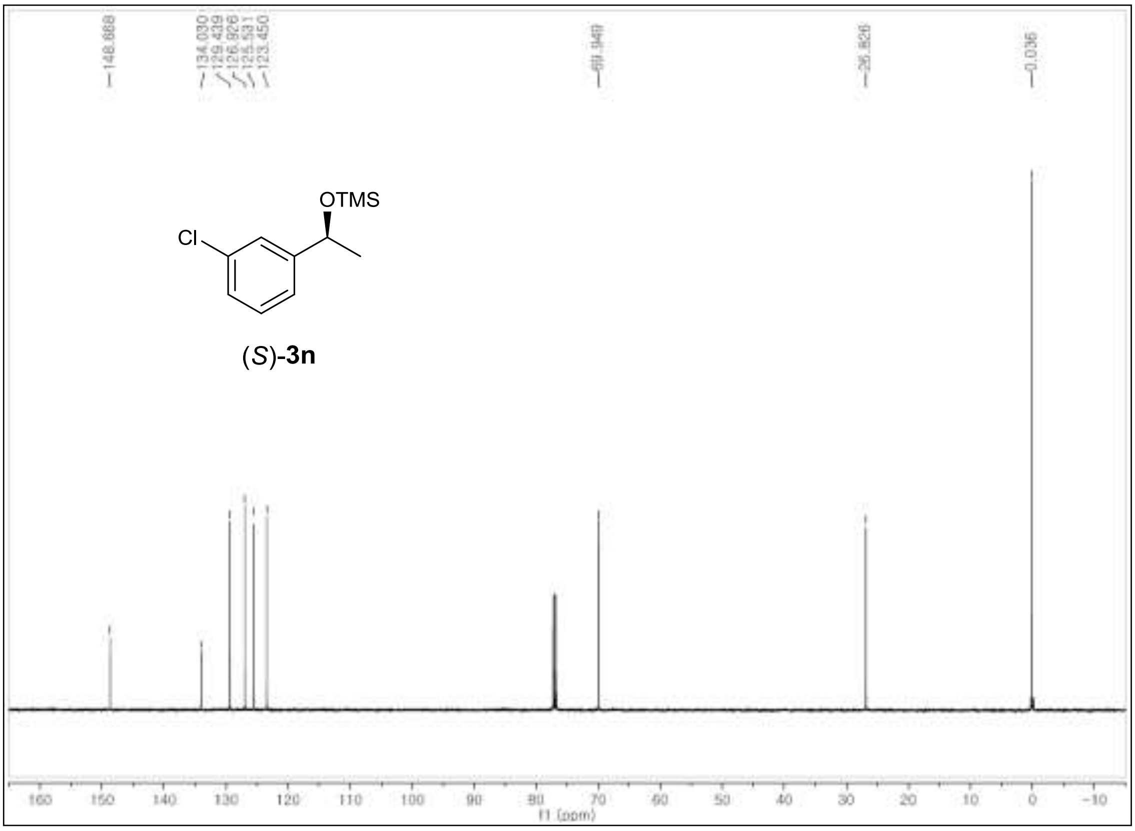

148.668
134.030
129.439
126.926
125.531
123.450
69.949
26.926
0.036
OTMS
Cl
(S)-3n
f1 (ppm)

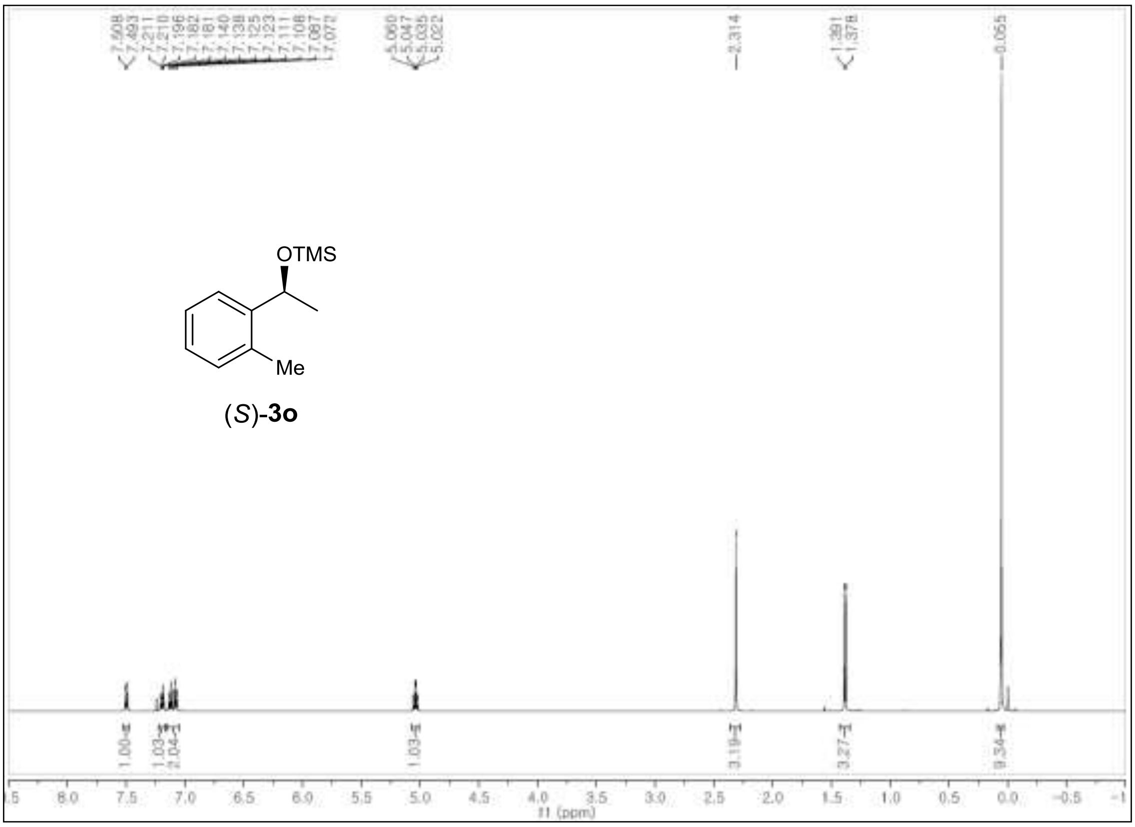

7.508
7.493
7.211
7.210
7.196
7.182
7.181
7.140
7.138
7.125
7.123
7.111
7.108
7.087
7.072
5.060
5.047
5.035
5.022
2.314
1.391
1.378
0.065
OTMS
Me
(S)-3o
1.00
1.03
2.04
1.03
3.19
3.27
9.34
f1 (ppm)

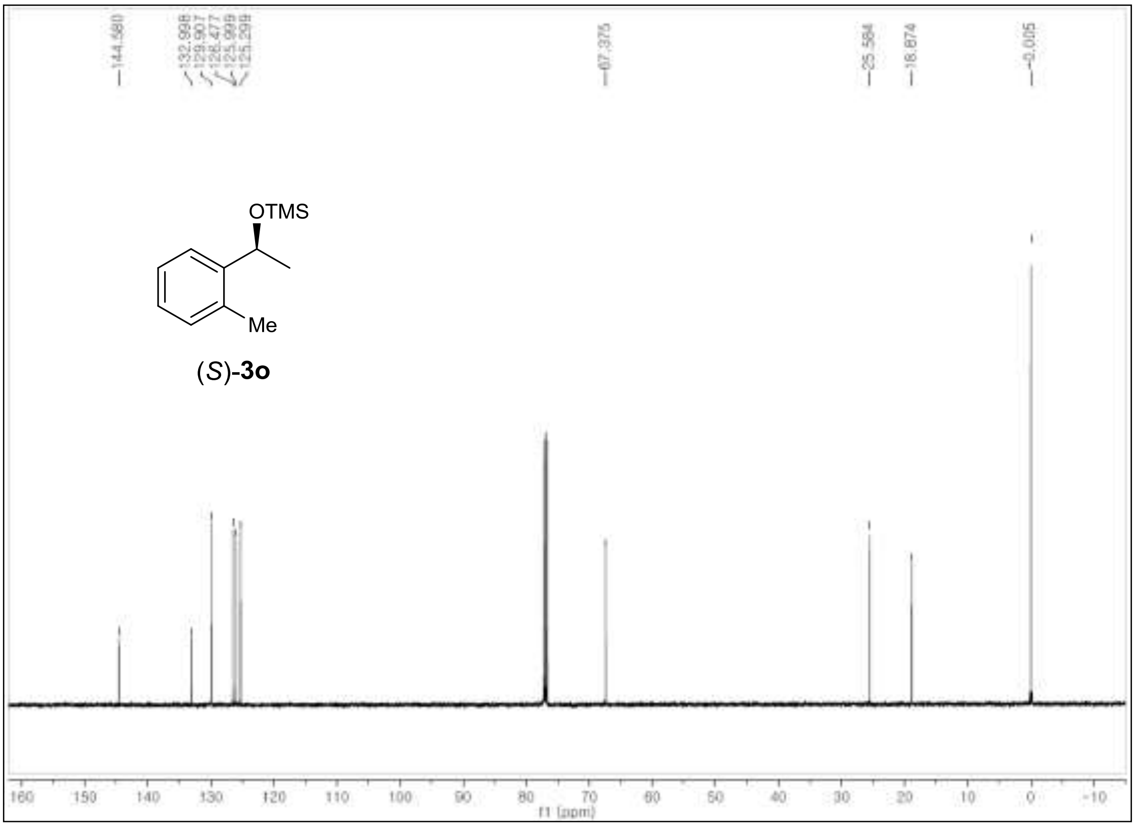
144.580
132.998
129.907
126.477
125.999
125.299
67.375
25.584
18.874
0.005
OTMS
Me
(S)-3o
f1 (ppm)

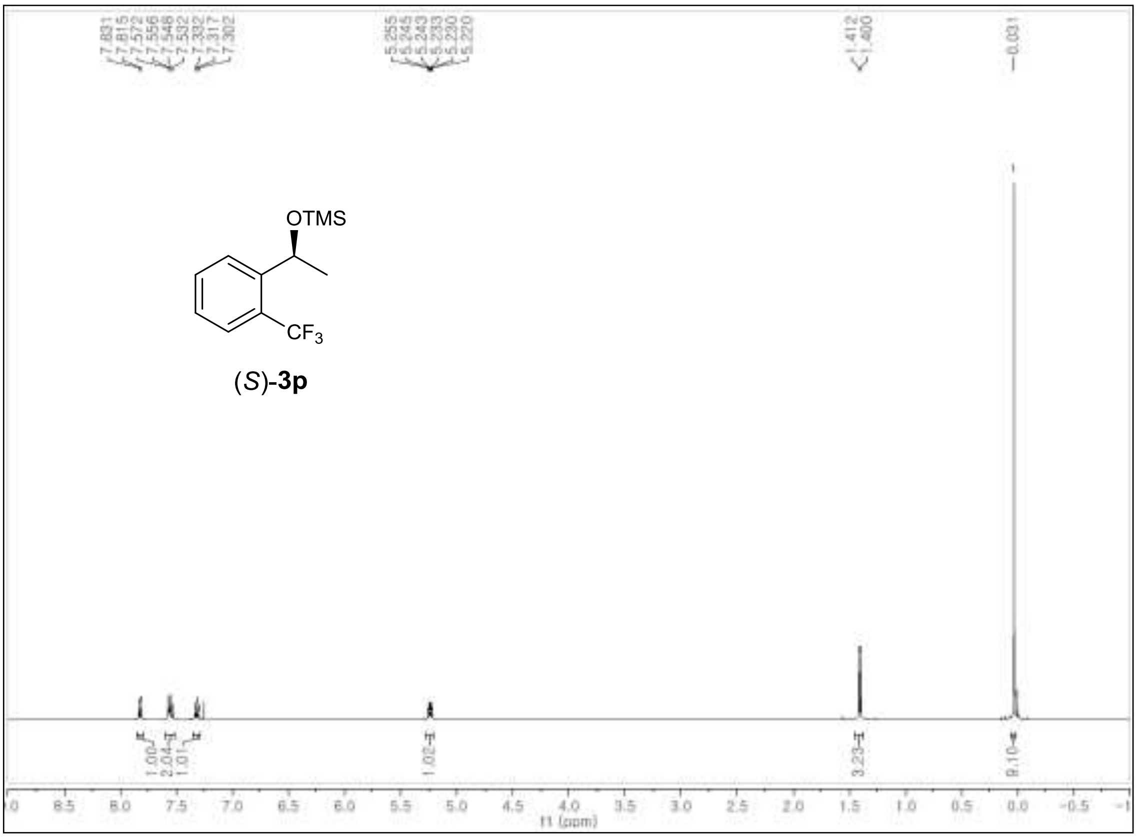
7.831
7.815
7.572
7.556
7.548
7.532
7.332
7.317
7.302
5.255
5.245
5.243
5.233
5.230
5.220
1.412
1.400
0.031
OTMS
CF3
(S)-3p
1.00
2.04
1.01
1.02
3.23
9.10
f1 (ppm)

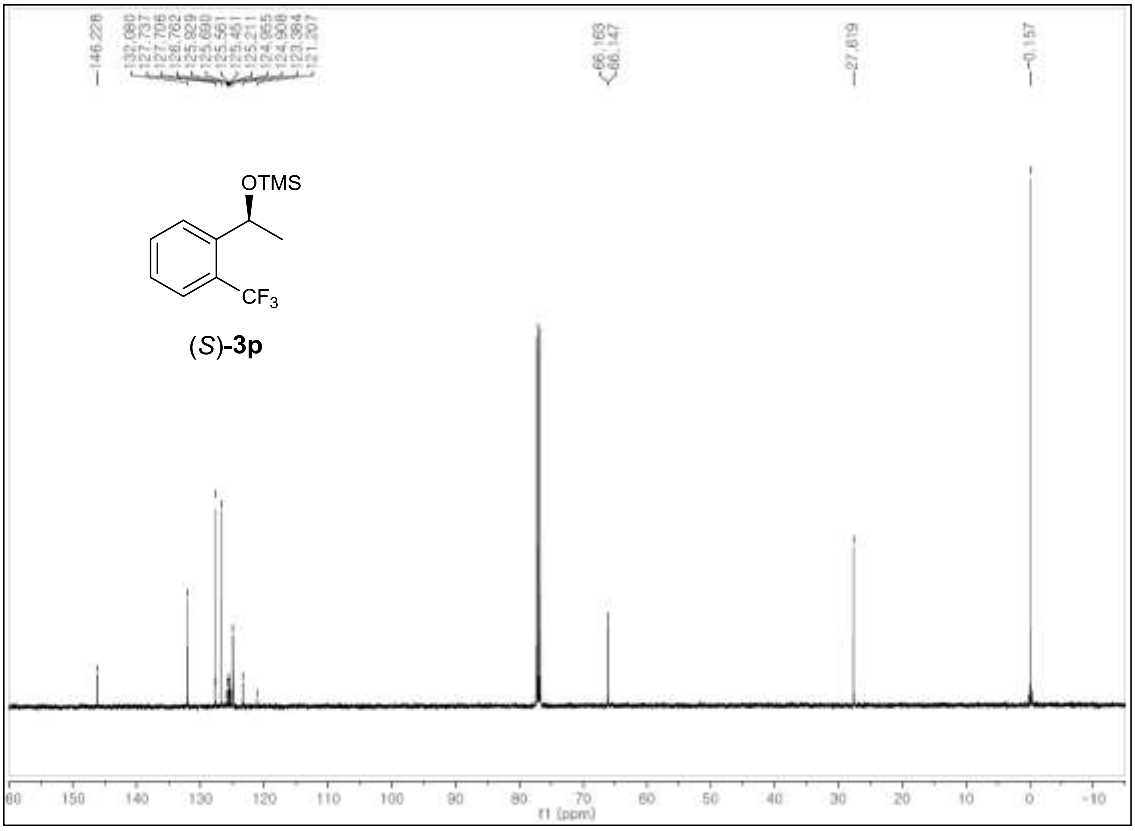
146.228
66.163
66.147
27.619
0.157
OTMS
CF3
(S)-3p
f1 (ppm)

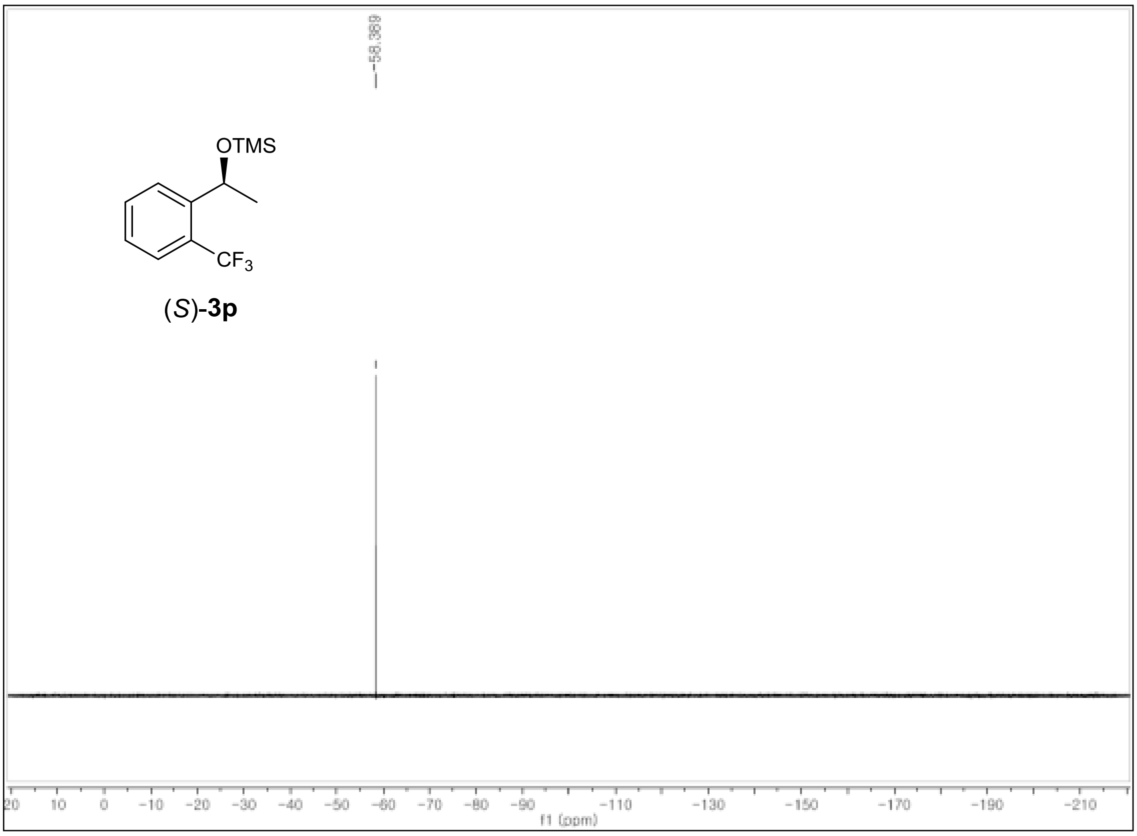
-58.389
OTMS
CF3
(S)-3p
f1 (ppm)

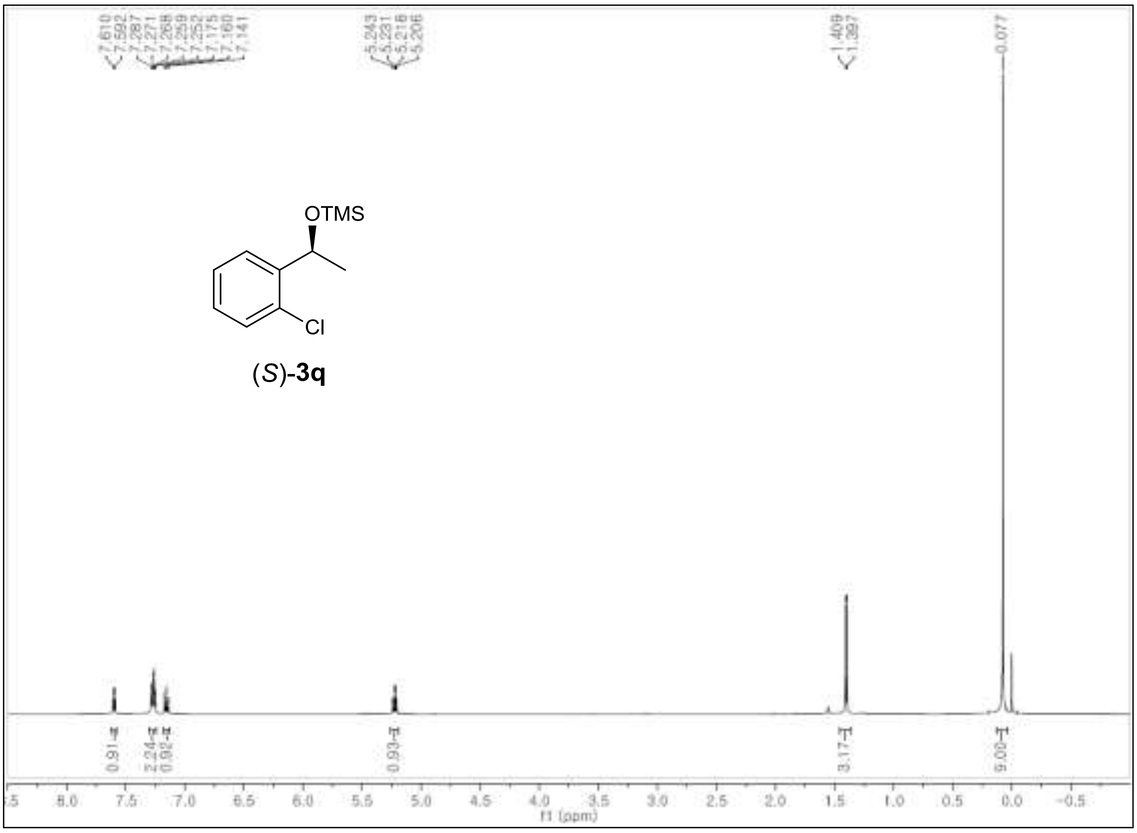

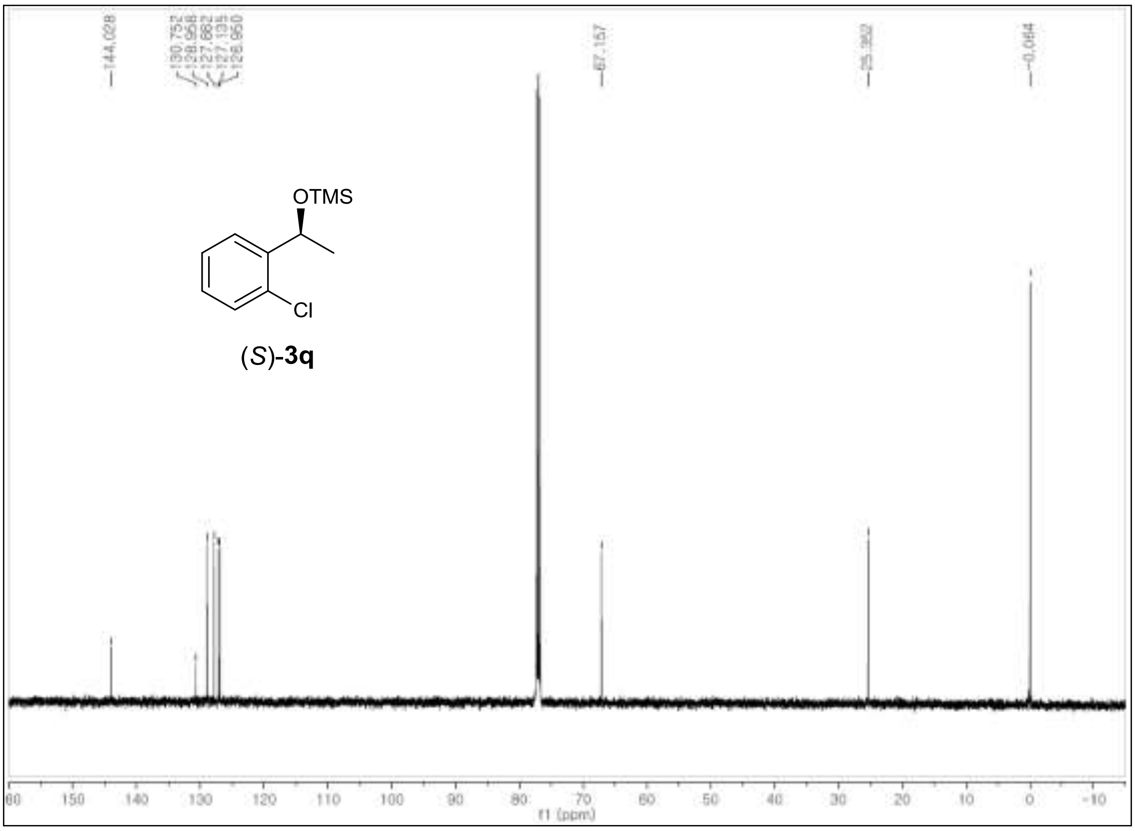

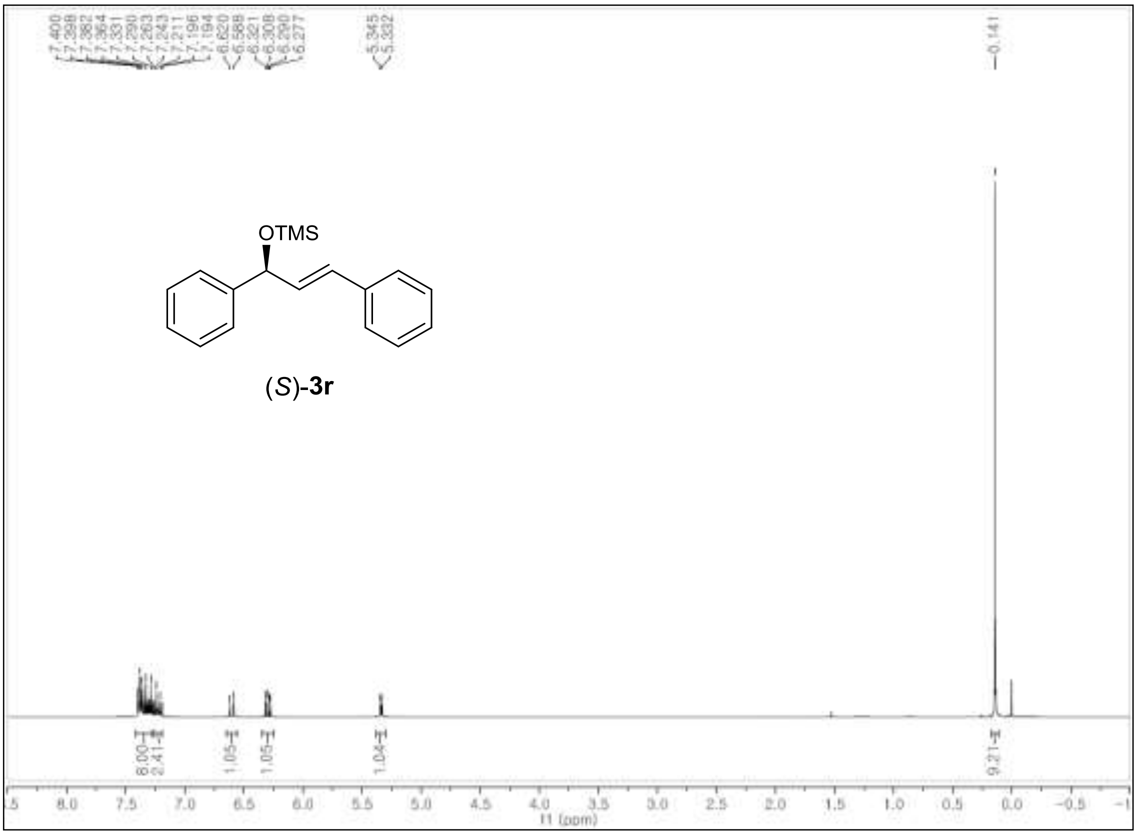
7.400
7.398
7.382
7.364
7.331
7.290
7.263
7.243
7.211
7.196
7.194
6.620
6.588
6.321
6.308
6.290
6.277
5.345
5.332
0.141
OTMS
(S)-3r
8.00
2.41
1.05
1.05
1.04
9.21
f1 (ppm)

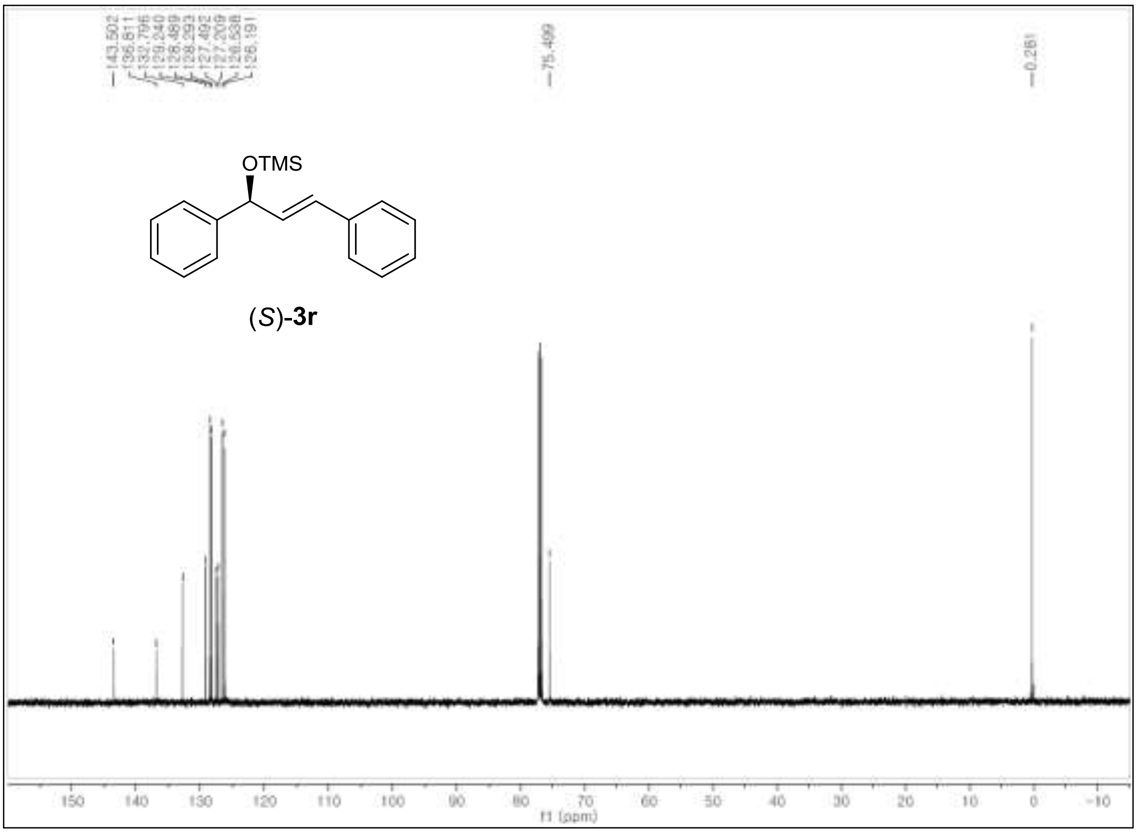
143.502
136.811
132.796
129.240
128.489
128.293
127.492
127.209
126.538
126.191
75.499
0.281
OTMS
(S)-3r
f1 (ppm)

Supplementary Figure 14. ^{1}H and ^{13}C NMR spectra of the *meso*-diols **4a–4c**

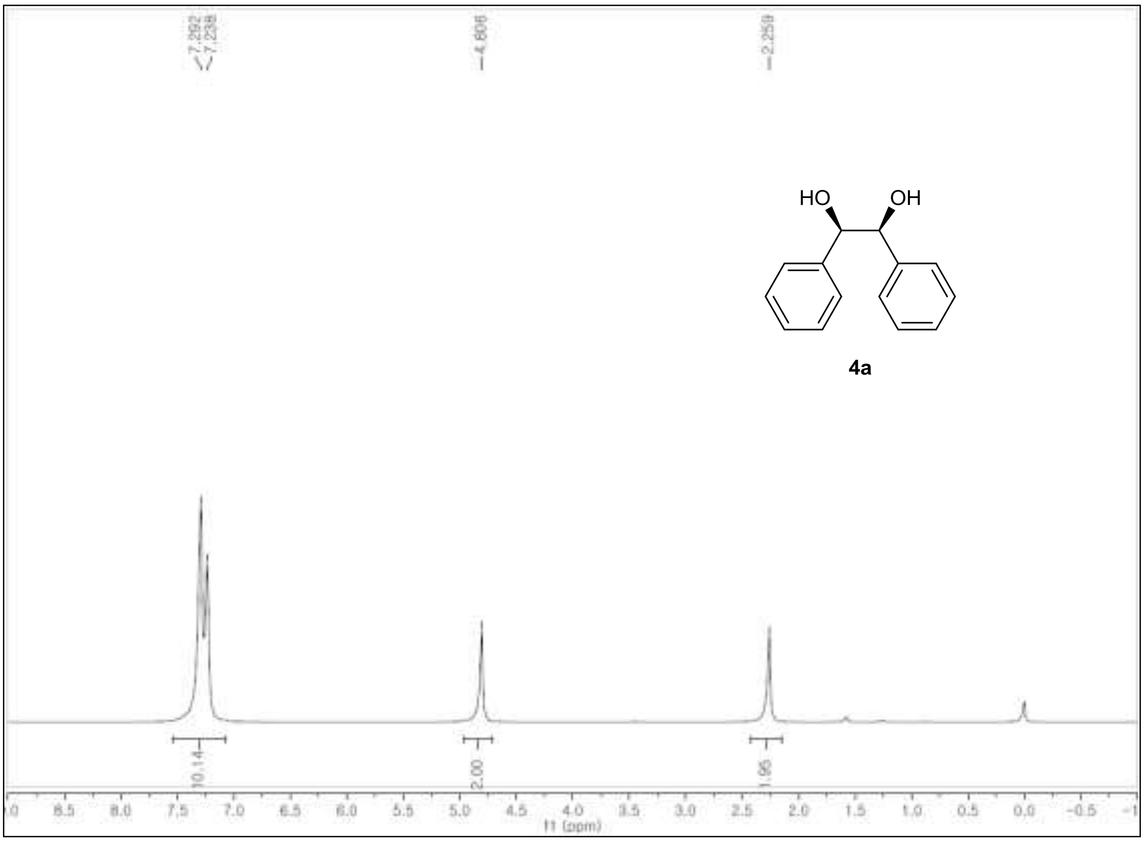

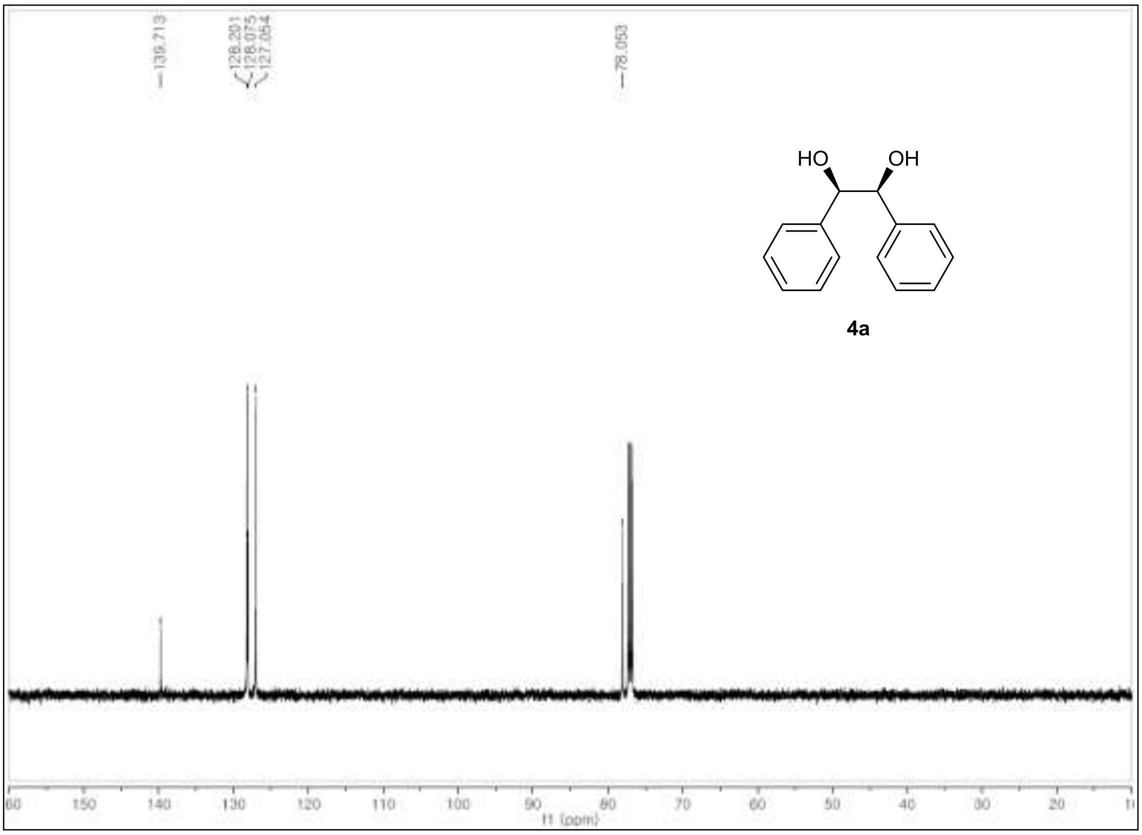

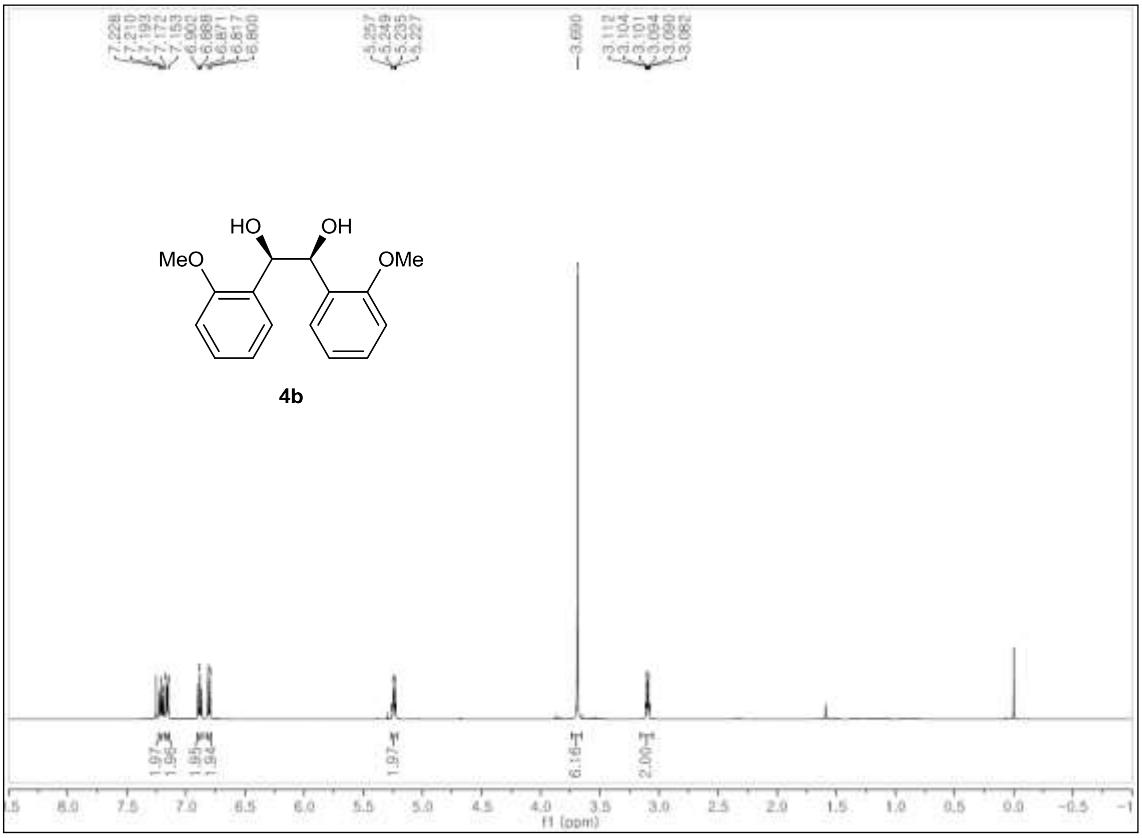
7.228
7.210
7.193
7.172
7.153
6.902
6.888
6.871
6.817
6.800
5.257
5.249
5.235
5.227
3.690
3.112
3.104
3.101
3.094
3.090
3.082
HO
OH
MeO
OMe
4b
1.97
1.96
1.95
1.94
1.97
6.16
2.00
f1 (ppm)

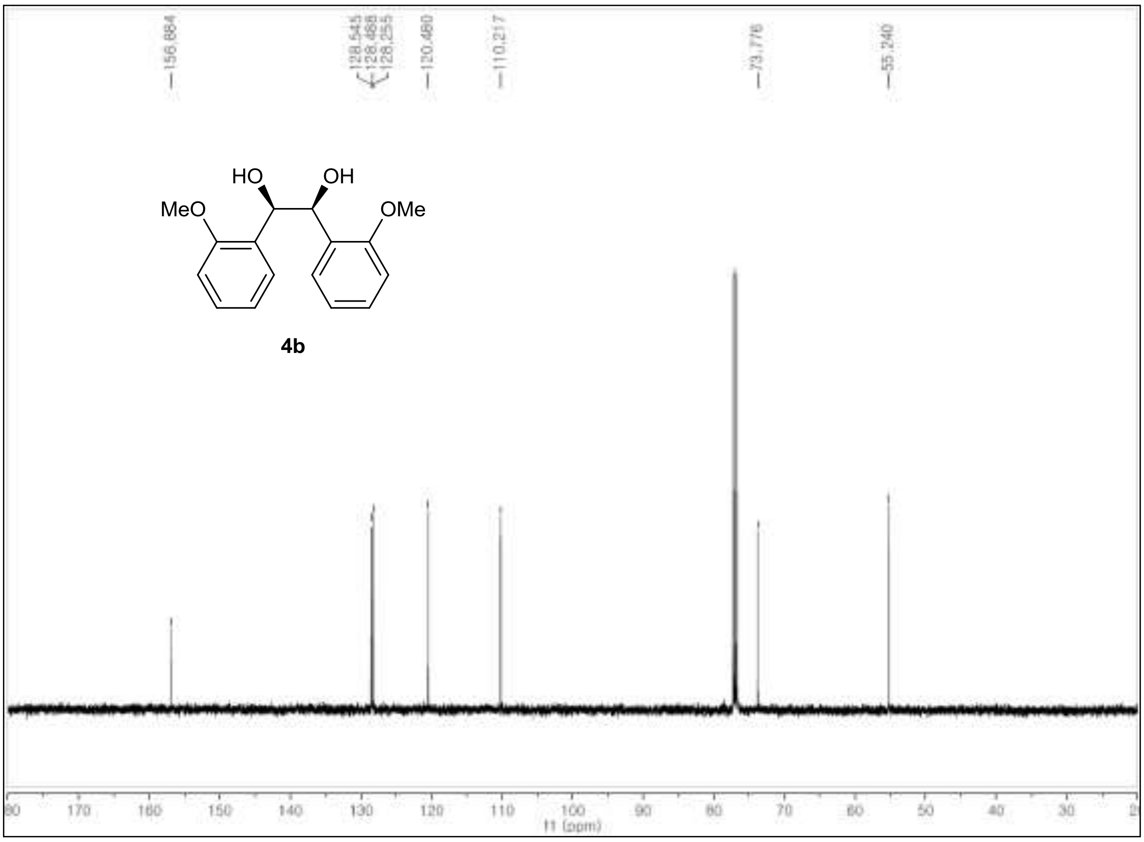
156.884
128.545
128.468
128.255
120.480
110.217
73.776
55.240
HO
OH
MeO
OMe
4b
f1 (ppm)

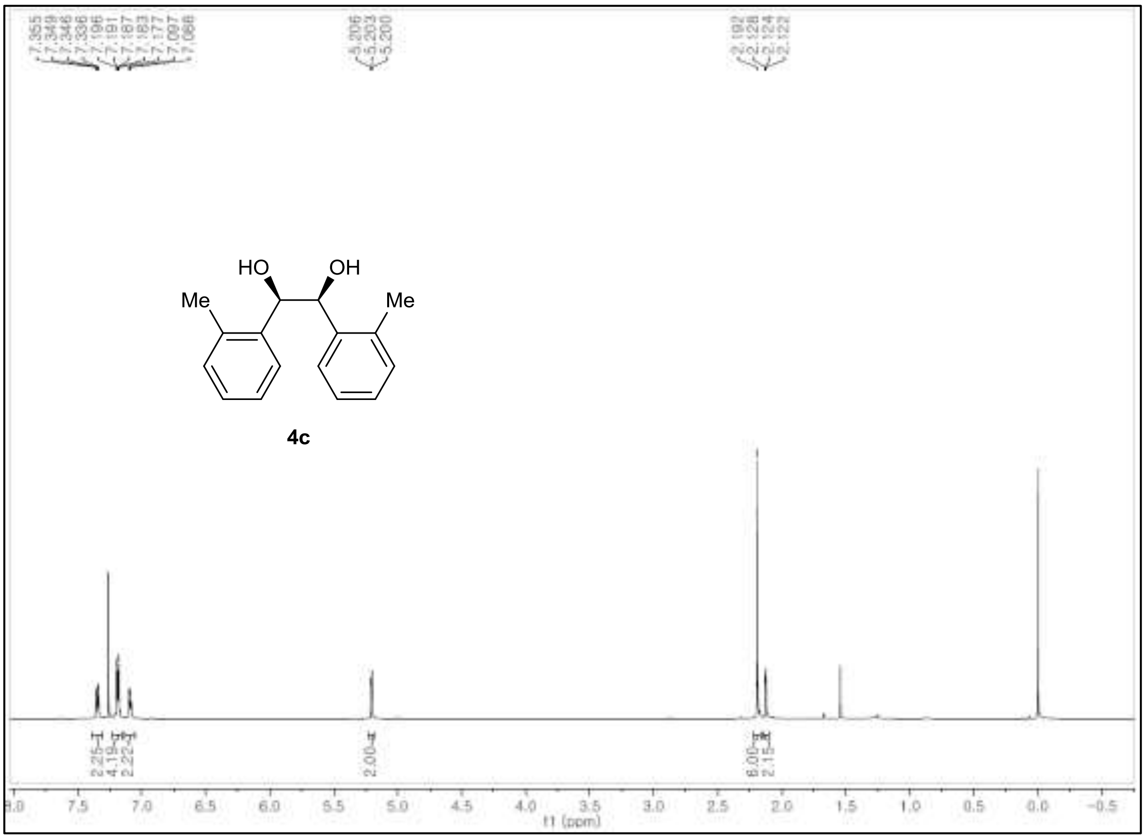
7.355
7.349
7.346
7.336
7.196
7.191
7.187
7.183
7.177
7.097
7.088
5.206
5.203
5.200
2.192
2.128
2.124
2.122
HO
OH
Me
Me
4c
2.25
4.19
2.22
2.00
6.00
2.15
f1 (ppm)

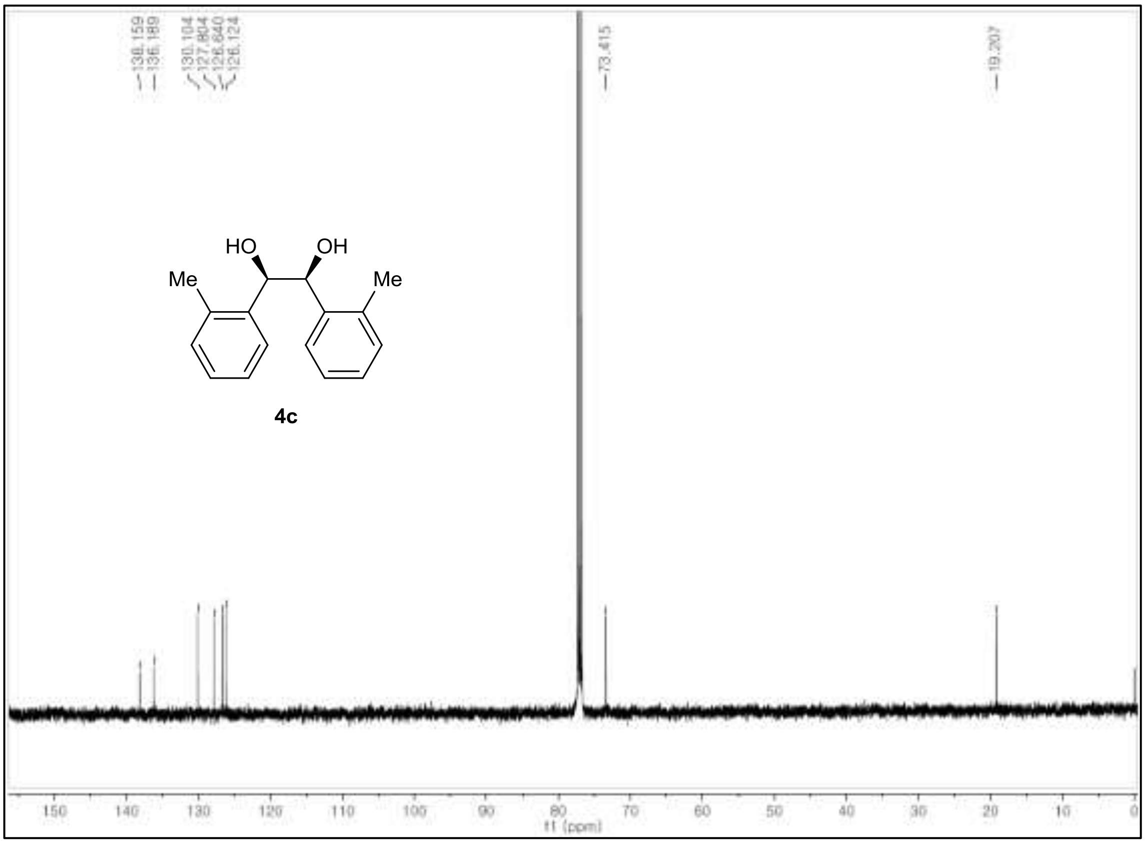
138.159
136.189
130.104
127.804
126.640
126.124
73.415
19.207
HO
OH
Me
Me
4c
f1 (ppm)

Supplementary Figure 15. ^{1}H and ^{13}C NMR spectra of the TMS-ether products **5a–5c**

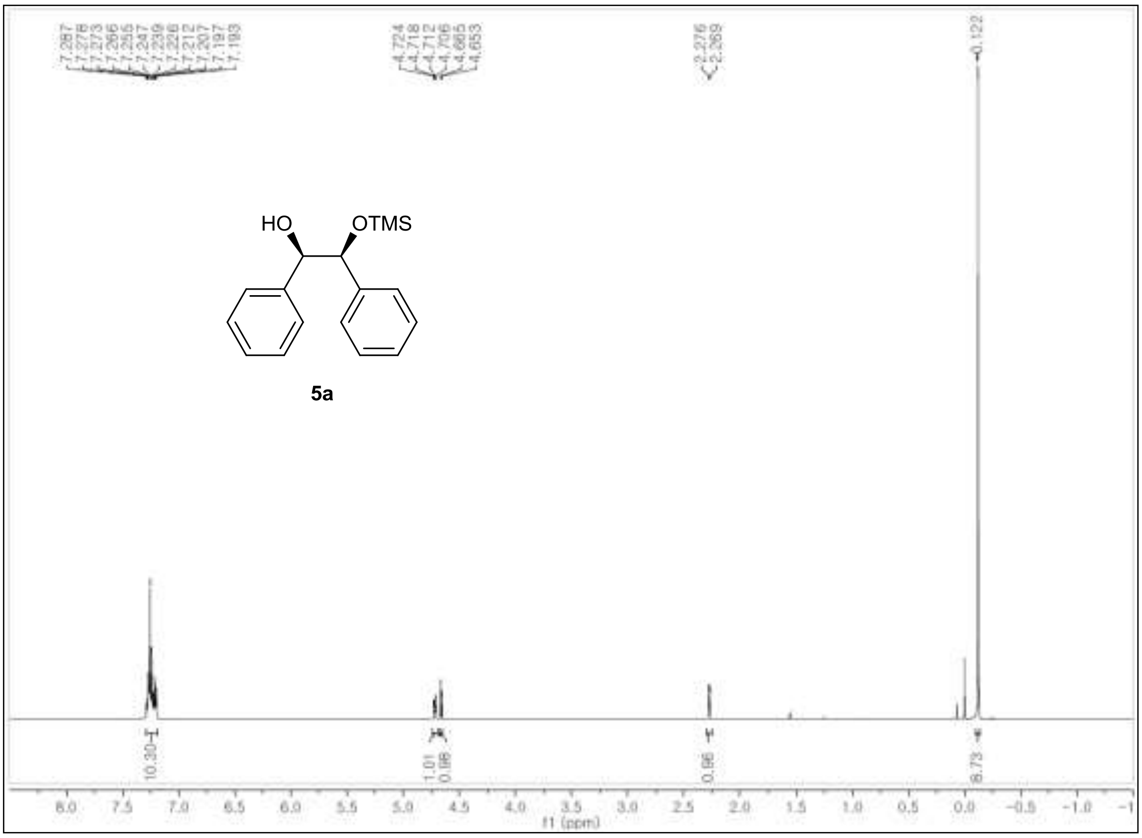

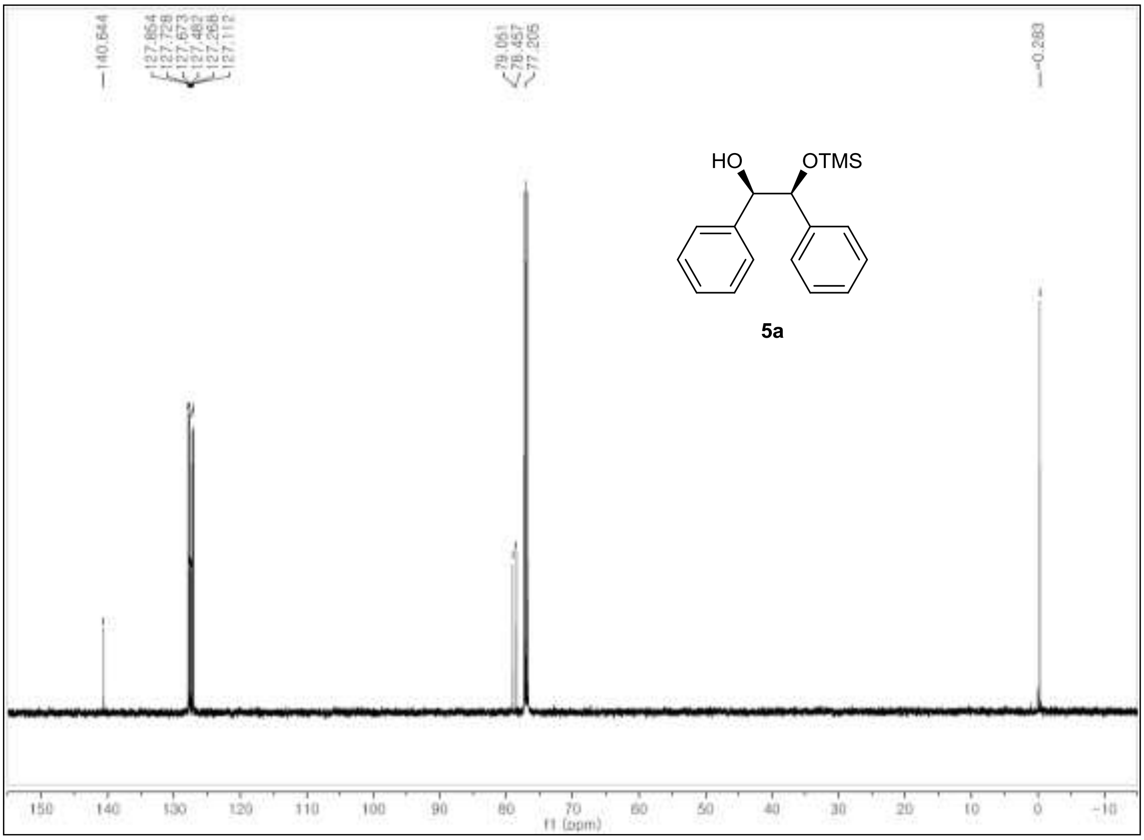

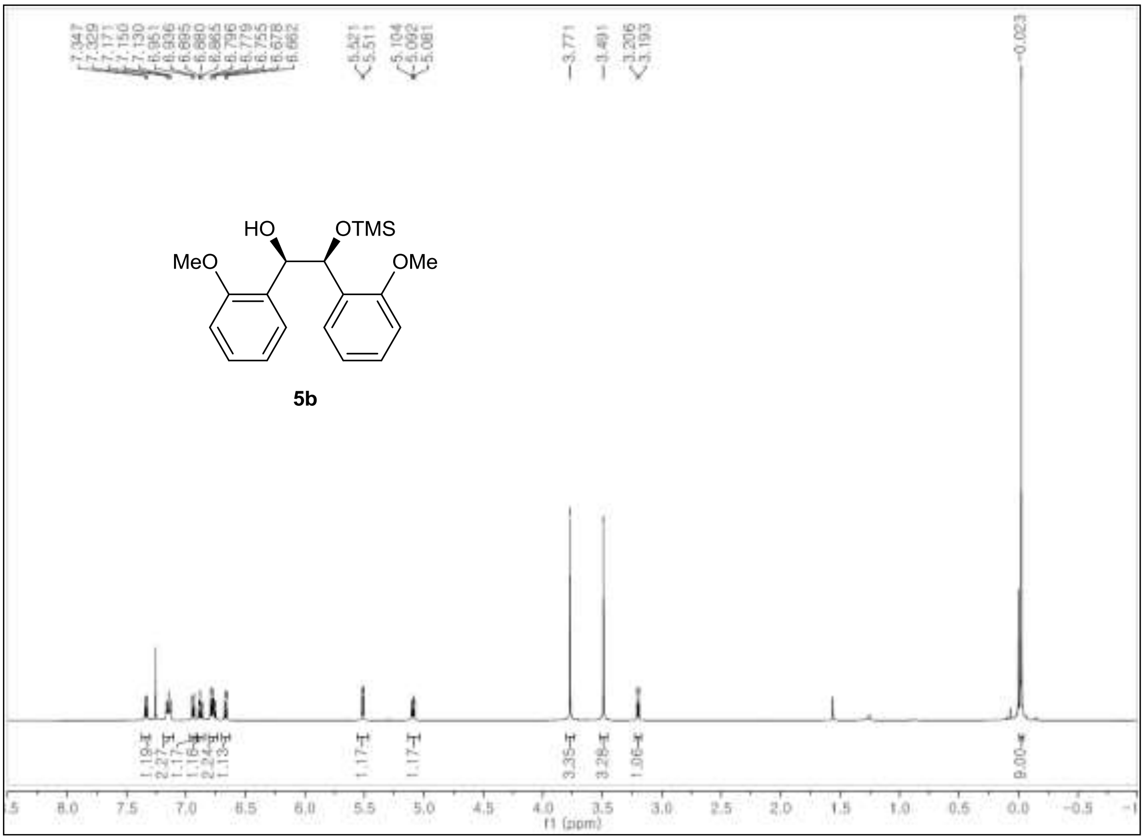

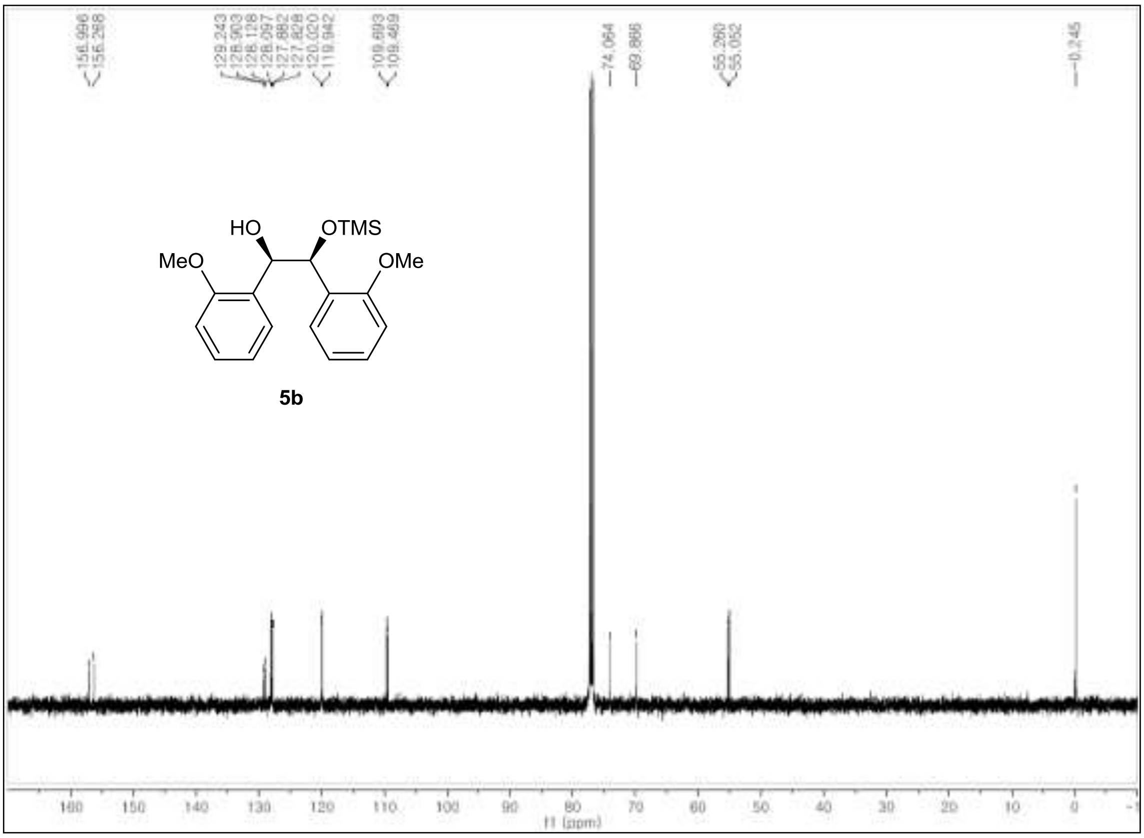

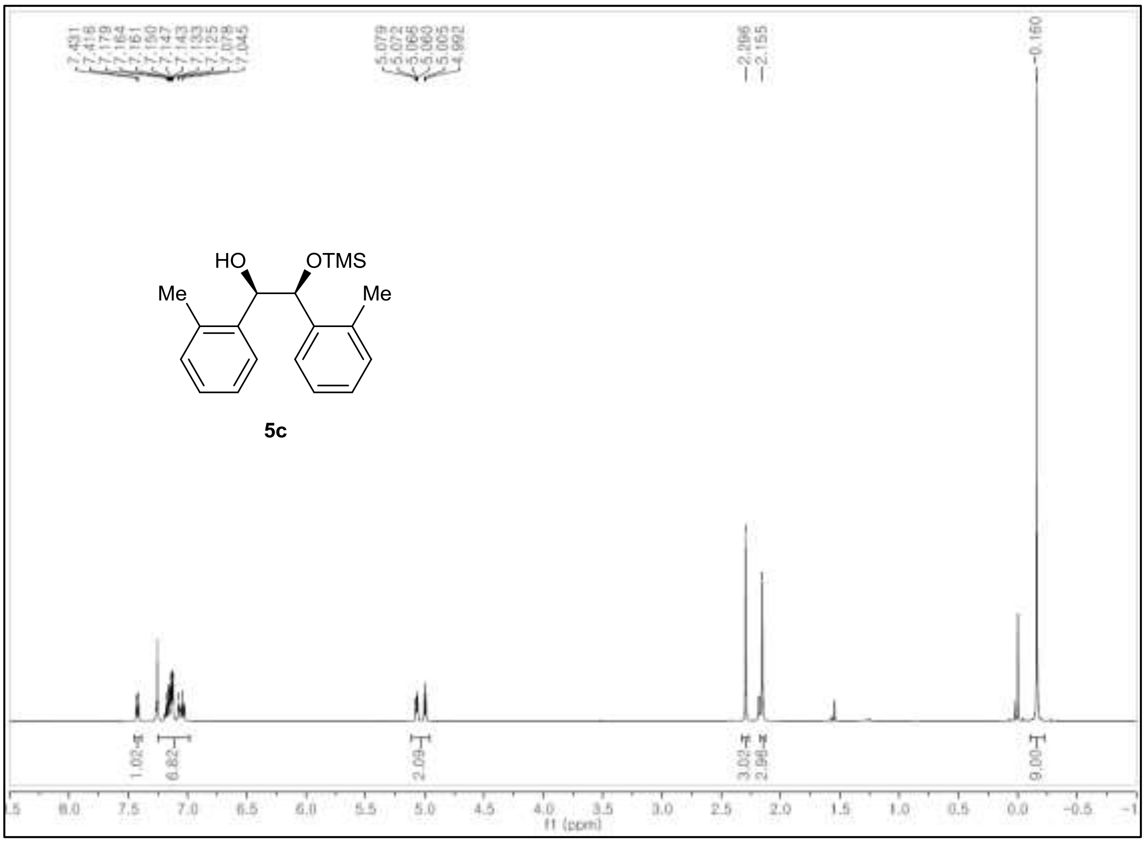
7.431
7.416
7.179
7.164
7.161
7.150
7.147
7.143
7.133
7.125
7.078
7.045
5.079
5.072
5.066
5.060
5.005
4.992
2.296
2.155
-0.160
HO
OTMS
Me
Me
5c
1.02
6.82
2.09
3.02
2.98
9.00
f1 (ppm)

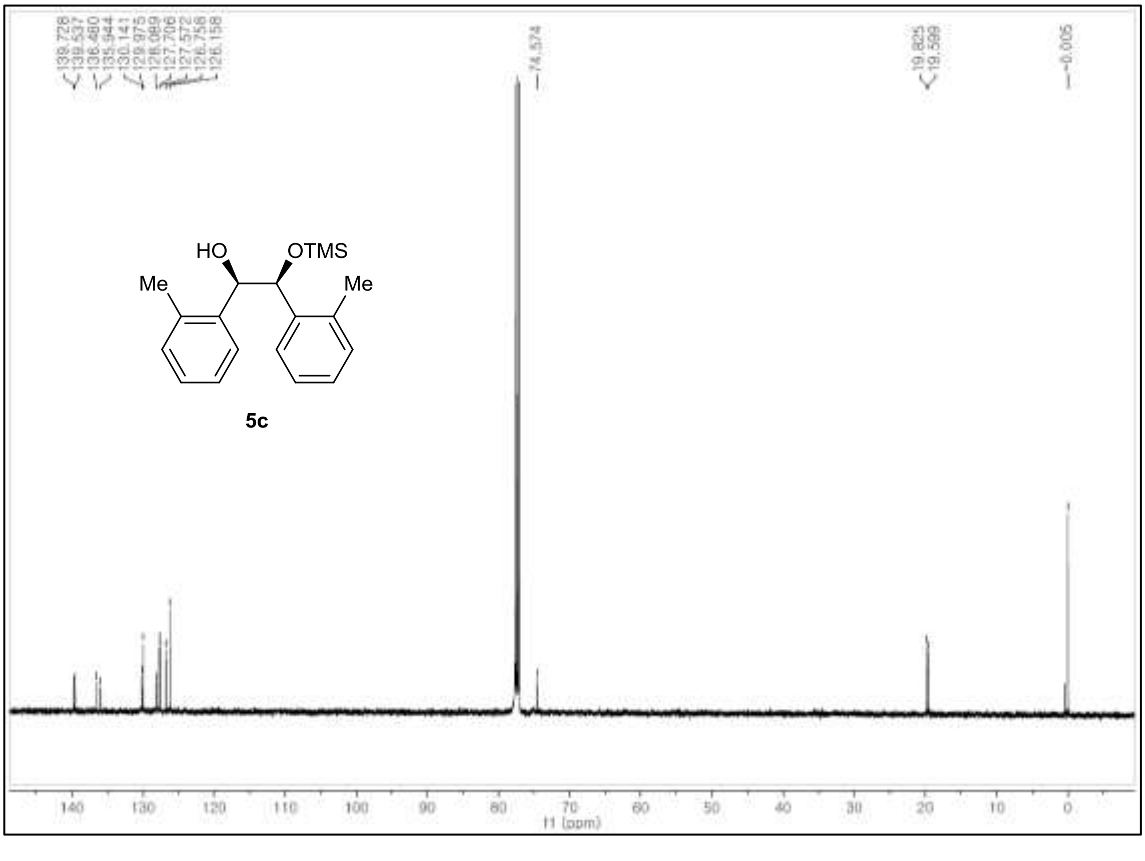
139.728
139.537
136.480
135.944
130.141
129.975
128.089
127.706
127.572
126.756
126.158
74.574
19.825
19.599
-0.005
HO
OTMS
Me
Me
5c
f1 (ppm)

Supplementary Figure 16. ^{1}H NMR and HPLC Spectra for Figure **3**

We monitored the conversion by ^{1}H-NMR spectrum and calculated by the following equation ($c = ee_{rsm}/(ee_{prod}+ee_{rsm})$)[1] in Figure **3** – Figure **5a**.

^{1}H NMR spectrum of the silylation reaction mixture of 2a after 7% conversion: cat 1c

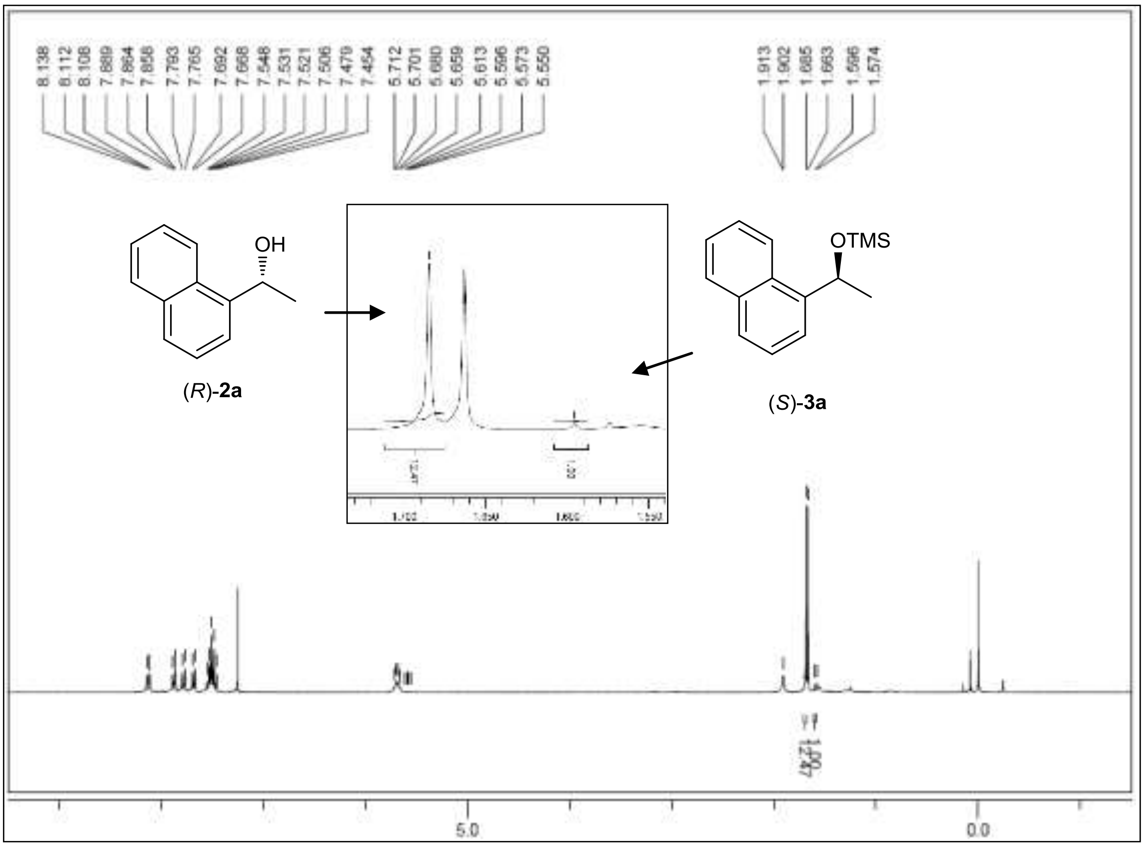

^{1}H NMR spectrum of the silylation reaction mixture of 1a after 11% conversion: cat 1d

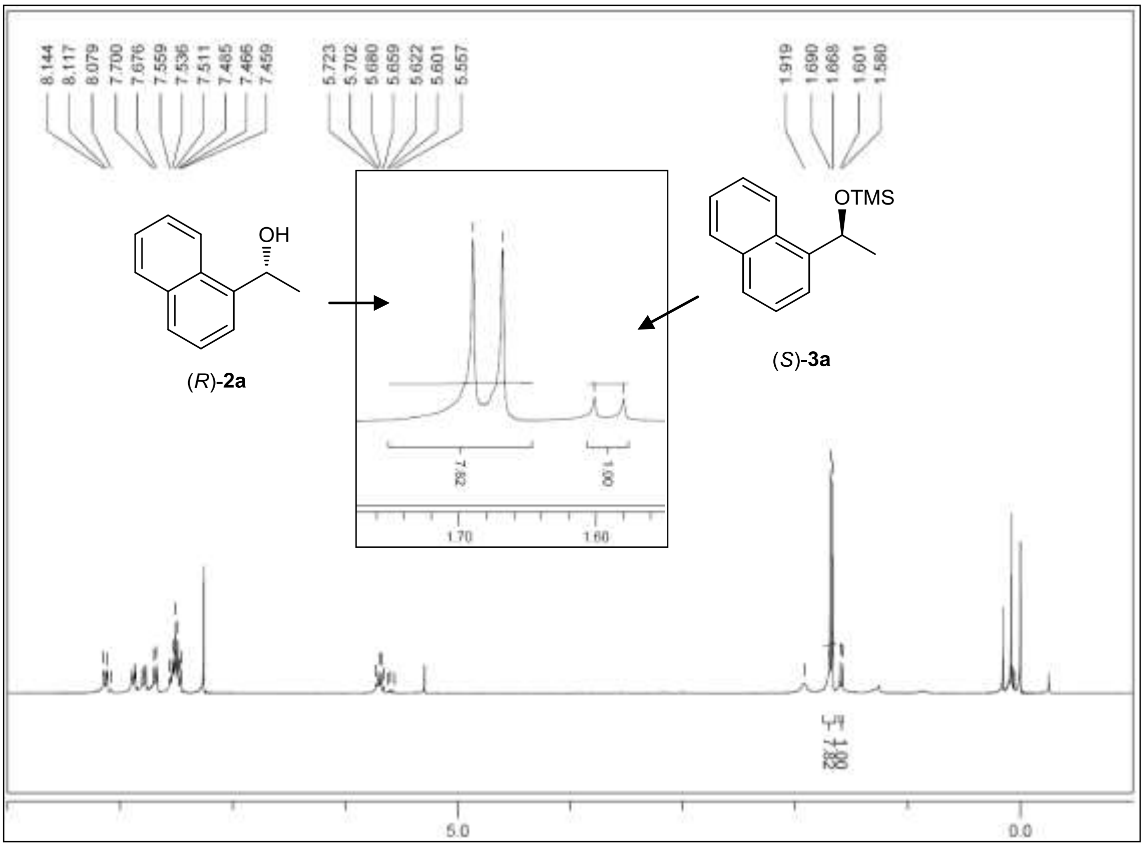

HPLC spectrum of the *rac*-**2a** (Chiralcel OD-H, Hexane/IPA = 90/10, 0.7 ml/min, 220 nm)

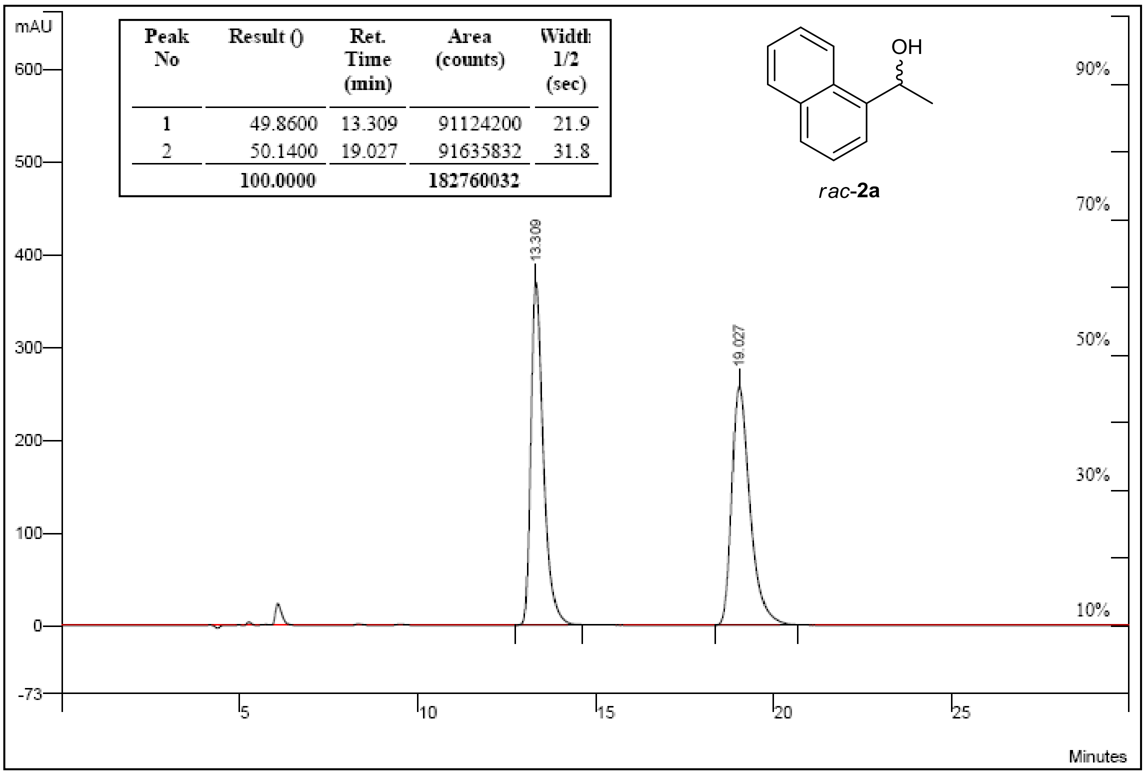

Peak No	Result ()	Ret. Time (min)	Area (counts)	Width 1/2 (sec)
1	49.8600	13.309	91124200	21.9
2	50.1400	19.027	91635832	31.8
	**100.0000**		**182760032**	

HPLC spectrum of the TMS-ether product (*S*)-**3a**

[Chiralcel OD-H, Hexane/IPA = 90/10, 0.7 ml/min, 220 nm]

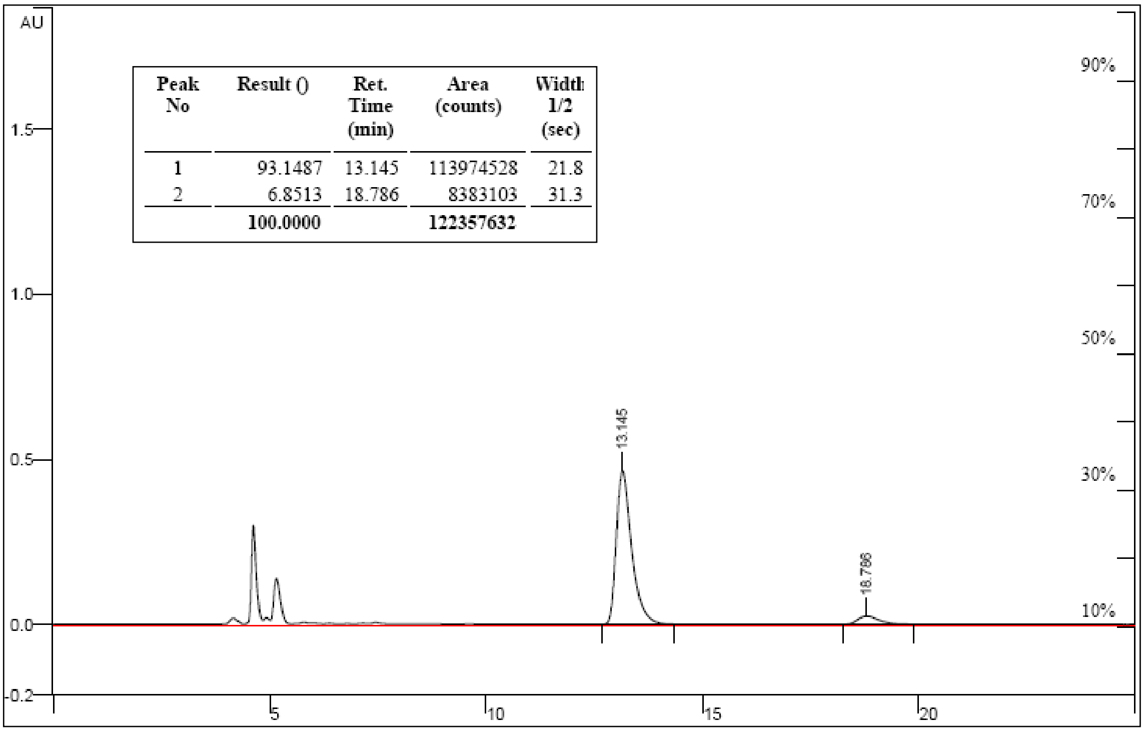

Peak No	Result ()	Ret. Time (min)	Area (counts)	Width 1/2 (sec)
1	93.1487	13.145	113974528	21.8
2	6.8513	18.786	8383103	31.3
	100.0000		122357632	

HPLC spectrum of the remaining alcohol (*R*)-**2a**

[Chiralcel OD-H, Hexane/IPA = 90/10, 0.7 ml/min, 220 nm]

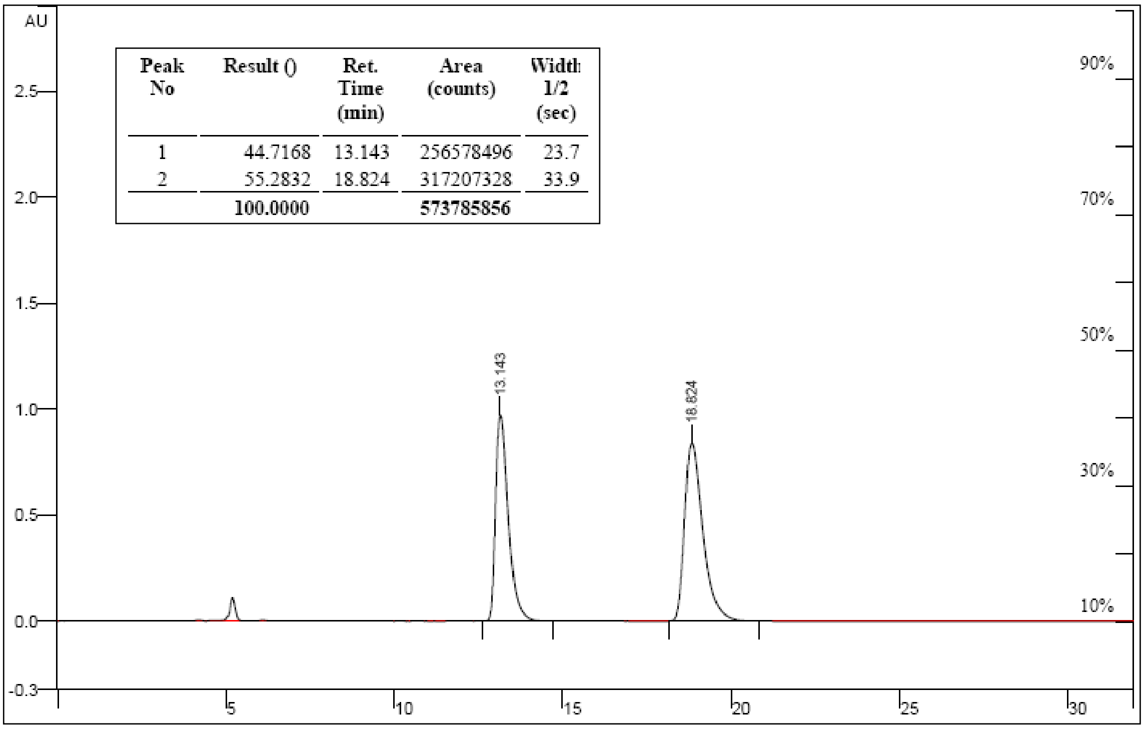

Peak No	Result ()	Ret. Time (min)	Area (counts)	Width 1/2 (sec)
1	44.7168	13.143	256578496	23.7
2	55.2832	18.824	317207328	33.9
	100.0000		573785856	

^{1}H NMR spectrum of the silylation reaction mixture of 2a after 49% conversion: cat 1e

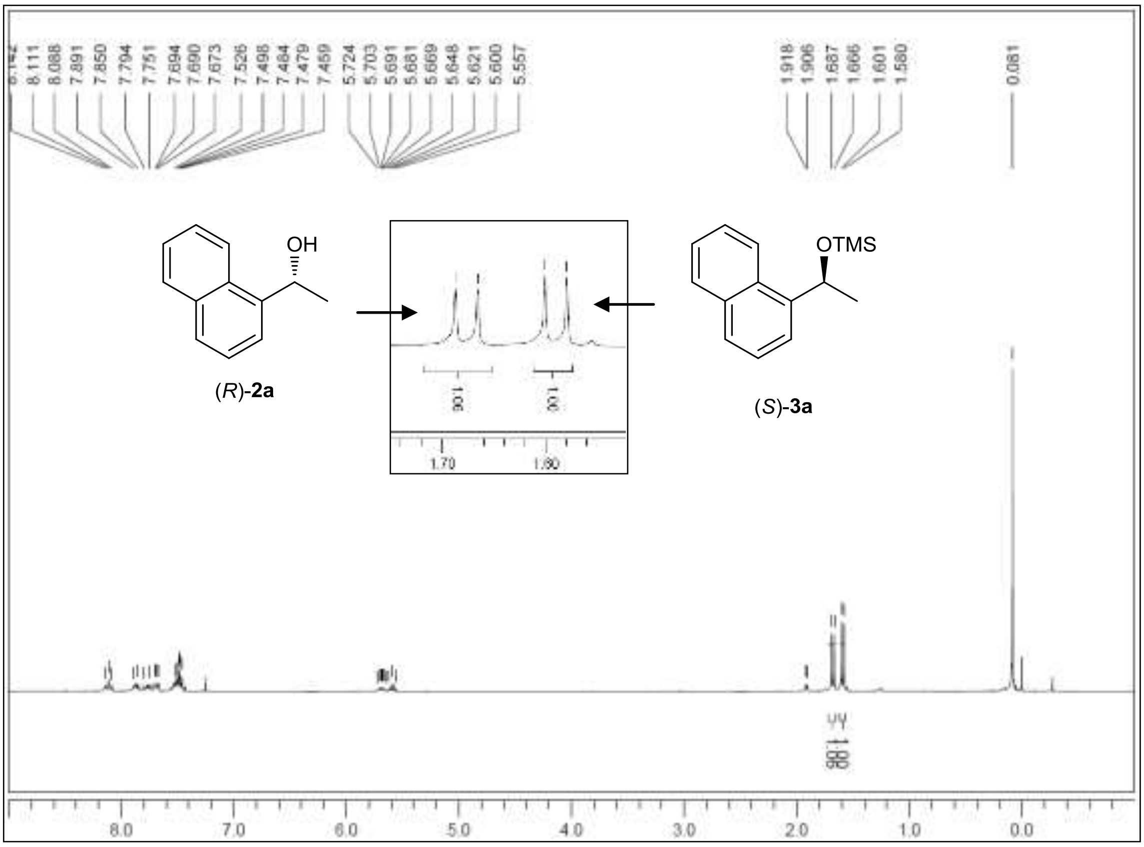

HPLC spectrum of the TMS-ether product (*S*)-**3a**

[Chiralcel OD-H, Hexane/IPA = 90/10, 0.7 ml/min, 220 nm]

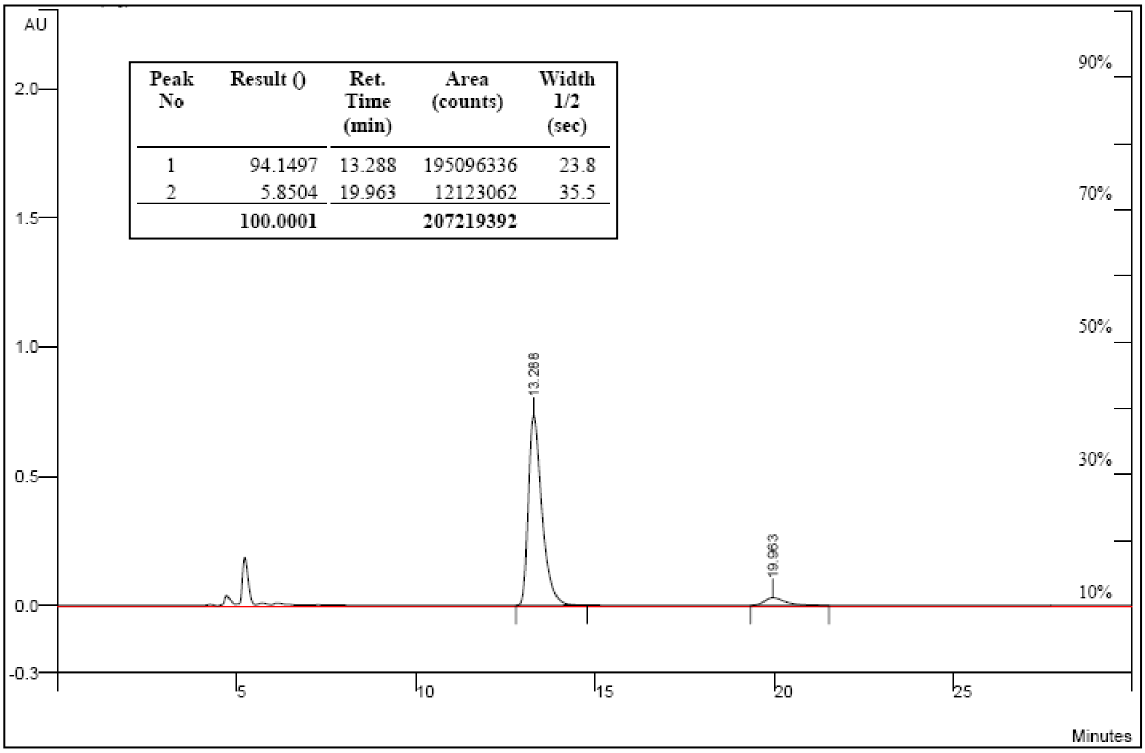

Peak No	Result ()	Ret. Time (min)	Area (counts)	Width 1/2 (sec)
1	94.1497	13.288	195096336	23.8
2	5.8504	19.963	12123062	35.5
	**100.0001**		**207219392**	

HPLC spectrum of the remaining alcohol (*R*)-**2a**

[Chiralcel OD-H, Hexane/IPA = 90/10, 0.7 ml/min, 220 nm]

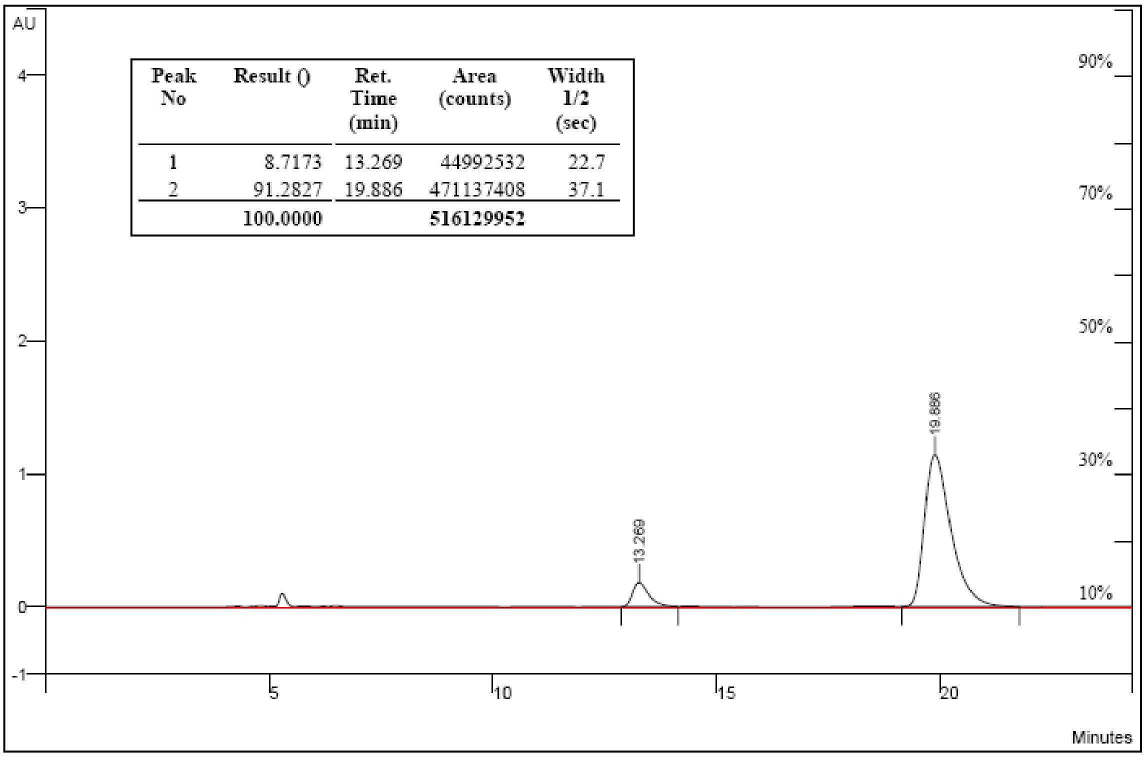

¹H NMR spectrum of the silylation reaction mixture of 2a after 44% conversion: cat 1f

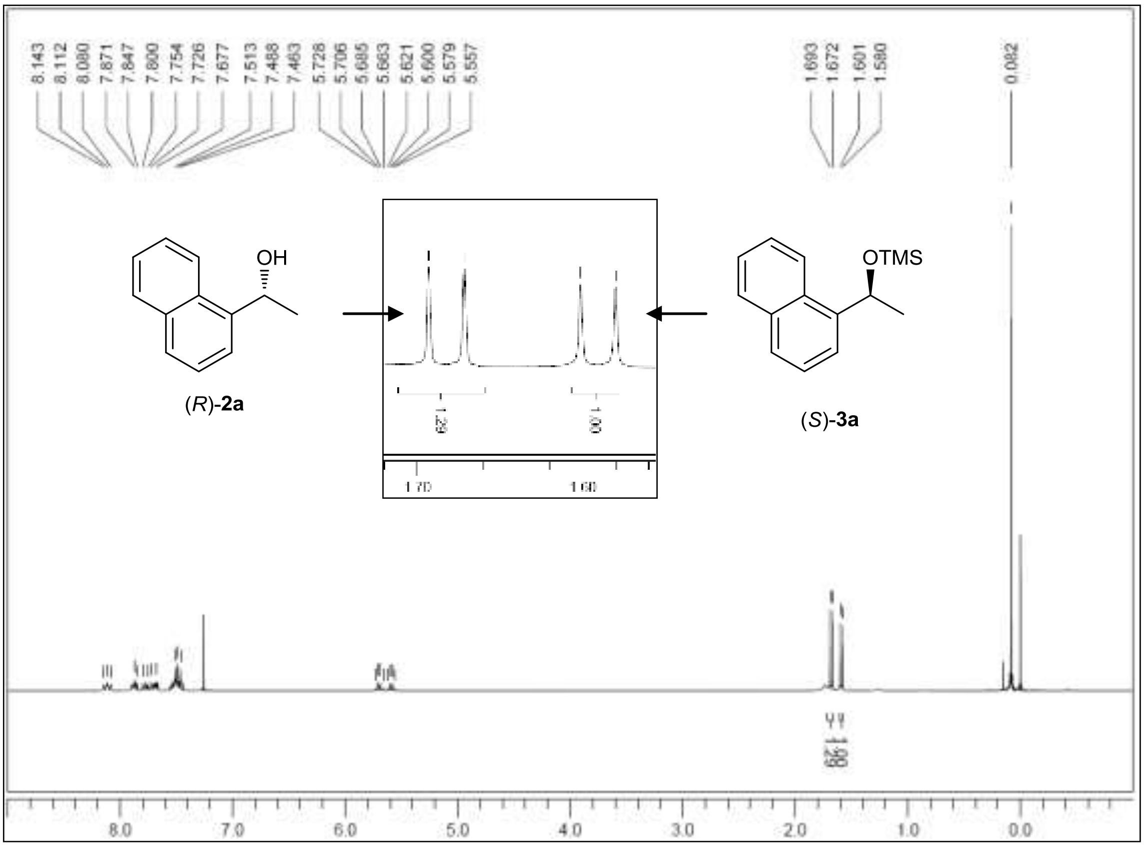

HPLC spectrum of the TMS-ether product (*S*)-**3a**

[Chiralcel OD-H, Hexane/IPA = 90/10, 0.7 ml/min, 220 nm]

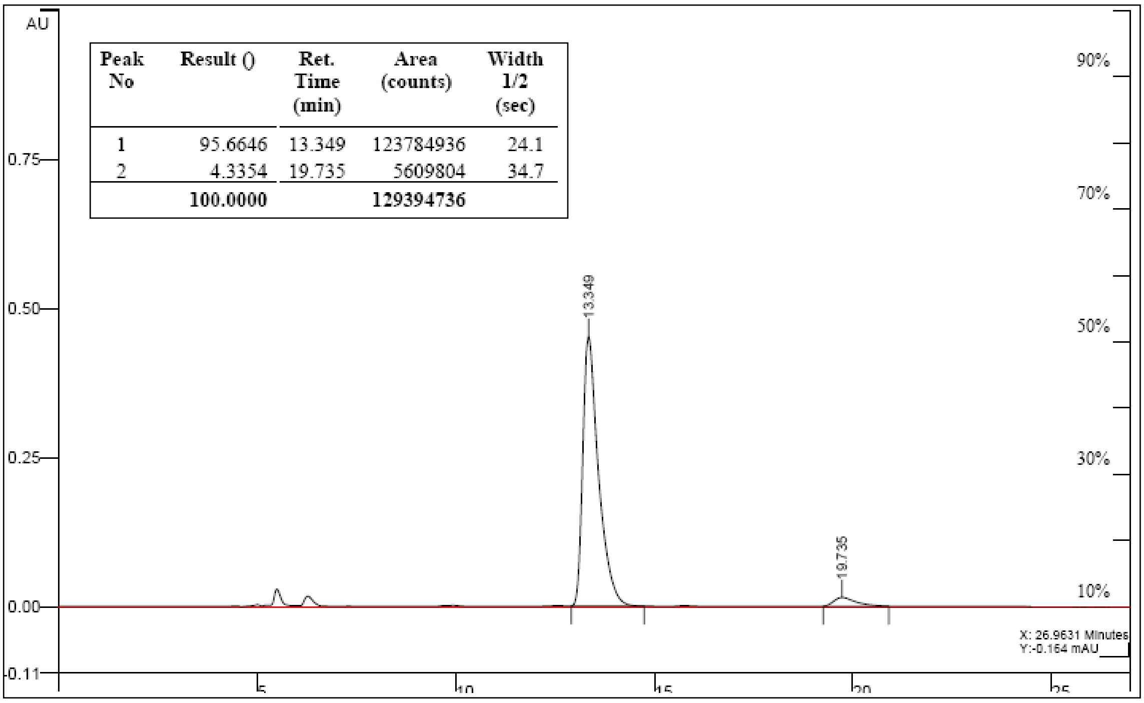

Peak No	Result ()	Ret. Time (min)	Area (counts)	Width 1/2 (sec)
1	95.6646	13.349	123784936	24.1
2	4.3354	19.735	5609804	34.7
	**100.0000**		**129394736**	

HPLC spectrum of the remaining alcohol (*R*)-**2a**

[Chiralcel OD-H, Hexane/IPA = 90/10, 0.7 ml/min, 220 nm]

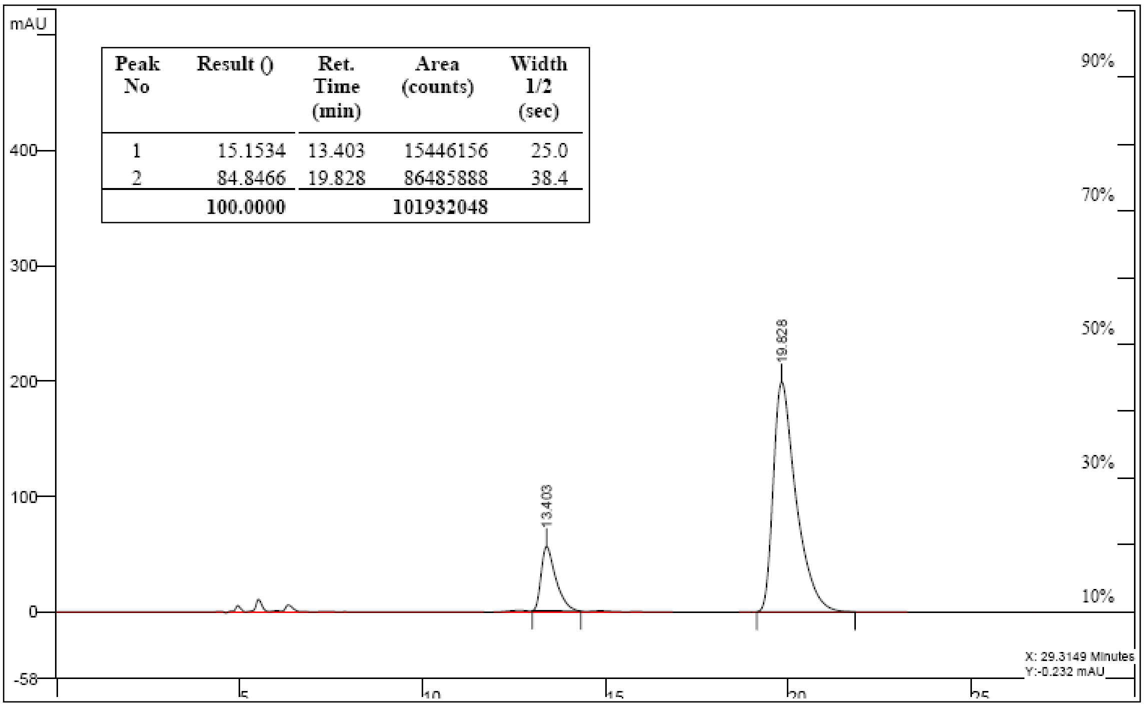

Peak No	Result ()	Ret. Time (min)	Area (counts)	Width 1/2 (sec)
1	15.1534	13.403	15446156	25.0
2	84.8466	19.828	86485888	38.4
	**100.0000**		**101932048**	

^{1}H NMR spectrum of the silylation reaction mixture of 2a after 8% conversion: cat 1g

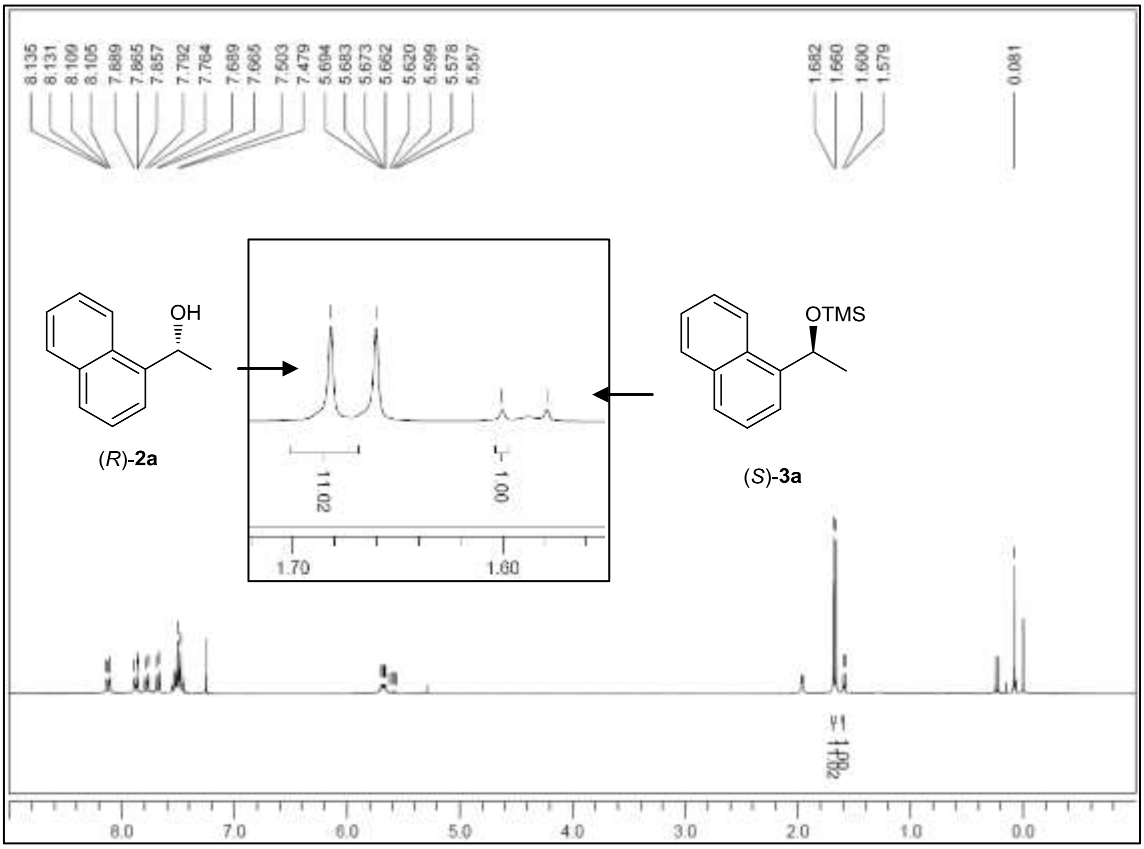

HPLC spectrum of the TMS-ether product (*S*)-**3a**

[Chiralcel OD-H, Hexane/IPA = 90/10, 0.7 ml/min, 220 nm]

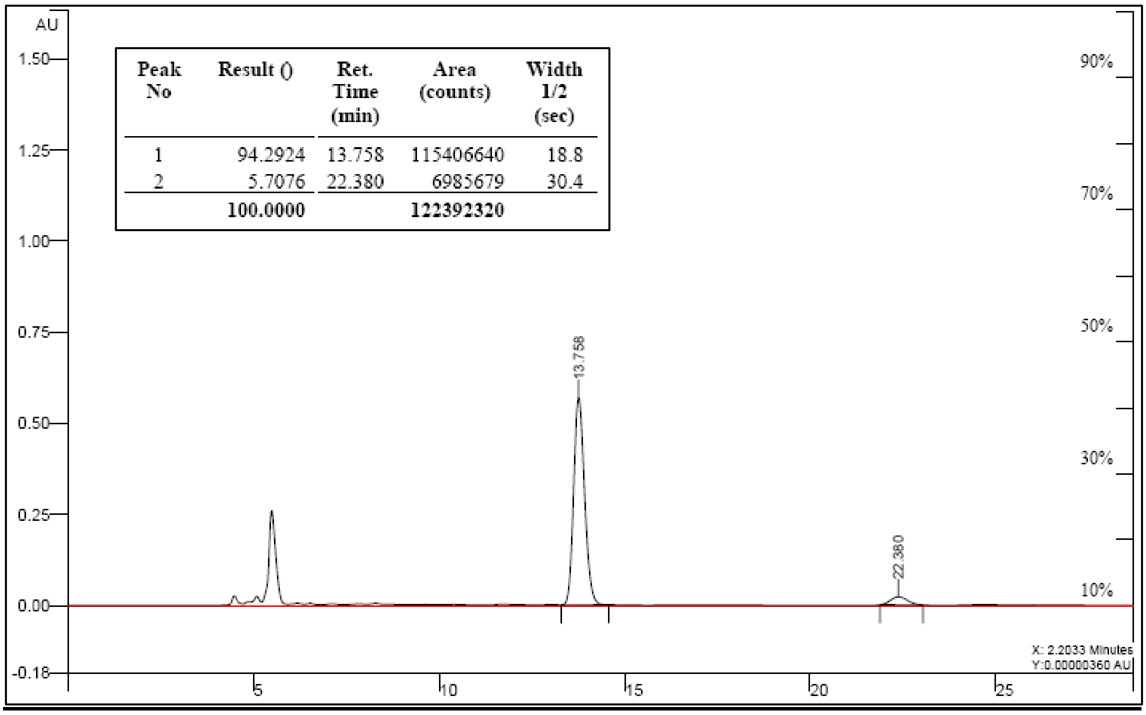

Peak No	Result ()	Ret. Time (min)	Area (counts)	Width 1/2 (sec)
1	94.2924	13.758	115406640	18.8
2	5.7076	22.380	6985679	30.4
	**100.0000**		**122392320**	

HPLC spectrum of the remaining alcohol (*R*)-**2a**

[Chiralcel OD-H, Hexane/IPA = 90/10, 0.7 ml/min, 220 nm]

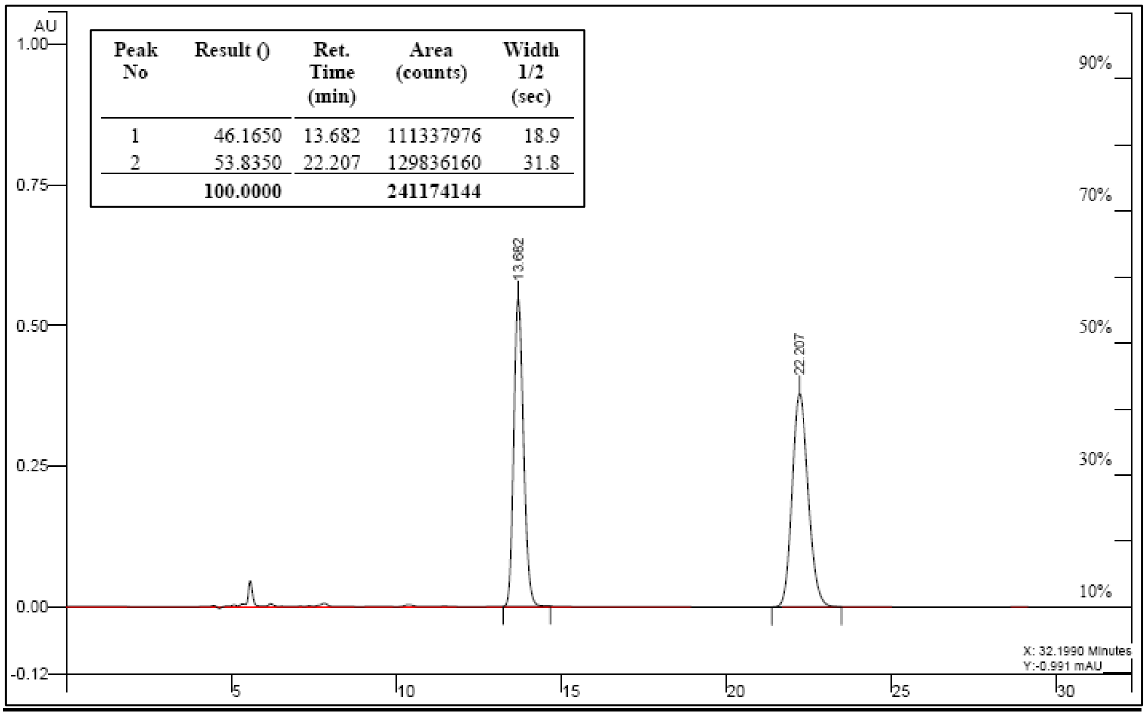

Peak No	Result ()	Ret. Time (min)	Area (counts)	Width 1/2 (sec)
1	46.1650	13.682	111337976	18.9
2	53.8350	22.207	129836160	31.8
	100.0000		241174144	

^{1}H NMR spectrum of the silylation reaction mixture of 2a after 3% conversion: cat 1h

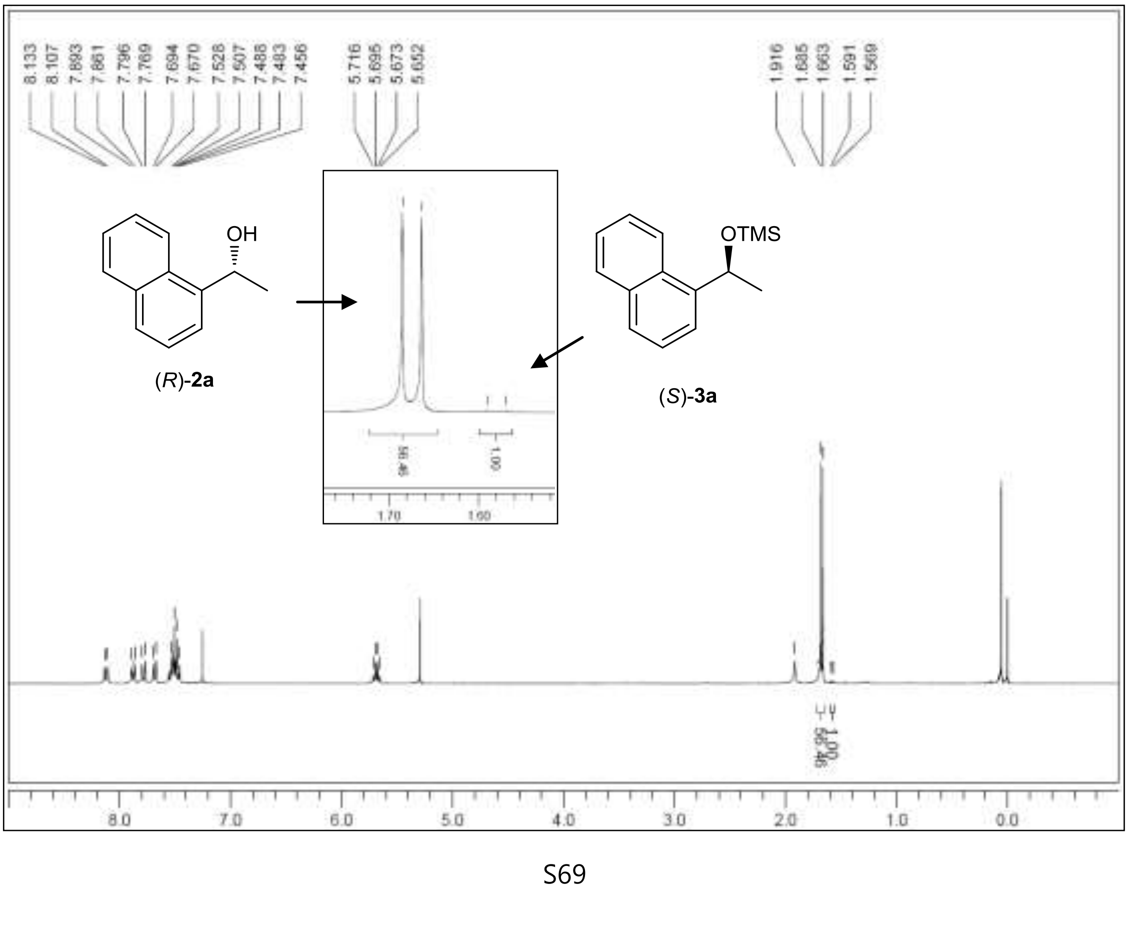

^{1}H NMR spectrum of the silylation reaction mixture of 2a after 6% conversion: cat 1k

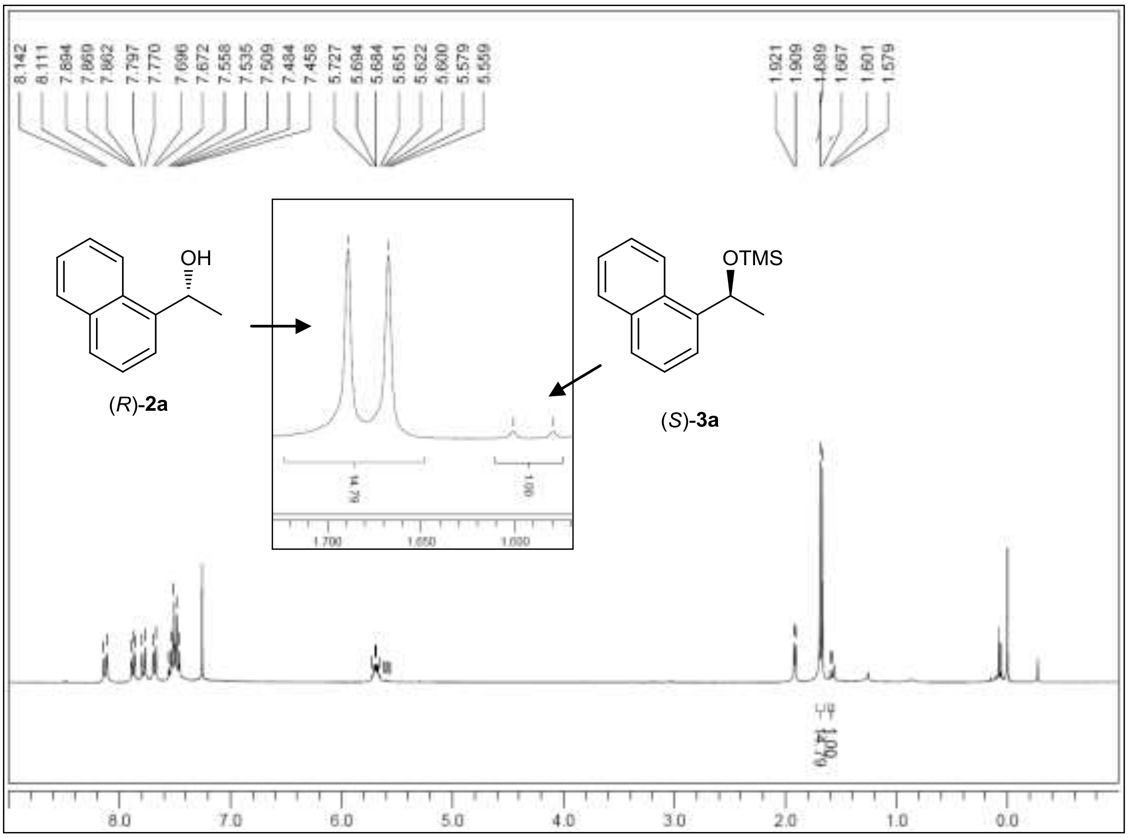

HPLC spectrum of the TMS-ether product (*S*)-**3a**

[Chiralcel OD-H, Hexane/IPA = 90/10, 0.7 ml/min, 220 nm]

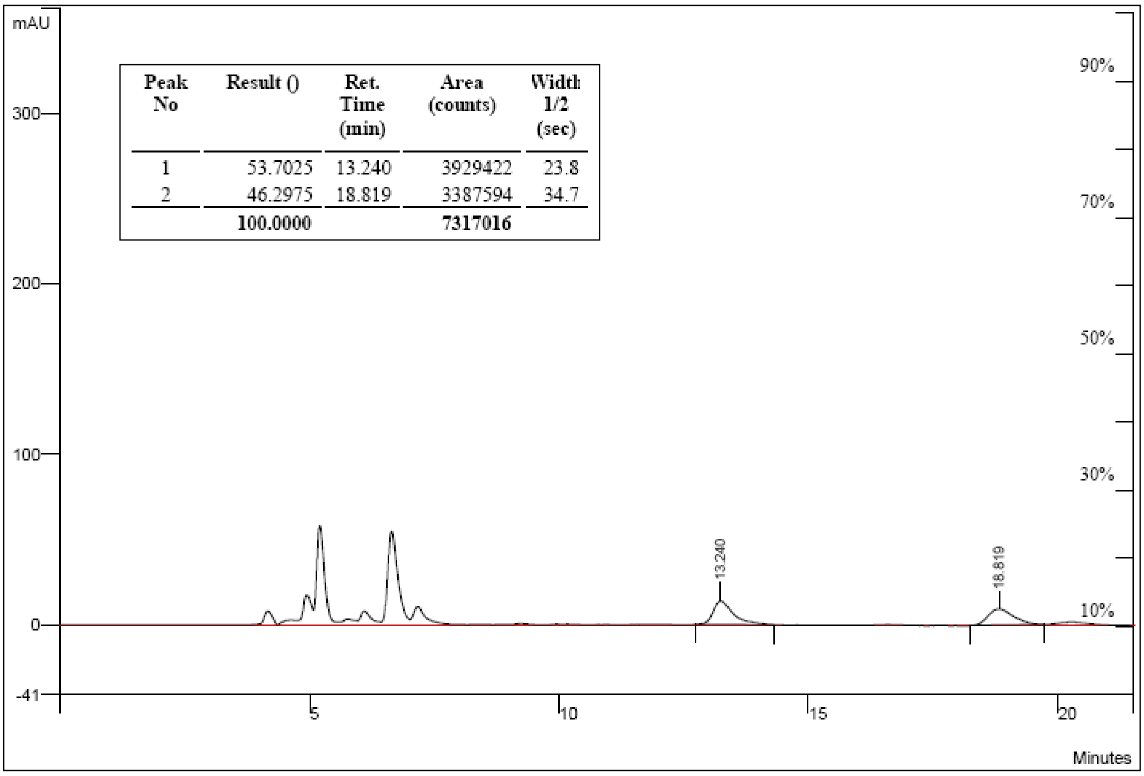

Peak No	Result ()	Ret. Time (min)	Area (counts)	Width 1/2 (sec)
1	53.7025	13.240	3929422	23.8
2	46.2975	18.819	3387594	34.7
	**100.0000**		**7317016**	

HPLC spectrum of the remaining alcohol (*R*)-**2a**

[Chiralcel OD-H, Hexane/IPA = 90/10, 0.7 ml/min, 220 nm]

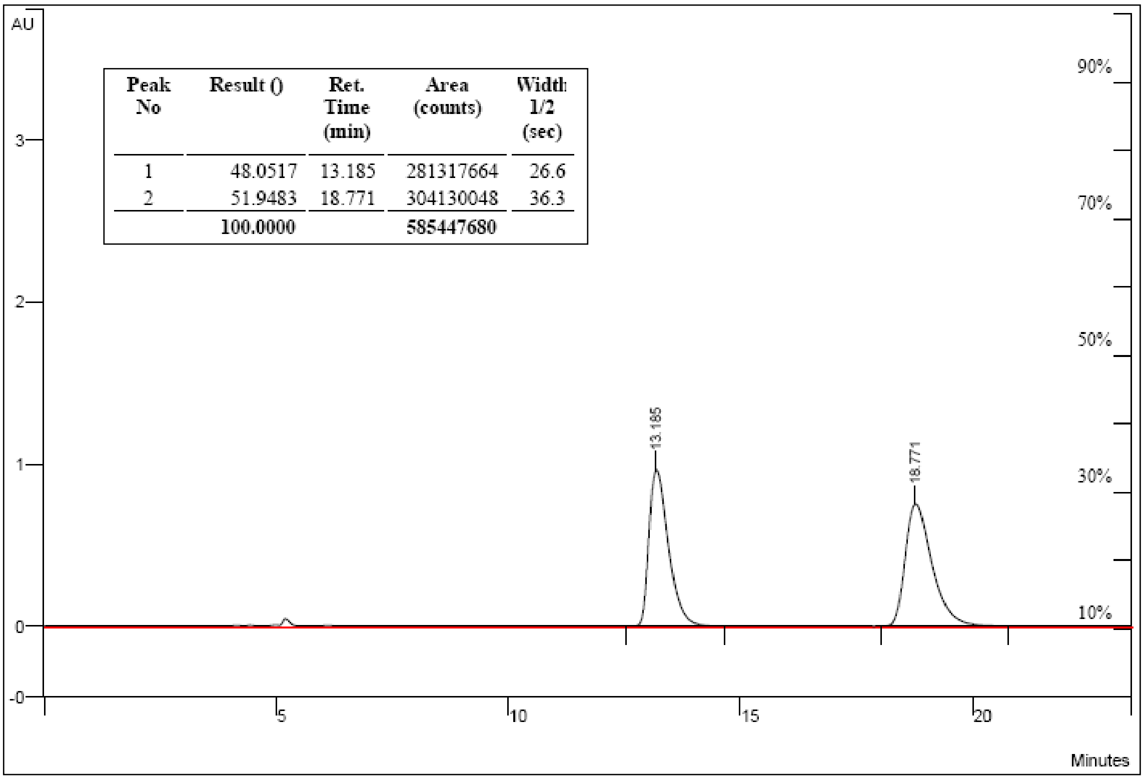

Peak No	Result ()	Ret. Time (min)	Area (counts)	Width 1/2 (sec)
1	48.0517	13.185	281317664	26.6
2	51.9483	18.771	304130048	36.3
	**100.0000**		**585447680**	

Supplementary Figure 17. ^{1}H NMR and HPLC Spectra for Table **1**

^{1}H NMR spectrum of the silylation reaction mixture of 2a after 4% conversion: entry1

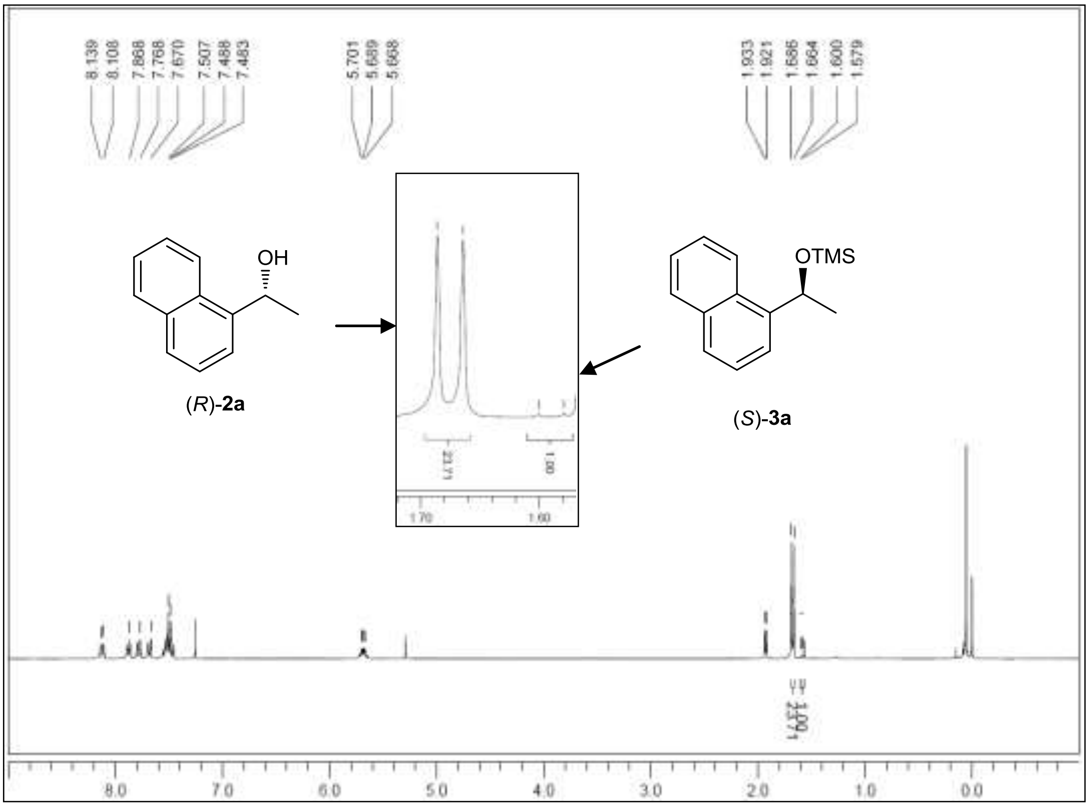

^{1}H NMR spectrum of the silylation reaction mixture of 2a after 17% conversion: entry2

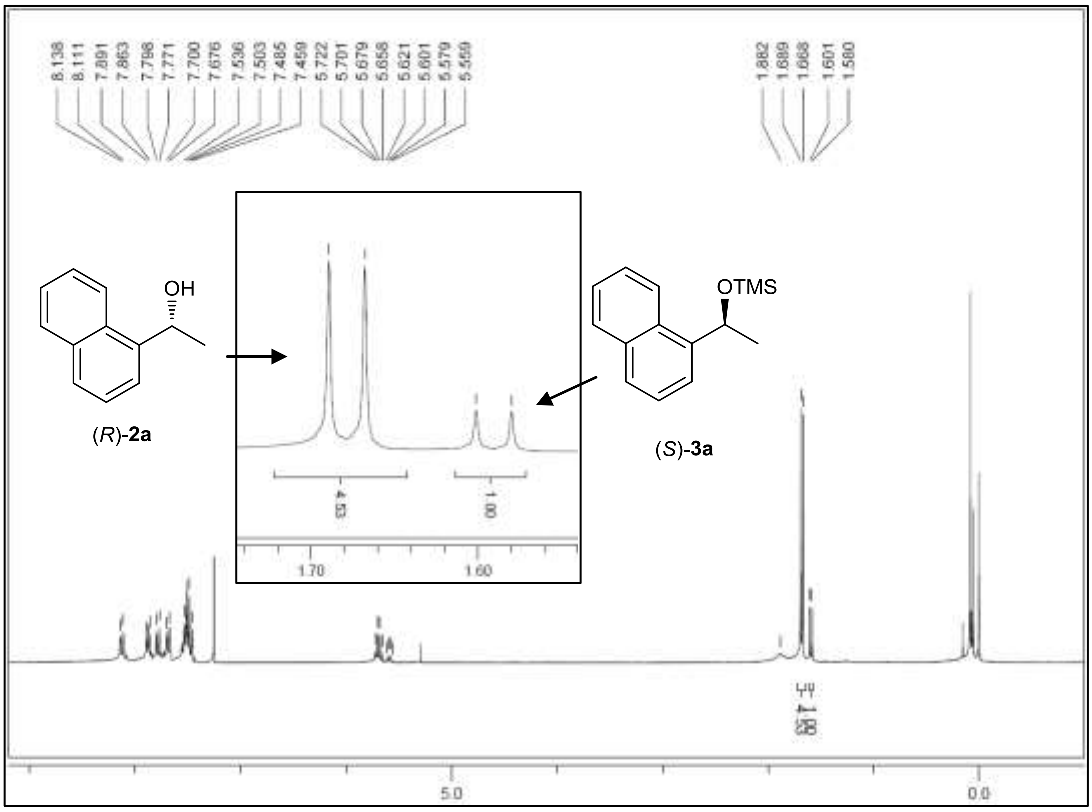

HPLC spectrum of the TMS-ether product (*S*)-**3a**

[Chiralcel OD-H, Hexane/IPA = 90/10, 0.7 ml/min, 220 nm]

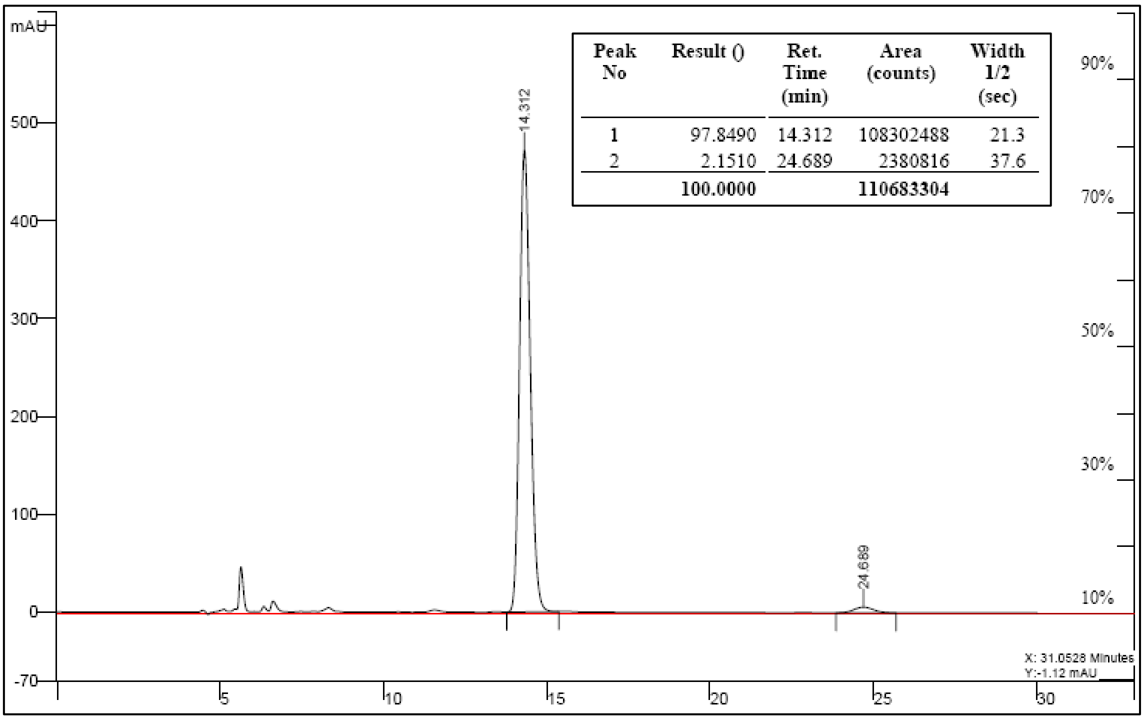

Peak No	Result ()	Ret. Time (min)	Area (counts)	Width 1/2 (sec)
1	97.8490	14.312	108302488	21.3
2	2.1510	24.689	2380816	37.6
	**100.0000**		**110683304**	

HPLC spectrum of the remaining alcohol (*R*)-**2a**

[Chiralcel OD-H, Hexane/IPA = 90/10, 0.7 ml/min, 220 nm]

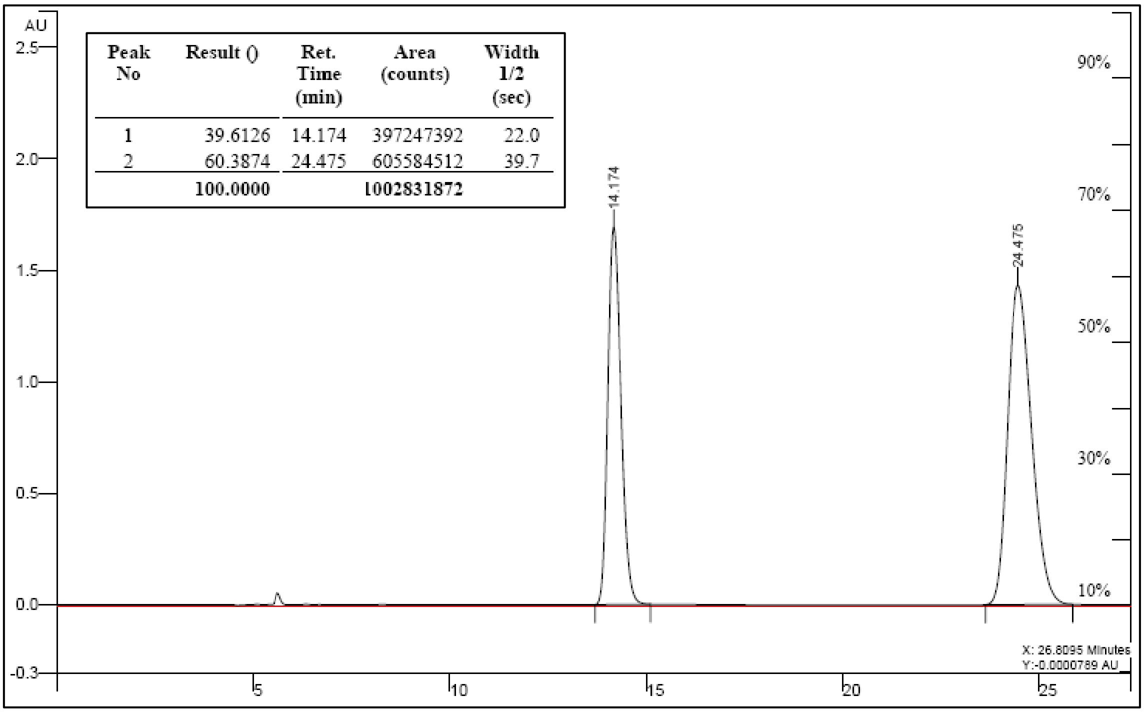

Peak No	Result ()	Ret. Time (min)	Area (counts)	Width 1/2 (sec)
1	39.6126	14.174	397247392	22.0
2	60.3874	24.475	605584512	39.7
	**100.0000**		**1002831872**	

^{1}H NMR spectrum of the silylation reaction mixture of 2a after 15% conversion: entry3

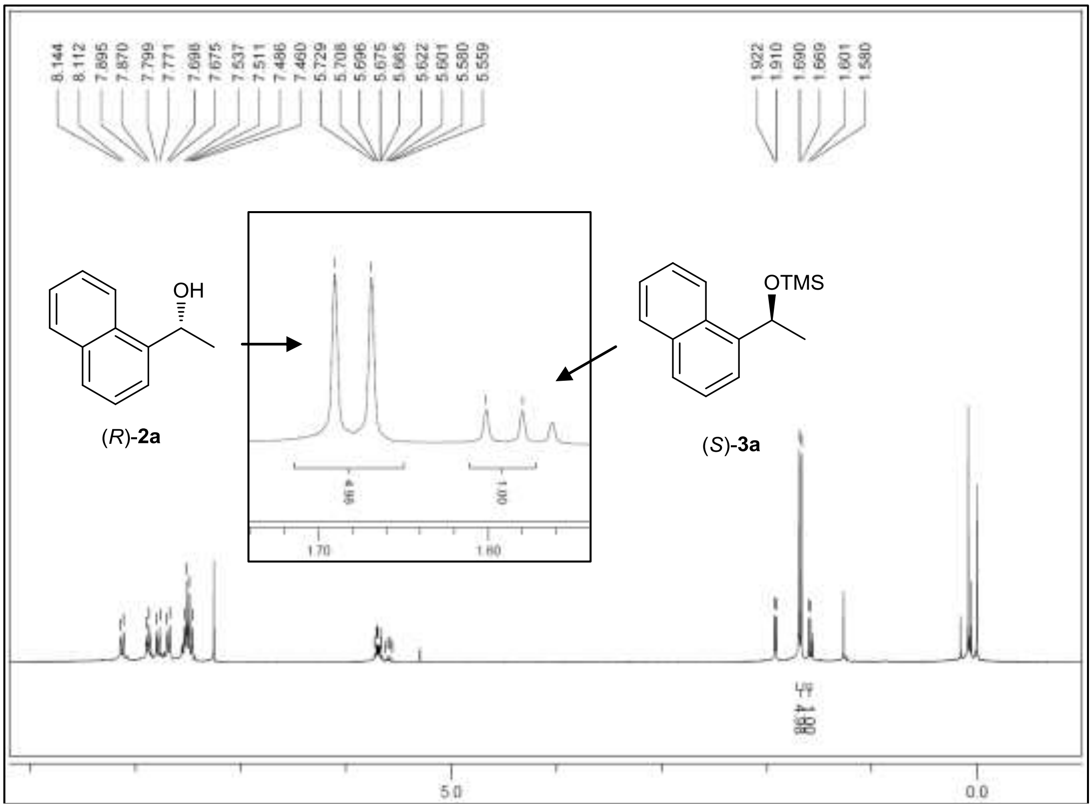

HPLC spectrum of the TMS-ether product (*S*)-**3a**

[Chiralcel OD-H, Hexane/IPA = 90/10, 0.7 ml/min, 220 nm]

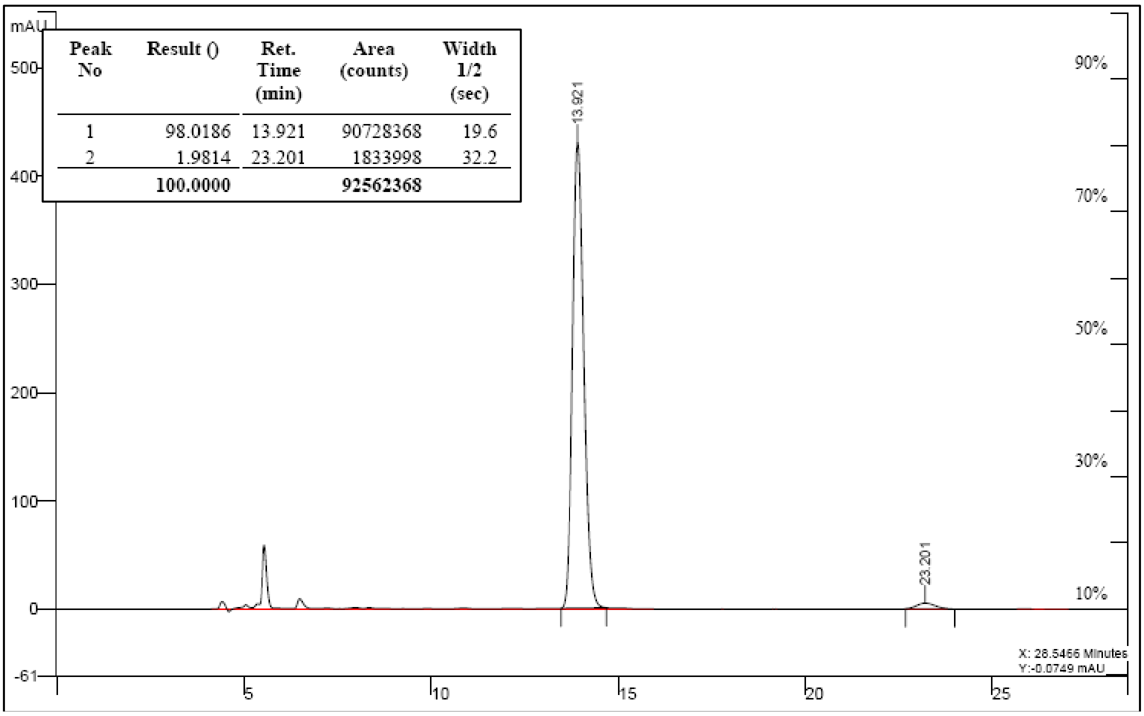

Peak No	Result ()	Ret. Time (min)	Area (counts)	Width 1/2 (sec)
1	98.0186	13.921	90728368	19.6
2	1.9814	23.201	1833998	32.2
	**100.0000**		**92562368**	

HPLC spectrum of the remaining alcohol (*R*)-**2a**

[Chiralcel OD-H, Hexane/IPA = 90/10, 0.7 ml/min, 220 nm]

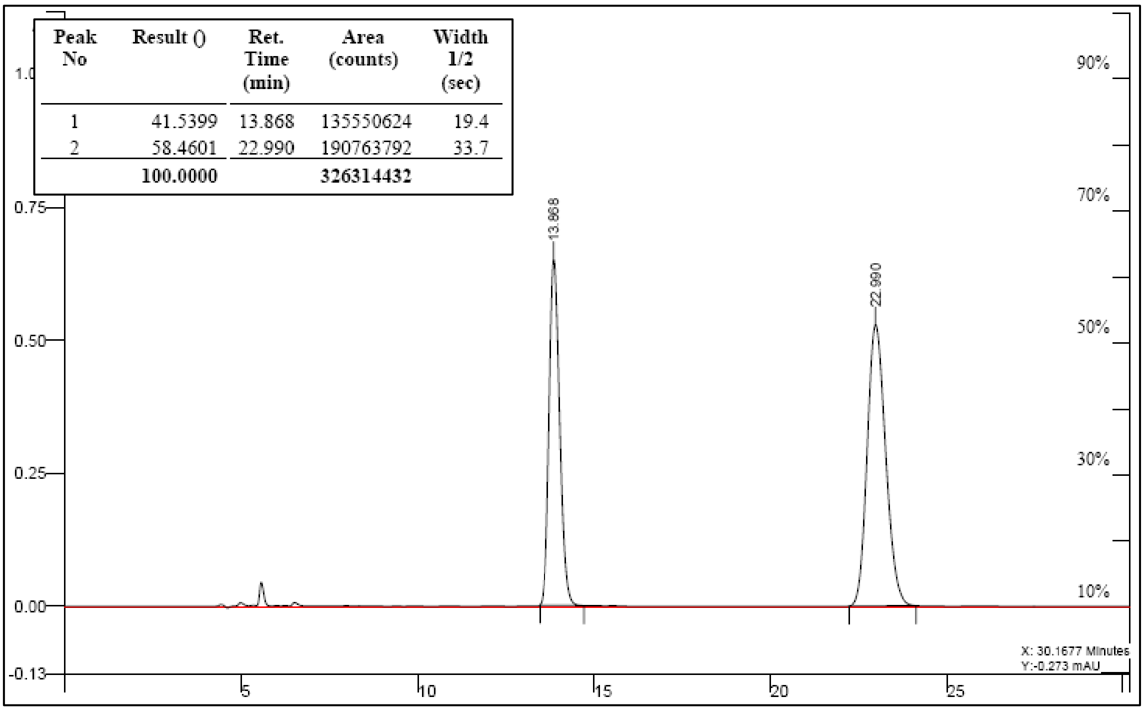

^{1}H NMR spectrum of the silylation reaction mixture of 2a after 18% conversion: entry4

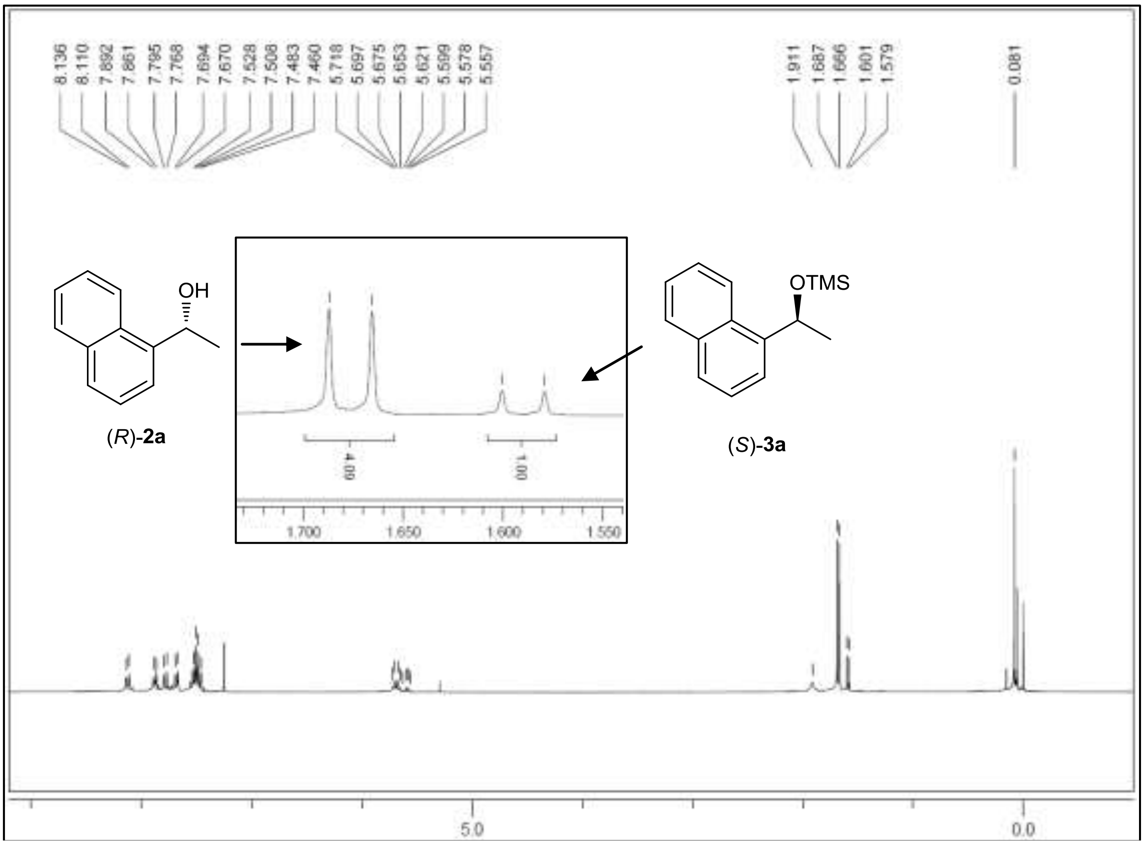

HPLC spectrum of the TMS-ether product (*S*)-**3a**

[Chiralcel OD-H, Hexane/IPA = 90/10, 0.7 ml/min, 220 nm]

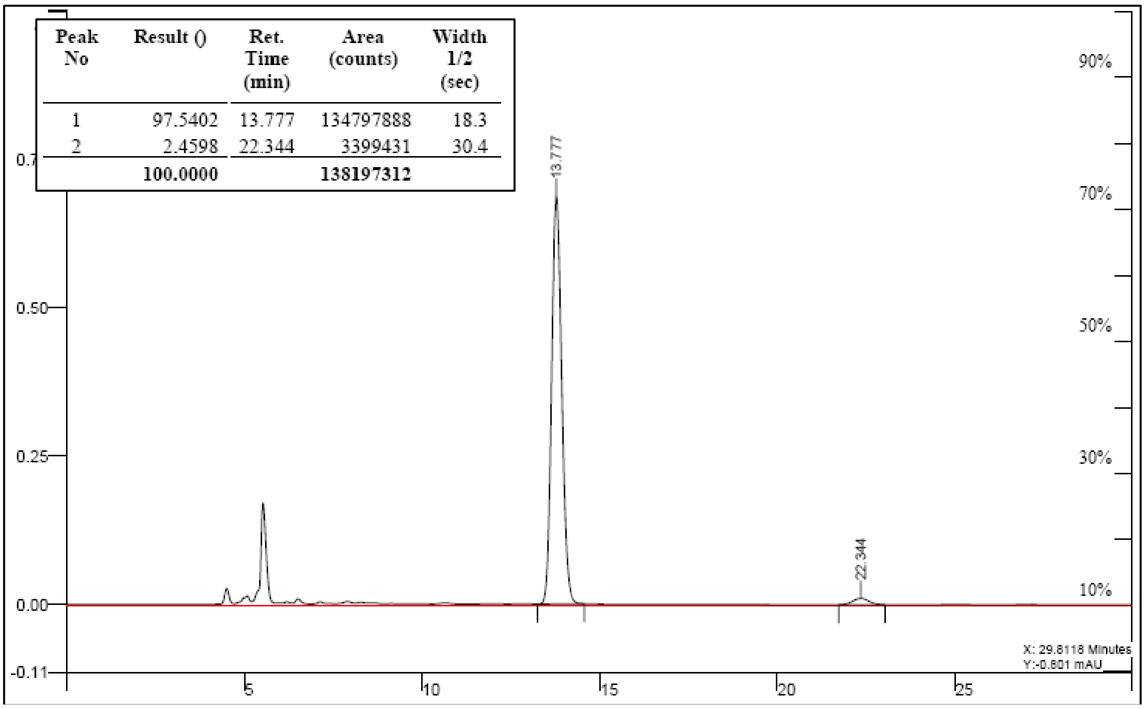

Peak No	Result ()	Ret. Time (min)	Area (counts)	Width 1/2 (sec)
1	97.5402	13.777	134797888	18.3
2	2.4598	22.344	3399431	30.4
	**100.0000**		**138197312**	

HPLC spectrum of the remaining alcohol (*R*)-**2a**

[Chiralcel OD-H, Hexane/IPA = 90/10, 0.7 ml/min, 220 nm]

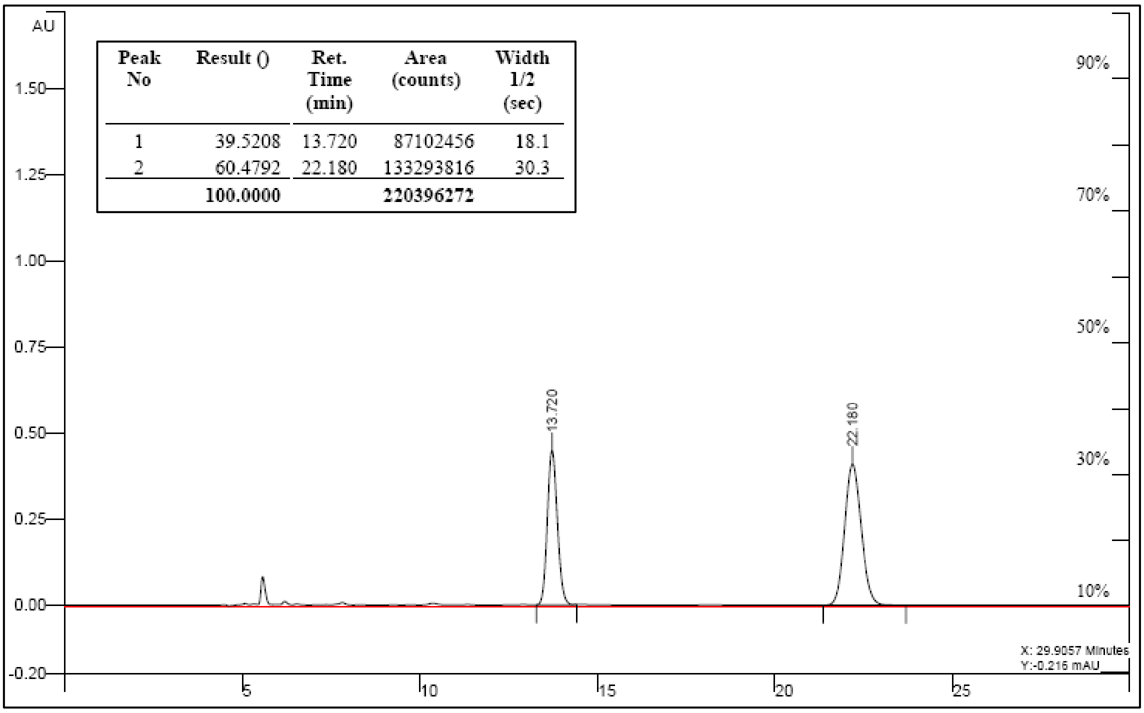

Peak No	Result ()	Ret. Time (min)	Area (counts)	Width 1/2 (sec)
1	39.5208	13.720	87102456	18.1
2	60.4792	22.180	133293816	30.3
	**100.0000**		**220396272**	

^{1}H NMR spectrum of the silylation reaction mixture of 2a after 52% conversion: entry5

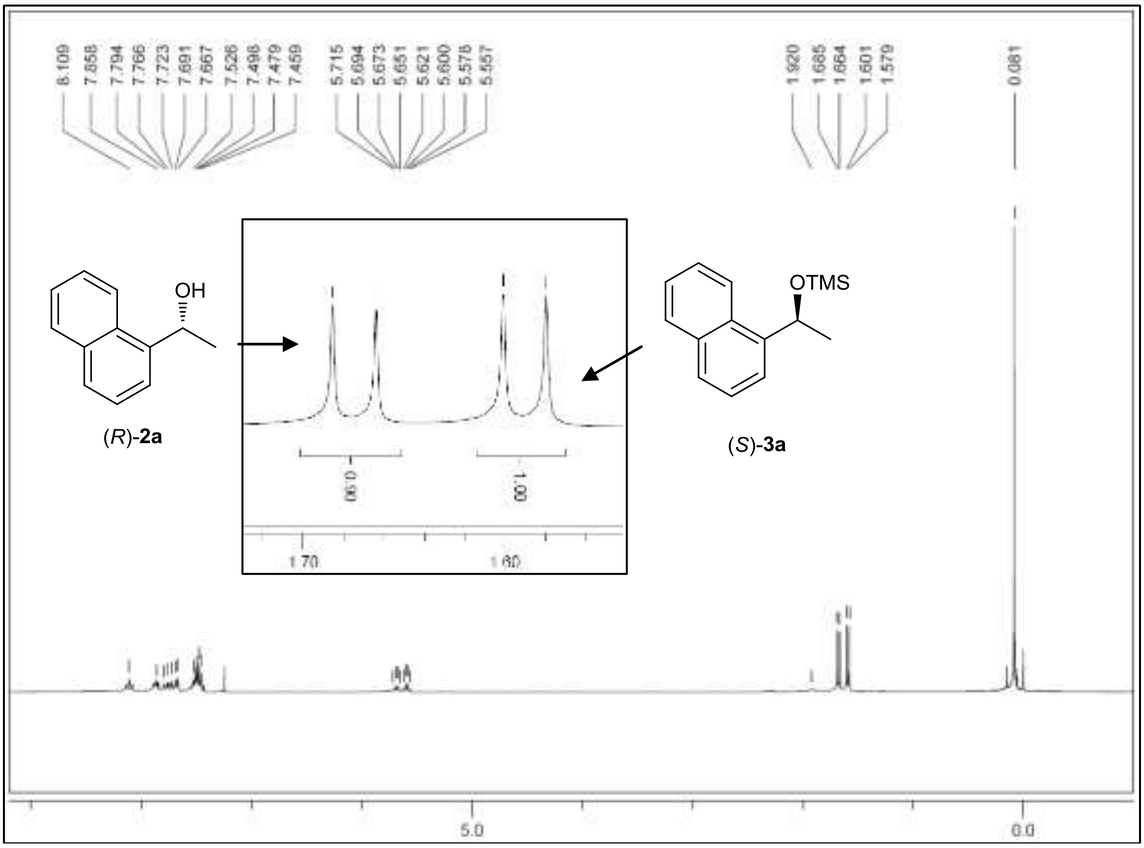

HPLC spectrum of the TMS-ether product (*S*)-**3a**

[Chiralcel OD-H, Hexane/IPA = 90/10, 0.7 ml/min, 220 nm]

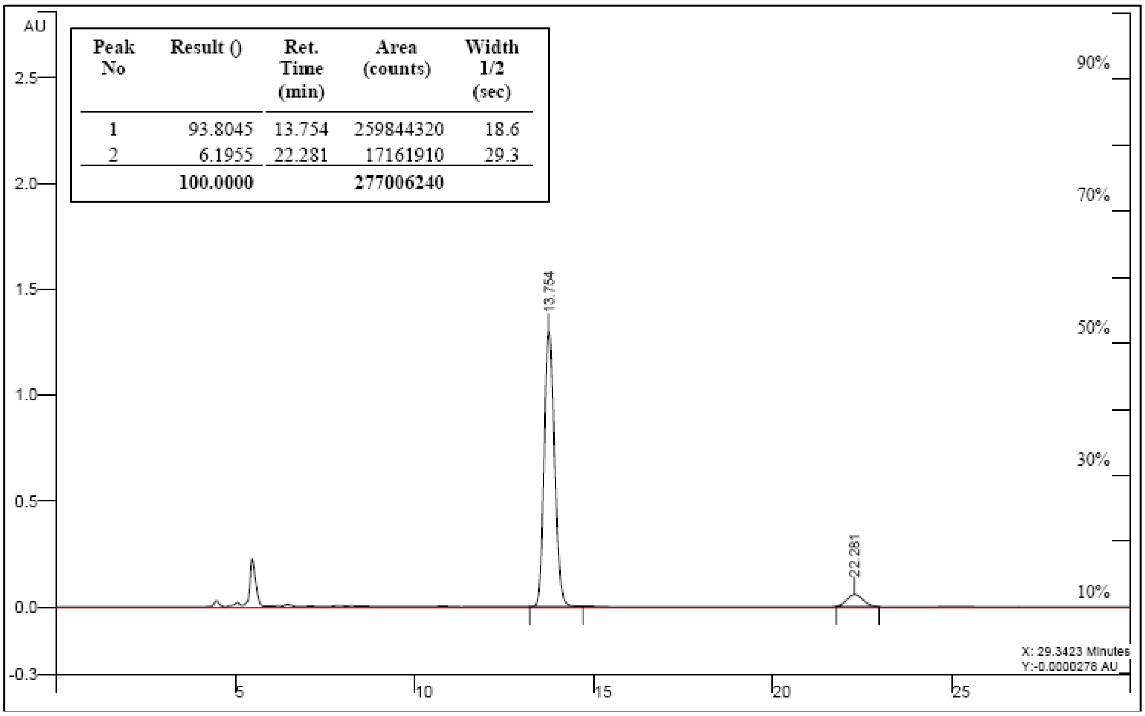

Peak No	Result ()	Ret. Time (min)	Area (counts)	Width 1/2 (sec)
1	93.8045	13.754	259844320	18.6
2	6.1955	22.281	17161910	29.3
	100.0000		277006240	

HPLC spectrum of the remaining alcohol (*R*)-**2a**

[Chiralcel OD-H, Hexane/IPA = 90/10, 0.7 ml/min, 220 nm]

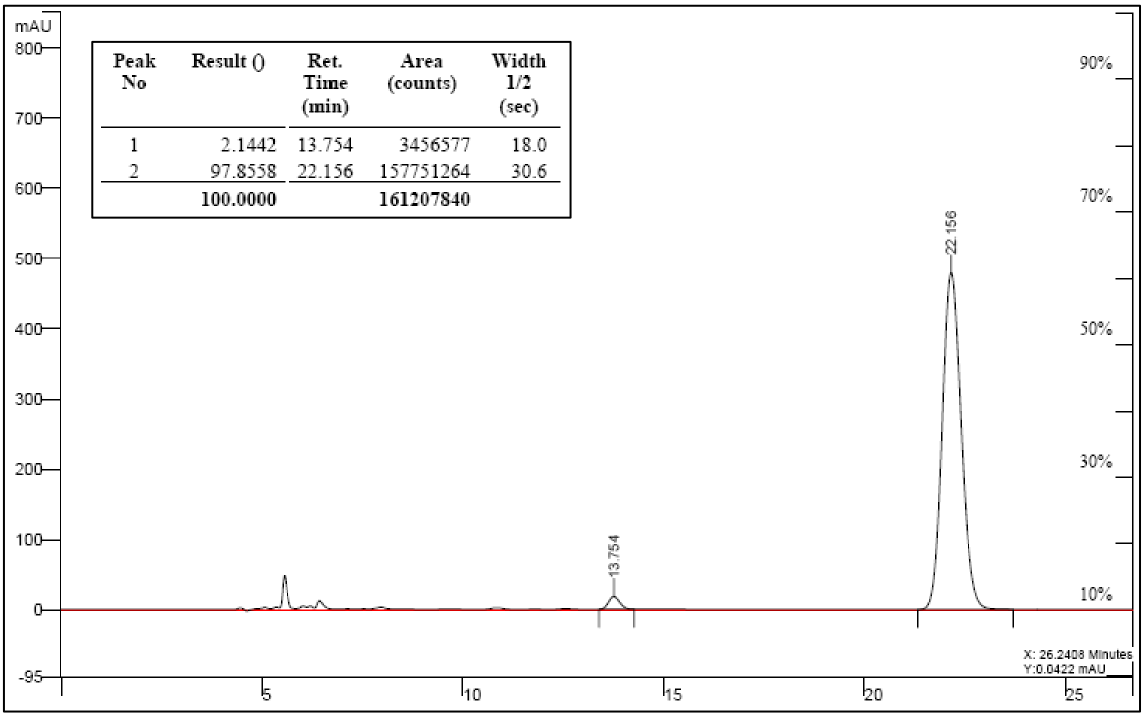

^{1}H NMR spectrum of the silylation reaction mixture of 2a after 51% conversion: entry6

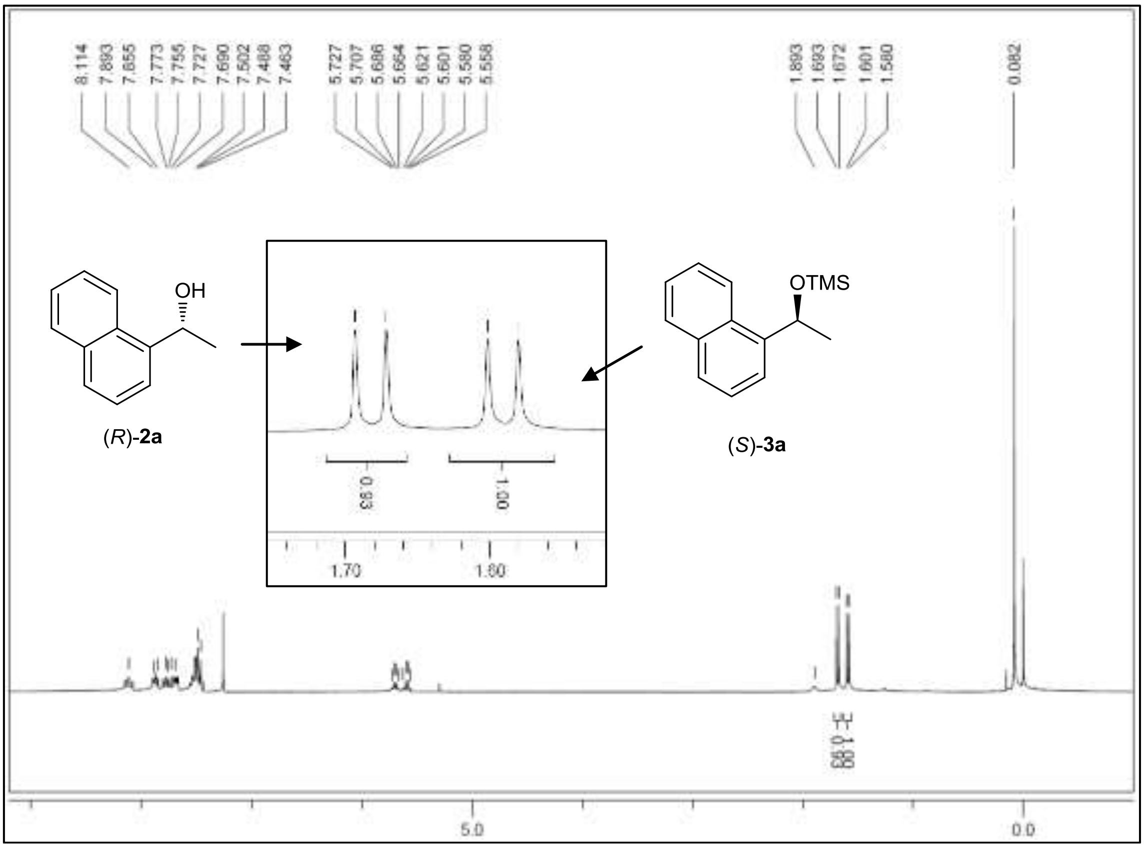

HPLC spectrum of the TMS-ether product (*S*)-**3a**

[Chiralcel OD-H, Hexane/IPA = 90/10, 0.7 ml/min, 220 nm]

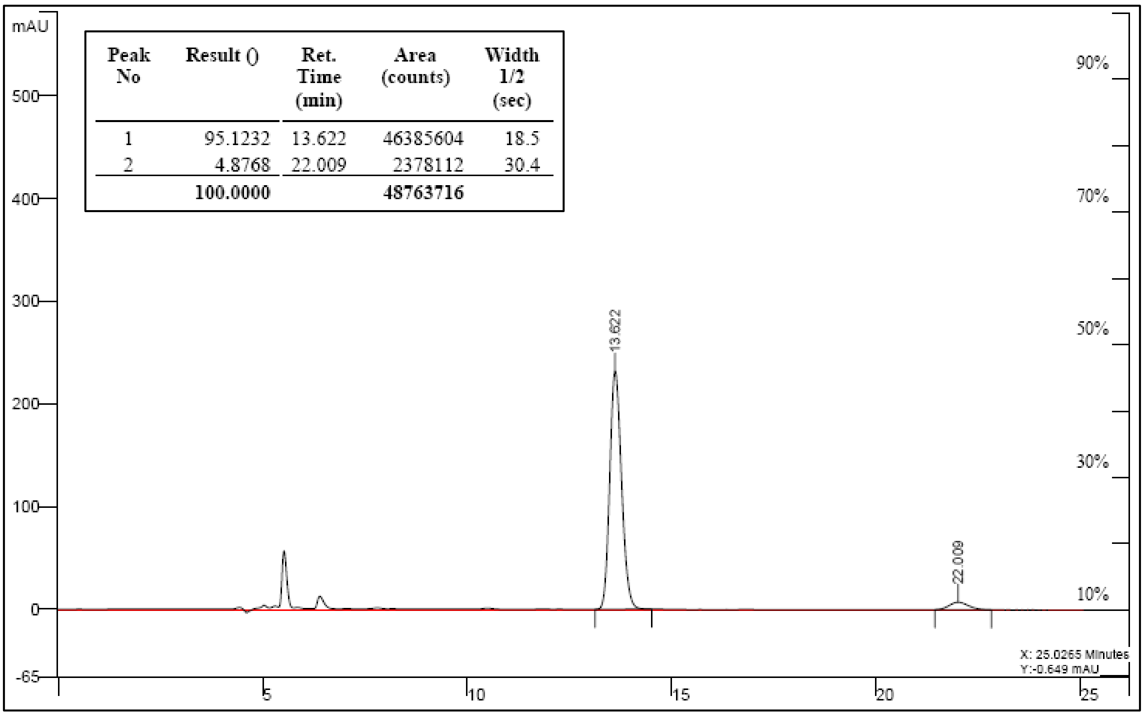

Peak No	Result ()	Ret. Time (min)	Area (counts)	Width 1/2 (sec)
1	95.1232	13.622	46385604	18.5
2	4.8768	22.009	2378112	30.4
	**100.0000**		**48763716**	

HPLC spectrum of the remaining alcohol (*R*)-**2a**

[Chiralcel OD-H, Hexane/IPA = 90/10, 0.7 ml/min, 220 nm]

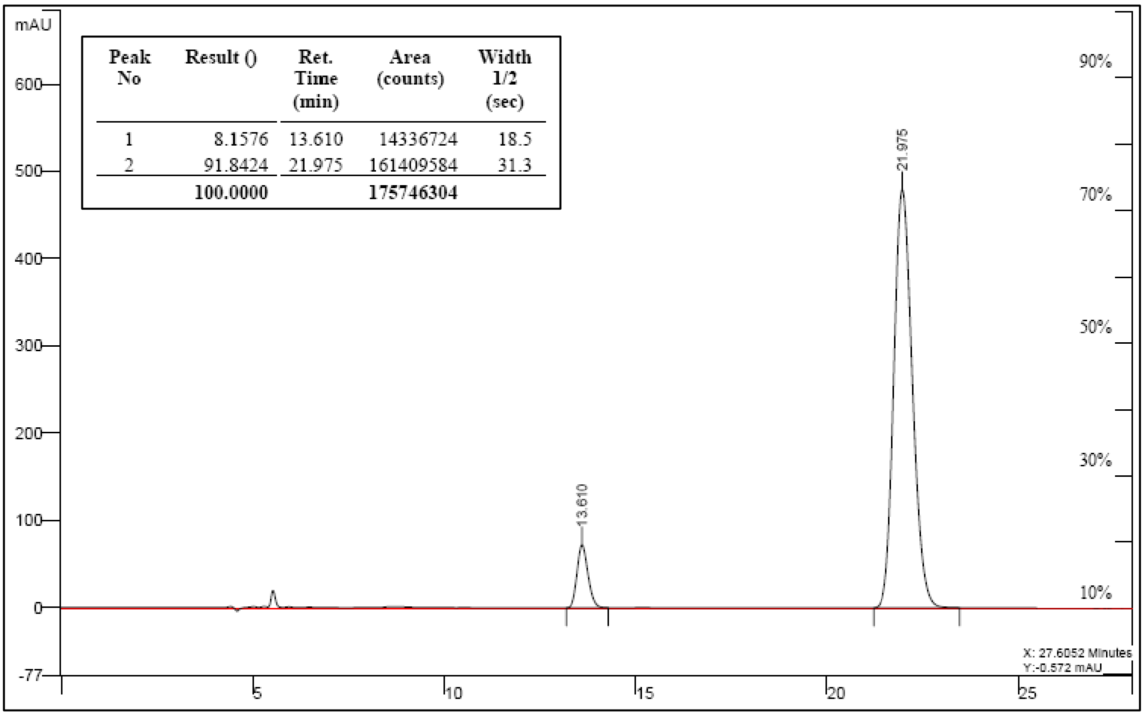

Peak No	Result ()	Ret. Time (min)	Area (counts)	Width 1/2 (sec)
1	8.1576	13.610	14336724	18.5
2	91.8424	21.975	161409584	31.3
	**100.0000**		**175746304**	

^{1}H NMR spectrum of the silylation reaction mixture of 2a after 50% conversion: entry8

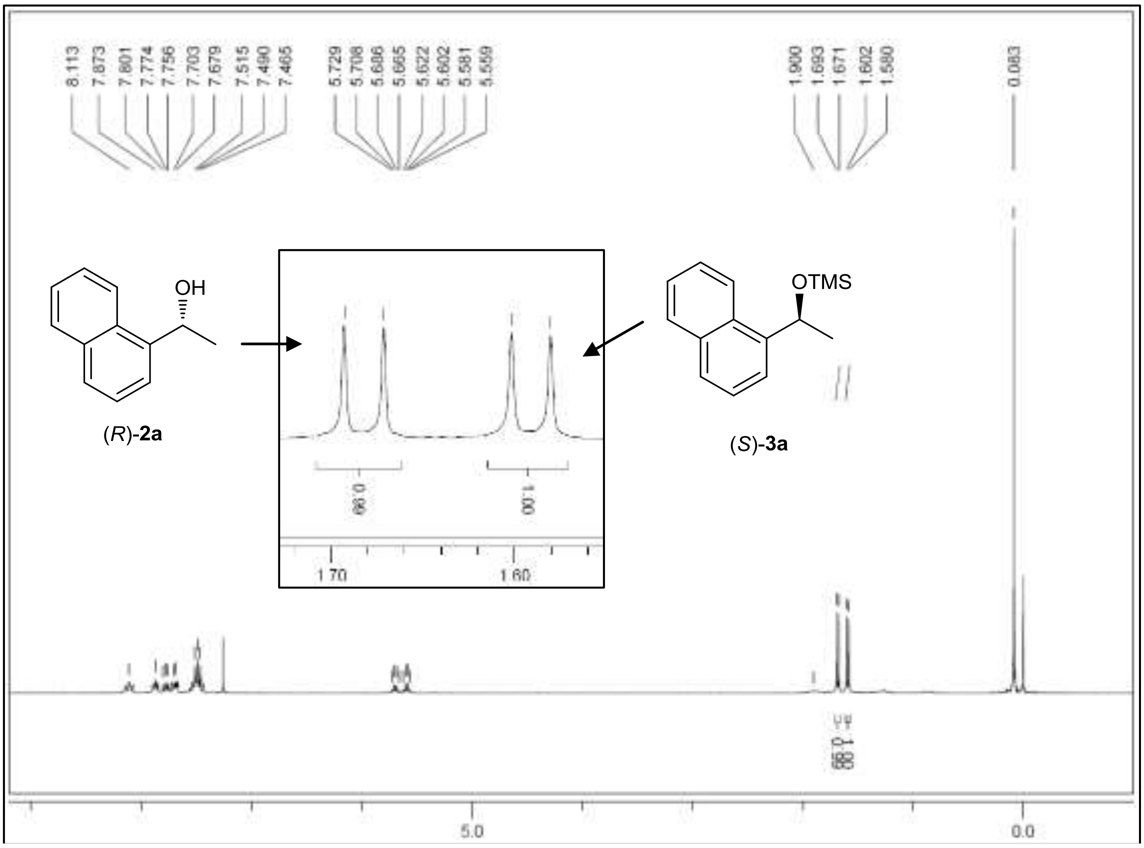

HPLC spectrum of the TMS-ether product (*S*)-**3a**

[Chiralcel OD-H, Hexane/IPA = 90/10, 0.7 ml/min, 220 nm]

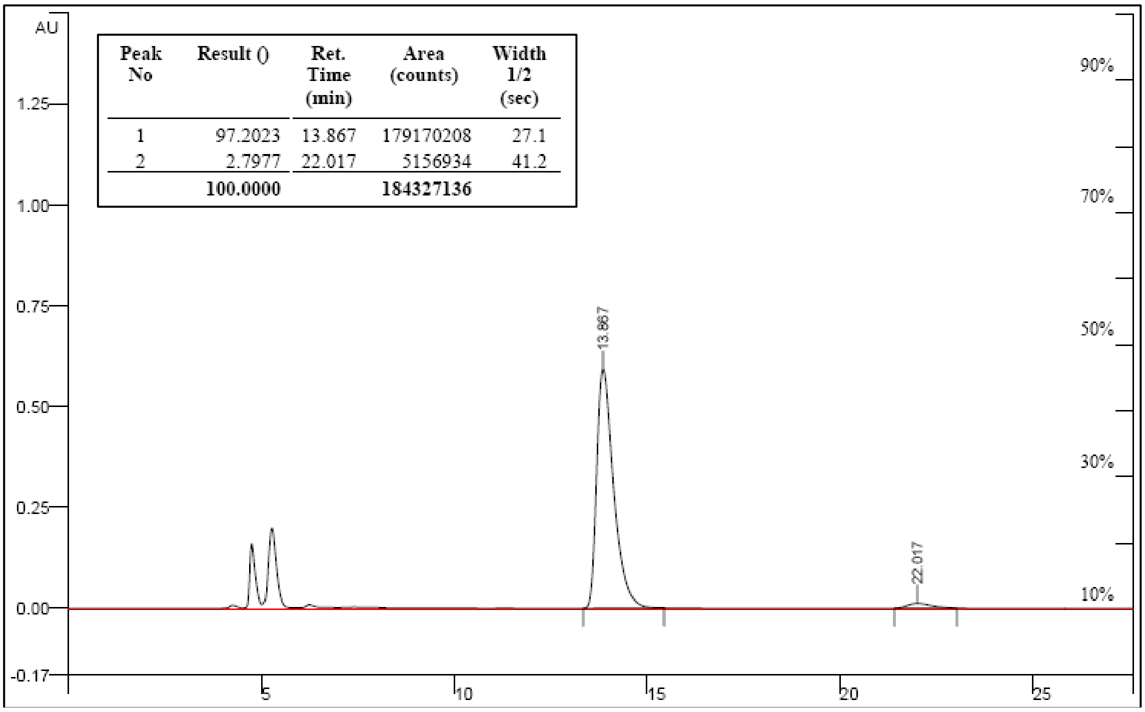

Peak No	Result ()	Ret. Time (min)	Area (counts)	Width 1/2 (sec)
1	97.2023	13.867	179170208	27.1
2	2.7977	22.017	5156934	41.2
	**100.0000**		**184327136**	

HPLC spectrum of the remaining alcohol (*R*)-**2a**

[Chiralcel OD-H, Hexane/IPA = 90/10, 0.7 ml/min, 220 nm]

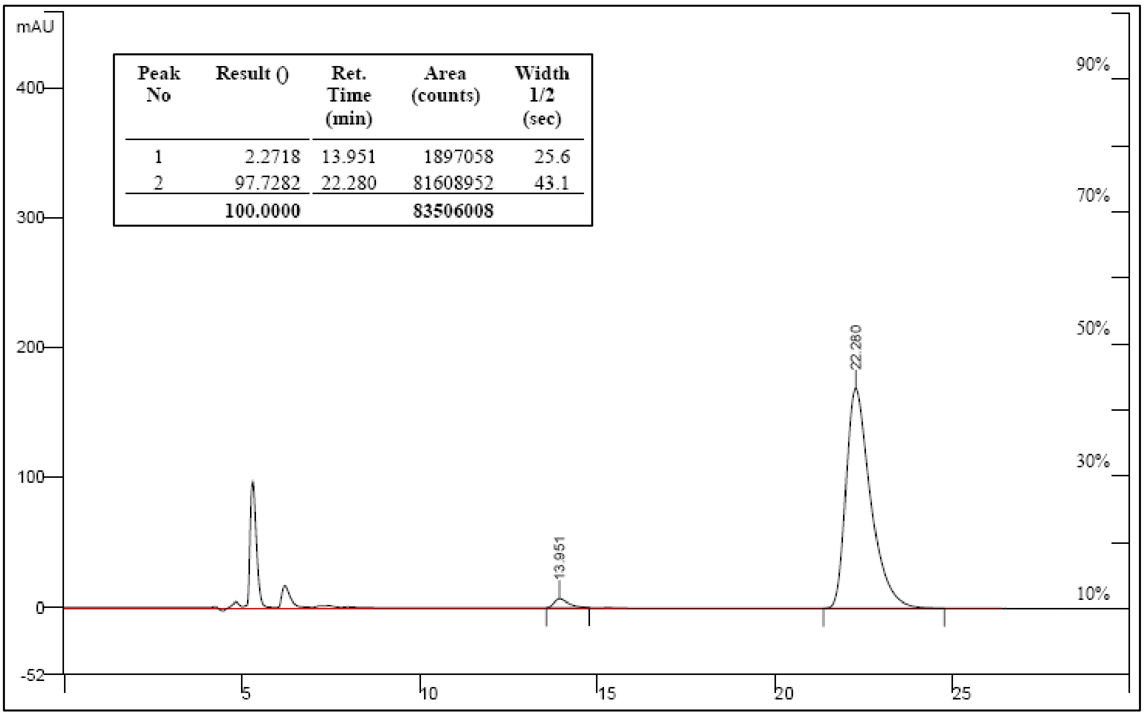

^{1}H NMR spectrum of the silylation reaction mixture of 2a after 52% conversion entry9

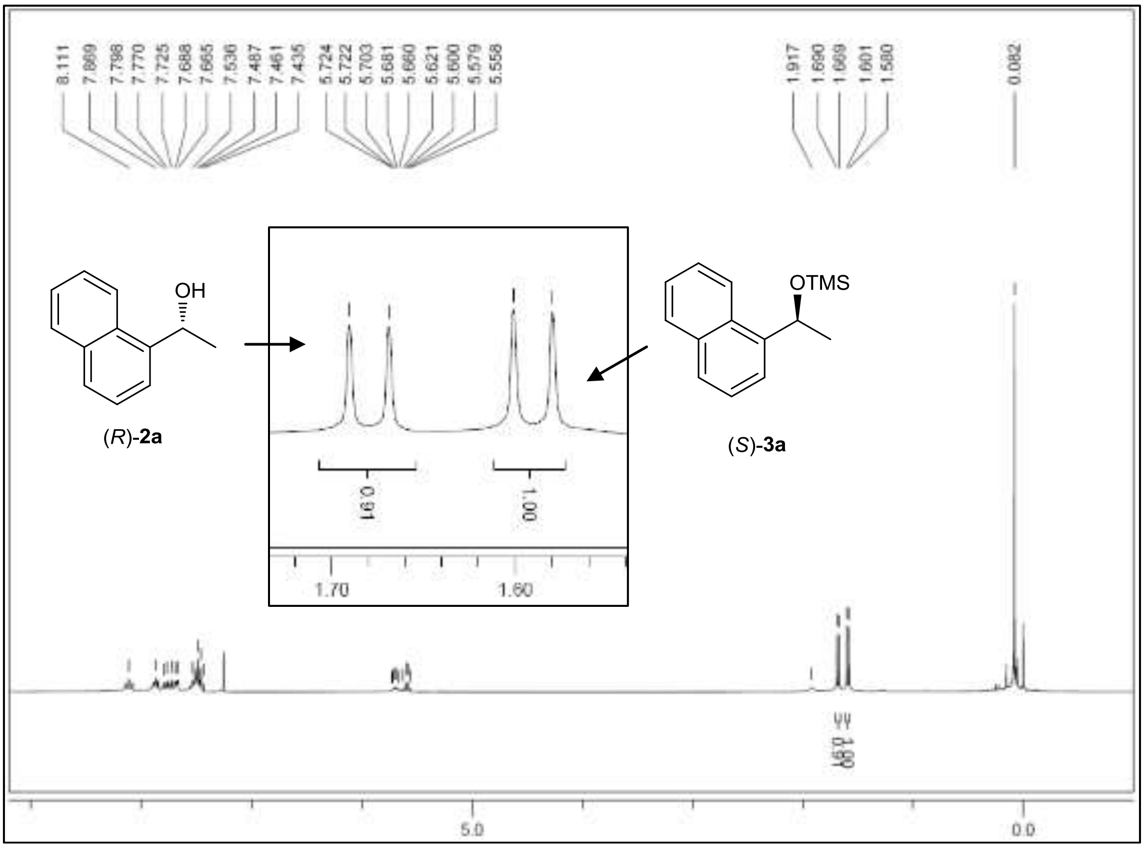

HPLC spectrum of the TMS-ether product (*S*)-**3a**

[Chiralcel OD-H, Hexane/IPA = 90/10, 0.7 ml/min, 220 nm]

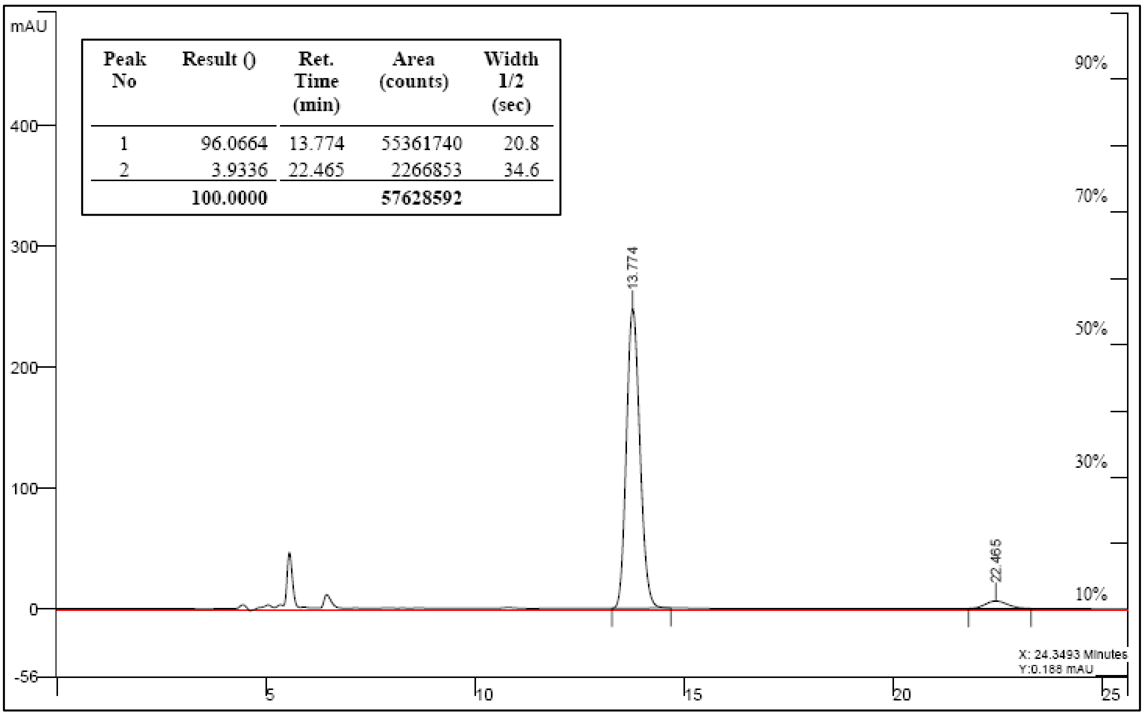

Peak No	Result ()	Ret. Time (min)	Area (counts)	Width 1/2 (sec)
1	96.0664	13.774	55361740	20.8
2	3.9336	22.465	2266853	34.6
	**100.0000**		**57628592**	

HPLC spectrum of the remaining alcohol (*R*)-**2a**

[Chiralcel OD-H, Hexane/IPA = 90/10, 0.7 ml/min, 220 nm]

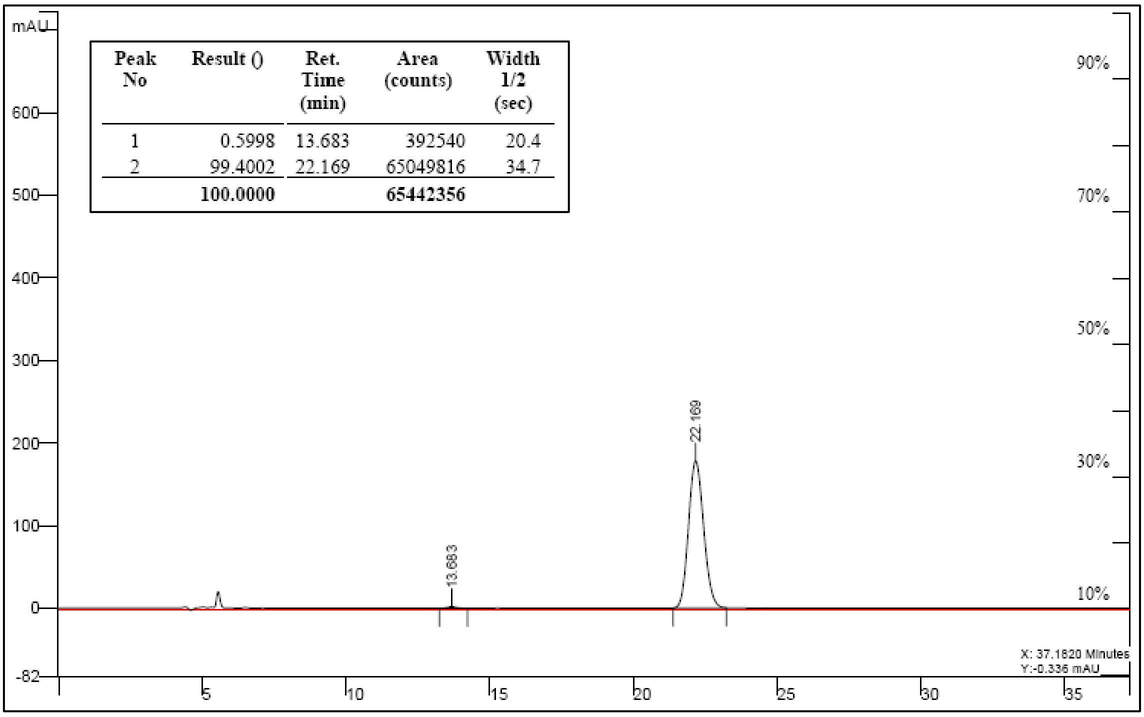

Peak No	Result ()	Ret. Time (min)	Area (counts)	Width 1/2 (sec)
1	0.5998	13.683	392540	20.4
2	99.4002	22.169	65049816	34.7
	**100.0000**		**65442356**	

^{1}H NMR spectrum of the silylation reaction mixture of 2a after 33% conversion entry10

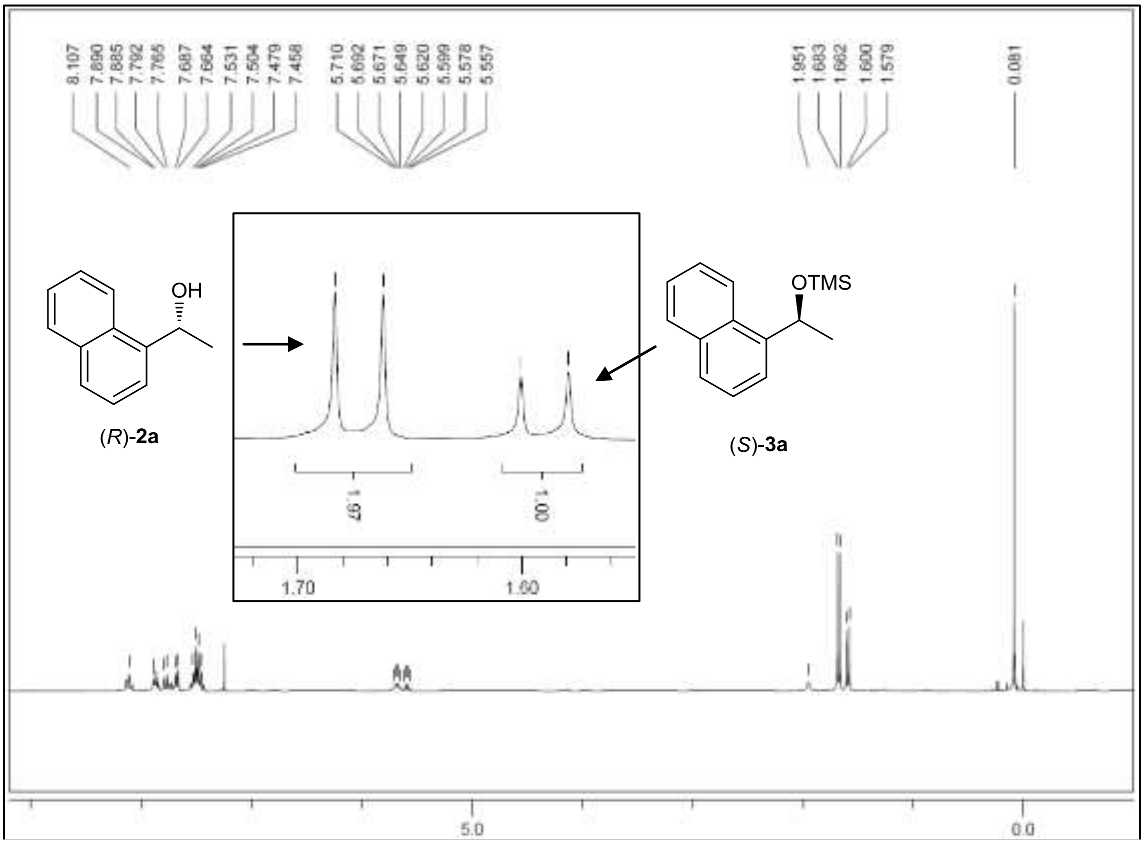

HPLC spectrum of the TMS-ether product (*S*)-**3a**

[Chiralcel OD-H, Hexane/IPA = 90/10, 0.7 ml/min, 220 nm]

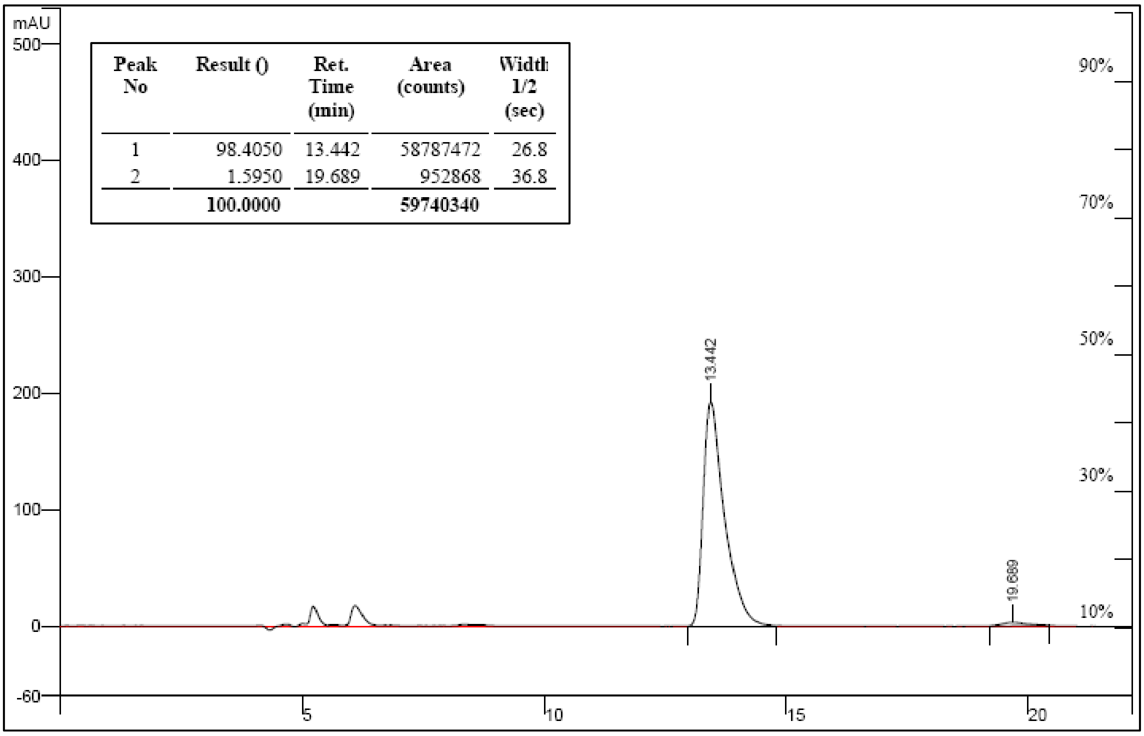

Peak No	Result ()	Ret. Time (min)	Area (counts)	Width 1/2 (sec)
1	98.4050	13.442	58787472	26.8
2	1.5950	19.689	952868	36.8
	**100.0000**		**59740340**	

HPLC spectrum of the remaining alcohol (*R*)-**2a**

[Chiralcel OD-H, Hexane/IPA = 90/10, 0.7 ml/min, 220 nm]

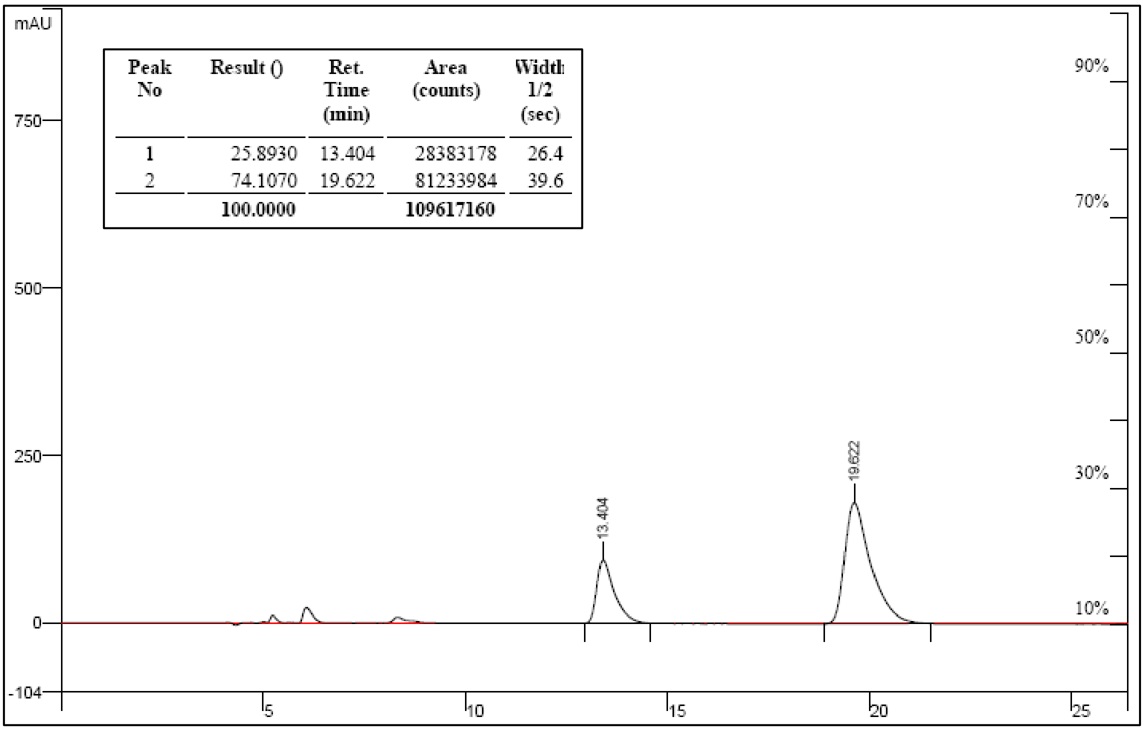

Supplementary Figure 18. ^{1}H NMR and HPLC Spectra for Figure **4**

^{1}H NMR spectrum of the silylation reaction mixture of 2a after 50.3% conversion

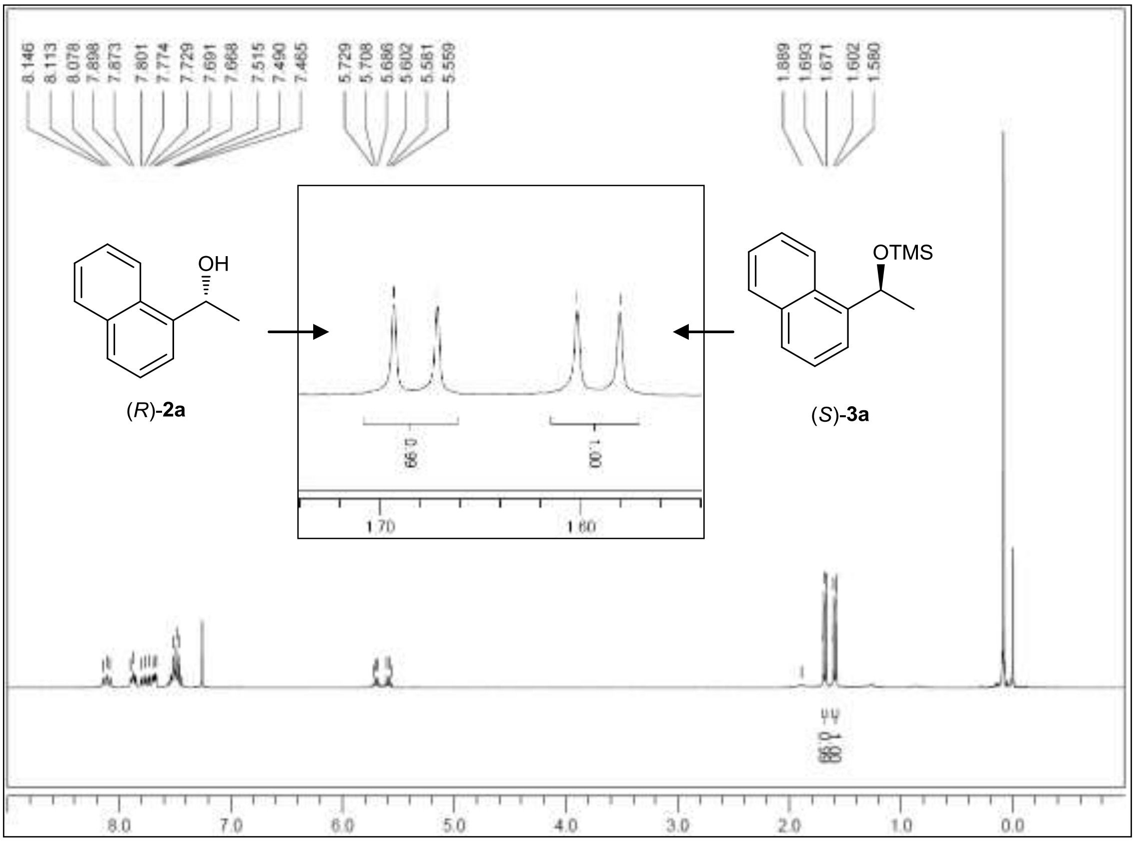

HPLC spectrum of *rac*-**2a** (Chiralcel OD-H, Hexane/IPA = 90/10, 0.7 ml/min, 220 nm)

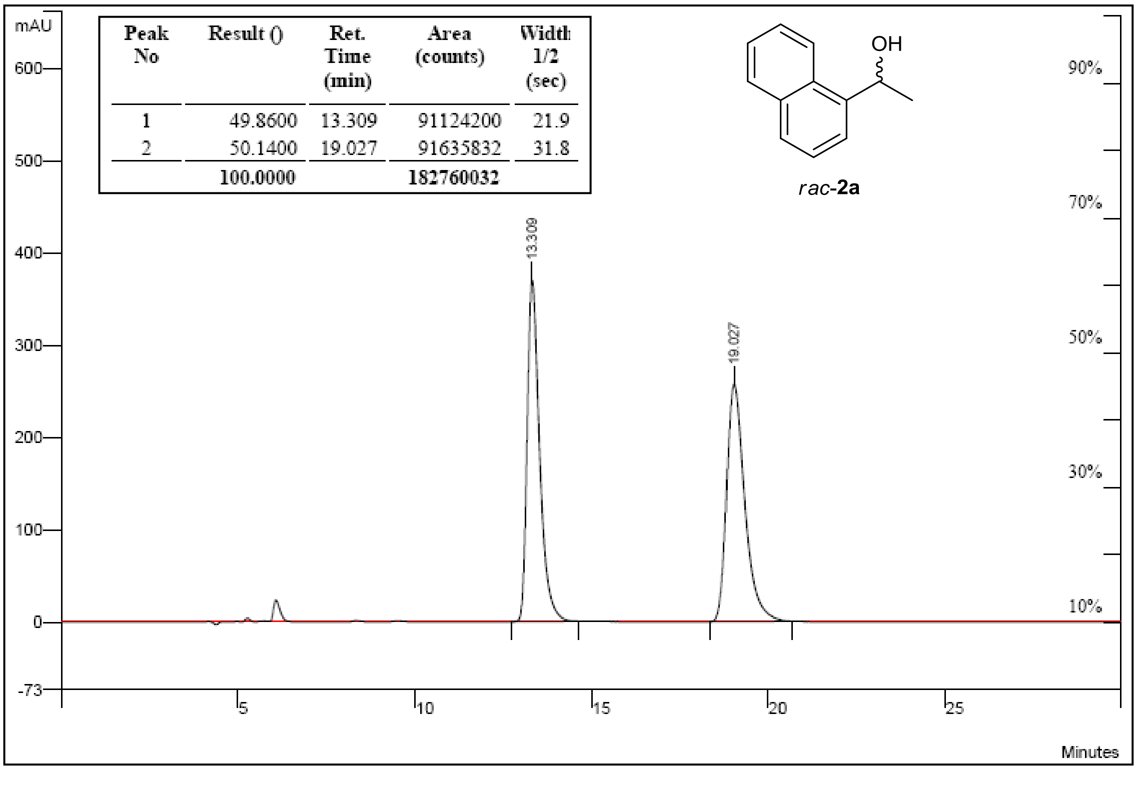

Peak No	Result ()	Ret. Time (min)	Area (counts)	Width 1/2 (sec)
1	49.8600	13.309	91124200	21.9
2	50.1400	19.027	91635832	31.8
	**100.0000**		**182760032**	

HPLC spectrum of the TMS-ether product (*S*)-**3a**

[Chiralcel OD-H, Hexane/IPA = 90/10, 0.7 ml/min, 220 nm]

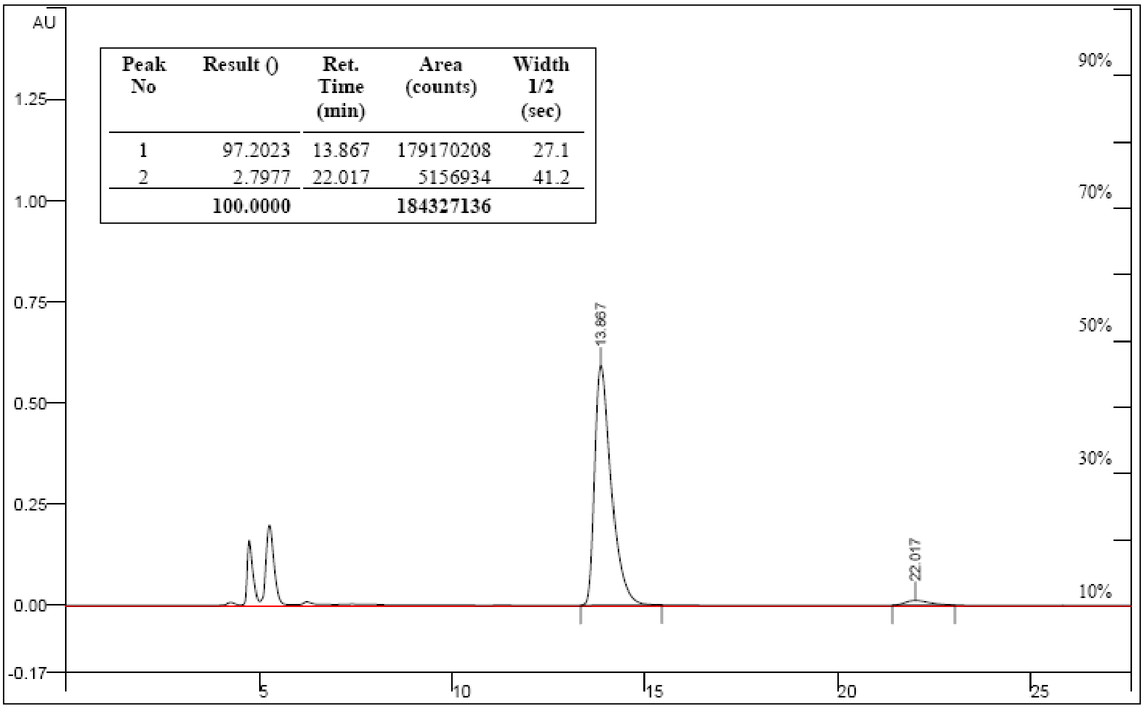

Peak No	Result ()	Ret. Time (min)	Area (counts)	Width 1/2 (sec)
1	97.2023	13.867	179170208	27.1
2	2.7977	22.017	5156934	41.2
	**100.0000**		**184327136**	

HPLC spectrum of the remaining alcohol (*R*)-**2a**

[Chiralcel OD-H, Hexane/IPA = 90/10, 0.7 ml/min, 220 nm]

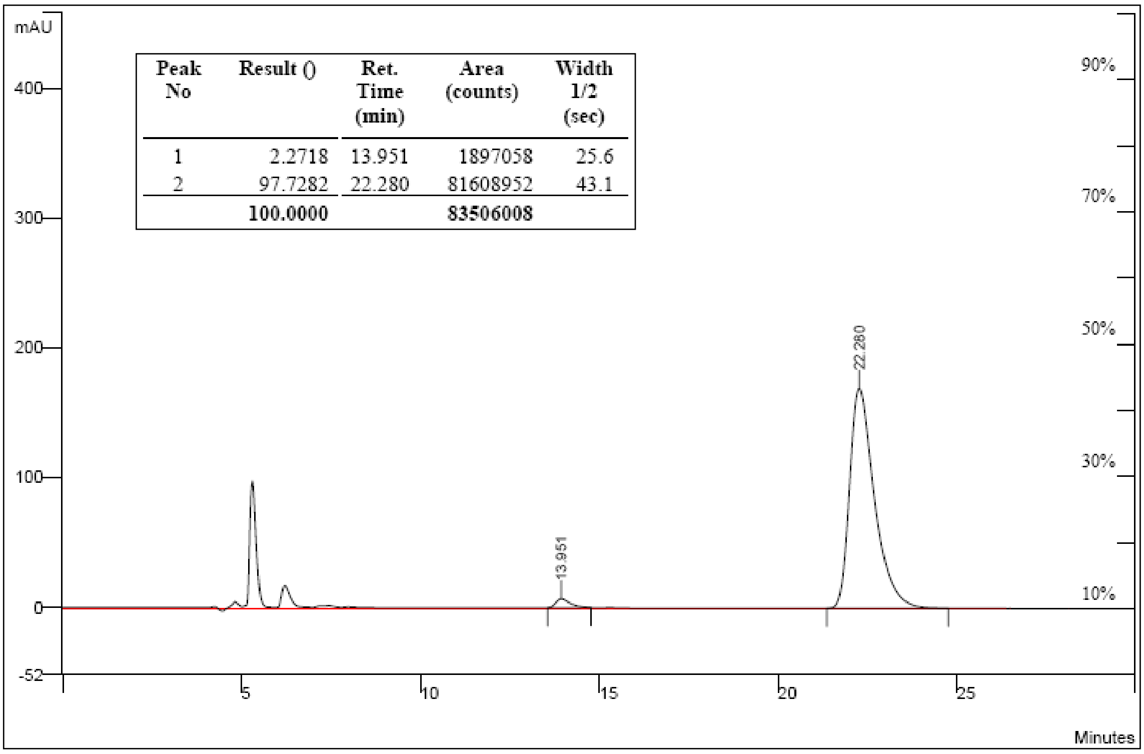

Peak No	Result ()	Ret. Time (min)	Area (counts)	Width 1/2 (sec)
1	2.2718	13.951	1897058	25.6
2	97.7282	22.280	81608952	43.1
	**100.0000**		**83506008**	

^{1}H NMR spectrum of the silylation reaction mixture of 2b after 54.5% conversion

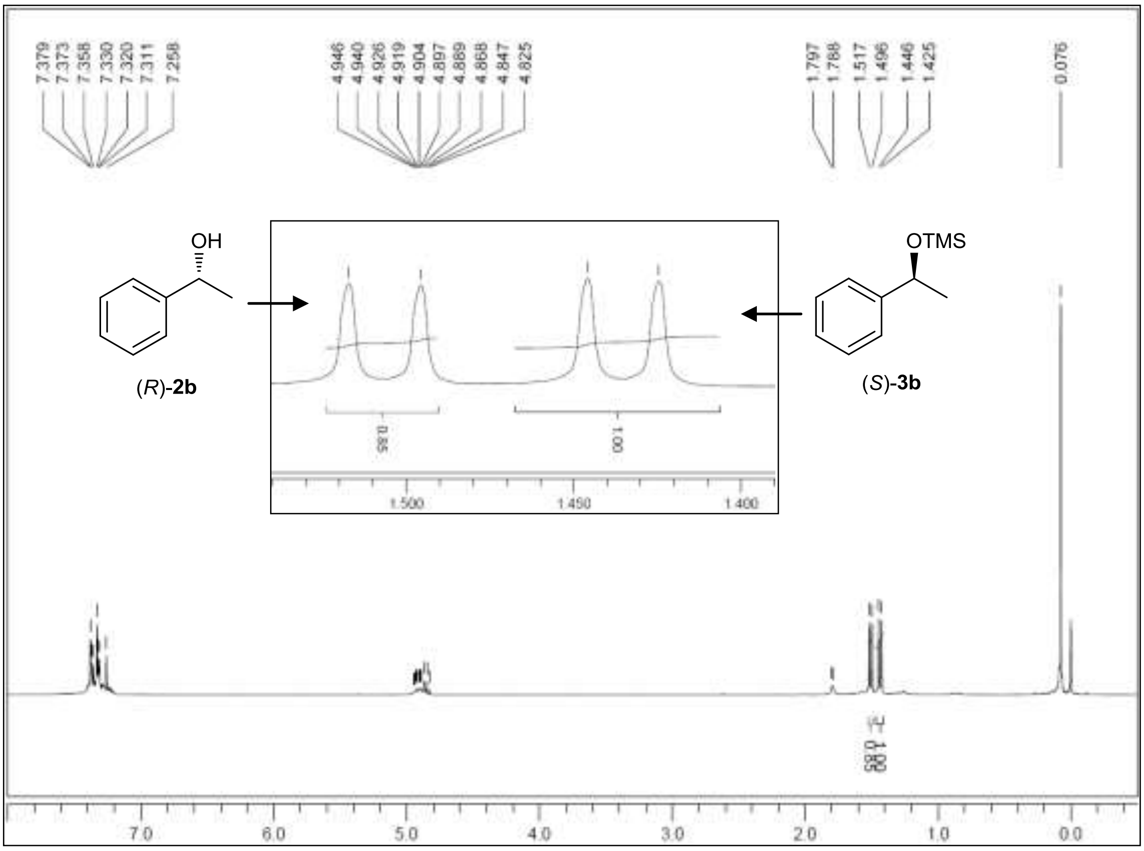

HPLC spectrum of *rac*-**2b** (Chiralcel OD-H, Hexane/IPA = 90/10, 0.5 ml/min, 220 nm)

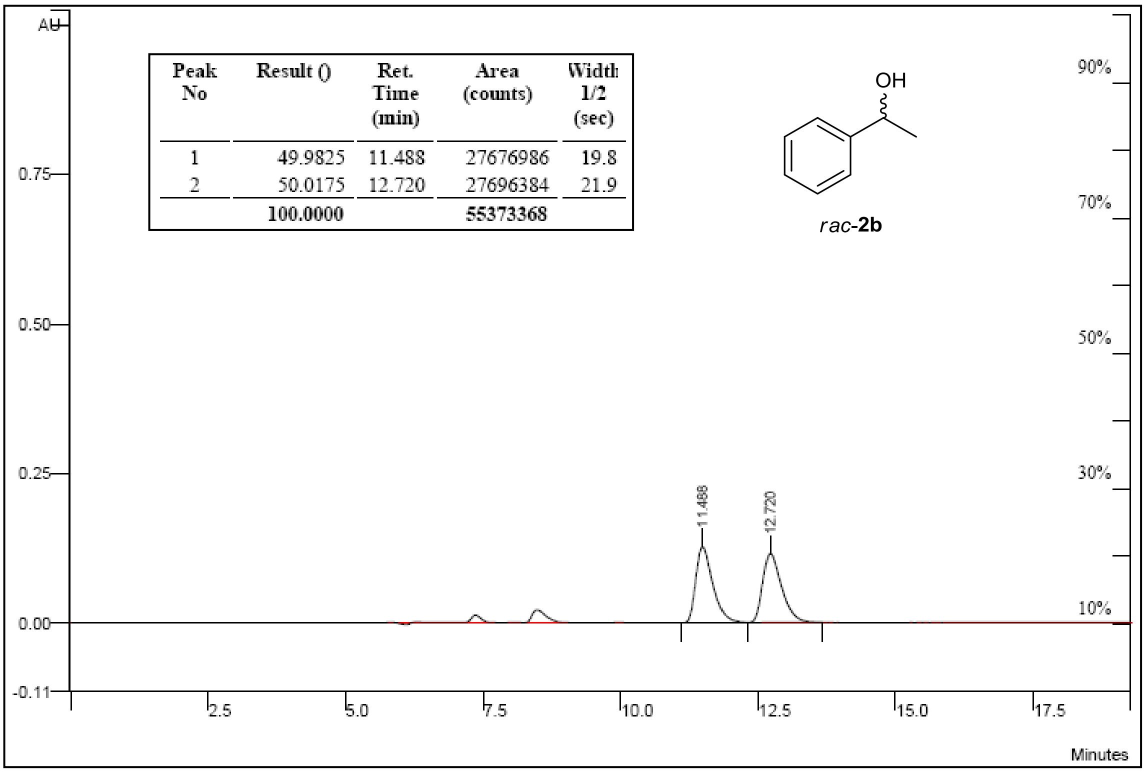

Peak No	Result ()	Ret. Time (min)	Area (counts)	Width 1/2 (sec)
1	49.9825	11.488	27676986	19.8
2	50.0175	12.720	27696384	21.9
	**100.0000**		**55373368**	

HPLC spectrum of the TMS-ether product (*S*)-**3b**

[Chiralcel OD-H, Hexane/IPA = 90/10, 0.5 ml/min, 220 nm]

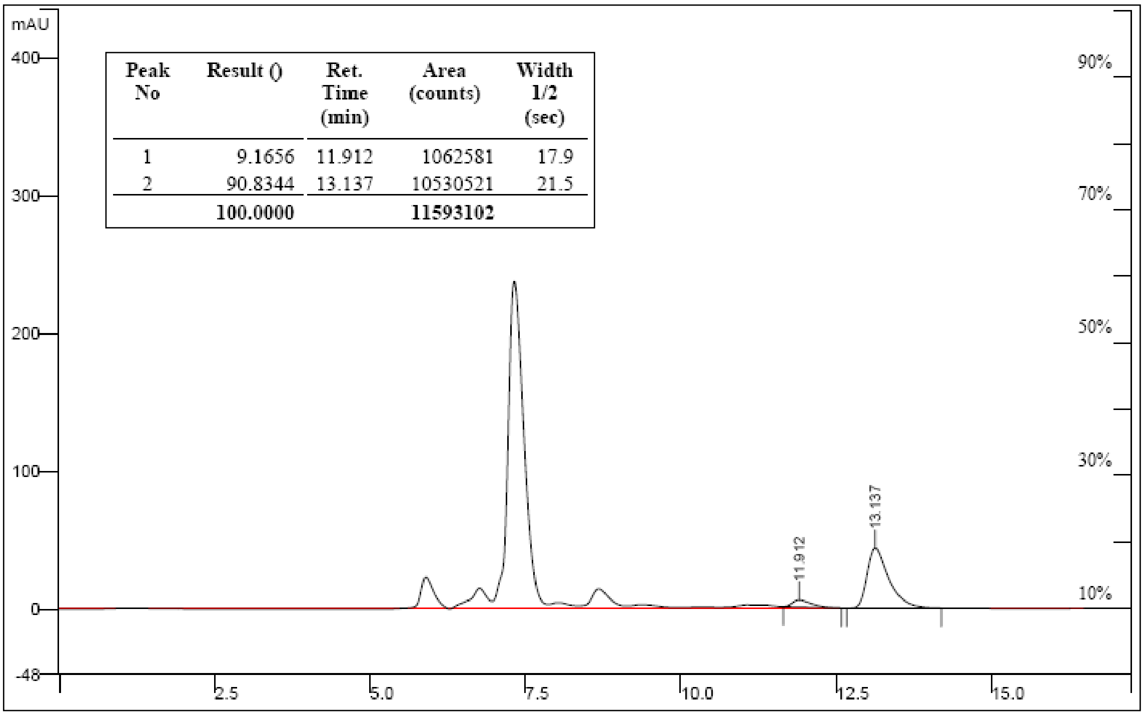

HPLC spectrum of the remaining alcohol (*R*)-**2b**

[Chiralcel OD-H, Hexane/IPA = 90/10, 0.5 ml/min, 220 nm]

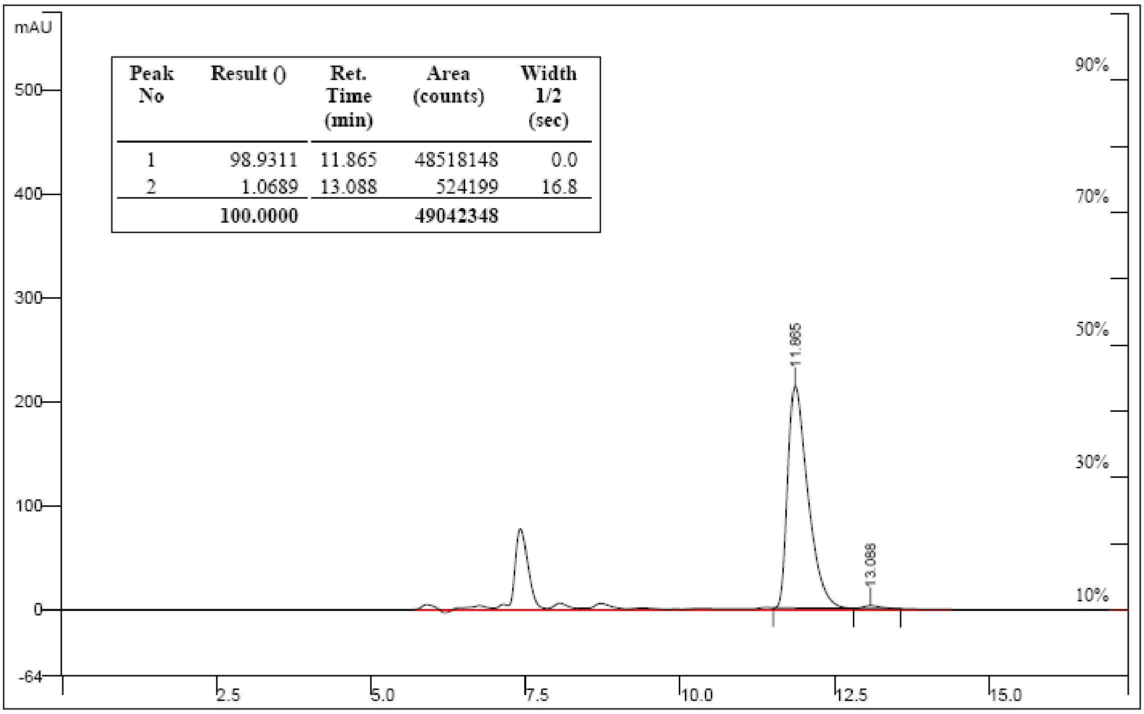

^{1}H NMR spectrum of the silylation reaction mixture of 2c after 55.8% conversion

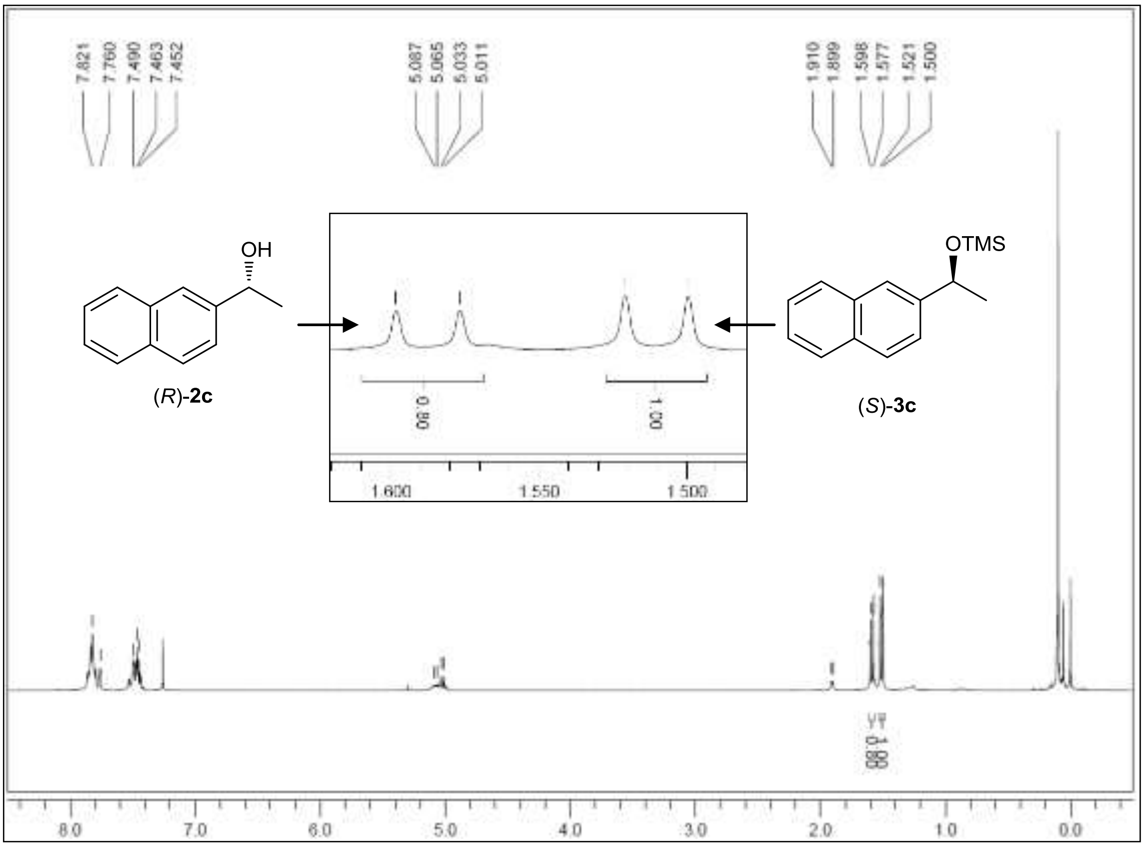

HPLC spectrum of *rac*-**2c** (Chiralcel OJ-H, Hexane/IPA = 95/5, 1.0 ml/min, 220 nm)

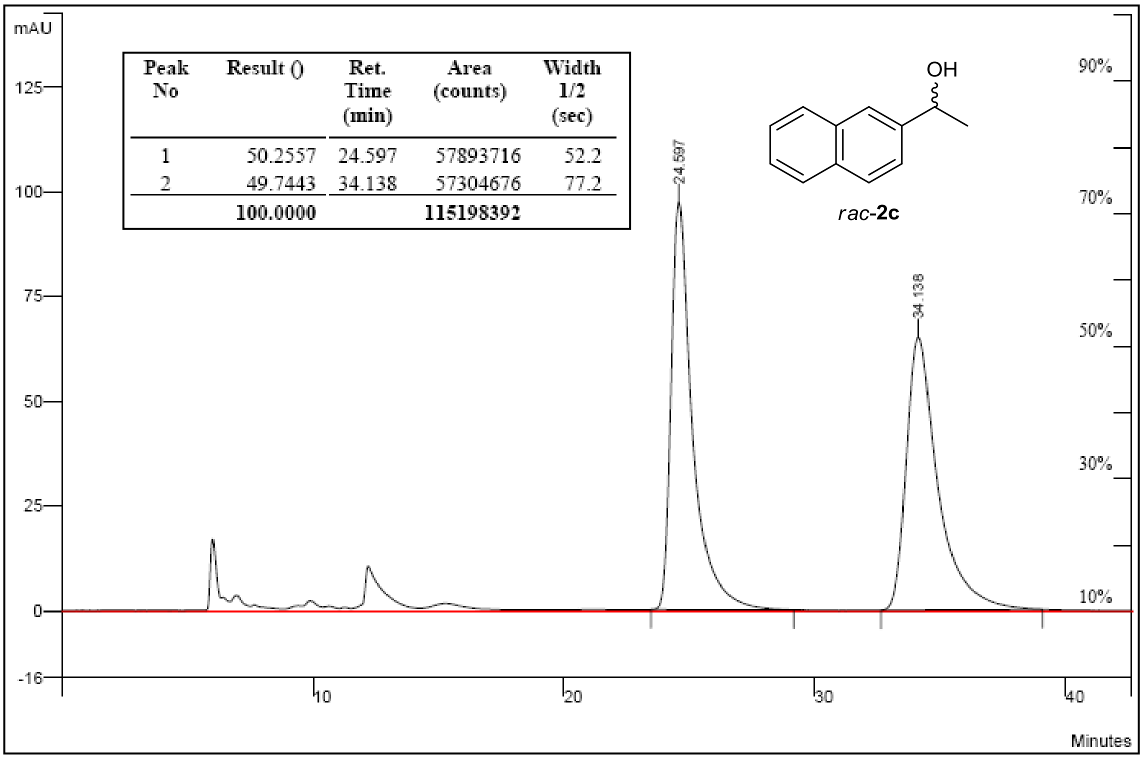

Peak No	Result ()	Ret. Time (min)	Area (counts)	Width 1/2 (sec)
1	50.2557	24.597	57893716	52.2
2	49.7443	34.138	57304676	77.2
	**100.0000**		**115198392**	

HPLC spectrum of the TMS-ether product (*S*)-**3c**

[Chiralcel OJ-H, Hexane/IPA = 95/5, 1.0 ml/min, 220 nm]

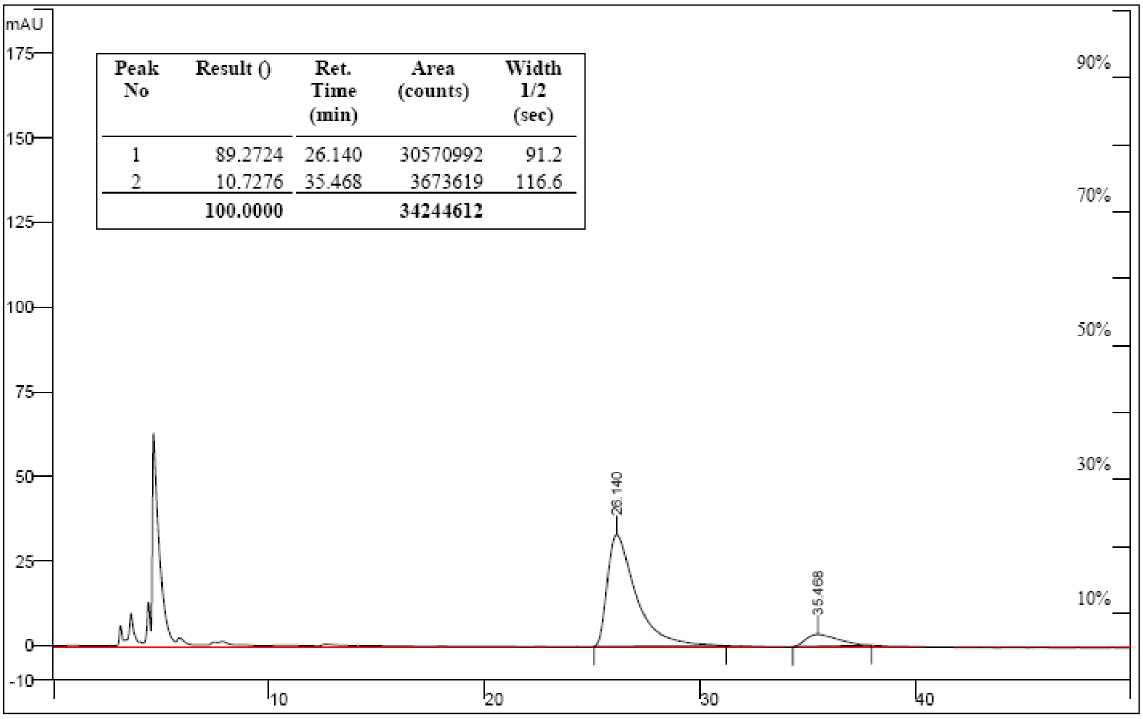

Peak No	Result ()	Ret. Time (min)	Area (counts)	Width 1/2 (sec)
1	89.2724	26.140	30570992	91.2
2	10.7276	35.468	3673619	116.6
	**100.0000**		**34244612**	

HPLC spectrum of the remaining alcohol (*R*)-**2c**

[Chiralcel OJ-H, Hexane/IPA = 95/5, 1.0 ml/min, 220 nm]

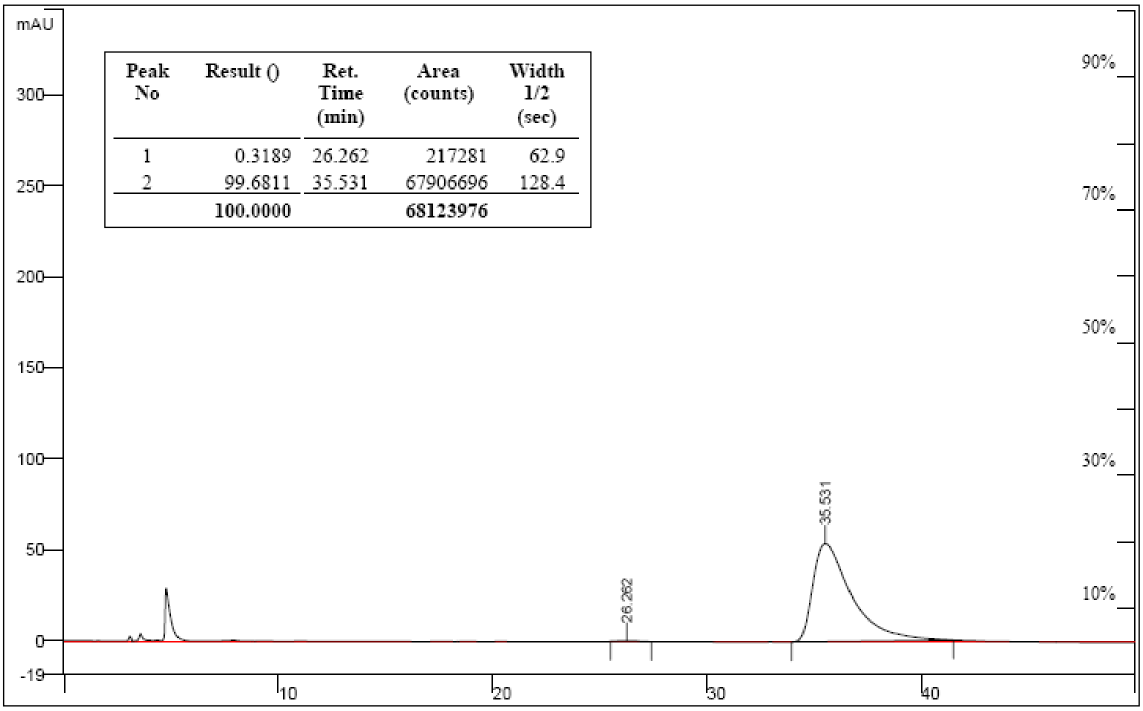

Peak No	Result ()	Ret. Time (min)	Area (counts)	Width 1/2 (sec)
1	0.3189	26.262	217281	62.9
2	99.6811	35.531	67906696	128.4
	**100.0000**		**68123976**	

^{1}H NMR spectrum of the silylation reaction mixture of 2d after 52.6% conversion

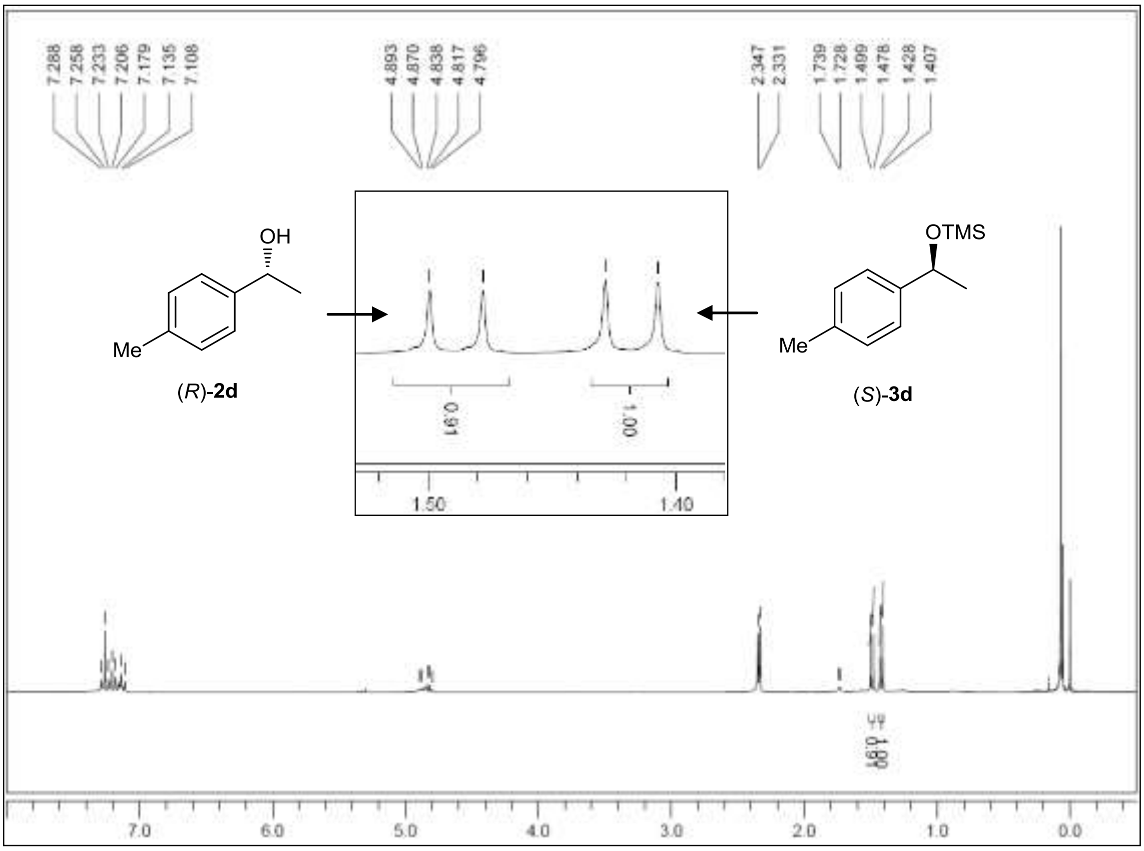

HPLC spectrum of *rac*-**2d** (Chiralcel OB-H, Hexane/IPA = 90/10, 0.5 ml/min, 220 nm)

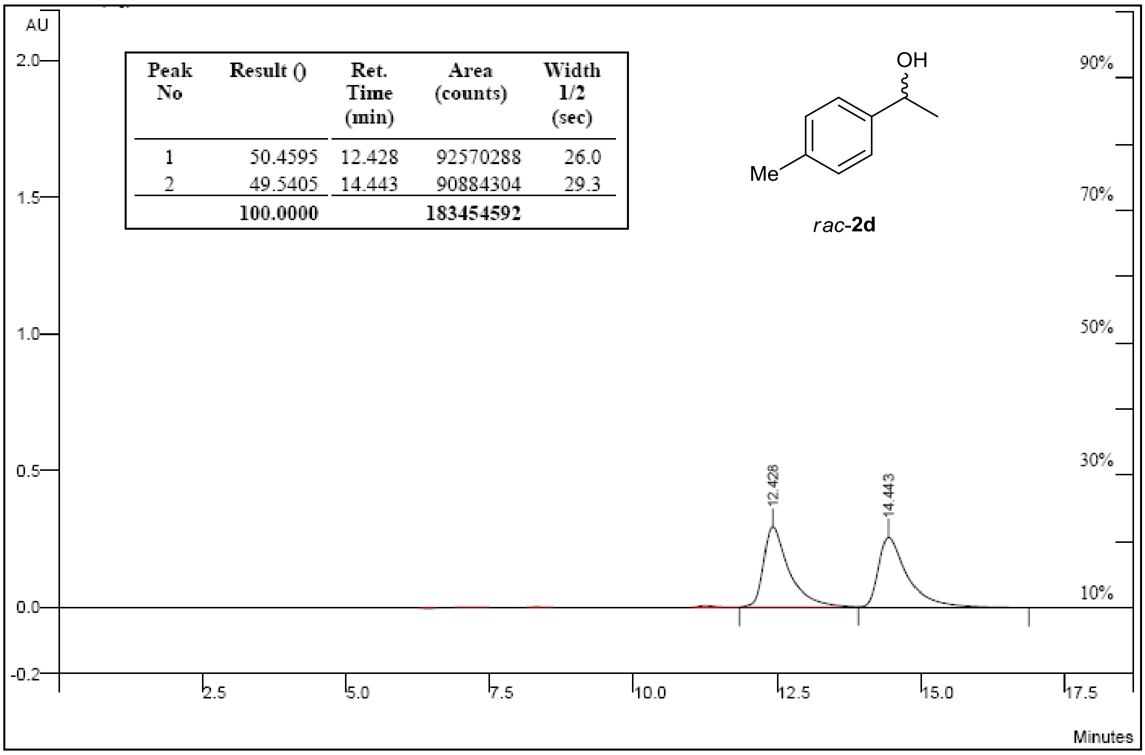

Peak No	Result ()	Ret. Time (min)	Area (counts)	Width 1/2 (sec)
1	50.4595	12.428	92570288	26.0
2	49.5405	14.443	90884304	29.3
	100.0000		183454592	

HPLC spectrum of the TMS-ether product (*S*)-**3d**

[Chiralcel OB-H, Hexane/IPA = 90/10, 0.5 ml/min, 220 nm]

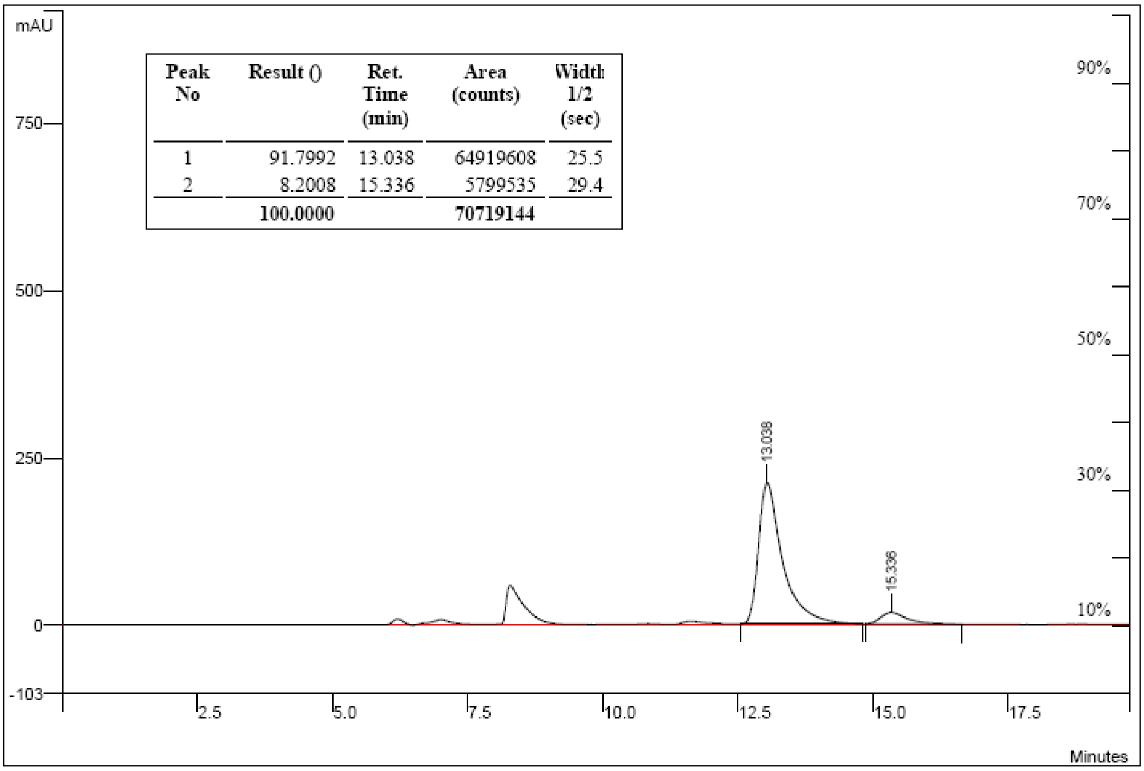

Peak No	Result ()	Ret. Time (min)	Area (counts)	Width 1/2 (sec)
1	91.7992	13.038	64919608	25.5
2	8.2008	15.336	5799535	29.4
	**100.0000**		**70719144**	

HPLC spectrum of the remaining alcohol (*R*)-**2d**

[Chiralcel OB-H, Hexane/IPA = 90/10, 0.5 ml/min, 220 nm]

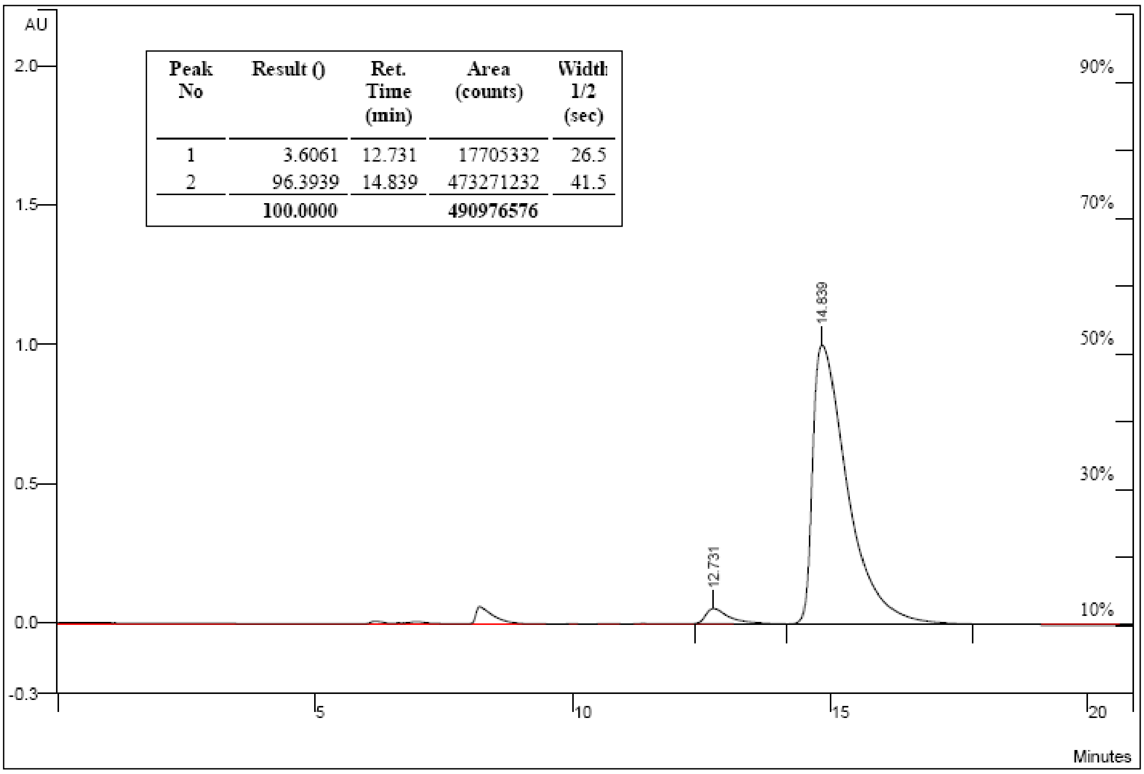

Peak No	Result ()	Ret. Time (min)	Area (counts)	Width 1/2 (sec)
1	3.6061	12.731	17705332	26.5
2	96.3939	14.839	473271232	41.5
	**100.0000**		**490976576**	

^{1}H NMR spectrum of the silylation reaction mixture of 2e after 53.5% conversion

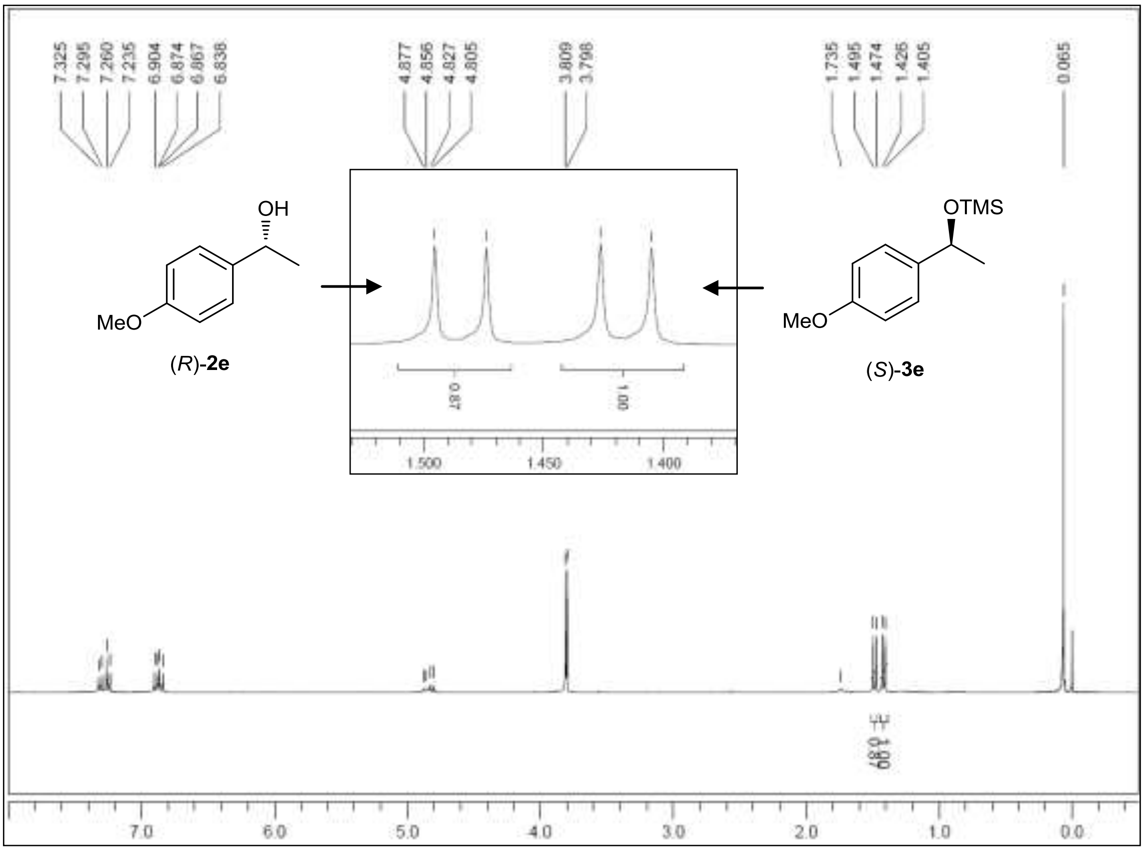

HPLC spectrum of *rac*-**2e** (Chiralcel OB-H, Hexane/IPA = 98/2, 0.5 ml/min, 220 nm)

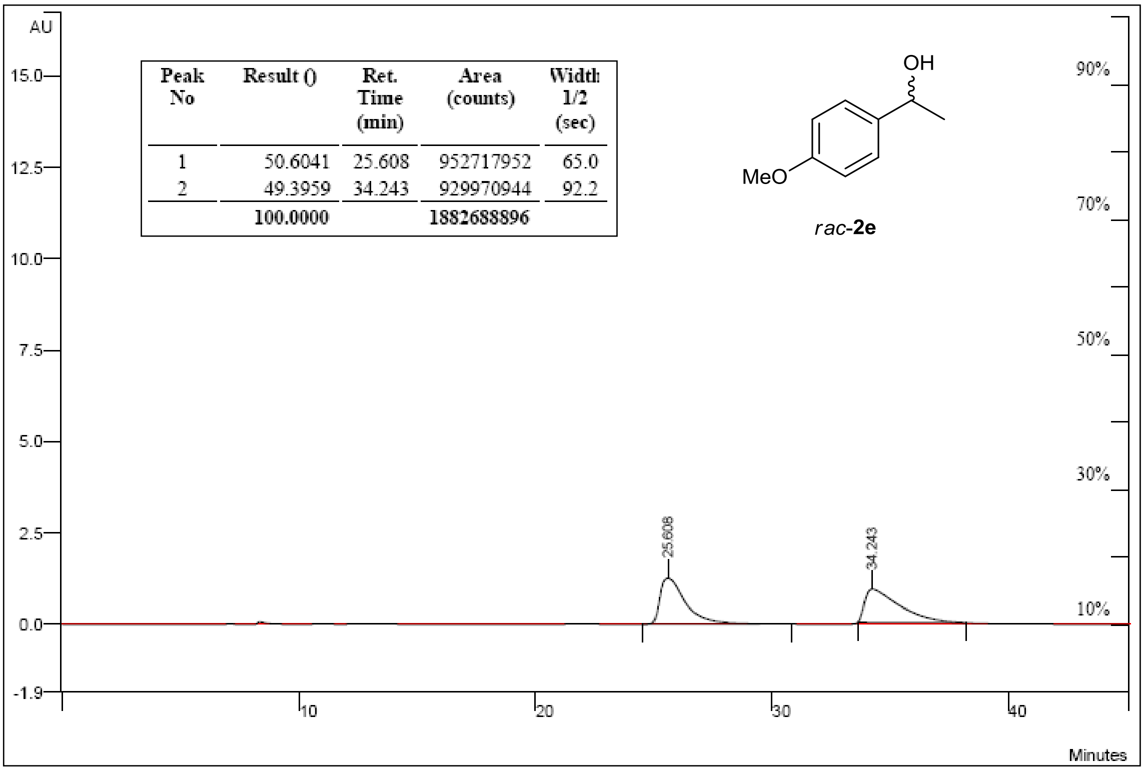

Peak No	Result ()	Ret. Time (min)	Area (counts)	Width 1/2 (sec)
1	50.6041	25.608	952717952	65.0
2	49.3959	34.243	929970944	92.2
	**100.0000**		**1882688896**	

HPLC spectrum of the TMS-ether product (*S*)-**3e**

[Chiralcel OB-H, Hexane/IPA = 98/2, 0.5 ml/min, 220 nm]

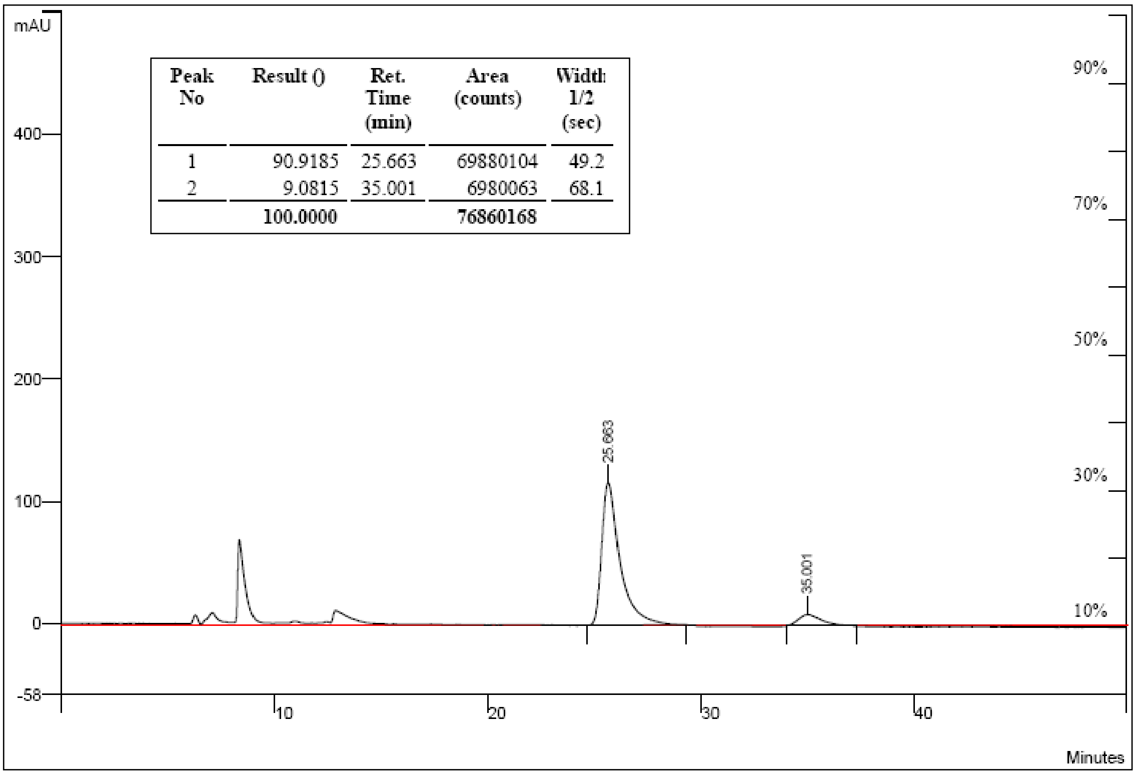

Peak No	Result ()	Ret. Time (min)	Area (counts)	Width 1/2 (sec)
1	90.9185	25.663	69880104	49.2
2	9.0815	35.001	6980063	68.1
	100.0000		76860168	

HPLC spectrum of the remaining alcohol (*R*)-**2e**

[Chiralcel OB-H, Hexane/IPA = 98/2, 0.5 ml/min, 220 nm]

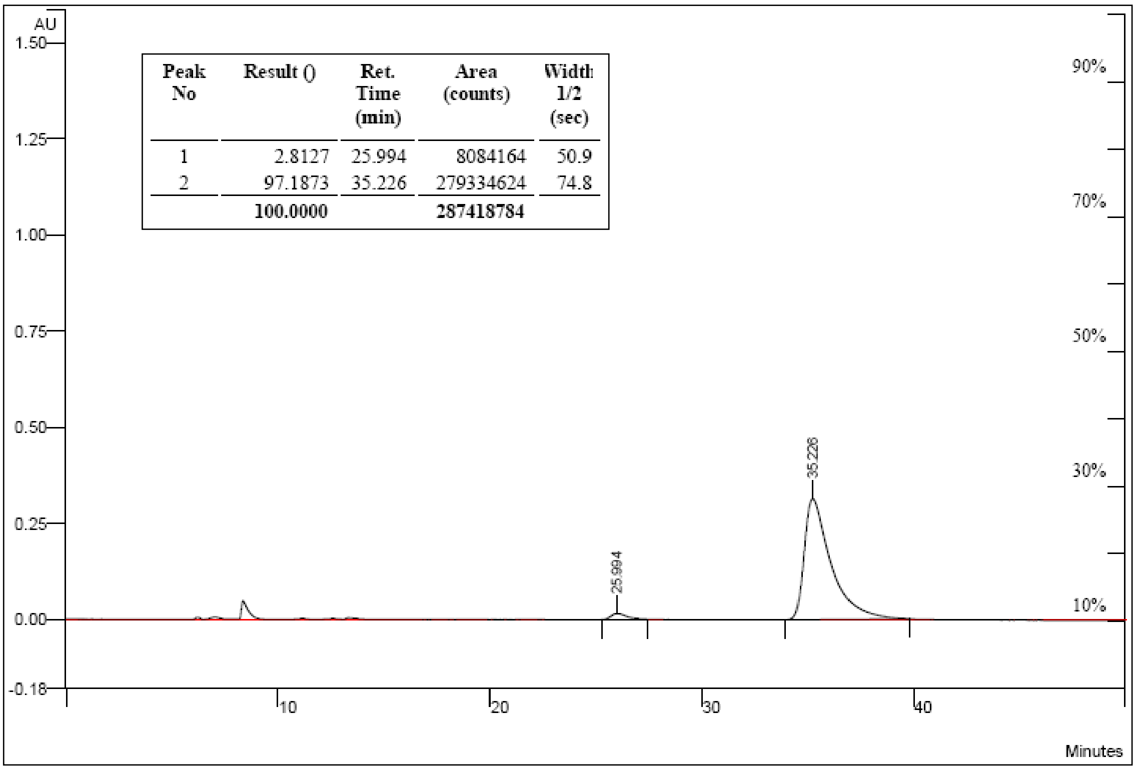

Peak No	Result ()	Ret. Time (min)	Area (counts)	Width 1/2 (sec)
1	2.8127	25.994	8084164	50.9
2	97.1873	35.226	279334624	74.8
	100.0000		287418784	

^{1}H NMR spectrum of the silylation reaction mixture of 2f after 51.4% conversion

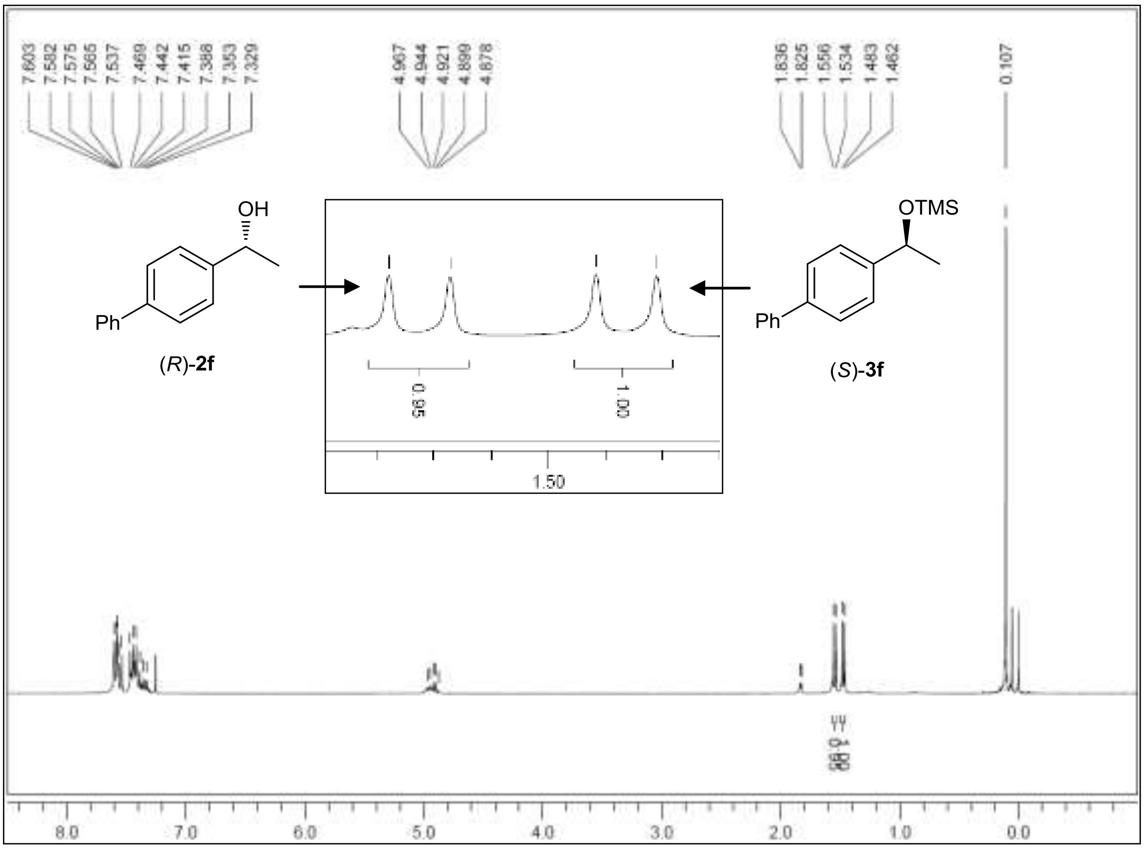

HPLC spectrum of *rac*-**2f** (Chiralcel OD-H, Hexane/IPA = 95/5, 0.8 ml/min, 220 nm)

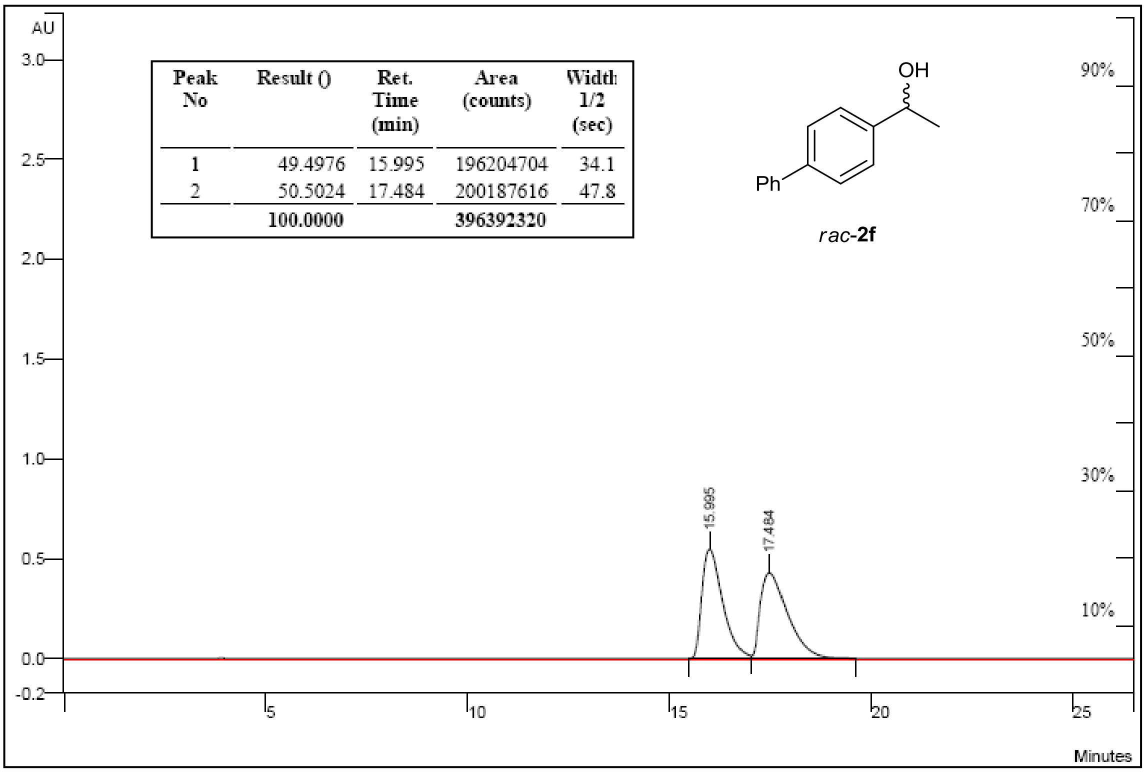

Peak No	Result ()	Ret. Time (min)	Area (counts)	Width 1/2 (sec)
1	49.4976	15.995	196204704	34.1
2	50.5024	17.484	200187616	47.8
	100.0000		396392320	

HPLC spectrum of the TMS-ether product (*S*)-**3f**

[Chiralcel OD-H, Hexane/IPA = 95/5, 0.8 ml/min, 220 nm]

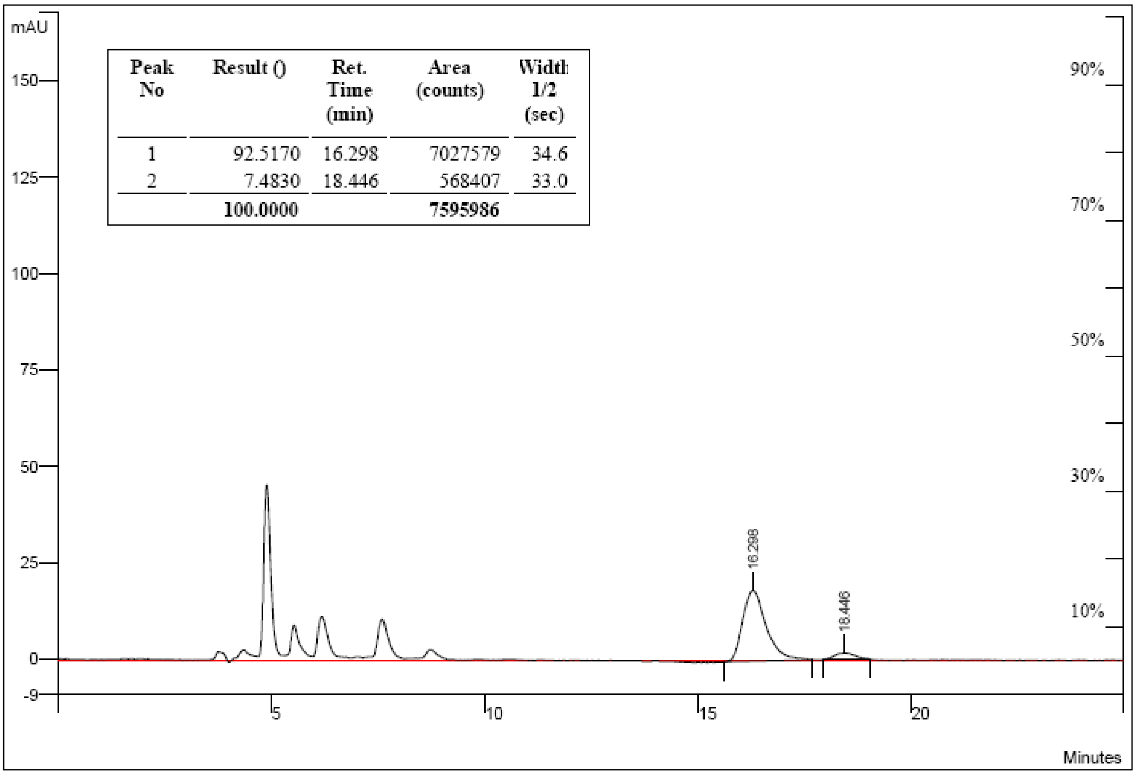

Peak No	Result ()	Ret. Time (min)	Area (counts)	Width 1/2 (sec)
1	92.5170	16.298	7027579	34.6
2	7.4830	18.446	568407	33.0
	**100.0000**		**7595986**	

HPLC spectrum of the remaining alcohol (*R*)-**2f**

[Chiralcel OD-H, Hexane/IPA = 95/5, 0.8 ml/min, 220 nm]

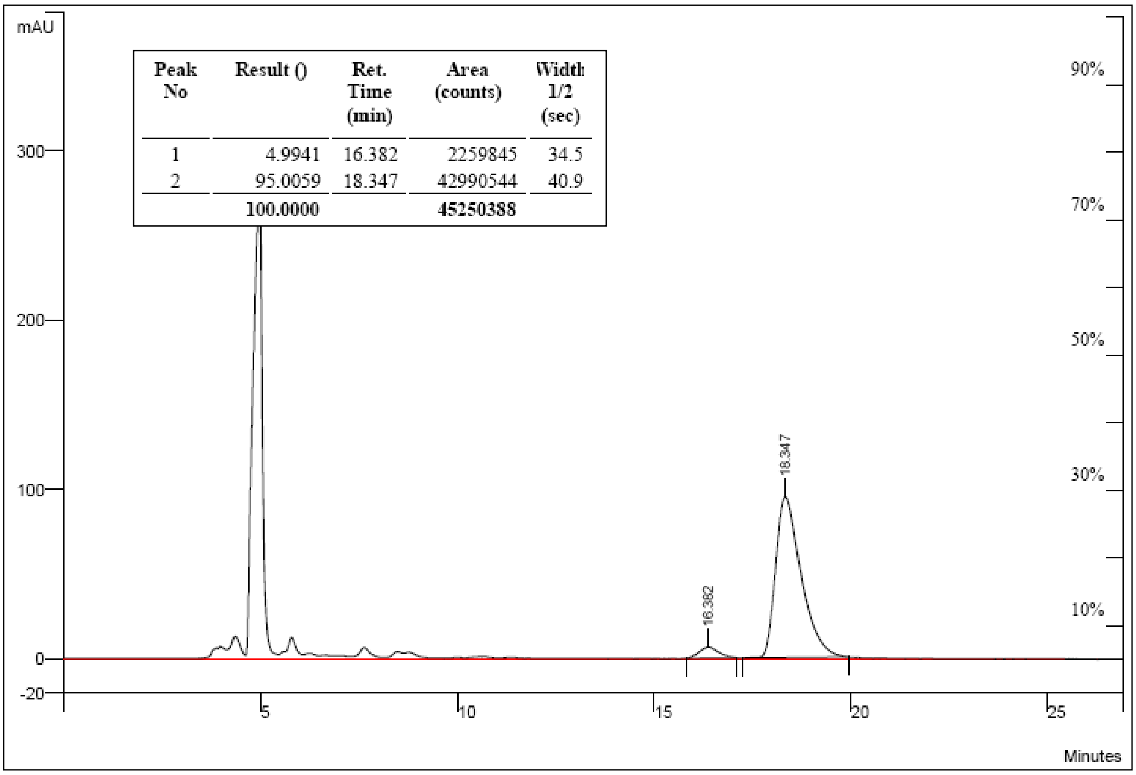

Peak No	Result ()	Ret. Time (min)	Area (counts)	Width 1/2 (sec)
1	4.9941	16.382	2259845	34.5
2	95.0059	18.347	42990544	40.9
	**100.0000**		**45250388**	

^{1}H NMR spectrum of the silylation reaction mixture of 2g after 53.7% conversion

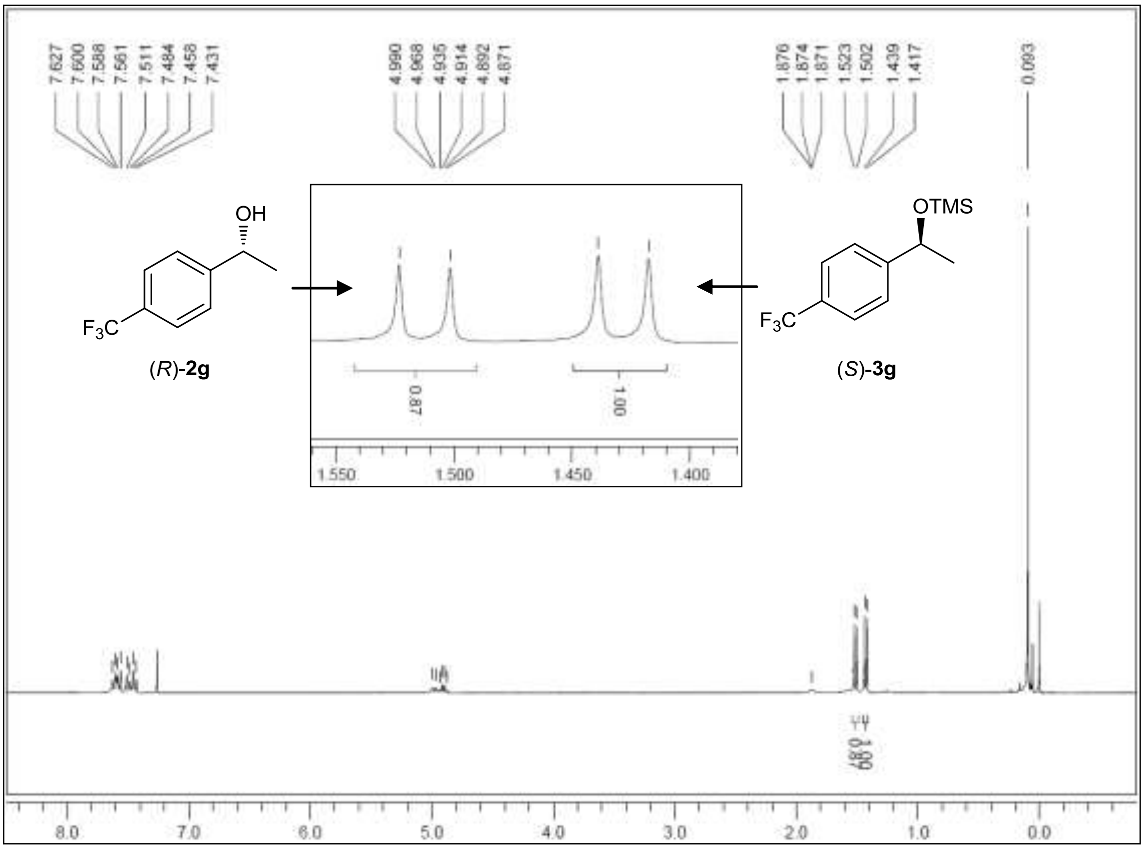

HPLC spectrum of *rac*-**2g** (Chiralcel OJ-H, Hexane/IPA = 99.8/0.2, 1.0 ml/min, 220 nm)

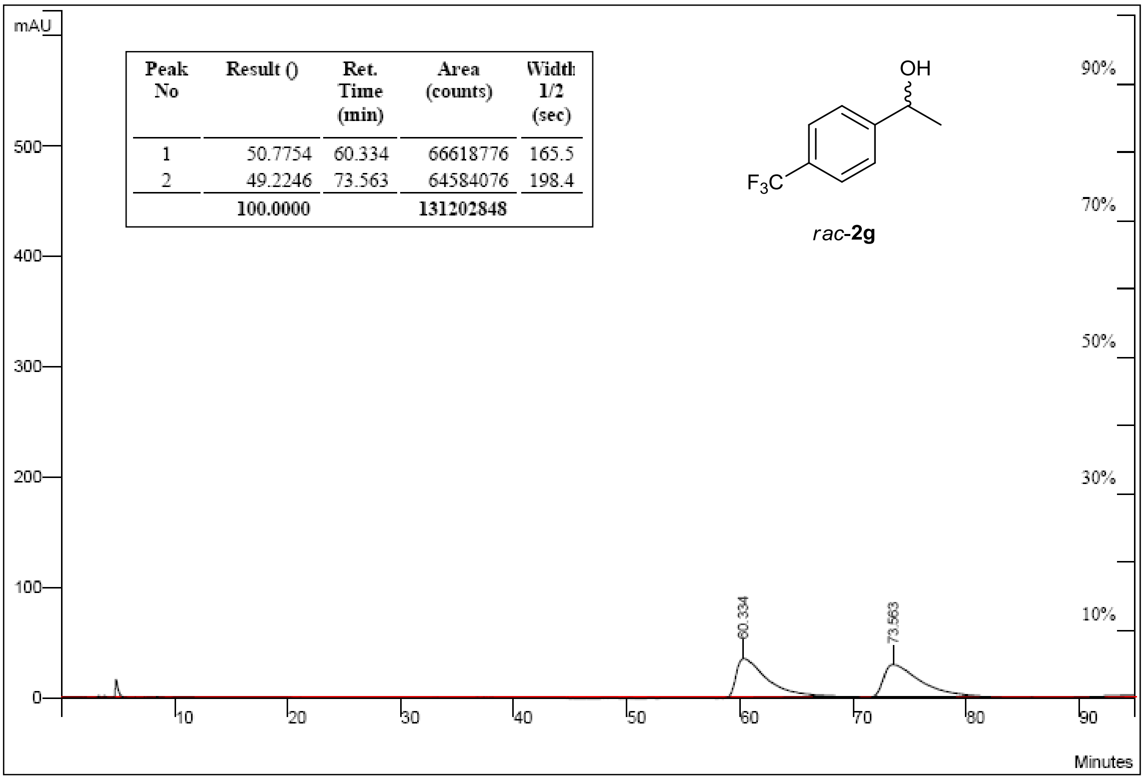

Peak No	Result ()	Ret. Time (min)	Area (counts)	Width 1/2 (sec)
1	50.7754	60.334	66618776	165.5
2	49.2246	73.563	64584076	198.4
	**100.0000**		**131202848**	

HPLC spectrum of the TMS-ether product (*S*)-**3g**

[Chiralcel OJ-H, Hexane/IPA = 99.8/0.2, 1.0 ml/min, 220 nm]

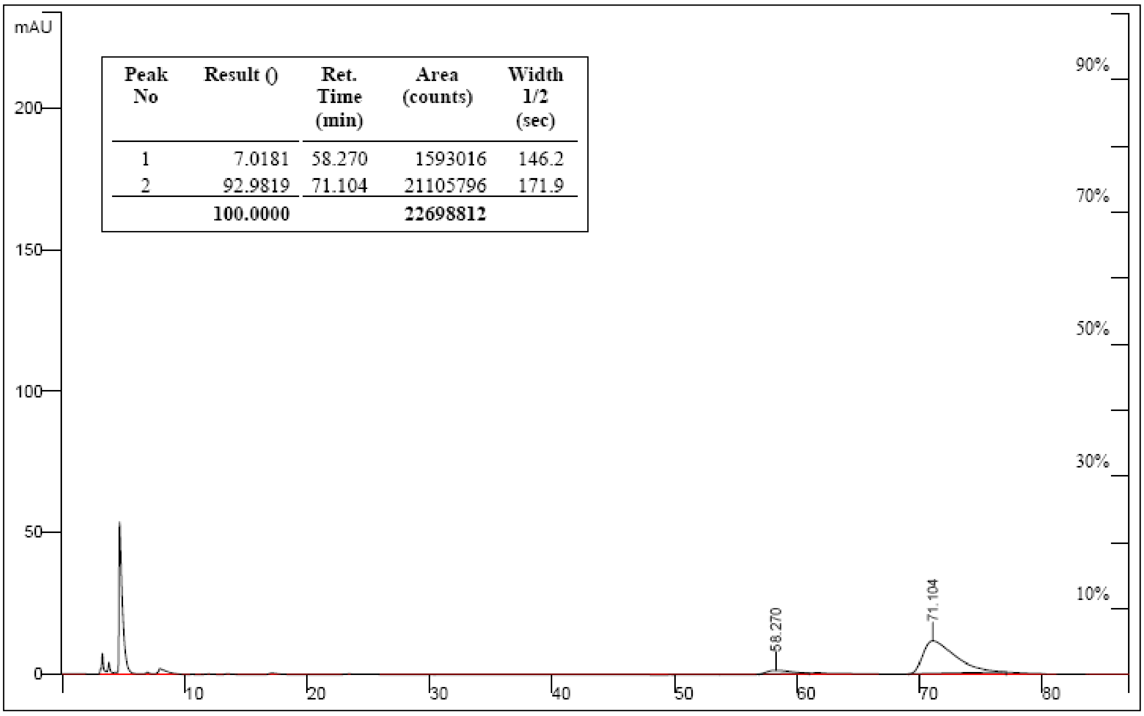

Peak No	Result ()	Ret. Time (min)	Area (counts)	Width 1/2 (sec)
1	7.0181	58.270	1593016	146.2
2	92.9819	71.104	21105796	171.9
	**100.0000**		**22698812**	

HPLC spectrum of the remaining alcohol (*R*)-**2g**

[Chiralcel OJ-H, Hexane/IPA = 99.8/0.2, 1.0 ml/min, 220 nm]

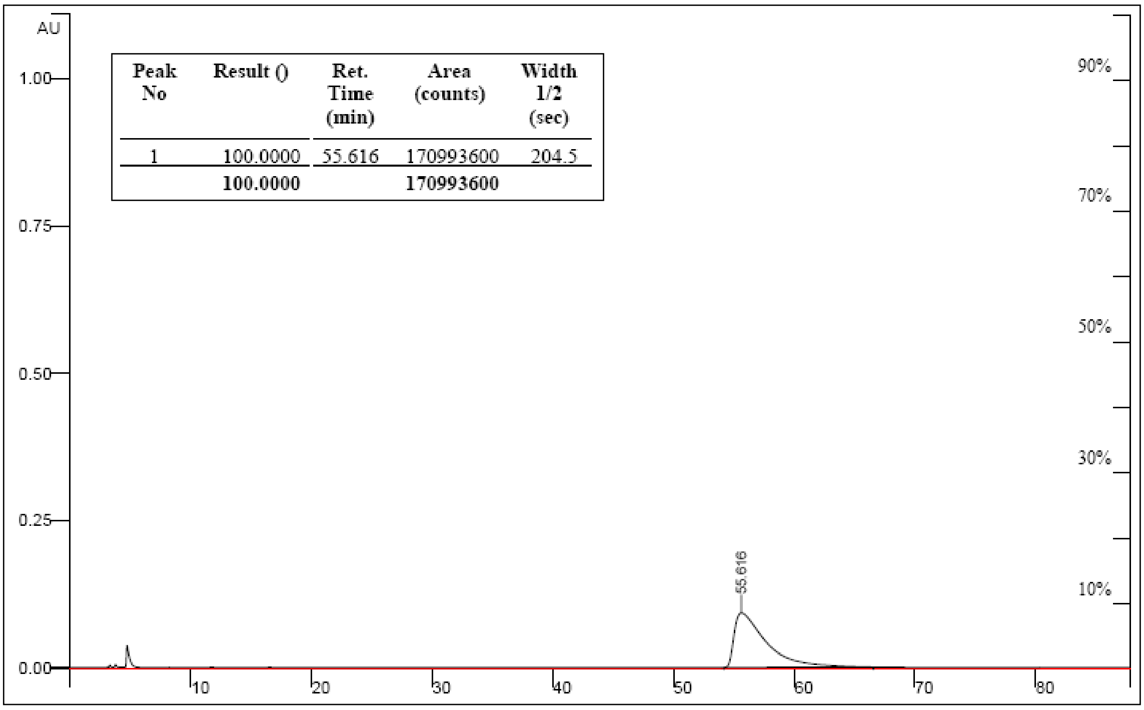

Peak No	Result ()	Ret. Time (min)	Area (counts)	Width 1/2 (sec)
1	100.0000	55.616	170993600	204.5
	**100.0000**		**170993600**	

^{1}H NMR spectrum of the silylation reaction mixture of 2h after 54.8% conversion

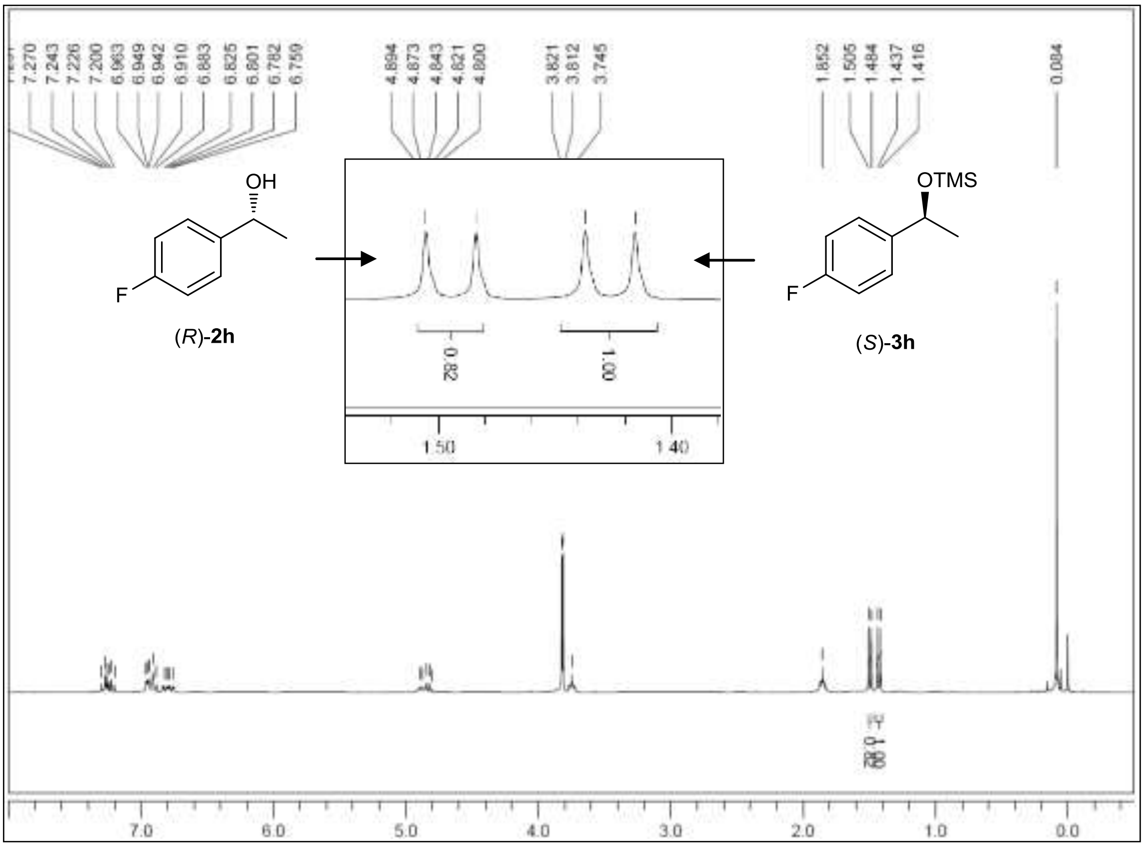

HPLC spectrum of *rac*-**2h** (Chiralcel OB-H, Hexane/IPA = 98/2, 0.5 ml/min, 220 nm)

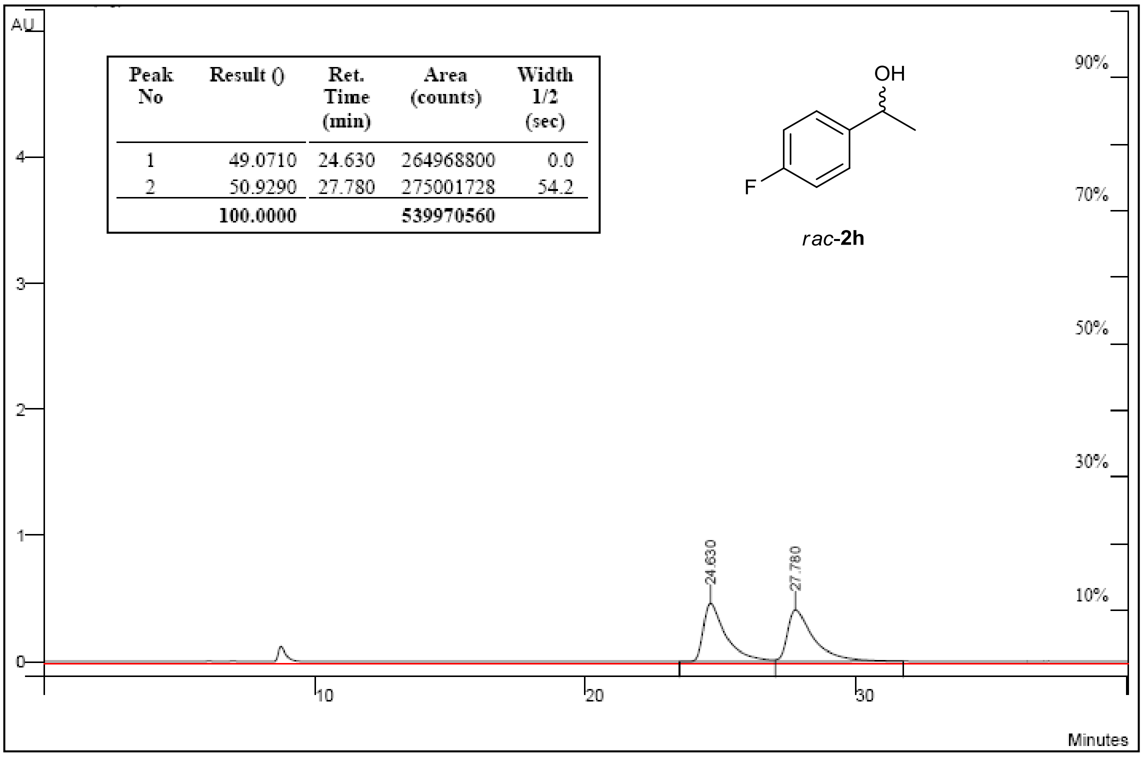

Peak No	Result ()	Ret. Time (min)	Area (counts)	Width 1/2 (sec)
1	49.0710	24.630	264968800	0.0
2	50.9290	27.780	275001728	54.2
	**100.0000**		**539970560**	

HPLC spectrum of the TMS-ether product (*S*)-**3h**

[Chiralcel OB-H, Hexane/IPA = 98/2, 0.5 ml/min, 220 nm]

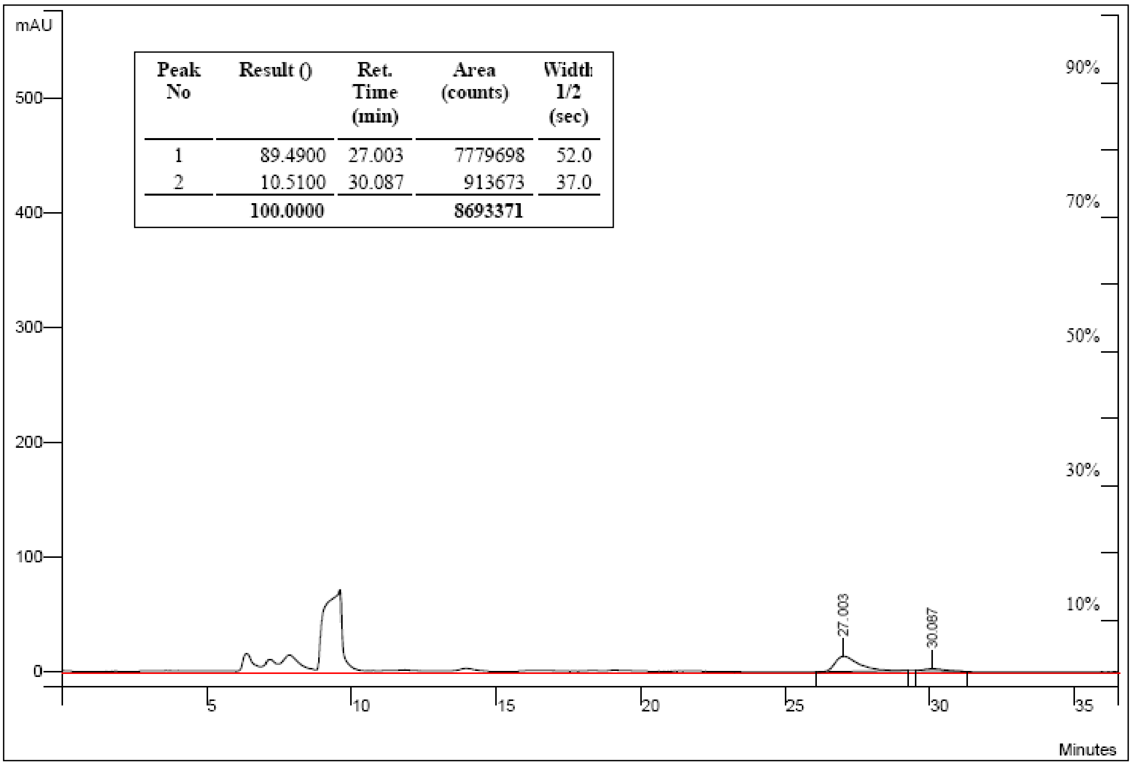

Peak No	Result ()	Ret. Time (min)	Area (counts)	Width 1/2 (sec)
1	89.4900	27.003	7779698	52.0
2	10.5100	30.087	913673	37.0
	**100.0000**		**8693371**	

HPLC spectrum of the remaining alcohol (*R*)-**2h**

[Chiralcel OB-H, Hexane/IPA = 98/2, 0.5 ml/min, 220 nm]

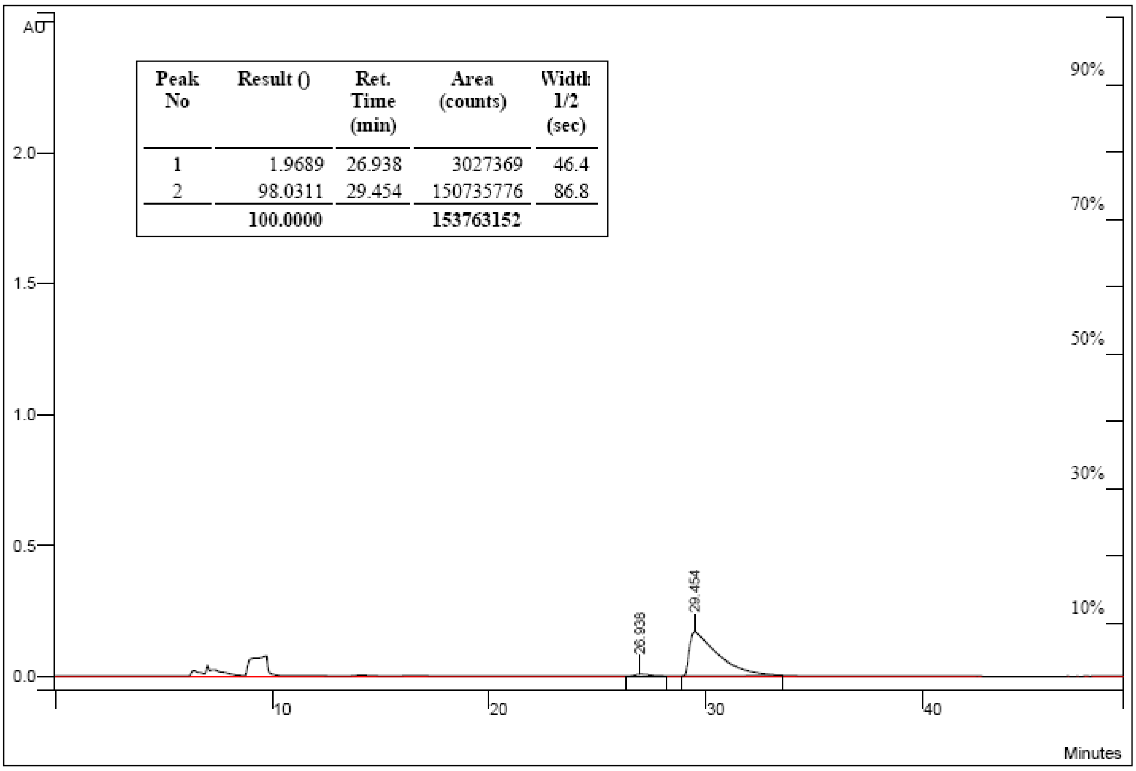

Peak No	Result ()	Ret. Time (min)	Area (counts)	Width 1/2 (sec)
1	1.9689	26.938	3027369	46.4
2	98.0311	29.454	150735776	86.8
	**100.0000**		**153763152**	

^{1}H NMR spectrum of the silylation reaction mixture of 2i after 55.7% conversion

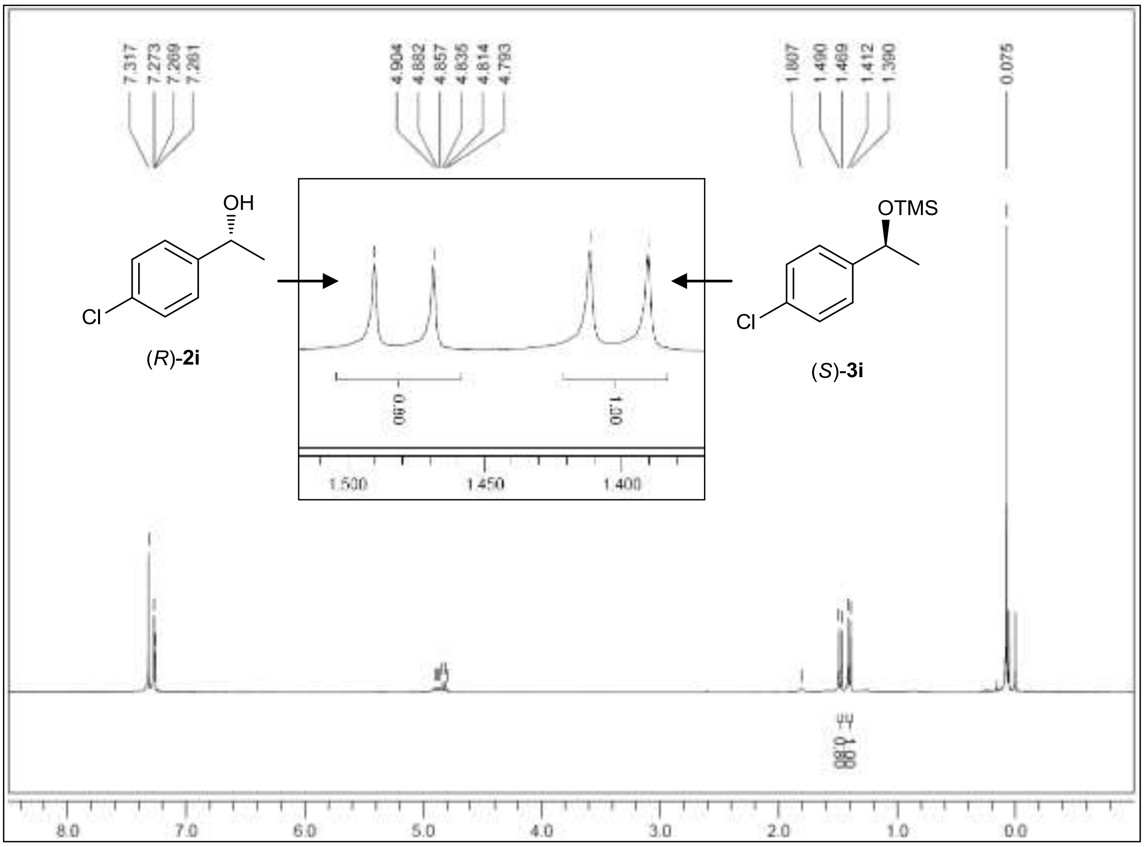

HPLC spectrum of *rac*-**2i** (Chiralcel OB-H, Hexane/IPA = 98/2, 0.5 ml/min, 220 nm)

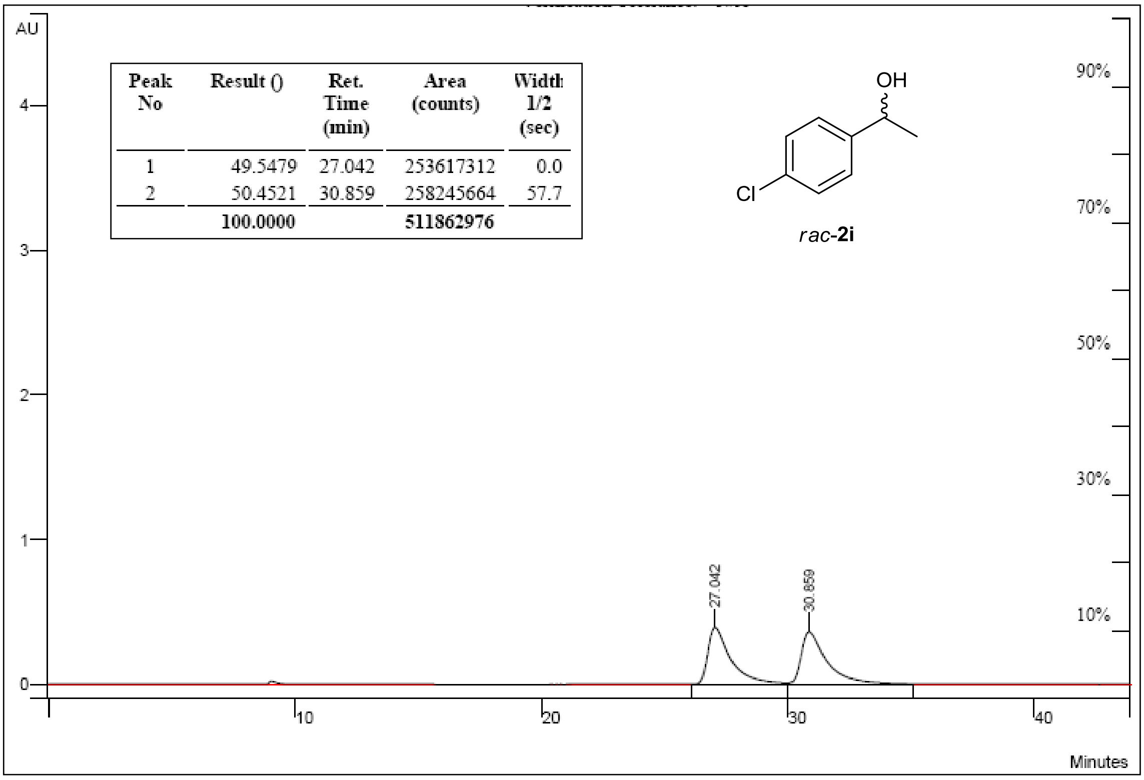

Peak No	Result ()	Ret. Time (min)	Area (counts)	Width 1/2 (sec)
1	49.5479	27.042	253617312	0.0
2	50.4521	30.859	258245664	57.7
	**100.0000**		**511862976**	

HPLC spectrum of the TMS-ether product (*S*)-**3i**

[Chiralcel OB-H, Hexane/IPA = 98/2, 0.5 ml/min, 220 nm]

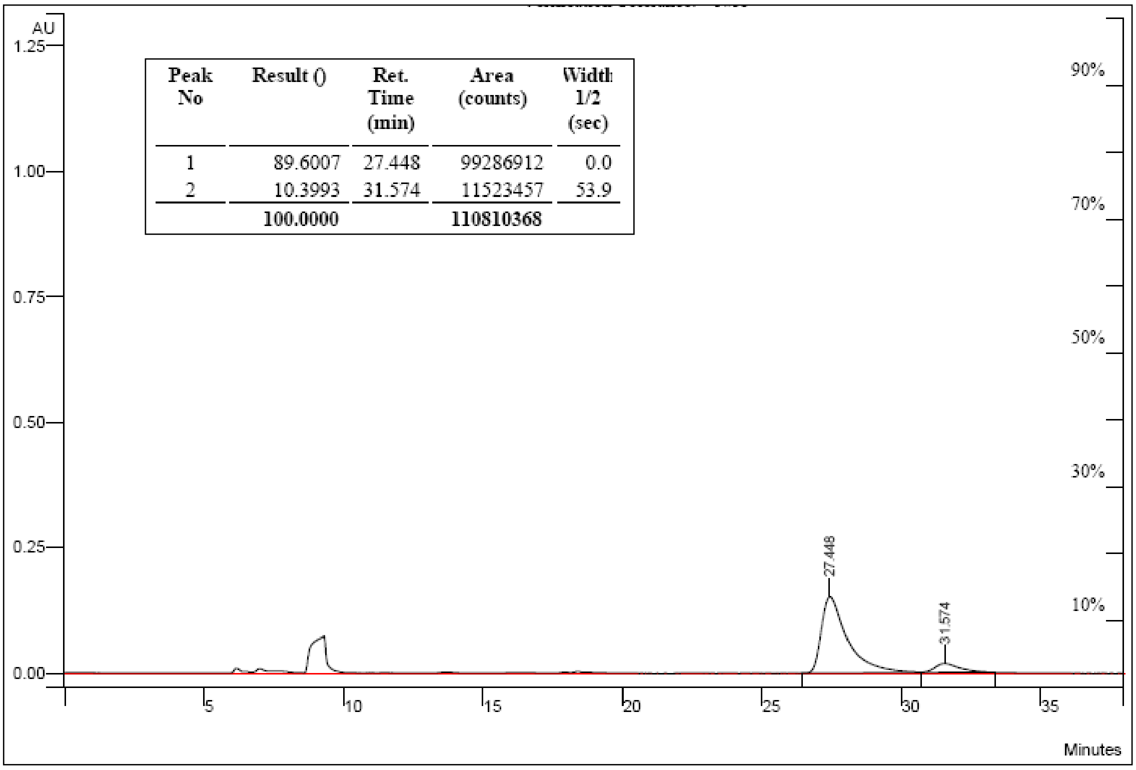

Peak No	Result ()	Ret. Time (min)	Area (counts)	Width 1/2 (sec)
1	89.6007	27.448	99286912	0.0
2	10.3993	31.574	11523457	53.9
	**100.0000**		**110810368**	

HPLC spectrum of the remaining alcohol (*R*)-**2i**

[Chiralcel OB-H, Hexane/IPA = 98/2, 0.5 ml/min, 220 nm]

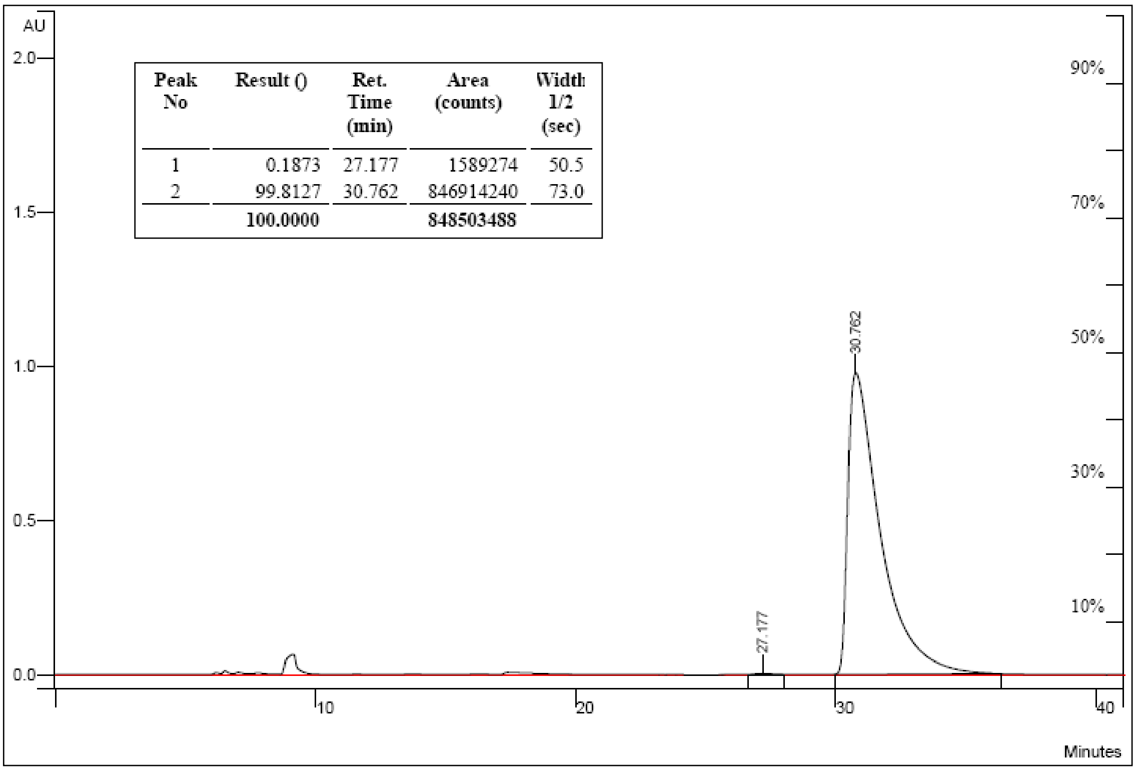

Peak No	Result ()	Ret. Time (min)	Area (counts)	Width 1/2 (sec)
1	0.1873	27.177	1589274	50.5
2	99.8127	30.762	846914240	73.0
	**100.0000**		**848503488**	

^{1}H NMR spectrum of the silylation reaction mixture of 2j after 57.5% conversion

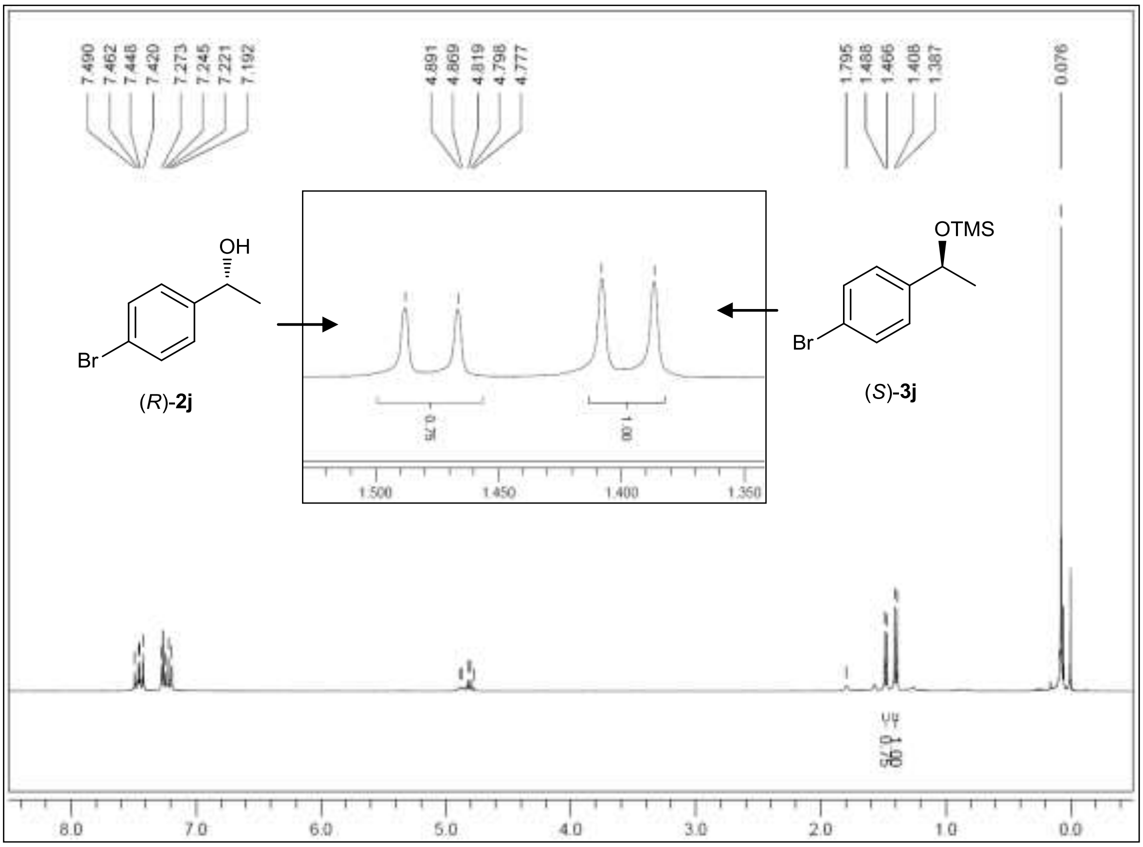

HPLC spectrum of *rac*-**2j** (Chiralcel OD-H, Hexane/IPA = 98/2, 0.5 ml/min, 220 nm)

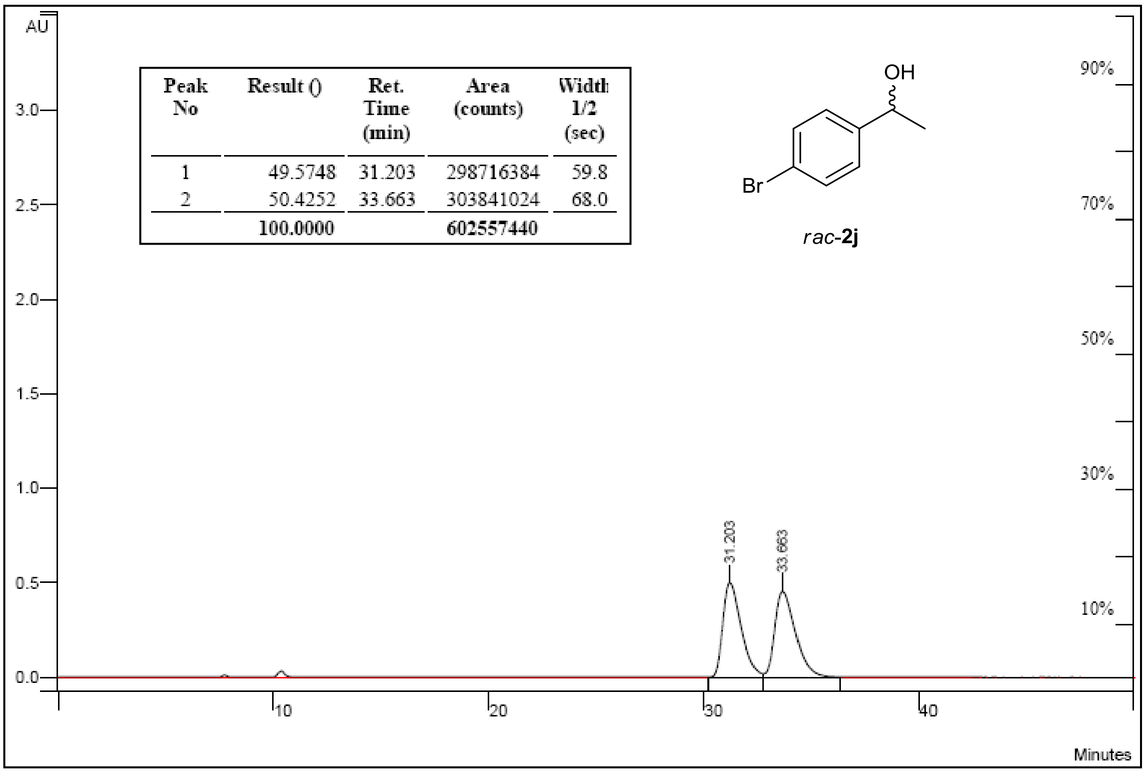

Peak No	Result ()	Ret. Time (min)	Area (counts)	Width 1/2 (sec)
1	49.5748	31.203	298716384	59.8
2	50.4252	33.663	303841024	68.0
	100.0000		602557440	

HPLC spectrum of the TMS-ether product (*S*)-**3j**

[Chiralcel OD-H, Hexane/IPA = 98/2, 0.5 ml/min, 220 nm]

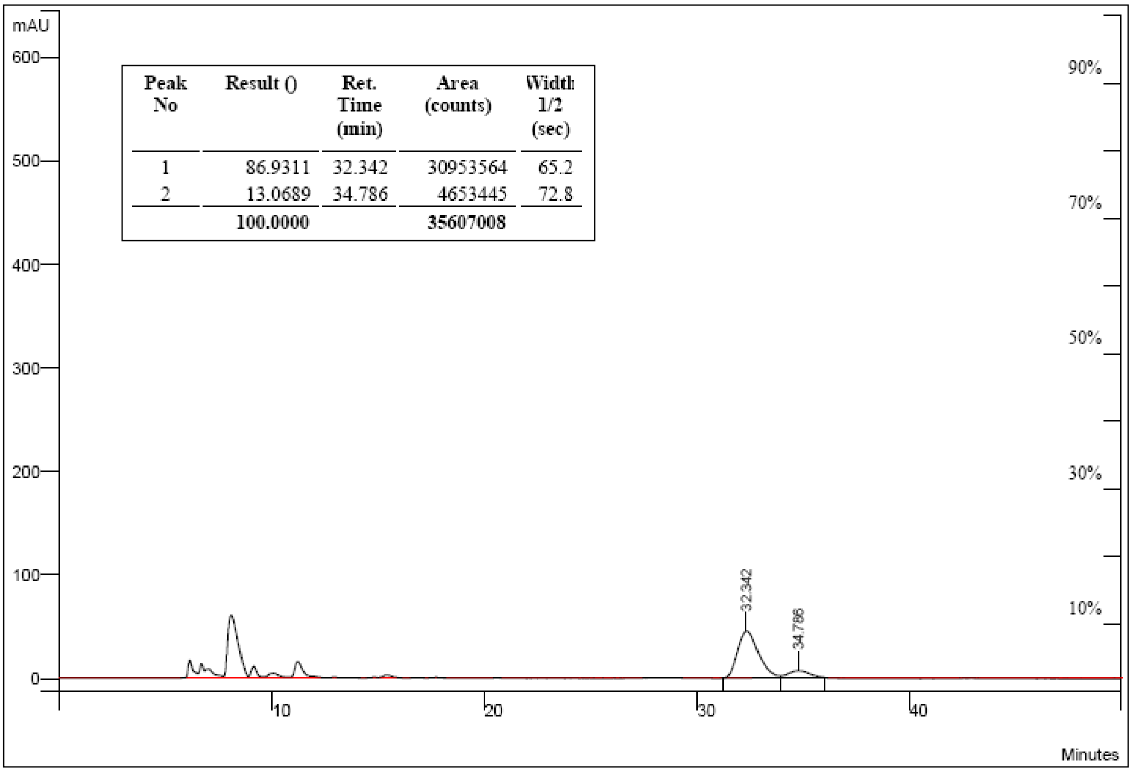

Peak No	Result ()	Ret. Time (min)	Area (counts)	Width 1/2 (sec)
1	86.9311	32.342	30953564	65.2
2	13.0689	34.786	4653445	72.8
	**100.0000**		**35607008**	

HPLC spectrum of the remaining alcohol (*R*)-**2j**

[Chiralcel OD-H, Hexane/IPA = 98/2, 0.5 ml/min, 220 nm]

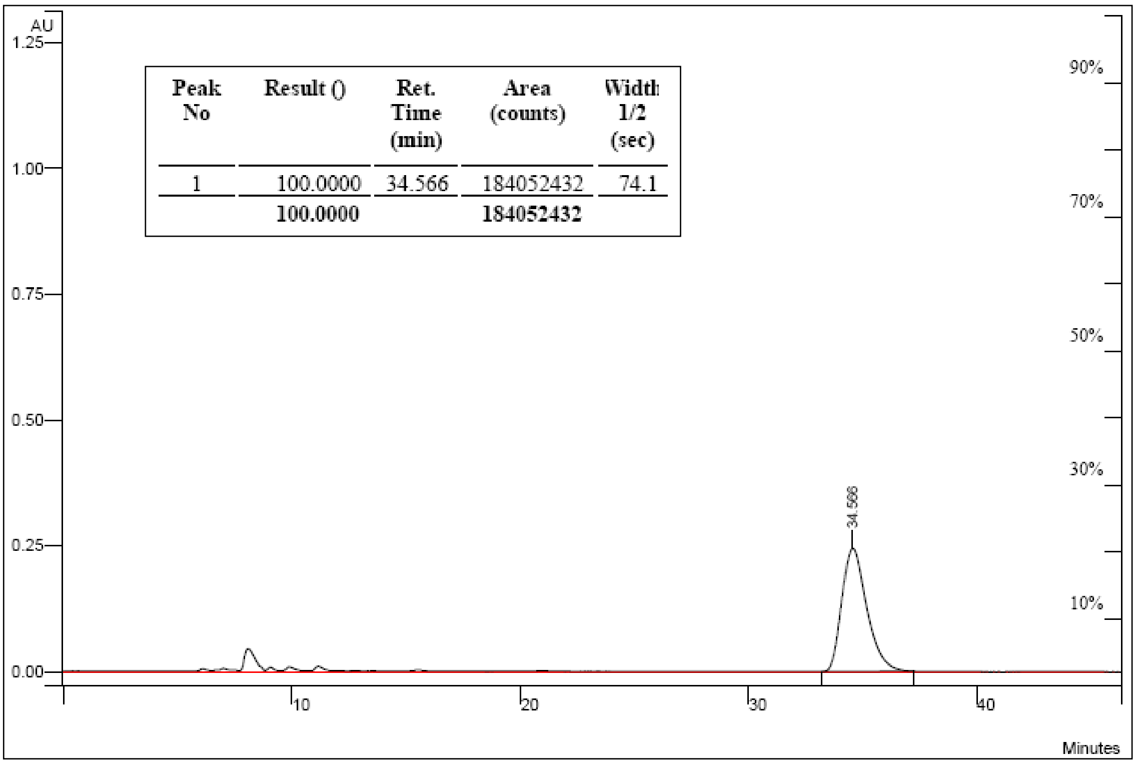

Peak No	Result ()	Ret. Time (min)	Area (counts)	Width 1/2 (sec)
1	100.0000	34.566	184052432	74.1
	**100.0000**		**184052432**	

^{1}H NMR spectrum of the silylation reaction mixture of 2k after 50.9% conversion

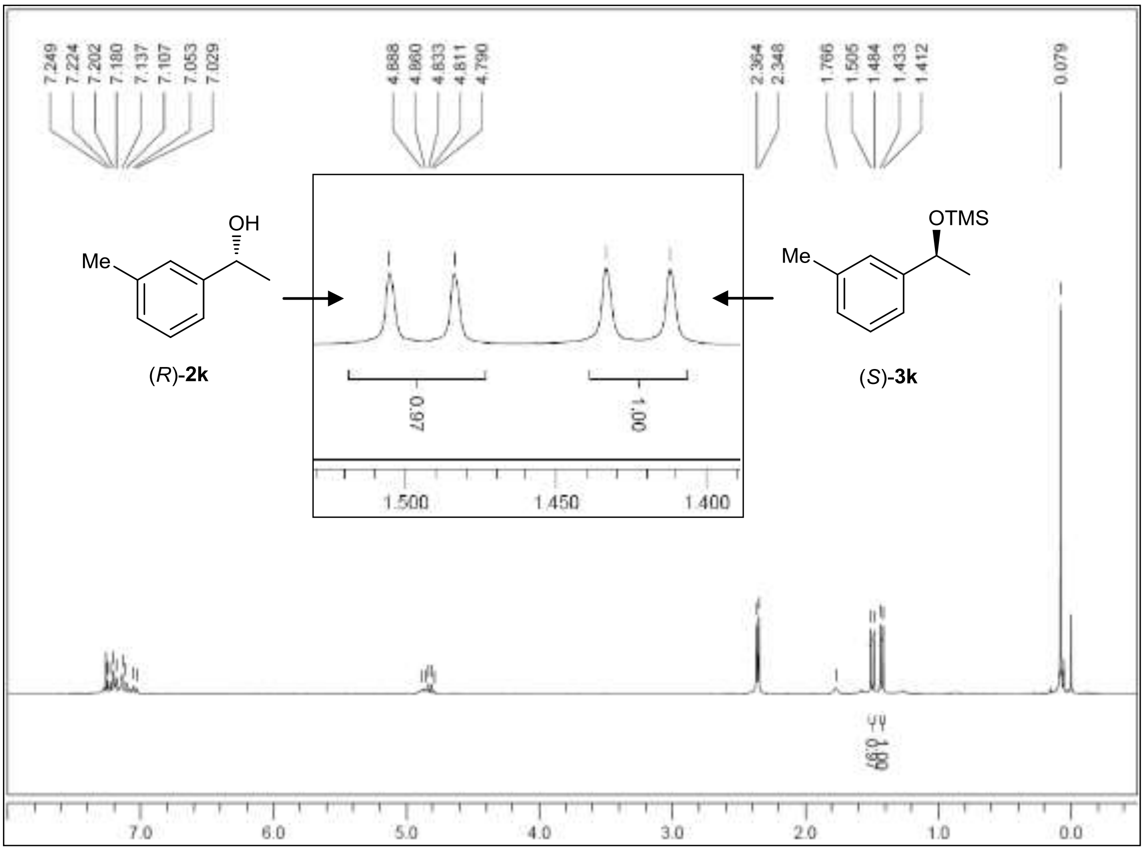

HPLC spectrum of *rac*-**2k** (Chiralcel OB-H, Hexane/IPA = 90/10, 0.5 ml/min, 220 nm)

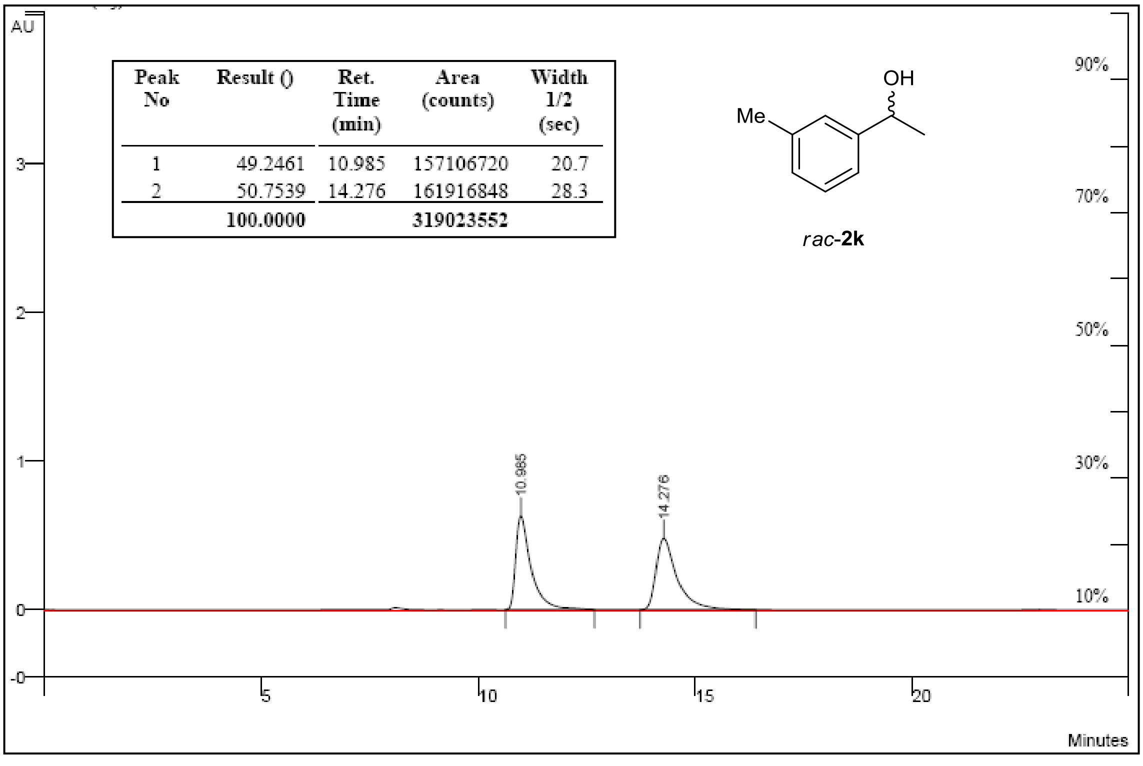

Peak No	Result ()	Ret. Time (min)	Area (counts)	Width 1/2 (sec)
1	49.2461	10.985	157106720	20.7
2	50.7539	14.276	161916848	28.3
	**100.0000**		**319023552**	

HPLC spectrum of the TMS-ether product (*S*)-**3k**

[Chiralcel OB-H, Hexane/IPA = 90/10, 0.5 ml/min, 220 nm]

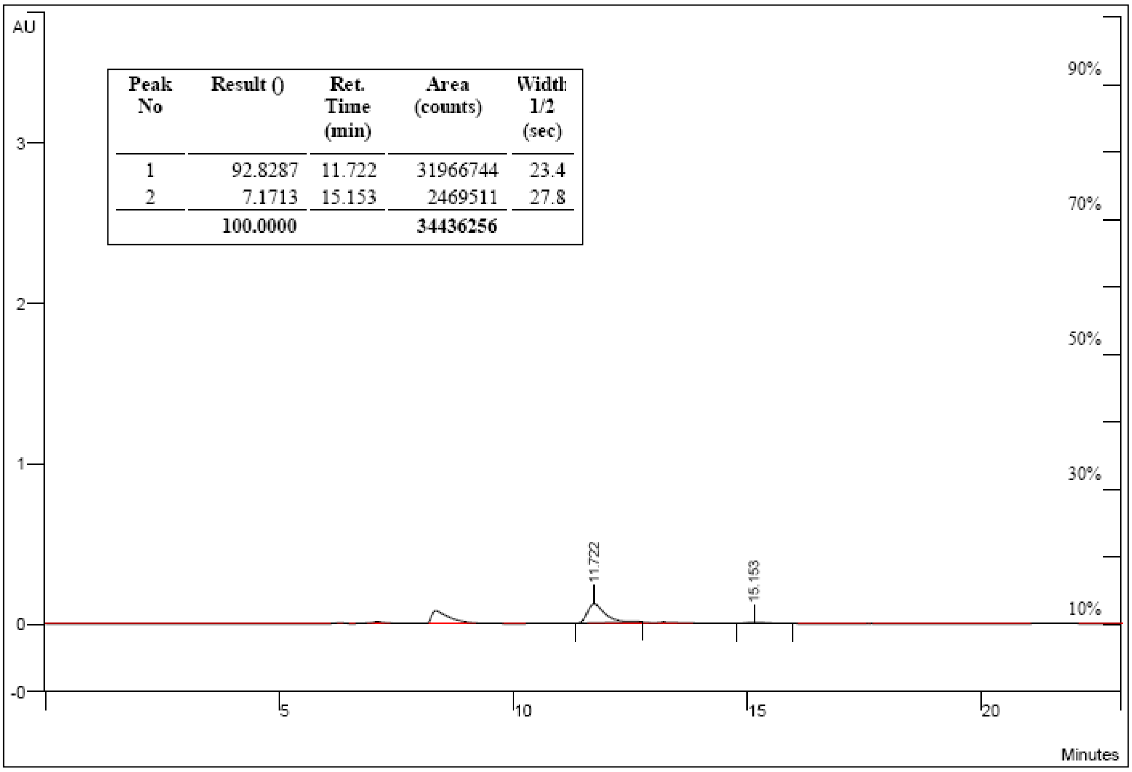

Peak No	Result ()	Ret. Time (min)	Area (counts)	Width 1/2 (sec)
1	92.8287	11.722	31966744	23.4
2	7.1713	15.153	2469511	27.8
	**100.0000**		**34436256**	

HPLC spectrum of the remaining alcohol (*R*)-**2k**

[Chiralcel OB-H, Hexane/IPA = 90/10, 0.5 ml/min, 220 nm]

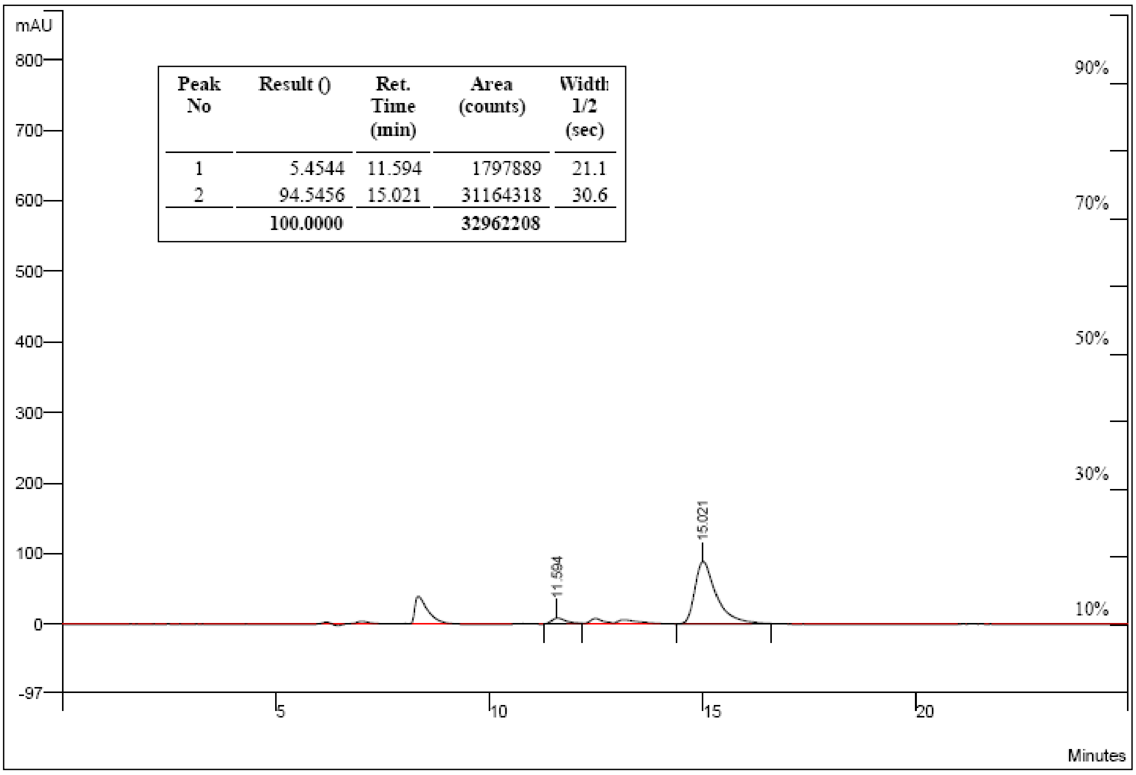

Peak No	Result ()	Ret. Time (min)	Area (counts)	Width 1/2 (sec)
1	5.4544	11.594	1797889	21.1
2	94.5456	15.021	31164318	30.6
	**100.0000**		**32962208**	

^{1}H NMR spectrum of the silylation reaction mixture of 2l after 52.9% conversion

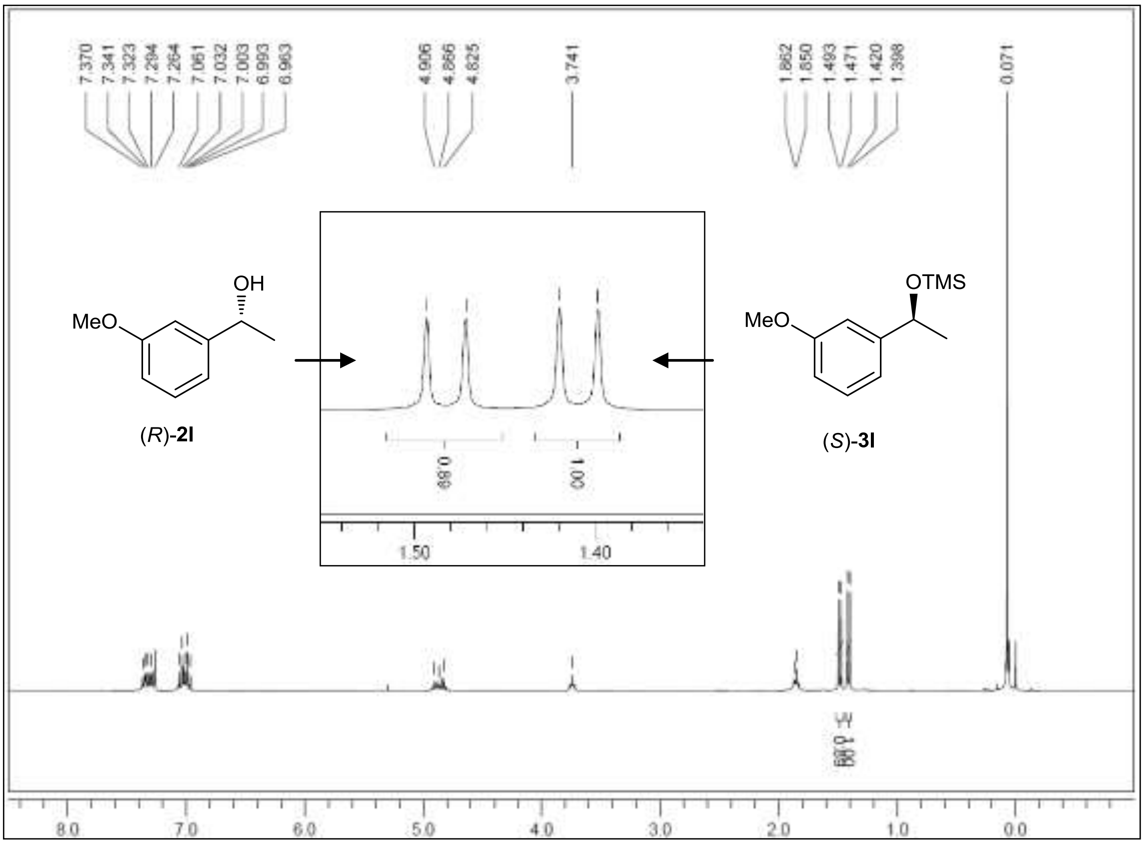

HPLC spectrum of *rac*-**2l** (Chiralcel OB-H, Hexane/IPA = 90/10, 0.5 ml/min, 220 nm)

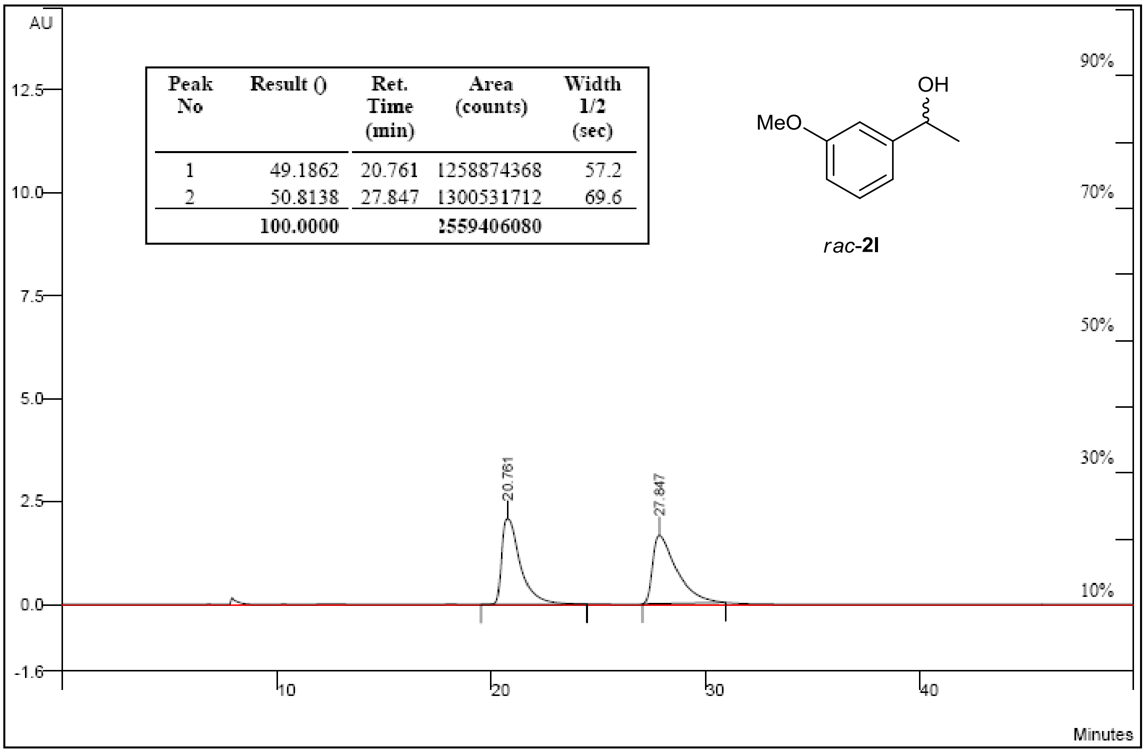

Peak No	Result ()	Ret. Time (min)	Area (counts)	Width 1/2 (sec)
1	49.1862	20.761	1258874368	57.2
2	50.8138	27.847	1300531712	69.6
	100.0000		2559406080	

HPLC spectrum of the TMS-ether product (*S*)-**3l**

[Chiralcel OB-H, Hexane/IPA = 90/10, 0.5 ml/min, 220 nm]

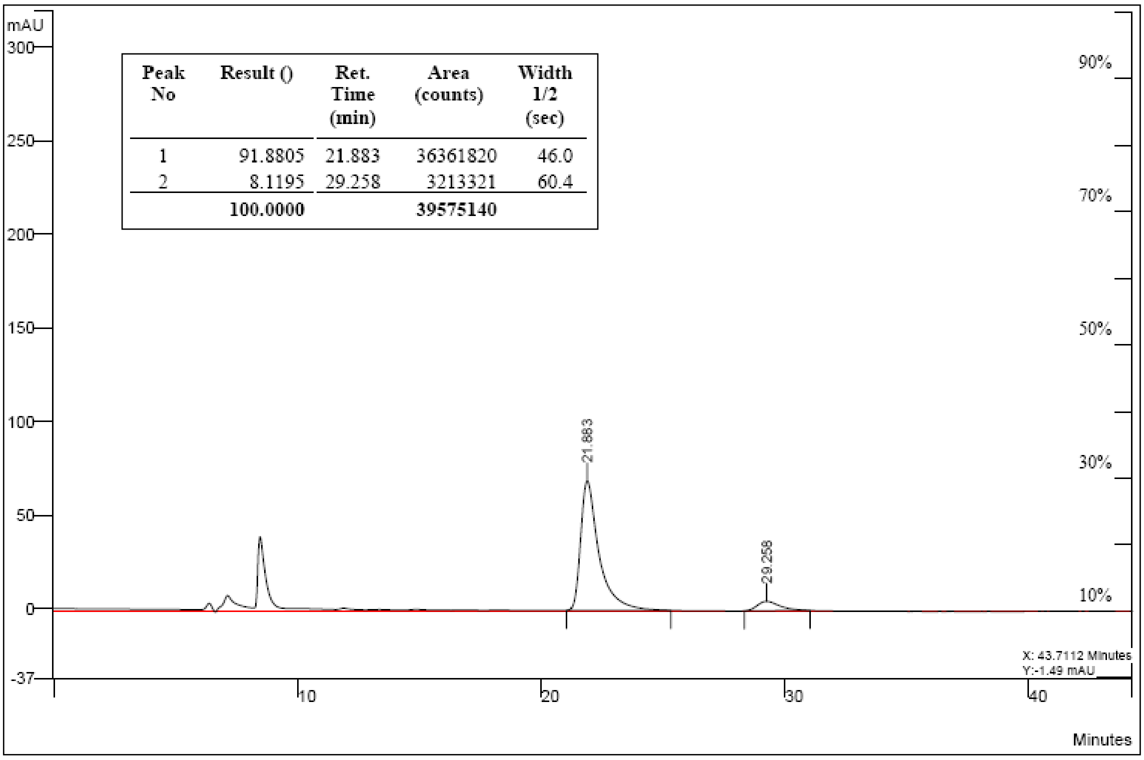

Peak No	Result ()	Ret. Time (min)	Area (counts)	Width 1/2 (sec)
1	91.8805	21.883	36361820	46.0
2	8.1195	29.258	3213321	60.4
	**100.0000**		**39575140**	

HPLC spectrum of the remaining alcohol (*R*)-**2l**

[Chiralcel OB-H, Hexane/IPA = 90/10, 0.5 ml/min, 220 nm]

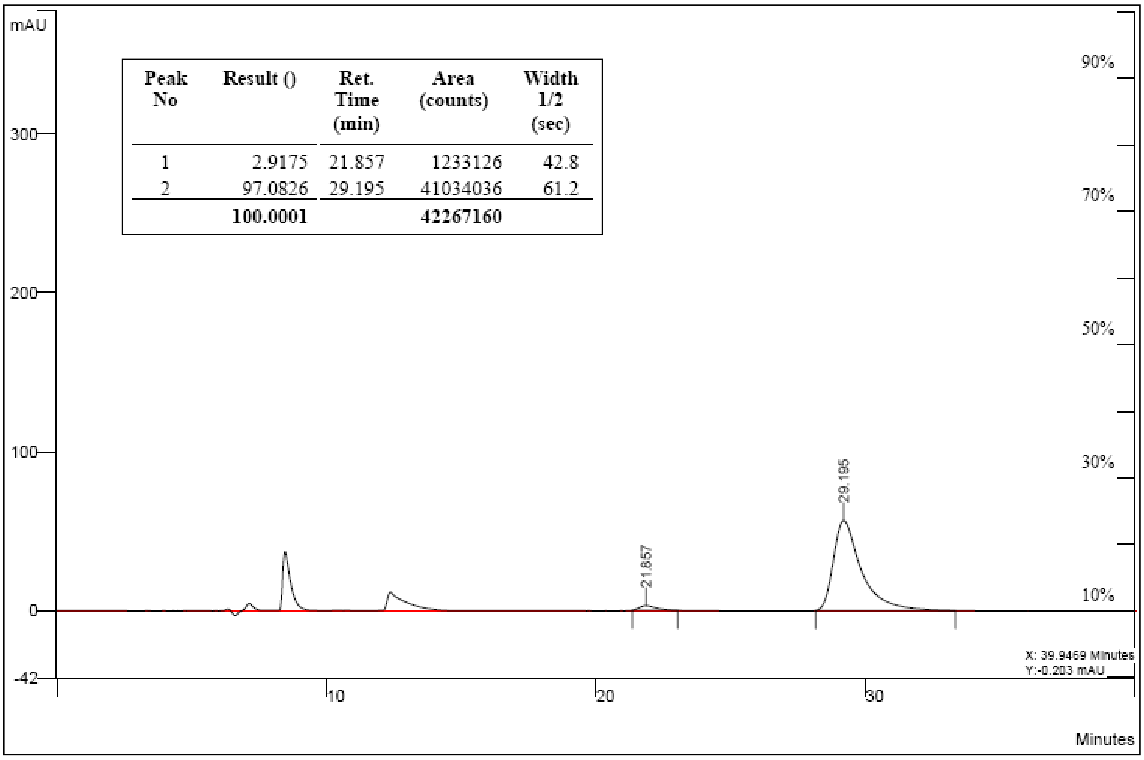

Peak No	Result ()	Ret. Time (min)	Area (counts)	Width 1/2 (sec)
1	2.9175	21.857	1233126	42.8
2	97.0826	29.195	41034036	61.2
	**100.0001**		**42267160**	

^{1}H NMR spectrum of the silylation reaction mixture of 2m after 49.7% conversion

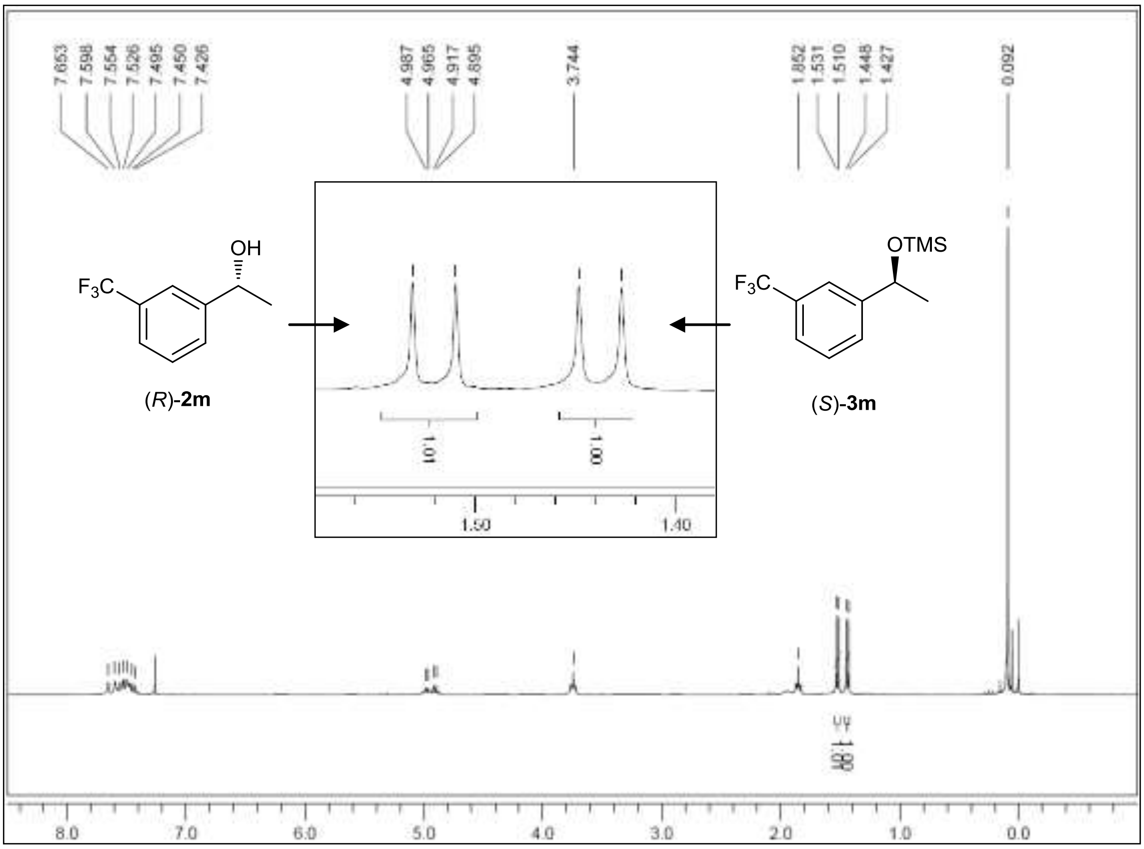

HPLC spectrum of *rac*-**2m** (Chiralcel OJ-H, Hexane/IPA = 99/1, 0.5 ml/min, 220 nm)

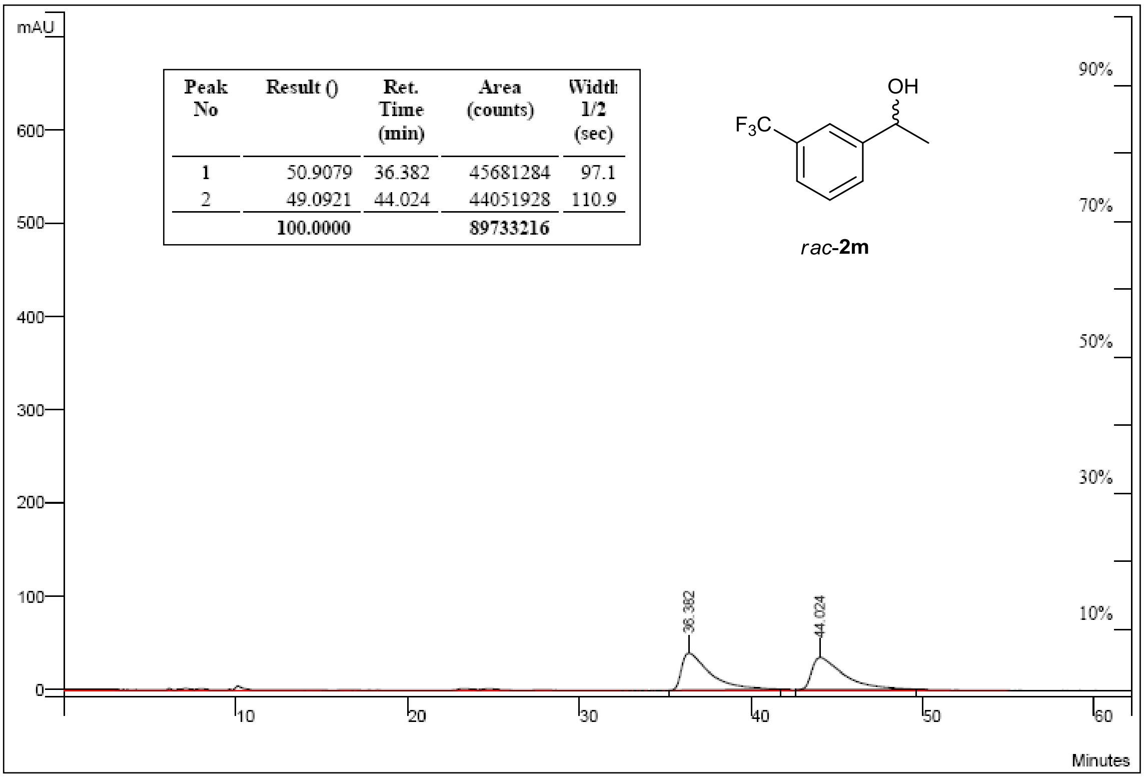

Peak No	Result ()	Ret. Time (min)	Area (counts)	Width 1/2 (sec)
1	50.9079	36.382	45681284	97.1
2	49.0921	44.024	44051928	110.9
	100.0000		89733216	

HPLC spectrum of the TMS-ether product (*S*)-**3m**

[Chiralcel OJ-H, Hexane/IPA = 99/1, 0.5 ml/min, 220 nm]

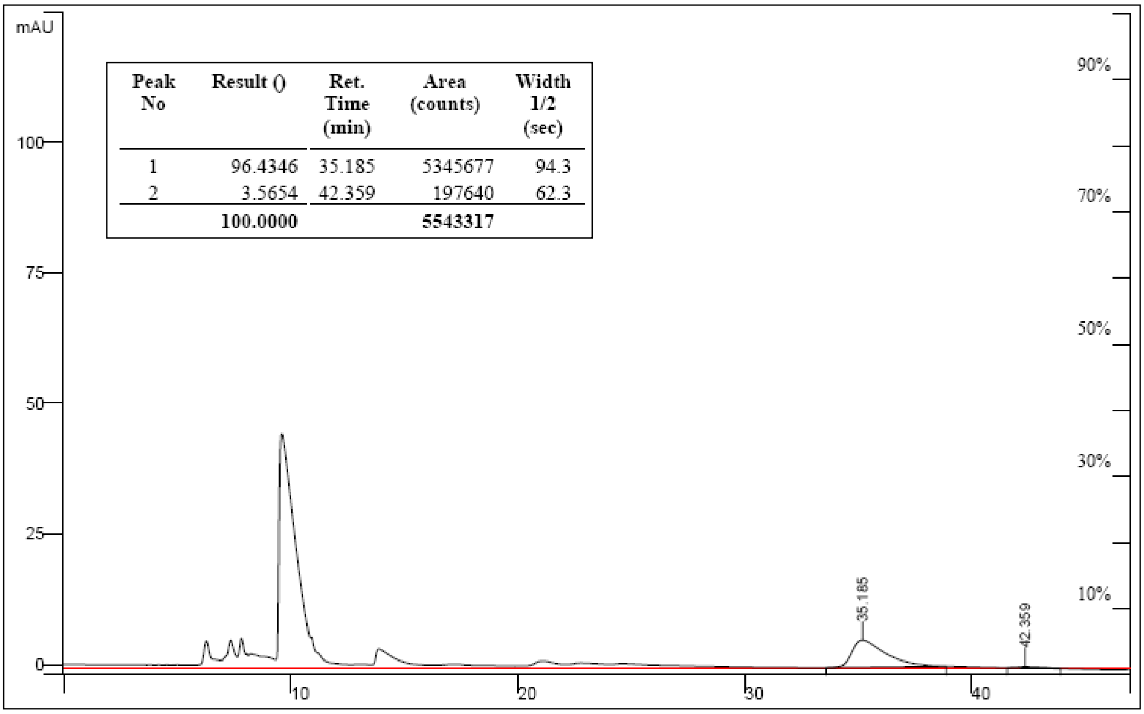

Peak No	Result ()	Ret. Time (min)	Area (counts)	Width 1/2 (sec)
1	96.4346	35.185	5345677	94.3
2	3.5654	42.359	197640	62.3
	**100.0000**		**5543317**	

HPLC spectrum of the remaining alcohol (*R*)-**2m**

[Chiralcel OJ-H, Hexane/IPA = 99/1, 0.5 ml/min, 220 nm]

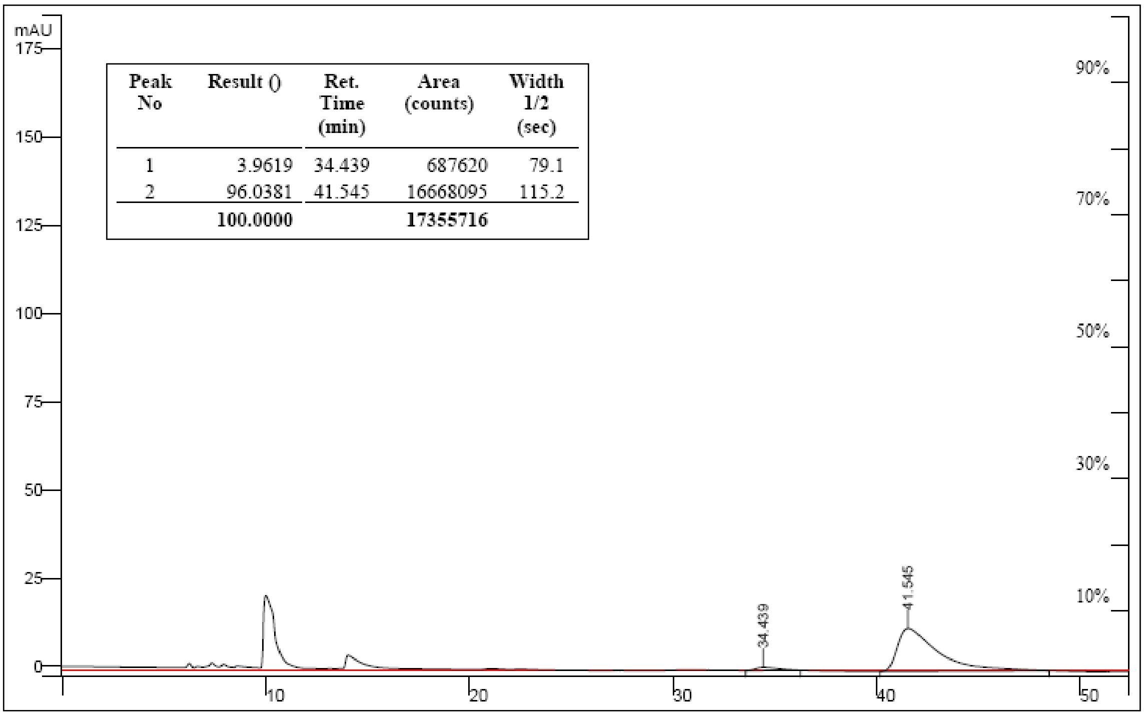

Peak No	Result ()	Ret. Time (min)	Area (counts)	Width 1/2 (sec)
1	3.9619	34.439	687620	79.1
2	96.0381	41.545	16668095	115.2
	**100.0000**		**17355716**	

^{1}H NMR spectrum of the silylation reaction mixture of 2n after 53.0% conversion

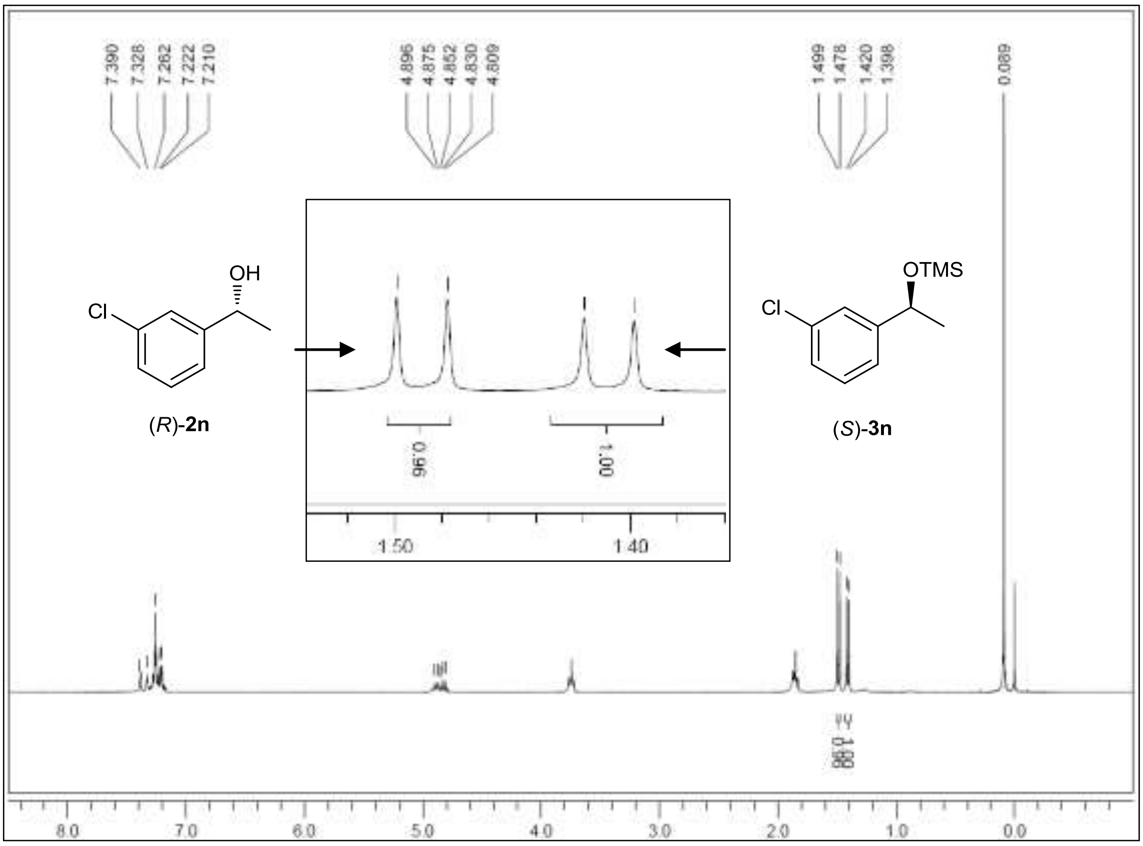

HPLC spectrum of *rac*-**2n** (Chiralcel OB-H, Hexane/IPA = 95/5, 0.5 ml/min, 220 nm)

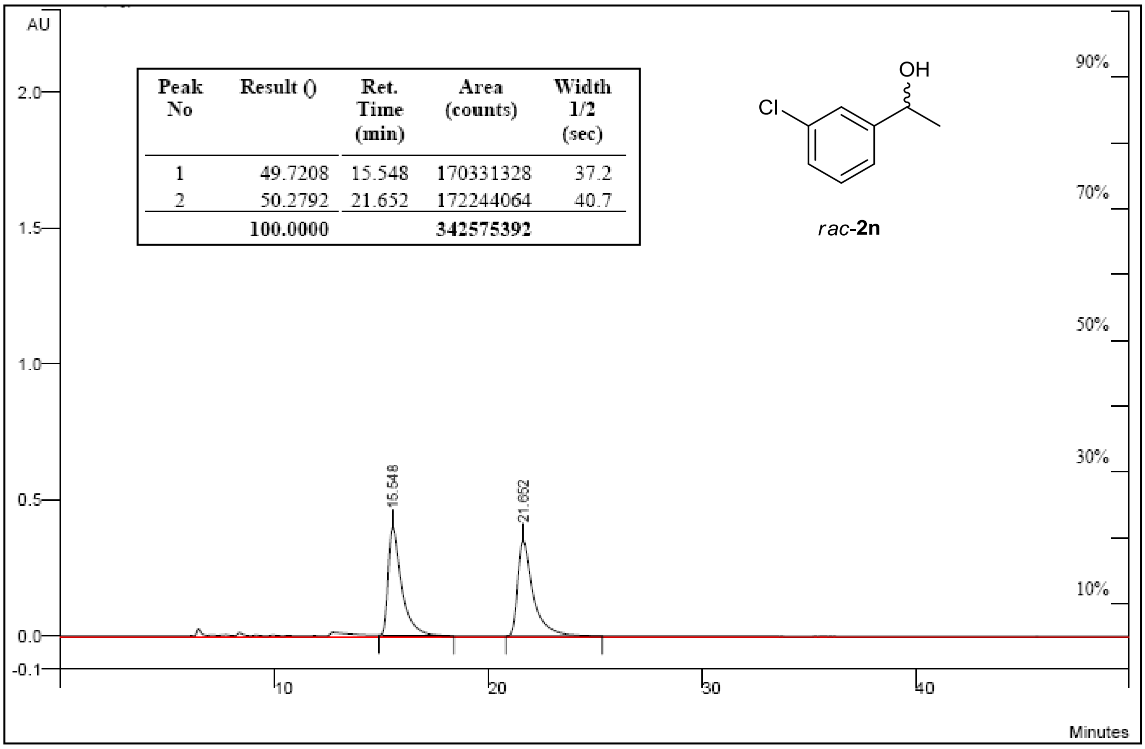

Peak No	Result ()	Ret. Time (min)	Area (counts)	Width 1/2 (sec)
1	49.7208	15.548	170331328	37.2
2	50.2792	21.652	172244064	40.7
	**100.0000**		**342575392**	

HPLC spectrum of the TMS-ether product (*S*)-**3n**

[Chiralcel OB-H, Hexane/IPA = 95/5, 0.5 ml/min, 220 nm]

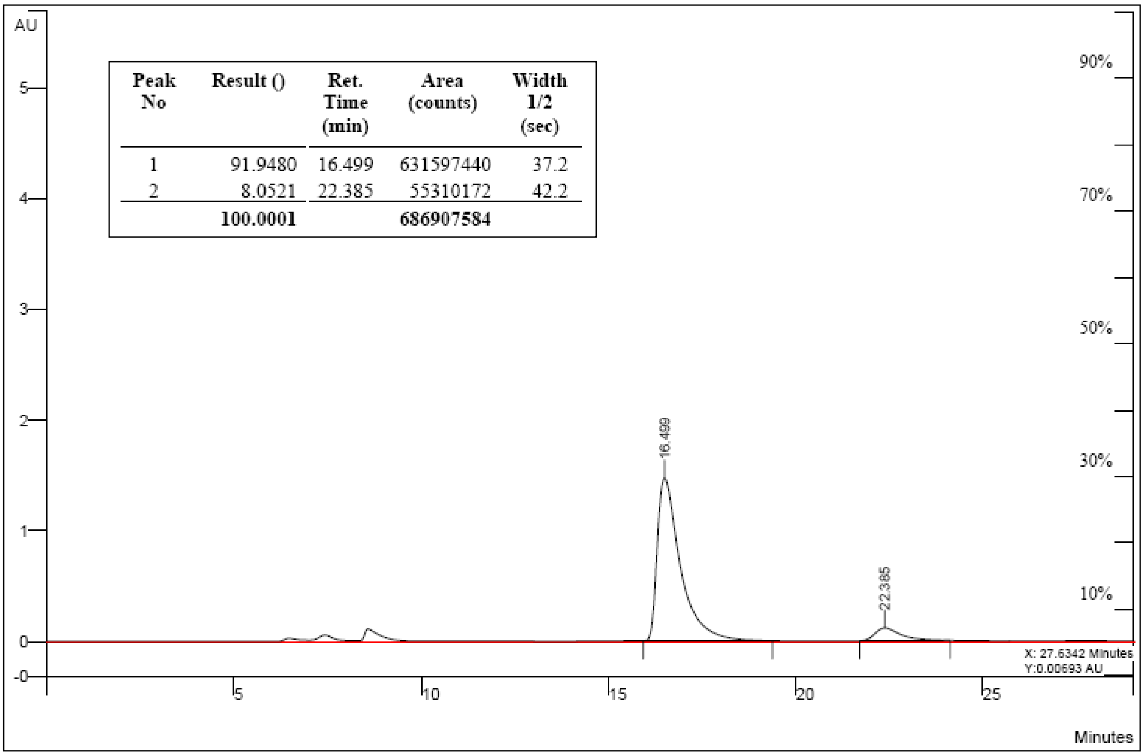

Peak No	Result ()	Ret. Time (min)	Area (counts)	Width 1/2 (sec)
1	91.9480	16.499	631597440	37.2
2	8.0521	22.385	55310172	42.2
	**100.0001**		**686907584**	

HPLC spectrum of the remaining alcohol (*R*)-**2n**

[Chiralcel OB-H, Hexane/IPA = 95/5, 0.5 ml/min, 220 nm]

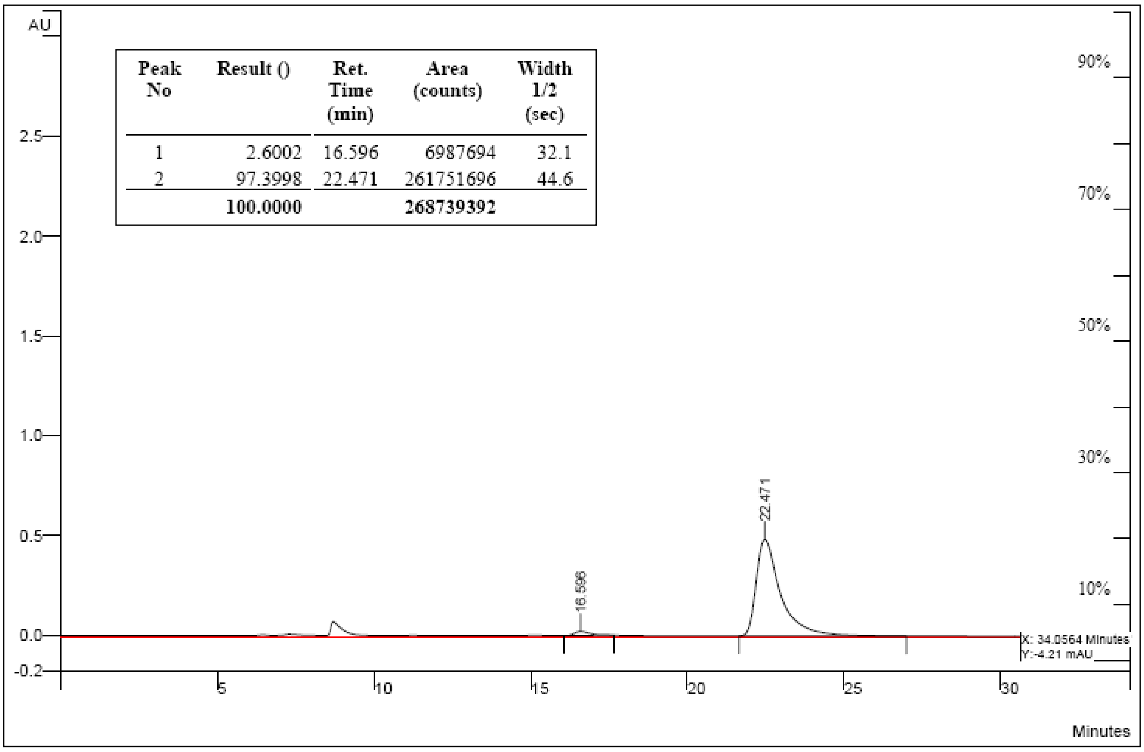

Peak No	Result ()	Ret. Time (min)	Area (counts)	Width 1/2 (sec)
1	2.6002	16.596	6987694	32.1
2	97.3998	22.471	261751696	44.6
	**100.0000**		**268739392**	

^{1}H NMR spectrum of the silylation reaction mixture of 2o after 52.6% conversion

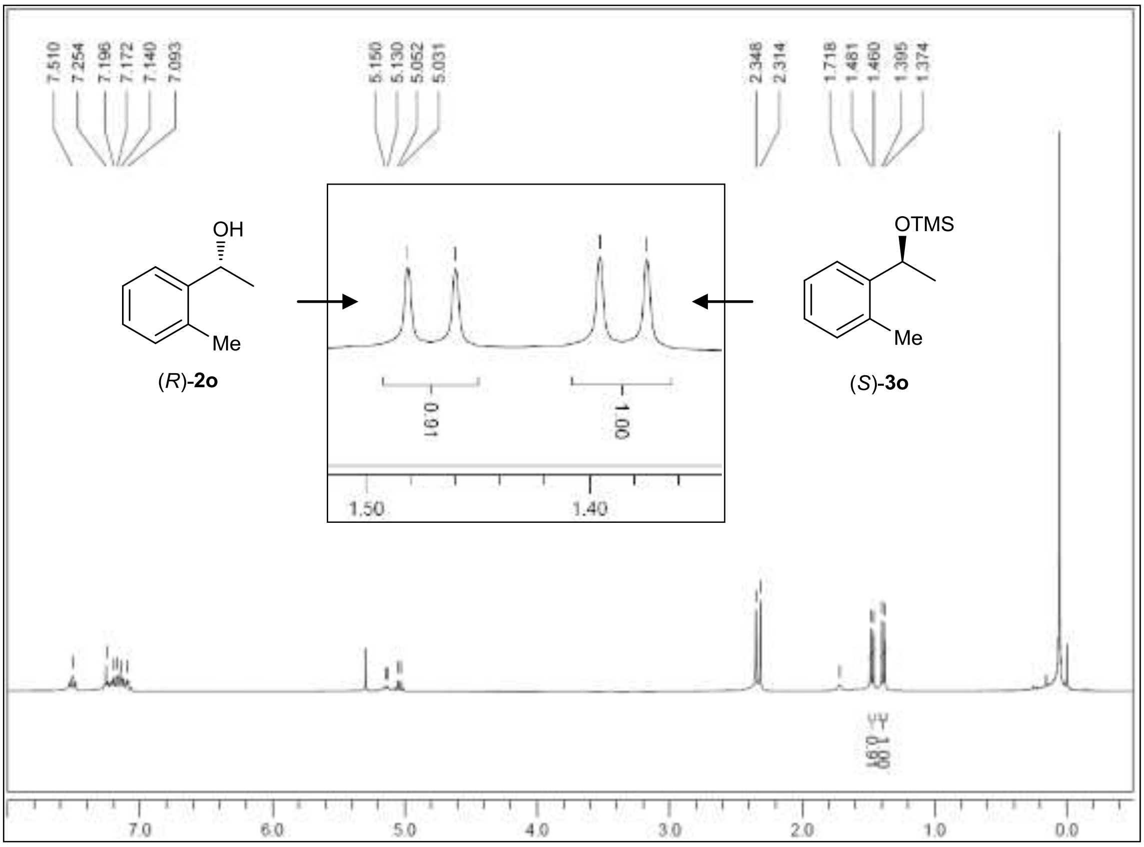

HPLC spectrum of *rac*-**2o** (Chiralcel OB-H, Hexane/IPA = 90/10, 0.5 ml/min, 220 nm)

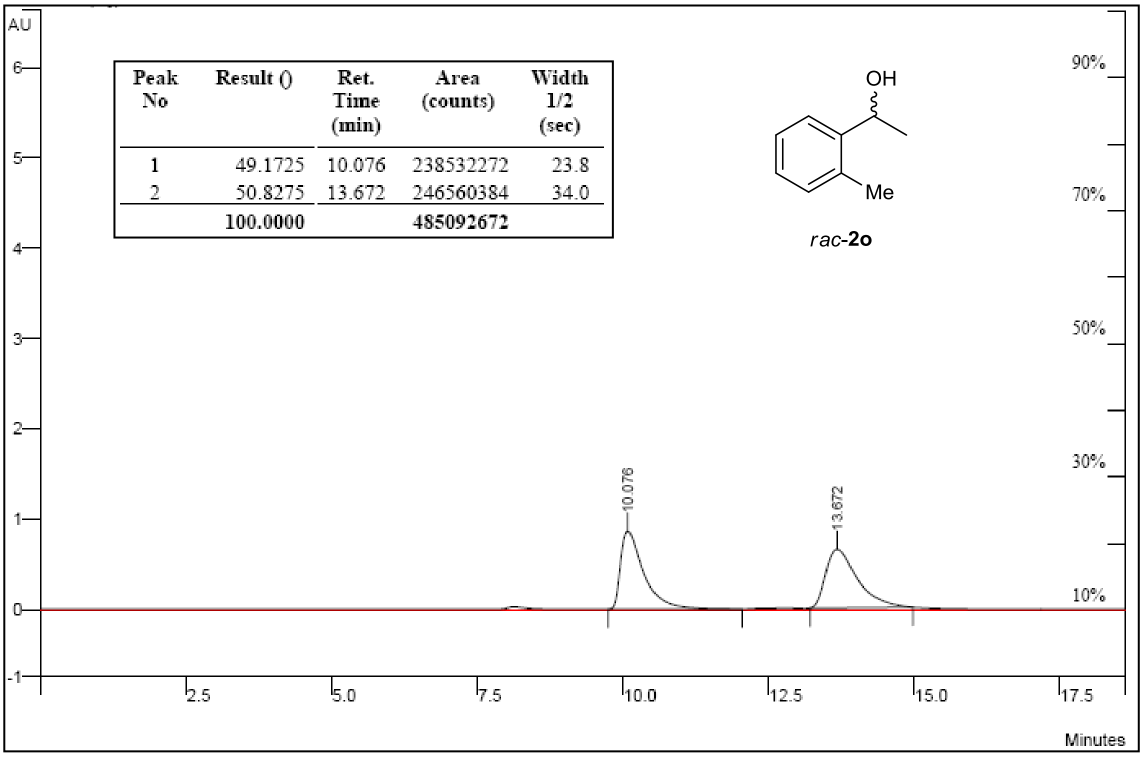

Peak No	Result ()	Ret. Time (min)	Area (counts)	Width 1/2 (sec)
1	49.1725	10.076	238532272	23.8
2	50.8275	13.672	246560384	34.0
	**100.0000**		**485092672**	

HPLC spectrum of the TMS-ether product (*S*)-**3o**

[Chiralcel OB-H, Hexane/IPA = 90/10, 0.5 ml/min, 220 nm]

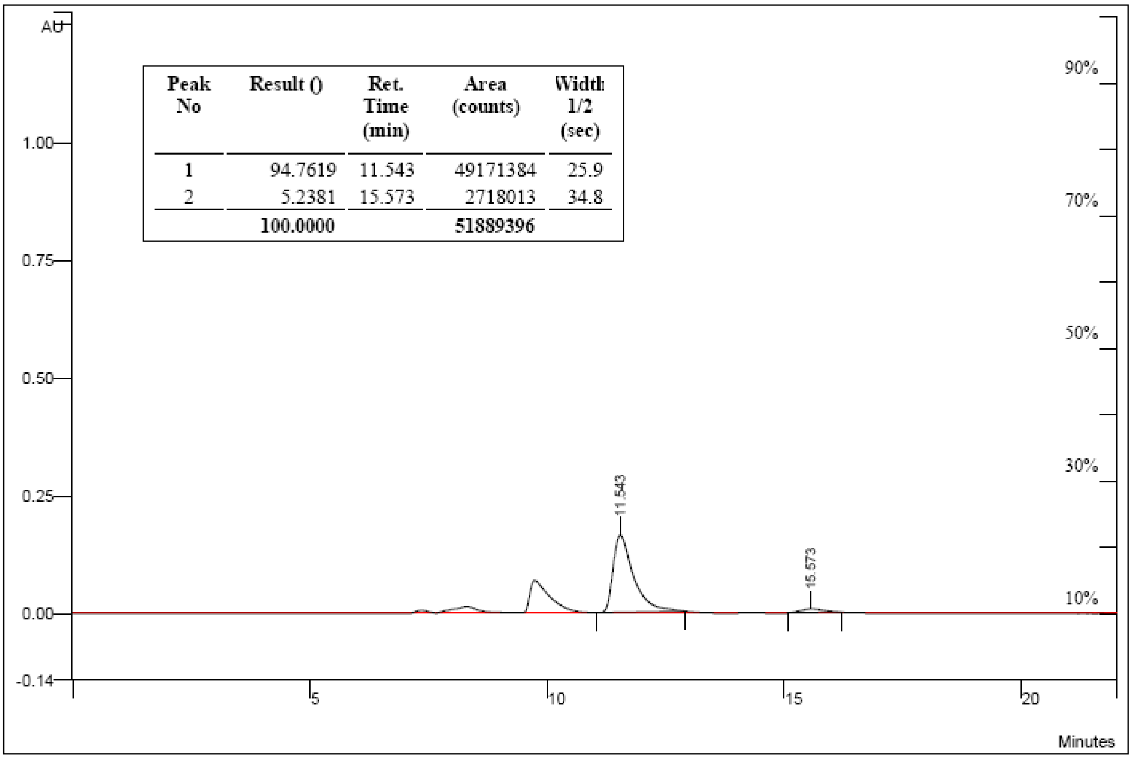

Peak No	Result ()	Ret. Time (min)	Area (counts)	Width 1/2 (sec)
1	94.7619	11.543	49171384	25.9
2	5.2381	15.573	2718013	34.8
	**100.0000**		**51889396**	

HPLC spectrum of the remaining alcohol (*R*)-**2o**

[Chiralcel OB-H, Hexane/IPA = 90/10, 0.5 ml/min, 220 nm]

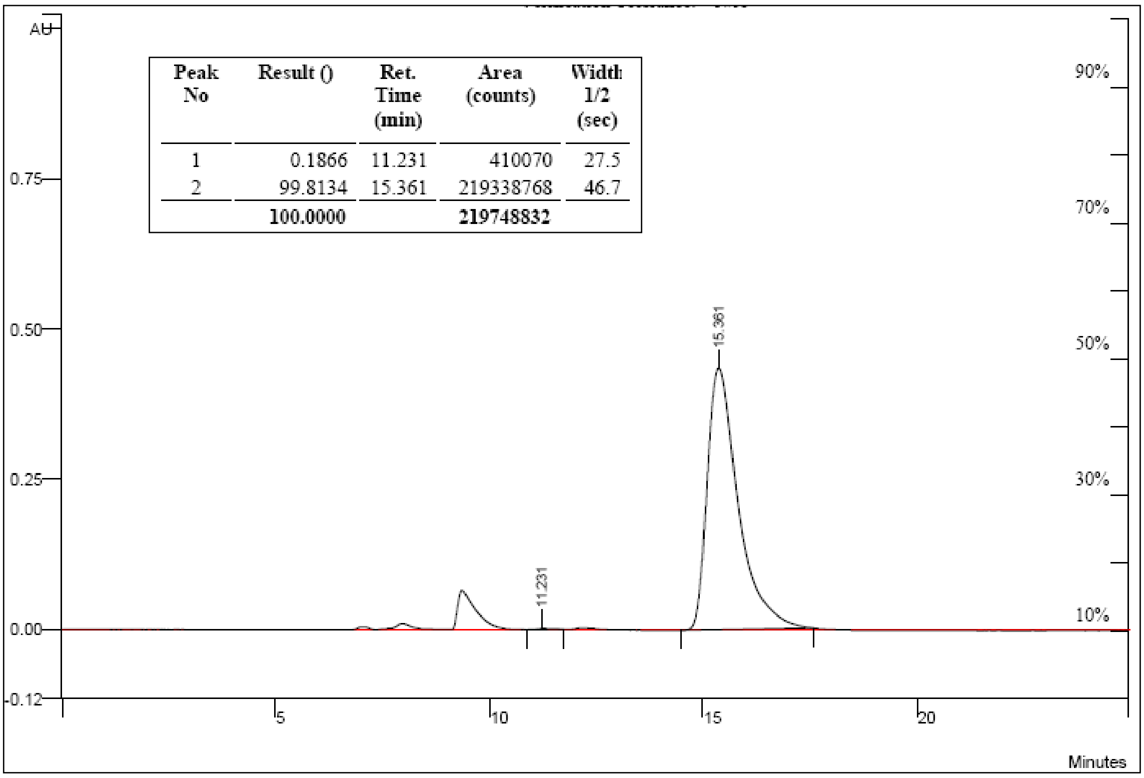

Peak No	Result ()	Ret. Time (min)	Area (counts)	Width 1/2 (sec)
1	0.1866	11.231	410070	27.5
2	99.8134	15.361	219338768	46.7
	**100.0000**		**219748832**	

^{1}H NMR spectrum of the silylation reaction mixture of 2p after 58.5% conversion (cat 1e)

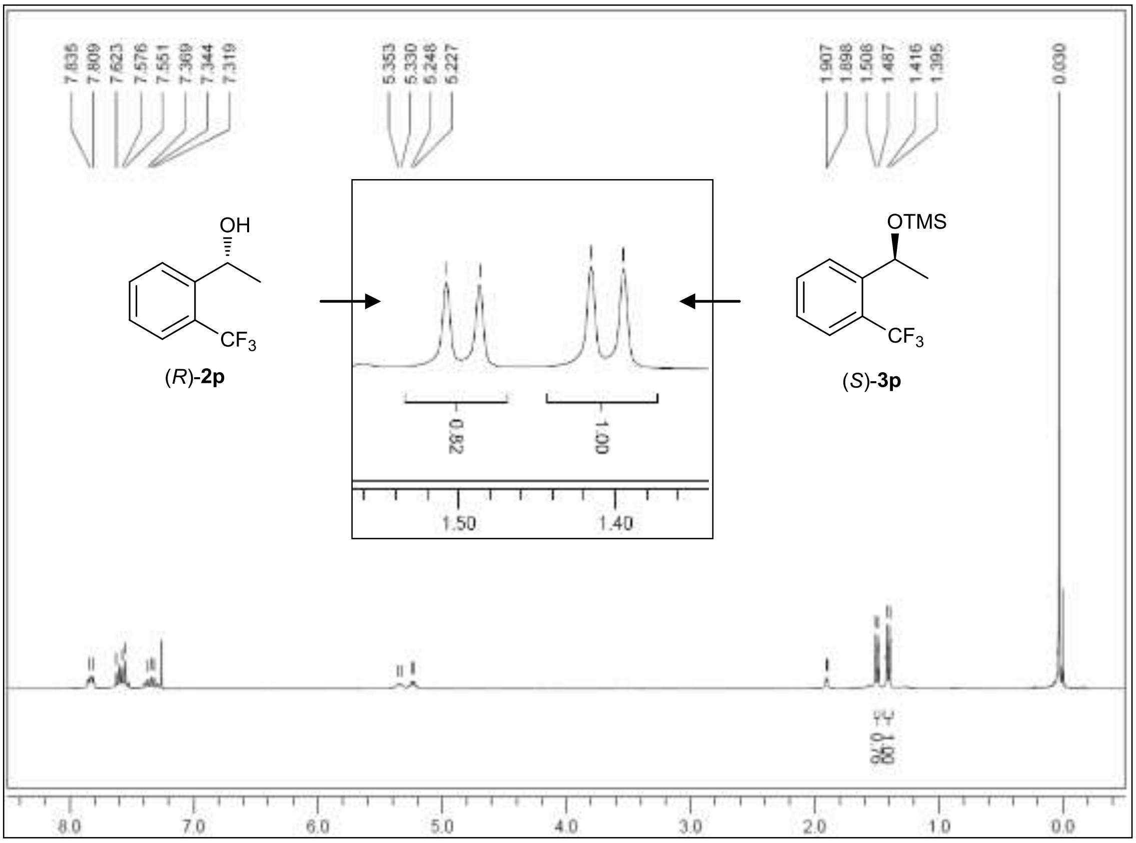

HPLC spectrum of *rac*-**2p** (Chiralcel OB-H, Hexane/IPA = 99.8/0.2, 0.5 ml/min, 220 nm)

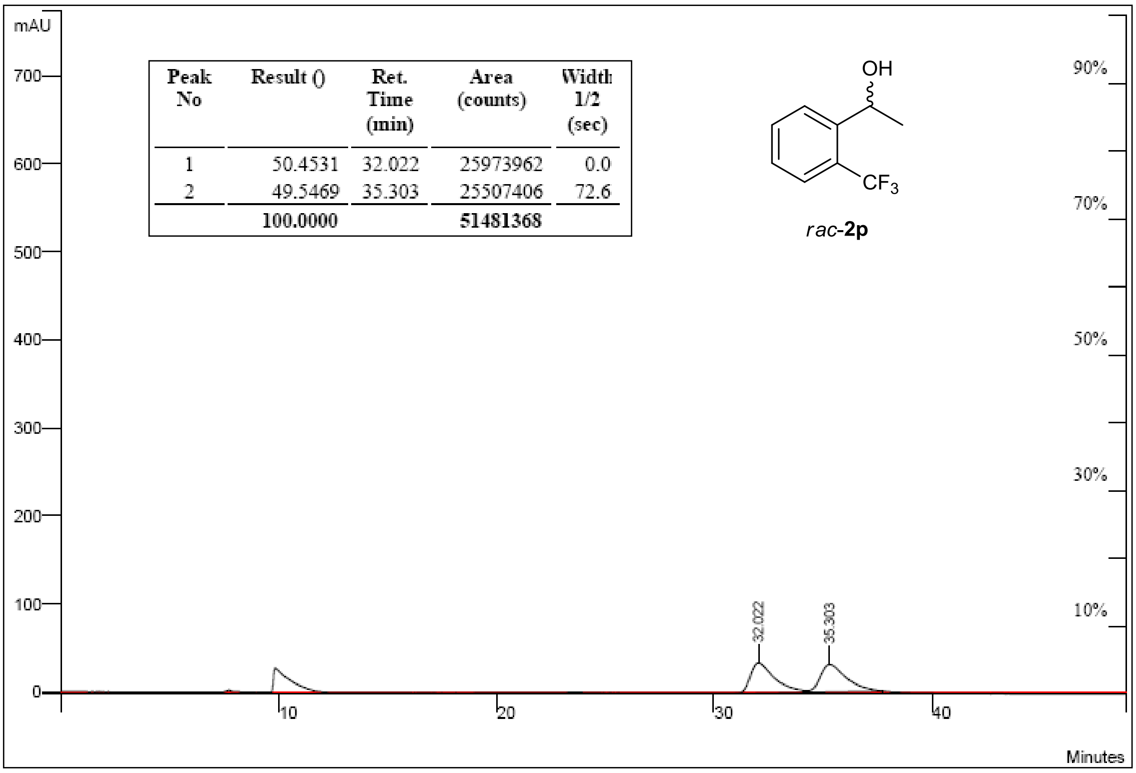

HPLC spectrum of the TMS-ether product (*S*)-**3p**

[Chiralcel OB-H, Hexane/IPA = 99.8/0.2, 0.5 ml/min, 220 nm]

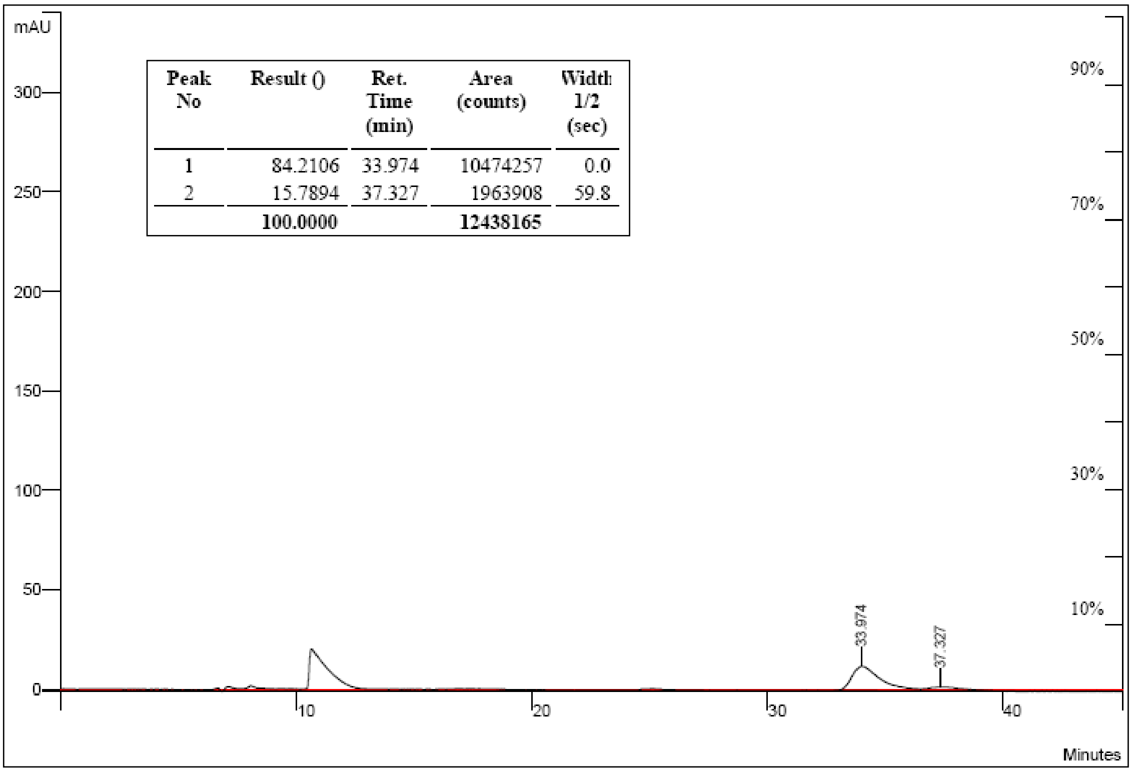

Peak No	Result ()	Ret. Time (min)	Area (counts)	Width 1/2 (sec)
1	84.2106	33.974	10474257	0.0
2	15.7894	37.327	1963908	59.8
	**100.0000**		**12438165**	

HPLC spectrum of the remaining alcohol (*R*)-**2p**

[Chiralcel OB-H, Hexane/IPA = 99.8/0.2, 0.5 ml/min, 220 nm]

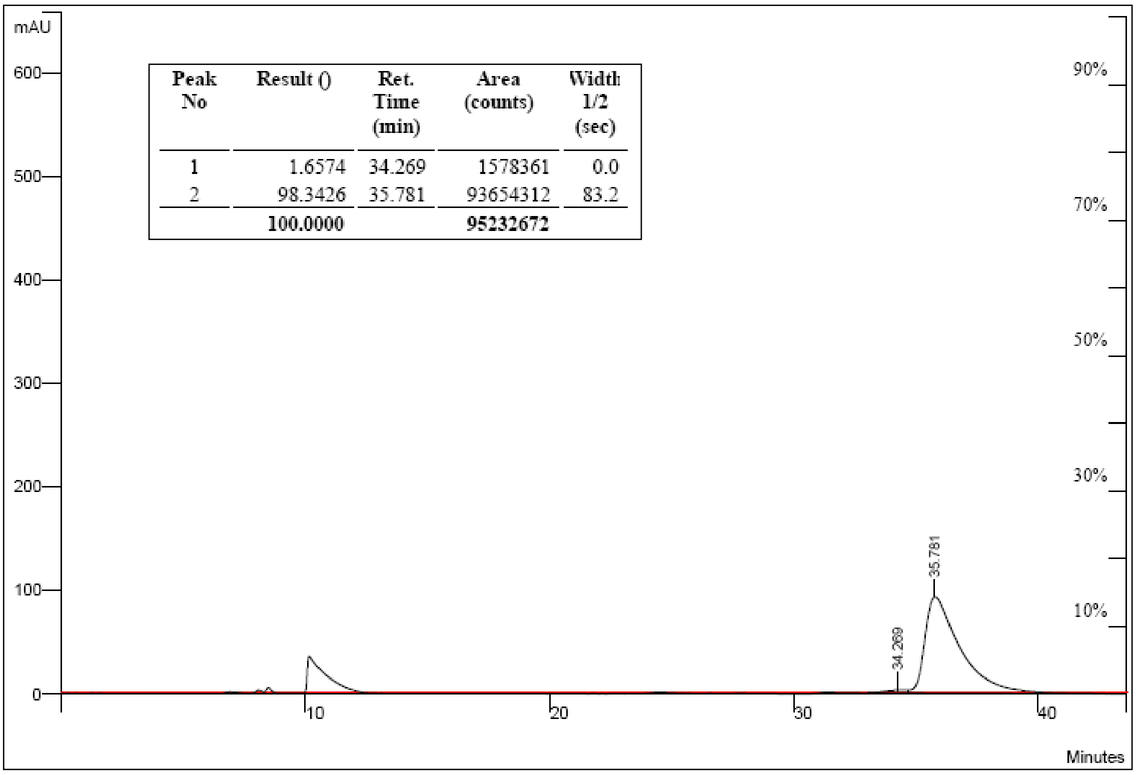

Peak No	Result ()	Ret. Time (min)	Area (counts)	Width 1/2 (sec)
1	1.6574	34.269	1578361	0.0
2	98.3426	35.781	93654312	83.2
	**100.0000**		**95232672**	

^{1}H NMR spectrum of the silylation reaction mixture of 2p after 48.5% conversion (cat 1d)

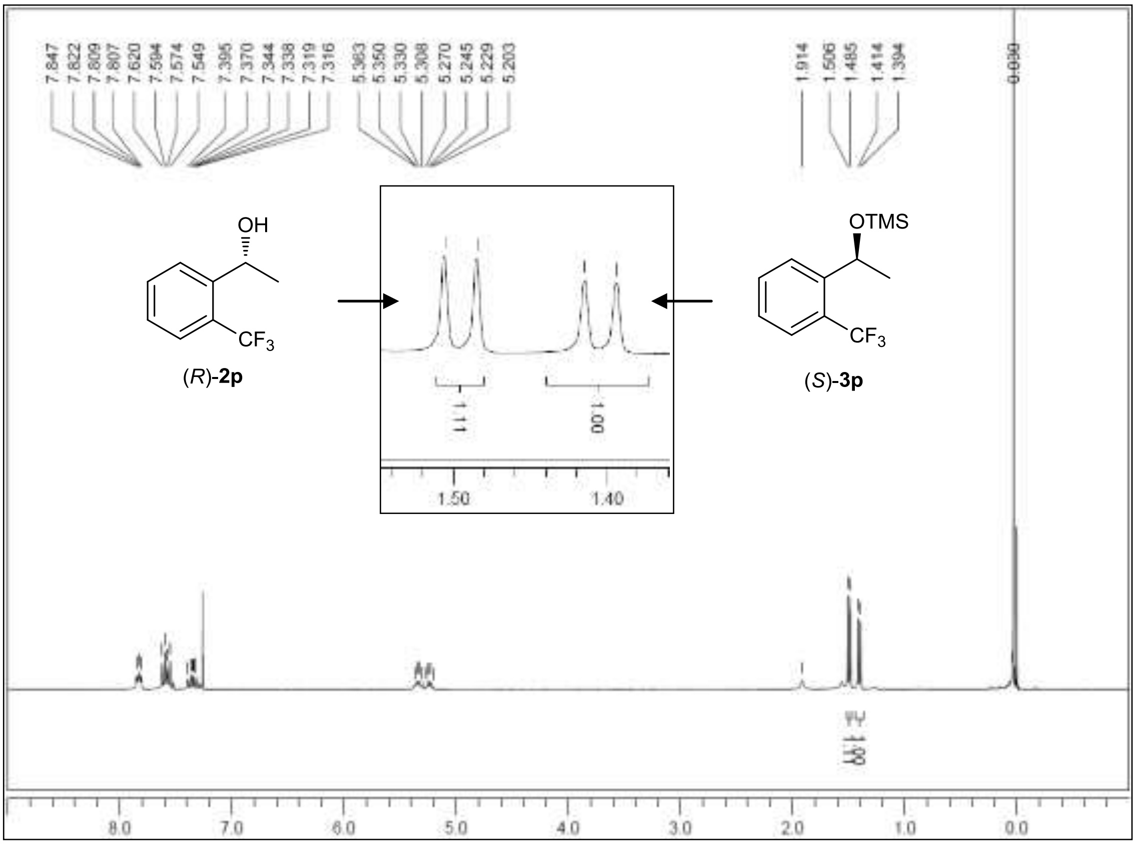

HPLC spectrum of *rac*-**2p** (Chiralcel OB-H, Hexane/IPA = 99.8/0.2, 0.5 ml/min, 220 nm)

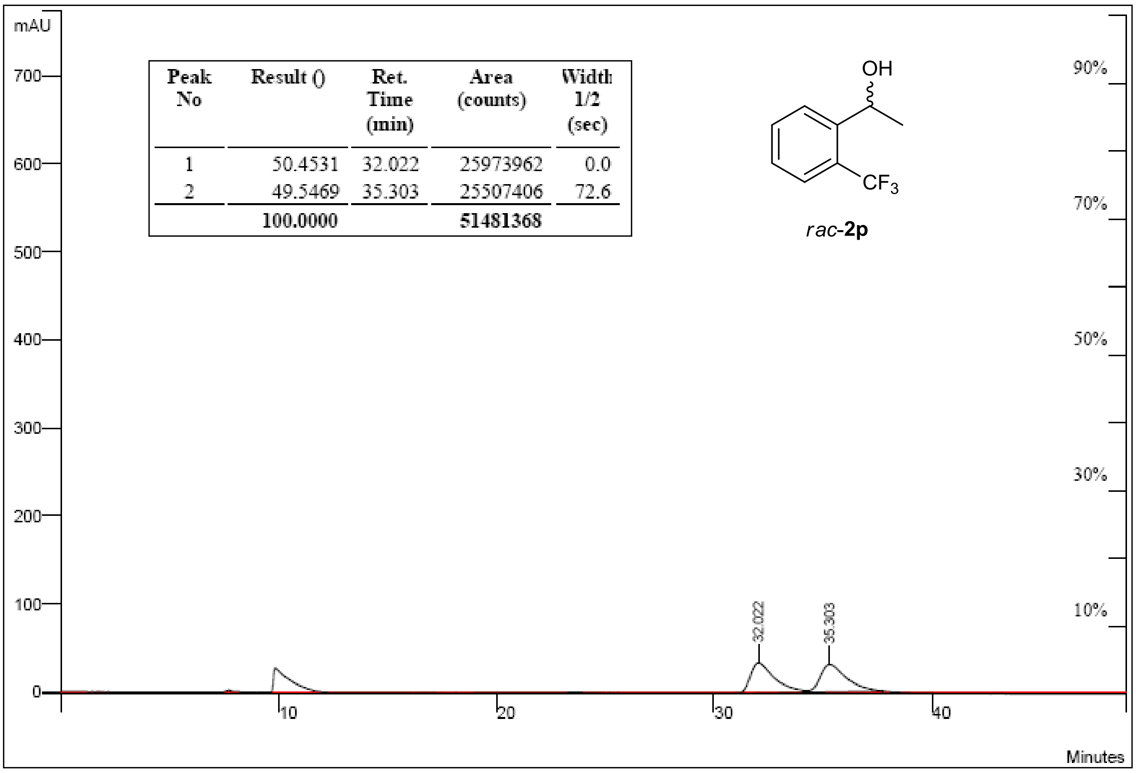

Peak No	Result ()	Ret. Time (min)	Area (counts)	Width 1/2 (sec)
1	50.4531	32.022	25973962	0.0
2	49.5469	35.303	25507406	72.6
	**100.0000**		**51481368**	

HPLC spectrum of the TMS-ether product (*S*)-**3p**

[Chiralcel OB-H, Hexane/IPA = 99.8/0.2, 0.5 ml/min, 220 nm]

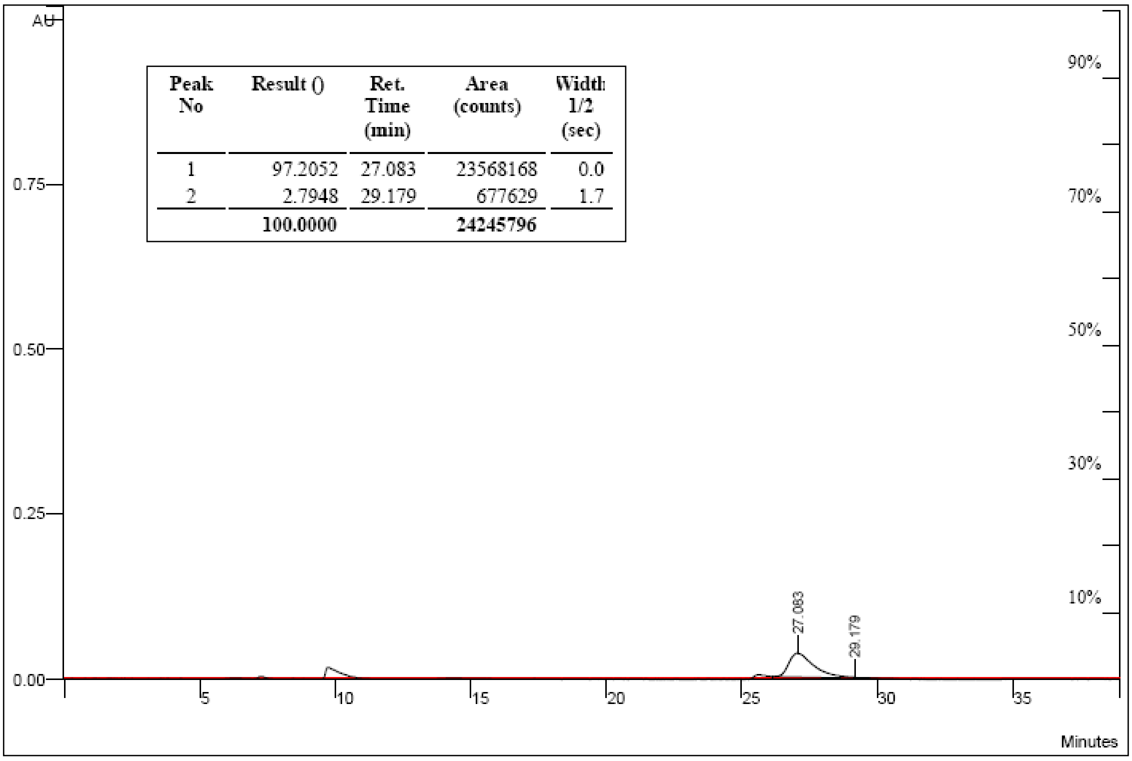

Peak No	Result ()	Ret. Time (min)	Area (counts)	Width 1/2 (sec)
1	97.2052	27.083	23568168	0.0
2	2.7948	29.179	677629	1.7
	**100.0000**		**24245796**	

HPLC spectrum of the remaining alcohol (*R*)-**2p**

[Chiralcel OB-H, Hexane/IPA = 99.8/0.2, 0.5 ml/min, 220 nm]

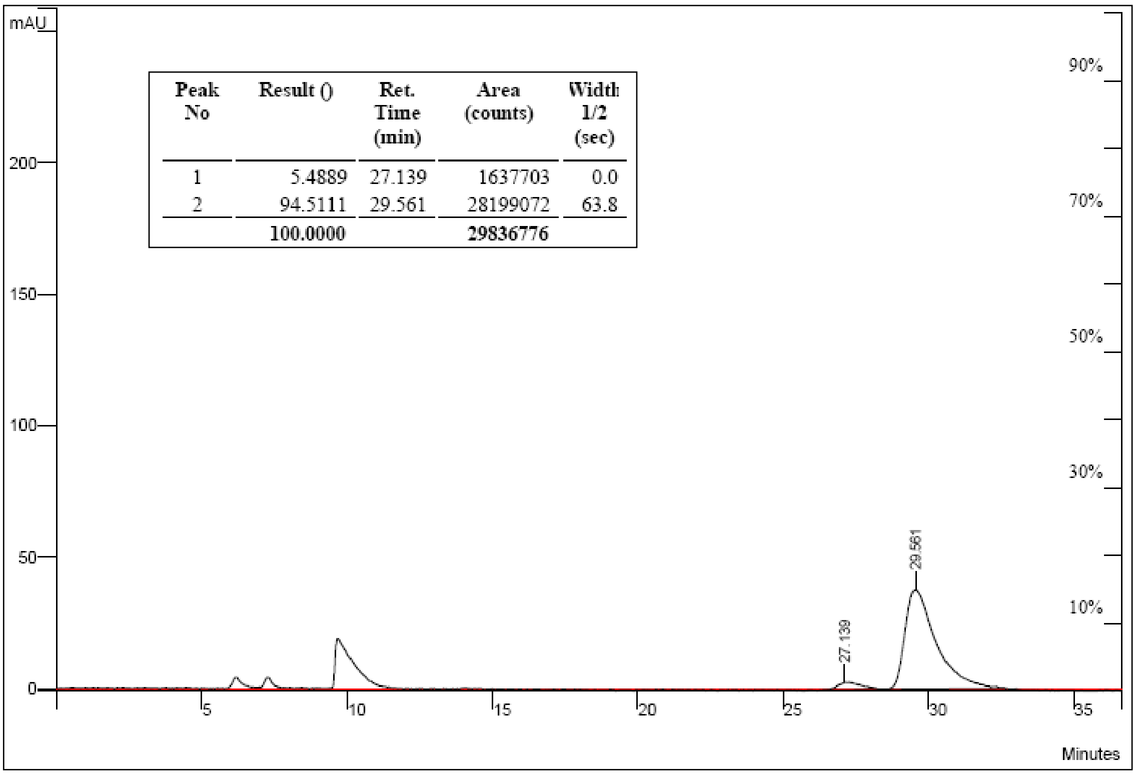

Peak No	Result ()	Ret. Time (min)	Area (counts)	Width 1/2 (sec)
1	5.4889	27.139	1637703	0.0
2	94.5111	29.561	28199072	63.8
	**100.0000**		**29836776**	

^{1}H NMR spectrum of the silylation reaction mixture of 2q after 52.8% conversion**

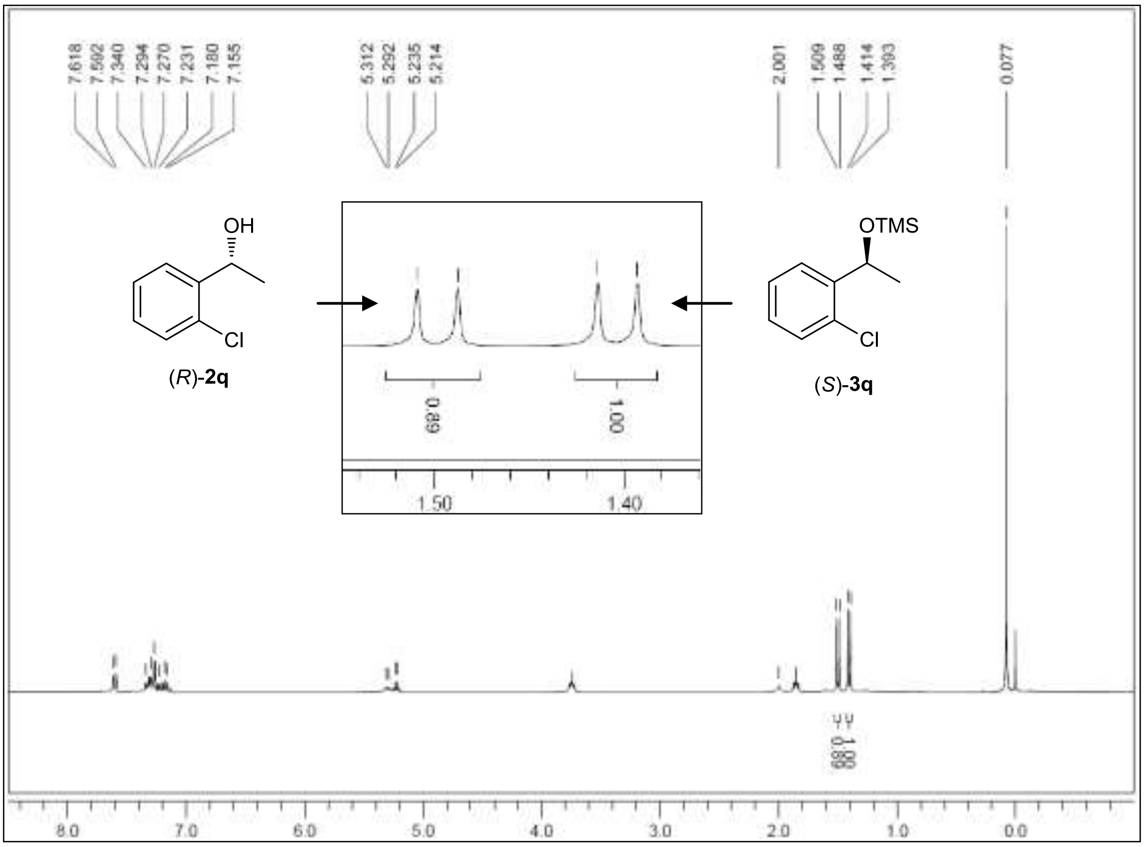

HPLC spectrum of *rac*-**2q** (Chiralcel OB-H, Hexane/IPA = 95/5, 0.5 ml/min, 220 nm)

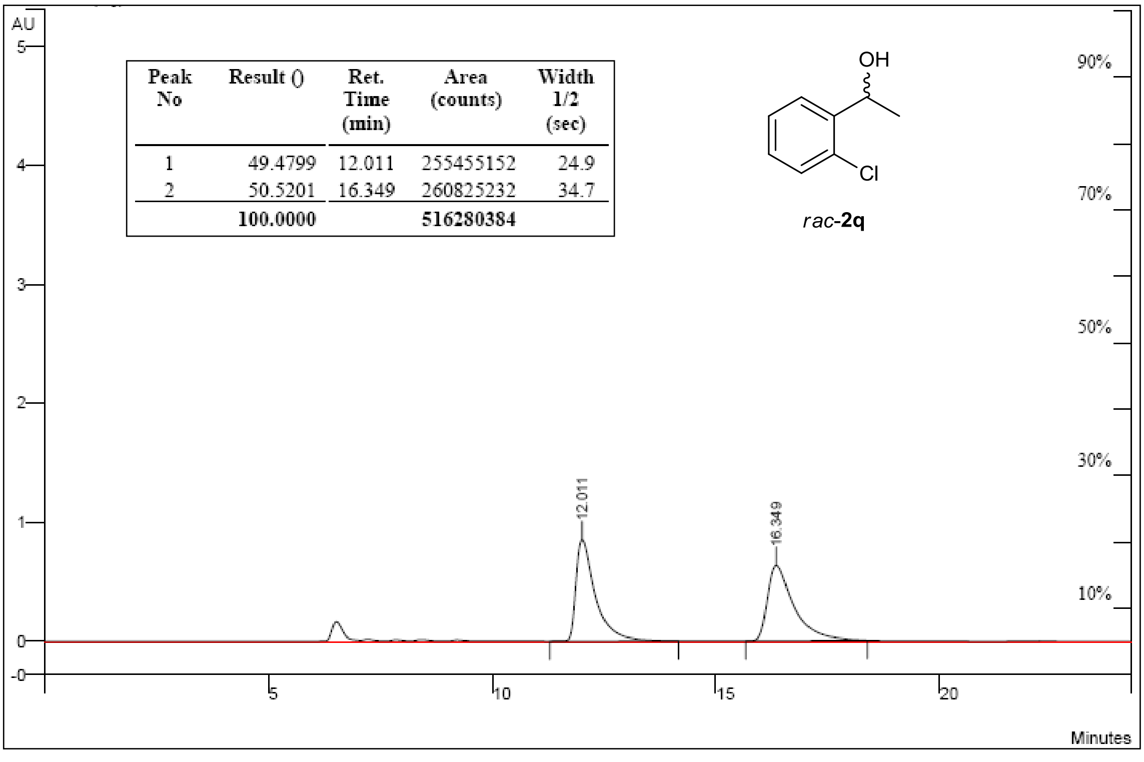

Peak No	Result ()	Ret. Time (min)	Area (counts)	Width 1/2 (sec)
1	49.4799	12.011	255455152	24.9
2	50.5201	16.349	260825232	34.7
	**100.0000**		**516280384**	

HPLC spectrum of the TMS-ether product (*S*)-**3q**

[Chiralcel OB-H, Hexane/IPA = 95/5, 0.5 ml/min, 220 nm]

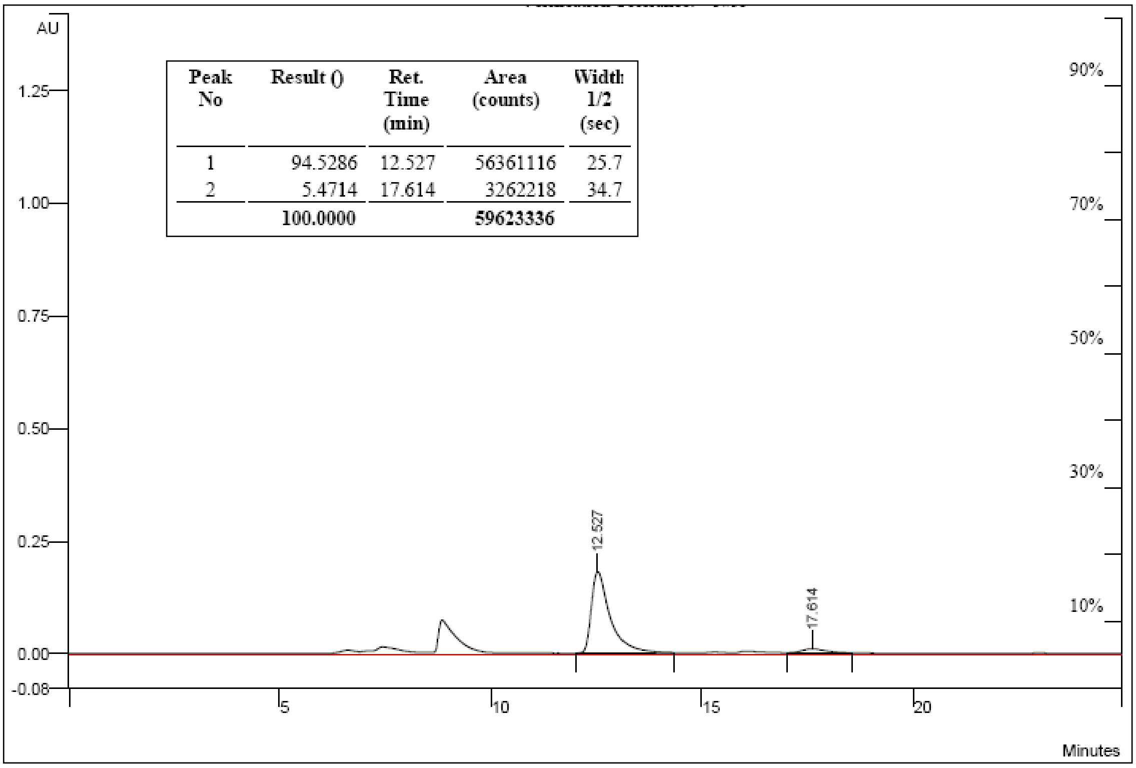

Peak No	Result ()	Ret. Time (min)	Area (counts)	Width 1/2 (sec)
1	94.5286	12.527	56361116	25.7
2	5.4714	17.614	3262218	34.7
	**100.0000**		**59623336**	

HPLC spectrum of the remaining alcohol (*R*)-**2q**

[Chiralcel OB-H, Hexane/IPA = 95/5, 0.5 ml/min, 220 nm]

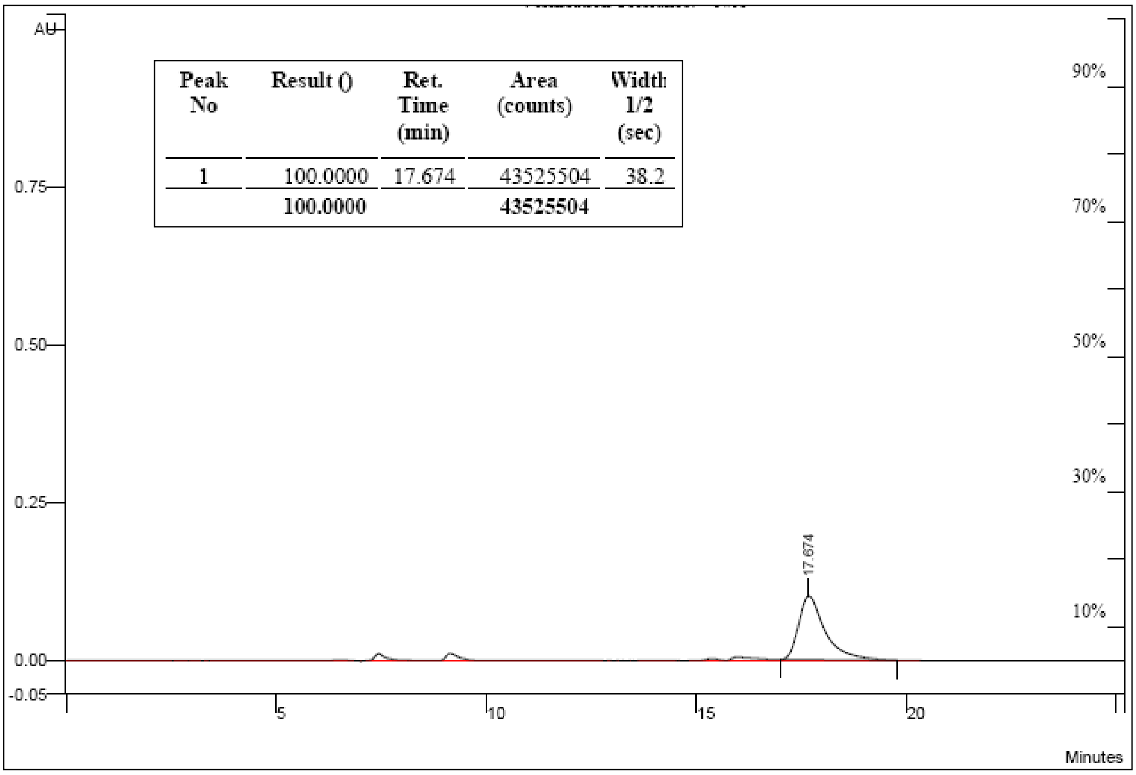

Peak No	Result ()	Ret. Time (min)	Area (counts)	Width 1/2 (sec)
1	100.0000	17.674	43525504	38.2
	**100.0000**		**43525504**	

^{1}H NMR spectrum of the silylation reaction mixture of 2r after 54.9% conversion

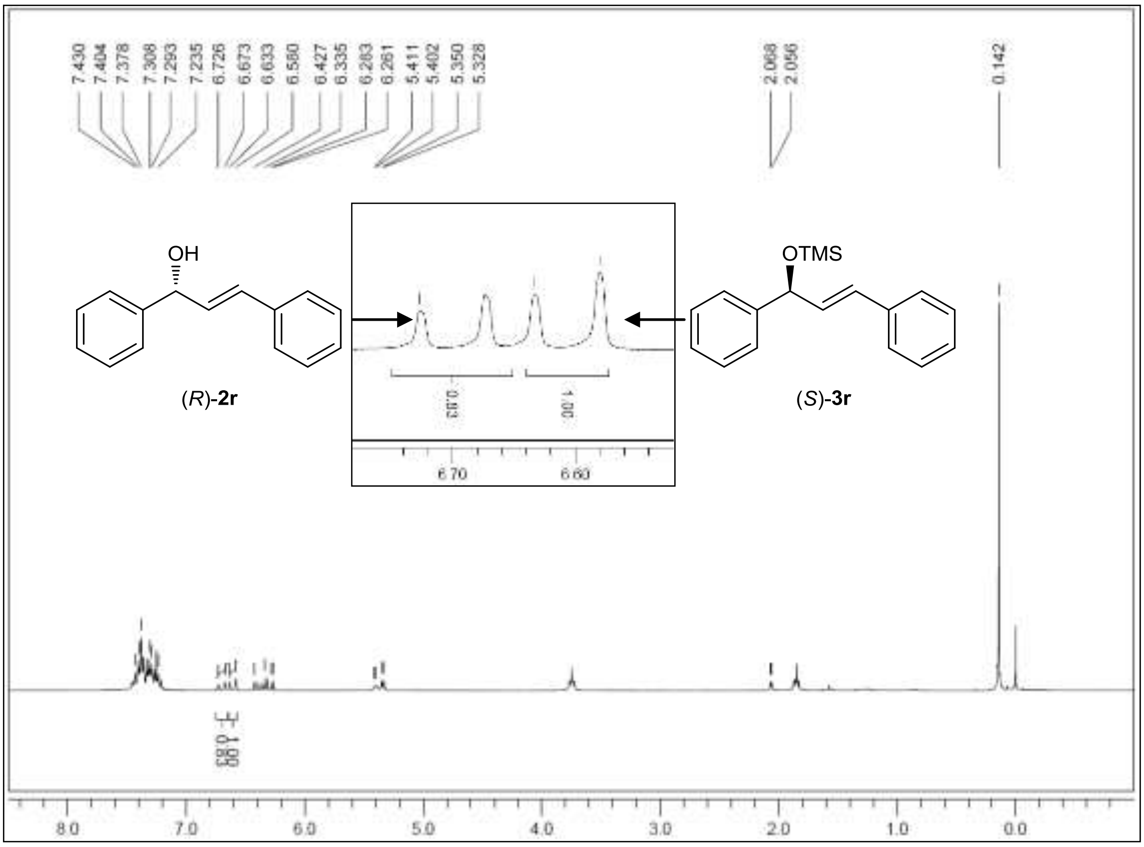

HPLC spectrum of *rac*-**2r** (Chiralcel OD-H, Hexane/IPA = 90/10, 1.0 ml/min, 220 nm)

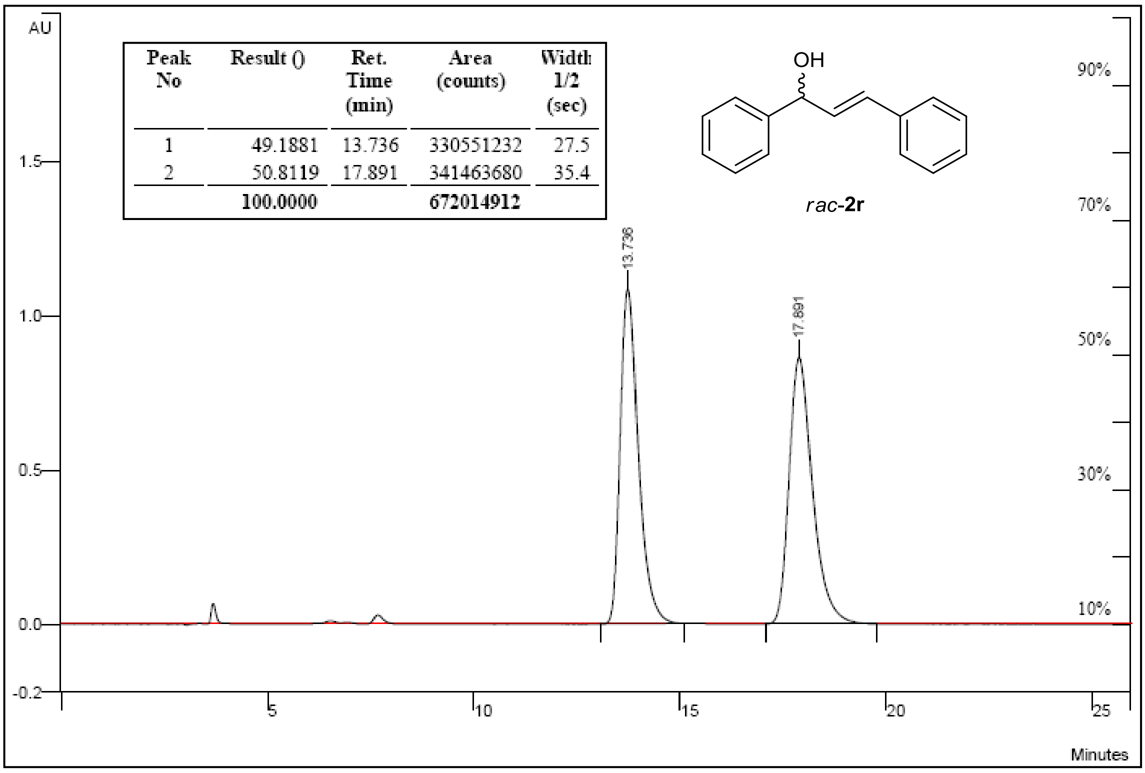

Peak No	Result ()	Ret. Time (min)	Area (counts)	Width 1/2 (sec)
1	49.1881	13.736	330551232	27.5
2	50.8119	17.891	341463680	35.4
	**100.0000**		**672014912**	

HPLC spectrum of the TMS-ether product (*S*)-**3r**

[Chiralcel OD-H, Hexane/IPA = 90/10, 1.0 ml/min, 220 nm]

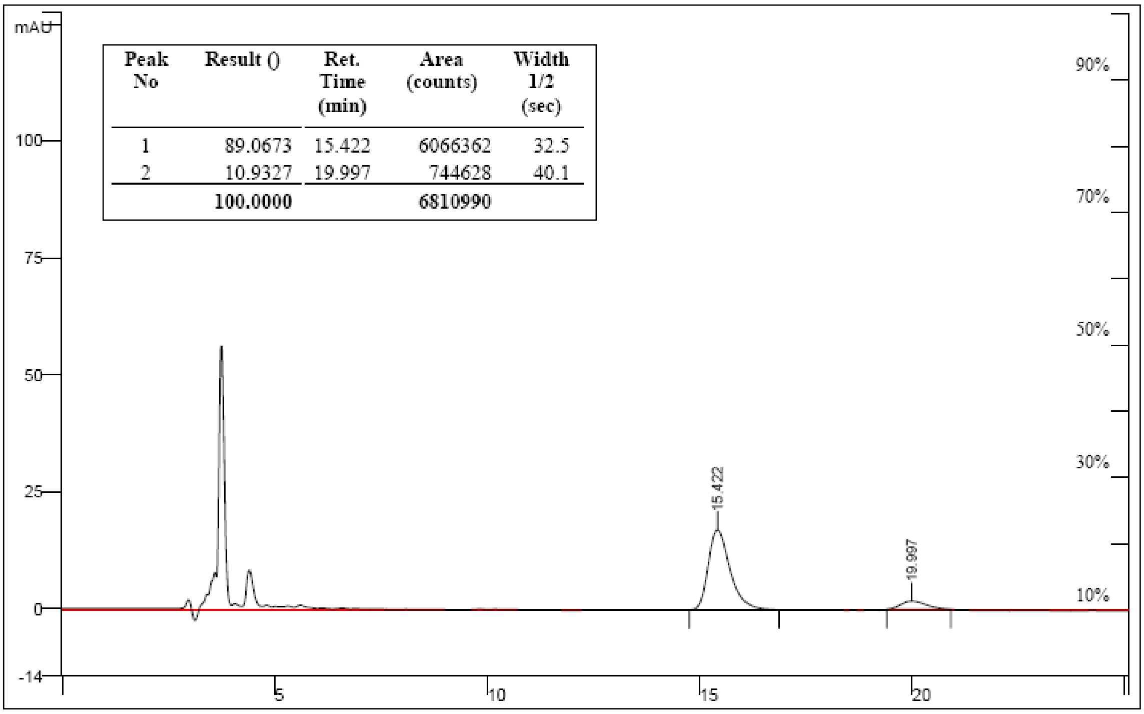

Peak No	Result ()	Ret. Time (min)	Area (counts)	Width 1/2 (sec)
1	89.0673	15.422	6066362	32.5
2	10.9327	19.997	744628	40.1
	**100.0000**		**6810990**	

HPLC spectrum of the remaining alcohol (*R*)-**2r**

[Chiralcel OD-H, Hexane/IPA = 90/10, 1.0 ml/min, 220 nm]

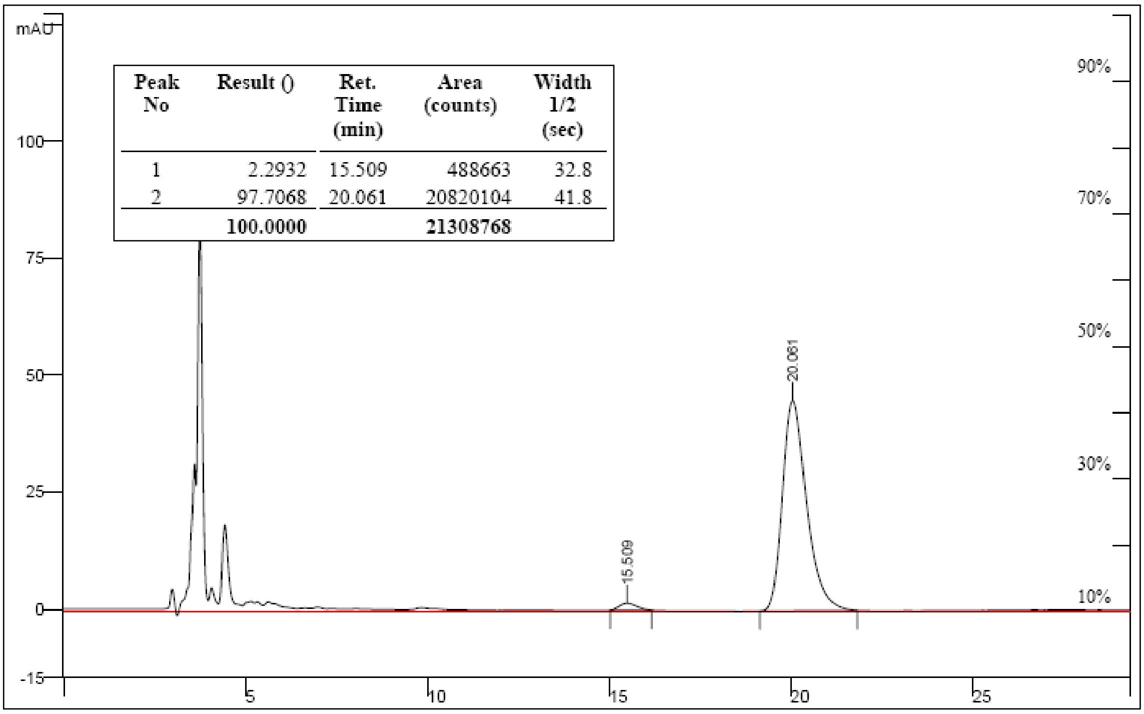

Peak No	Result ()	Ret. Time (min)	Area (counts)	Width 1/2 (sec)
1	2.2932	15.509	488663	32.8
2	97.7068	20.061	20820104	41.8
	**100.0000**		**21308768**	

Supplementary Figure 19. ^{1}H NMR and HPLC Spectra for Figure **5a**

^{1}H NMR spectrum of the silylation reaction mixture of 2a after 45.0% conversion

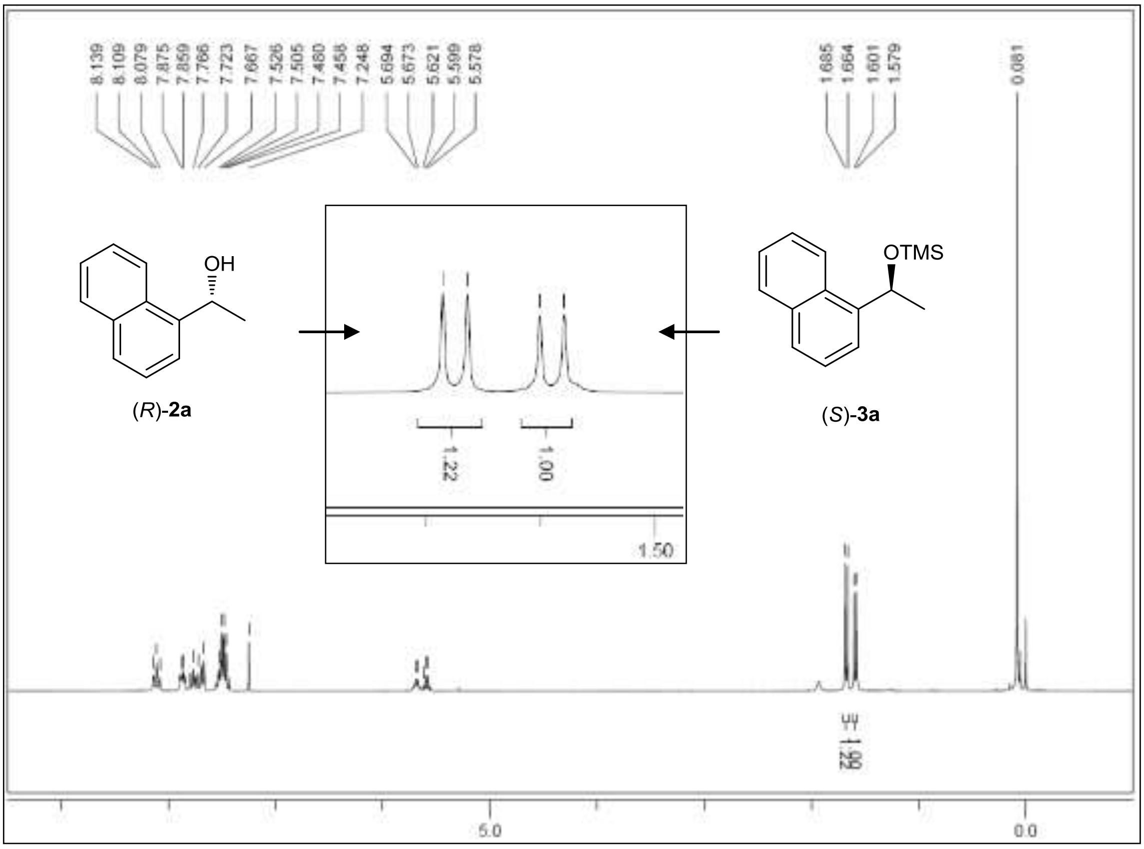

HPLC spectrum of *rac*-**2a** (Chiralcel OD-H, Hexane/IPA = 90/10, 0.7 ml/min, 220 nm)

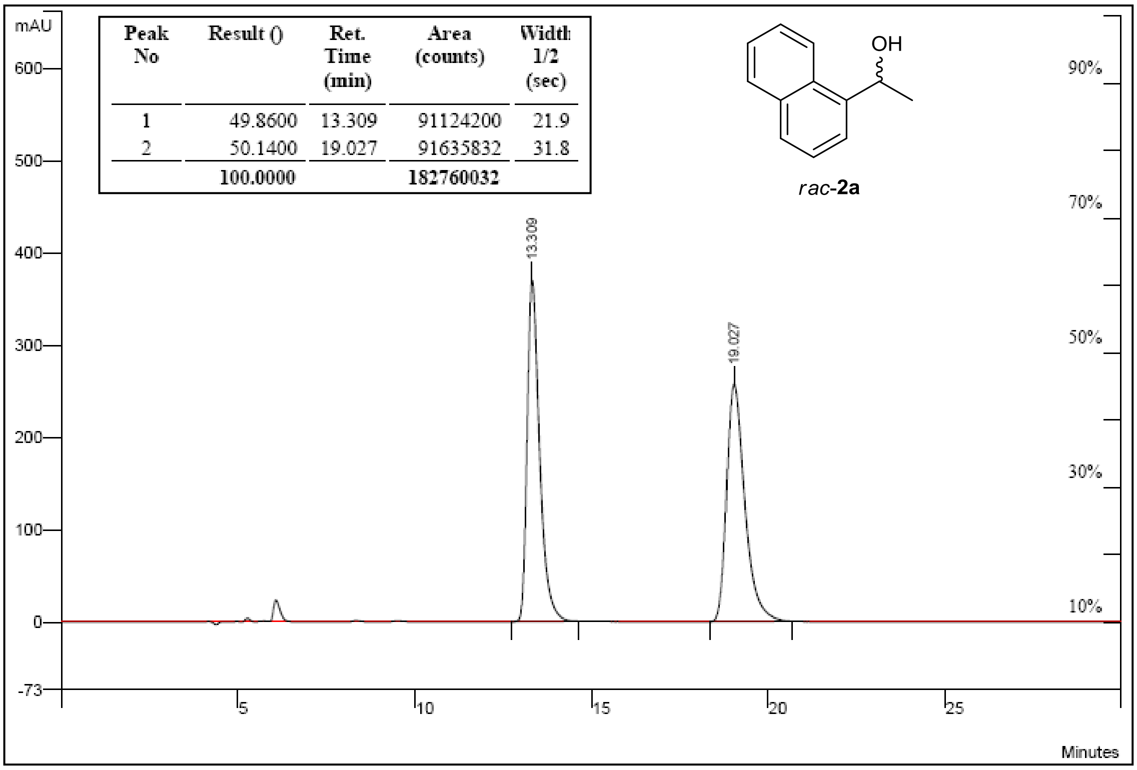

Peak No	Result ()	Ret. Time (min)	Area (counts)	Width 1/2 (sec)
1	49.8600	13.309	91124200	21.9
2	50.1400	19.027	91635832	31.8
	100.0000		182760032	

HPLC spectrum of the TMS-ether product (*S*)-**3a**

[Chiralcel OD-H, Hexane/IPA = 90/10, 0.7 ml/min, 220 nm]

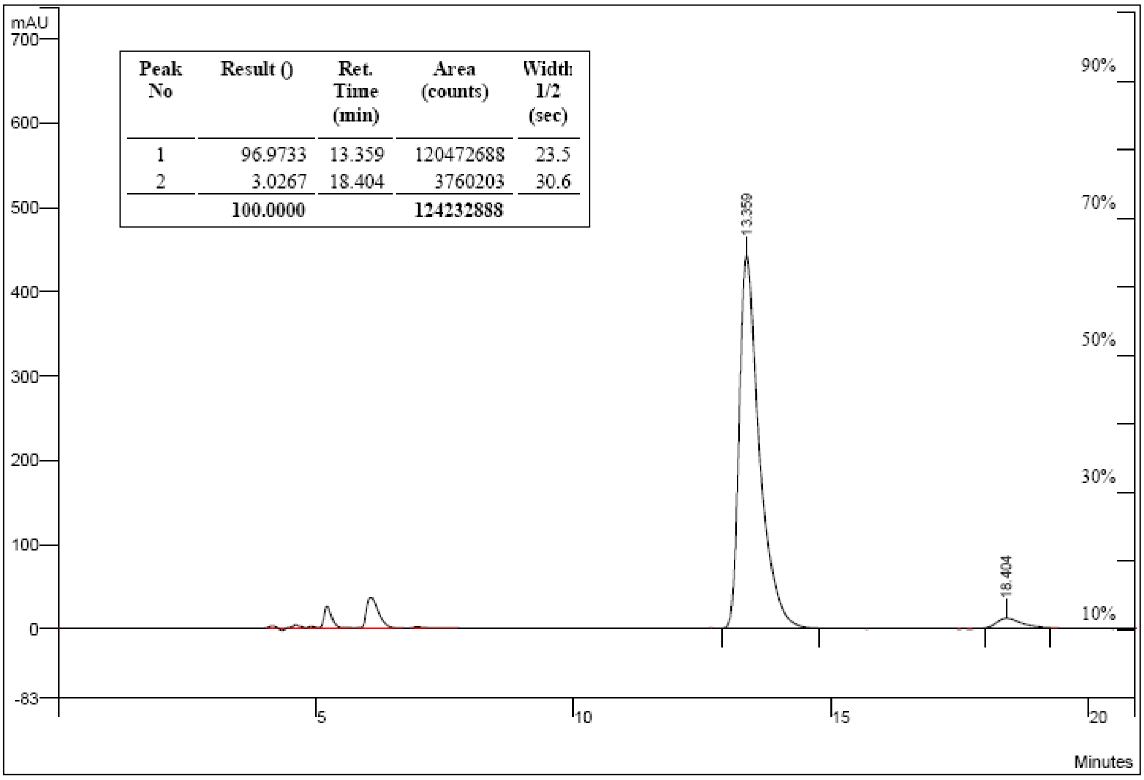

HPLC spectrum of the remaining alcohol (*R*)-**2a**

[Chiralcel OD-H, Hexane/IPA = 90/10, 0.7 ml/min, 220 nm]

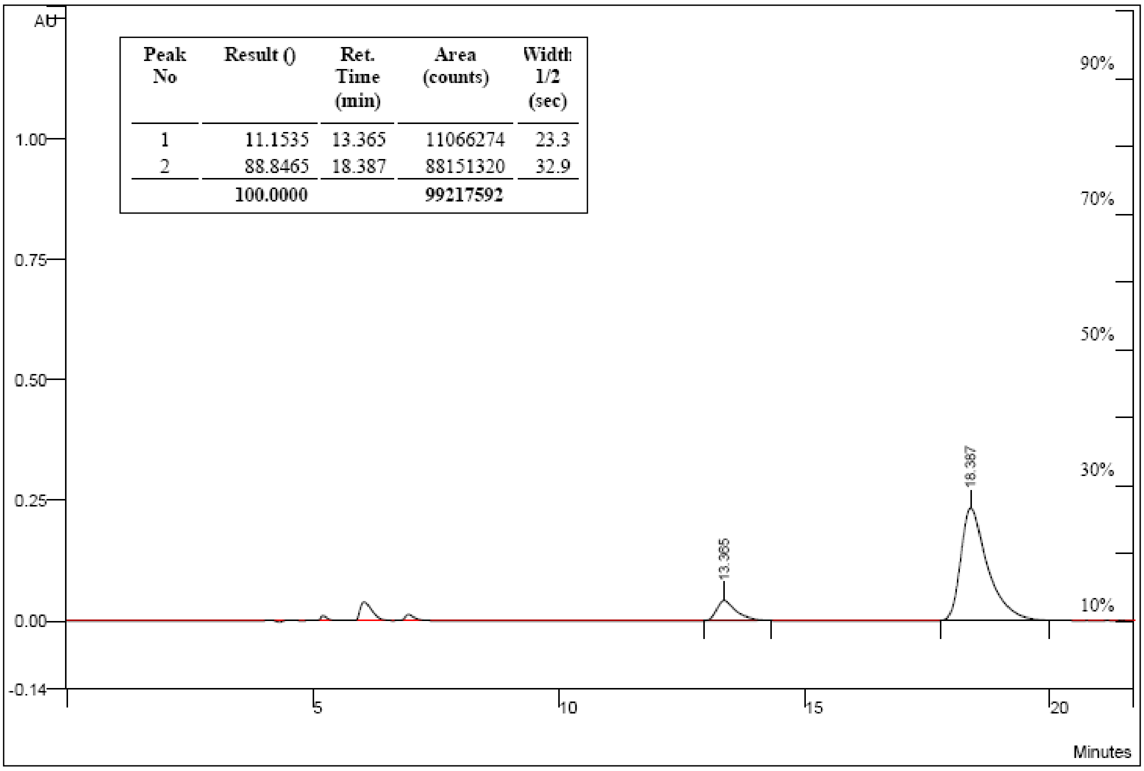

Supplementary Figure 20. ^{1}H NMR and HPLC Spectra for Figure **5b**

^{1}H NMR spectrum of the silylation reaction mixture of 4a after 95% conversion

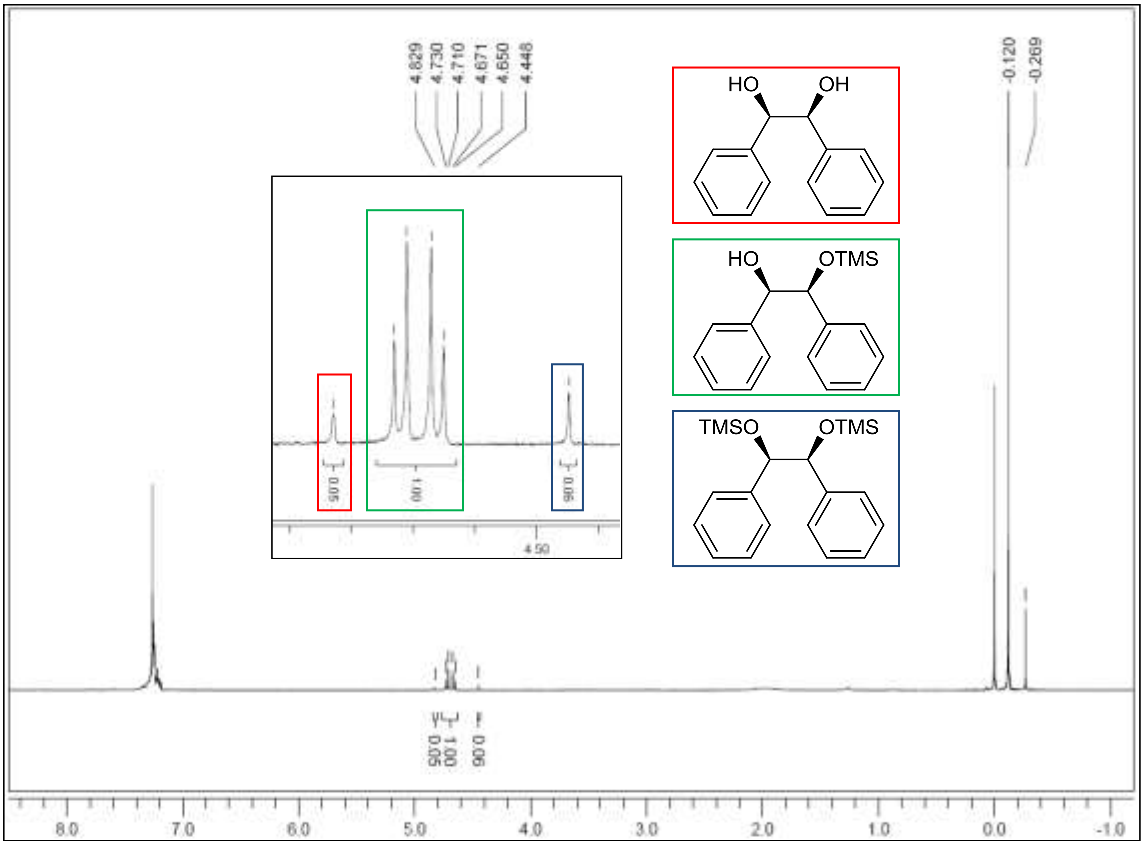

HPLC spectrum of *rac*-**5a** (Chiralcel OD-H, Hexane/IPA = 98/2, 1.0 ml/min, 220 nm)

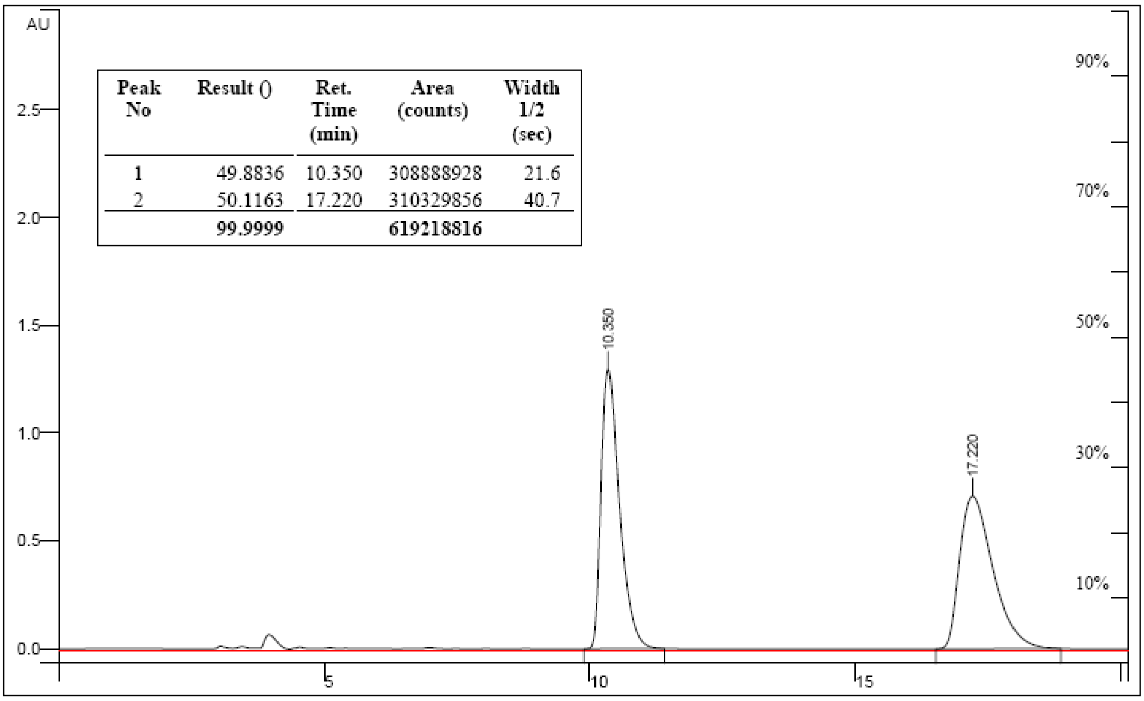

Peak No	Result ()	Ret. Time (min)	Area (counts)	Width 1/2 (sec)
1	49.8836	10.350	308888928	21.6
2	50.1163	17.220	310329856	40.7
	**99.9999**		**619218816**	

HPLC spectrum of the TMS-ether product **5a**

[Chiralcel OD-H, Hexane/IPA = 98/2, 1.0 ml/min, 220 nm]

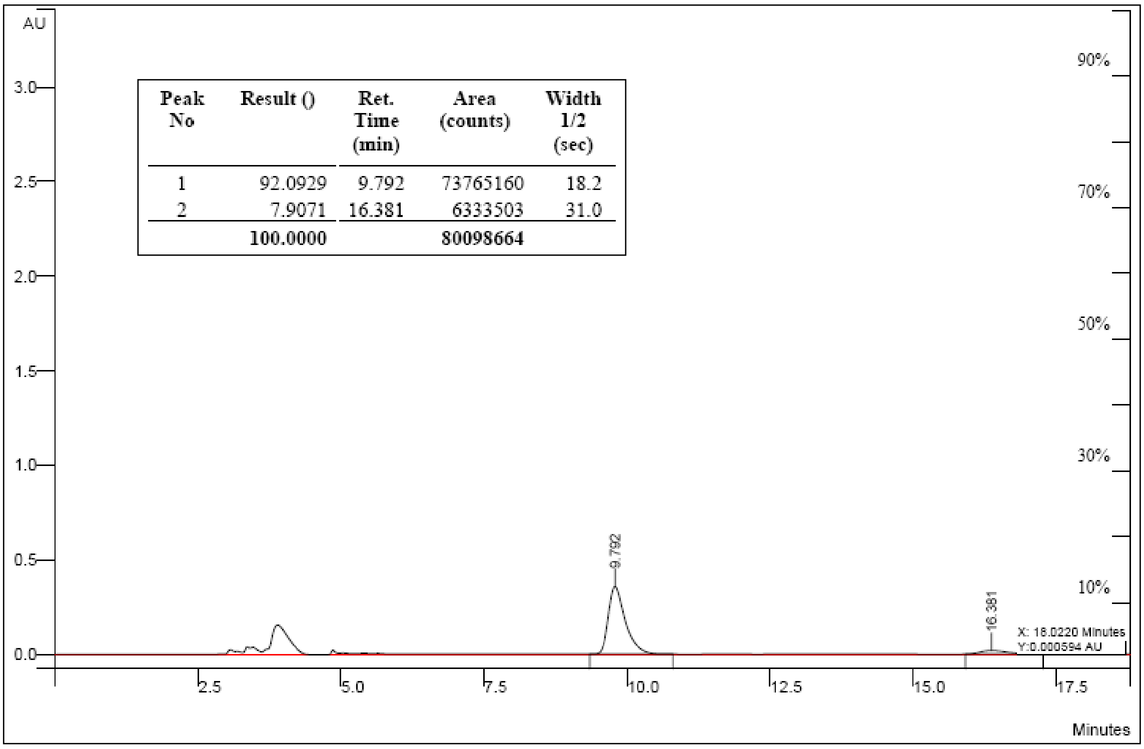

Peak No	Result ()	Ret. Time (min)	Area (counts)	Width 1/2 (sec)
1	92.0929	9.792	73765160	18.2
2	7.9071	16.381	6333503	31.0
	100.0000		80098664	

^{1}H NMR spectrum of the silylation reaction mixture of 4b after 92% conversion

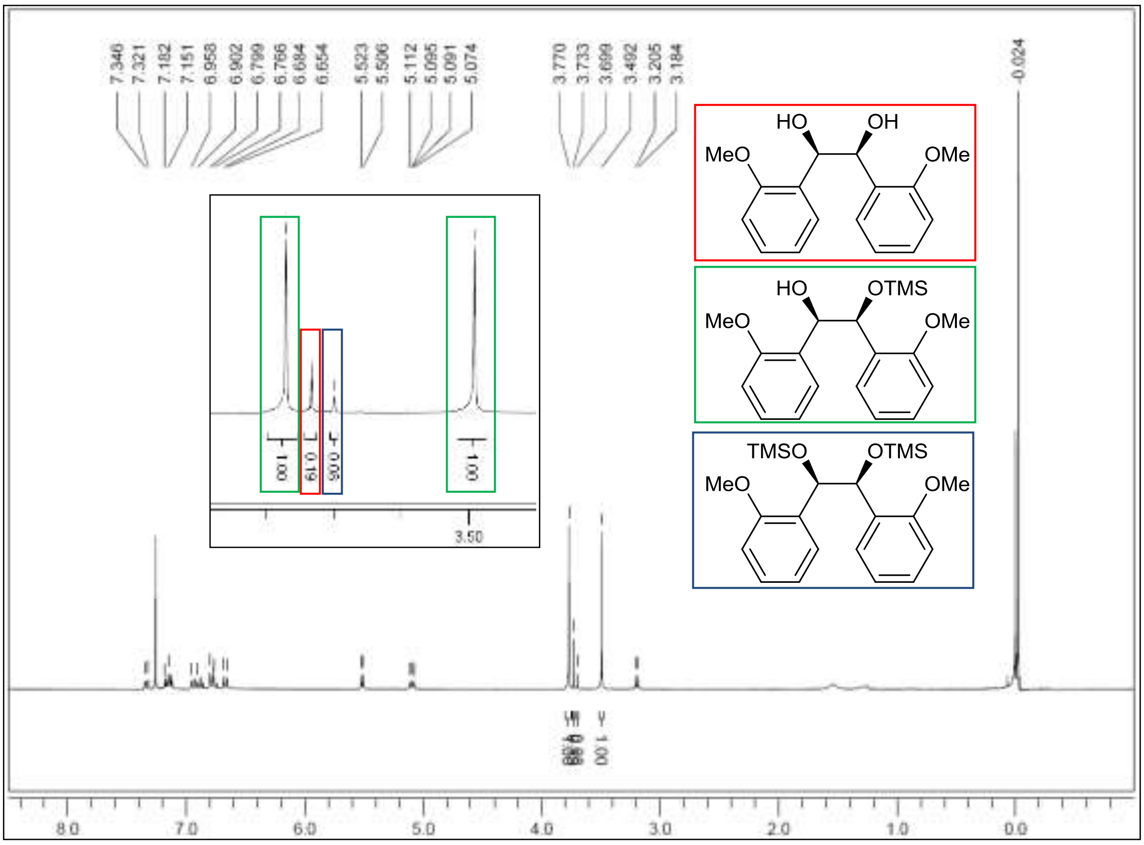

HPLC spectrum of *rac*-**5b**

[Chiralcel OD-H, Hexane/IPA = 98/2, 1.0 ml/min, 220 nm]

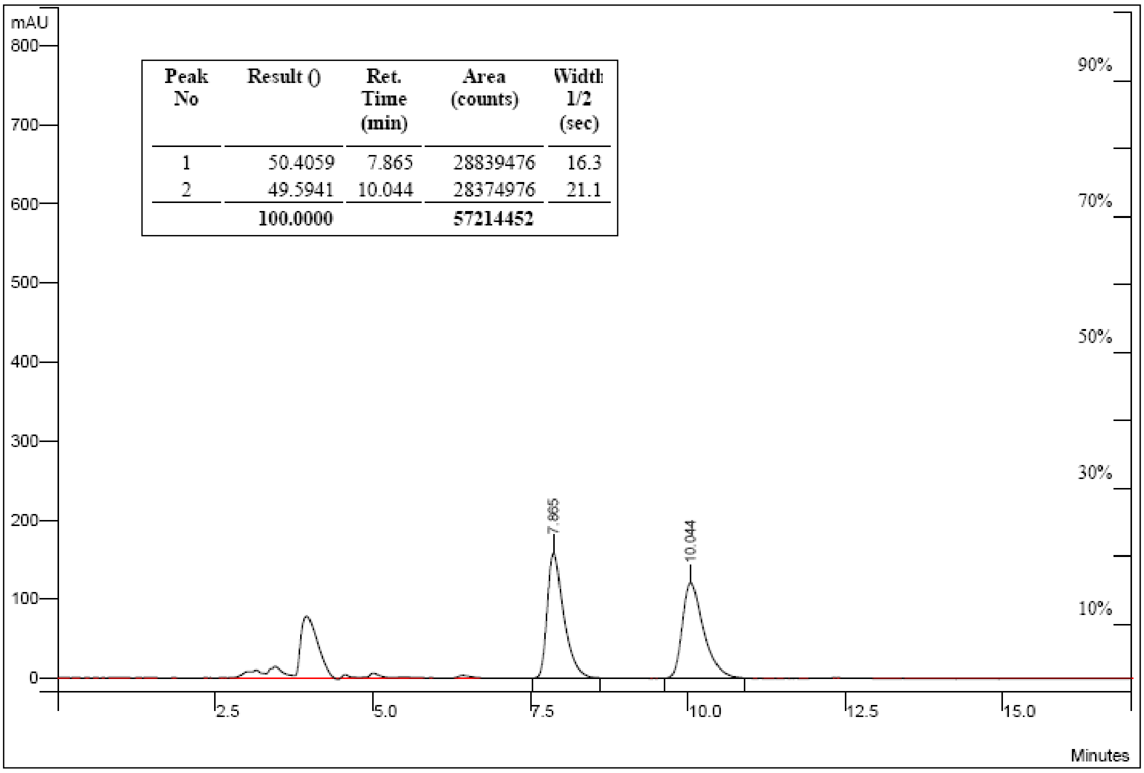

Peak No	Result ()	Ret. Time (min)	Area (counts)	Width 1/2 (sec)
1	50.4059	7.865	28839476	16.3
2	49.5941	10.044	28374976	21.1
	**100.0000**		**57214452**	

HPLC spectrum of the TMS-ether product **5b**

[Chiralcel OD-H, Hexane/IPA = 98/2, 1.0 ml/min, 220 nm]

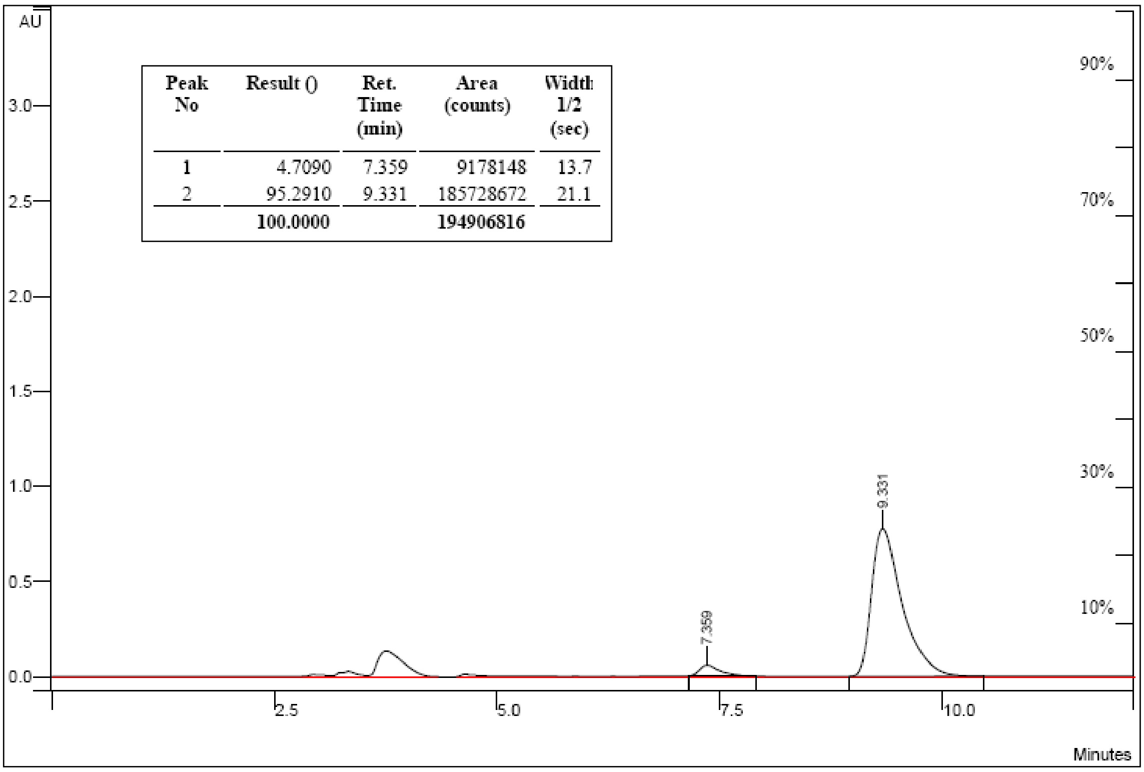

Peak No	Result ()	Ret. Time (min)	Area (counts)	Width 1/2 (sec)
1	4.7090	7.359	9178148	13.7
2	95.2910	9.331	185728672	21.1
	**100.0000**		**194906816**	

^{1}H NMR spectrum of the silylation reaction mixture of 4c after 91% conversion at 20°C

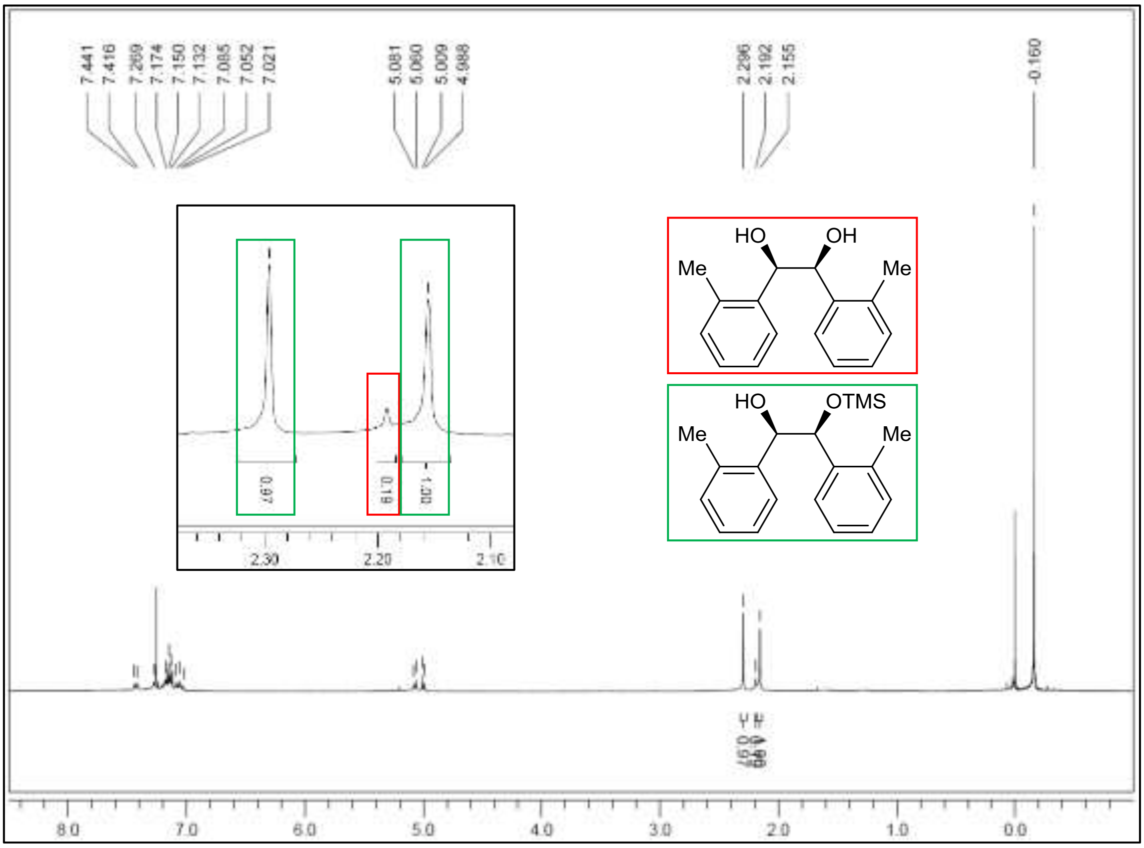

HPLC spectrum of *rac*-**5c**

[Chiralcel OD-H, Hexane/IPA = 98/2, 1.0 ml/min, 220 nm]

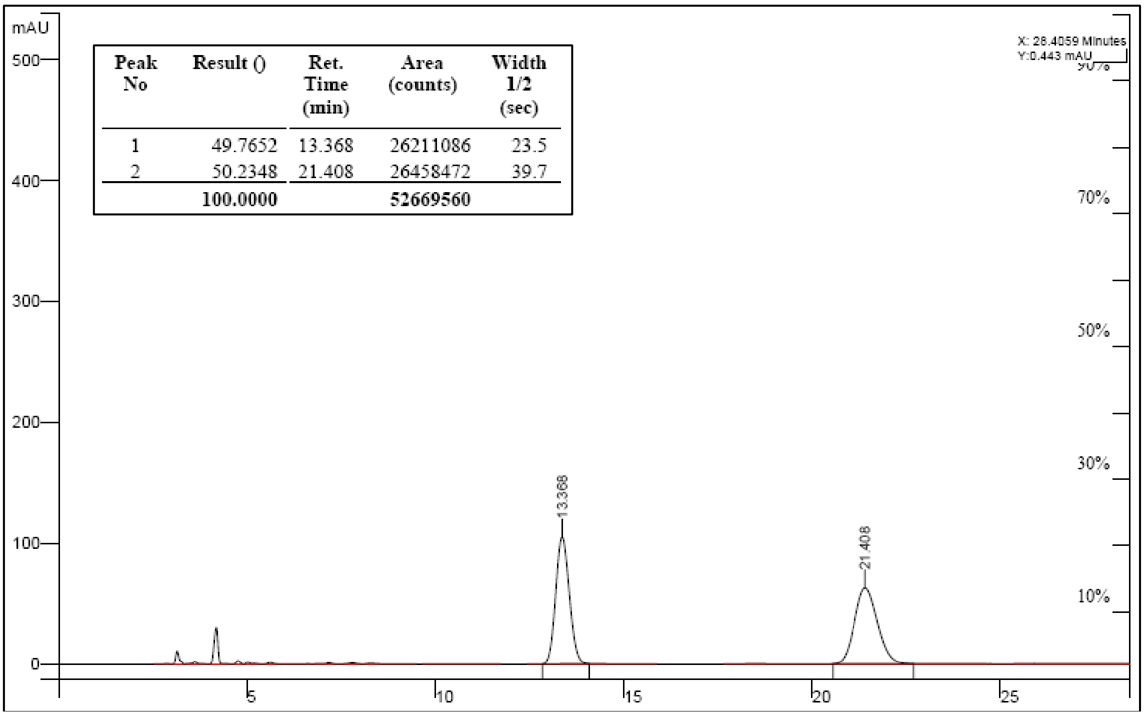

Peak No	Result ()	Ret. Time (min)	Area (counts)	Width 1/2 (sec)
1	49.7652	13.368	26211086	23.5
2	50.2348	21.408	26458472	39.7
	**100.0000**		**52669560**	

HPLC spectrum of the TMS-ether product **5c**

[Chiralcel OD-H, Hexane/IPA = 98/2, 1.0 ml/min, 220 nm]

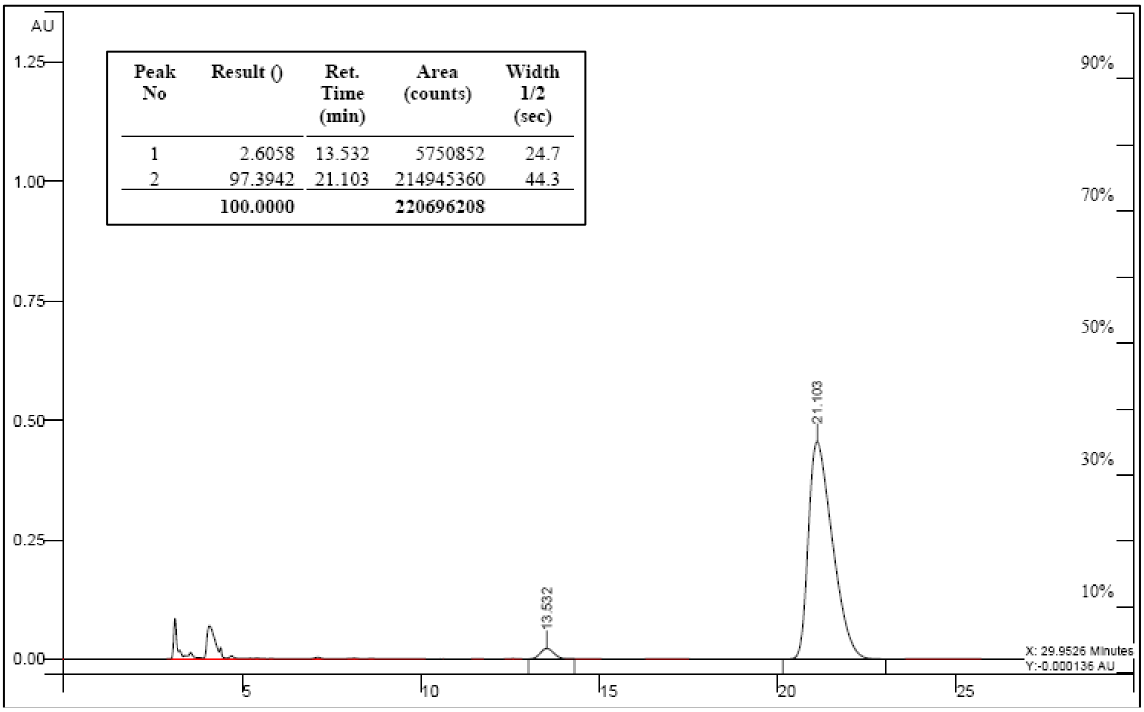

Peak No	Result ()	Ret. Time (min)	Area (counts)	Width 1/2 (sec)
1	2.6058	13.532	5750852	24.7
2	97.3942	21.103	214945360	44.3
	100.0000		220696208	

^{1}H NMR spectrum of the silylation reaction mixture of 4c after 93% conversion at 0°C

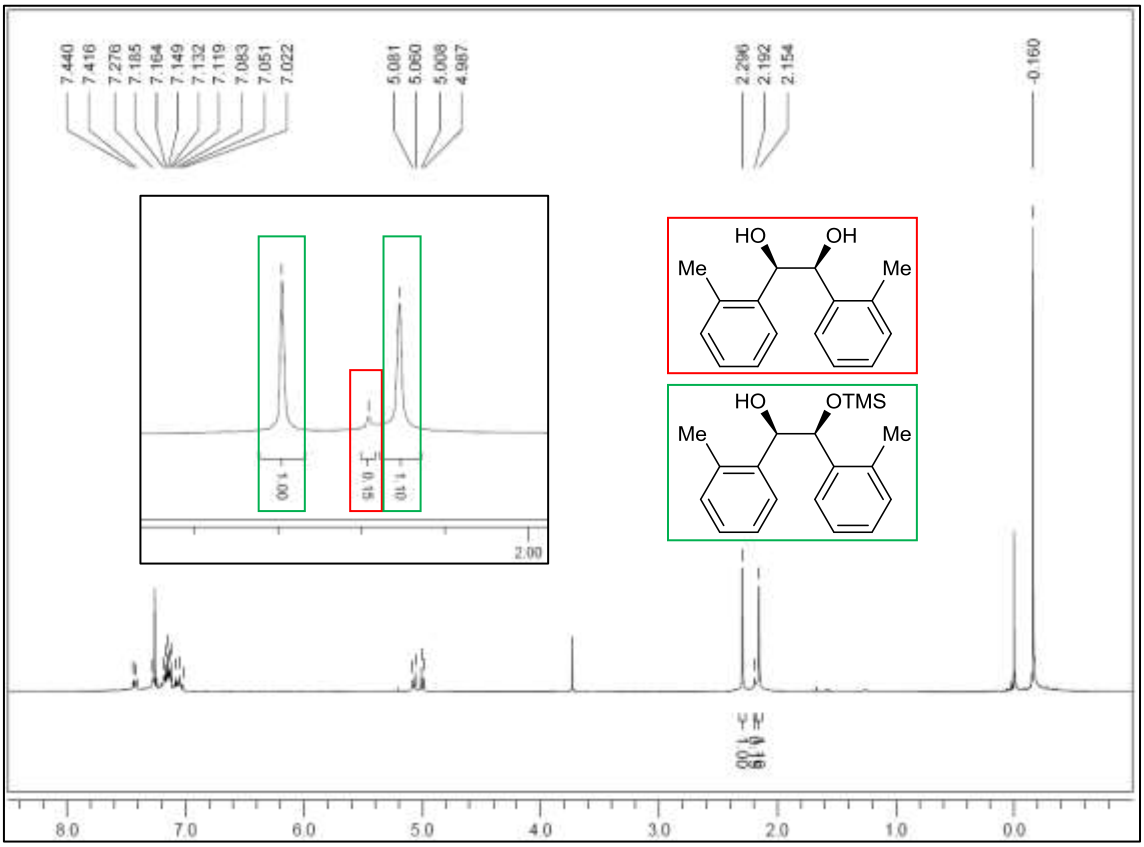

HPLC spectrum of the TMS-ether product **5c**

[Chiralcel OD-H, Hexane/IPA = 98/2, 1.0 ml/min, 220 nm]

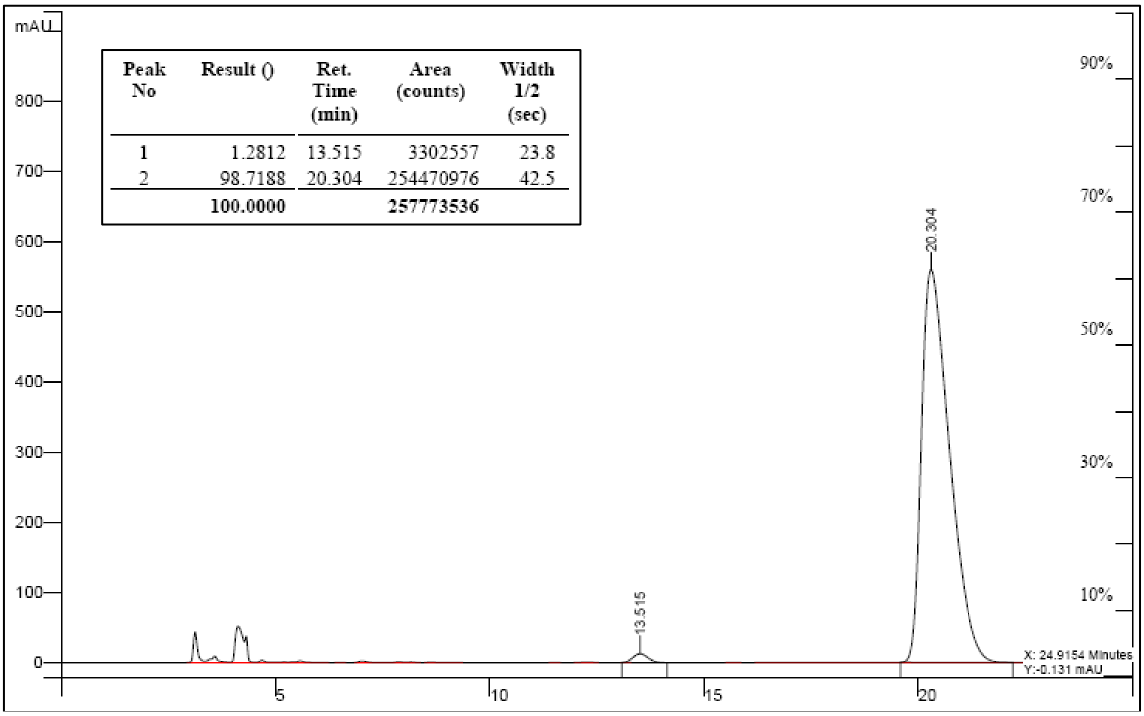

Supplementary Table

Supplementary Table 1. Silylating reagent screening

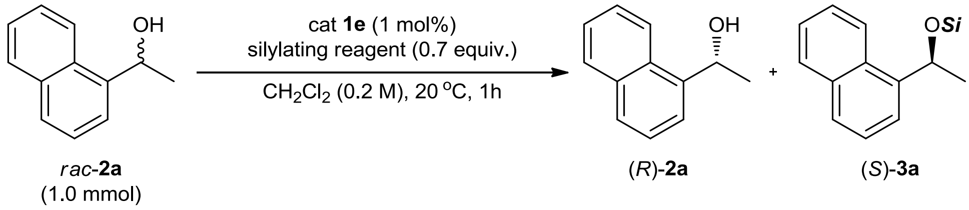

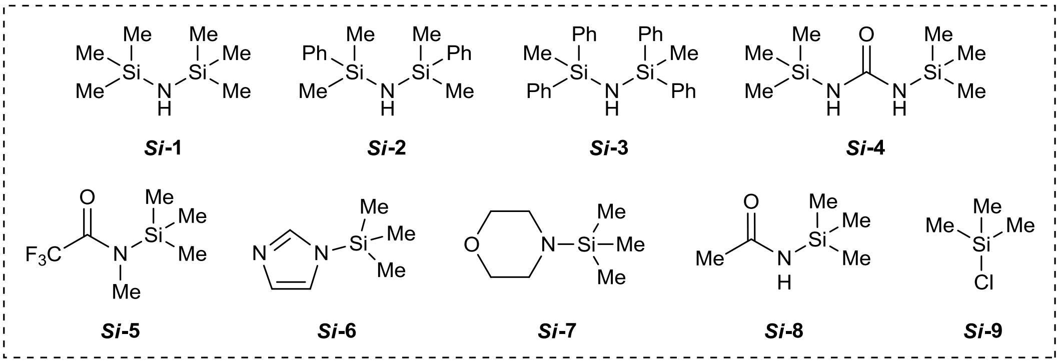

Entry	*Si*-reagent	Conv. (%)[a]	e.r. of (*R*)-**2a**	e.r. of (*S*)-**3a**	*s*[b]
1	***Si*-1**	48.5	90:10	85:15	41.1
2*	***Si*-1**	28.2 (after 24 h)	68.5:31.5	97:3	46.4
3	***Si*-2**	26.3	65:35	92:8	15.4
4	***Si*-3**	n.r.	-	-	-
5	***Si*-4**	1.5	-	racemic	n.d.
6	***Si*-5**	28.6	racemic	racemic	n.d.
7	***Si*-6**	19.8	racemic	racemic	n.d.
8	***Si*-7**	15.4	52:48	61:39	1.6
9	***Si*-8**	4.0	racemic	racemic	n.d.
10	***Si*-9**	n.r.	-	-	-

[a] Determined by ^{1}H-NMR analysis of the unpurified reaction mixture. [b] $s = \ln[1-\text{conv.}(1+ee_{(S)\text{-}3a})] / \ln[1-\text{conv.}(1-ee_{(S)\text{-}3a})]$. *0.25 equiv. of ***Si*-1** was used. n.d., not determined. e.r., enantiomeric ratio.

Comments: We observed reasonable conversion and enantioselectivity only with HMDS (***Si*-1**, Supplementary Table 1). Most of silylating reagents are not capable of producing silyl-protected alcohols in the presence of catalyst **1e**.

Supplementary Table 2. Solvent screening

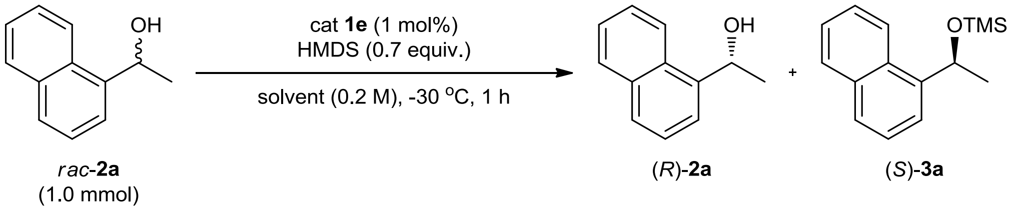

Entry	Solvent	Conv. (%)[a]	e.r. of (*R*)-**2a**	e.r. of (*S*)-**3a**	*s*[b]
1	CH_2Cl_2	51.6	98:2	95:5	89.1
2	Toluene	35.3	74:26	95:5	30.9
3	THF	5.1	52:48	97:3	29.0
4	Et_2O	12.8	56:44	91:9	11.4
5	DCE	53.0	99:1	93:7	66.1

[a] Determined by ^{1}H-NMR analysis of the unpurified reaction mixture. [b] s = ln[1−conv.(1+ee$_{(S)\text{-}3a}$] / ln[1−conv.(1−ee$_{(S)\text{-}3a}$]. e.r., enantiomeric ratio.

Comments: We observed lower enantioselecivity in the case of ether-based solvents, which can be as ascribed to the competitive interactions of solvents and the polyether-based catalyst. In non-polar solvents, superior enantioselectivities were obtained under identical reaction conditions.

Supplementary Table 3. Concentration effect

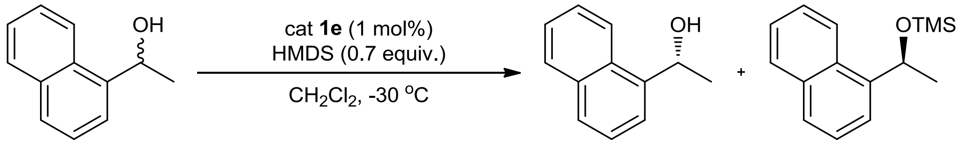

Entry	Conc. (M)	Time (min)	Conv. (%)[a]	e.r. of (*R*)-**2a**	e.r. of (*S*)-**3a**	*s*[b]
1	0.1	90	52.4	98.5:1.5	94:6	66.1
2	0.2	60	51.6	98:2	95:5	89.1
3	0.4	30	51.1	97.5:2.5	95.5:4.5	79.2

[a] Determined by ^{1}H-NMR analysis of the unpurified reaction mixture. [b] $s = \ln[1-\text{conv.}(1+\text{ee}_{(S)\text{-3a}}] / \ln[1-\text{conv.}(1-\text{ee}_{(S)\text{-3a}}]$. e.r., enantiomeric ratio.

Supplementary Table 4. ^{13}C Spin-lattice relaxation measurements (catalyst 1e)

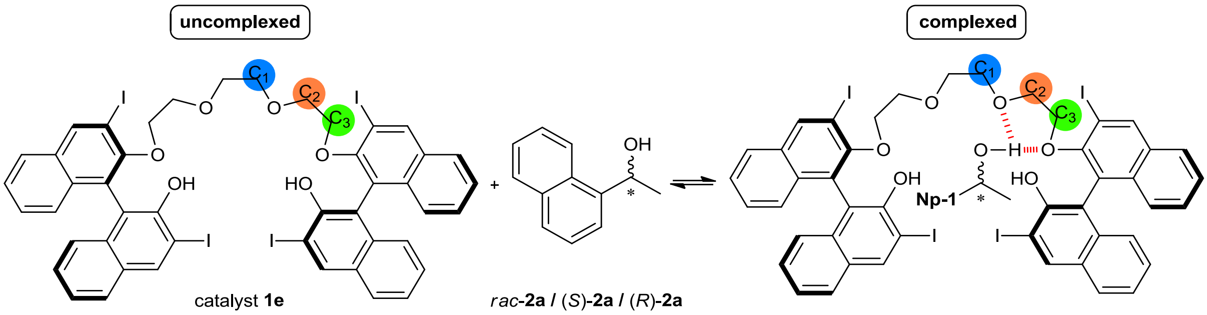

Sample (ratio of cat **1e** : **2a** = 1 : 1)	T_1 [s]			T_1(uncompl.)/T_1(compl.)		
	C_1	C_2	**C_3**	C_1	C_2	**C_3**
cat **1e**	0.364	0.397	**0.604**	-	-	-
cat **1e** & (*R*)-**2a**	0.396	0.492	**0.505**	0.91	0.80	**1.19**
cat **1e** & *rac*-**2a**	0.403	0.424	**0.488**	0.90	0.94	**1.24**
cat **1e** & (*S*)-**2a**	0.352	0.416	**0.408**	1.03	0.95	**1.48**

All NMR experiments were carried out in $CDCl_3$ at 22 ^{o}C, [**1e**] = 0.04 M.

Comments: To verify the catalyst-secondary alcohol interactions, we conducted ^{13}C Spin-lattice relaxation measurements. A significant decrease in the T_1 value of C_3 was observed upon complexation of catalyst **1e** with alcohols **2a**. This significant decrease in the T_1 value of C_3 indicates that the complexation of the alcohol with catalyst **1e** significantly reduces the mobility of the ether units. Furthermore, the fast reacting (*S*)-configured substrate ((*S*)-**2a**) gave more decreased T_1 value of C_3 than that of the slow reacting (*R*)-**2a**, directly indicating a selective association of the catalyst with the fast reacting (*S*)-**2a**

Supplementary Table 5. [13]C Spin-lattice relaxation measurements (catalyst 1m)

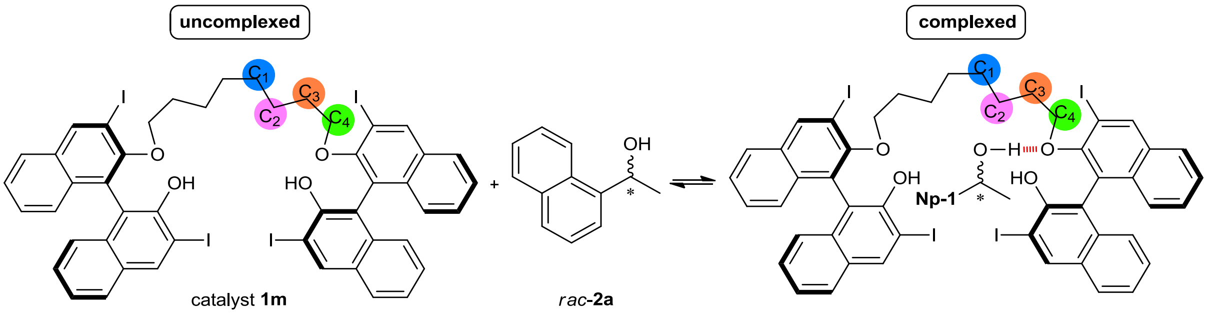

Sample (ratio of cat **1m** : **2a** = 1 : 1)	T_1 [s]				T_1(uncompl.)/T_1(compl.)			
	C_1	C_2	C_3	**C_4**	C_1	C_2	C_3	**C_4**
cat **1m**	0.529	0.446	0.389	0.339	-	-	-	-
cat **1m** & *rac*-**2a**	0.507	0.453	0.418	0.337	1.04	0.99	0.93	1.01

All NMR experiments were carried out in $CDCl_3$ at 22 °C, [**1e**] = 0.075 M.

Comments: No change of T_1 value was observed when the alcohol **2a** was mixed with **1m** in which ether chain was replaced with the alkyl chain, verifying the crucial role of the polyether backbone.

Supplementary Table 6. ^{29}Si NMR data of HMDS in the presence of diverse phenols

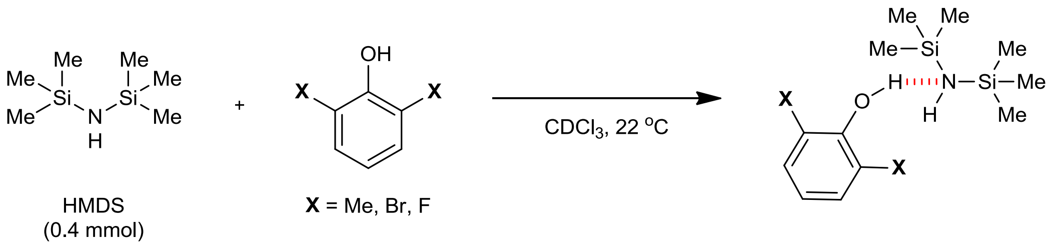

Sample	^{29}Si chemical shift, ppm* (Δυ in Hz)	
	HMDS : phenol = 1 : 1 (molar ratio)	HMDS : phenol = 1 : 5
HMDS	2.457	2.457
HMDS & 2,6-dimethylphenol	2.475 (1.8 Hz)	2.511 (5.4 Hz)
HMDS & 2,6-dibromophenol	2.479 (2.2 Hz)	2.539 (8.2 Hz)
HMDS & 2,6-difluorophenol	2.545 (8.8 Hz)	n.d.*

*Using tetramethysilane as an internal standard. [HMDS] = 0.4 M. n.d. = not determined; Under this condition, 2,6-difluorophenol was silylated by HMDS very fast.

Comments: ^{29}Si NMR experiments were used to establish the interaction between phenolic proton of the catalyst and HMDS. As shown in Supplementary Table 6, ^{29}Si NMR data of HMDS in the presence of diverse phenols exhibit ^{29}Si chemical shifts that are shifted downfield relative to the uncoordinated HMDS, depending on the acidity of phenols. This result clearly indicates that the acidic phenolic proton interacts with HMDS, enhancing the electrophilicity of silicon atom.

Supplementary Table 7. Steric demand of 3,3'-substituents of the catalysts

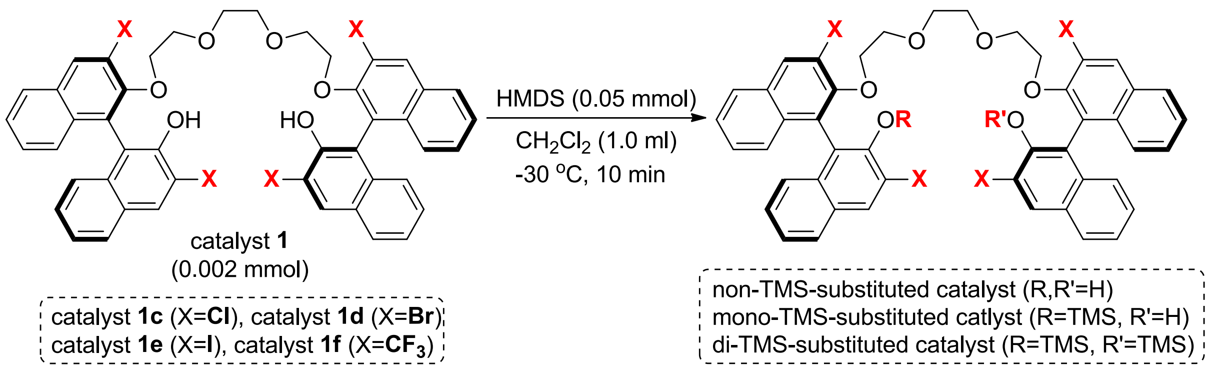

		Ratio (%)*		
Entry	Catalyst	mono-TMS	di-TMS	non-TMS
1	**1c** (X=Cl)	9	64	27
2	**1d** (X=Br)	31	32	37
3	**1e** (X=I)	27	20	53
4	**1f** (X=CF_3)	18	39	43

*Determined by ^{1}H-NMR analysis of the unpurified reaction mixture in $CDCl_3$ at 22 °C.

Comments: After observing the TMS-protected catalyst is inactive towards the silylation reaction, we conducted silylation reaction of various catalyst **1c–1f** under the same reaction conditions. As summarized in Supplementary Table 7, steric hindrance of substituents on 3,3'-positions can inhibit the silylation of phenolic alcohol.

Supplementary Table 8. The relative catalytic activity of **1a–1f** under the optimized reaction conditions

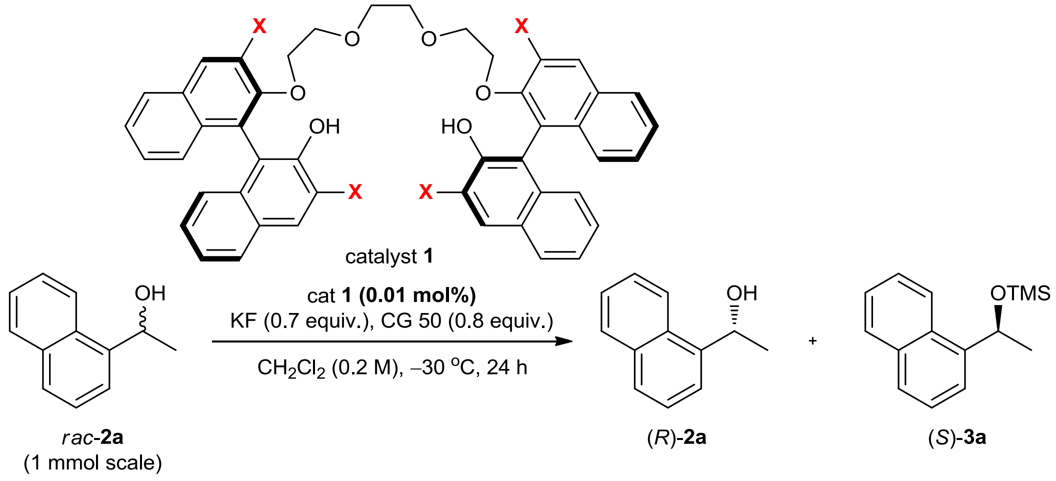

Entry	Catalyst	Conv. (%)[a]	e.r. of (*R*)-**2a**	e.r. of (*S*)-**3a**	*s*[b]
1	**1a** (X=H)	n.r.	-	-	-
2	**1b** (X=Ph)	n.r.	-	-	-
3	**1c** (X=Cl)	16.8	59:41	94.5:5.5	20.8
4	**1d** (X=Br)	36.7	77:23	96.5:3.5	48.4
5	**1e** (X=I)	50.3	98:2	97:3	132
6	**1f** (X=CF_3)	50.8	95.5:4.5	94:6	50.1
7	**1g** (X=C_2F_5)	34.1	73:27	95:5	30.8

[a] Determined by ^{1}H-NMR analysis of the unpurified reaction mixture. [b] s = ln[1−conv.(1+ee$_{(S)\text{-}3a}$] / ln[1−conv.(1−ee$_{(S)\text{-}3a}$]. e.r., enantiomeric ratio. n.r., no reaction.

Comments: As shown from the results in Supplementary Table 8, the relative catalytic activity of **1a–1f** under the optimized reaction conditions is almost similar with that shown in Figure 3.

Supplementary Methods

General information

All starting materials and solvents were obtained from commercial suppliers and used without purification. The organocatalysts **1a–1d, 1i** and **1j** were obtained starting from BINOL according to the literature procedure[3,4]. The catalyst **1e** was purchased from C-Tri Co. Ltd. (www.c-tri.co.kr). The *meso*-diol **4b** and **4c** were prepared from the corresponding aldehyde according to the literature procedure[5]. Thin-layer chromatography (TLC) was performed using silica gel plates (Merck, Kieselgel 60 F254 0.25 mm). Chromatographic purification of the products was performed by using silica gel 60 (230–400 mesh, Merck). ^{1}H NMR (300 and 500 MHz), ^{13}C NMR (75.4 and 125.7 MHz) and ^{29}Si NMR (99.2 MHz) spectra were recorded using a Varian 300 or Bruker 500 spectrometer. Chemical shifts are reported in ppm downfield from tetramethylsilane (TMS) as the *internal standard or* with the solvent reference as the internal standard ($CDCl_3$: δ 7.26 for ^{1}H NMR and δ 77.0 for ^{13}C NMR). ^{19}F NMR (470.4 MHz) spectra were recorded using a Bruker 500 spectrometer with benzotrifluoride ($C_6H_5CF_3$) as the external standard. HPLC analyses for the determination of the enantiomeric excess (ee) of the products were performed using a Varian Pro Star Series instrument equipped with an isostatic pump using a chiral column (CHIRALPACK AD-H, Chiralcel OD-H, OB-H or OJ-H; 250 × 4.6 mm). IR spectra were recorded using a Bruker Vertex 70 spectrometer with the MIRacle Micro ATR accessory. High-resolution mass spectra (HRMS) spectra were recorded using the FAB method using a Jeol JMS-700 MStation. ESI-MS spectra were recorded using a Finnigan Ultra Mass TSQ 7000. Melting points (Mps) were determined using a Buchi B-540 melting point apparatus and are uncorrected. Optical rotations were measured using a PerkinElmer Polarimeter 343 plus. Abbreviations: e.r. (enantiomeric ratio), *s* (selectivity factor), n.d. (not determined), n.r. (no reaction), DMF (*N*,*N*-dimethylformamide), THF (tetrahydrofuran), DCE (1,2-dichloroethane), HMDS (1,1,1,3,3,3-hexamethyldisilazane), TMS (trimethylsilyl), TBAF (tetra-*n*-butylammonium fluoride).

Preparation and characterization of chiral catalysts (1f–1h, 1k–1m)

Chiral catalyst 1f

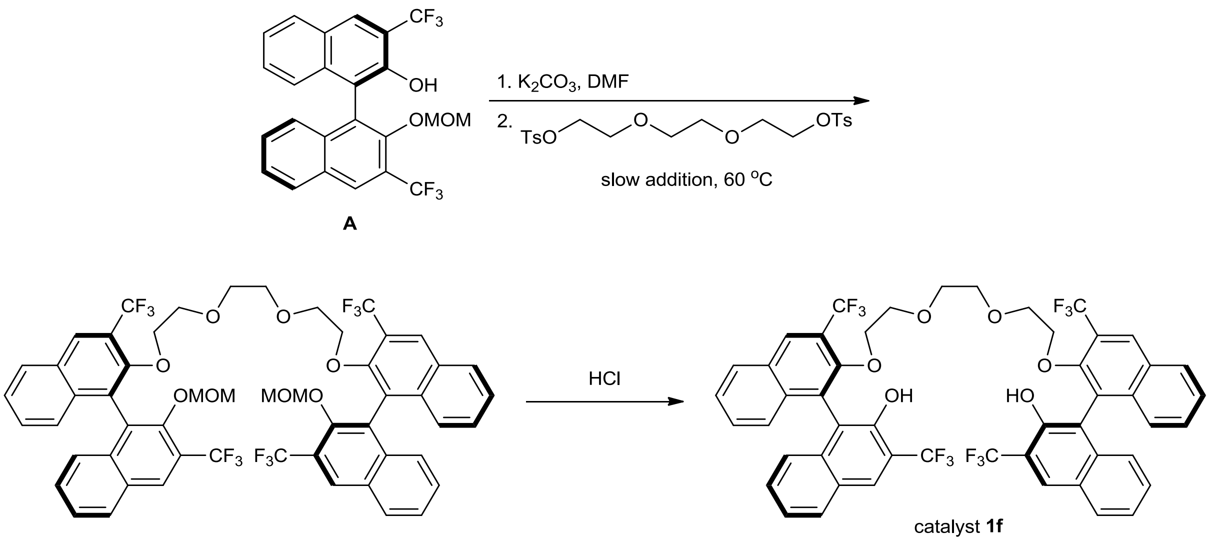

To a solution of the mono-MOM-protected 3,3′-CF_3-(*R*)-BINOL (**A**)[6] (466.4 mg, 1 mmol) in DMF (10 mL), finely powdered K_2CO_3 (344.7 mg, 2.5 mmol) was added, and the reaction mixture stirred for 2 h at room temperature. To this mixture, a solution of bis-tosylated triethylene glycol (252.1 mg, 0.55 mmol) in DMF (5 mL) was then added via a syringe pump for 24 h at 60 °C. The resulting reaction mixture was allowed to cool down to room temperature, followed by the addition of concentrated HCl (35%) (5 mL). The reaction mixture was stirred for an additional 2 h and then diluted with H_2O and EtOAc. The organic layer was separated, washed with brine, dried over anhydrous $NaSO_4$, filtered, and concentrated *in vacuo* to afford a dark brown solid. The crude solid was purified by column chromatography (acetone/hexanes = 1/10) to afford the chiral catalyst **1f** as a white solid.

White solid; mp: 166–168 °C; TLC (acetone : n-hexane, 1:5 v/v): R_f = 0.21; $[\alpha]_D^{20}$ = −16.0 (*c* = 0.1 in $CHCl_3$); ^{1}H NMR (500 MHz, $CDCl_3$): δ 8.33 (s, 2H), 8.28 (s, 2H), 7.97 (d, J = 8.0 Hz, 2H), 7.92 (d, J = 8.0 Hz, 2H), 7.52–7.48 (m, 2H), 7.41–7.33 (m, 6H), 7.13 (d, J = 8.5 Hz, 2H), 7.04 (d, J = 8.5 Hz, 2H), 5.79 (bs, 2H), 3.59–3.51 (m, 4H), 3.11–3.03 (m, 4H), 2.99–2.90 (m, 4H); ^{13}C NMR (125 MHz, $CDCl_3$): δ 153.12, 148.33, 135.02, 134.80, 129.83 (q, $^3J_{C-F}$ = 5.5 Hz)), 129.40, 129.35, 129.34, 129.28, 129.22, 129.12 (q, $^3J_{C-F}$ = 5.5 Hz), 127.39, 126.47, 124.94, 124.89, 124.65, 123.66 (q, $^2J_{C-F}$ = 30.2 Hz)), 123.49 (q, $^1J_{C-F}$ = 271.0 Hz), 123.42 (q, $^1J_{C-F}$ = 271.0 Hz), 122.36, 119.16 (q, $^2J_{C-F}$ = 30.2 Hz), 116.92, 72.99, 69.68, 69.51; ^{19}F NMR

(470 MHz, $CDCl_3$): δ −60.94 (s, 6F), −62.41 (s, 6F); IR: 2960, 2895, 1627, 1603, 1503, 1454, 1325, 1294, 1206, 1130, 1045, 1019, 907, 752, 725 cm^{-1}; HRMS (m/z, FAB): $[M + H]^+$ calcd. for $C_{50}H_{35}F_{12}O_6$, 959.2242; found, 959.2240.

Chiral catalyst 1g

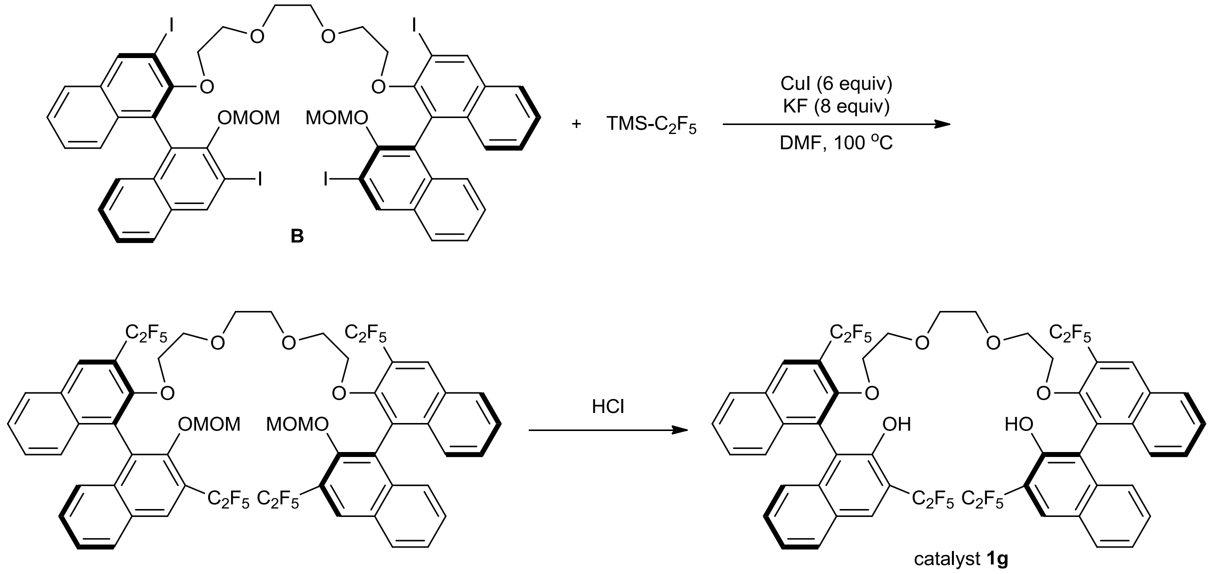

To a solution of the di-MOM-protected compound of **1e** (**B**)[3] (1.2785g, 1 mmol) in DMF (6 mL), KF (464.8 mg, 8 mmol) and CuI (1.1388 g, 6 mmol) were added and the reaction mixture stirred for 10 min at room temperature. To this reaction mixture, $TMSC_2F_5$ (1.5376 g, 8 mmol) was added and stirred for 48 h at 100 °C. The reaction mixture was allowed to cool down to room temperature and concentrated HCl (35%) was added (5 mL). The resulting reaction mixture was stirred for an additional 2 h, and then diluted with H_2O and EtOAc. The organic layer was separated, washed with brine, dried over anhydrous $NaSO_4$, filtered, and concentrated *in vacuo* to afford a dark brown solid. The solid thus obtained was purified by column chromatography (acetone/hexanes = 1/10) to afford the chiral catalyst **1g** as the yellow solid.

Yellow solid; mp: 90–92 °C; TLC (acetone : n-hexane, 1:5 v/v): R_f = 0.26; $[\alpha]_D^{20}$ = −6.4 (*c* = 0.1 in $CHCl_3$); ^1H NMR (500 MHz, $CDCl_3$): δ 8.30 (s, 2H), 8.25 (s, 2H), 7.92 (d, J = 8.0 Hz, 2H), 7.52–7.48 (m, 2H), 7.41–7.33 (m, 6H), 7.25 (d, J = 8.5 Hz, 2H), 7.10 (d, J = 8.0 Hz, 2H), 5.78 (bs, 2H), 3.55–3.50 (m, 4H), 3.02–2.97 (m, 4H), 2.90–2.82 (m, 4H); ^{13}C NMR (125 MHz, $CDCl_3$): δ 153.57, 148.81, 135.10, 134.90, 131.87 (t, $^4J_{C-F}$ = 8.7 Hz), 131.24 (t, $^4J_{C-F}$ =

8.7 Hz), 129.65, 129.51, 129.38, 129.35, 129.26, 127.64, 126.51, 124.94, 124.81, 124.59, 123.02, 121.80 (t, ${}^{3}J_{C\text{-}F}$ = 21.2 Hz), 119.50 (qt, ${}^{1}J_{C\text{-}F}$ = 306.2 Hz and ${}^{1}J_{C\text{-}F}$ = 33.7 Hz), 119.28 (qt, ${}^{1}J_{C\text{-}F}$ = 306.2 Hz and ${}^{2}J_{C\text{-}F}$ = 33.7 Hz), 117.44, 117.11 (t, ${}^{3}J_{C\text{-}F}$ = 21.2 Hz), 113.78 (tq, ${}^{1}J_{C\text{-}F}$ = 254.3 Hz and ${}^{2}J_{C\text{-}F}$ = 40.0 Hz), 113.72 (tq, ${}^{1}J_{C\text{-}F}$ = 254.3 Hz and ${}^{2}J_{C\text{-}F}$ = 40.0 Hz), 73.23, 69.66, 69.26; ${}^{19}F$ NMR (470 MHz, $CDCl_3$): δ −83.02 (s, 6F), −83.43 (s, 6F), −110.52 (s, 2F), −110.593 (s, 2F), −111.375 (s, 2F), −111.43 (s, 2F); IR: 3072, 2889, 1626, 1599, 1501, 1454, 1357, 1278, 1194, 1144, 1128, 1092, 1071, 1029, 969, 905, 829, 751, 718, 640 cm^{-1}; HRMS (m/z, FAB): $[M + Na]^+$ calcd. for $C_{54}H_{34}F_{20}O_6Na_1$, 1181.1934; found, 1181.1934.

Chiral catalyst 1h

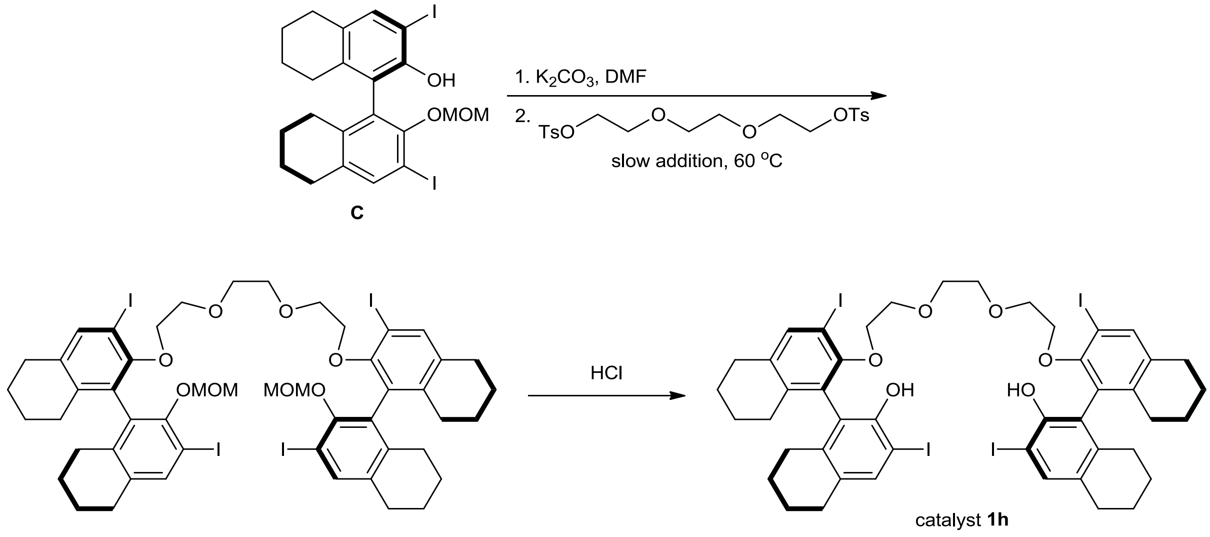

To a solution of the mono-MOM-protected 3,3′-I-(*R*)-H_8-BINOL (**C**) (590.2 mg, 1 mmol) in DMF (10 mL), finely powdered K_2CO_3 (344.1 mg, 2.5 mmol) was added and the reaction mixture stirred for 2 h at room temperature. To this reaction mixture, a solution of bis-tosylated triethylene glycol (251.1 mg, 0.55 mmol) in DMF (5 mL) was then added via a syringe pump for 24 h at 60 °C. The reaction mixture was allowed to cool down to room temperature and concentrated HCl (35%) was added (5 mL). The resulting reaction mixture was stirred for an additional 2 h, and then diluted with H_2O and EtOAc. The organic layer was separated, washed with brine, dried over anhydrous $NaSO_4$, filtered, and concentrated *in vacuo* to afford a dark brown solid. The solid thus obtained was purified by column chromatography (acetone/hexanes = 1/10) to afford the chiral catalyst **1h** as a white solid.

White solid; mp: 220–222 °C; TLC (acetone : n-hexane, 1:5 v/v): R_f = 0.22; $[\alpha]_D^{20}$ = −30.0 (*c*

= 0.1 in $CHCl_3$); ^{1}H NMR (500 MHz, $CDCl_3$): δ 7.57 (s, 2H), 7.45 (s, 2H), 5.16 (s, 2H), 3.88–3.84 (m, 2H), 3.61–3.57 (m, 2H), 3.44 (t, J = 5.0 Hz, 4H), 3.28 (s, 4H), 2.75–2.69 (m, 8H), 2.37–2.28 (m, 4H), 2.06–1.95 (m, 4H), 1.72–1.56 (m, 16H); ^{13}C NMR (125 MHz, $CDCl_3$): δ 154.19, 149.17, 139.69, 138.29, 138.00, 137.59, 136.03, 132.17, 129.62, 123.30, 89.09, 81.15, 72.10, 70.17, 70.08, 29.18, 29.02, 27.18, 26.75, 22.85, 22.78, 22.75, 22.59; IR: 3464, 2934, 2856, 1736, 1654, 1573, 1441, 1354, 1241, 1137, 1070, 1012, 955, 877, 823, 723, 673 cm^{-1}; HRMS (m/z, FAB): $[M + H]^+$ calcd. for $C_{46}H_{51}I_4O_6$, 1206.9865; found, 1206.9866.

Chiral catalyst 1k

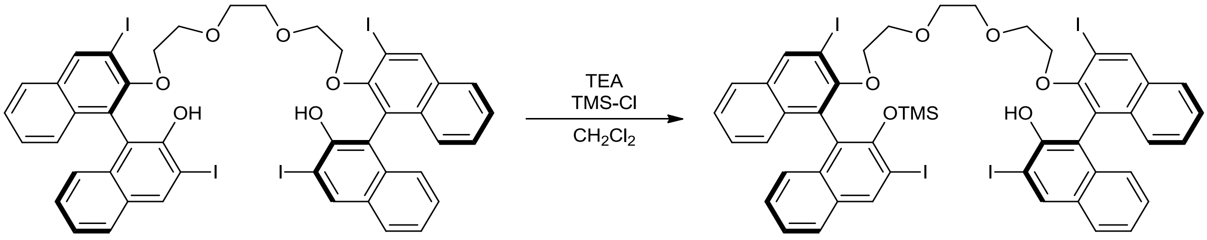

To a solution of catalyst **1e** (119.0 mg, 0.1 mmol) in CH_2Cl_2 (2.5 mL), triethylamine (12.2 mg, 0.12 mmol) and chlorotrimethylsilane (13.0 mg, 0.12 mmol) were added, and the reaction mixture was stirred for 4 h at room temperature for completion, and then the reaction mixture was purified by short column chromatography (acetone/hexane = 1/5) without work-up, affording chiral catalyst **1k** as a white solid.

White solid; mp: 140–142 °C; TLC (acetone : n-hexane, 1:5 v/v): R_f = 0.35; $[\alpha]_D^{20}$ = −44.0 (*c* = 0.1 in $CHCl_3$); ^{1}H NMR (500 MHz, $CDCl_3$): δ 8.53 (s, 1H), 8.49 (s, 2H), 8.43 (s, 1H), 7.78 (d, J = 8.5 Hz, 2H), 7.72–7.70 (m, 2H), 7.41–7.39 (m, 2H), 7.31–7.17 (m, 6H), 7.11–7.08 (m, 2H), 7.01–6.96 (m, 2H), 5.76 (s, 1H), 3.83–3.55 (m, 3H), 3.41–3.23 (m, 3H), 3.17–2.93 (m, 6H), −0.27 (s, 9H); ^{13}C NMR (125 MHz, $CDCl_3$): δ 154.21, 154.11, 151.35, 149.47, 140.37, 139.71, 139.59, 139.53, 133.95, 133.90, 133.52, 133.40, 132.48, 132.31, 130.63, 130.32, 127.48, 127.43, 127.05, 127.03, 126.92, 126.85, 126.84, 126.10, 126.05, 125.97, 125.62, 125.60, 125.37, 124.97, 124.63, 124.34, 123.14, 121.80, 115.44, 93.74, 93.06, 92.81, 87.20, 87.13, 72.59, 72.34, 69.97, 69.93, 69.86, 69.47, 0.91; ^{29}Si NMR (100 MHz, $CDCl_3$): δ 20.69; IR: 3059, 2951, 2892, 2358, 1615, 1571, 1558, 1491, 1446, 1412, 1391, 1348, 1249, 1224, 1136, 1046, 1008, 946, 882, 848, 775, 747, 696 cm^{-1}; HRMS (m/z, FAB): $[M]^+$ calcd. for

$C_{49}H_{41}O_6I_4Si_1$, 1260.8845; found, 1260.8846.

Chiral catalyst 1l

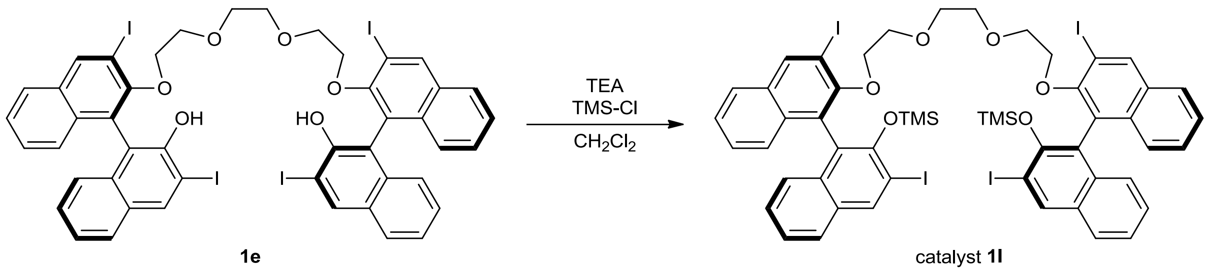

To a solution of catalyst **1e** (119.0 mg, 0.1 mmol) in CH_2Cl_2 (2.5 mL), triethylamine (24.4 mg, 0.24 mmol) and chlorotrimethylsilane (26.0 mg, 0.24 mmol) were added, and the reaction mixture stirred for 4 h at room temperature for completion. The reaction mixture then was purified by short column chromatography (acetone/hexane = 1/5) without work-up, affording chiral catalyst **1l** as a white solid.

White solid; mp: 128–130 ^{o}C; TLC (acetone : n-hexane, 1:5 v/v): R_f = 0.54; $[\alpha]_D^{20}$ = −128 (*c* = 0.1 in $CHCl_3$); ^{1}H NMR (500 MHz, $CDCl_3$): δ 8.49 (s, 2H), 8.48 (s, 2H), 7.78 (d, J = 8.0 Hz, 2H), 7.70 (d, J = 8.0 Hz, 2H), 7.43–7.38 (m, 2H), 7.31–7.24 (m, 4H), 7.21–7.16 (m, 6H), 7.10 (d, J = 8.5 Hz, 2H), 6.97 (d, J = 8.5 Hz, 2H), 3.85–3.81 (m, 2H), 3.41–3.37 (m, 2H), 3.24–3.15 (m, 4H), 3.07–3.00 (m, 4H), −0.27 (s, 18H); ^{13}C NMR (125 MHz, $CDCl_3$): δ 154.16, 151.38, 139.72, 139.57, 133.96, 133.90, 132.30, 130.64, 127.03, 126.92, 126.85, 126.84, 126.12, 126.05, 125.62, 125.58, 124.63, 121.78, 93.74, 93.08, 72.31, 69.92, 69.54, 0.91; ^{29}Si NMR (100 MHz, $CDCl_3$): δ 20.70; IR: 3054, 2951, 2898, 1559, 1489, 1445, 1411, 1394, 1346, 1249, 1150, 1135, 1046, 1008, 946, 846, 800, 747, 693, 656 cm^{-1}; HRMS (m/z, FAB): $[M + H]^+$ calcd. for $C_{52}H_{51}O_6I_4Si_2$, 1334.9398; found, 1334.9398.

Chiral catalyst 1m

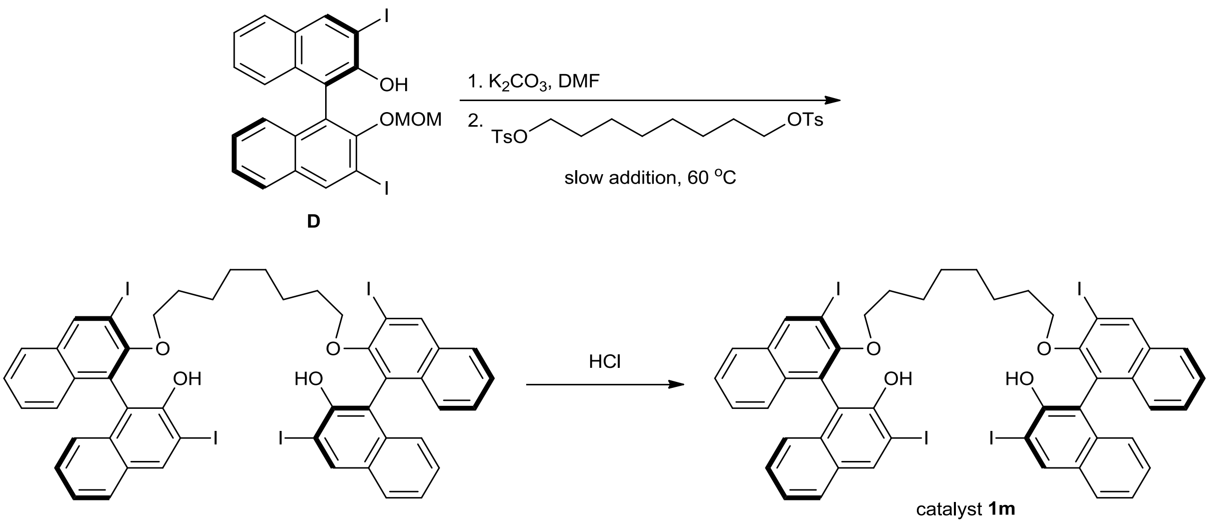

To a solution of the mono-MOM-protected 3,3′-I-(*R*)-BINOL (**D**) (582.1 mg, 1 mmol) in DMF (10 mL), finely powdered K_2CO_3 (344.7 mg, 2.5 mmol) was added, and the reaction mixture stirred for 2 h at room temperature. To this mixture, a solution of bis-tosylated octanediol (250.0 mg, 0.55 mmol) in DMF (5 mL) was then added via a syringe pump for 24 h at 60 °C. The reaction mixture was allowed to cool down to room temperature, followed by the addition of concentrated HCl (35%, 5 mL). The reaction mixture was stirred for an additional 2 h and then diluted with H_2O and EtOAc. The organic layer was separated, washed with brine, dried over anhydrous $NaSO_4$ filtered, and concentrated in vacuo afforded a dark brown solid. The solid thus obtained was purified by column chromatography (acetone/hexane = 1/10) to afford chiral catalyst **1m** as a white solid.

White solid; mp: 204–206 °C; TLC (acetone : n-hexane, 1:5 v/v): R_f = 0.26; $[\alpha]_D^{20}$ = −36.0 (*c* = 0.1 in $CHCl_3$); ^{1}H NMR (500 MHz, $CDCl_3$): δ 8.55 (s, 2H), 8.42 (s, 2H), 7.81 (d, J = 8.0 Hz, 2H), 7.70 (d, J = 8.0 Hz, 2H), 7.44–7.41 (m, 2H), 7.31–7.27 (m, 4H), 7.25–7.21 (m, 2H), 7.14 (d, J = 8.5 Hz, 2H), 7.02 (d, J = 8.5 Hz, 2H), 5.50 (s, 2H), 3.68–3.64 (m, 2H), 3.43–3.38 (m, 2H), 1.31–1.18 (m, 4H), 0.73–0.60 (m, 6H), 0.57–0.51 (m, 2H); ^{13}C NMR (125 MHz, $CDCl_3$): δ 154.38, 149.39, 140.45, 139.41, 133.76, 133.50, 132.57, 130.32, 127.45, 127.42, 127.13, 127.06, 126.02, 125.51, 125.09, 124.31, 123.61, 115.57, 92.98, 87.01, 74.09, 29.66, 28.80, 25.23; IR: 3507, 3056, 2929, 2853, 1614, 1572, 1494, 1446, 1352, 1226, 1197, 1144, 1080, 1009, 952, 886, 796, 747, 679, 636 cm^{-1}; HRMS (m/z, FAB): $[M + Na]^+$ calcd. for $C_{48}H_{38}I_4O_4Na_1$, 1208.8847; found, 1208.8846.

Analytical data of the remaining alcohols ((*R*)-2a–2r)

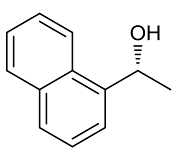

(*R*)-**2a**

White solid[3]; TLC (acetone : n-hexane, 1:5 v/v): R_f = 0.28; ^{1}H NMR (300 MHz, $CDCl_3$): δ 8.15–8.10 (m, 1H), 7.90–7.85 (m, 1H), 7.80–7.75 (m, 1H), 7.70–7.65 (m, 1H) 7.56–7.44 (m, 3H), 5.67 (qd, J = 6.3 Hz and J = 3.6 Hz, 1H), 1.94 (d, J = 3.6 Hz, 1H), 1.67 (d, J = 6.3 Hz, 3H); ^{13}C NMR (75 MHz, $CDCl_3$): δ 141.34, 133.82, 130.29, 128.88, 127.93, 126.02, 125.53, 125.51, 123.16, 121.98, 67.143, 24.34.

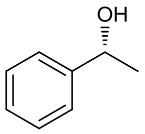

(*R*)-**2b**

Colorless liquid[3]; TLC (acetone : n-hexane, 1:5 v/v): R_f = 0.39; ^{1}H NMR (300 MHz, $CDCl_3$): δ 7.38–7.22 (m, 5H), 4.86 (qd, J = 6.3 Hz and J = 3.3 Hz, 1H), 2.07 (d, J = 3.3 Hz, 1H), 7.71 (d, J = 6.3 Hz, 3H); ^{13}C NMR (75 MHz, $CDCl_3$): δ 145.78, 128.43, 127.39, 125.33, 70.32, 25.09.

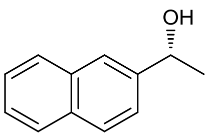

(*R*)-**2c**

White solid[3]; TLC (acetone : n-hexane, 1:5 v/v): R_f = 0.25; ^{1}H NMR (500 MHz, $CDCl_3$): δ 7.85–7.79 (m, 4H), 7.51–7.44 (m, 3H), 5.06 (qd, J = 6.5 Hz and J = 4.0 Hz, 1H), 1.94 (d, J = 3.5 Hz, 1H), 1.58 (d, J = 6.5 Hz, 3H); ^{13}C NMR (125 MHz, $CDCl_3$): δ 143.19, 133.34, 132.94, 128.35, 127.95, 127.70, 126.18, 125.83, 123.82, 70.57, 25.17.

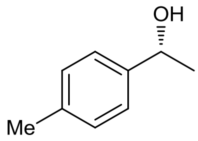

(*R*)-**2d**

Colorless liquid[3]; TLC (acetone : n-hexane, 1:5 v/v): R_f = 0.33; ^{1}H NMR (300 MHz, $CDCl_3$): δ 7.26–7.22 (m, 2H), 7.16–7.11 (m, 2H), 4.82 (qd, J = 6.3 Hz and J = 3.3 Hz, 1H), 2.33 (s, 3H), 2.05 (d, J = 3.3 Hz, 1H), 1.45 (d, J = 6.6 Hz, 3H); ^{13}C NMR (75 MHz, $CDCl_3$): δ 142.86, 137.01, 129.07, 125.30, 70.13, 25.01, 21.02.

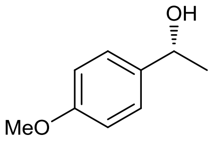

(*R*)-**2e**

Colorless liquid[3]; TLC (acetone : n-hexane, 1:5 v/v): R_f = 0.22; ^{1}H NMR (500 MHz, $CDCl_3$): δ 7.32–7.28 (m, 2H), 6.90–6.85 (m, 2H), 4.85 (q, J = 6.0 Hz, 1H), 3.80 (s, 3H), 1.82 (s, 1H), 1.47 (d, J = 6.5 Hz, 3H); ^{13}C NMR (125 MHz, $CDCl_3$): δ 158.94, 137.96, 126.63, 113.80, 69.95, 55.26, 24.99.

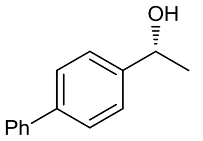

(*R*)-**2f**

White solid[7]; TLC (acetone : n-hexane, 1:5 v/v): R_f = 0.13; ^{1}H NMR (500 MHz, $CDCl_3$): δ 7.61–7.55 (m, 4H), 7.47–7.41 (m, 4H), 7.37–7.32 (m, 1H), 4.95 (qd, J = 6.5 Hz and J = 3.5 Hz, 1H), 1.85 (d, J = 4.0 Hz, 1H), 1.54 (d, J = 6.5 Hz, 3H); ^{13}C NMR (125 MHz, $CDCl_3$): δ 144.81, 140.85, 140.47, 128.77, 127.27, 127.09, 125.85, 70.19, 25.16.

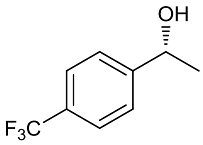

(*R*)-**2g**

Colorless liquid[8]; TLC (acetone : n-hexane, 1:5 v/v): R_f = 0.23; ^{1}H NMR (500 MHz, $CDCl_3$): δ 7.63–7.58 (m, 2H), 7.52–7.46 (m, 2H), 4.96 (qd, J = 6.5 Hz and J = 4.0 Hz, 1H), 1.98 (d, J = 3.5 Hz, 1H), 1.50 (d, J = 6.5 Hz, 3H); ^{13}C NMR (125 MHz, $CDCl_3$): δ 149.68, 129.63 (q, ^{2}J(C-C-F) = 32.3 Hz)), 125.65, 125.46 (q, ^{3}J(C-C-C-F) = 3.7 Hz)), 124.16 (q, ^{1}J(C-F) = 271.2 Hz)), 69.84, 25.40. ^{19}F NMR (470 MHz, $CDCl_3$): δ −62.45 (s, 3F).

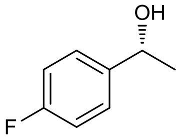

(*R*)-**2h**

Colorless liquid[3]; TLC (acetone : n-hexane, 1:5 v/v): R_f = 0.31; ^{1}H NMR (300 MHz, $CDCl_3$): δ 7.37–7.30 (m, 2H), 7.06–6.99 (m, 2H), 4.87 (qd, J = 6.6 Hz and J = 3.6 Hz, 1H), 1.87 (d, J = 3.3 Hz, 1H), 1.47 (d, J = 6.6 Hz, 3H); ^{13}C NMR (75 MHz, $CDCl_3$): 162.11 (d, ^{1}J(C-F) = 243.8 Hz), 141.51 (d, ^{4}J(C-C-C-C-F) = 3.2 Hz), 127.01 (d, ^{3}J(C-C-C-F) = 7.9 Hz), 115.23 (d, ^{2}J(C-C-F) = 21.2 Hz), 69.77, 25.28.

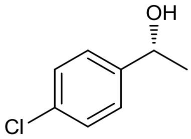

(*R*)-**2i**

Colorless liquid[3]; TLC (acetone : n-hexane, 1:5 v/v): R_f = 0.31; ^{1}H NMR (300 MHz, $CDCl_3$): δ 7.33–7.26 (m, 4H), 4.86 (q, J = 6.6 Hz, 1H), 1.99 (bs, 1H), 1.46 (d, J = 6.3 Hz, 3H); ^{13}C NMR (75 MHz, $CDCl_3$): δ 144.22, 133.03, 128.56, 126.75, 69.70, 25.22.

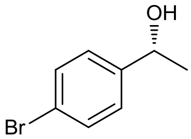

(*R*)-**2j**

White solid[3]; TLC (acetone : n-hexane, 1:5 v/v): R_f = 0.31; ^{1}H NMR (300 MHz, $CDCl_3$): δ 7.49–7.43 (m, 2H), 7.26–7.21 (m, 2H), 4.84 (qd, J = 6.3 Hz and J = 3.6 Hz, 1H), 1.96 (d, J = 3.6 Hz, 1H), 1.46 (d, J = 6.6 Hz, 3H); ^{13}C NMR (75 MHz, $CDCl_3$): δ 144.74, 131.51, 127.11, 121.12, 69.74, 25.21.

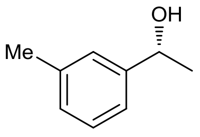

(*R*)-**2k**

Colorless liquid[9]; TLC (acetone : n-hexane, 1:5 v/v): R_f = 0.20; ^{1}H NMR (500 MHz, $CDCl_3$): δ 7.26–7.21 (m, 1H), 7.20–7.13 (m, 2H), 7.10–7.06 (m, 1H), 4.85 (qd, J = 6.5 Hz and J = 3.0 Hz, 1H), 2.36 (s, 3H), 1.90 (d, J = 3.0 Hz, 1H), 1.48 (d, J = 6.5 Hz, 3H); ^{13}C NMR (125 MHz, $CDCl_3$): δ 145.81, 138.18, 128.44, 128.23, 126.12, 122.45, 70.45, 25.14, 21.48.

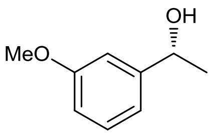

(*R*)-**2l**

Colorless liquid[10]; TLC (acetone : n-hexane, 1:5 v/v): R_f = 0.20; ^{1}H NMR (500 MHz, $CDCl_3$): δ 7.29–7.24 (m, 1H), 6.97–6.92 (m, 2H), 6.84–6.79 (m, 1H), 4.87 (qd, J = 6.5 Hz and J = 3.5 Hz, 1H), 3.82 (s, 3H), 1.87 (d, J = 3.5 Hz, 1H), 1.49 (d, J = 6.5 Hz, 3H); ^{13}C NMR (125 MHz, $CDCl_3$): δ 159.80, 147.60, 129.56, 177.69, 112.91, 110.90, 70.37, 55.24, 25.16.

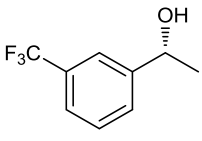

(*R*)-**2m**

Colorless liquid[10]; TLC (acetone : n-hexane, 1:5 v/v): R_f = 0.24; ^{1}H NMR (500 MHz, $CDCl_3$): δ 7.66–7.63 (m, 1H), 7.57–7.51 (m, 2H), 7.49–7.44 (m, 1H), 4.97 (q, J = 6.5 Hz, 1H), 1.93 (s, 1H), 1.52 (d, J = 6.5 Hz, 3H); ^{13}C NMR (125 MHz, $CDCl_3$): δ 146.71, 130.82 (q, ^{2}J(C-C-F) = 31.8 Hz)), 128.94, 128.78, 124.24 (q, ^{4}J(C-C-C-C-F) = 3.8 Hz)), 124.15 (q, ^{1}J(C-F) = 270.6 Hz)), 122.21 (q, ^{3}J(C-C-C-F) = 3.9 Hz)), 69.85, 25.39. ^{19}F NMR (470 MHz, $CDCl_3$): δ −62.59 (s).

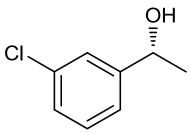

(*R*)-**2n**

Colorless liquid[9]; TLC (acetone : n-hexane, 1:5 v/v): R_f = 0.25; ^{1}H NMR (500 MHz, $CDCl_3$): δ 7.38–7.36 (m, 1H), 7.29–7.22 (m, 3H), 4.87 (q, J = 6.5 Hz, 1H), 1.98 (bs, 1H), 1.48 (d, J = 6.5 Hz, 3H); ^{13}C NMR (125 MHz, $CDCl_3$): δ 147.86, 134.38, 129.81, 127.55, 125.64, 123.55, 69.82, 25.26.

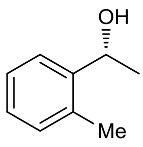

(*R*)-**2o**

Colorless liquid[8]; TLC (acetone : n-hexane, 1:5 v/v): R_f = 0.36; ^{1}H NMR (500 MHz, $CDCl_3$): δ 7.52–7.48 (m, 1H), 7.25–7.21 (m, 1H), 7.19–7.10 (m, 2H), 5.12 (qd, J = 6.0 Hz and J = 3.0 Hz, 1H), 2.34 (s, 3H), 1.80 (d, J = 3.5 Hz, 1H), 1.46 (d, J = 6.5 Hz, 3H); ^{13}C NMR (125 MHz, $CDCl_3$): δ 143.85, 134.25, 130.39, 127.20, 126.40, 124.47, 66.84, 23.95, 18.93.

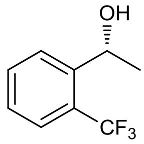

(*R*)-**2p**

Colorless liquid[11]; TLC (acetone : n-hexane, 1:5 v/v): R_f = 0.32; ^{1}H NMR (500 MHz, $CDCl_3$): δ 7.84–7.81 (m, 1H), 7.62–7.56 (m, 2H), 7.39–7.34 (m, 1H), 5.36–5.30 (m, 1H), 2.01–1.97 (m, 1H), 1.49 (d, J = 6.0 Hz, 3H); ^{13}C NMR (125 MHz, $CDCl_3$): δ 145.02, 132.41, 127.37, 127.09, 126.48 (q, ^{2}J(C-C-F) = 29.9 Hz)), 125.31 (q, ^{3}J(C-C-C-F) = 6.2 Hz)), 124.38 (q, ^{1}J(C-F) = 272.5 Hz)), 65.70 (q, ^{4}J(C-C-C-C-F) = 2.5 Hz)), 25.44. ^{19}F NMR (470 MHz, $CDCl_3$): δ −58.32 (s, 3F).

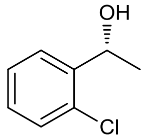

(*R*)-**2q**

Colorless liquid[8]; TLC (acetone : n-hexane, 1:5 v/v): R_f = 0.30; ^{1}H NMR (500 MHz, $CDCl_3$): δ 7.60–7.57 (m, 1H), 7.33–7.27 (m, 2H), 7.22–7.17 (m, 1H), 5.27 (qd, J = 6.5 Hz and J = 3.5 Hz, 1H), 2.16 (d, J = 3.5 Hz, 1H), 1.48 (d, J = 6.0 Hz, 3H); ^{13}C NMR (125 MHz, $CDCl_3$): δ 143.06, 131.64, 129.41, 128.41, 127.23, 126.42, 66.97, 23.52.

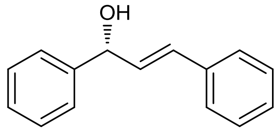

(*R*)-**2r**

White solid[3]; TLC (acetone : n-hexane, 1:5 v/v): R_f = 0.28; ^{1}H NMR (500 MHz, $CDCl_3$): δ 7.45–7.42 (m, 2H), 7.40–7.35 (m, 4H), 7.32–7.28 (m, 3H), 7.25–7.21 (m, 1H), 6.72–6.66 (m, 1H), 6.41–6.36 (m, 1H), 5.39 (q, J = 3.0 Hz, 1H), 2.04 (d, J = 3.5 Hz, 1H); ^{13}C NMR (125 MHz, $CDCl_3$): δ 142.74, 136.49, 131.48, 130.55, 128.62, 128.56, 127.81, 127.78, 126.60, 126.33, 75.14.

Analytical data of the TMS-ether products ((*S*)-3a–3r)

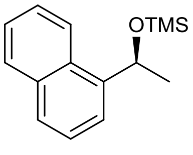

(*S*)-**3a**

Colorless liquid[3]; TLC (acetone : n-hexane, 1:20 v/v): R_f = 0.76; ^{1}H NMR (500 MHz, $CDCl_3$): δ 8.11–8.07 (m, 1H), 7.88–7.85 (m, 1H), 7.76–7.72 (m, 1H), 7.69–7.66 (m, 1H), 7.52–7.44 (m, 2H), 5.59 (q, J = 6.5 Hz, 1H), 1.59 (d, J = 6.5 Hz, 3H), 0.08 (s, 9H); ^{13}C NMR (125 MHz, $CDCl_3$): δ 142.20, 133.71, 129.86, 128.85, 127.29, 125.60, 125.56, 125.19, 123.25, 122.72, 68.06, 26.47, 0.08.

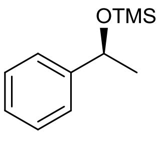

(*S*)-**3b**

Colorless liquid[3]; TLC (acetone : n-hexane, 1:20 v/v): R_f = 0.78; ^{1}H NMR (500 MHz, $CDCl_3$): δ 7.35–7.29 (m, 4H), 7.25–7.20 (m, 1H), 4.85 (q, J = 6.5 Hz, 1H), 1.43 (d, J = 6.5 Hz, 3H), 0.08 (s, 9H); ^{13}C NMR (125 MHz, $CDCl_3$): δ 128.13, 126.82, 125.35, 70.57, 26.86, 0.09.

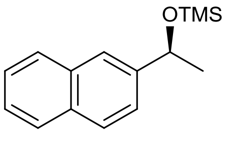

(*S*)-**3c**

Colorless liquid[3]; TLC (acetone : n-hexane, 1:20 v/v): R_f = 0.77; ^{1}H NMR (500 MHz, $CDCl_3$): δ 7.84–7.78 (m, 3H), 7.77–7.74 (m, 1H), 7.50–7.41 (m, 3H), 5.02 (q, J = 6.0 Hz, 1H), 1.51 (d, J = 6.5 Hz, 3H), 0.10 (s, 9H); ^{13}C NMR (125 MHz, $CDCl_3$): δ 143.94, 133.29, 132.70, 127.89, 127.63, 125.89, 125.45, 124.08, 123.60, 70.75, 26.88, 0.13.

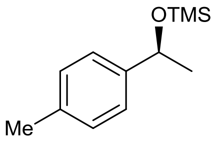

(*S*)-**3d**

Colorless liquid[3]; TLC (acetone : n-hexane, 1:5 v/v): R_f = 0.75; ^{1}H NMR (500 MHz, $CDCl_3$): δ 7.23–7.20 (m, 2H), 7.13–7.10 (m, 2H), 4.83 (q, J = 6.0 Hz, 1H), 2.33 (s, 3H), 1.42 (d, J = 6.5 Hz, 3H), 0.07 (s, 9H); ^{13}C NMR (125 MHz, $CDCl_3$): δ 143.50, 136.35, 128.80, 125.29, 70.43, 26.88, 21.07, 0.10.

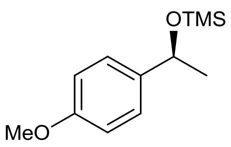

(*S*)-**3e**

Colorless liquid[3]; TLC (acetone : n-hexane, 1:20 v/v): R_f = 0.71; ^{1}H NMR (500 MHz, $CDCl_3$): δ 7.27–7.23 (m, 2H), 6.87–6.83 (m, 2H), 4.81 (q, J = 6.5 Hz, 1H), 3.79 (s, 3H), 1.41 (d, J = 6.5 Hz, 3H), 0.06 (s, 9H); ^{13}C NMR (125 MHz, $CDCl_3$): δ 158.49, 138.66, 126.52, 113.47, 70.19, 55.21, 26.82, 0.11.

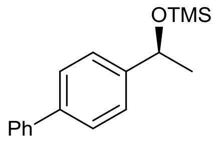

(*S*)-**3f**

Colorless liquid[12]; TLC (acetone : n-hexane, 1:20 v/v): R_f = 0.75; $[\alpha]_D^{20}$ = −40.0 (c = 0.1 in $CHCl_3$); ^{1}H NMR (500 MHz, $CDCl_3$): δ 7.60–7.54 (m, 4H), 7.44–7.39 (m, 4H), 7.35–7.31 (m, 1H), 4.91 (q, J = 6.5 Hz, 1H), 1.47 (d, J = 6.5 Hz, 3H), 0.11 (s, 9H); ^{13}C NMR (125 MHz, $CDCl_3$): δ 145.54, 141.03, 139.75, 128.69, 127.08, 127.05, 126.91, 125.78, 70.32, 26.83, 0.13.

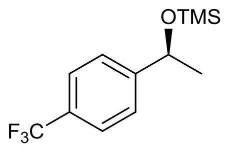

(*S*)-**3g**

New compound: colorless liquid; TLC (acetone : n-hexane, 1:20 v/v): R_f = 0.72; $[\alpha]_D^{20}$ = −46.0 (c = 0.1 in $CHCl_3$); ^{1}H NMR (500 MHz, $CDCl_3$): δ 7.59–7.55 (m, 2H), 7.46–7.42 (m, 2H), 4.90 (q, J = 6.5 Hz, 1H), 1.42 (d, J = 6.5 Hz, 3H), 0.09 (s, 9H); ^{13}C NMR (125 MHz, $CDCl_3$): δ 150.55, 129.07 (q, ^{2}J(C-C-F) = 32.0 Hz)), 125.55, 125.15 (q, ^{3}J(C-C-C-F) = 3.8 Hz)), 124.27 (q, ^{1}J(C-F) = 270.2 Hz)), 70.02, 26.85, 0.02. ^{19}F NMR (470 MHz, $CDCl_3$): δ −62.33 (s, 3F); IR: 2961, 2868, 1620, 1416, 1325, 1253, 1165, 1125, 1095, 1032, 958, 840, 750 cm^{-1}; HRMS (m/z, EI): $[M]^+$ calcd. for $C_{12}H_{17}O_1F_3Si_1$, 262.0995; found, 262.0996.

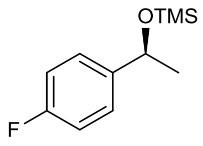

(*S*)-**3h**

Colorless liquid[3]; TLC (acetone : n-hexane, 1:20 v/v): R_f = 0.73; ^{1}H NMR (300 MHz, $CDCl_3$): δ 7.31–7.27 (m, 2H), 7.01–6.97 (m, 2H), 4.83 (q, J = 6.5 Hz, 1H), 1.41 (d, J = 6.5 Hz, 3H), 0.07 (s, 9H); ^{13}C NMR (125 MHz, $CDCl_3$): δ 161.77 (d, ^{1}J(C-F) = 242.6 Hz)), 142.24 (d, ^{4}J(C-C-C-C-F) = 3.0 Hz)), 126.86 (d, ^{3}J(C-C-C-F) = 7.7 Hz)), 114.88 (d, ^{2}J(C-C-F) = 21.1 Hz)), 69.98, 26.93, 0.05. ^{19}F NMR (470 MHz, $CDCl_3$): δ −116.39 (s, 1F).

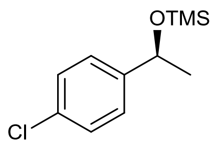

(*S*)-**3i**

Colorless liquid[3]; TLC (acetone : n-hexane, 1:20 v/v): R_f = 0.74; ^{1}H NMR (500 MHz, $CDCl_3$): δ 7.29–7.25 (m, 4H), 4.82 (q, J = 6.5 Hz, 1H), 1.40 (d, J = 6.0 Hz, 3H), 0.08 (s, 9H); ^{13}C NMR (125 MHz, $CDCl_3$): δ 145.05, 132.39, 128.27, 126.71, 69.93, 26.86, 0.04.

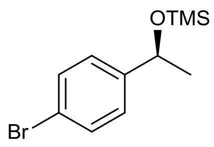

(*S*)-**3j**

Colorless liquid[3]; TLC (acetone : n-hexane, 1:20 v/v): R_f = 0.75; ^{1}H NMR (500 MHz, $CDCl_3$): δ 7.44–7.42 (m, 2H), 7.22–7.19 (m, 2H), 4.80 (q, J = 6.0 Hz, 1H), 1.40 (d, J = 6.0 Hz, 3H), 0.08 (s, 9H); ^{13}C NMR (125 MHz, $CDCl_3$): δ 145.58, 131.21, 127.09, 120.49, 69.96, 26.83, 0.04.

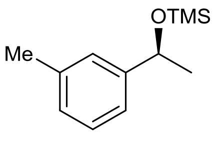

(*S*)-**3k**

New compound: colorless liquid; TLC (acetone : n-hexane, 1:20 v/v): R_f = 0.77; mp: 148-150 °C; $[\alpha]_D^{20}$ = −45.0 (c = 0.1 in $CHCl_3$); ^{1}H NMR (500 MHz, $CDCl_3$): δ 7.21–7.18 (m, 1H), 7.14–7.11 (m, 2H), 7.05–7.03 (m, 1H), 4.82 (q, J = 6.5 Hz, 1H), 2.35 (s, 3H), 1.42 (d, J = 6.5 Hz, 3H), 0.08 (s, 9H); ^{13}C NMR (125 MHz, $CDCl_3$): δ 146.38, 137.66, 128.02, 127.56, 126.04, 122.43, 70.58, 26.86, 21.48, 0.11; IR: 2973, 2926, 2867, 1609, 1488, 1369, 1251, 1163, 1095, 1036, 964, 840, 750, 702 cm^{-1}; HRMS (m/z, EI): $[M]^+$ calcd. for $C_{12}H_{20}O_1Si_1$, 208.1278; found, 208.1280.

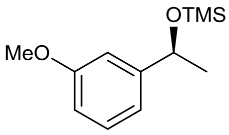

(*S*)-**3l**

New compound: colorless liquid; TLC (acetone : n-hexane, 1:20 v/v): R_f = 0.73; $[\alpha]_D^{20}$ = −45.0 (c = 0.1 in $CHCl_3$); ^{1}H NMR (500 MHz, $CDCl_3$): δ 7.24–7.20 (m, 1H), 6.92–6.89 (m, 2H), 6.78–6.76 (m, 1H), 4.83 (q, J = 6.5 Hz, 1H), 3.81 (s, 3H), 1.42 (d, J = 6.5 Hz, 3H), 0.08 (s, 9H); ^{13}C NMR (125 MHz, $CDCl_3$): δ 159.53, 148.26, 129.11, 117.75, 112.15, 110.93, 70.466, 55.15, 26.85, 0.08; IR: 2957, 2868, 1602, 1486, 1456, 1252, 1159, 1095, 1035, 969, 840, 750, 698 cm^{-1}; HRMS (m/z, EI): $[M]^+$ calcd. for $C_{12}H_{20}O_2Si_1$, 224.1227; found, 224.1225.

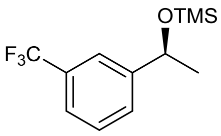

(*S*)-**3m**

Colorless liquid[12]; TLC (acetone : n-hexane, 1:20 v/v): R_f = 0.71; $[\alpha]_D^{20}$ = −36.0 (c = 0.1 in $CHCl_3$); ^{1}H NMR (500 MHz, $CDCl_3$): δ 7.62–7.59 (m, 1H), 7.52–7.48 (m, 2H), 7.44–7.41 (m, 1H), 4.91 (q, J = 6.5 Hz, 1H), 1.44 (d, J = 6.0 Hz, 3H), 0.09 (s, 9H); ^{13}C NMR (125 MHz, $CDCl_3$): δ 147.55, 130.50 (q, ^{2}J(C-C-F) = 31.8 Hz)), 128.67 (d, ^{4}J(C-C-C-C-F) = 1.0 Hz)), 128.60, 124.27 (q, ^{1}J(C-F) = 270.6 Hz)), 123.67 (q, ^{3}J(C-C-C-F) = 3.8 Hz)), 122.13 (q, ^{3}J(C-C-C-F) = 3.8 Hz)), 70.02, 26.86, 0.01. ^{19}F NMR (470 MHz, $CDCl_3$): δ −62.57 (s, 3F).

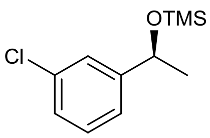

(*S*)-**3n**

Colorless liquid[13]; TLC (acetone : n-hexane, 1:20 v/v): R_f = 0.72; $[\alpha]_D^{20}$ = −47.0 (c = 0.1 in $CHCl_3$); ^{1}H NMR (500 MHz, $CDCl_3$): δ 7.33–7.32 (m, 1H), 7.25–7.18 (m, 3H), 4.81 (q, J = 6.5 Hz, 1H), 1.40 (d, J = 6.5 Hz, 3H), 0.09 (s, 9H); ^{13}C NMR (125 MHz, $CDCl_3$): δ 148.67, 134.03, 129.44, 126.93, 125.53, 123.45, 69.95, 26.83, 0.04.

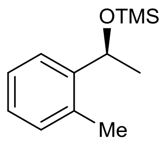

(*S*)-**3o**

Colorless liquid[14]; TLC (acetone : n-hexane, 1:20 v/v): R_f = 0.71; ^{1}H NMR (500 MHz, $CDCl_3$): δ 7.52–7.48 (m, 1H), 7.21–7.18 (m, 1H), 7.14–7.07 (m, 2H), 5.04 (q, J = 6.0 Hz, 1H), 2.31 (s, 3H), 1.38 (d, J = 6.5 Hz, 3H), 0.06 (s, 9H); ^{13}C NMR (125 MHz, $CDCl_3$): δ 144.58, 133.00, 129.91, 126.48, 126.00, 125.30, 67.38, 25.58, 18.87, −0.01.

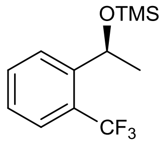

(*S*)-**3p**

New compound: colorless liquid; TLC (acetone : n-hexane, 1:20 v/v): R_f = 0.70; $[\alpha]_D^{20}$ = −16.0 (c = 0.05 in $CHCl_3$); ^{1}H NMR (500 MHz, $CDCl_3$): δ 7.83–7.81 (m, 1H), 7.57–7.53 (m, 2H), 7.33–7.30 (m, 1H), 5.26–5.22 (m, 1H), 1.40 (d, J = 6.0 Hz, 3H), 0.03 (s, 9H); ^{13}C NMR (125 MHz, $CDCl_3$): δ 146.23, 132.08, 127.71, 126.76, 125.57 (q, ^{2}J(C-C-F) = 29.9 Hz)), 124.93 (q, ^{3}J(C-C-C-F) = 5.9 Hz)), 124.47 (q, ^{1}J(C-F) = 272.1 Hz)), 66.15 (q, ^{4}J(C-C-C-C-F) = 2.0 Hz)), 27.62, −0.16. ^{19}F NMR (470 MHz, $CDCl_3$): δ −58.39 (s, 3F); IR: 2979, 2960, 2902, 1609, 1454, 1373, 1313, 1253, 1164, 1121, 1092, 1060, 1027, 957, 841, 767 cm^{-1}; HRMS (m/z, EI): $[M]^+$ calcd. for $C_{12}H_{17}O_1F_3Si_1$, 262.0995; found, 262.0993.

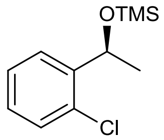

(*S*)-**3q**

New compound: colorless liquid; TLC (acetone : n-hexane, 1:20 v/v): R_f = 0.73; $[\alpha]_D^{20}$ = −49.0 (c = 0.1 in $CHCl_3$); ^{1}H NMR (500 MHz, $CDCl_3$): δ 7.61–7.59 (m, 1H), 7.29–7.25 (m, 2H), 7.18–7.14 (m, 1H), 5.22 (q, J = 6.5 Hz, 1H), 1.40 (d, J = 6.0 Hz, 3H), 0.08 (s, 9H); ^{13}C NMR (125 MHz, $CDCl_3$): δ 144.03, 130.75, 128.96, 127.88, 127.14, 126.95, 67.16, 25.35, −0.06; IR: 3069, 2959, 2899, 1574, 1473, 1439, 1370, 1252, 1203, 1099, 1029, 957, 840, 752, 691 cm^{-1}; HRMS (m/z, EI): $[M]^+$ calcd. for $C_{11}H_{17}O_1Cl_1Si_1$, 228.0732; found, 228.0730.

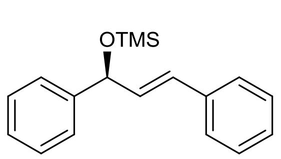

(*S*)-**3r**

Colorless liquid[3]; TLC (acetone : n-hexane, 1:20 v/v): R_f = 0.66; ^{1}H NMR (500 MHz, $CDCl_3$): δ 7.40–7.29 (m, 8H), 7.26–7.19 (m, 2H), 6.63–6.58 (m, 1H), 6.33–6.27 (m, 1H), 5.33 (d, J = 6.5 Hz, 1H), 0.14 (s, 9H); ^{13}C NMR (125 MHz, $CDCl_3$): δ 143.50, 136.81, 132.79, 129.24, 128.49, 128.29, 127.49, 127.21, 126.54, 126.19, 75.49, 0.28.

Analytical data of the *meso*-diols (4a–4c)

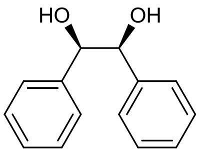

4a

White solid[15]; TLC (acetone : n-hexane, 1:2 v/v): R_f = 0.31; ^{1}H NMR (500 MHz, $CDCl_3$): δ 7.29–7.24 (m, 10H), 4.81 (s, 2H), 2.26 (s, 2H); ^{13}C NMR (125 MHz, $CDCl_3$): δ 139.71, 128.20, 128.08, 127.05, 78.05.

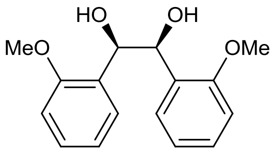

4b

White solid[16]; TLC (acetone : n-hexane, 1:2 v/v): R_f = 0.28; ^{1}H NMR (500 MHz, $CDCl_3$): δ 7.23–7.21 (m, 2H), 7.17–7.15 (m, 2H), 6.90–6.89 (m, 2H), 6.82–6.80 (m, 2H), 5.26–5.23 (m, 2H), 3.69 (s, 6H), 3.11–3.08 (m, 2H); ^{13}C NMR (125 MHz, $CDCl_3$): δ 156.88, 128.55, 128.49, 128.26, 120.48, 110.22, 73.78, 55.24.

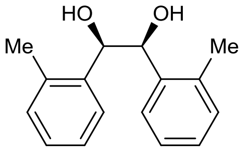

4c

White solid[16]; TLC (acetone : n-hexane, 1:2 v/v): R_f = 0.24; ^{1}H NMR (500 MHz, $CDCl_3$): δ 7.36–7.33 (m, 2H), 7.20–7.17 (m, 4H), 7.10–7.08 (m, 2H), 5.22–5.19 (m, 2H), 2.14–2.11 (m, 2H); ^{13}C NMR (125 MHz, $CDCl_3$): δ 138.16, 136.19, 130.10, 127.80, 126.64, 126.12, 73.42, 19.21.

Analytical data of the TMS-ether products (5a–5c)

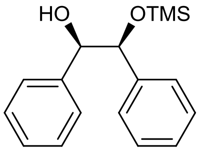

5a

Colorless liquid[17]; TLC (acetone : n-hexane, 1:5 v/v): R_f = 0.59; ^{1}H NMR (500 MHz, $CDCl_3$): δ 7.29–7.19 (m, 10H), 4.72–4.70 (m, 1H), 4.67–4.65 (m, 1H), 2.27 (d, J = 3.5 Hz, 1H), −0.12 (s, 9H); ^{13}C NMR (125 MHz, $CDCl_3$): δ 140.64, 127.85, 127.73, 127.67, 127.48, 127.26, 127.11, 79.05, 78.46, 77.21, −0.28.

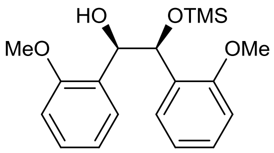

5b

New compound: colorless liquid; TLC (acetone : n-hexane, 1:5 v/v): R_f = 0.54; $[\alpha]_D^{20}$ = +42.0 (c = 0.1 in benzene); ^{1}H NMR (500 MHz, $CDCl_3$): δ 7.35–7.33 (m, 1H), 7.17–7.13 (m, 2H), 6.95–6.94 (m, 1H), 6.90–6.86 (m, 1H), 6.80–6.76 (m, 2H), 6.69–6.65 (m, 1H), 5.53–5.50 (m, 1H), 5.11–5.07 (m, 1H), 3.77 (s, 3H), 3.49 (s ,3H), 3.20 (d, J = 6.5 Hz, 1H), −0.02(s, 9H); ^{13}C NMR (125 MHz, $CDCl_3$): δ 156.99, 156.27, 129.24, 128.90, 128.13, 128.10, 127.88,

127.83, 120.02, 119.94, 109.69, 109.47, 74.06, 69.87, 55.26, 55.05, −0.25; IR: 2956, 2835, 1602, 1588, 1491, 1462, 1438, 1286, 1242, 1188, 1112, 1073, 1049, 1030, 901, 840, 751, 661 cm^{-1}; HRMS (m/z, FAB): $[M]^+$ calcd. for $C_{19}H_{25}O_4Si_1$, 345.1516; found, 345.1517.

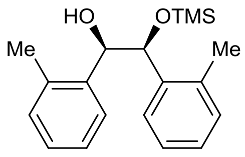

5c

New compound: colorless liquid; TLC (acetone : n-hexane, 1:5 v/v): R_f = 0.51; $[\alpha]_D^{20}$ = +48.0 (c = 0.1 in benzene); ^{1}H NMR (500 MHz, $CDCl_3$): δ 7.43–7.41 (m, 1H), 7.25–7.03 (m, 7H), 5.08–5.06 (m, 1H), 5.01–4.99 (m, 2H), 2.29 (s, 3H), 2.15 (s, 3H), −0.16 (s, 9H); ^{13}C NMR (125 MHz, $CDCl_3$): δ 139.72, 139.53, 136.48, 135.94, 130.14, 129.97, 128.08, 127.70, 127.57, 126.76, 126.16, 74.57, 19.83, 19.60, −0.01; IR: 3059, 3025, 2956, 1490, 1462, 1378, 1251, 1179, 1111, 1083, 1047, 945, 889, 841, 749, 737, 610 cm^{-1}; HRMS (m/z, FAB): $[M + Na]^+$ calcd. for $C_{19}H_{26}O_2Si_1Na_1$, 337.1600; found, 337.1600.

General procedure for silylative kinetic resolution of racemic alcohols

On a 1 mmol scale (Figure 4)

Catalyst stock solution: 11.9 mg of **1e** in 50 mL of CH_2Cl_2

To a solution of the catalyst stock solution (0.5 mL = 0.119 mg, 0.01 mol%) and racemic secondary alcohol **2a** (172.2 mg, 1.0 mmol) in CH_2Cl_2 (4.5 mL), spray dried KF (58.1 mg, 100 mol%) and Amberite CG 50 (80 mg, 10 mmol H^+ per gram) were added in one portion. The reaction mixture was stirred at –30 °C for 20 min, followed by adding hexamethyldisilazane (0.15 mL, 0.7 equiv.) to the reaction mixture. After stirring for 24 h at –30 °C, the reaction mixture was filtered, and the filtrate was concentrated in vacuo to afford a colorless oil. The residue thus obtained was purified by short silica gel chromatography (EA/hexanes = 1/5) and analyzed by chiral HPLC (Chiralcel OD-H, hexanes/IPA = 90/10, 0.7 mL/min, 220 nm). The enatiomeric excess of TMS-ether product **3a** was determined after the deprotection of its trimethylsilyl group with TBAF.

The absolute configurations of remaining alcohols **2a–2r** were determined by comparison of the retention time of HPLC with the literature data[3,7–11]. Silyl ethers **3a**–**3f, 3i**–**3j, 3m**–**3o** and **3r** are known compounds in literature, and their spectroscopic data were consistent with previously reported values[3,12–14].

Selectivity factors (k_{rel}) were calculated according to Kagan's equation[1]: $k_{rel} = \ln((1-c)(1-ee_{rsm}))/\ln((1-c)(1+ee_{rsm})) = \ln(1-c(1+ee_{prod}))/\ln(1-c(1-ee_{prod}))$, wherein c is conversion of the reaction, ee_{prod} is the enantiomeric excess of the silyl ether product and ee_{rsm} is the enantiomeric excess of the recovered alcohol. Conversions (c) were calculated by the following equation: $c = ee_{rsm}/(ee_{prod}+ee_{rsm})$.

On a 10 mmol scale (Figure 5a)

Catalyst stock solution: 1.2 mg of **1e** in 250 mL of CH_2Cl_2

To a solution of the catalyst stock solution (2.5 mL = 0.012 mg, 0.0001 mol%) and racemic secondary alcohol **2a** (1722 mg, 10 mmol) in CH_2Cl_2 (22.5 mL), spray dried KF (290 mg, 50 mol%) and Amberite CG 50 (200 mg, 10 mmol H^+ per gram) were added in one portion. The reaction mixture was stirred at −15 °C for 20 min followed by adding HMDS (1.5 mL, 0.7 equiv) to the reaction mixture. After stirring for 14 d at −15 °C, the reaction mixture was filtered, and the filtrate was concentrated in vacuo to afford a colorless oil. The crude product was purified by short silica gel chromatography (EA/hexanes = 1/5) and analyzed by chiral HPLC (Chiralcel OD-H, hexanes/IPA = 90/10, 0.7 mL/min, 220 nm) to afford 900 mg of silyl ether (*S*)-**3a** (41% yield, 94 % *ee*) and 774 mg of recovered alcohol (*R*)-**2a** (44% yield, 77% *ee*) as colorless oils. The selectivity factor was 77 at 45.0% conversion.

General procedure for silylative desymmetrization of *meso*-diols

To a solution of chiral catalyst **1e** (1.2 mg, 1.0 mol%) and *mseo*-diol **4a** (21.4 mg, 0.1 mmol) in distilled dichloroethane (2.0 mL), spray dried KF (5.8 mg, 100 mol%) and Amberite CG 50 (6.0 mg) were added in one portion. The reaction mixture was stirred at 20 °C for 10 min, followed by slow addition of HMDS (0.04 mL, 2.0 equiv) to the reaction mixture. After stirring for 48 h at 20 °C, the reaction mixture was filtered, and the filtrate was concentrated in vacuo to afford a colorless oil. The conversion of the reaction was determined from ^{1}H-NMR spectrum of the residue, (95.5% conv.); mono-silyl ether **5a**/di-silyl ether = 94/6.; The product was purified by silica gel chromatography (dichloromethane/hexanes = 1/20) and analyzed by chiral HPLC (Chiralcel OD-H, hexanes/IPA = 98/2, 1.0 mL/min, 220 nm) to afford 25.8 mg of silyl ether **5a** (90% yield, 92:8 e.r.) as a colorless oil.

Determination of the absolute configuration of 5a

The absolute configuration of **5a** was established to be (*S*)-1-silyloxy-(*R*)-2-hydroxy-1,2-diphenylethane by its conversion to the corresponding (*R*)-1-acetoxy-(*S*)-2-hydroxy-1,2-diphenylethane[15,16,18]. The absolute configuration of TMS-ether **5b** and **5c** were assigned by analogy.

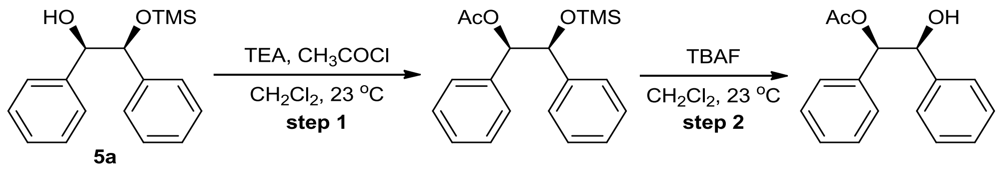

Supplementary References

1. Kagan, H. B. & Fiaud, J. C. *Topics in Stereochemistry,* Wiley, New York, **18**, 249–331 (John Wiley & Sons, 1988).
2. Yan, H., Oh, J. S. & Song, C. E. A mild and efficient method for the selective deprotection of silyl ethers using KF in the presence of tetraethylene glycol. *Org. Biomol. Chem.* **9**, 8119–8121 (2011).
3. Yan, H. *et al*. A chiral–anion generator: application to catalytic desilylative kinetic resolution of silyl-protected secondary alcohols. *Angew. Chem. Int. Ed.* **49**, 8915–8917 (2010).
4. Yan, H. *et al*. Scalable organocatalytic asymmetric Strecker reactions catalysed by a chiral cyanide generator. *Nat. Commun*. 3:1212 doi: 10.1038ncomms/2216 (2012).
5. Broeker, J., Knollmueller, M. & Gaertner, P. Chiral linker. Part 3: Synthesis and evaluation of aryl substituted *m*-hydrobenzoins as solid supported open chain chiral auxiliaries for the diastereoselective reduction of α-keto esters. Tetrahedron: Asymmetry. **17**, 2413–2429 (2006).
6. Le, P. Q., Nguyen, T.S. & May, J. A General Method for the Enantioselective Synthesis of α-Chiral Heterocycles. A. *Org. Lett.* **23**, 6104–6107 (2012).
7. Sauer, D.C., Wadepohl, H. & Gade, L. H. Cobalt Alkyl Complexes of New Family of Chiral 1,2-Bis(2-pyridylimino)isoindolates and Their Application in Asymmetric Hydrosilylation. *Inorg. Chem*. **51**, 12948–12958 (2012).
8. Jiang, F., Yuan, L., Achard, M. & Bruneau, C. Ruthenium-Containing Phosphinesulfonate Chelate for the Hydrogenation of Aryl Ketones. *Chem. Eur. J*. **19**, 10343–10352 (2013).
9. Wang, R. *et al.* Anchored $[RhCl_2(p\text{-cymene})]_2$ in hybrid zirconium phosphate-phosphonate coated and pillared with double-stranded hydrophobic linear polystyrene as heterogeneous catalyst suitable for aqueous asymmetric transfer hydrogenation. *Dalton Trans.* **42**, 6513–6522 (2013).
10. Nie, H. *et al.* Asymmetric hydrogenation of aromatic ketones using an iridium(I) catalyst containing ferrocene-based P-N-N tridentate ligands. *Tetrahedron: Asymmetry*. **24**, 1567–1571 (2013).
11. Martins, J. E. D. & Wills, M. Ir(Ⅲ) complex of diamine ligands for asymmetric ketone hydrogenation. *Tetrahedron*. **65**, 5782–5786 (2009).

12. Jalsovszky, G. *et al.* GC-IR STUDY OF TRIMETHYLSILYL ETHERS. *J. Mol. Struct.* **143**, 533–536 (1986).
13. Rajagopal, G., Lee, H. & Kim, S. S. Nafion® SAC-13: heterogeneous and reusable catalyst for the activation of HMDS for efficient and selective O-silylation reactions under solvent-free condition. *Tetrahedron*. **65**, 4735–4741 (2009).
14. Manju, K. & Trehan, S. Origin of diastereoselectivity in the addition of allylsilane to chiral acyclic mixed acetals. *Chem. Commun.* **19**, 1929–1930 (1999).
15. Muller, C. E., Zell, D. & Schreiner. P. R. One-Pot Desymmetrization of *meso*-1,2-Hydrocarbon Diols through Acylation and Oxidation. *Chem. Eur. J.* **15**, 9647–9650 (2009).
16. Wallace, T. W. *et al*. Heterodiene cycloadditions of C_2 symmetric 4,5-disubstituted ketene acetals: the net asymmetric conjugate addition of recyclable acetic ester enolate equivalents to an activated enone. *J. Chem. Soc. Perkin Trans. 1*, **18**, 2293–2308 (1995).
17. Svatos, A. & Boiand, W. Reductive Pinacol-Coupling of Aromatic Carbonyl Compounds Catalytic in Chromium(Ⅱ). *Synlett*. **5**, 549–551 (1998).
18. Nicolosi, G., Patti, A., Piattelli, M. & Sanfilippo, C. Desymmetrization of *meso*-Hydrobenzoin *via* Stereoselective Enzymatic Esterification. *Tetrahedron: Asymmetry*, **5**, 283–288 (1994).
